# Assessing the non-inferiority of prosthesis constructs used in total and unicondylar knee replacements using data from the National Joint Registry of England, Wales, Northern Ireland and the Isle of Man: a benchmarking study

**DOI:** 10.1136/bmjopen-2018-026736

**Published:** 2019-04-29

**Authors:** Kevin C Deere, Michael R Whitehouse, Martyn Porter, Ashley W Blom, Adrian Sayers

**Affiliations:** 1 Musculoskeletal Research Unit, Translational Health Sciences, University of Bristol, Bristol, UK; 2 National Institute for Health Research, Bristol Biomedical Research Centre, University Hospitals Bristol NHS Foundation Trust and University of Bristol, Bristol, UK; 3 Centre for Hip Surgery, Wrightington Hospital, Wigan, UK; 4 Population Health Sciences, Bristol Medical School, University of Bristol, Bristol, UK

**Keywords:** knee arthroplasty, non-inferiority, national joint registry, benchmarking, medical devices

## Abstract

**Objectives:**

To investigate the relative performance of knee replacement constructs compared with the best performing construct and illustrate the substantial variability in performance.

**Design:**

A non-inferiority study.

**Setting:**

England and Wales.

**Participants:**

All primary total and unicondylar knee replacements performed and registered in the National Joint Registry between 1 April 2003 and 31 December 2016.

**Main outcome measures:**

Kaplan-Meier failure function for knee replacement constructs. Failure difference between best performing construct (the benchmark) and other constructs.

**Methods:**

Using a non-inferiority analysis, the performance of knee replacement constructs by brand were compared with the best performing construct. Construct failure was estimated using the 1-Kaplan Meier method, that is, an estimate of net failure. The difference in failure between the contemporary benchmark construct and all other constructs were tested.

**Results:**

Of the 449 different knee replacement constructs used, only 27 had ≥500 procedures at risk at 10 years postprimary, 18 of which were classified as inferior to the benchmark by at least 20% relative risk of failure. Two of these 18 were unicondylar constructs that were inferior by at least 100% relative risk. In men, aged 55–75 years, 12 of 27 (44%) constructs were inferior by at least 20% to the benchmark at 7 years postprimary. In women, aged 55–75 years, 8 of 32 (25%) constructs were inferior at 7 years postprimary. Very few constructs were classified as non-inferior to the contemporary benchmark.

**Conclusions:**

There are few knee replacement constructs that can be shown to be non-inferior to a contemporary benchmark. Unicondylar knee constructs have, almost universally, at least 100% worse revision outcomes compared with the best performing total knee replacement. These results will help to inform patients, clinicians and commissioners when considering knee replacement surgery.

**Strengths and limitations of this study**

- Data presented from the largest joint registry in the world.
- A novel and systematic comparison of the performance of knee constructs to a contemporary benchmark knee construct.
- Unambiguous presentation of data will facilitate the consenting process for patients and allows surgeons and policy makers to be more informed with respect to success and failure of different constructs options available in knee replacement.
- Residual and unmeasured confounding factors are likely to be present.
- Potential for selection bias whereby certain constructs may be implanted for particular indications and in particular groups with different risks.

## Introduction

Over 90 000 knee replacements are performed annually in England and Wales, and there is a bewildering choice of total and unicondylar knee replacement (UKR) options available from which clinicians and patients can choose. When patients are considering a knee replacement, it is understandable that many assume that the different constructs function equally. However, all constructs are not equal as evidenced by variation in revision rates between brands and knee construct types.^1^ The National Joint Registry for England, Wales, Northern Ireland and the Isle of Man (NJR) is the largest arthroplasty database in the world and publishes the unadjusted cumulative failure rates of the most commonly used constructs in knee replacement surgery. This is a useful format for measuring absolute failure but does not allow easy head-to-head comparison of constructs. Benchmarking bodies such as the Orthopaedic Device Evaluation Panel (ODEP) in the UK,^2^ NOV in the Netherlands^3^ and the Australian superior clinical performance programme^4^ compare construct performance against externally set benchmarks but do not perform head-to-head comparison. Although it is reassuring a certain standard has been met, this simple dichotomisation does not facilitate head-to-head comparison and the sample sizes used are arbitrarily set. Sayers *et al* 5 recently proposed a method of comparison for joint replacement constructs using a non-inferiority design against an external benchmark. However, the primary limitation of this method remains the arbitrary requirement for an externally specified benchmark.

In a non-inferiority clinical trial,^6^ investigating failure, two treatments (comparator and reference) can be directly compared to ensure that the comparator treatment is within a clinically acceptable range (non-inferiority margin) of performance at a specified point in time.^7 8^ Standard methods for assessing non-inferiority could be applied in an orthopaedic benchmarking setting, assuming an appropriate comparator, non-inferiority margin and time of interest can be identified. This is a method we have applied in a medical device setting, namely, hip replacements using NJR data, in which we assessed the non-inferiority of hip replacement constructs as compared with a benchmark construct.^9^

Choosing an appropriate outcome and contemporary reference is difficult. There is no single outcome, no gold standard or evidence from randomised trials that suggests any construct outperforms all others; therefore, the choice is more heuristic. Patients would like to receive the best available care and clinicians would like to provide the best possible care, or at least care that is non-inferior to the best. A binary, unambiguous, endpoint such as revision surgery represents one potential outcome of interest. Therefore, the natural choice of reference is the construct with the lowest failure rate. However, in order to protect against chance, the construct should be used in large enough numbers to mitigate sampling variability. The failure rate of a construct is influenced by both age and gender; therefore, the choice of reference should reflect this specificity. The selection of an appropriate time and non-inferiority margin to assess construct performance is more subjective. For example, construct survivorship in the long term is less relevant to older patients with shorter life expectancy, where improved quality of life, reduction in pain or improved physical functioning maybe more relevant.

The aim of this study is to investigate the relative performance of knee replacement constructs as compared with the best performing contemporary construct, the benchmark, using a non-inferiority study design and to illustrate the substantial variability in performance of widely used constructs. This research focuses on total knee replacements (TKRs) and unicondylar knee replacements (UKRs) as these are commonly used and therefore there is sufficient data to make robust comparisons. TKRs and UKRs are examined against non-inferiority margins of 20% relative risk and 100% relative risk at 3, 5, 7 and 10 years following surgery. This is predicated on our belief that patients would at least want reassurance that the construct they are to receive is not estimated to be 100% worse than the best alternatives when used in patients with the same gender and age as them.

## Methods

### Patients and data sources

We identified all patients with a primary TKR or UKR registered in the NJR between 1 April 2003 and 31 December 2016. All patients were consented to be included in the NJR as part of the standard NJR process.

Procedures were excluded if the patient age or gender were missing, or the National Health Service number was untraceable and therefore mortality unknown. Procedures where the constraint or fixation type were unknown were excluded from the analysis. Patellofemoral replacements were also removed owing to the low number in the sample.

Brands of constructs are usually subdivided by fixation, mobility of the bearing and degree of constraint. NJR Annual Report data have shown that these characteristics influence revision rates, and thus, we treated each subdivision as a separate construct.

### Patient involvement

Patient representatives sit on the committee structure of the NJR. The research priorities of the NJR are identified by this committee structure and approved by the patient representatives. Patients were not involved in the setting of the research question or the outcome measures, nor were they involved in designing or implementing this work or interpretation of the results. We are unable to disseminate results of this study directly to study participants due to the anonymous nature of the data. We plan to disseminate our findings to the NJR, via their communications team, to consultations relevant to the provision of joint replacement and to the general population through the local and national press.

### Statistical methods

Using a non-inferiority analysis, the performance of knee constructs were compared with an internally identified benchmark group. Construct failure was estimated using the 1-Kaplan Meier method, that is, an estimate of net failure, which is appropriate when the risk of revision is considered.^10^

Failure is defined using the first linked surgical revision, where revision was defined as any addition, removal or modification of an implant to a joint that had previously undergone a TKR or UKR. Patients were censored at death or administratively censored on 31 December 2016. The difference in stratum specific failure probabilities compared with the benchmark were calculated at 3, 5, 7 and 10 years for all constructs, stratified by gender, and stratified by gender and age group (<55 years, 55–75 years and >75 years).

The difference and 95% CI of the difference between the comparator construct and the benchmark construct was estimated at the specified time points. We used a 95% CI, as is the convention in the majority of medical research. The SE of the difference was constructed using a pooled estimate of the Greenwood SE^11^

$$\hat{SE\left(Diff\right)}=\sqrt{GSE_{xi}^{2}\;+\;GSE_{ref}^{2}},$$

and a z-test comparing the difference between the benchmark and test construct was then constructed using,

$$Z=\left(\left(\hat{F_{xi}}-\hat{F_{ref}}\right)+\delta \right)/\hat{SE\left(Diff\right)}.$$

The stratum specific contemporaneous benchmark construct was selected as the knee construct (TKR or UKR) with the lowest failure rate with at least 1000 patients at risk at the time point of interest. The choice of 1000 procedures of the same construct was based on simulation work by Sayers *et al*, which demonstrated that 1000 procedures at risk will give rise to a CI width of approximately 3% (±1.5%).^5^ We believe this is the minimal level of accuracy to be considered a suitable reference standard.

Two non-inferiority margins were chosen to illustrate the sensitivity of the choice. The first margin was conservatively set at a 20% increase in relative risk of failure compared with the benchmark, in line with clinical trials using this methodology, although towards the upper end.^12^ The second was a 100% increase in relative risk, that is, a doubling in cumulative probability of failure, as this is an easily interpretable outcome.

Results are graphically reported for all comparator constructs with at least 500 patients still at risk at the beginning of the time point of interest. Results are also reported in a tabular format for all comparator constructs with at least 250 patients at risk at the beginning of the time point of interest (see online supplementary tables).

10.1136/bmjopen-2018-026736.supp1 Supplementary file 1

**Figure 1:**
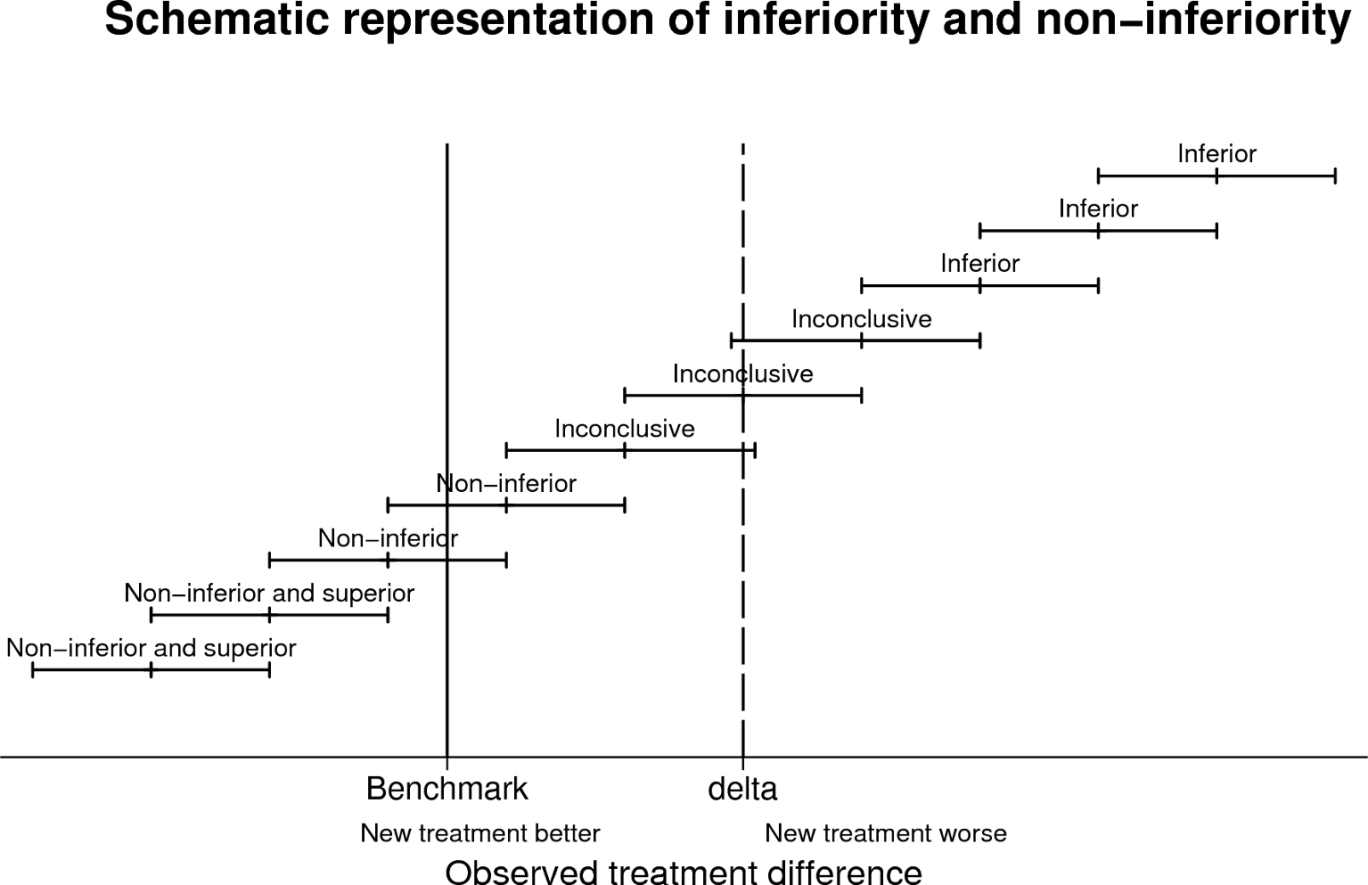
Schematic representation of inferiority and non-inferiority.

Constructs were either classified as non-inferior, inconclusive or inferior. If the upper CI is less than or equal to the 20% non-inferiority margin, the construct was non-inferior. If the lower CI of the difference was greater than the non-inferiority margin at either 20% or 100%, the construct was classed as inferior at 20% or 100%, respectively. If the lower confidence limit is less than the non-inferiority margin, and the upper confidence is greater than non-inferiority margin, the evidence against the construct was described as inconclusive (see figure 1 for graphical representation of the classification). All analyses were carried out using Stata V.15.1.

### Sensitivity analysis

We repeated all analyses using the best performing knee construct at 10 years with at least 1000 still at risk in the stratum of interest as the benchmark at the 3, 5 and 7 year time points.

## Results

There were 975 739 primary knee replacements included in the NJR between 1 April 2003 and 31 December 2016, using 649 different combinations of brand, fixation, constraint and bearing type. Following the application of the exclusion criteria (see online supplementary figure 1), 947 686 procedures were included in the final analysis (863 551 [91.1%] TKRs), using 449 different combinations of brand, fixation, constraint and bearing type (405 TKRs and 44 UKRs).

A detailed description of non-inferiority across all procedures is provided. Due to the large number of clinically relevant subdivisions and sensitivity analyses, results will be described more broadly. Each stratification of age group, gender and time point of interest are provided in online supplementary material.

Constructs are described by brand, fixation, the degree of constraint for TKR and whether the bearing was fixed or mobile. Constraint types were either unconstrained (cruciate retaining) or posterior stabilised (posterior cruciate sacrificing). The vast majority of benchmark constructs were cemented. In each subdivision of our analyses, the construct that met our benchmark criteria was a TKR that was unconstrained with a fixed bearing. However, not all total knees that were unconstrained with fixed bearings performed well as there were 15 separate brands of this type that were found to be inferior to the benchmark by at least 20% relative risk in at least one subdivision analysis.

### Non-inferiority: all procedures

The benchmark construct at 3 years was identified as the NexGen cemented, unconstrained TKR with a fixed bearing. There were 34 558 procedures remaining at risk at 3 years for this construct, and the failure rate was 1.10% (95% CI 1.01 to 1.20). There were 73 constructs with ≥500 procedures at risk. Thirty-nine constructs were classified as inferior to the benchmark by at least 20% relative risk of failure. Nine of the 73 were shown to be inferior by at least 100% relative risk (online supplementary figure 2). One prosthesis, the NexGen TKR with a monobloc polyethylene tibia, was non-inferior. The remaining 32 constructs were classified as non-inferiority not shown.

**Figure 2:**
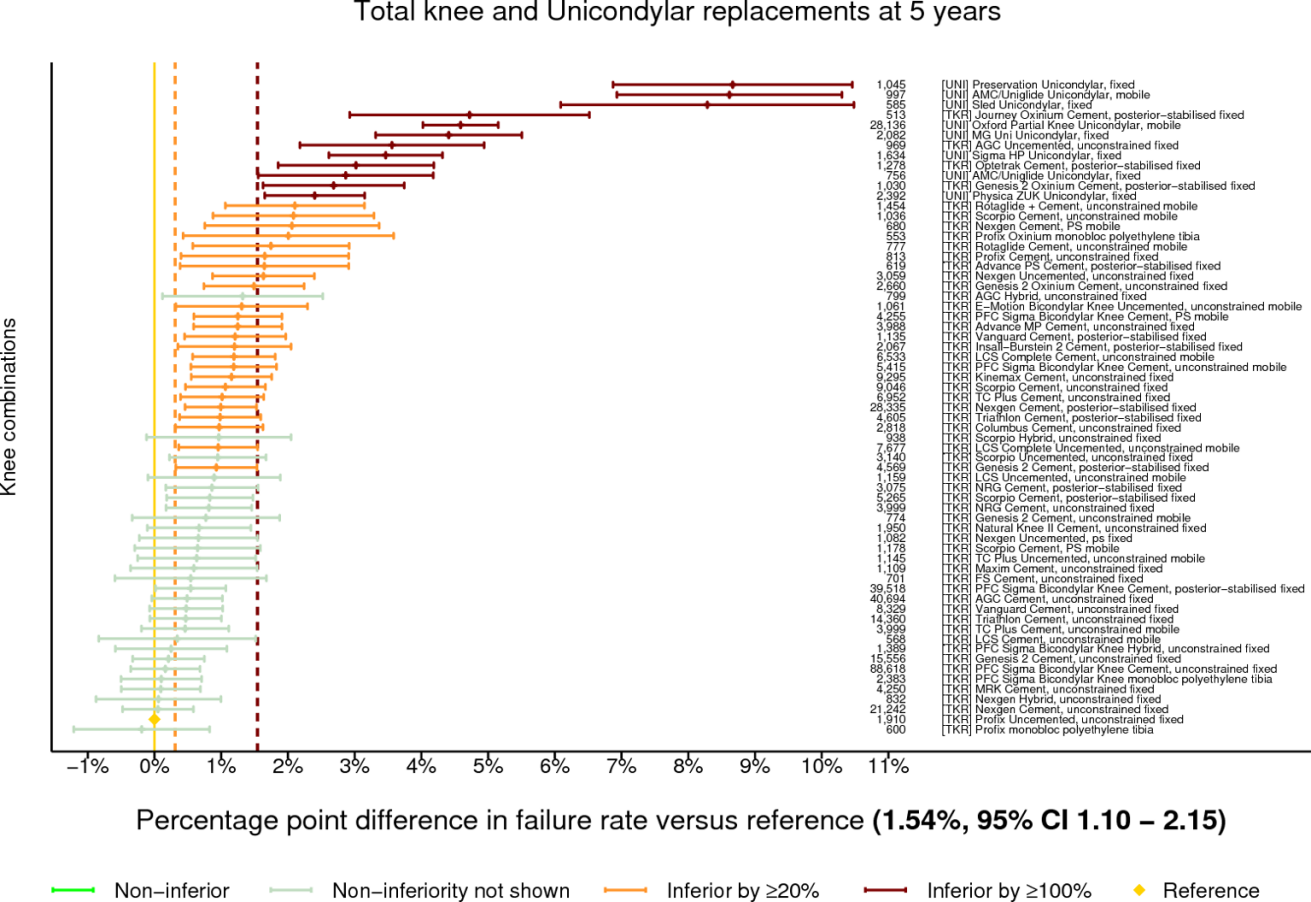
Difference in cumulative revision of knee constructs compared with a contemporary benchmark at 5 years, using all total knee and unicondylar replacements with ≥500 procedures remaining at risk.

The benchmark construct at 5 years was identified as the Profix uncemented unconstrained TKR with a fixed bearing. There were 1910 procedures remaining at risk and the failure rate was 1.54% (95% CI 1.10 to 2.15). There were 65 constructs with ≥500 procedures at risk. Thirty-six were classified as inferior to the benchmark by at least 20% relative risk of failure. Twelve of the 36 were shown to be inferior by at least 100% relative risk (figure 2). All of the UKRs with ≥500 procedures at risk (n=8) were inferior by at least 100% relative risk. No construct could be described as non-inferior.

The benchmark construct at 7 years was identified, again, as the Profix uncemented unconstrained TKR with a fixed bearing. There were 1501 procedures remaining at risk and the failure rate was 1.77% (95% CI 1.29 to 2.43). There were 57 constructs with ≥500 procedures at risk. Thirty constructs were classified as inferior to the benchmark by at least 20% relative risk of failure. Eight of the 30 were shown to be inferior by at least 100% relative risk (online supplementary figure 3). All of the UKR constructs with ≥500 procedures at risk (n=5) were inferior by at least 100% relative risk. No construct could be described as non-inferior.

**Figure 3:**
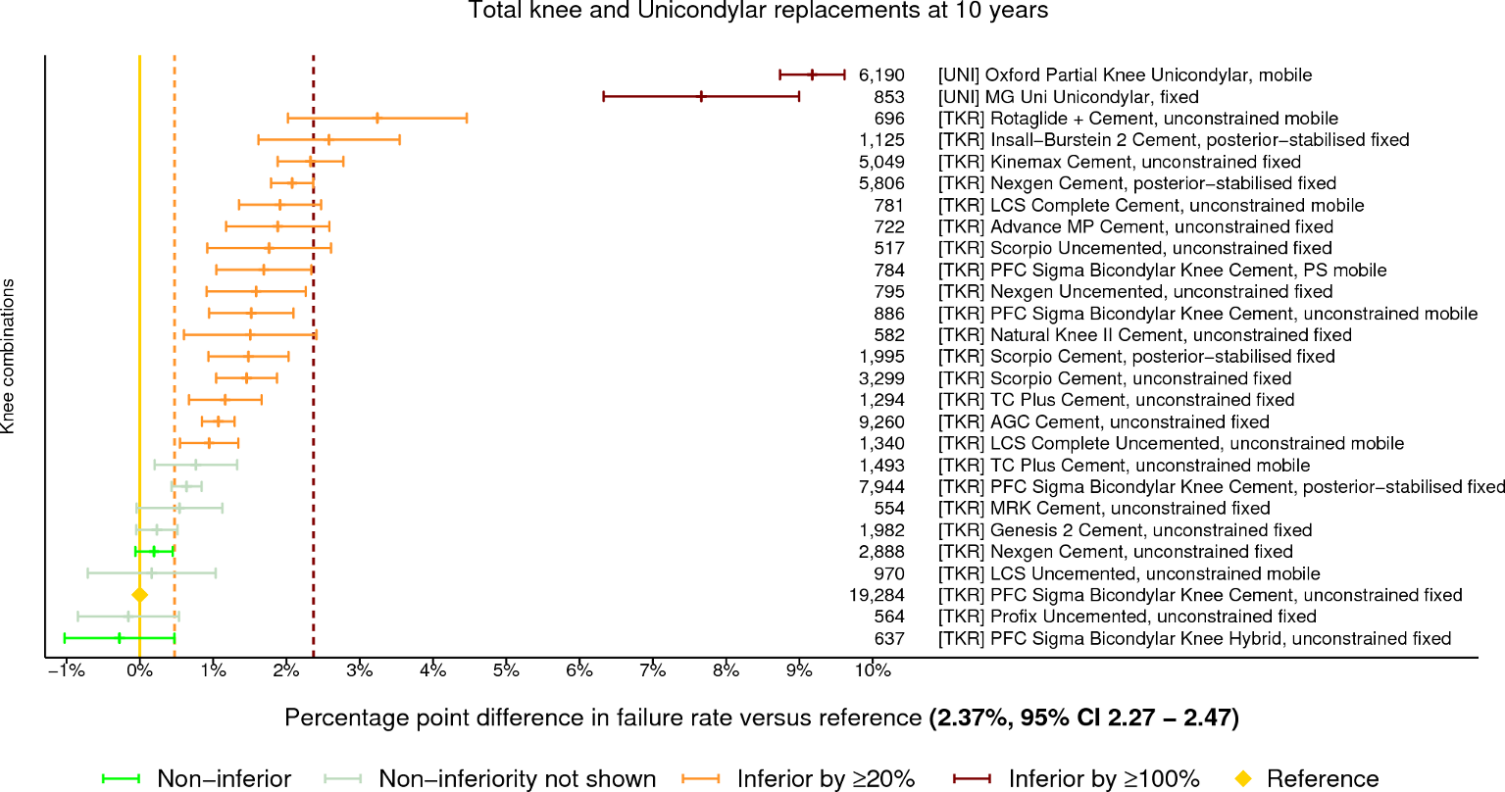
Difference in cumulative revision of knee constructs compared with a contemporary benchmark at 10 years, using all total knee and unicondylar replacements with ≥500 procedures remaining at risk.

The benchmark construct at 10 years was identified as the PFC Sigma Bicondylar Knee cemented unconstrained TKR with a fixed bearing. There were 19 284 procedures remaining at risk, and the failure rate was 2.37% (95% CI 2.27 to 2.47). There were 27 constructs with ≥500 procedures at risk. Eighteen constructs were classified as inferior to the benchmark by at least 20% relative risk of failure. Two of the 18 were shown to be inferior by at least 100% relative risk (figure 3). There were only two UKRs with ≥500 procedures at risk at 10 years, both of which were inferior to the benchmark by at least 100% relative risk. Two constructs were identified as non-inferior, the NexGen cemented unconstrained TKR with fixed bearing and the TKR PFC Sigma Bicondylar Knee hybrid uncemented with a fixed bearing.

Estimates for the difference in failure between the benchmark and comparator constructs with ≥250 procedure at risk at the time of interest are shown in online supplementary tables 1–4.

### Non-inferiority: gender specific

Gender specific non-inferiority analyses were also performed at 3, 5, 7 and 10 years after the primary operation.

At 3 years, there were no constructs that demonstrated non-inferiority in comparison with the benchmark prosthesis. Most striking is the general similarity in constructs used and their performance regardless of gender. There were 56 different constructs with at least 500 procedures still at risk in women versus 50 in men. There were 25 constructs with at least a 20% increase in relative risk in women versus 18 in men, although the increased number of inferior constructs demonstrated in women is likely owing to the slightly better performing benchmark group than in men. Three constructs were inferior by at least 100% in men, each a UKR, and eight constructs were inferior by at least 100% in women (six UKRs and two TKRs).

At 5 years, in women, there were 49 constructs with ≥500 procedures at risk. Twenty-nine of these constructs were classified as inferior to the benchmark by at least 20% relative risk of failure. Six of the 29 were shown to be inferior by at least 100% relative risk, four of which were UKRs. Similarly, in men, there were 43 constructs used with 19 inferior by at least a 20% increase in relative risk. Four of the 19 were inferior by at least 100% relative risk, all of which were UKRs. Results for men and women at 5 years with ≥250 procedures at risk can be seen in tables 1 and 2, respectively.

**Table 1:** Difference in cumulative percentage revision of knee constructs compared with a contemporary benchmark at 5 years postprimary in men for knee replacements with ≥250 procedures remaining at risk

Knee brand, bearing and constraint	Number at risk	Cumulative failure (%)	Difference in failure (%)	95% CI	Equivalence status	P value
(TKR) NexGen cemented, unconstrained fixed	8639	1.76	(Reference)			
(TKR) AGC cemented, unconstrained fixed	16 871	2.28	0.52	(0.24 to 0.80)	Non-inferiority not shown	<0.001
(TKR) AGC hybrid, unconstrained fixed	369	2.59	0.83	(−0.70 to 2.36)	Non-inferiority not shown	0.143
(TKR) AGC uncemented, unconstrained fixed	468	4.87	3.11	(1.33 to 4.88)	Inferior by ≥20%	<0.001
(TKR) Advance MP cemented, unconstrained fixed	1725	3.18	1.42	(0.74 to 2.11)	Inferior by ≥20%	<0.001
(TKR) Advance PS cemented, posterior-stabilised fixed	266	3.48	1.72	(−0.11 to 3.55)	Non-inferiority not shown	0.033
(TKR) Columbus cemented, unconstrained fixed	1224	2.49	0.73	(0.08 to 1.38)	Non-inferiority not shown	0.013
(TKR) E-Motion Bicondylar Knee uncemented, unconstrained mobile	519	2.49	0.73	(−0.41 to 1.88)	Non-inferiority not shown	0.105
(TKR) FS cemented, unconstrained fixed	305	1.76	0.00	(−1.41 to 1.40)	Non-inferiority not shown	0.497
(TKR) Genesis 2 cemented, posterior-stabilised fixed	1723	2.97	1.21	(0.61 to 1.82)	Inferior by ≥20%	<0.001
(TKR) Genesis 2 cemented, unconstrained fixed	6545	2.17	0.41	(0.08 to 0.74)	Non-inferiority not shown	0.008
(TKR) Genesis 2 cemented, unconstrained mobile	310	3.52	1.76	(−0.13 to 3.65)	Non-inferiority not shown	0.034
(TKR) Genesis 2 Oxinium cemented, posterior-stabilised fixed	411	5.27	3.51	(1.89 to 5.13)	Inferior by ≥100%	<0.001
(TKR) Genesis 2 Oxinium cemented, unconstrained fixed	1175	3.42	1.65	(0.76 to 2.55)	Inferior by ≥20%	<0.001
(TKR) Insall-Burstein 2 cemented, posterior-stabilised fixed	892	2.88	1.12	(0.06 to 2.17)	Non-inferiority not shown	0.019
(TKR) Kinemax cemented, unconstrained fixed	3927	3.08	1.32	(0.76 to 1.87)	Inferior by ≥20%	<0.001
(TKR) LCS Complete cemented, unconstrained mobile	2774	2.88	1.12	(0.54 to 1.70)	Inferior by ≥20%	<0.001
(TKR) LCS Complete uncemented, unconstrained mobile	3472	2.36	0.60	(0.14 to 1.06)	Non-inferiority not shown	0.005
(TKR) LCS uncemented, unconstrained mobile	485	2.82	1.06	(−0.36 to 2.48)	Non-inferiority not shown	0.072
(TKR) MRK cemented, unconstrained fixed	1681	2.07	0.31	(−0.26 to 0.87)	Non-inferiority not shown	0.143
(TKR) Maxim cemented, unconstrained fixed	481	1.86	0.10	(−1.06 to 1.25)	Non-inferiority not shown	0.436
(TKR) NRG cemented, posterior-stabilised fixed	1291	2.62	0.86	(0.10 to 1.62)	Non-inferiority not shown	0.014
(TKR) NRG cemented, unconstrained fixed	1649	3.14	1.38	(0.67 to 2.09)	Inferior by ≥20%	<0.001
(TKR) Natural Knee II cemented, unconstrained fixed	767	2.02	0.26	(−0.65 to 1.16)	Non-inferiority not shown	0.288
(TKR) NexGen cemented, posterior-stabilised mobile	267	3.93	2.17	(0.20 to 4.14)	Non-inferiority not shown	0.016
(TKR) NexGen cemented, posterior-stabilised fixed	11 354	2.89	1.13	(0.81 to 1.45)	Inferior by ≥20%	<0.001
(TKR) NexGen hybrid, unconstrained fixed	379	1.47	−0.29	(−1.39 to 0.81)	Non-inferiority not shown	0.303
(TKR) NexGen uncemented, posterior-stabilised fixed	568	1.81	0.05	(−0.87 to 0.97)	Non-inferiority not shown	0.456
(TKR) NexGen uncemented, unconstrained fixed	1639	3.13	1.36	(0.59 to 2.14)	Inferior by ≥20%	<0.001
(TKR) Optetrak cemented, posterior-stabilised fixed	533	3.51	1.75	(0.29 to 3.20)	Non-inferiority not shown	0.009
(TKR) PFC Sigma Bicondylar Knee cemented, posterior-stabilised mobile	1976	2.65	0.89	(0.27 to 1.51)	Non-inferiority not shown	0.002
(TKR) PFC Sigma Bicondylar Knee cemented, posterior-stabilised fixed	15 828	2.16	0.40	(0.13 to 0.67)	Non-inferiority not shown	0.002
(TKR) PFC Sigma Bicondylar Knee cemented, unconstrained fixed	36 856	1.83	0.07	(−0.16 to 0.30)	Non-inferior	0.279
(TKR) PFC Sigma Bicondylar Knee cemented, unconstrained mobile	2565	2.75	0.98	(0.40 to 1.57)	Inferior by ≥20%	0.001
(TKR) PFC Sigma Bicondylar Knee hybrid, unconstrained fixed	619	1.86	0.10	(−0.93 to 1.12)	Non-inferiority not shown	0.428
(TKR) PFC Sigma Bicondylar Knee monobloc polyethylene tibia	898	1.69	−0.07	(−0.61 to 0.46)	Non-inferiority not shown	0.394
(TKR) Profix cemented, unconstrained fixed	360	3.97	2.21	(0.29 to 4.12)	Non-inferiority not shown	0.012
(TKR) Profix uncemented, unconstrained fixed	856	1.21	−0.55	(−1.26 to 0.16)	Non-inferior	0.064
(TKR) Rotaglide+ cemented, unconstrained mobile	610	4.40	2.64	(1.11 to 4.17)	Inferior by ≥20%	<0.001
(TKR) Rotaglide cemented, unconstrained mobile	291	4.30	2.54	(0.62 to 4.45)	Inferior by ≥20%	0.005
(TKR) Scorpio cemented, posterior-stabilised mobile	517	2.39	0.63	(−0.62 to 1.89)	Non-inferiority not shown	0.162
(TKR) Scorpio cemented, posterior-stabilised fixed	2090	2.91	1.14	(0.43 to 1.86)	Inferior by ≥20%	0.001
(TKR) Scorpio cemented, unconstrained fixed	3667	3.06	1.30	(0.75 to 1.86)	Inferior by ≥20%	<0.001
(TKR) Scorpio cemented, unconstrained mobile	436	3.94	2.18	(0.43 to 3.93)	Inferior by ≥20%	0.007
(TKR) Scorpio hybrid, unconstrained fixed	374	3.33	1.57	(−0.16 to 3.30)	Non-inferiority not shown	0.038
(TKR) Scorpio uncemented, unconstrained fixed	1440	2.50	0.74	(−0.04 to 1.51)	Non-inferiority not shown	0.031
(TKR) TC Plus cemented, unconstrained fixed	3133	3.00	1.23	(0.64 to 1.83)	Inferior by ≥20%	<0.001
(TKR) TC Plus cemented, unconstrained mobile	1758	2.61	0.84	(0.13 to 1.56)	Non-inferiority not shown	0.010
(TKR) TC Plus uncemented, unconstrained mobile	434	1.80	0.04	(−1.00 to 1.07)	Non-inferiority not shown	0.473
(TKR) Triathlon cemented, posterior-stabilised fixed	1851	2.53	0.77	(0.23 to 1.31)	Non-inferiority not shown	0.003
(TKR) Triathlon cemented, unconstrained fixed	5995	1.94	0.18	(−0.13 to 0.48)	Non-inferiority not shown	0.126
(TKR) Vanguard cemented, posterior-stabilised fixed	471	2.57	0.81	(−0.05 to 1.67)	Non-inferiority not shown	0.033
(TKR) Vanguard cemented, unconstrained fixed	3489	2.10	0.34	(−0.01 to 0.70)	Non-inferiority not shown	0.028
(UNI) AMC/Uniglide Unicondylar, fixed	348	3.97	2.21	(0.51 to 3.90)	Inferior by ≥20%	0.005
(UNI) AMC/Uniglide Unicondylar, mobile	519	8.90	7.14	(5.06 to 9.22)	Inferior by ≥100%	<0.001
(UNI) MG Uni Unicondylar, fixed	1130	5.36	3.60	(2.33 to 4.86)	Inferior by ≥100%	<0.001
(UNI) Oxford Partial Knee Unicondylar, mobile	14 581	5.72	3.96	(3.59 to 4.33)	Inferior by ≥100%	<0.001
(UNI) Physica ZUK Unicondylar, fixed	1280	3.88	2.12	(1.35 to 2.89)	Inferior by ≥20%	<0.001
(UNI) Preservation Unicondylar, fixed	566	8.55	6.79	(4.61 to 8.96)	Inferior by ≥100%	<0.001
(UNI) Sigma HP Unicondylar, fixed	948	4.36	2.59	(1.75 to 3.44)	Inferior by ≥20%	<0.001
(UNI) Sled Unicondylar, fixed	261	11.47	9.71	(6.30 to 13.12)	Inferior by ≥100%	<0.001

TKR, total knee replacement; UNI, unicondylar knee replacement.

**Table 2:** Difference in cumulative percentage revision of knee constructs compared with a contemporary benchmark at 5 years postprimary in women for knee replacements with ≥250 procedures remaining at risk

Knee brand, bearing and constraint	Number at risk	Cumulative failure (%)	Difference in failure (%)	95% CI	Equivalence status	P value
(TKR) MRK cemented, unconstrained fixed	2570	1.35	(Reference)			
(TKR) AGC cemented, posterior-stabilised fixed	264	2.65	1.31	(−0.54 to 3.16)	Non-inferiority not shown	0.083
(TKR) AGC cemented, unconstrained fixed	23 829	1.85	0.50	(0.13 to 0.88)	Non-inferiority not shown	0.004
(TKR) AGC hybrid, unconstrained fixed	431	3.11	1.76	(0.17 to 3.35)	Non-inferiority not shown	0.015
(TKR) AGC uncemented, unconstrained fixed	502	5.34	4.00	(2.10 to 5.89)	Inferior by ≥100%	<0.001
(TKR) Advance MP cemented, unconstrained fixed	2264	2.46	1.11	(0.48 to 1.75)	Inferior by ≥20%	<0.001
(TKR) Advance MP Stature cemented, unconstrained fixed	378	2.93	1.58	(0.31 to 2.86)	Inferior by ≥20%	0.007
(TKR) Advance PS cemented, posterior-stabilised fixed	354	2.97	1.62	(0.09 to 3.15)	Non-inferiority not shown	0.019
(TKR) Columbus cemented, unconstrained fixed	1595	2.53	1.19	(0.54 to 1.83)	Inferior by ≥20%	<0.001
(TKR) E-Motion Bicondylar Knee uncemented, unconstrained mobile	544	3.22	1.87	(0.57 to 3.17)	Inferior by ≥20%	0.002
(TKR) FS cemented, unconstrained fixed	397	2.34	0.99	(−0.48 to 2.47)	Non-inferiority not shown	0.093
(TKR) Genesis 2 cemented, posterior-stabilised fixed	2848	2.15	0.80	(0.29 to 1.32)	Inferior by≥20%	0.001
(TKR) Genesis 2 cemented, unconstrained fixed	9021	1.44	0.10	(−0.29 to 0.49)	Non-inferiority not shown	0.311
(TKR) Genesis 2 cemented, unconstrained mobile	464	1.48	0.14	(−0.94 to 1.22)	Non-inferiority not shown	0.401
(TKR) Genesis 2 Oxinium cemented, posterior-stabilised fixed	620	3.49	2.14	(1.00 to 3.29)	Inferior by ≥20%	<0.001
(TKR) Genesis 2 Oxinium cemented, unconstrained fixed	1485	2.75	1.41	(0.63 to 2.19)	Inferior by ≥20%	<0.001
(TKR) Insall-Burstein 2 cemented, posterior-stabilised fixed	1177	2.63	1.28	(0.33 to 2.23)	Inferior by ≥20%	0.004
(TKR) Journey Oxinium cemented, posterior-stabilised fixed	283	7.30	5.96	(3.45 to 8.47)	Inferior by ≥100%	<0.001
(TKR) Kinemax cemented, unconstrained fixed	5369	2.41	1.07	(0.54 to 1.59)	Inferior by ≥20%	<0.001
(TKR) LCS cemented, unconstrained mobile	340	1.85	0.51	(−0.90 to 1.91)	Non-inferiority not shown	0.239
(TKR) LCS Complete cemented, unconstrained mobile	3759	2.62	1.28	(0.72 to 1.84)	Inferior by ≥20%	<0.001
(TKR) LCS Complete uncemented, unconstrained mobile	4205	2.61	1.26	(0.73 to 1.80)	Inferior by ≥20%	<0.001
(TKR) LCS uncemented, unconstrained mobile	675	2.16	0.82	(−0.29 to 1.92)	Non-inferiority not shown	0.073
(TKR) Maxim cemented, posterior-stabilised fixed	282	3.24	1.90	(−0.02 to 3.82)	Non-inferiority not shown	0.026
(TKR) Maxim cemented, unconstrained fixed	629	2.34	1.00	(−0.16 to 2.15)	Non-inferiority not shown	0.045
(TKR) NRG cemented, posterior-stabilised fixed	1784	2.24	0.90	(0.22 to 1.58)	Non-inferiority not shown	0.005
(TKR) NRG cemented, unconstrained fixed	2350	1.79	0.44	(−0.11 to 1.00)	Non-inferiority not shown	0.059
(TKR) Natural Knee II cemented, unconstrained fixed	1183	2.34	1.00	(0.15 to 1.84)	Non-inferiority not shown	0.010
(TKR) NexGen cemented, posterior-stabilised mobile	414	3.39	2.04	(0.49 to 3.60)	Inferior by ≥20%	0.005
(TKR) NexGen cemented, posterior-stabilised fixed	16 989	2.30	0.95	(0.56 to 1.34)	Inferior by ≥20%	<0.001
(TKR) NexGen cemented, unconstrained fixed	12 603	1.47	0.13	(−0.25 to 0.51)	Non-inferiority not shown	0.253
(TKR) NexGen hybrid, unconstrained fixed	454	1.72	0.37	(−0.79 to 1.54)	Non-inferiority not shown	0.266
(TKR) NexGen uncemented, posterior-stabilised fixed	514	2.66	1.31	(0.10 to 2.52)	Non-inferiority not shown	0.017
(TKR) NexGen uncemented, unconstrained fixed	1421	3.25	1.90	(0.98 to 2.83)	Inferior by ≥20%	<0.001
(TKR) Optetrak cemented, posterior-stabilised fixed	746	5.29	3.94	(2.45 to 5.44)	Inferior by ≥100%	<0.001
(TKR) PFC Sigma Bicondylar Knee cemented, posterior-stabilised mobile	2280	2.92	1.57	(0.90 to 2.24)	Inferior by ≥20%	<0.001
(TKR) PFC Sigma Bicondylar Knee cemented, posterior-stabilised fixed	23 690	2.03	0.69	(0.31 to 1.06)	Inferior by ≥20%	<0.001
(TKR) PFC Sigma Bicondylar Knee cemented, unconstrained fixed	51 762	1.61	0.27	(−0.09 to 0.63)	Non-inferiority not shown	0.071
(TKR) PFC Sigma Bicondylar Knee cemented, unconstrained mobile	2852	2.72	1.37	(0.73 to 2.01)	Inferior by ≥20%	<0.001
(TKR) PFC Sigma Bicondylar Knee hybrid, unconstrained fixed	771	1.75	0.40	(−0.54 to 1.34)	Non-inferiority not shown	0.202
(TKR) PFC Sigma Bicondylar Knee uncemented, unconstrained mobile	254	1.60	0.25	(−1.07 to 1.57)	Non-inferiority not shown	0.355
(TKR) PFC Sigma Bicondylar Knee monobloc polyethylene tibia	1486	1.61	0.27	(−0.27 to 0.80)	Non-inferiority not shown	0.163
(TKR) Profix cemented, unconstrained fixed	454	2.58	1.23	(−0.19 to 2.66)	Non-inferiority not shown	0.045
(TKR) Profix Oxinium monobloc polyethylene tibia	317	3.58	2.24	(0.22 to 4.26)	Non-inferiority not shown	0.015
(TKR) Profix uncemented, unconstrained fixed	1055	1.81	0.47	(−0.36 to 1.29)	Non-inferiority not shown	0.134
(TKR) Profix monobloc polyethylene tibia	371	1.47	0.13	(−1.09 to 1.34)	Non-inferiority not shown	0.420
(TKR) Rotaglide+ cemented, unconstrained mobile	845	3.08	1.74	(0.58 to 2.89)	Inferior by ≥20%	0.002
(TKR) Rotaglide cemented, unconstrained mobile	487	2.65	1.31	(0.04 to 2.58)	Non-inferiority not shown	0.022
(TKR) Scorpio cemented, posterior-stabilised mobile	662	2.03	0.68	(−0.39 to 1.76)	Non-inferiority not shown	0.106
(TKR) Scorpio cemented, posterior-stabilised fixed	3175	2.01	0.67	(0.09 to 1.25)	Non-inferiority not shown	0.012
(TKR) Scorpio cemented, unconstrained fixed	5380	2.29	0.94	(0.43 to 1.45)	Inferior by ≥20%	<0.001
(TKR) Scorpio cemented, unconstrained mobile	601	3.39	2.05	(0.61 to 3.49)	Inferior by ≥20%	0.003
(TKR) Scorpio hybrid, unconstrained fixed	565	1.95	0.60	(−0.54 to 1.75)	Non-inferiority not shown	0.152
(TKR) Scorpio uncemented, unconstrained fixed	1702	2.48	1.14	(0.36 to 1.91)	Inferior by ≥20%	0.002
(TKR) TC Plus cemented, unconstrained fixed	3820	2.19	0.84	(0.28 to 1.40)	Inferior by ≥20%	0.002
(TKR) TC Plus cemented, unconstrained mobile	2242	1.52	0.18	(−0.41 to 0.76)	Non-inferiority not shown	0.279
(TKR) TC Plus uncemented, unconstrained mobile	712	2.40	1.06	(0.02 to 2.09)	Non-inferiority not shown	0.023
(TKR) Triathlon cemented, posterior-stabilised fixed	2754	2.52	1.18	(0.63 to 1.72)	Inferior by ≥20%	<0.001
(TKR) Triathlon cemented, unconstrained fixed	8378	2.07	0.72	(0.32 to 1.12)	Inferior by ≥20%	<0.001
(TKR) Triathlon uncemented, unconstrained fixed	257	3.51	2.17	(0.50 to 3.84)	Inferior by ≥20%	0.005
(TKR) Vanguard cemented, posterior-stabilised fixed	665	2.87	1.53	(0.71 to 2.35)	Inferior by ≥20%	<0.001
(TKR) Vanguard cemented, unconstrained fixed	4840	1.96	0.61	(0.19 to 1.04)	Non-inferiority not shown	0.002
(UNI) AMC/Uniglide Unicondylar, fixed	409	4.80	3.45	(1.70 to 5.21)	Inferior by ≥100%	<0.001
(UNI) AMC/Uniglide Unicondylar, mobile	479	11.54	10.19	(7.69 to 12.69)	Inferior by ≥100%	<0.001
(UNI) MG Uni Unicondylar, fixed	953	6.66	5.32	(3.77 to 6.87)	Inferior by ≥100%	<0.001
(UNI) Oxford Partial Knee Unicondylar, mobile	13 555	6.58	5.23	(4.74 to 5.72)	Inferior by ≥100%	<0.001
(UNI) Physica ZUK Unicondylar, fixed	1113	4.02	2.67	(1.80 to 3.54)	Inferior by ≥100%	<0.001
(UNI) Preservation Unicondylar, fixed	480	12.12	10.78	(8.04 to 13.52)	Inferior by ≥100%	<0.001
(UNI) Sigma HP Unicondylar, fixed	688	5.89	4.55	(3.35 to 5.75)	Inferior by ≥100%	<0.001
(UNI) Sled Unicondylar, fixed	325	8.48	7.13	(4.41 to 9.85)	Inferior by ≥100%	<0.001

TKR, total knee replacement; UNI, unicondylar knee replacement.

At 7 years, the results were again similar between men and women. There were 40 different constructs used in women and 36 in men. In women, there were 19 constructs that were inferior by at least 20% relative risk, two of which were inferior by at least 100%. These were both UKRs. In men, 18 of the 36 constructs with ≥500 procedures still at risk were deemed to be inferior to the benchmark by at least 20% relative risk. Three of these 18 were inferior by at least 100% relative risk, all of which were UKRs. Two constructs were demonstrated to be non-inferior to the benchmark.

At 10 years, in both men and women, there was only one brand of UKR that had at least 500 procedures still at risk. In both instances, this was inferior by at least 100% relative risk and was the only construct to be classified as such. There were no brands found to be non-inferior to the benchmark in either men and women. In women, there were 8 of 15 constructs that were inferior to the benchmark by at least 20% relative risk. In men, 8 out of 13 constructs that were inferior to the benchmark by at least 20% relative risk. Data for gender specific stratification can be viewed in online supplementary figures 4, 5, 6 and 7 for men at 3, 5, 7 and 10 years, respectively, and online supplementary figures 8, 9, 10 and 11 for women at 3, 5, 7 and 10 years, respectively.

### Non-inferiority: gender and age specific

Subdividing procedures by age and gender highlights the paucity of information available for male or female patients <55 years compared with those ≥55 years. There is little data at 7 years, and no suitable benchmark could be found for women at 10 years in the <55 age group. There is a similar mix of construct types in each of the age groups in both men and women with cemented unconstrained TKRs with a fixed bearing the most popular type. Data for men under 55 years of age at 3, 5 and 7 years can be viewed in online supplementary figures 12, 13 and 14, respectively. Data for women under 55 years of age at 3, 5 and 7 years can be viewed in online supplementary figures 15, 16 and 17, respectively.

**Figure 4:**
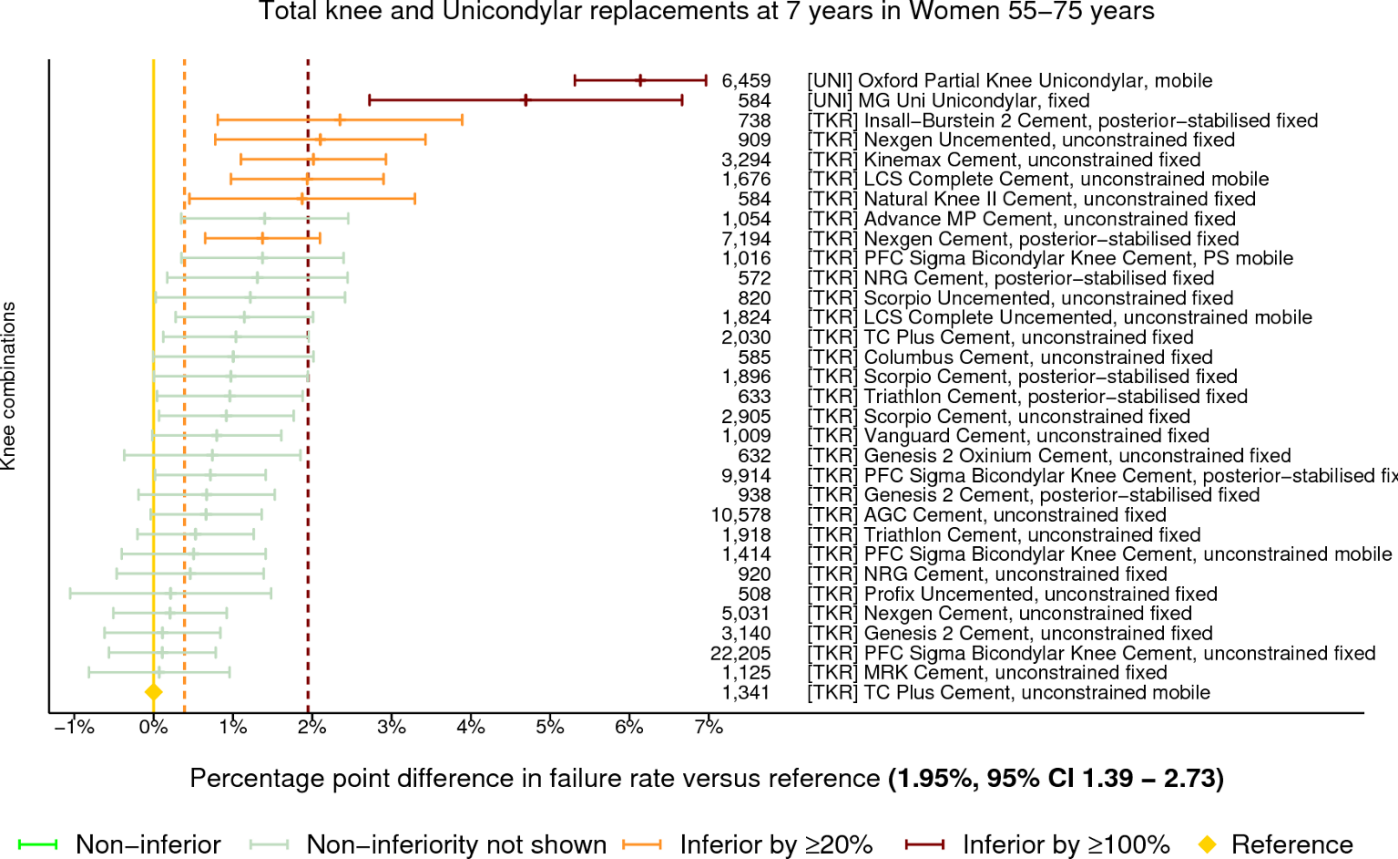
Difference in cumulative revision of knee constructs compared with a contemporary benchmark at 7 years in women aged between 55 years and 75 years, using all total knee and unicondylar replacements with ≥500 procedures remaining at risk.

**Figure 5:**
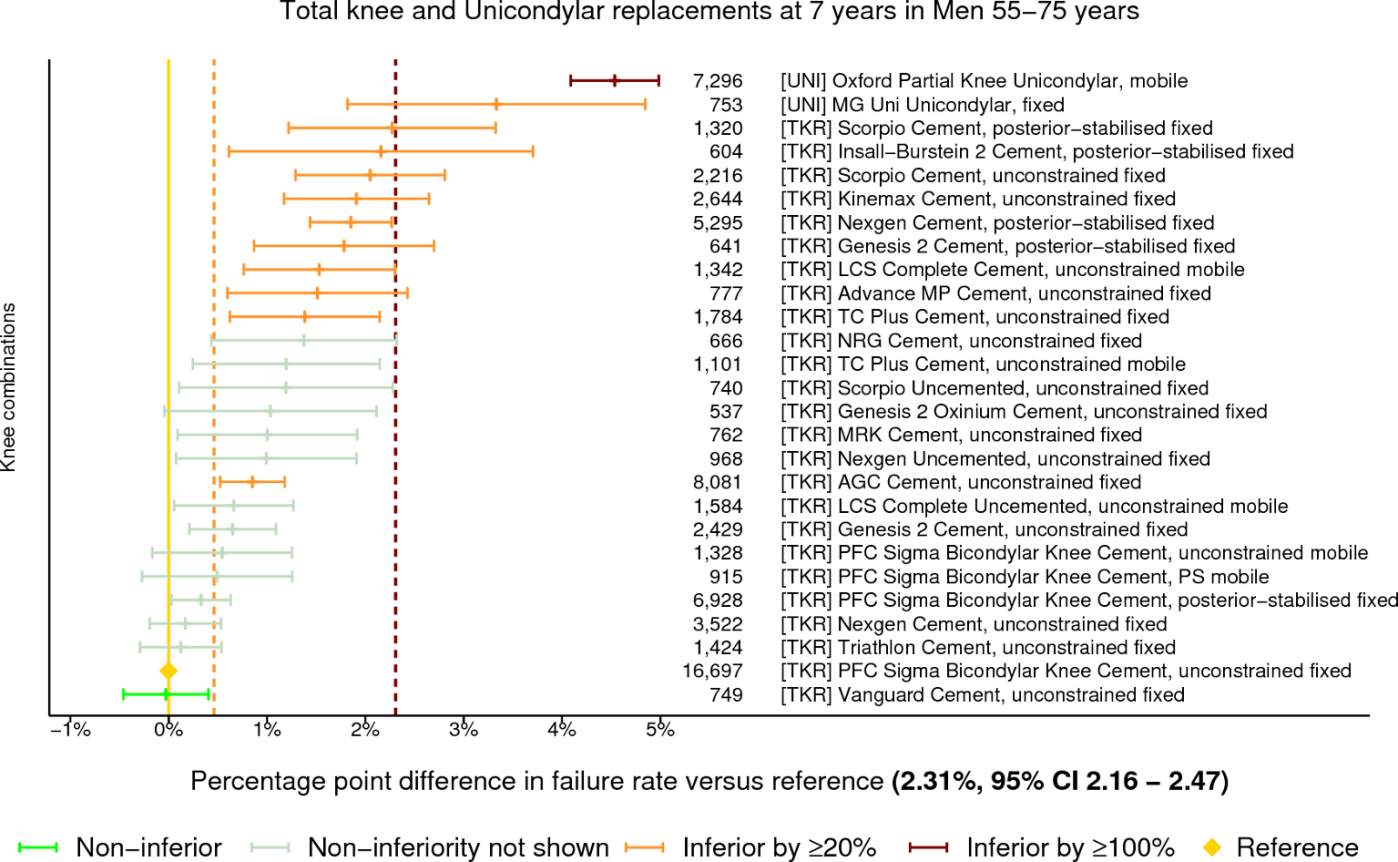
Difference in cumulative revision of knee constructs compared with a contemporary benchmark at 7 years in men aged between 55 years and 75 years, using all total knee and unicondylar replacements with ≥500 procedures remaining at risk.

At 7 years in women aged 55–75 years, there were 32 different constructs that met the threshold of 500 cases for analysis with 8 being classified as inferior by at least 20% relative risk (figure 4). Two of these were inferior by at least 100% relative risk and both were UKRs. No constructs were demonstrated to be non-inferior to the benchmark. In men, there were 27 constructs meeting the threshold with 12 being classified as inferior by at least 20% relative risk (figure 5). A single prosthesis, 1 of the 2 UKRs with at least 500 procedures still at risk, was classified as being inferior by at least 100% relative risk. One construct was found to be non-inferior to the benchmark. Data for men between 55 years and 75 years of age at 3, 5 and 10 years postprimary can be viewed in online supplementary figures 18, 19 and 20, respectively. Data for women between 55 years and 75 years of age at 3, 5 and 10 years postprimary can be viewed in online supplementary figures 21,22 and 23, respectively.

In the >75 years age group, we found only one inferior construct in both men and women at 7 years. Non-inferiority was not shown for any other prostheses, but very few met the threshold of 500 cases needed for analysis. At 10 years, only three constructs with ≥500 procedures still at risk were present in men and five in women. Non-inferiority was not demonstrated in any of these. At this time point and age group subdivision, there were no UKRs with enough procedures still at risk to be included in the analysis. Data for men >75 years of age at 3, 5, 7 and 10 years postprimary can be viewed in online supplementary figures 24, 25, 26 and 27, respectively. Data for women >75 years of age at 3, 5, 7 and 10 years postprimary can be viewed in online supplementary figures 28, 29, 30 and 31, respectively.

Data for each level of stratification for comparator constructs with at least 250 patients at risk at the beginning of the time point of interest can be viewed in the following supplemental tables. Data for all men at 3, 7 and 10 years can be seen in online supplemental tables 5, 6 and 7, respectively, and for all women at 3, 7 and 10 years in online supplemental tables 8, 9 and 10. Data for men <55 years of age at 3, 5 and 7 years can be seen in online supplemental tables 11, 12 and 13, respectively, and women <55 at 3, 5 and 7 years can be seen in online supplemental tables 14, 15 and 16, respectively. Data for men aged between 55 years and 75 years at 3, 5, 7 and 10 years can be seen in online supplemental tables 17, 18, 19 and 20, respectively, and for women aged between 55 years and 75 years at 3, 5, 7 and 10 years data can be seen in online supplemental tables 21, 22, 23 and 24, respectively. Data for men aged >75 years at 3, 5, 7 and 10 years postprimary can be seen in online supplemental tables 25, 26, 27 and 28, respectively, and for women >75 years at 3, 5, 7 and 10 years postprimary data can be seen in online supplemental tables 29, 30, 31 and 32, respectively.

### Sensitivity analysis

Using the benchmark construct at 10 years as the benchmark at 3, 5 and 7 years illustrates the temporal improvements in failure and the selective trajectory tracking of new constructs. The benchmark construct in all procedures at 10 years is the PFC Sigma Bicondylar Knee cemented unconstrained TKR with a fixed bearing that had a failure rate of 2.37% (95% CI 2.27 to 2.47). At 3, 5 and 7 years, the contemporary benchmark has a 0.15%, a 0.17% and 0.23% lower point-estimate failure rate than the historical benchmark, respectively. The performance of the constructs appears to track in a consistent manner from each time point to the next.

## Discussion

We have demonstrated, in 947 686 primary TKRs and UKRs, the relative performance of knee constructs in comparison with an internally selected relevant contemporary benchmark. There are two striking findings from the data analysed here. First, UKR brands almost universally have 100% worse revision outcomes in all ages, gender and time points of interest compared with the benchmark standard brand of knee replacement. Second, very few brands of knee replacement can be proven not to be at least 20% worse than the benchmark brands despite 449 brand constructs having been implanted in England and Wales since 2003. Between one-third and two-thirds of the knee constructs for which sufficient data were available at each time point were at least 20% inferior to the contemporary benchmark. The vast majority of constructs are implanted in too few cases to allow meaningful analysis. Many of those implanted in sufficient number have here been demonstrated to be inferior in terms of construct survivorship, while very few TKRs have been demonstrated to be non-inferior to a contemporary benchmark.

It is known that TKRs as a class have lower revision rates than UKRs, which poses the question, should these two classes be directly compared? Since every patient who received a UKR could have received a TKR instead, this comparison is justified. There is no evidence to suggest that the subsequent observed revision rates would be any different if those receiving a UKR had received a TKR instead. Furthermore, ODEP (currently) do not provide a rating for UKRs so this method provides extra transparency and previously unavailable information for patients undergoing knee replacement.

### Comparison with other studies and implications in light of existing evidence

No other published study has performed a direct head-to-head comparison of all available knee replacement constructs. The closest available data are from national registry annual reports such as the NJR,^1^ which reports absolute failure of the most common constructs by age and gender. This shows that low failure rates are achieved by a number of constructs, but this does not facilitate easy direct comparison between them. Using the data presented here alongside the annual report data will greatly enhance the information available to surgeons, commissioners and to inform patient choice.

Consideration of the difference in outcome of UKR compared with TKR is complex. Using propensity score matching of registry data, Liddle *et al* 13 showed that UKRs have higher revision rates than TKRs but lower risks of complications. Hunt *et al* and Liddle *et al* showed UKRs to be associated with lower early postoperative mortality.^13 14^ Kleeblad *et al* 15 performed a systematic review and meta-analysis of 49 cohort studies and found no difference in function as measured by Hospital for Special Surgery Score, Knee Society Score, Oxford Knee Score, Visual Analog Pain Scale and Western Ontario and McMaster Universities Osteoarthritis Index Score but did show higher revision rates with UKRs. Liddle *et al* 16 also showed no clinically important difference in Oxford Knee Scores (one point difference favouring UKR), but UKR patients were more likely to be highly satisfied 6 months after surgery. A recent study by the same group has shown that UKR is less costly than TKR particularly in older patients, who are less likely to require revision, and when performed by higher volume surgeons.^17^ Our data add to this complex picture by clearly demonstrating the increased risk of revision associated with almost all brands of UKRs at all time points in both genders and all age groups when compared with the best performing TKRs.

The major weakness of all registry studies is selection bias whereby certain constructs may be implanted for particular indications and in particular groups with different risks. We have mitigated against this by analysing data by age and gender, the two variables with the greatest association with revision rates. Furthermore, revision thresholds may be lower in certain groups or for certain modes of failure. This study has looked at a single, but extremely important, outcome measure: revision. Other outcomes of interest such as mortality and patient-report outcome measures need to be considered when making choices about treatment. With over 900 000 subjects, this is the largest study of knee replacement published to date and comes from the largest implant registry in the world. Data entry is mandated, and data capture is extremely high (over 95%),^18^ thus the findings are highly likely to be generalisable. The methods used are novel and, for the first time, allow a meaningfully direct comparison between all available constructs.

### Conclusions, policy and future research implications

The use of product benchmarking has the potential to be highly informative for patients, change the practice of surgeons and influence policy makers if presented clearly and unambiguously. The implications of this research are far reaching. We are unable to definitively state which construct is the best choice for all patients, due to the presence of selection effects and residual confounding. However, we believe that the information presented here illustrates the variability, frequency and performance of different constructs currently used in clinical practice which, in turn, should be used to further inform the consenting process between the patient and the surgeon and facilitate implant selection. We believe commissioners and policy makers should consider the variability and performance of different implants in the commissioning of healthcare providers.

Patients should be actively involved in decision making about their treatment. Here we provide, for the first time, data that allow patients and clinicians to directly compare revision rates associated with the use of different UKR and TKR constructs. The information provided should be used to inform patient choice, surgical decision making and commissioning.

## Supplementary Material

Reviewer comments

Author's manuscript
