# Supplementary material for: Assessing the non-inferiority of prosthesis constructs used in total and unicondylar knee replacements using data from the National Joint Registry of England, Wales, Northern Ireland and the Isle of Man: a benchmarking study
Source: BMJ Open. 2019 Apr 29;9(4):e026736. doi: 10.1136/bmjopen-2018-026736 (PMC6502008; doi:10.1136/bmjopen-2018-026736)
Supplement: Supplementary file 1 [file bmjopen-2018-026736supp001.pdf]

Supplementary Figure 1: Flow diagram of showing derivation of procedures used in analyses

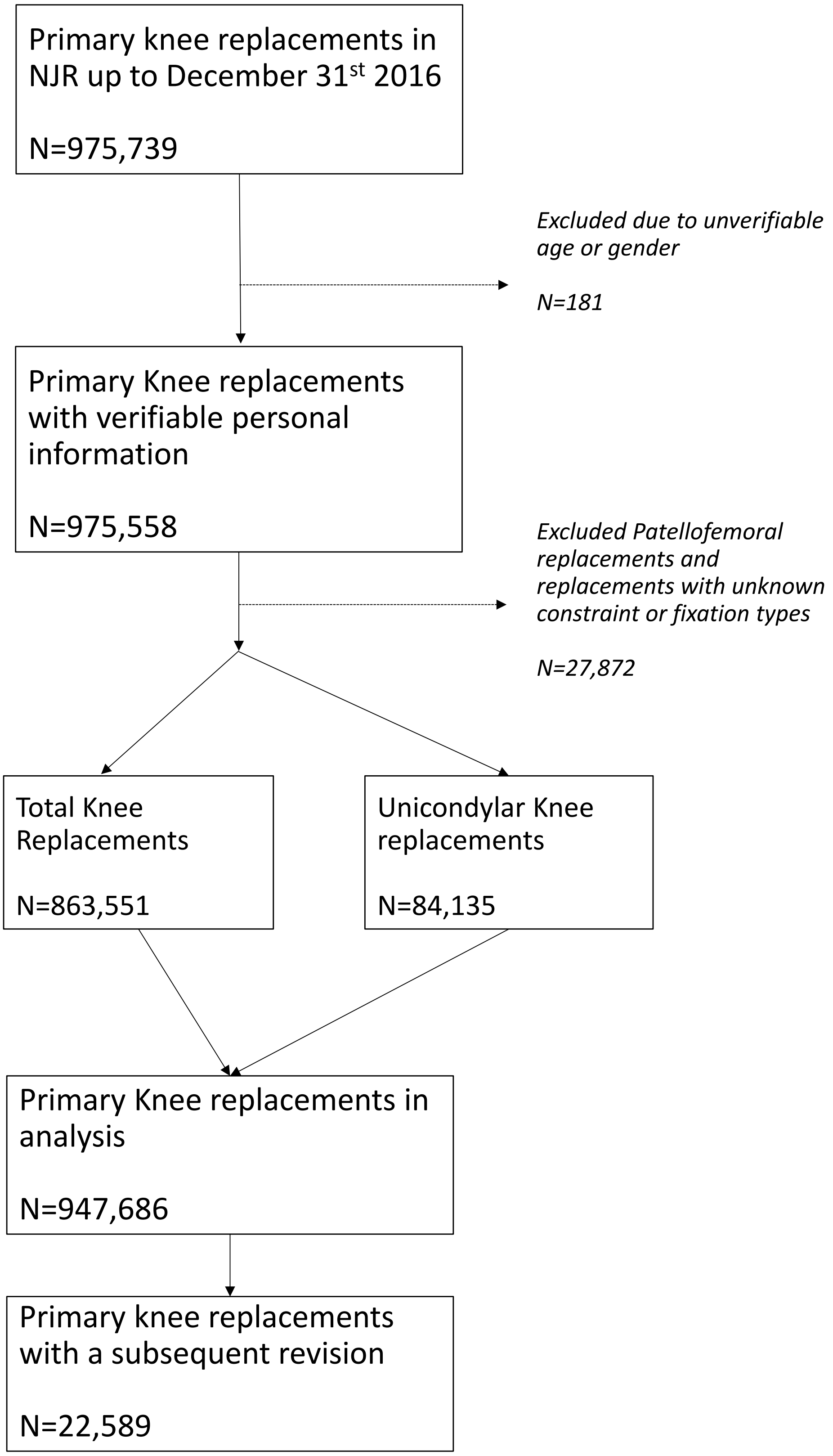

Supplementary Figure 2: Difference in cumulative revision of knee implants compared to a contemporary reference at 3 years, using all total knee and unicondylar replacements with ≥500 procedures remaining at risk.

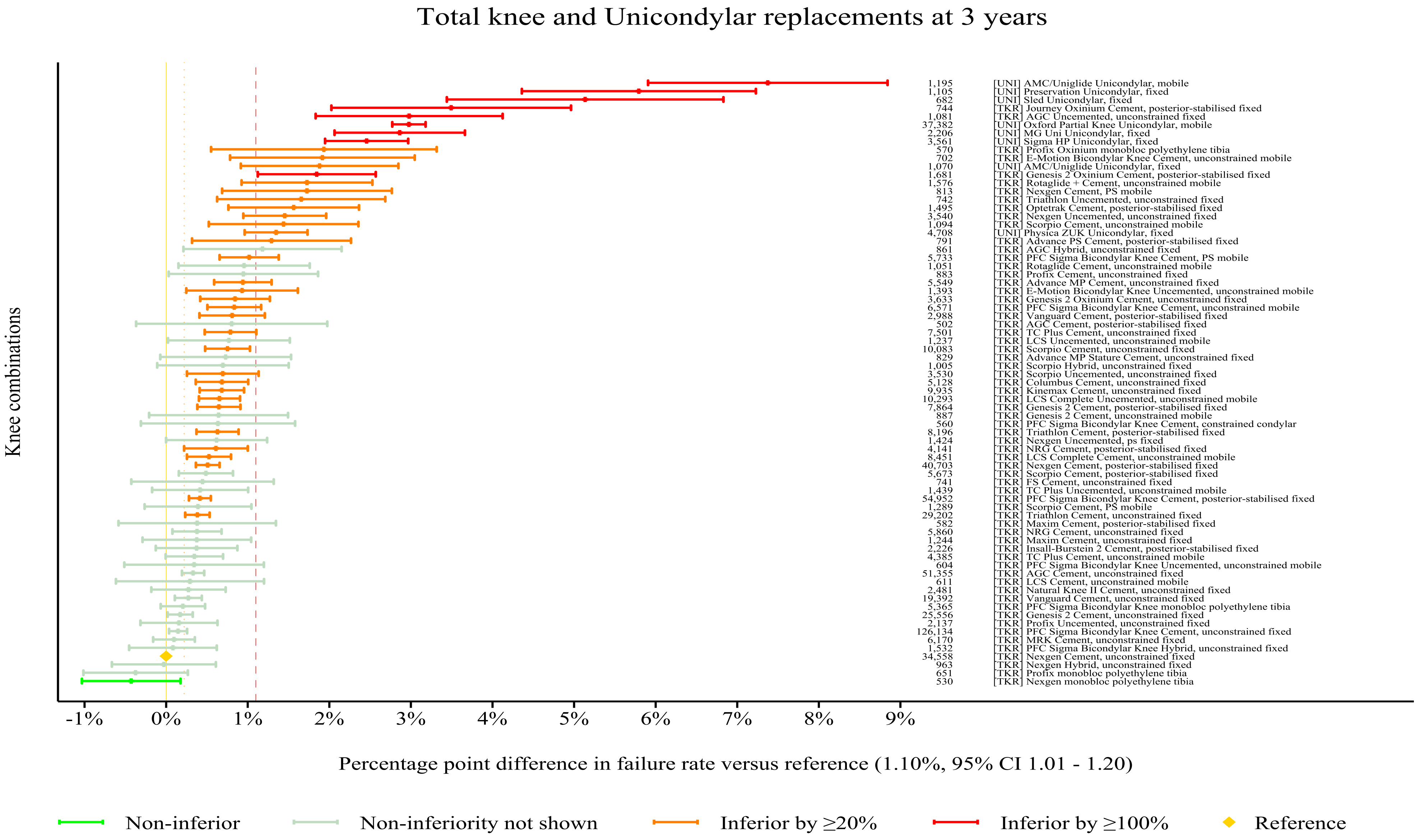

Supplementary Figure 3: Difference in cumulative revision of knee implants compared to a contemporary reference at 7 years, using all total knee and unicondylar replacements with ≥500 procedures remaining at risk.

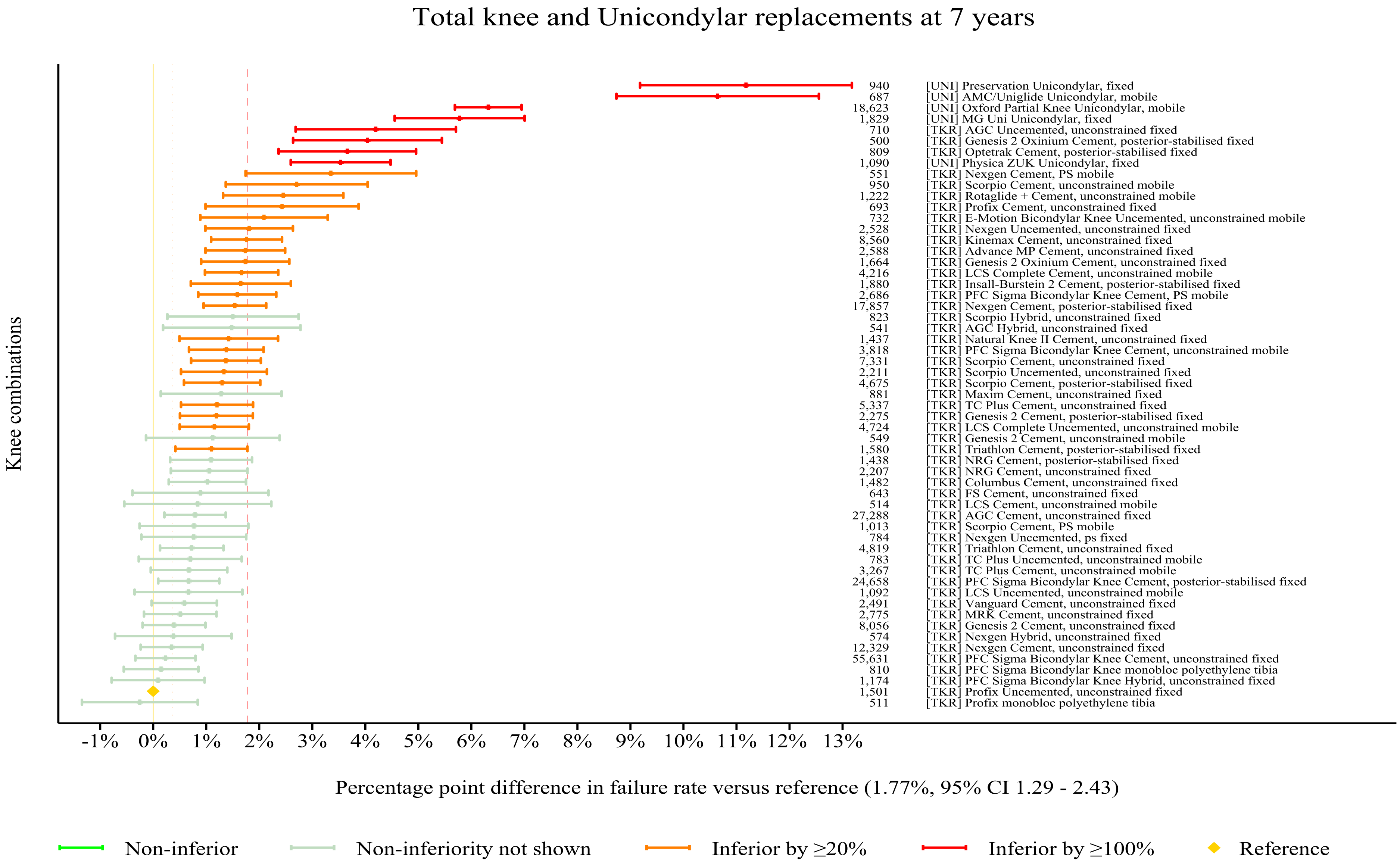

Supplementary Figure 4: Difference in cumulative revision of knee implants compared to a contemporary reference at 3 years in men, using all total knee and unicondylar replacements with ≥500 procedures remaining at risk.

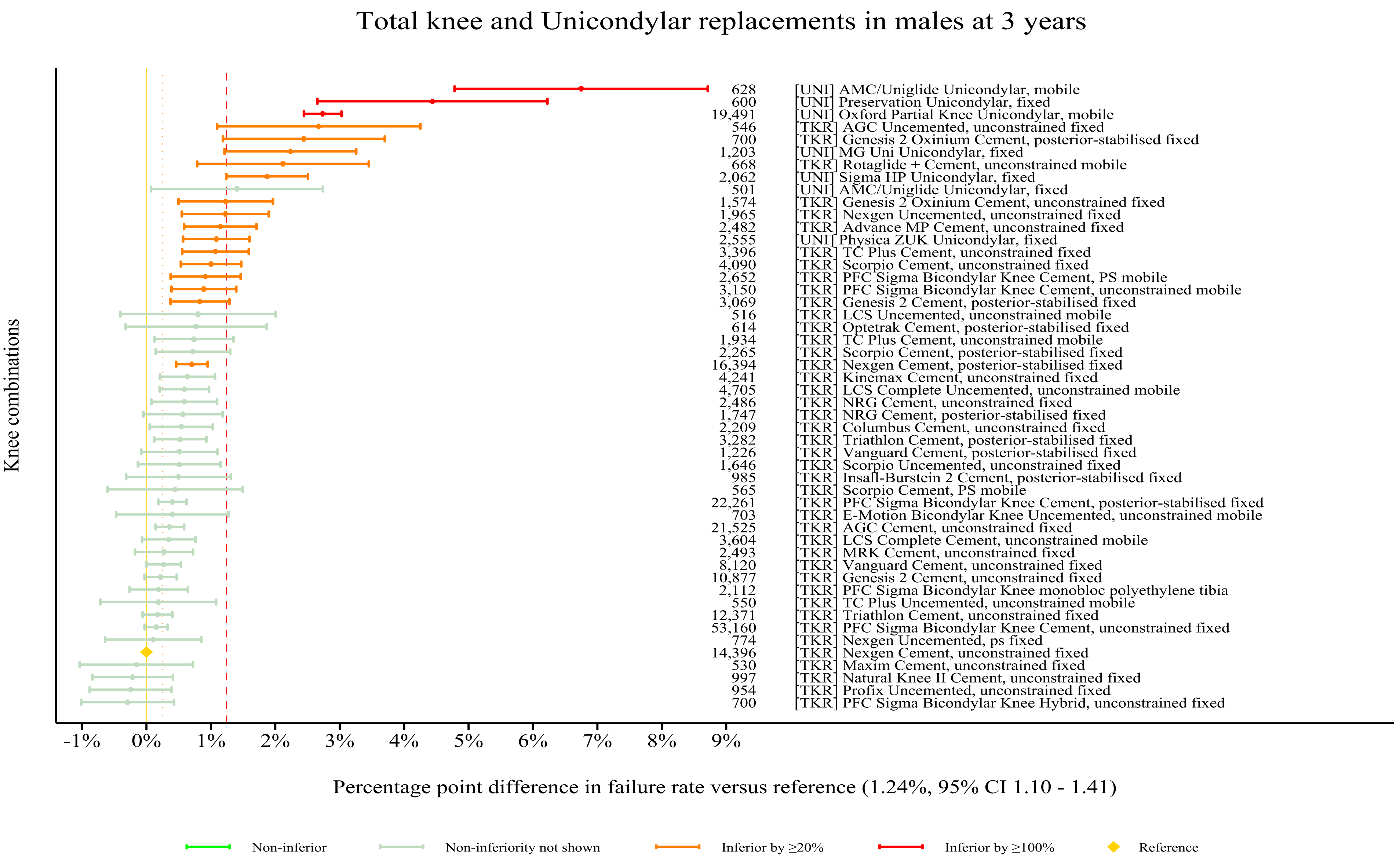

Supplementary Figure 5: Difference in cumulative revision of knee implants compared to a contemporary reference at 5 years in men, using all total knee and unicondylar replacements with ≥500 procedures remaining at risk.

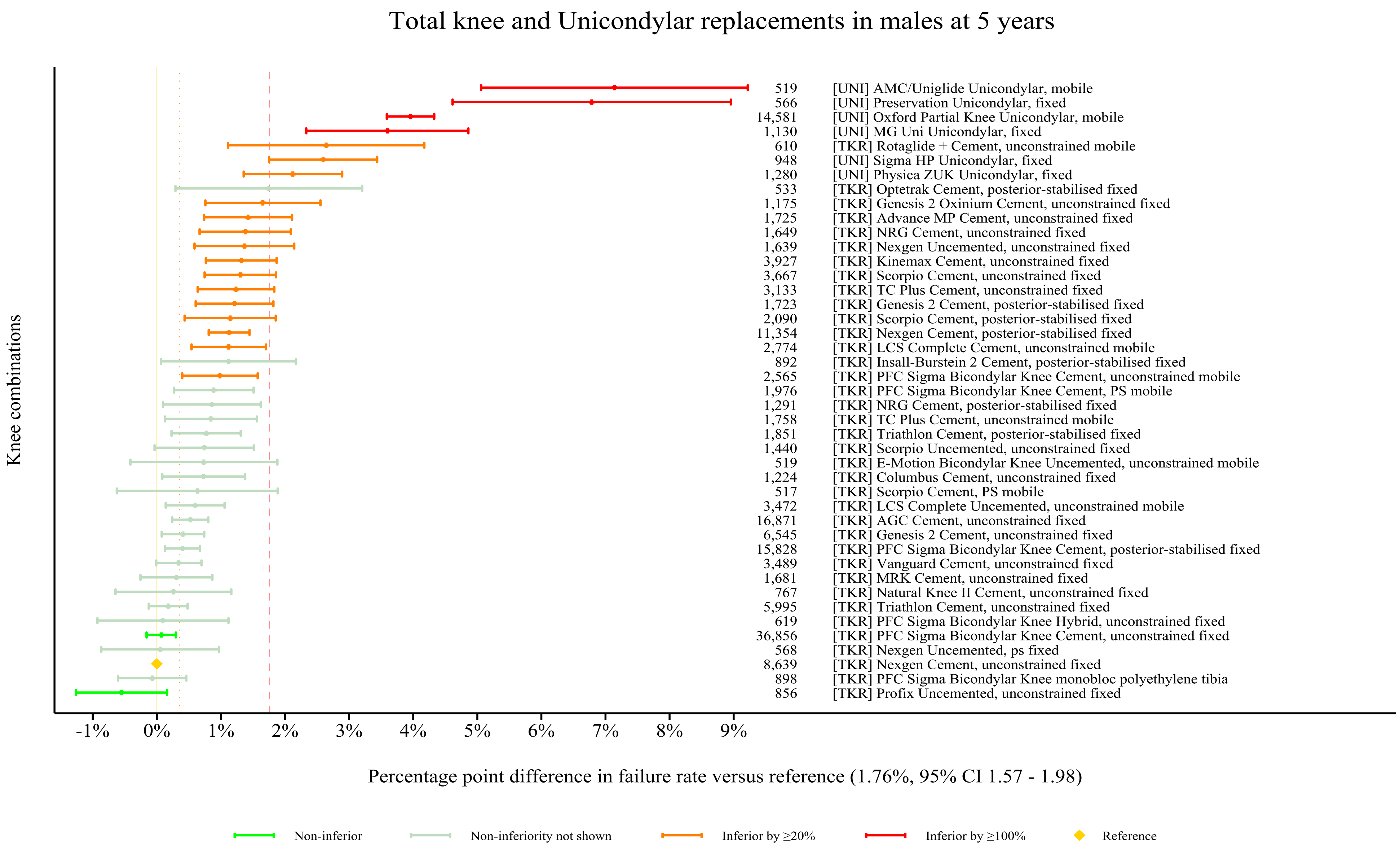

Supplementary Figure 6: Difference in cumulative revision of knee implants compared to a contemporary reference at 7 years in men, using all total knee and unicondylar replacements with ≥500 procedures remaining at risk.

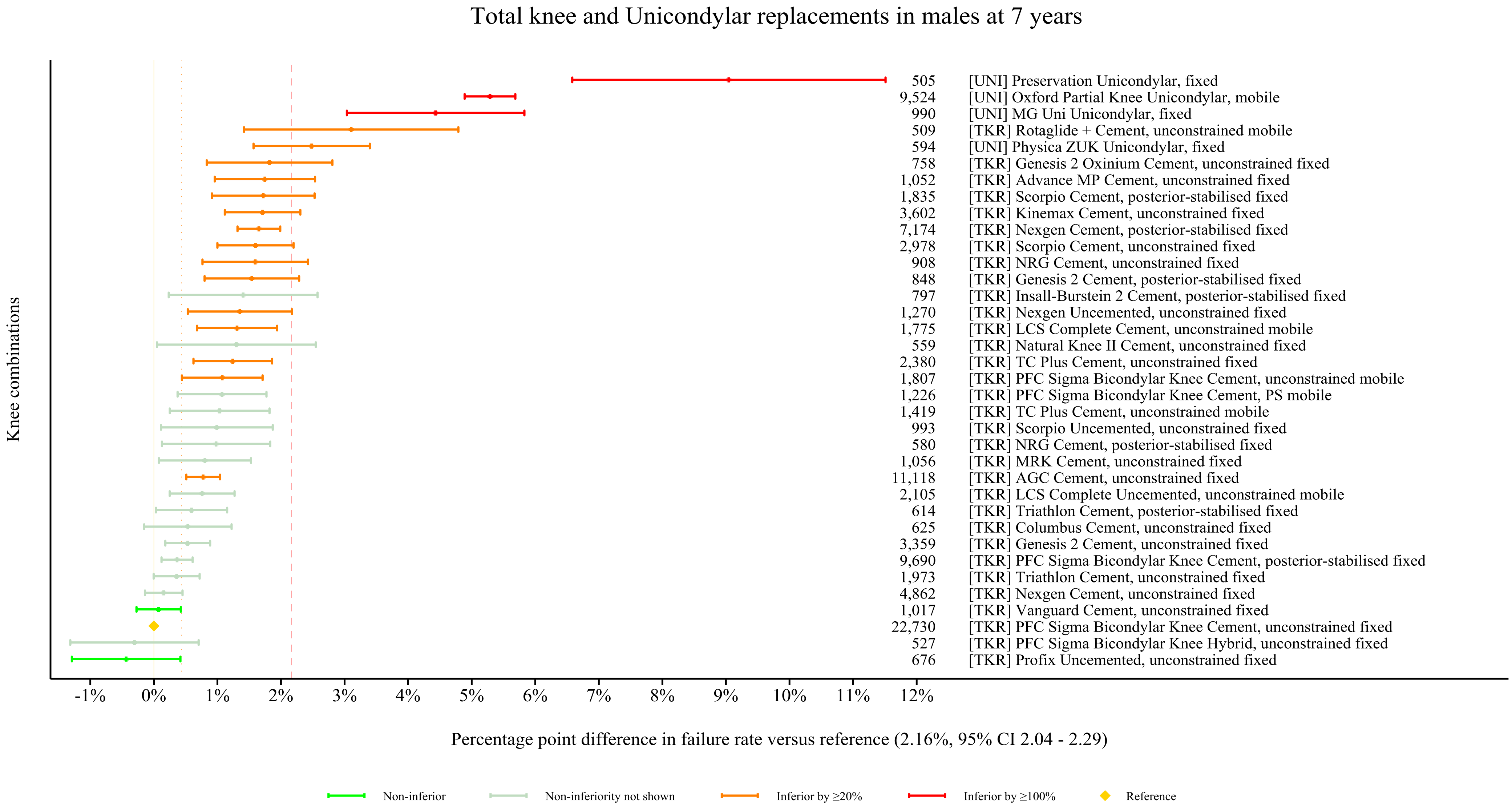

Supplementary Figure 7: Difference in cumulative revision of knee implants compared to a contemporary reference at 10 years in men, using all total knee and unicondylar replacements with ≥500 procedures remaining at risk.

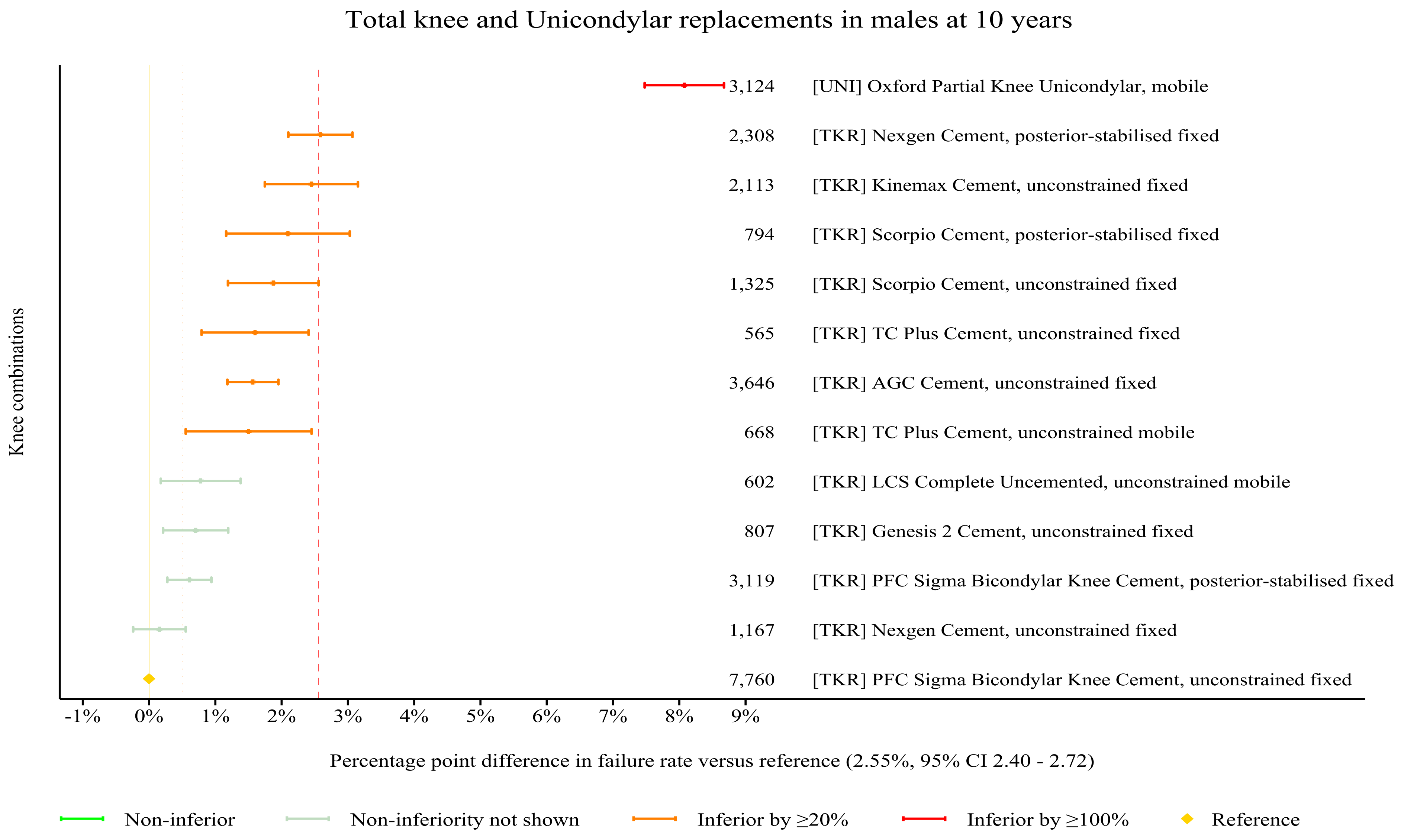

Supplementary Figure 8: Difference in cumulative revision of knee implants compared to a contemporary reference at 3 years in women, using all total knee and unicondylar replacements with ≥500 procedures remaining at risk.

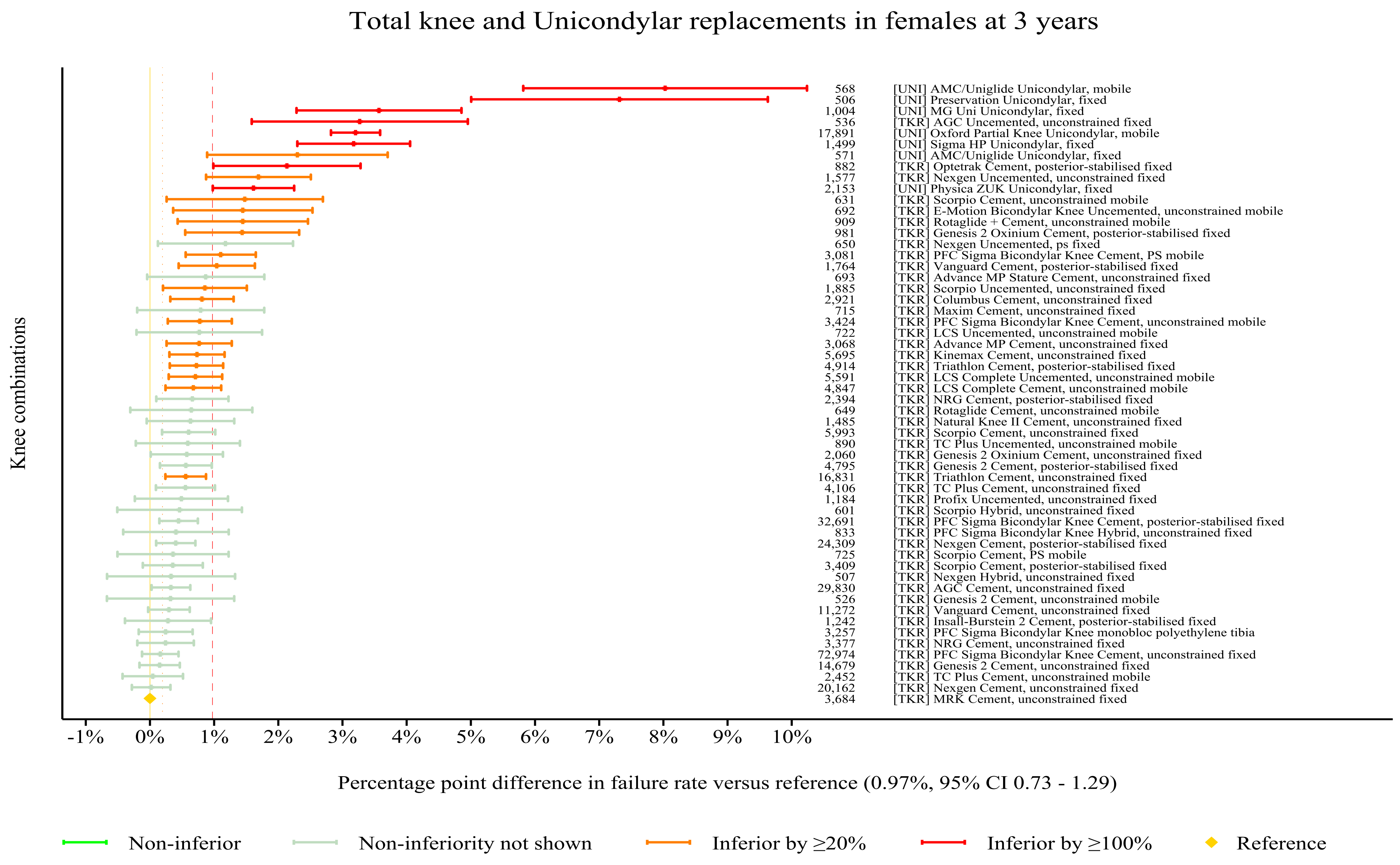

Supplementary Figure 9: Difference in cumulative revision of knee implants compared to a contemporary reference at 5 years in women, using all total knee and unicondylar replacements with ≥500 procedures remaining at risk.

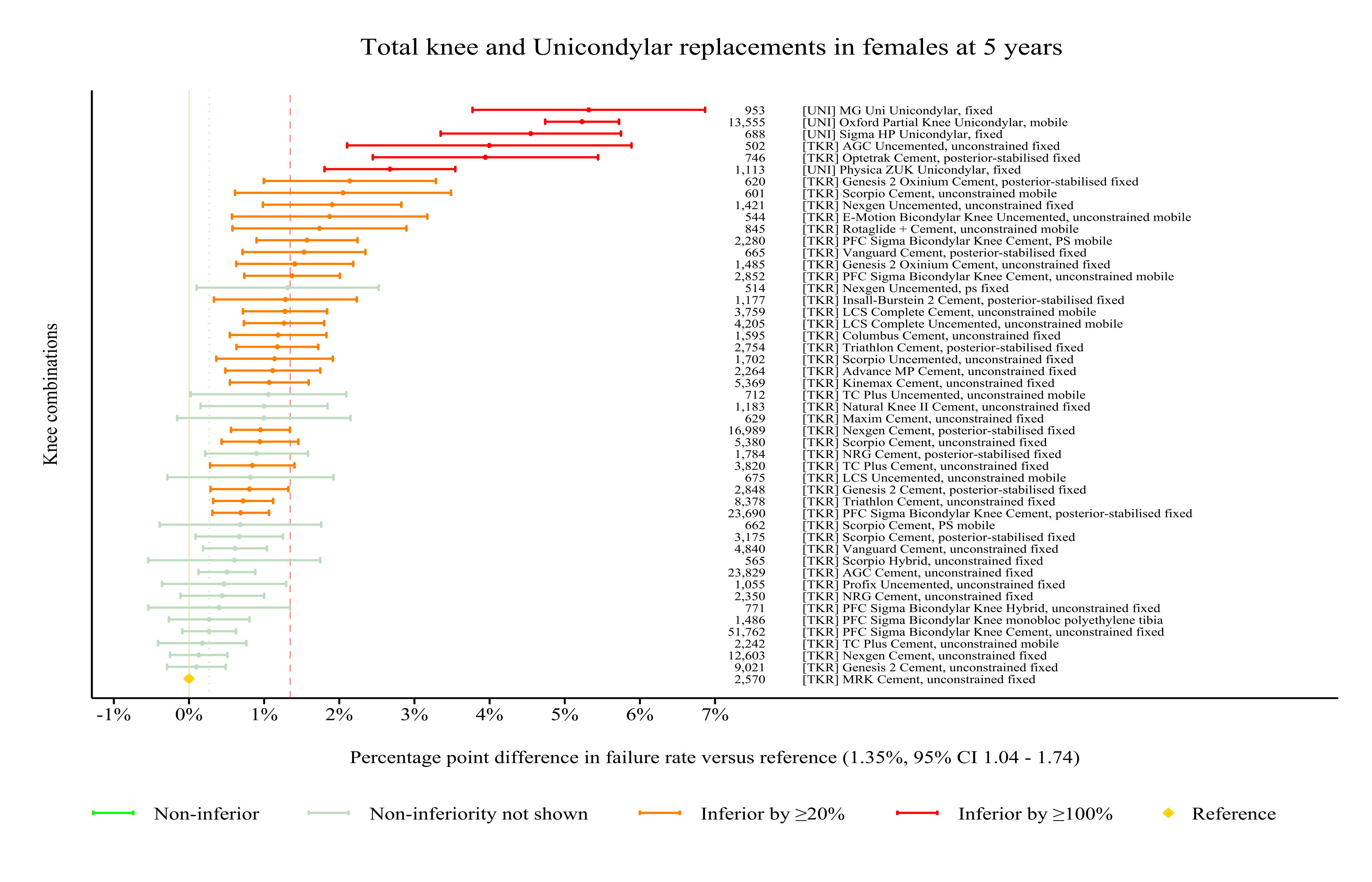

Supplementary Figure 10: Difference in cumulative revision of knee implants compared to a contemporary reference at 7 years in women, using all total knee and unicondylar replacements with ≥500 procedures remaining at risk.

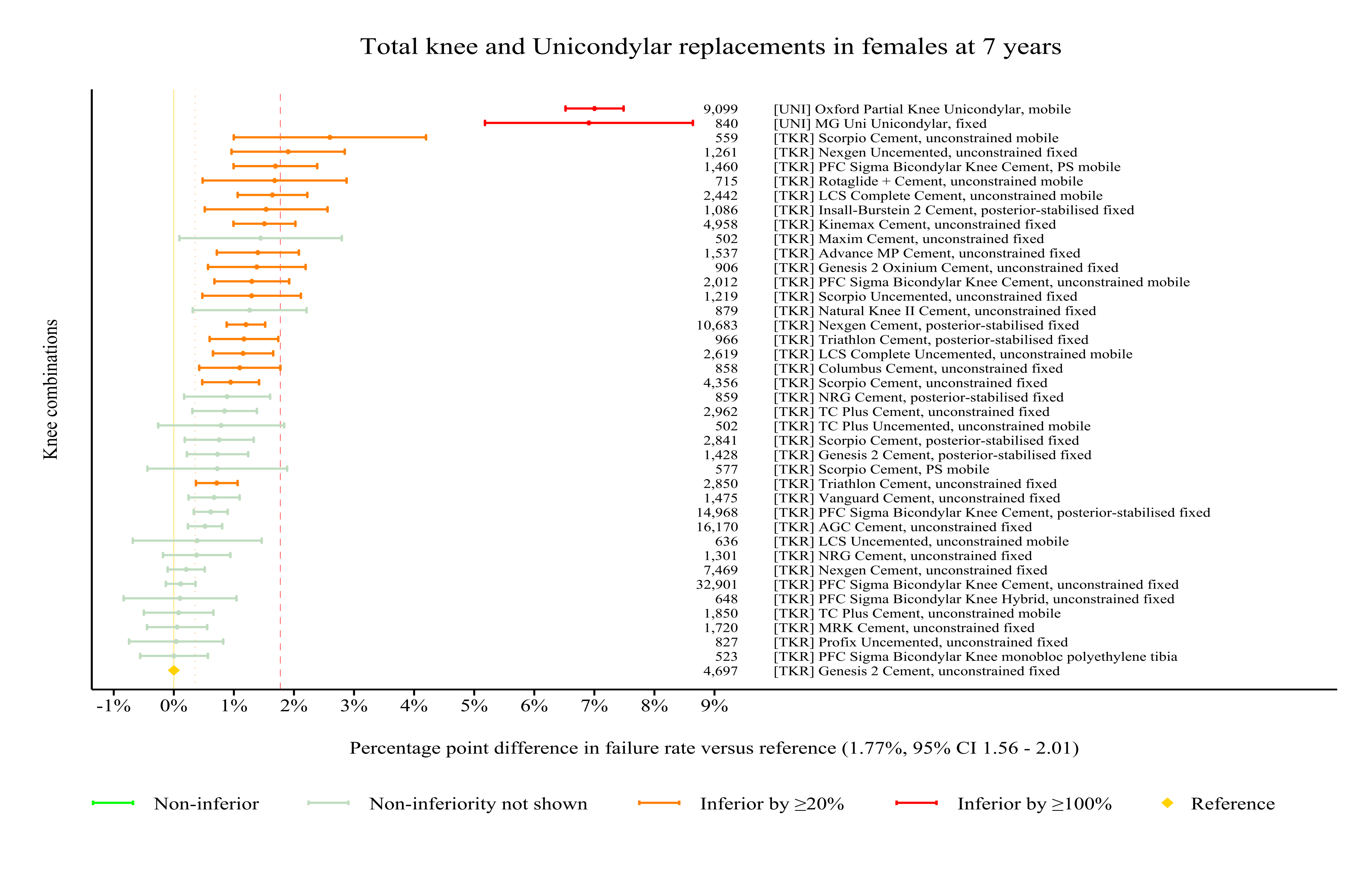

Supplementary Figure 11: Difference in cumulative revision of knee implants compared to a contemporary reference at 10 years in women, using all total knee and unicondylar replacements with ≥500 procedures remaining at risk.

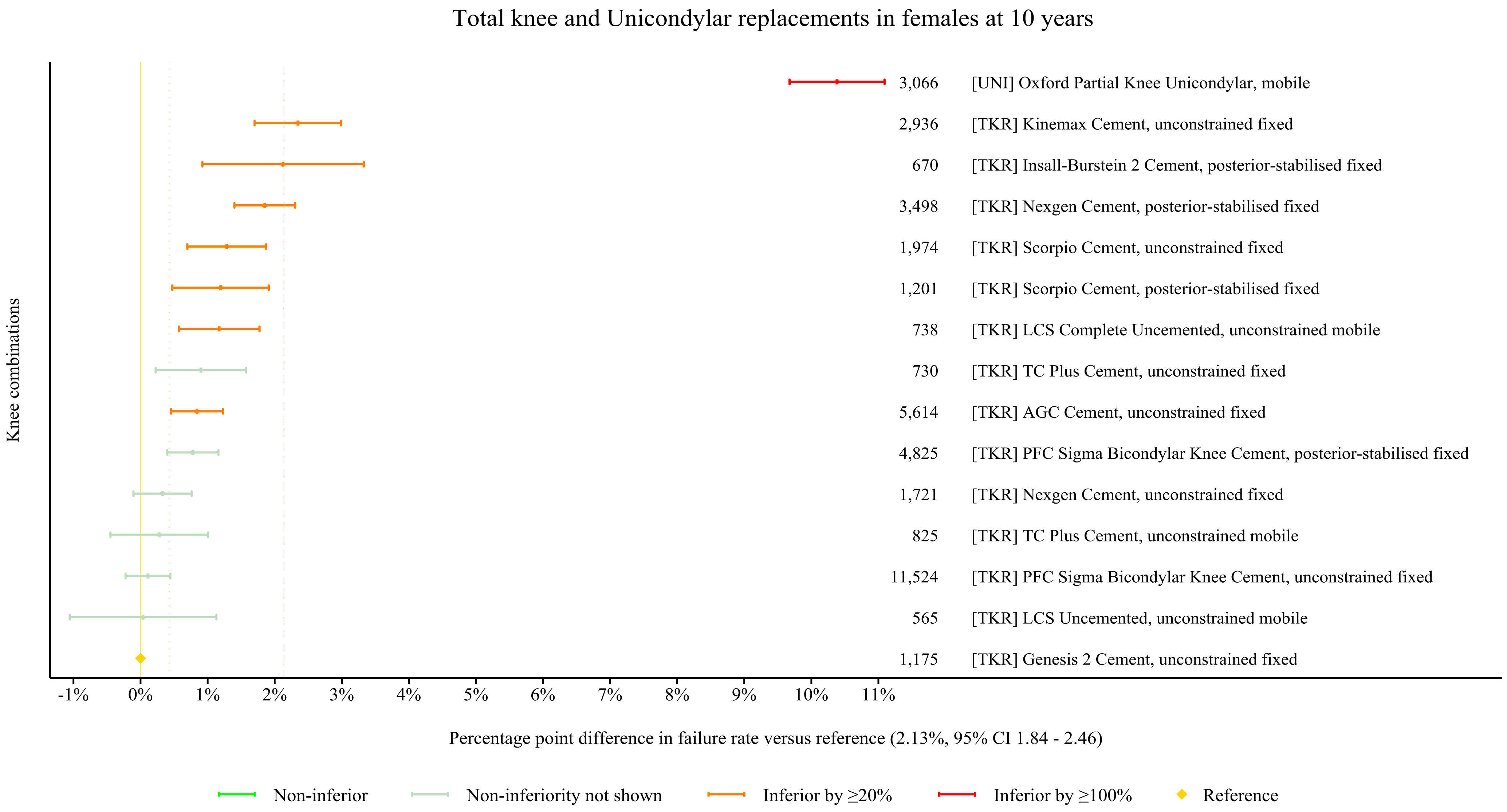

Supplementary Figure 12: Difference in cumulative revision of knee implants compared to a contemporary reference at 3 years in men less than 55 years, using all total knee and unicondylar replacements with ≥500 procedures remaining at risk.

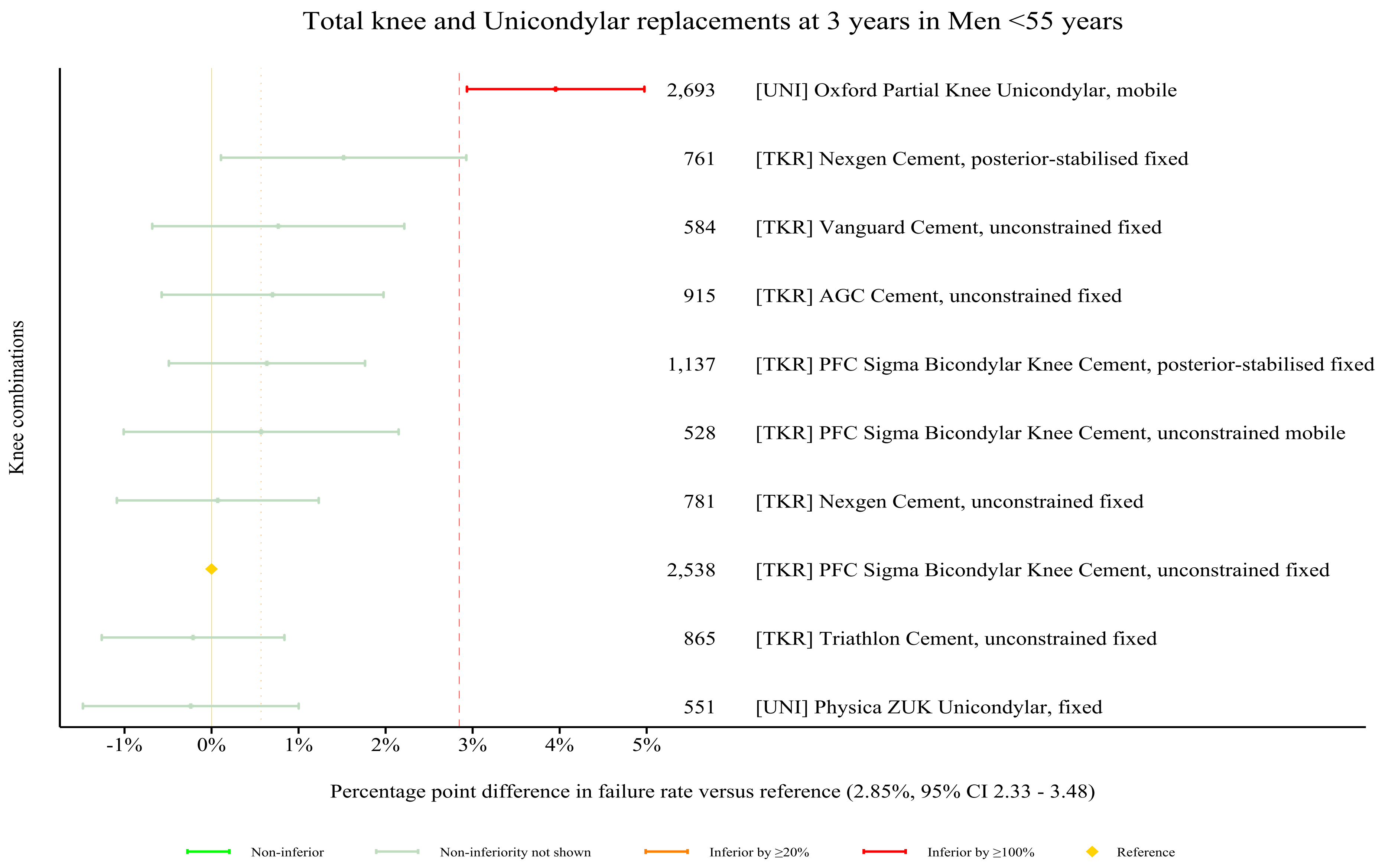

Supplementary Figure 13: Difference in cumulative revision of knee implants compared to a contemporary reference at 5 years in men less than 55 years, using all total knee and unicondylar replacements with ≥500 procedures remaining at risk.

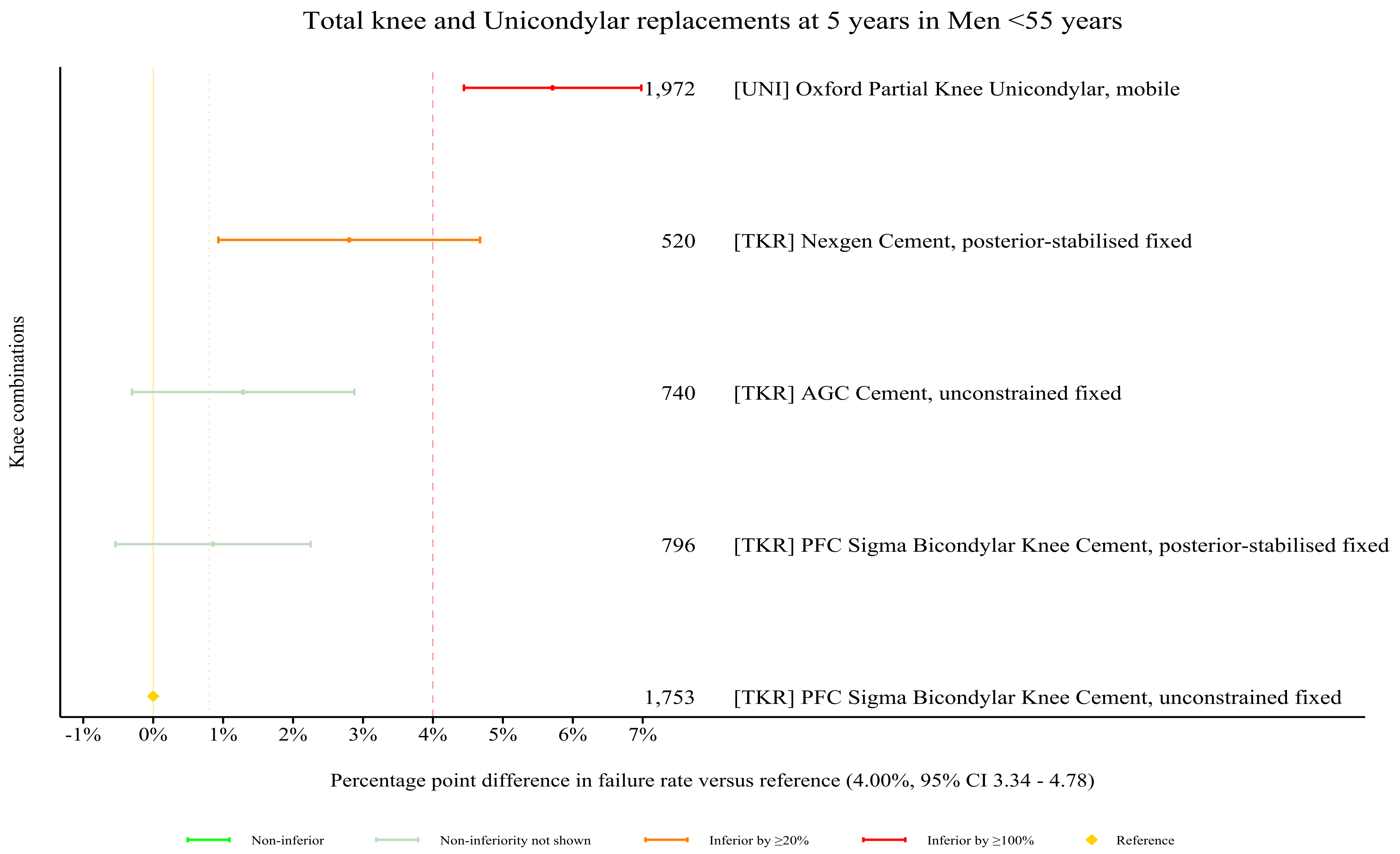

Supplementary Figure 14: Difference in cumulative revision of knee implants compared to a contemporary reference at 7 years in men less than 55 years, using all total knee and unicondylar replacements with ≥500 procedures remaining at risk.

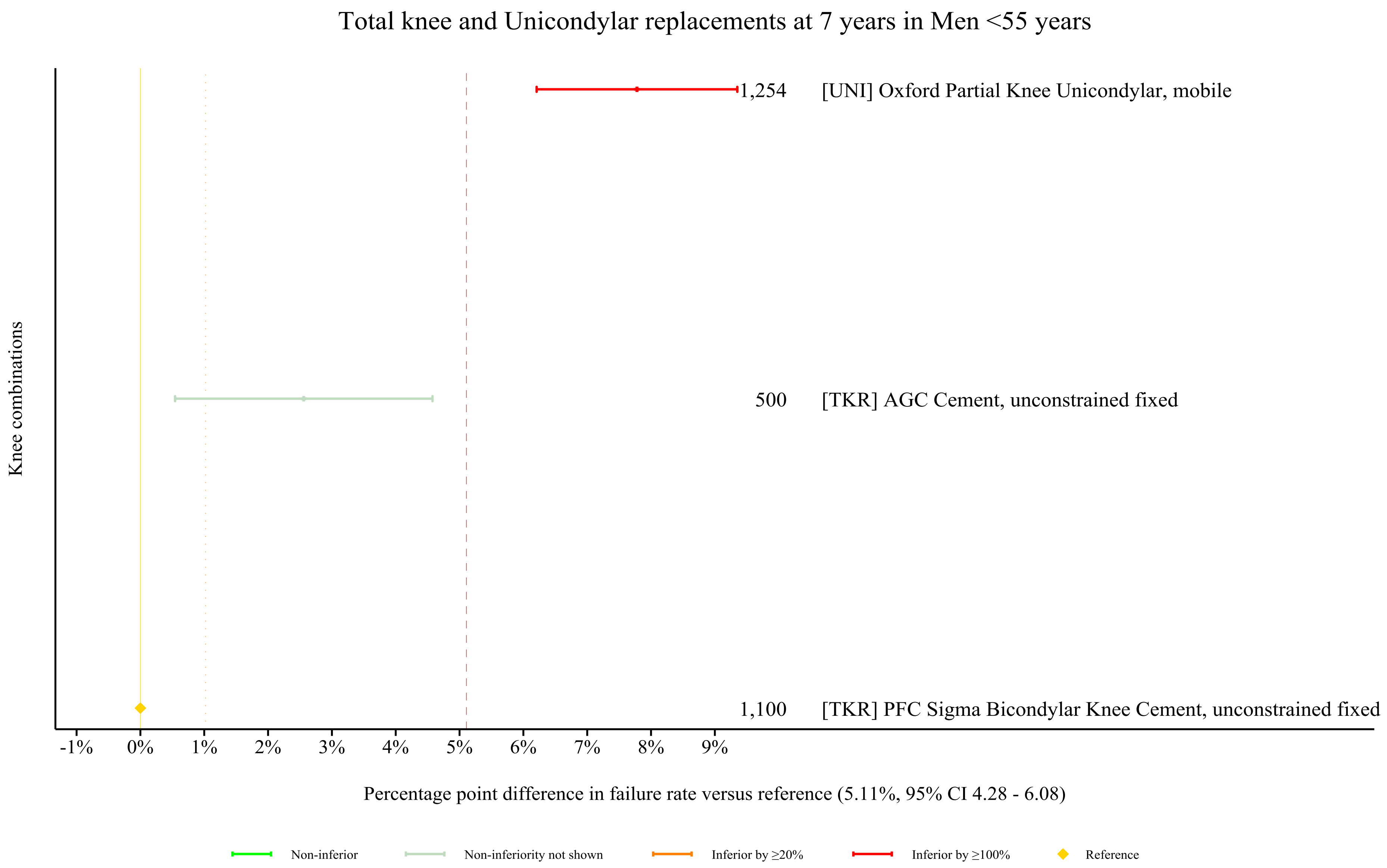

Supplementary Figure 15: Difference in cumulative revision of knee implants compared to a contemporary reference at 3 years in women less than 55 years, using all total knee and unicondylar replacements with ≥500 procedures remaining at risk.

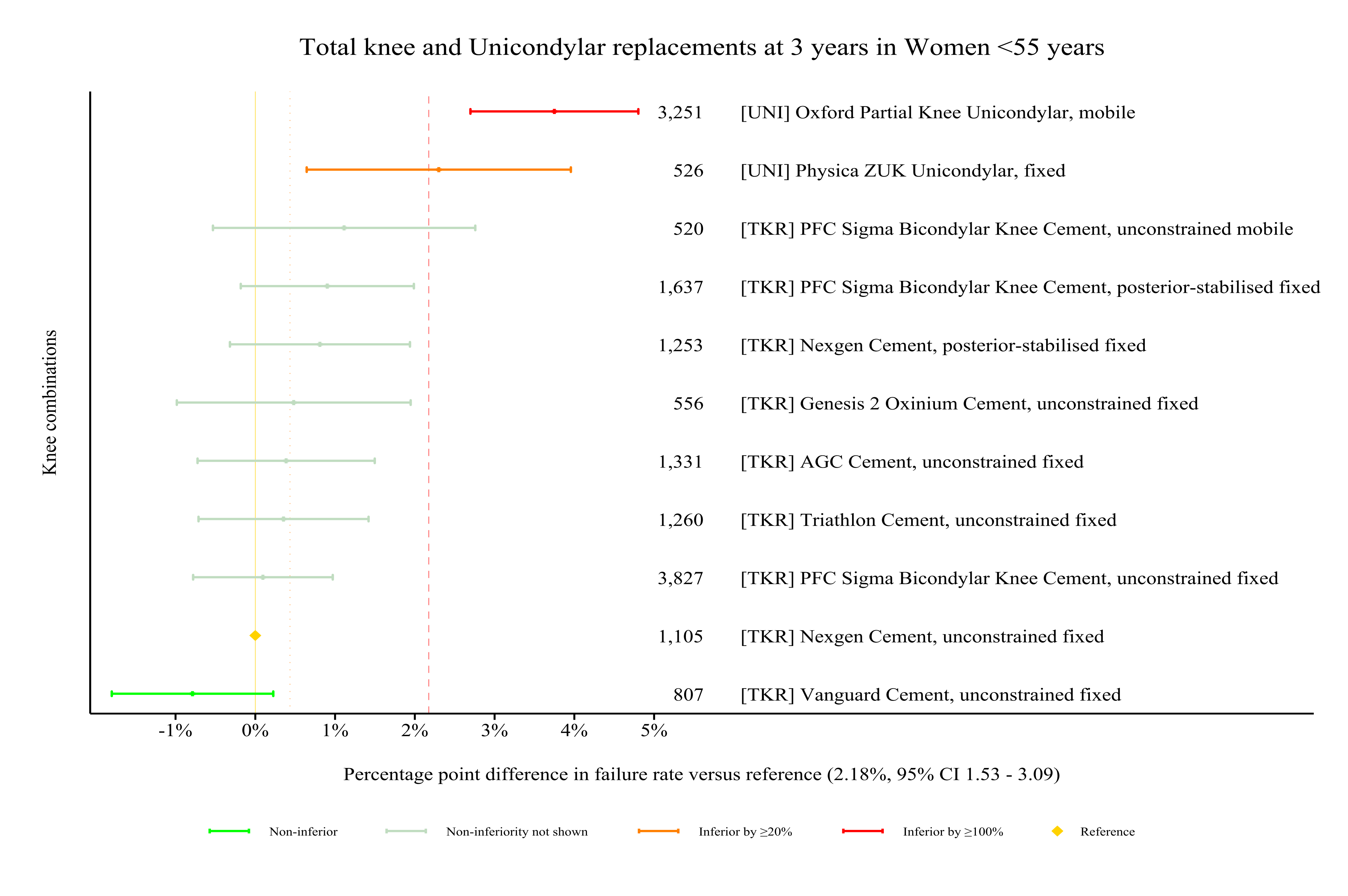

Supplementary Figure 16: Difference in cumulative revision of knee implants compared to a contemporary reference at 5 years in women less than 55 years, using all total knee and unicondylar replacements with ≥500 procedures remaining at risk.

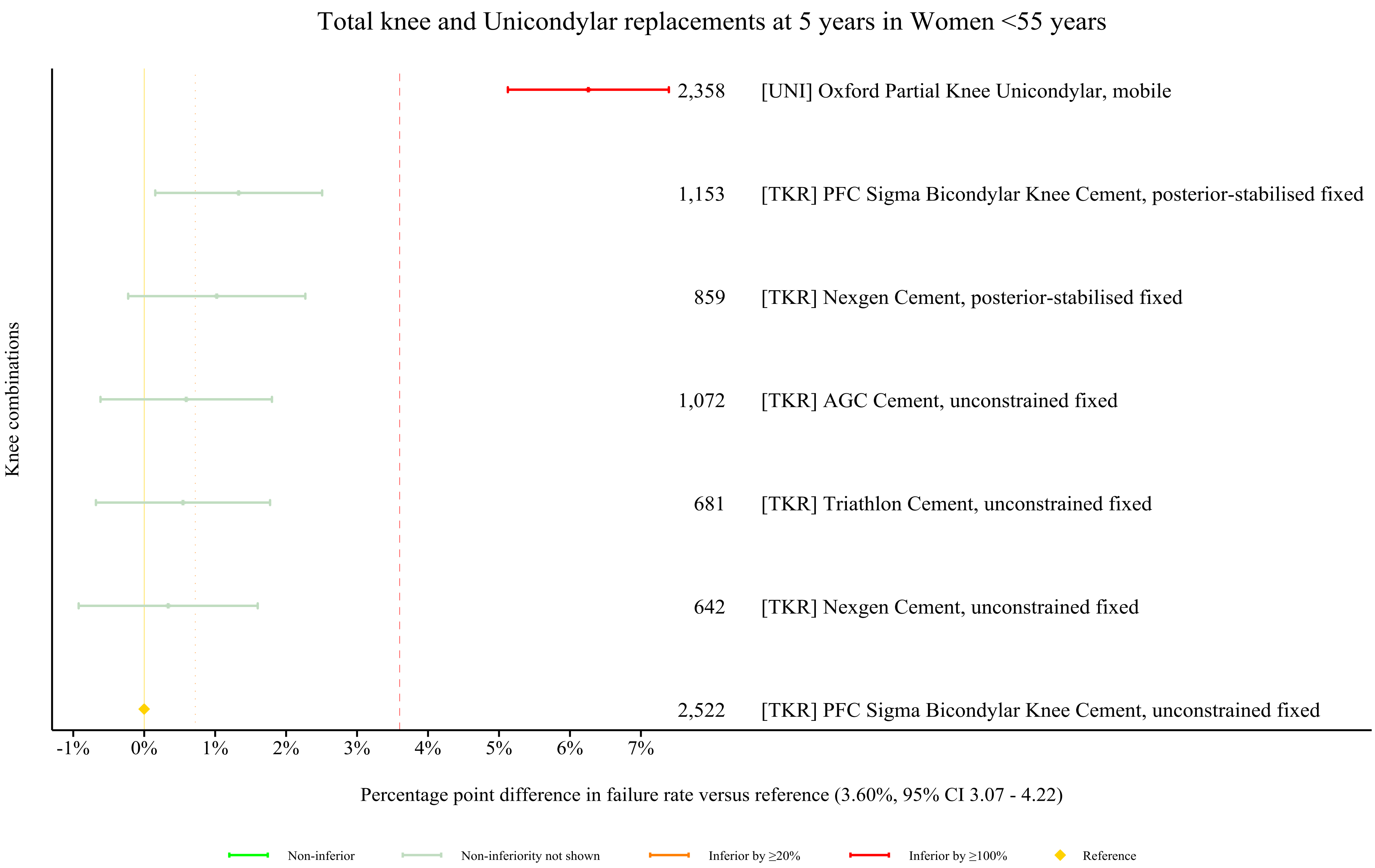

Supplementary Figure 17: Difference in cumulative revision of knee implants compared to a contemporary reference at 7 years in women less than 55 years, using all total knee and unicondylar replacements with ≥500 procedures remaining at risk.

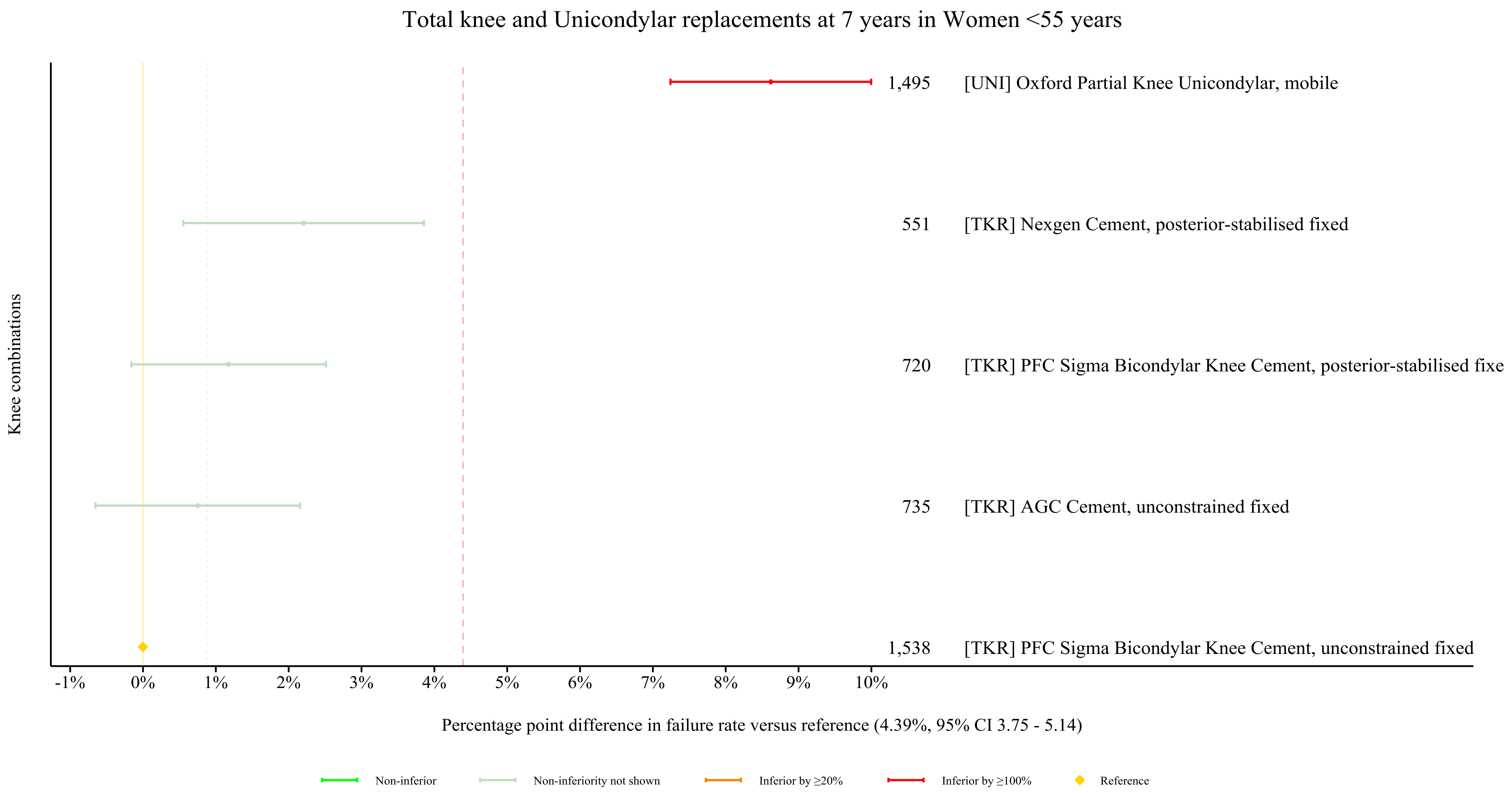

Supplementary Figure 18: Difference in cumulative revision of knee implants compared to a contemporary reference at 3 years in men aged between 55 and 75 years, using all total knee and unicondylar replacements with ≥500 procedures remaining at risk.

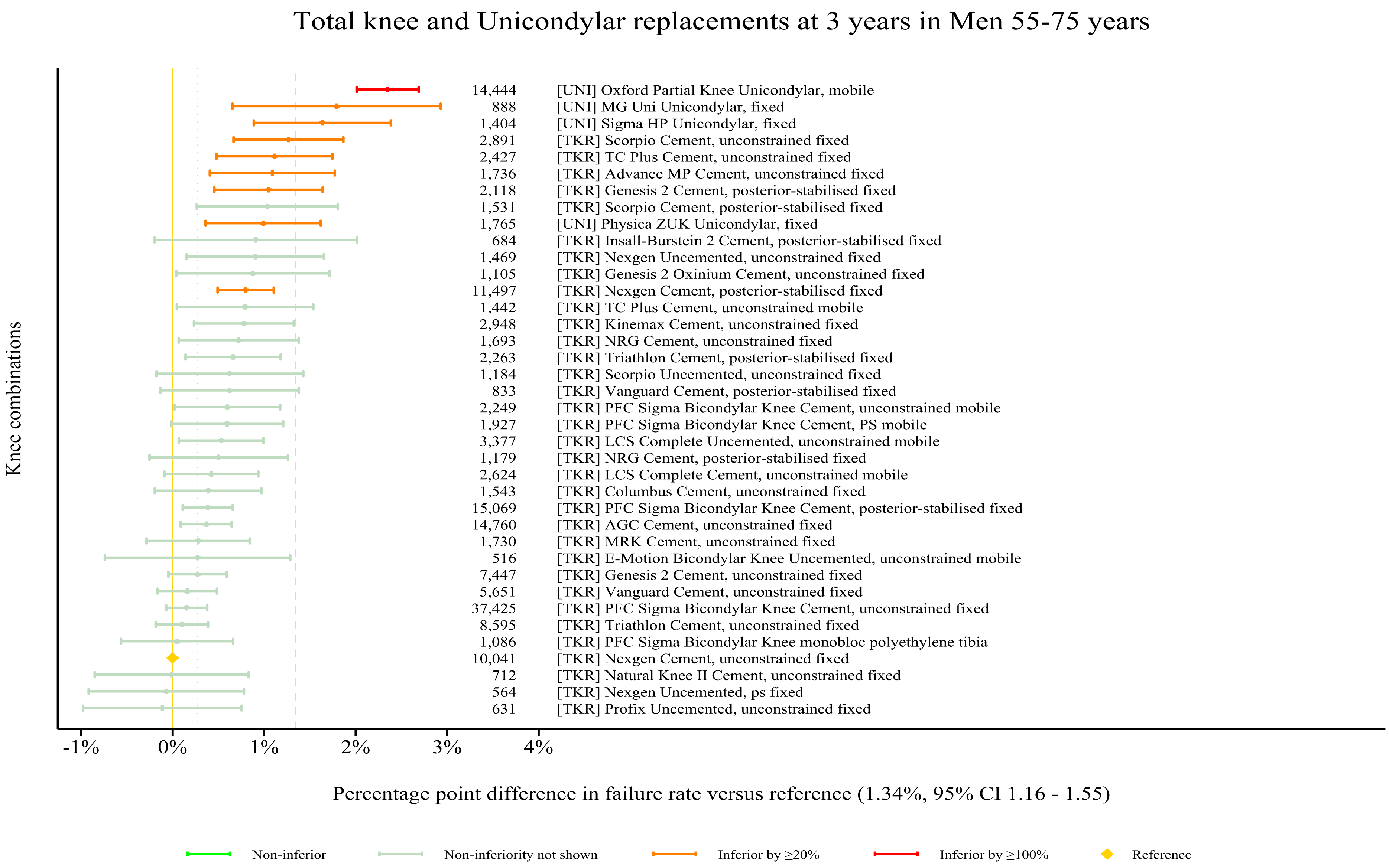

Supplementary Figure 19: Difference in cumulative revision of knee implants compared to a contemporary reference at 5 years in men aged between 55 and 75 years, using all total knee and unicondylar replacements with ≥500 procedures remaining at risk.

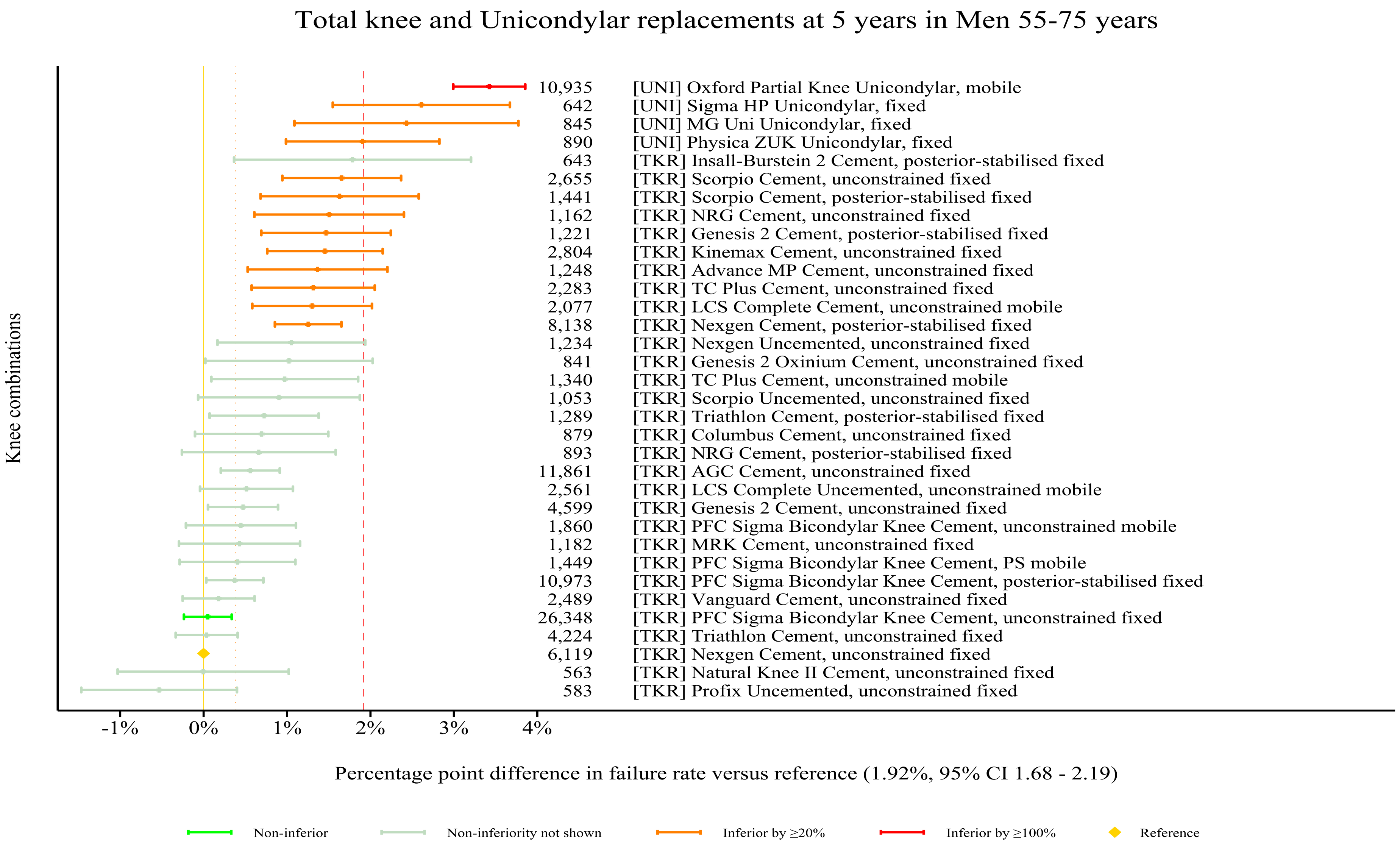

Supplementary Figure 20: Difference in cumulative revision of knee implants compared to a contemporary reference at 10 years in men aged between 55 and 75 years, using all total knee and unicondylar replacements with ≥500 procedures remaining at risk.

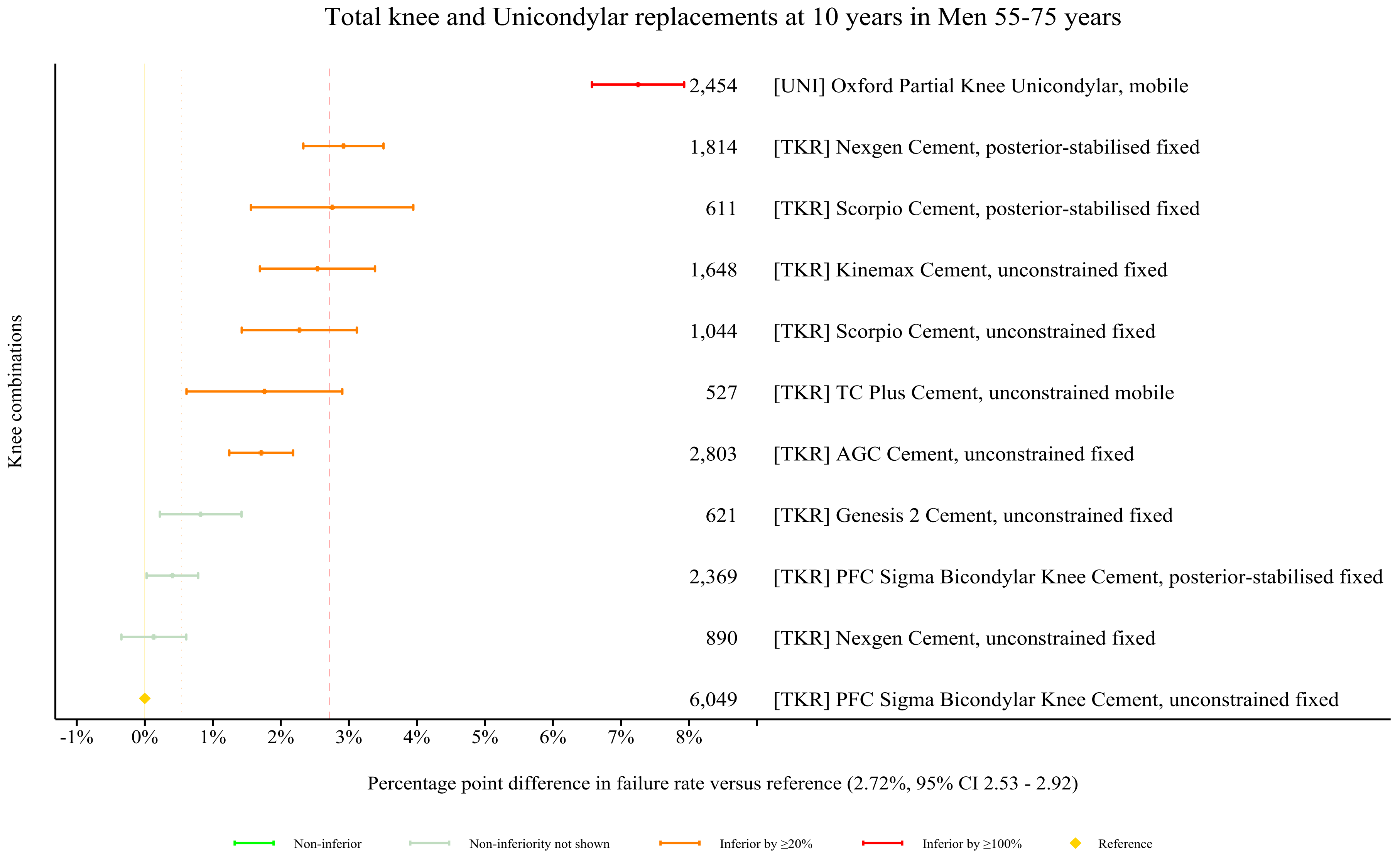

Supplementary Figure 21: Difference in cumulative revision of knee implants compared to a contemporary reference at 3 years in women aged between 55 and 75 years, using all total knee and unicondylar replacements with ≥500 procedures remaining at risk.

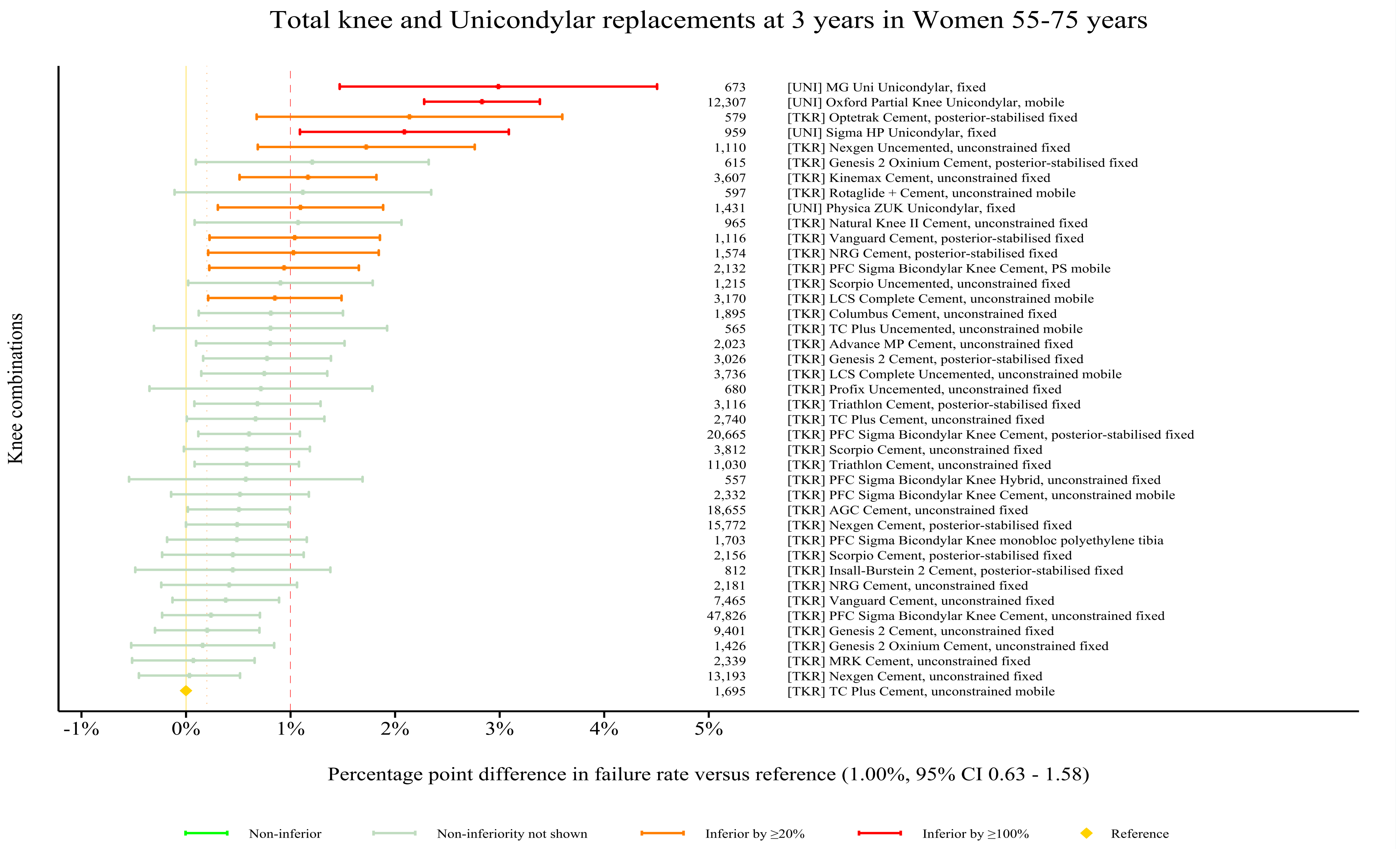

Supplementary Figure 22: Difference in cumulative revision of knee implants compared to a contemporary reference at 5 years in women aged between 55 and 75 years, using all total knee and unicondylar replacements with ≥500 procedures remaining at risk.

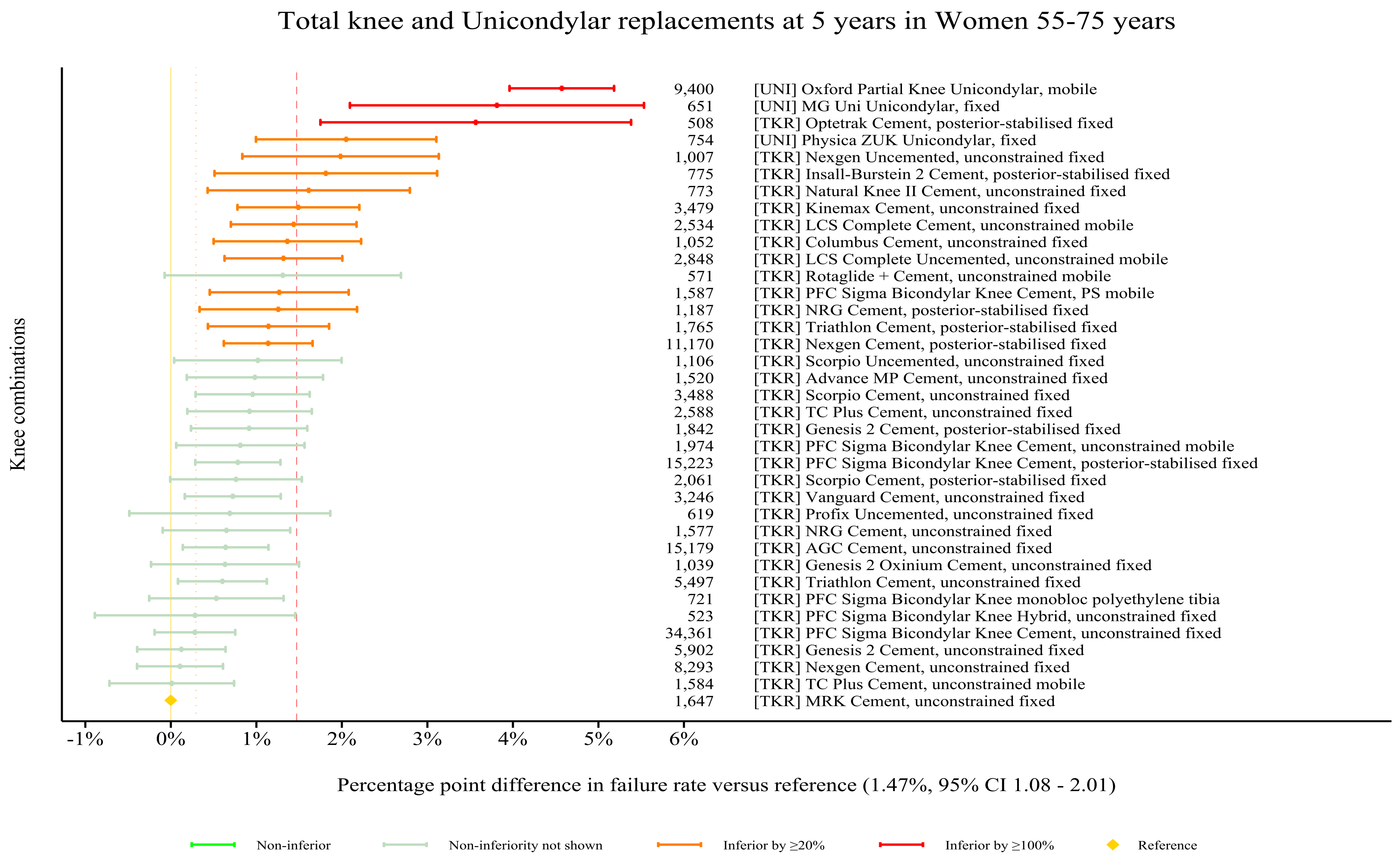

Supplementary Figure 23: Difference in cumulative revision of knee implants compared to a contemporary reference at 10 years in women aged between 55 and 75 years, using all total knee and unicondylar replacements with ≥500 procedures remaining at risk.

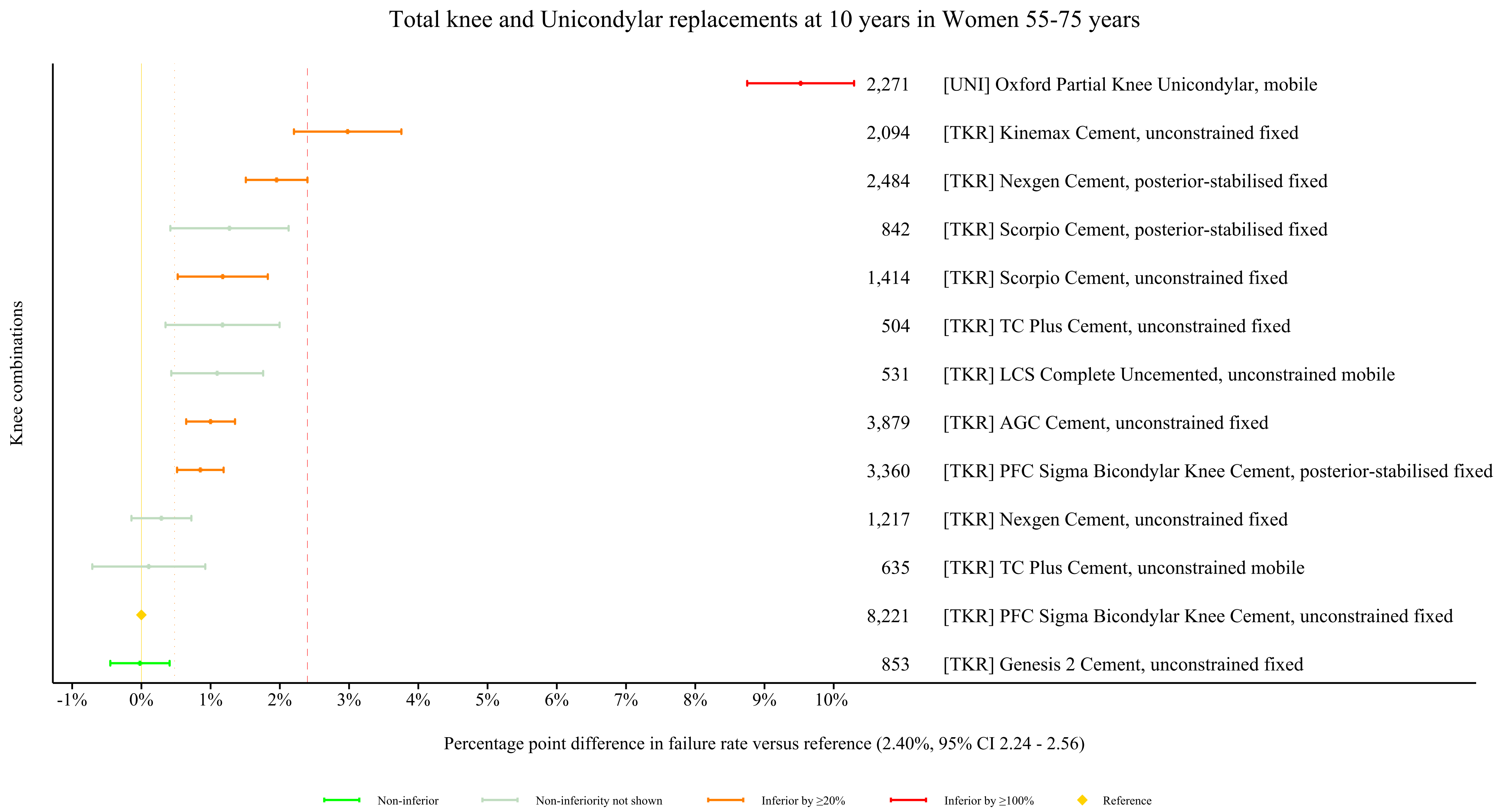

Supplementary Figure 24: Difference in cumulative revision of knee implants compared to a contemporary reference at 3 years in men aged over 75 years, using all total knee and unicondylar replacements with ≥500 procedures remaining at risk.

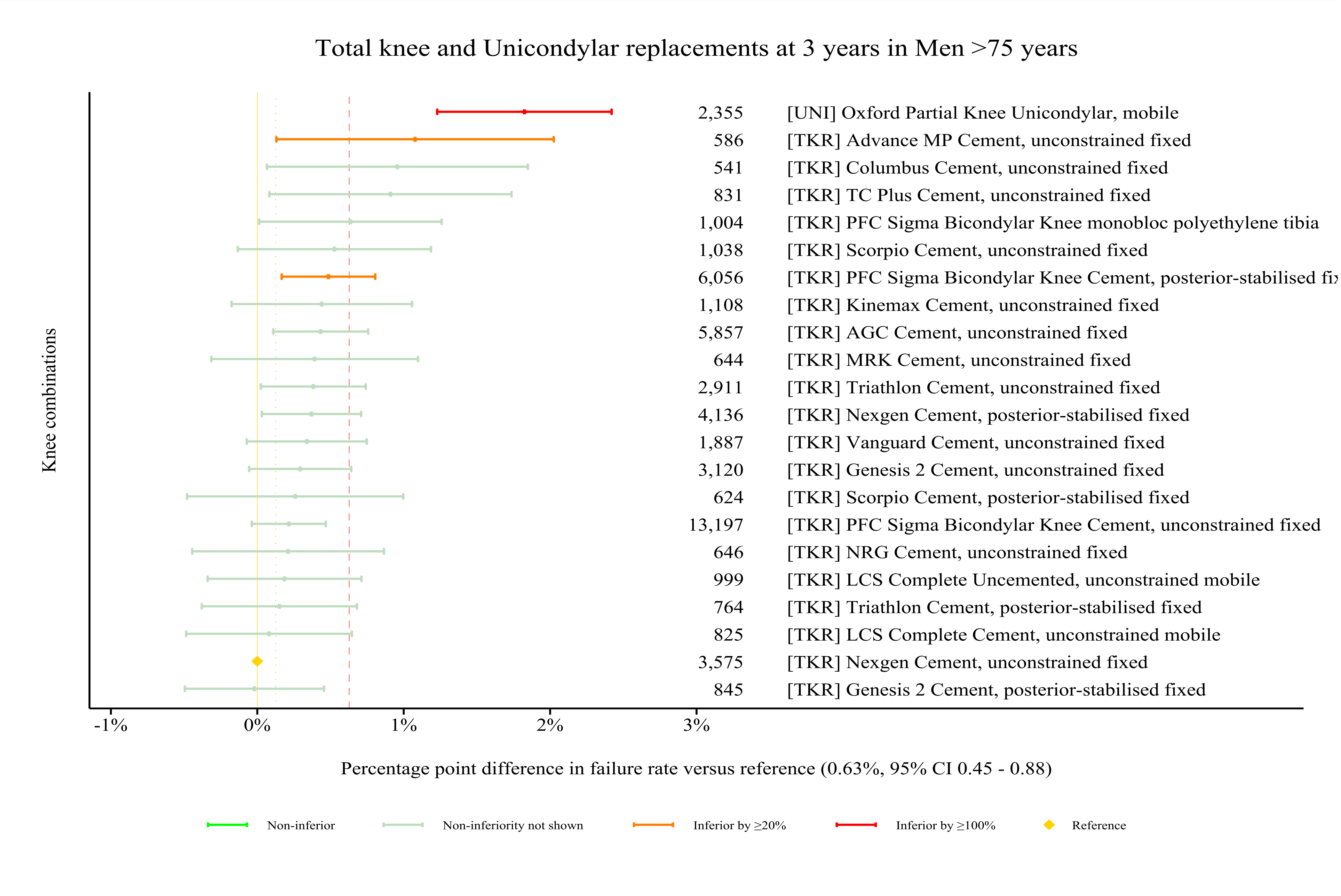

Supplementary Figure 25: Difference in cumulative revision of knee implants compared to a contemporary reference at 5 years in men aged over 75 years, using all total knee and unicondylar replacements with ≥500 procedures remaining at risk.

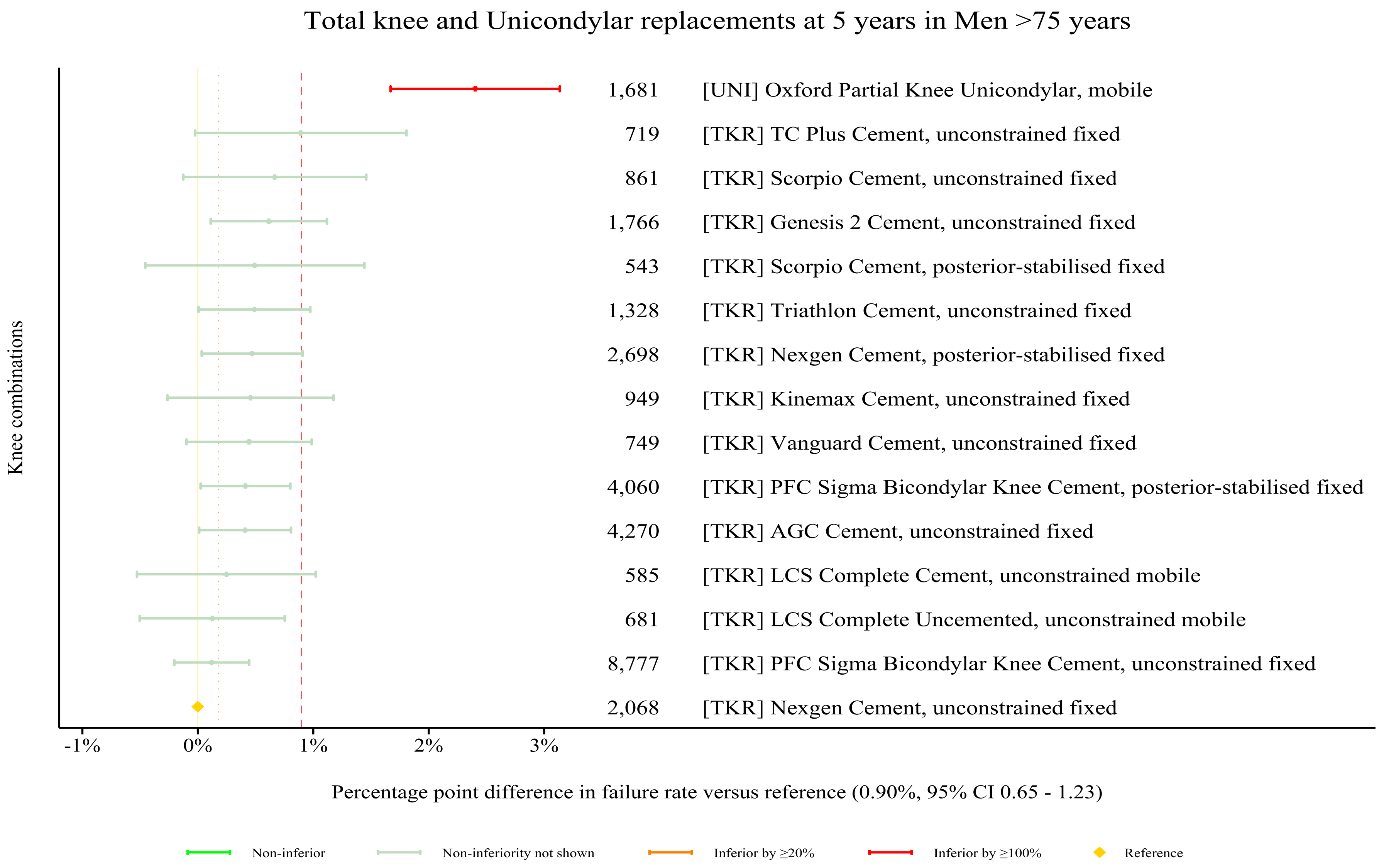

Supplementary Figure 26: Difference in cumulative revision of knee implants compared to a contemporary reference at 7 years in men aged over 75 years, using all total knee and unicondylar replacements with ≥500 procedures remaining at risk.

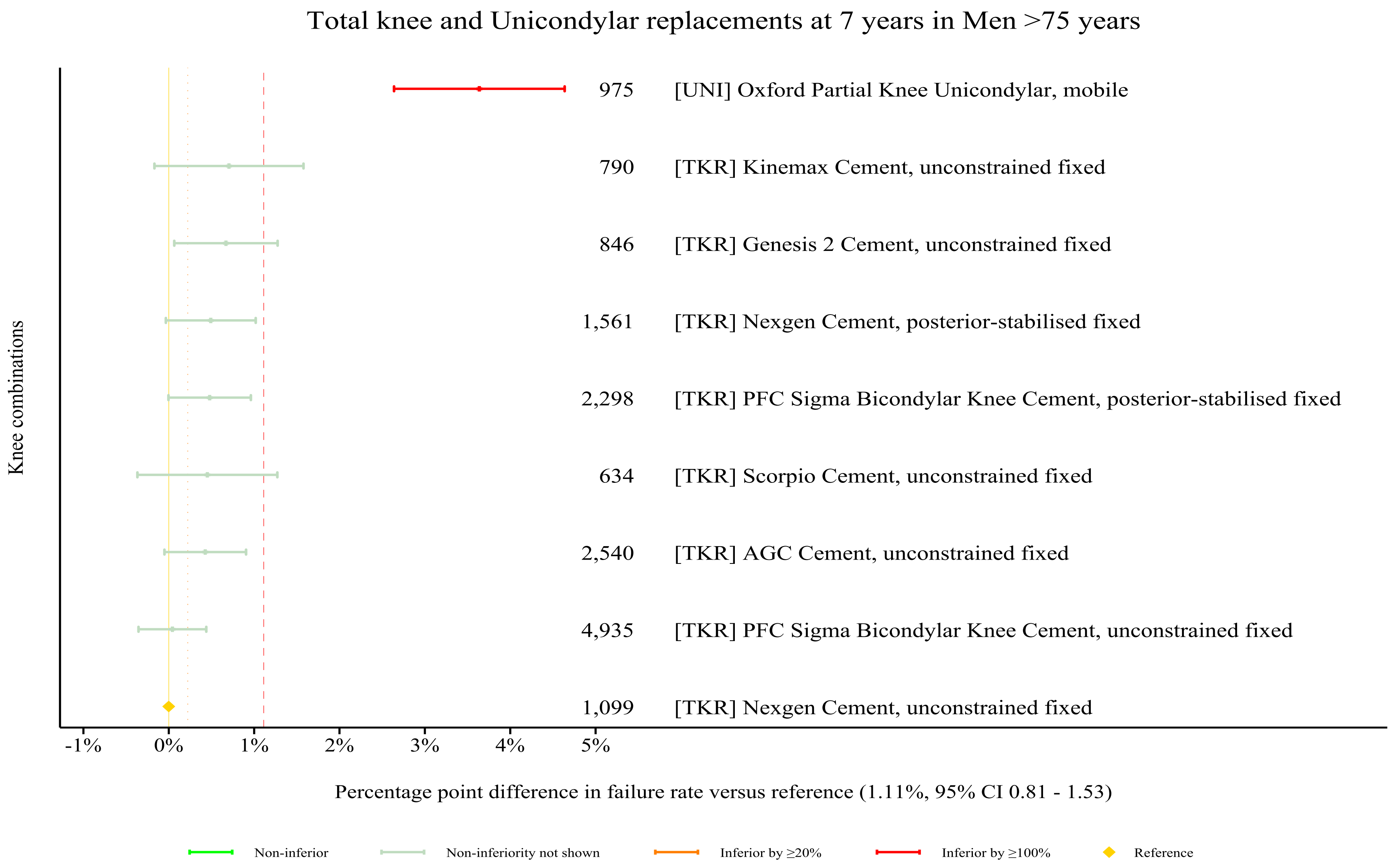

Supplementary Figure 27: Difference in cumulative revision of knee implants compared to a contemporary reference at 10 years in men aged over 75 years, using all total knee and unicondylar replacements with ≥500 procedures remaining at risk.

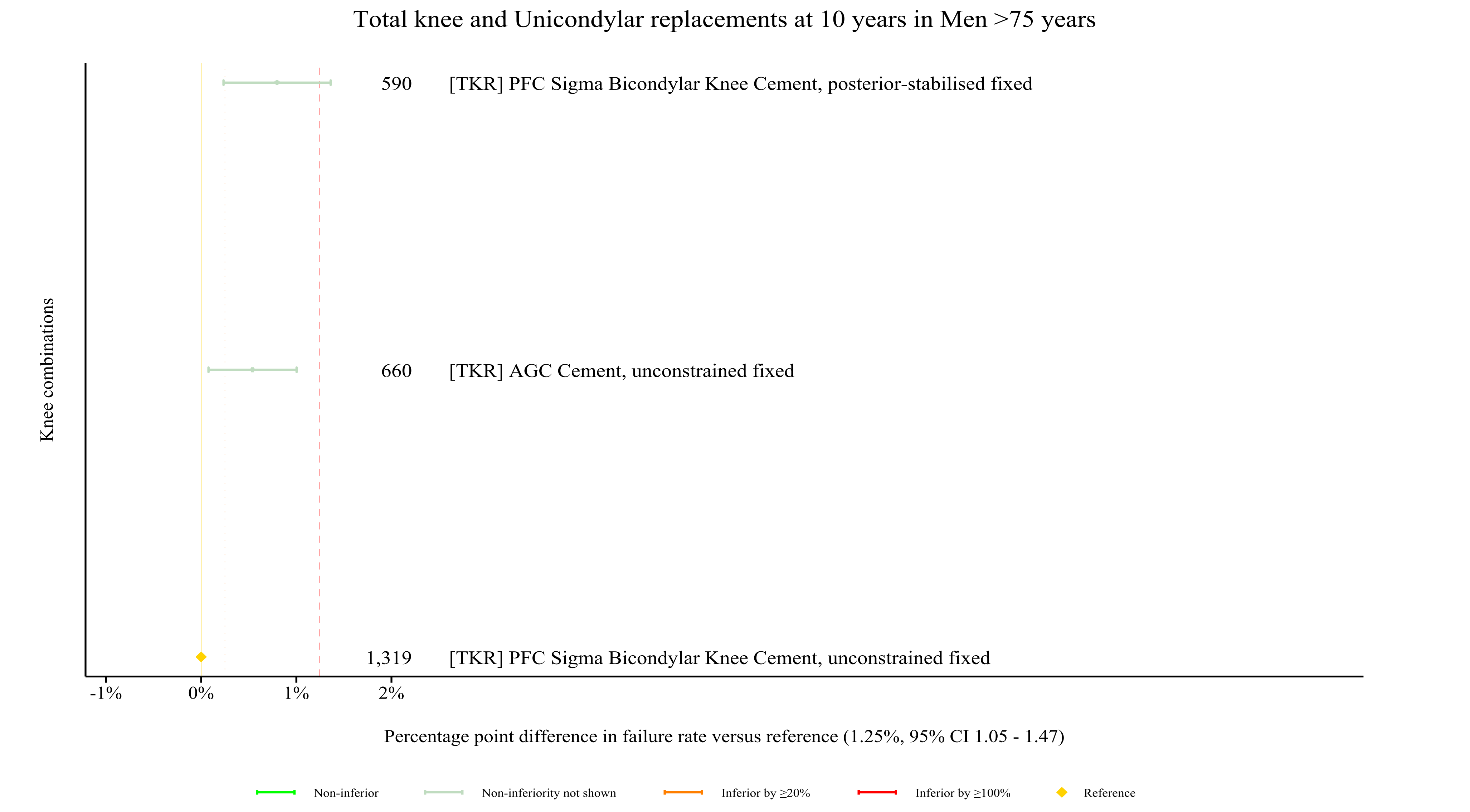

Supplementary Figure 28: Difference in cumulative revision of knee implants compared to a contemporary reference at 3 years in women aged over 75 years, using all total knee and unicondylar replacements with ≥500 procedures remaining at risk.

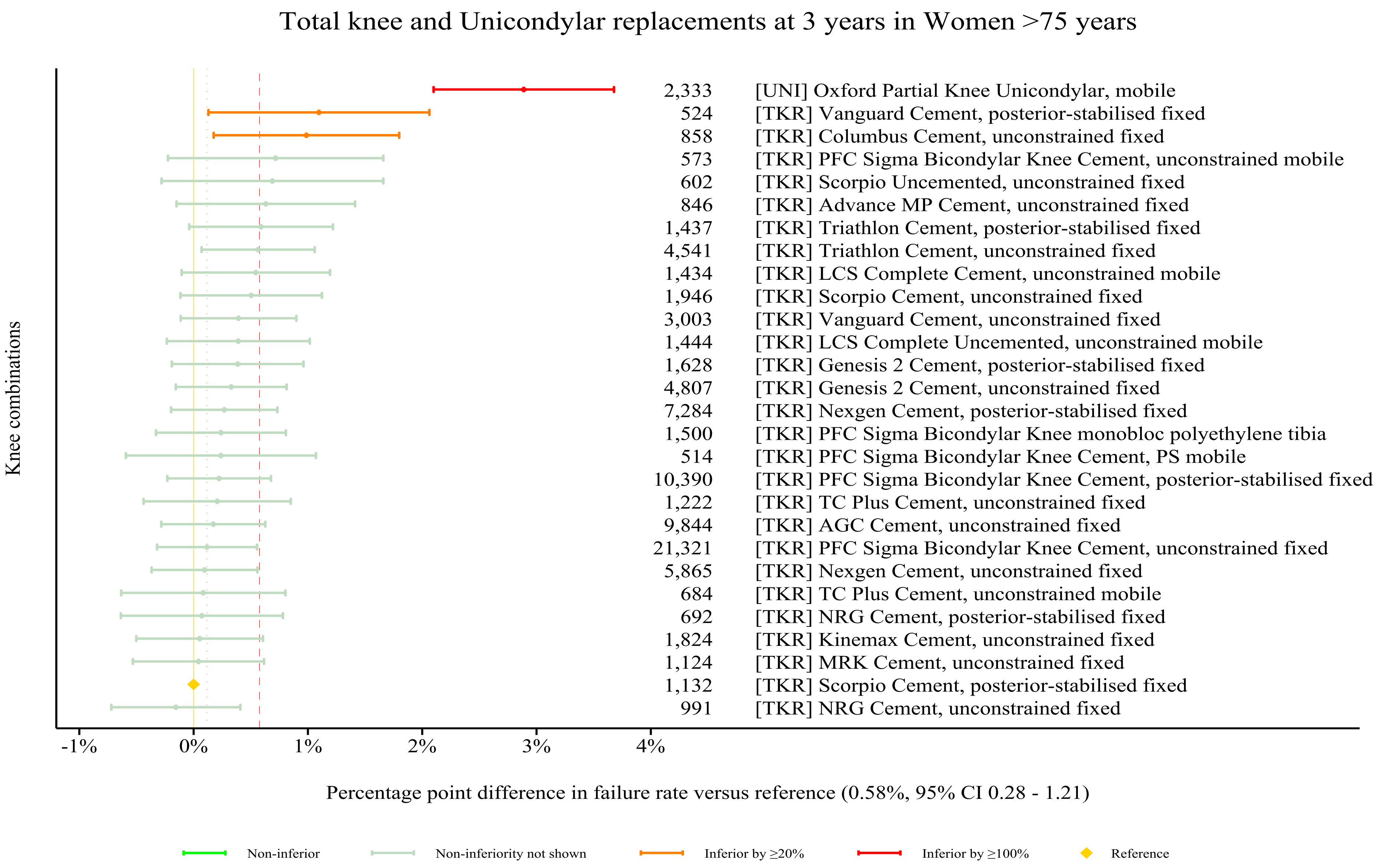

Supplementary Figure 29: Difference in cumulative revision of knee implants compared to a contemporary reference at 5 years in women aged over 75 years, using all total knee and unicondylar replacements with ≥500 procedures remaining at risk.

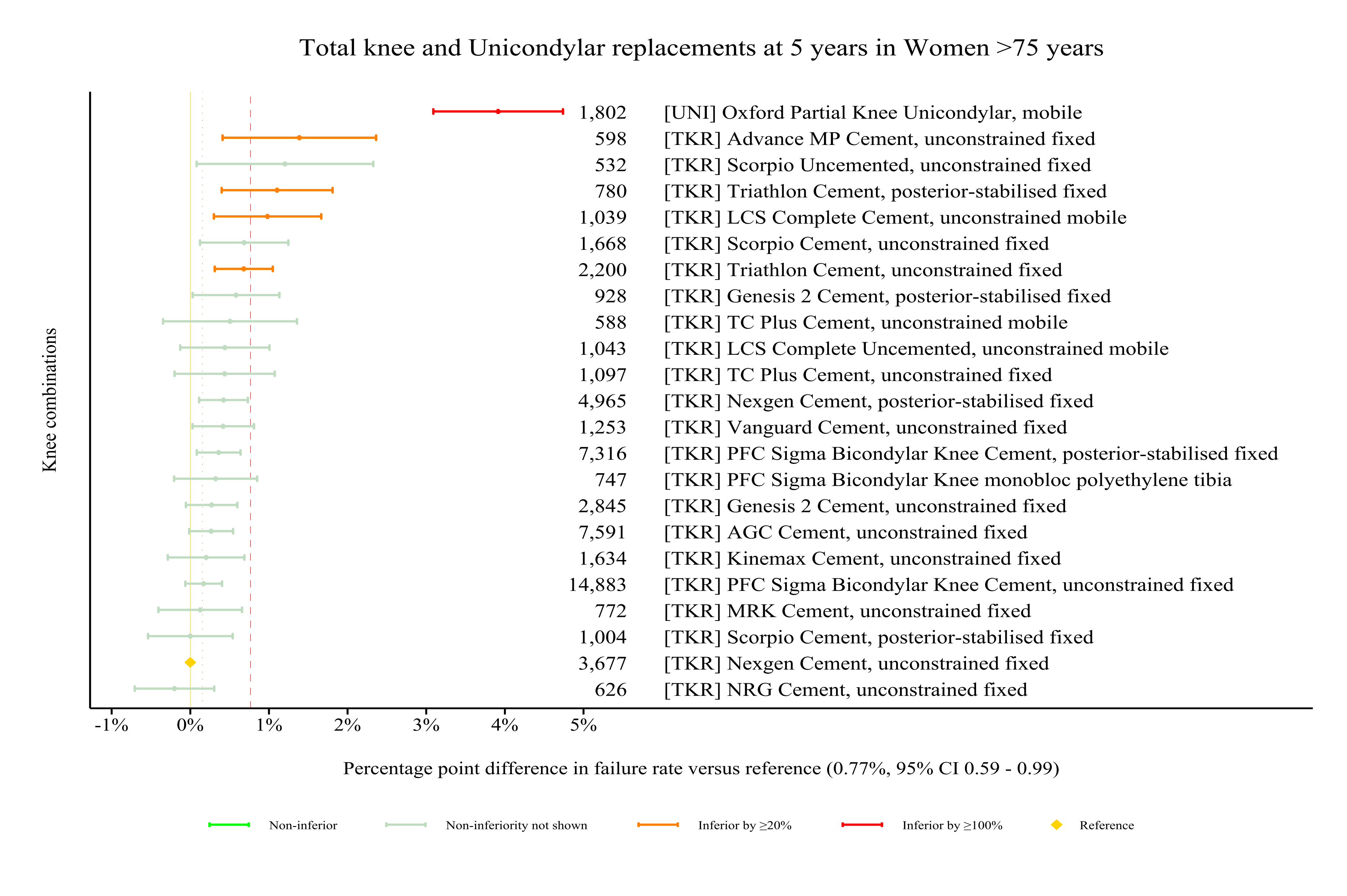

Supplementary Figure 30: Difference in cumulative revision of knee implants compared to a contemporary reference at 7 years in women aged over 75 years, using all total knee and unicondylar replacements with ≥500 procedures remaining at risk.

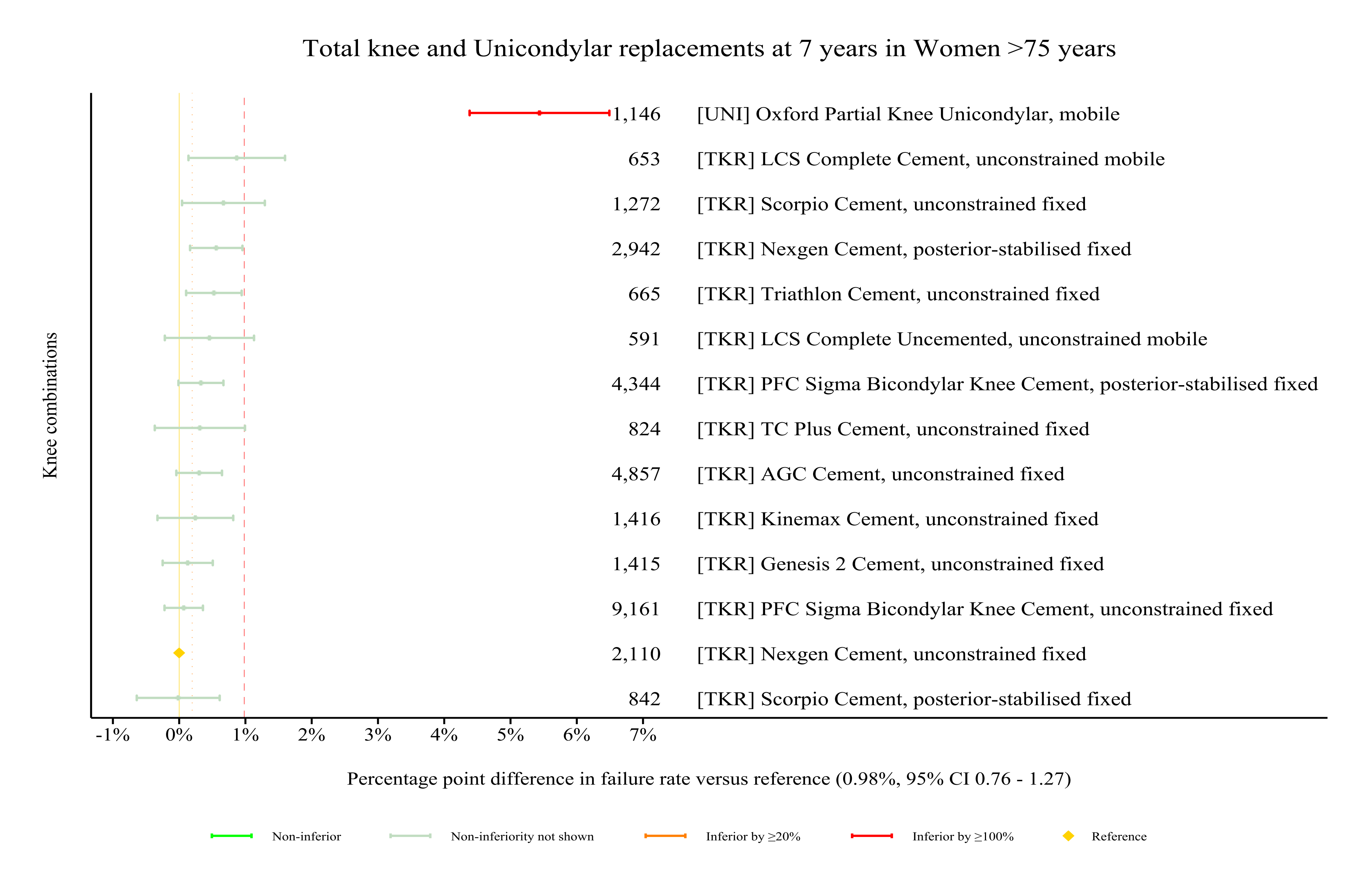

Supplementary Figure 31: Difference in cumulative revision of knee implants compared to a contemporary reference at 10 years in women aged over 75 years, using all total knee and unicondylar replacements with ≥500 procedures remaining at risk.

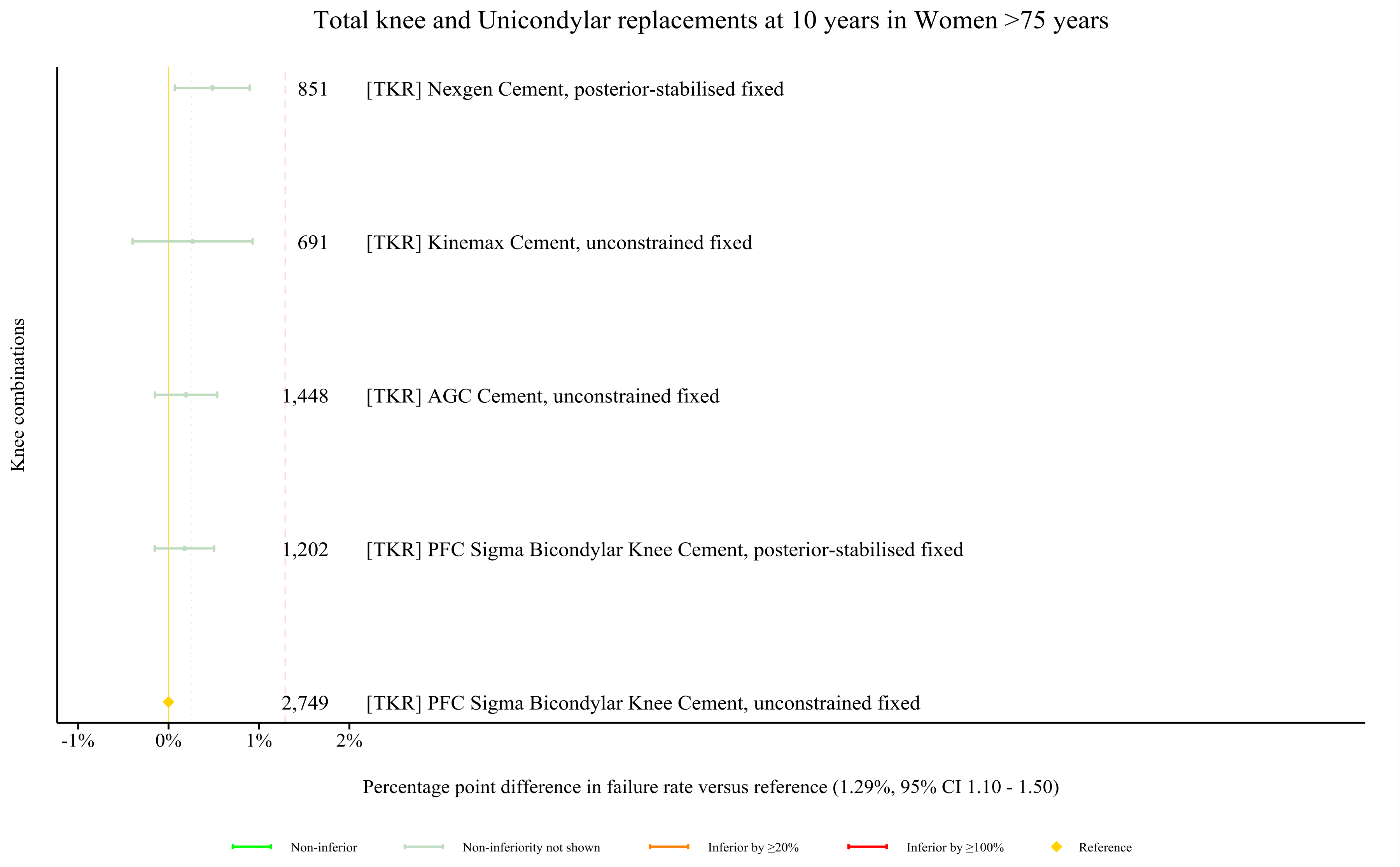

Supplementary table 1: Difference in cumulative percentage revision of knee implants compared to a contemporary benchmark at 3 years post primary for knee replacements with ≥250 procedures remaining at risk

Knee brand, bearing and constraint	Number at risk	Cumulative failure (%)	Difference in failure (%)	95% CI	Equivalence status	p-value
[TKR] NexGen Cement, unconstrained fixed	34,558	1.10	[REFERENCE]			
[TKR] ACS Uncemented, unconstrained fixed	448	3.42	2.32	[0.67 , 3.97]	Inferior by ≥20%	0.003
[TKR] ACS Uncemented, unconstrained mobile	428	3.20	2.10	[0.55 , 3.65]	Inferior by ≥20%	0.004
[TKR] AGC Cement, posterior-stabilised fixed	502	1.90	0.80	[-0.37 , 1.98]	Non-inferiority not shown	0.090
[TKR] AGC Cement, unconstrained fixed	51,355	1.43	0.33	[0.19 , 0.47]	Non-inferiority not shown	<0.001
[TKR] AGC Hybrid, unconstrained fixed	861	2.28	1.18	[0.21 , 2.15]	Non-inferiority not shown	0.008
[TKR] AGC Uncemented, unconstrained fixed	1,081	4.08	2.98	[1.83 , 4.12]	Inferior by ≥100%	<0.001
[TKR] AMK Cement, unconstrained fixed	378	0.48	-0.61	[-1.29 , 0.06]	Non-inferior	0.037
[TKR] Advance MP Cement, unconstrained fixed	5,549	2.04	0.94	[0.59 , 1.29]	Inferior by ≥20%	<0.001
[TKR] Advance MP Stature Cement, unconstrained fixed	829	1.83	0.73	[-0.07 , 1.53]	Non-inferiority not shown	0.037
[TKR] Advance PS Cement, posterior-stabilised fixed	791	2.39	1.29	[0.32 , 2.27]	Inferior by ≥20%	0.005
[TKR] Alpina Hybrid, unconstrained fixed	441	1.94	0.84	[-0.42 , 2.10]	Non-inferiority not shown	0.095
[TKR] Birmingham Knee Replacement Cement, posterior-stabilised mobile	420	1.86	0.76	[-0.30 , 1.81]	Non-inferiority not shown	0.080
[TKR] Columbus Cement, unconstrained fixed	5,128	1.78	0.68	[0.36 , 1.01]	Inferior by ≥20%	<0.001
[TKR] E-Motion Bicondylar Knee Cement, unconstrained mobile	702	3.01	1.92	[0.78 , 3.05]	Inferior by ≥20%	<0.001
[TKR] E-Motion Bicondylar Knee Uncemented, unconstrained mobile	1,393	2.03	0.93	[0.25 , 1.61]	Inferior by ≥20%	0.004
[TKR] FS Cement, unconstrained fixed	741	1.54	0.45	[-0.43 , 1.32]	Non-inferiority not shown	0.158
[TKR] Genesis 2 Cement, posterior-stabilised fixed	7,864	1.75	0.65	[0.38 , 0.91]	Inferior by ≥20%	<0.001
[TKR] Genesis 2 Cement, unconstrained fixed	25,556	1.27	0.17	[0.02 , 0.33]	Non-inferiority not shown	0.014
[TKR] Genesis 2 Cement, unconstrained mobile	887	1.74	0.64	[-0.21 , 1.49]	Non-inferiority not shown	0.070
[TKR] Genesis 2 Oxinium Cement, posterior-stabilised fixed	1,681	2.95	1.85	[1.12 , 2.57]	Inferior by ≥100%	<0.001
[TKR] Genesis 2 Oxinium Cement, unconstrained fixed	3,633	1.94	0.84	[0.42 , 1.27]	Inferior by ≥20%	<0.001
[TKR] Innex Uncemented, unconstrained mobile	358	1.62	0.52	[-0.77 , 1.80]	Non-inferiority not shown	0.216
[TKR] Insall-Burstein 2 Cement, posterior-stabilised fixed	2,226	1.47	0.37	[-0.13 , 0.87]	Non-inferiority not shown	0.072
[TKR] Journey Oxinium Cement, posterior-stabilised fixed	744	4.59	3.49	[2.03 , 4.96]	Inferior by ≥100%	<0.001
[TKR] Kinemax Cement, unconstrained fixed	9,935	1.78	0.68	[0.41 , 0.95]	Inferior by ≥20%	<0.001
[TKR] LCS Cement, unconstrained mobile	611	1.39	0.29	[-0.62 , 1.20]	Non-inferiority not shown	0.264
[TKR] LCS Complete Cement, unconstrained mobile	8,451	1.62	0.52	[0.25 , 0.80]	Inferior by ≥20%	<0.001
[TKR] LCS Complete Hybrid, unconstrained mobile	381	1.68	0.59	[-0.47 , 1.64]	Non-inferiority not shown	0.137

[TKR] LCS Complete Uncemented, unconstrained mobile	10,293	1.75	0.65	[0.40 , 0.91]	Inferior by ≥20%	<0.001
[TKR] LCS Uncemented, unconstrained mobile	1,237	1.87	0.77	[0.02 , 1.52]	Non-inferiority not shown	0.022
[TKR] Legion Cement, unconstrained fixed	395	1.50	0.40	[-0.53 , 1.33]	Non-inferiority not shown	0.202
[TKR] MBK Cement, unconstrained mobile	372	1.30	0.20	[-0.94 , 1.33]	Non-inferiority not shown	0.365
[TKR] MRK Cement, unconstrained fixed	6,170	1.20	0.10	[-0.16 , 0.35]	Non-inferiority not shown	0.231
[TKR] Maxim Cement, posterior-stabilised fixed	582	1.48	0.38	[-0.58 , 1.35]	Non-inferiority not shown	0.220
[TKR] Maxim Cement, unconstrained fixed	1,244	1.48	0.38	[-0.29 , 1.04]	Non-inferiority not shown	0.134
[TKR] NRG Cement, posterior-stabilised fixed	4,141	1.71	0.61	[0.22 , 1.00]	Inferior by ≥20%	0.001
[TKR] NRG Cement, unconstrained fixed	5,860	1.48	0.38	[0.08 , 0.68]	Non-inferiority not shown	0.007
[TKR] Natural Knee II Cement, unconstrained fixed	2,481	1.37	0.27	[-0.18 , 0.73]	Non-inferiority not shown	0.120
[TKR] NexGen Cement, posterior-stabilised mobile	813	2.83	1.73	[0.69 , 2.77]	Inferior by ≥20%	0.001
[TKR] NexGen Cement, posterior-stabilised fixed	40,703	1.61	0.51	[0.36 , 0.65]	Inferior by ≥20%	<0.001
[TKR] NexGen Hinge Type Cement, unconstrained mobile	292	1.65	0.56	[-0.69 , 1.80]	Non-inferiority not shown	0.191
[TKR] NexGen Hybrid, unconstrained fixed	963	1.07	-0.03	[-0.66 , 0.61]	Non-inferiority not shown	0.466
[TKR] NexGen LCCK Cement, constrained condylar	282	2.77	1.67	[0.01 , 3.33]	Non-inferiority not shown	0.024
[TKR] NexGen Uncemented, posterior-stabilised fixed	1,424	1.72	0.62	[-0.00 , 1.24]	Non-inferiority not shown	0.025
[TKR] NexGen Uncemented, unconstrained fixed	3,540	2.55	1.45	[0.95 , 1.96]	Inferior by ≥20%	<0.001
[TKR] NexGen monobloc polyethylene tibia	530	0.67	-0.43	[-1.03 , 0.18]	Non-inferior	0.083
[TKR] Optetrak Cement, posterior-stabilised fixed	1,495	2.66	1.56	[0.76 , 2.36]	Inferior by ≥20%	<0.001
[TKR] Optetrak Cement, unconstrained fixed	360	3.73	2.63	[0.77 , 4.48]	Inferior by ≥20%	0.003
[TKR] PFC Sigma Bicondylar Knee Cement, posterior-stabilised mobile	5,733	2.12	1.02	[0.65 , 1.38]	Inferior by ≥20%	<0.001
[TKR] PFC Sigma Bicondylar Knee Cement, constrained condylar	560	1.73	0.64	[-0.31 , 1.58]	Non-inferiority not shown	0.094
[TKR] PFC Sigma Bicondylar Knee Cement, posterior-stabilised fixed	54,952	1.51	0.41	[0.28 , 0.55]	Inferior by ≥20%	<0.001
[TKR] PFC Sigma Bicondylar Knee Cement, unconstrained fixed	126,134	1.25	0.15	[0.04 , 0.26]	Non-inferiority not shown	0.005
[TKR] PFC Sigma Bicondylar Knee Cement, unconstrained mobile	6,571	1.93	0.83	[0.51 , 1.16]	Inferior by ≥20%	<0.001
[TKR] PFC Sigma Bicondylar Knee Hybrid, unconstrained fixed	1,532	1.18	0.08	[-0.45 , 0.62]	Non-inferiority not shown	0.381
[TKR] PFC Sigma Bicondylar Knee Uncemented, unconstrained mobile	604	1.44	0.34	[-0.51 , 1.20]	Non-inferiority not shown	0.216
[TKR] PFC Sigma Bicondylar Knee monobloc polyethylene tibia	5,365	1.30	0.21	[-0.07 , 0.48]	Non-inferiority not shown	0.070
[TKR] Profix Cement, unconstrained fixed	883	2.05	0.95	[0.03 , 1.86]	Non-inferiority not shown	0.021
[TKR] Profix Oxinium monobloc polyethylene tibia	570	3.03	1.93	[0.55 , 3.32]	Inferior by ≥20%	0.003
[TKR] Profix Uncemented, unconstrained fixed	2,137	1.26	0.16	[-0.32 , 0.63]	Non-inferiority not shown	0.258
[TKR] Profix monobloc polyethylene tibia	651	0.72	-0.37	[-1.01 , 0.27]	Non-inferiority not shown	0.126
[TKR] Rotaglide + Cement, unconstrained mobile	1,576	2.83	1.73	[0.92 , 2.53]	Inferior by ≥20%	<0.001
[TKR] Rotaglide Cement, unconstrained mobile	1,051	2.05	0.96	[0.15 , 1.76]	Non-inferiority not shown	0.010
[TKR] Scorpio Cement, posterior-stabilised mobile	1,289	1.49	0.39	[-0.26 , 1.05]	Non-inferiority not shown	0.121
[TKR] Scorpio Cement, posterior-stabilised fixed	5,673	1.59	0.49	[0.16 , 0.82]	Non-inferiority not shown	0.002

[TKR] Scorpio Cement, unconstrained fixed	10,083	1.85	0.75	[0.48 , 1.03]	Inferior by ≥20%	<0.001
[TKR] Scorpio Cement, unconstrained mobile	1,094	2.54	1.44	[0.52 , 2.36]	Inferior by ≥20%	0.001
[TKR] Scorpio Hybrid, unconstrained fixed	1,005	1.80	0.70	[-0.11 , 1.50]	Non-inferiority not shown	0.045
[TKR] Scorpio Uncemented, unconstrained fixed	3,530	1.79	0.69	[0.26 , 1.13]	Inferior by ≥20%	0.001
[TKR] TC Plus Cement, unconstrained fixed	7,501	1.89	0.79	[0.47 , 1.10]	Inferior by ≥20%	<0.001
[TKR] TC Plus Cement, unconstrained mobile	4,385	1.44	0.35	[-0.01 , 0.70]	Non-inferiority not shown	0.027
[TKR] TC Plus Uncemented, unconstrained mobile	1,439	1.52	0.42	[-0.17 , 1.00]	Non-inferiority not shown	0.083
[TKR] Triathlon Cement, posterior-stabilised fixed	8,196	1.73	0.63	[0.37 , 0.89]	Inferior by ≥20%	<0.001
[TKR] Triathlon Cement, unconstrained fixed	29,202	1.48	0.38	[0.23 , 0.53]	Inferior by ≥20%	<0.001
[TKR] Triathlon Uncemented, unconstrained fixed	742	2.75	1.66	[0.63 , 2.69]	Inferior by ≥20%	0.001
[TKR] Vanguard Cement, constrained condylar	436	1.08	-0.02	[-0.72 , 0.69]	Non-inferiority not shown	0.479
[TKR] Vanguard Cement, posterior-stabilised fixed	2,988	1.91	0.81	[0.41 , 1.21]	Inferior by ≥20%	<0.001
[TKR] Vanguard Cement, unconstrained fixed	19,392	1.37	0.27	[0.11 , 0.44]	Non-inferiority not shown	0.001
[TKR] balanSys CR cemented Cement, unconstrained fixed	293	1.72	0.63	[-0.75 , 2.00]	Non-inferiority not shown	0.186
[UNI] AMC/Uniglide Unicondylar, fixed	1,070	2.98	1.88	[0.92 , 2.85]	Inferior by ≥20%	<0.001
[UNI] AMC/Uniglide Unicondylar, mobile	1,195	8.47	7.37	[5.91 , 8.84]	Inferior by ≥100%	<0.001
[UNI] EIUS Unicondylar, fixed	314	9.39	8.29	[5.24 , 11.34]	Inferior by ≥100%	<0.001
[UNI] Genesis Uni Oxinium Unicondylar, fixed	439	3.61	2.51	[0.87 , 4.15]	Inferior by ≥20%	0.001
[UNI] MG Uni Unicondylar, fixed	2,206	3.96	2.86	[2.06 , 3.66]	Inferior by ≥100%	<0.001
[UNI] Oxford Partial Knee Unicondylar, fixed	378	3.57	2.47	[0.79 , 4.16]	Inferior by ≥20%	0.002
[UNI] Oxford Partial Knee Unicondylar, mobile	37,382	4.07	2.98	[2.77 , 3.18]	Inferior by ≥100%	<0.001
[UNI] Physica ZUK Unicondylar, fixed	4,708	2.45	1.35	[0.96 , 1.73]	Inferior by ≥20%	<0.001
[UNI] Preservation Unicondylar, fixed	1,105	6.89	5.79	[4.36 , 7.23]	Inferior by ≥100%	<0.001
[UNI] Sigma HP Unicondylar, fixed	3,561	3.56	2.46	[1.95 , 2.97]	Inferior by ≥100%	<0.001
[UNI] Sled Unicondylar, fixed	682	6.23	5.14	[3.44 , 6.83]	Inferior by ≥100%	<0.001
[UNI] Triathlon Uni Unicondylar, fixed	433	5.69	4.59	[2.76 , 6.43]	Inferior by ≥100%	<0.001

Supplementary table 2: Difference in cumulative percentage revision of knee implants compared to a contemporary benchmark at 5 years post primary for knee replacements with ≥250 procedures remaining at risk

Knee brand, bearing and constraint	Number at risk	Cumulative failure (%)	Difference in failure (%)	95% CI	Equivalence status	p-value
[TKR] Profix Uncemented, unconstrained fixed	1,910	1.54	[REFERENCE]			
[TKR] ACS Uncemented, unconstrained fixed	431	3.86	2.32	[0.49 , 4.14]	Inferior by ≥20%	0.006
[TKR] ACS Uncemented, unconstrained mobile	347	3.66	2.12	[0.37 , 3.87]	Inferior by ≥20%	0.009
[TKR] AGC Cement, posterior-stabilised fixed	440	2.71	1.17	[-0.33 , 2.66]	Non-inferiority not shown	0.063
[TKR] AGC Cement, unconstrained fixed	40,694	2.03	0.49	[-0.04 , 1.02]	Non-inferiority not shown	0.035
[TKR] AGC Hybrid, unconstrained fixed	799	2.86	1.32	[0.12 , 2.52]	Non-inferiority not shown	0.016
[TKR] AGC Uncemented, unconstrained fixed	969	5.10	3.56	[2.18 , 4.94]	Inferior by ≥100%	<0.001
[TKR] AMK Cement, unconstrained fixed	350	1.02	-0.52	[-1.64 , 0.60]	Non-inferiority not shown	0.180
[TKR] Advance MP Cement, unconstrained fixed	3,988	2.79	1.25	[0.59 , 1.91]	Inferior by ≥20%	<0.001
[TKR] Advance MP Stature Cement, unconstrained fixed	452	3.20	1.66	[0.36 , 2.96]	Inferior by ≥20%	0.006
[TKR] Advance PS Cement, posterior-stabilised fixed	619	3.19	1.65	[0.38 , 2.91]	Inferior by ≥20%	0.005
[TKR] Alpina Hybrid, unconstrained fixed	390	2.19	0.65	[-0.79 , 2.09]	Non-inferiority not shown	0.189
[TKR] Birmingham Knee Replacement Cement, posterior-stabilised mobile	270	2.76	1.21	[-0.34 , 2.76]	Non-inferiority not shown	0.062
[TKR] Columbus Cement, unconstrained fixed	2,818	2.51	0.97	[0.31 , 1.63]	Inferior by ≥20%	0.002
[TKR] E-Motion Bicondylar Knee Cement, unconstrained mobile	354	3.92	2.38	[0.91 , 3.85]	Inferior by ≥20%	0.001
[TKR] E-Motion Bicondylar Knee Uncemented, unconstrained mobile	1,061	2.85	1.31	[0.32 , 2.29]	Inferior by ≥20%	0.005
[TKR] FS Cement, unconstrained fixed	701	2.09	0.54	[-0.59 , 1.68]	Non-inferiority not shown	0.174
[TKR] Genesis 2 Cement, posterior-stabilised fixed	4,569	2.47	0.93	[0.32 , 1.54]	Inferior by ≥20%	0.001
[TKR] Genesis 2 Cement, unconstrained fixed	15,556	1.75	0.21	[-0.33 , 0.75]	Non-inferiority not shown	0.222
[TKR] Genesis 2 Cement, unconstrained mobile	774	2.31	0.77	[-0.34 , 1.88]	Non-inferiority not shown	0.086
[TKR] Genesis 2 Oxinium Cement, posterior-stabilised fixed	1,030	4.23	2.69	[1.63 , 3.74]	Inferior by ≥100%	<0.001
[TKR] Genesis 2 Oxinium Cement, unconstrained fixed	2,660	3.03	1.49	[0.74 , 2.24]	Inferior by ≥20%	<0.001
[TKR] Innex Uncemented, unconstrained mobile	348	2.45	0.91	[-0.76 , 2.57]	Non-inferiority not shown	0.142
[TKR] Insall-Burstein 2 Cement, posterior-stabilised fixed	2,067	2.74	1.20	[0.35 , 2.05]	Inferior by ≥20%	0.003
[TKR] Journey Oxinium Cement, posterior-stabilised fixed	513	6.26	4.72	[2.93 , 6.52]	Inferior by ≥100%	<0.001
[TKR] Kinemax Cement, unconstrained fixed	9,295	2.70	1.15	[0.55 , 1.76]	Inferior by ≥20%	<0.001
[TKR] LCS Cement, unconstrained mobile	568	1.88	0.34	[-0.83 , 1.52]	Non-inferiority not shown	0.285
[TKR] LCS Complete Cement, unconstrained mobile	6,533	2.73	1.19	[0.57 , 1.81]	Inferior by ≥20%	<0.001
[TKR] LCS Complete Hybrid, unconstrained mobile	268	2.35	0.81	[-0.68 , 2.29]	Non-inferiority not shown	0.143

[TKR] LCS Complete Uncemented, unconstrained mobile	7,677	2.50	0.95	[0.36 , 1.55]	Inferior by ≥20%	0.001
[TKR] LCS Uncemented, unconstrained mobile	1,159	2.44	0.89	[-0.10 , 1.89]	Non-inferiority not shown	0.039
[TKR] MBK Cement, unconstrained mobile	360	2.63	1.09	[-0.60 , 2.78]	Non-inferiority not shown	0.103
[TKR] MRK Cement, unconstrained fixed	4,250	1.64	0.09	[-0.50 , 0.69]	Non-inferiority not shown	0.377
[TKR] Maxim Cement, posterior-stabilised fixed	480	3.12	1.58	[0.06 , 3.09]	Non-inferiority not shown	0.021
[TKR] Maxim Cement, unconstrained fixed	1,109	2.13	0.59	[-0.36 , 1.54]	Non-inferiority not shown	0.111
[TKR] NRG Cement, posterior-stabilised fixed	3,075	2.40	0.86	[0.17 , 1.55]	Non-inferiority not shown	0.007
[TKR] NRG Cement, unconstrained fixed	3,999	2.36	0.82	[0.17 , 1.46]	Non-inferiority not shown	0.006
[TKR] Natural Knee II Cement, unconstrained fixed	1,950	2.21	0.67	[-0.11 , 1.44]	Non-inferiority not shown	0.046
[TKR] NexGen Cement, posterior-stabilised mobile	680	3.60	2.06	[0.75 , 3.37]	Inferior by ≥20%	0.001
[TKR] NexGen Cement, posterior-stabilised fixed	28,335	2.54	0.99	[0.46 , 1.53]	Inferior by ≥20%	<0.001
[TKR] NexGen Cement, unconstrained fixed	21,242	1.60	0.05	[-0.48 , 0.58]	Non-inferiority not shown	0.423
[TKR] NexGen Hybrid, unconstrained fixed	832	1.60	0.06	[-0.87 , 1.00]	Non-inferiority not shown	0.450
[TKR] NexGen Uncemented, posterior-stabilised fixed	1,082	2.20	0.66	[-0.23 , 1.54]	Non-inferiority not shown	0.073
[TKR] NexGen Uncemented, unconstrained fixed	3,059	3.18	1.63	[0.87 , 2.40]	Inferior by ≥20%	<0.001
[TKR] Optetrak Cement, posterior-stabilised fixed	1,278	4.56	3.02	[1.85 , 4.19]	Inferior by ≥100%	<0.001
[TKR] PFC Sigma Bicondylar Knee Cement, posterior-stabilised mobile	4,255	2.79	1.25	[0.59 , 1.91]	Inferior by ≥20%	<0.001
[TKR] PFC Sigma Bicondylar Knee Cement, constrained condylar	366	2.56	1.02	[-0.32 , 2.36]	Non-inferiority not shown	0.068
[TKR] PFC Sigma Bicondylar Knee Cement, posterior-stabilised fixed	39,518	2.09	0.54	[0.01 , 1.07]	Non-inferiority not shown	0.022
[TKR] PFC Sigma Bicondylar Knee Cement, unconstrained fixed	88,618	1.71	0.16	[-0.36 , 0.68]	Non-inferiority not shown	0.270
[TKR] PFC Sigma Bicondylar Knee Cement, unconstrained mobile	5,415	2.73	1.19	[0.54 , 1.83]	Inferior by ≥20%	<0.001
[TKR] PFC Sigma Bicondylar Knee Hybrid, unconstrained fixed	1,389	1.79	0.25	[-0.59 , 1.08]	Non-inferiority not shown	0.280
[TKR] PFC Sigma Bicondylar Knee Uncemented, unconstrained mobile	450	1.78	0.24	[-0.86 , 1.34]	Non-inferiority not shown	0.335
[TKR] PFC Sigma Bicondylar Knee monobloc polyethylene tibia	2,383	1.65	0.10	[-0.50 , 0.71]	Non-inferiority not shown	0.370
[TKR] Profix Cement, unconstrained fixed	813	3.20	1.65	[0.40 , 2.91]	Inferior by ≥20%	0.005
[TKR] Profix Oxinium monobloc polyethylene tibia	553	3.55	2.01	[0.43 , 3.58]	Inferior by ≥20%	0.006
[TKR] Profix monobloc polyethylene tibia	600	1.35	-0.19	[-1.21 , 0.83]	Non-inferiority not shown	0.356
[TKR] Rotaglide + Cement, unconstrained mobile	1,454	3.65	2.10	[1.06 , 3.15]	Inferior by ≥20%	<0.001
[TKR] Rotaglide Cement, unconstrained mobile	777	3.29	1.75	[0.57 , 2.92]	Inferior by ≥20%	0.002
[TKR] Scorpio Cement, posterior-stabilised mobile	1,178	2.19	0.65	[-0.30 , 1.59]	Non-inferiority not shown	0.089
[TKR] Scorpio Cement, posterior-stabilised fixed	5,265	2.37	0.83	[0.18 , 1.48]	Non-inferiority not shown	0.006
[TKR] Scorpio Cement, unconstrained fixed	9,046	2.61	1.06	[0.46 , 1.66]	Inferior by ≥20%	<0.001
[TKR] Scorpio Cement, unconstrained mobile	1,036	3.63	2.08	[0.88 , 3.29]	Inferior by ≥20%	<0.001
[TKR] Scorpio Hybrid, unconstrained fixed	938	2.51	0.96	[-0.12 , 2.05]	Non-inferiority not shown	0.040
[TKR] Scorpio Uncemented, unconstrained fixed	3,140	2.49	0.95	[0.22 , 1.67]	Non-inferiority not shown	0.005
[TKR] TC Plus Cement, unconstrained fixed	6,952	2.56	1.01	[0.39 , 1.64]	Inferior by ≥20%	0.001

[TKR] TC Plus Cement, unconstrained mobile	3,999	2.00	0.46	[-0.20 , 1.11]	Non-inferiority not shown	0.085
[TKR] TC Plus Uncemented, unconstrained mobile	1,145	2.18	0.63	[-0.25 , 1.52]	Non-inferiority not shown	0.081
[TKR] Triathlon Cement, posterior-stabilised fixed	4,605	2.53	0.98	[0.38 , 1.59]	Inferior by ≥20%	0.001
[TKR] Triathlon Cement, unconstrained fixed	14,360	2.01	0.47	[-0.07 , 1.00]	Non-inferiority not shown	0.044
[TKR] Triathlon Uncemented, unconstrained fixed	418	3.39	1.85	[0.54 , 3.15]	Inferior by ≥20%	0.003
[TKR] Vanguard Cement, posterior-stabilised fixed	1,135	2.75	1.21	[0.45 , 1.97]	Inferior by ≥20%	0.001
[TKR] Vanguard Cement, unconstrained fixed	8,329	2.02	0.48	[-0.07 , 1.02]	Non-inferiority not shown	0.045
[UNI] AMC/Uniglide Unicondylar, fixed	756	4.41	2.87	[1.55 , 4.18]	Inferior by ≥100%	<0.001
[UNI] AMC/Uniglide Unicondylar, mobile	997	10.16	8.62	[6.93 , 10.30]	Inferior by ≥100%	<0.001
[UNI] EIUS Unicondylar, fixed	289	14.92	13.38	[9.60 , 17.16]	Inferior by ≥100%	<0.001
[UNI] Genesis Uni Oxinium Unicondylar, fixed	330	7.05	5.51	[3.07 , 7.94]	Inferior by ≥100%	<0.001
[UNI] MG Uni Unicondylar, fixed	2,082	5.95	4.41	[3.31 , 5.51]	Inferior by ≥100%	<0.001
[UNI] Oxford Partial Knee Unicondylar, fixed	284	4.65	3.10	[1.07 , 5.13]	Inferior by ≥20%	0.001
[UNI] Oxford Partial Knee Unicondylar, mobile	28,136	6.13	4.59	[4.02 , 5.15]	Inferior by ≥100%	<0.001
[UNI] Physica ZUK Unicondylar, fixed	2,392	3.94	2.40	[1.65 , 3.15]	Inferior by ≥100%	<0.001
[UNI] Preservation Unicondylar, fixed	1,045	10.21	8.67	[6.87 , 10.46]	Inferior by ≥100%	<0.001
[UNI] Sigma HP Unicondylar, fixed	1,634	5.01	3.47	[2.61 , 4.32]	Inferior by ≥100%	<0.001
[UNI] Sled Unicondylar, fixed	585	9.83	8.29	[6.09 , 10.48]	Inferior by ≥100%	<0.001

Supplementary table 3: Difference in cumulative percentage revision of knee implants compared to a contemporary benchmark at 7 years post primary for knee replacements with ≥250 procedures remaining at risk

Knee brand, bearing and constraint	Number at risk	Cumulative failure (%)	Difference in failure (%)	95% CI	Equivalence status	p-value
[TKR] Profix Uncemented, unconstrained fixed	1,501	1.77	[REFERENCE]			
[TKR] ACS Uncemented, unconstrained fixed	389	5.01	3.24	[1.16 , 5.31]	Inferior by ≥20%	0.001
[TKR] AGC Cement, posterior-stabilised fixed	355	3.97	2.19	[0.34 , 4.05]	Non-inferiority not shown	0.010
[TKR] AGC Cement, unconstrained fixed	27,288	2.56	0.79	[0.21 , 1.37]	Non-inferiority not shown	0.004
[TKR] AGC Hybrid, unconstrained fixed	541	3.25	1.48	[0.19 , 2.78]	Non-inferiority not shown	0.013
[TKR] AGC Uncemented, unconstrained fixed	710	5.97	4.20	[2.69 , 5.71]	Inferior by ≥100%	<0.001
[TKR] AMK Cement, unconstrained fixed	318	1.02	-0.75	[-1.90 , 0.39]	Non-inferiority not shown	0.098
[TKR] Advance MP Cement, unconstrained fixed	2,588	3.51	1.74	[0.99 , 2.49]	Inferior by ≥20%	<0.001
[TKR] Advance PS Cement, posterior-stabilised fixed	425	4.29	2.52	[0.97 , 4.07]	Inferior by ≥20%	0.001
[TKR] Alpina Hybrid, unconstrained fixed	317	3.84	2.07	[0.13 , 4.01]	Non-inferiority not shown	0.018
[TKR] Columbus Cement, unconstrained fixed	1,482	2.79	1.02	[0.29 , 1.75]	Non-inferiority not shown	0.003
[TKR] E-Motion Bicondylar Knee Uncemented, unconstrained mobile	732	3.86	2.09	[0.89 , 3.29]	Inferior by ≥20%	<0.001
[TKR] FS Cement, unconstrained fixed	643	2.66	0.89	[-0.39 , 2.17]	Non-inferiority not shown	0.087
[TKR] Genesis 2 Cement, posterior-stabilised fixed	2,275	2.96	1.19	[0.50 , 1.88]	Inferior by ≥20%	<0.001
[TKR] Genesis 2 Cement, unconstrained fixed	8,056	2.17	0.39	[-0.20 , 0.99]	Non-inferiority not shown	0.097
[TKR] Genesis 2 Cement, unconstrained mobile	549	2.90	1.12	[-0.14 , 2.38]	Non-inferiority not shown	0.040
[TKR] Genesis 2 Oxinium Cement, posterior-stabilised fixed	500	5.81	4.04	[2.64 , 5.44]	Inferior by ≥100%	<0.001
[TKR] Genesis 2 Oxinium Cement, unconstrained fixed	1,664	3.51	1.74	[0.90 , 2.57]	Inferior by ≥20%	<0.001
[TKR] Innex Uncemented, unconstrained mobile	314	2.45	0.68	[-1.00 , 2.36]	Non-inferiority not shown	0.214
[TKR] Insall-Burstein 2 Cement, posterior-stabilised fixed	1,880	3.43	1.65	[0.71 , 2.60]	Inferior by ≥20%	<0.001
[TKR] Journey Oxinium Cement, posterior-stabilised fixed	334	6.26	4.49	[2.68 , 6.30]	Inferior by ≥100%	<0.001
[TKR] Kinemax Cement, unconstrained fixed	8,560	3.53	1.76	[1.09 , 2.43]	Inferior by ≥20%	<0.001
[TKR] LCS Cement, unconstrained mobile	514	2.61	0.84	[-0.55 , 2.23]	Non-inferiority not shown	0.117
[TKR] LCS Complete Cement, unconstrained mobile	4,216	3.44	1.67	[0.98 , 2.36]	Inferior by ≥20%	<0.001
[TKR] LCS Complete Uncemented, unconstrained mobile	4,724	2.92	1.15	[0.50 , 1.80]	Inferior by ≥20%	<0.001
[TKR] LCS Uncemented, unconstrained mobile	1,092	2.44	0.67	[-0.35 , 1.68]	Non-inferiority not shown	0.100
[TKR] MBK Cement, unconstrained mobile	347	3.18	1.41	[-0.45 , 3.26]	Non-inferiority not shown	0.069
[TKR] MRK Cement, unconstrained fixed	2,775	2.28	0.51	[-0.17 , 1.19]	Non-inferiority not shown	0.072
[TKR] Maxim Cement, posterior-stabilised fixed	353	3.60	1.83	[0.16 , 3.49]	Non-inferiority not shown	0.016

[TKR] Maxim Cement, unconstrained fixed	881	3.05	1.28	[0.14 , 2.42]	Non-inferiority not shown	0.014
[TKR] NRG Cement, posterior-stabilised fixed	1,438	2.86	1.09	[0.32 , 1.86]	Non-inferiority not shown	0.003
[TKR] NRG Cement, unconstrained fixed	2,207	2.83	1.06	[0.33 , 1.78]	Non-inferiority not shown	0.002
[TKR] Natural Knee II Cement, unconstrained fixed	1,437	3.20	1.42	[0.50 , 2.35]	Inferior by ≥20%	0.001
[TKR] Nexgen Cement, PS mobile	551	5.12	3.35	[1.74 , 4.96]	Inferior by ≥20%	<0.001
[TKR] Nexgen Cement, posterior-stabilised fixed	17,857	3.31	1.54	[0.95 , 2.13]	Inferior by ≥20%	<0.001
[TKR] Nexgen Cement, unconstrained fixed	12,329	2.12	0.35	[-0.24 , 0.93]	Non-inferiority not shown	0.122
[TKR] Nexgen Hybrid, unconstrained fixed	574	2.15	0.38	[-0.72 , 1.48]	Non-inferiority not shown	0.250
[TKR] Nexgen Uncemented, ps fixed	784	2.54	0.77	[-0.22 , 1.75]	Non-inferiority not shown	0.064
[TKR] Nexgen Uncemented, unconstrained fixed	2,528	3.58	1.81	[0.98 , 2.64]	Inferior by ≥20%	<0.001
[TKR] Optetrak Cement, posterior-stabilised fixed	809	5.43	3.66	[2.37 , 4.96]	Inferior by ≥100%	<0.001
[TKR] PFC Sigma Bicondylar Knee Cement, PS mobile	2,686	3.36	1.59	[0.85 , 2.32]	Inferior by ≥20%	<0.001
[TKR] PFC Sigma Bicondylar Knee Cement, posterior-stabilised fixed	24,658	2.44	0.67	[0.10 , 1.25]	Non-inferiority not shown	0.011
[TKR] PFC Sigma Bicondylar Knee Cement, unconstrained fixed	55,631	2.00	0.23	[-0.34 , 0.80]	Non-inferiority not shown	0.212
[TKR] PFC Sigma Bicondylar Knee Cement, unconstrained mobile	3,818	3.15	1.38	[0.67 , 2.08]	Inferior by ≥20%	<0.001
[TKR] PFC Sigma Bicondylar Knee Hybrid, unconstrained fixed	1,174	1.86	0.09	[-0.78 , 0.97]	Non-inferiority not shown	0.418
[TKR] PFC Sigma Bicondylar Knee Uncemented, unconstrained mobile	258	2.02	0.25	[-0.96 , 1.46]	Non-inferiority not shown	0.344
[TKR] PFC Sigma Bicondylar Knee monobloc polyethylene tibia	810	1.92	0.15	[-0.55 , 0.85]	Non-inferiority not shown	0.340
[TKR] Profix Cement, unconstrained fixed	693	4.20	2.43	[0.99 , 3.87]	Inferior by ≥20%	<0.001
[TKR] Profix Oxinium monobloc polyethylene tibia	471	4.10	2.32	[0.62 , 4.03]	Inferior by ≥20%	0.004
[TKR] Profix monobloc polyethylene tibia	511	1.52	-0.25	[-1.34 , 0.84]	Non-inferiority not shown	0.325
[TKR] Rotaglide + Cement, unconstrained mobile	1,222	4.22	2.45	[1.32 , 3.59]	Inferior by ≥20%	<0.001
[TKR] Rotaglide Cement, unconstrained mobile	488	3.71	1.94	[0.66 , 3.22]	Inferior by ≥20%	0.002
[TKR] Scorpio Cement, PS mobile	1,013	2.54	0.77	[-0.26 , 1.79]	Non-inferiority not shown	0.071
[TKR] Scorpio Cement, posterior-stabilised fixed	4,675	3.07	1.30	[0.58 , 2.02]	Inferior by ≥20%	<0.001
[TKR] Scorpio Cement, unconstrained fixed	7,331	3.14	1.37	[0.71 , 2.03]	Inferior by ≥20%	<0.001
[TKR] Scorpio Cement, unconstrained mobile	950	4.48	2.71	[1.37 , 4.04]	Inferior by ≥20%	<0.001
[TKR] Scorpio Hybrid, unconstrained fixed	823	3.28	1.50	[0.27 , 2.74]	Non-inferiority not shown	0.009
[TKR] Scorpio Uncemented, unconstrained fixed	2,211	3.11	1.33	[0.52 , 2.14]	Inferior by ≥20%	0.001
[TKR] TC Plus Cement, unconstrained fixed	5,337	2.98	1.20	[0.52 , 1.88]	Inferior by ≥20%	<0.001
[TKR] TC Plus Cement, unconstrained mobile	3,267	2.45	0.68	[-0.05 , 1.40]	Non-inferiority not shown	0.033
[TKR] TC Plus Uncemented, unconstrained mobile	783	2.47	0.70	[-0.27 , 1.67]	Non-inferiority not shown	0.079
[TKR] Triathlon Cement, posterior-stabilised fixed	1,580	2.87	1.10	[0.42 , 1.78]	Inferior by ≥20%	0.001
[TKR] Triathlon Cement, unconstrained fixed	4,819	2.50	0.73	[0.13 , 1.33]	Non-inferiority not shown	0.009
[TKR] Vanguard Cement, posterior-stabilised fixed	360	3.58	1.81	[0.76 , 2.86]	Inferior by ≥20%	<0.001

[TKR] Vanguard Cement, unconstrained fixed	2,491	2.36	0.59	[-0.03 , 1.20]	Non-inferiority not shown	0.031
[UNI] AMC/Uniglide Unicondylar, fixed	401	6.44	4.67	[2.95 , 6.38]	Inferior by ≥100%	<0.001
[UNI] AMC/Uniglide Unicondylar, mobile	687	12.42	10.64	[8.74 , 12.55]	Inferior by ≥100%	<0.001
[UNI] EIUS Unicondylar, fixed	280	16.41	14.64	[10.70 , 18.58]	Inferior by ≥100%	<0.001
[UNI] MG Uni Unicondylar, fixed	1,829	7.55	5.78	[4.55 , 7.01]	Inferior by ≥100%	<0.001
[UNI] Oxford Partial Knee Unicondylar, mobile	18,623	8.09	6.32	[5.69 , 6.95]	Inferior by ≥100%	<0.001
[UNI] Physica ZUK Unicondylar, fixed	1,090	5.31	3.54	[2.59 , 4.48]	Inferior by ≥100%	<0.001
[UNI] Preservation Unicondylar, fixed	940	12.95	11.18	[9.18 , 13.18]	Inferior by ≥100%	<0.001
[UNI] Sled Unicondylar, fixed	476	12.39	10.62	[8.12 , 13.12]	Inferior by ≥100%	<0.001

Supplementary table 4: Difference in cumulative percentage revision of knee implants compared to a contemporary benchmark at 10 years post primary for knee replacements with ≥250 procedures remaining at risk

Knee brand, bearing and constraint	Number at risk	Cumulative failure (%)	Difference in failure (%)	95% CI	Equivalence status	p-value
[TKR] PFC Sigma Bicondylar Knee Cement, unconstrained fixed	19,284	2.37	[REFERENCE]			
[TKR] AGC Cement, unconstrained fixed	9,260	3.44	1.07	[0.85 , 1.29]	Inferior by ≥20%	<0.001
[TKR] AGC Uncemented, unconstrained fixed	253	7.76	5.39	[3.55 , 7.24]	Inferior by ≥100%	<0.001
[TKR] AMK Cement, unconstrained fixed	270	1.35	-1.02	[-2.21 , 0.17]	Non-inferior	0.047
[TKR] Advance MP Cement, unconstrained fixed	722	4.25	1.88	[1.18 , 2.59]	Inferior by ≥20%	<0.001
[TKR] Genesis 2 Cement, posterior-stabilised fixed	324	3.85	1.48	[0.66 , 2.30]	Inferior by ≥20%	<0.001
[TKR] Genesis 2 Cement, unconstrained fixed	1,982	2.60	0.23	[-0.05 , 0.52]	Non-inferiority not shown	0.052
[TKR] Genesis 2 Oxinium Cement, unconstrained fixed	486	4.95	2.58	[1.63 , 3.53]	Inferior by ≥20%	<0.001
[TKR] Insall-Burstein 2 Cement, posterior-stabilised fixed	1,125	4.95	2.58	[1.62 , 3.54]	Inferior by ≥20%	<0.001
[TKR] Kinemax Cement, unconstrained fixed	5,049	4.70	2.33	[1.88 , 2.78]	Inferior by ≥20%	<0.001
[TKR] LCS Cement, unconstrained mobile	442	3.62	1.25	[-0.28 , 2.79]	Non-inferiority not shown	0.055
[TKR] LCS Complete Cement, unconstrained mobile	781	4.28	1.91	[1.35 , 2.47]	Inferior by ≥20%	<0.001
[TKR] LCS Complete Uncemented, unconstrained mobile	1,340	3.31	0.94	[0.55 , 1.34]	Inferior by ≥20%	<0.001
[TKR] LCS Uncemented, unconstrained mobile	970	2.53	0.16	[-0.71 , 1.03]	Non-inferiority not shown	0.358
[TKR] MRK Cement, unconstrained fixed	554	2.91	0.54	[-0.04 , 1.13]	Non-inferiority not shown	0.035
[TKR] Maxim Cement, unconstrained fixed	408	4.32	1.95	[0.67 , 3.24]	Inferior by ≥20%	0.001
[TKR] Natural Knee II Cement, unconstrained fixed	582	3.88	1.51	[0.60 , 2.41]	Inferior by ≥20%	0.001
[TKR] NexGen Cement, posterior-stabilised mobile	268	7.13	4.76	[2.83 , 6.69]	Inferior by ≥100%	<0.001
[TKR] NexGen Cement, posterior-stabilised fixed	5,806	4.45	2.08	[1.79 , 2.37]	Inferior by ≥20%	<0.001
[TKR] NexGen Cement, unconstrained fixed	2,888	2.56	0.19	[-0.06 , 0.45]	Non-inferior	0.069
[TKR] NexGen Hybrid, unconstrained fixed	280	2.15	-0.22	[-1.17 , 0.73]	Non-inferiority not shown	0.326
[TKR] NexGen Uncemented, posterior-stabilised fixed	251	3.12	0.75	[-0.24 , 1.75]	Non-inferiority not shown	0.069
[TKR] NexGen Uncemented, unconstrained fixed	795	3.96	1.59	[0.91 , 2.26]	Inferior by ≥20%	<0.001
[TKR] PFC Sigma Bicondylar Knee Cement, posterior-stabilised mobile	784	4.06	1.69	[1.04 , 2.34]	Inferior by ≥20%	<0.001
[TKR] PFC Sigma Bicondylar Knee Cement, posterior-stabilised fixed	7,944	3.01	0.64	[0.43 , 0.84]	Non-inferiority not shown	<0.001
[TKR] PFC Sigma Bicondylar Knee Cement, unconstrained mobile	886	3.89	1.52	[0.94 , 2.10]	Inferior by ≥20%	<0.001
[TKR] PFC Sigma Bicondylar Knee Hybrid, unconstrained fixed	637	2.09	-0.28	[-1.03 , 0.47]	Non-inferior	0.234
[TKR] Profix Cement, unconstrained fixed	295	5.03	2.66	[1.14 , 4.18]	Inferior by ≥20%	<0.001
[TKR] Profix Uncemented, unconstrained fixed	564	2.21	-0.16	[-0.85 , 0.53]	Non-inferiority not shown	0.329

[TKR] Rotaglide + Cement, unconstrained mobile	696	5.61	3.24	[2.02 , 4.46]	Inferior by ≥20%	<0.001
[TKR] Scorpio Cement, posterior-stabilised mobile	480	3.27	0.90	[-0.14 , 1.94]	Non-inferiority not shown	0.045
[TKR] Scorpio Cement, posterior-stabilised fixed	1,995	3.85	1.48	[0.94 , 2.03]	Inferior by ≥20%	<0.001
[TKR] Scorpio Cement, unconstrained fixed	3,299	3.83	1.46	[1.04 , 1.87]	Inferior by ≥20%	<0.001
[TKR] Scorpio Hybrid, unconstrained fixed	316	4.27	1.90	[0.53 , 3.26]	Inferior by ≥20%	0.003
[TKR] Scorpio Uncemented, unconstrained fixed	517	4.13	1.76	[0.92 , 2.61]	Inferior by ≥20%	<0.001
[TKR] TC Plus Cement, unconstrained fixed	1,294	3.54	1.17	[0.67 , 1.66]	Inferior by ≥20%	<0.001
[TKR] TC Plus Cement, unconstrained mobile	1,493	3.13	0.76	[0.20 , 1.33]	Non-inferiority not shown	0.004
[TKR] Triathlon Cement, unconstrained fixed	334	3.20	0.83	[0.38 , 1.27]	Non-inferiority not shown	<0.001
[UNI] MG Uni Unicondylar, fixed	853	10.03	7.66	[6.33 , 9.00]	Inferior by ≥100%	<0.001
[UNI] Oxford Partial Knee Unicondylar, mobile	6,190	11.54	9.17	[8.73 , 9.62]	Inferior by ≥100%	<0.001
[UNI] Preservation Unicondylar, fixed	398	15.09	12.72	[10.59 , 14.86]	Inferior by ≥100%	<0.001

Supplementary table 5: Difference in cumulative percentage revision of knee implants compared to a contemporary benchmark at 3 years post primary in men for knee replacements with ≥250 procedures remaining at risk

Knee brand, bearing and constraint	Number at risk	Cumulative failure (%)	Difference in failure (%)	95% CI	Equivalence status	p-value
[TKR] NexGen Cement, unconstrained fixed	14,396	1.24	[REFERENCE]			
[TKR] AGC Cement, unconstrained fixed	21,525	1.61	0.36	[0.14 , 0.58]	Non-inferiority not shown	0.001
[TKR] AGC Hybrid, unconstrained fixed	400	2.07	0.83	[-0.52 , 2.17]	Non-inferiority not shown	0.115
[TKR] AGC Uncemented, unconstrained fixed	546	3.92	2.67	[1.10 , 4.25]	Inferior by ≥20%	<0.001
[TKR] Advance MP Cement, unconstrained fixed	2,482	2.39	1.15	[0.58 , 1.71]	Inferior by ≥20%	<0.001
[TKR] Advance PS Cement, posterior-stabilised fixed	342	2.55	1.30	[-0.20 , 2.80]	Non-inferiority not shown	0.045
[TKR] Columbus Cement, unconstrained fixed	2,209	1.78	0.54	[0.05 , 1.03]	Non-inferiority not shown	0.015
[TKR] E-Motion Bicondylar Knee Cement, unconstrained mobile	252	3.17	1.92	[-0.02 , 3.87]	Non-inferiority not shown	0.026
[TKR] E-Motion Bicondylar Knee Uncemented, unconstrained mobile	703	1.65	0.40	[-0.47 , 1.27]	Non-inferiority not shown	0.183
[TKR] FS Cement, unconstrained fixed	325	1.45	0.20	[-1.07 , 1.47]	Non-inferiority not shown	0.376
[TKR] Genesis 2 Cement, posterior-stabilised fixed	3,069	2.07	0.83	[0.37 , 1.29]	Inferior by ≥20%	<0.001
[TKR] Genesis 2 Cement, unconstrained fixed	10,877	1.47	0.22	[-0.03 , 0.47]	Non-inferiority not shown	0.042
[TKR] Genesis 2 Cement, unconstrained mobile	362	2.39	1.14	[-0.41 , 2.69]	Non-inferiority not shown	0.074
[TKR] Genesis 2 Oxinium Cement, posterior-stabilised fixed	700	3.69	2.44	[1.19 , 3.70]	Inferior by ≥20%	<0.001
[TKR] Genesis 2 Oxinium Cement, unconstrained fixed	1,574	2.48	1.23	[0.50 , 1.96]	Inferior by ≥20%	0.001
[TKR] Insall-Burstein 2 Cement, posterior-stabilised fixed	985	1.74	0.50	[-0.32 , 1.31]	Non-inferiority not shown	0.115
[TKR] Journey Oxinium Cement, posterior-stabilised fixed	338	3.13	1.89	[0.06 , 3.72]	Non-inferiority not shown	0.021
[TKR] Kinemax Cement, unconstrained fixed	4,241	1.88	0.64	[0.21 , 1.06]	Non-inferiority not shown	0.002
[TKR] LCS Complete Cement, unconstrained mobile	3,604	1.59	0.35	[-0.07 , 0.76]	Non-inferiority not shown	0.051
[TKR] LCS Complete Uncemented, unconstrained mobile	4,705	1.83	0.59	[0.21 , 0.97]	Non-inferiority not shown	0.001
[TKR] LCS Uncemented, unconstrained mobile	516	2.04	0.80	[-0.41 , 2.01]	Non-inferiority not shown	0.097
[TKR] MRK Cement, unconstrained fixed	2,493	1.52	0.27	[-0.18 , 0.72]	Non-inferiority not shown	0.117
[TKR] Maxim Cement, unconstrained fixed	530	1.09	-0.16	[-1.04 , 0.72]	Non-inferiority not shown	0.364
[TKR] NRG Cement, posterior-stabilised fixed	1,747	1.81	0.57	[-0.05 , 1.18]	Non-inferiority not shown	0.035
[TKR] NRG Cement, unconstrained fixed	2,486	1.83	0.59	[0.08 , 1.10]	Non-inferiority not shown	0.012
[TKR] Natural Knee II Cement, unconstrained fixed	997	1.03	-0.21	[-0.84 , 0.41]	Non-inferiority not shown	0.251
[TKR] NexGen Cement, posterior-stabilised mobile	324	3.61	2.36	[0.50 , 4.23]	Inferior by ≥20%	0.007
[TKR] NexGen Cement, posterior-stabilised fixed	16,394	1.95	0.71	[0.46 , 0.95]	Inferior by ≥20%	<0.001
[TKR] NexGen Hybrid, unconstrained fixed	457	0.81	-0.43	[-1.24 , 0.37]	Non-inferiority not shown	0.147
[TKR] NexGen Uncemented, posterior-stabilised fixed	774	1.35	0.10	[-0.64 , 0.85]	Non-inferiority not shown	0.392
[TKR] NexGen Uncemented, unconstrained fixed	1,965	2.47	1.22	[0.55 , 1.90]	Inferior by ≥20%	<0.001
[TKR] Optetrak Cement, posterior-stabilised fixed	614	2.01	0.77	[-0.32 , 1.87]	Non-inferiority not shown	0.084
[TKR] PFC Sigma Bicondylar Knee Cement, posterior-stabilised mobile	2,652	2.16	0.92	[0.38 , 1.46]	Inferior by ≥20%	<0.001
[TKR] PFC Sigma Bicondylar Knee Cement, constrained condylar	251	1.88	0.63	[-0.87 , 2.13]	Non-inferiority not shown	0.205
[TKR] PFC Sigma Bicondylar Knee Cement, posterior-stabilised fixed	22,261	1.65	0.40	[0.18 , 0.62]	Non-inferiority not shown	<0.001
[TKR] PFC Sigma Bicondylar Knee Cement, unconstrained fixed	53,160	1.39	0.15	[-0.03 , 0.33]	Non-inferiority not shown	0.050
[TKR] PFC Sigma Bicondylar Knee Cement, unconstrained mobile	3,150	2.14	0.89	[0.39 , 1.39]	Inferior by ≥20%	<0.001
[TKR] PFC Sigma Bicondylar Knee Hybrid, unconstrained fixed	700	0.95	-0.29	[-1.01 , 0.43]	Non-inferiority not shown	0.213
[TKR] PFC Sigma Bicondylar Knee Uncemented, unconstrained mobile	280	1.61	0.36	[-0.93 , 1.65]	Non-inferiority not shown	0.292
[TKR] PFC Sigma Bicondylar Knee monobloc polyethylene tibia	2,112	1.44	0.19	[-0.26 , 0.64]	Non-inferiority not shown	0.204

[TKR] Profix Cement, unconstrained fixed	392	2.15	0.90	[-0.49 , 2.30]	Non-inferiority not shown	0.102
[TKR] Profix Uncemented, unconstrained fixed	954	1.00	-0.25	[-0.88 , 0.39]	Non-inferiority not shown	0.225
[TKR] Profix monobloc polyethylene tibia	257	0.36	-0.88	[-1.61 , -0.15]	Non-inferior	0.009
[TKR] Rotaglide + Cement, unconstrained mobile	668	3.36	2.12	[0.79 , 3.45]	Inferior by ≥20%	0.001
[TKR] Rotaglide Cement, unconstrained mobile	403	2.75	1.51	[0.02 , 2.99]	Non-inferiority not shown	0.024
[TKR] Scorpio Cement, posterior-stabilised mobile	565	1.69	0.44	[-0.61 , 1.49]	Non-inferiority not shown	0.204
[TKR] Scorpio Cement, posterior-stabilised fixed	2,265	1.97	0.72	[0.14 , 1.30]	Non-inferiority not shown	0.007
[TKR] Scorpio Cement, unconstrained fixed	4,090	2.25	1.00	[0.53 , 1.47]	Inferior by ≥20%	<0.001
[TKR] Scorpio Cement, unconstrained mobile	464	2.66	1.41	[-0.02 , 2.84]	Non-inferiority not shown	0.027
[TKR] Scorpio Hybrid, unconstrained fixed	405	2.32	1.07	[-0.35 , 2.50]	Non-inferiority not shown	0.070
[TKR] Scorpio Uncemented, unconstrained fixed	1,646	1.75	0.51	[-0.13 , 1.15]	Non-inferiority not shown	0.060
[TKR] TC Plus Cement, unconstrained fixed	3,396	2.32	1.07	[0.56 , 1.59]	Inferior by ≥20%	<0.001
[TKR] TC Plus Cement, unconstrained mobile	1,934	1.98	0.74	[0.12 , 1.35]	Non-inferiority not shown	0.009
[TKR] TC Plus Uncemented, unconstrained mobile	550	1.43	0.18	[-0.71 , 1.08]	Non-inferiority not shown	0.345
[TKR] Triathlon Cement, posterior-stabilised fixed	3,282	1.77	0.52	[0.12 , 0.93]	Non-inferiority not shown	0.006
[TKR] Triathlon Cement, unconstrained fixed	12,371	1.42	0.17	[-0.06 , 0.40]	Non-inferiority not shown	0.073
[TKR] Triathlon Uncemented, unconstrained fixed	332	2.49	1.24	[-0.19 , 2.67]	Non-inferiority not shown	0.044
[TKR] Vanguard Cement, posterior-stabilised fixed	1,226	1.75	0.51	[-0.08 , 1.10]	Non-inferiority not shown	0.046
[TKR] Vanguard Cement, unconstrained fixed	8,120	1.51	0.27	[-0.00 , 0.54]	Non-inferiority not shown	0.025
[UNI] AMC/Uniglide Unicondylar, fixed	501	2.65	1.40	[0.07 , 2.74]	Non-inferiority not shown	0.020
[UNI] AMC/Uniglide Unicondylar, mobile	628	7.99	6.75	[4.78 , 8.71]	Inferior by ≥100%	<0.001
[UNI] MG Uni Unicondylar, fixed	1,203	3.48	2.23	[1.21 , 3.26]	Inferior by ≥20%	<0.001
[UNI] Oxford Partial Knee Unicondylar, mobile	19,491	3.98	2.74	[2.45 , 3.03]	Inferior by ≥100%	<0.001
[UNI] Physica ZUK Unicondylar, fixed	2,555	2.33	1.08	[0.57 , 1.60]	Inferior by ≥20%	<0.001
[UNI] Preservation Unicondylar, fixed	600	5.68	4.44	[2.65 , 6.22]	Inferior by ≥100%	<0.001
[UNI] Sigma HP Unicondylar, fixed	2,062	3.12	1.87	[1.24 , 2.51]	Inferior by ≥20%	<0.001
[UNI] Sled Unicondylar, fixed	308	6.69	5.45	[2.85 , 8.04]	Inferior by ≥100%	<0.001

Supplementary table 6: Difference in cumulative percentage revision of knee implants compared to a contemporary benchmark at 7 years post primary in men for knee replacements with ≥250 procedures remaining at risk

Knee brand, bearing and constraint	Number at risk	Cumulative failure (%)	Difference in failure (%)	95% CI	Equivalence status	p-value
[TKR] PFC Sigma Bicondylar Knee Cement, unconstrained fixed	22,730	2.16	[REFERENCE]			
[TKR] AGC Cement, unconstrained fixed	11,118	2.94	0.78	[0.51 , 1.04]	Inferior by ≥20%	<0.001
[TKR] AGC Hybrid, unconstrained fixed	250	3.18	1.02	[-0.70 , 2.73]	Non-inferiority not shown	0.123
[TKR] AGC Uncemented, unconstrained fixed	324	5.56	3.40	[1.48 , 5.33]	Inferior by ≥20%	<0.001
[TKR] Advance MP Cement, unconstrained fixed	1,052	3.91	1.75	[0.96 , 2.53]	Inferior by ≥20%	<0.001
[TKR] Columbus Cement, unconstrained fixed	625	2.70	0.53	[-0.15 , 1.22]	Non-inferiority not shown	0.064
[TKR] E-Motion Bicondylar Knee Uncemented, unconstrained mobile	352	3.20	1.04	[-0.34 , 2.43]	Non-inferiority not shown	0.070
[TKR] FS Cement, unconstrained fixed	276	2.10	-0.06	[-1.61 , 1.48]	Non-inferiority not shown	0.468
[TKR] Genesis 2 Cement, posterior-stabilised fixed	848	3.70	1.54	[0.80 , 2.28]	Inferior by ≥20%	<0.001
[TKR] Genesis 2 Cement, unconstrained fixed	3,359	2.69	0.53	[0.18 , 0.88]	Non-inferiority not shown	0.002
[TKR] Genesis 2 Oxinium Cement, unconstrained fixed	758	3.98	1.82	[0.83 , 2.81]	Inferior by ≥20%	<0.001
[TKR] Insall-Burstein 2 Cement, posterior-stabilised fixed	797	3.57	1.40	[0.23 , 2.57]	Non-inferiority not shown	0.009
[TKR] Kinemax Cement, unconstrained fixed	3,602	3.87	1.71	[1.12 , 2.30]	Inferior by ≥20%	<0.001
[TKR] LCS Complete Cement, unconstrained mobile	1,775	3.47	1.31	[0.68 , 1.94]	Inferior by ≥20%	<0.001
[TKR] LCS Complete Uncemented, unconstrained mobile	2,105	2.92	0.76	[0.25 , 1.27]	Non-inferiority not shown	0.002
[TKR] LCS Uncemented, unconstrained mobile	457	2.82	0.66	[-0.75 , 2.07]	Non-inferiority not shown	0.181
[TKR] MRK Cement, unconstrained fixed	1,056	2.97	0.80	[0.08 , 1.53]	Non-inferiority not shown	0.015
[TKR] Maxim Cement, unconstrained fixed	380	2.84	0.68	[-0.81 , 2.16]	Non-inferiority not shown	0.186
[TKR] NRG Cement, posterior-stabilised fixed	580	3.14	0.98	[0.13 , 1.83]	Non-inferiority not shown	0.012
[TKR] NRG Cement, unconstrained fixed	908	3.76	1.60	[0.77 , 2.43]	Inferior by ≥20%	<0.001
[TKR] Natural Knee II Cement, unconstrained fixed	559	3.46	1.30	[0.05 , 2.55]	Non-inferiority not shown	0.021
[TKR] NexGen Cement, posterior-stabilised fixed	7,174	3.81	1.65	[1.32 , 1.99]	Inferior by ≥20%	<0.001
[TKR] NexGen Cement, unconstrained fixed	4,862	2.32	0.16	[-0.14 , 0.45]	Non-inferiority not shown	0.151
[TKR] NexGen Uncemented, posterior-stabilised fixed	419	2.04	-0.12	[-1.13 , 0.89]	Non-inferiority not shown	0.410
[TKR] NexGen Uncemented, unconstrained fixed	1,270	3.52	1.35	[0.53 , 2.17]	Inferior by ≥20%	0.001
[TKR] Optetrak Cement, posterior-stabilised fixed	335	4.79	2.63	[0.87 , 4.38]	Inferior by ≥20%	0.002
[TKR] PFC Sigma Bicondylar Knee Cement, posterior-stabilised mobile	1,226	3.23	1.07	[0.37 , 1.77]	Non-inferiority not shown	0.001
[TKR] PFC Sigma Bicondylar Knee Cement, posterior-stabilised fixed	9,690	2.53	0.37	[0.12 , 0.61]	Non-inferiority not shown	0.002
[TKR] PFC Sigma Bicondylar Knee Cement, unconstrained mobile	1,807	3.24	1.08	[0.44 , 1.71]	Inferior by ≥20%	<0.001
[TKR] PFC Sigma Bicondylar Knee Hybrid, unconstrained fixed	527	1.86	-0.31	[-1.31 , 0.70]	Non-inferiority not shown	0.277
[TKR] PFC Sigma Bicondylar Knee monobloc polyethylene tibia	287	2.16	0.00	[-0.75 , 0.75]	Non-inferiority not shown	0.498
[TKR] Profix Cement, unconstrained fixed	306	5.09	2.93	[0.74 , 5.11]	Inferior by ≥20%	0.004
[TKR] Profix Uncemented, unconstrained fixed	676	1.73	-0.44	[-1.29 , 0.42]	Non-inferior	0.158
[TKR] Rotaglide + Cement, unconstrained mobile	509	5.27	3.10	[1.42 , 4.79]	Inferior by ≥20%	<0.001
[TKR] Scorpio Cement, posterior-stabilised mobile	437	2.60	0.44	[-0.87 , 1.74]	Non-inferiority not shown	0.256
[TKR] Scorpio Cement, posterior-stabilised fixed	1,835	3.88	1.72	[0.91 , 2.53]	Inferior by ≥20%	<0.001
[TKR] Scorpio Cement, unconstrained fixed	2,978	3.76	1.60	[1.00 , 2.20]	Inferior by ≥20%	<0.001
[TKR] Scorpio Cement, unconstrained mobile	392	4.62	2.46	[0.57 , 4.35]	Inferior by ≥20%	0.005
[TKR] Scorpio Hybrid, unconstrained fixed	326	4.13	1.97	[0.04 , 3.91]	Non-inferiority not shown	0.023
[TKR] Scorpio Uncemented, unconstrained fixed	993	3.15	0.99	[0.11 , 1.87]	Non-inferiority not shown	0.014

[TKR] TC Plus Cement, unconstrained fixed	2,380	3.40	1.24	[0.62 , 1.86]	Inferior by ≥20%	<0.001
[TKR] TC Plus Cement, unconstrained mobile	1,419	3.20	1.04	[0.25 , 1.82]	Non-inferiority not shown	0.005
[TKR] TC Plus Uncemented, unconstrained mobile	281	2.32	0.16	[-1.10 , 1.42]	Non-inferiority not shown	0.400
[TKR] Triathlon Cement, posterior-stabilised fixed	614	2.75	0.59	[0.03 , 1.15]	Non-inferiority not shown	0.019
[TKR] Triathlon Cement, unconstrained fixed	1,973	2.52	0.36	[-0.00 , 0.72]	Non-inferiority not shown	0.026
[TKR] Vanguard Cement, unconstrained fixed	1,017	2.24	0.08	[-0.27 , 0.42]	Non-inferior	0.336
[UNI] AMC/Uniglide Unicondylar, mobile	350	10.65	8.49	[6.16 , 10.82]	Inferior by ≥100%	<0.001
[UNI] MG Uni Unicondylar, fixed	990	6.59	4.43	[3.04 , 5.83]	Inferior by ≥100%	<0.001
[UNI] Oxford Partial Knee Unicondylar, mobile	9,524	7.45	5.29	[4.89 , 5.69]	Inferior by ≥100%	<0.001
[UNI] Physica ZUK Unicondylar, fixed	594	4.64	2.48	[1.57 , 3.40]	Inferior by ≥20%	<0.001
[UNI] Preservation Unicondylar, fixed	505	11.21	9.05	[6.58 , 11.51]	Inferior by ≥100%	<0.001

Supplementary table 7: Difference in cumulative percentage revision of knee implants compared to a contemporary benchmark at 10 years post primary in men for knee replacements with ≥250 procedures remaining at risk

Knee brand, bearing and constraint	Number at risk	Cumulative failure (%)	Difference in failure (%)	95% CI	Equivalence status	p-value
[TKR] PFC Sigma Bicondylar Knee Cement, unconstrained fixed	7,760	2.55	[REFERENCE]			
[TKR] AGC Cement, unconstrained fixed	3,646	4.12	1.57	[1.18 , 1.95]	Inferior by ≥20%	<0.001
[TKR] Advance MP Cement, unconstrained fixed	279	4.50	1.94	[0.85 , 3.03]	Inferior by ≥20%	<0.001
[TKR] Genesis 2 Cement, unconstrained fixed	807	3.26	0.70	[0.21 , 1.19]	Non-inferiority not shown	0.003
[TKR] Insall-Burstein 2 Cement, posterior-stabilised fixed	457	5.91	3.35	[1.73 , 4.98]	Inferior by ≥20%	<0.001
[TKR] Kinemax Cement, unconstrained fixed	2,113	5.00	2.45	[1.75 , 3.15]	Inferior by ≥20%	<0.001
[TKR] LCS Complete Cement, unconstrained mobile	303	4.25	1.70	[0.91 , 2.49]	Inferior by ≥20%	<0.001
[TKR] LCS Complete Uncemented, unconstrained mobile	602	3.33	0.78	[0.18 , 1.38]	Non-inferiority not shown	0.006
[TKR] LCS Uncemented, unconstrained mobile	406	3.04	0.49	[-0.99 , 1.97]	Non-inferiority not shown	0.258
[TKR] NexGen Cement, posterior-stabilised fixed	2,308	5.14	2.58	[2.10 , 3.07]	Inferior by ≥20%	<0.001
[TKR] NexGen Cement, unconstrained fixed	1,167	2.71	0.16	[-0.24 , 0.55]	Non-inferiority not shown	0.220
[TKR] NexGen Uncemented, unconstrained fixed	348	3.61	1.05	[0.21 , 1.90]	Non-inferiority not shown	0.007
[TKR] PFC Sigma Bicondylar Knee Cement, posterior-stabilised mobile	340	4.31	1.75	[0.71 , 2.80]	Inferior by ≥20%	0.001
[TKR] PFC Sigma Bicondylar Knee Cement, posterior-stabilised fixed	3,119	3.16	0.61	[0.28 , 0.94]	Non-inferiority not shown	<0.001
[TKR] PFC Sigma Bicondylar Knee Cement, unconstrained mobile	419	3.97	1.41	[0.55 , 2.27]	Inferior by ≥20%	0.001
[TKR] PFC Sigma Bicondylar Knee Hybrid, unconstrained fixed	276	2.14	-0.41	[-1.57 , 0.74]	Non-inferiority not shown	0.241
[TKR] Profix Uncemented, unconstrained fixed	278	2.35	-0.20	[-1.32 , 0.91]	Non-inferiority not shown	0.361
[TKR] Rotaglide + Cement, unconstrained mobile	289	6.95	4.40	[2.31 , 6.48]	Inferior by ≥20%	<0.001
[TKR] Scorpio Cement, posterior-stabilised fixed	794	4.65	2.10	[1.16 , 3.03]	Inferior by ≥20%	<0.001
[TKR] Scorpio Cement, unconstrained fixed	1,325	4.43	1.87	[1.19 , 2.56]	Inferior by ≥20%	<0.001
[TKR] TC Plus Cement, unconstrained fixed	565	4.15	1.60	[0.79 , 2.41]	Inferior by ≥20%	<0.001
[TKR] TC Plus Cement, unconstrained mobile	668	4.06	1.50	[0.55 , 2.45]	Inferior by ≥20%	0.001
[UNI] MG Uni Unicondylar, fixed	445	8.58	6.02	[4.34 , 7.70]	Inferior by ≥100%	<0.001
[UNI] Oxford Partial Knee Unicondylar, mobile	3,124	10.63	8.08	[7.48 , 8.67]	Inferior by ≥100%	<0.001

Supplementary table 8: Difference in cumulative percentage revision of knee implants compared to a contemporary benchmark at 3 years post primary in women for knee replacements with ≥250 procedures remaining at risk

Knee brand, bearing and constraint	Number at risk	Cumulative failure (%)	Difference in failure (%)	95% CI	Equivalence status	p-value
[TKR] MRK Cement, unconstrained fixed	3,684	0.97	[REFERENCE]			
[TKR] AGC Cement, posterior-stabilised fixed	292	2.31	1.33	[-0.38 , 3.05]	Non-inferiority not shown	0.063
[TKR] AGC Cement, unconstrained fixed	29,830	1.30	0.33	[0.02 , 0.63]	Non-inferiority not shown	0.017
[TKR] AGC Hybrid, unconstrained fixed	462	2.46	1.49	[0.09 , 2.90]	Non-inferiority not shown	0.019
[TKR] AGC Uncemented, unconstrained fixed	536	4.24	3.27	[1.59 , 4.95]	Inferior by ≥100%	<0.001
[TKR] Advance MP Cement, unconstrained fixed	3,068	1.74	0.77	[0.26 , 1.27]	Inferior by ≥20%	0.002
[TKR] Advance MP Stature Cement, unconstrained fixed	693	1.84	0.87	[-0.04 , 1.78]	Non-inferiority not shown	0.031
[TKR] Advance PS Cement, posterior-stabilised fixed	449	2.27	1.29	[-0.01 , 2.60]	Non-inferiority not shown	0.026
[TKR] Alpina Hybrid, unconstrained fixed	278	1.74	0.77	[-0.77 , 2.31]	Non-inferiority not shown	0.164
[TKR] Columbus Cement, unconstrained fixed	2,921	1.78	0.81	[0.32 , 1.30]	Inferior by ≥20%	0.001
[TKR] E-Motion Bicondylar Knee Cement, unconstrained mobile	451	2.94	1.96	[0.55 , 3.38]	Inferior by ≥20%	0.003
[TKR] E-Motion Bicondylar Knee Uncemented, unconstrained mobile	692	2.42	1.45	[0.36 , 2.53]	Inferior by ≥20%	0.004
[TKR] FS Cement, unconstrained fixed	417	1.62	0.65	[-0.58 , 1.87]	Non-inferiority not shown	0.150
[TKR] Genesis 2 Cement, posterior-stabilised fixed	4,795	1.53	0.56	[0.16 , 0.96]	Non-inferiority not shown	0.003
[TKR] Genesis 2 Cement, unconstrained fixed	14,679	1.12	0.15	[-0.16 , 0.46]	Non-inferiority not shown	0.174
[TKR] Genesis 2 Cement, unconstrained mobile	526	1.29	0.32	[-0.67 , 1.31]	Non-inferiority not shown	0.263
[TKR] Genesis 2 Oxinium Cement, posterior-stabilised fixed	981	2.41	1.44	[0.55 , 2.32]	Inferior by ≥20%	0.001
[TKR] Genesis 2 Oxinium Cement, unconstrained fixed	2,060	1.55	0.57	[0.01 , 1.14]	Non-inferiority not shown	0.023
[TKR] Insall-Burstein 2 Cement, posterior-stabilised fixed	1,242	1.25	0.28	[-0.39 , 0.95]	Non-inferiority not shown	0.205
[TKR] Journey Oxinium Cement, posterior-stabilised fixed	407	5.77	4.80	[2.59 , 7.01]	Inferior by ≥100%	<0.001
[TKR] Kinemax Cement, unconstrained fixed	5,695	1.71	0.73	[0.30 , 1.16]	Inferior by ≥20%	<0.001
[TKR] LCS Cement, unconstrained mobile	363	1.58	0.60	[-0.68 , 1.89]	Non-inferiority not shown	0.178
[TKR] LCS Complete Cement, unconstrained mobile	4,847	1.65	0.68	[0.24 , 1.11]	Inferior by ≥20%	0.001
[TKR] LCS Complete Uncemented, unconstrained mobile	5,591	1.68	0.71	[0.29 , 1.13]	Inferior by ≥20%	<0.001
[TKR] LCS Uncemented, unconstrained mobile	722	1.74	0.77	[-0.21 , 1.75]	Non-inferiority not shown	0.062
[TKR] Maxim Cement, posterior-stabilised fixed	340	1.69	0.72	[-0.65 , 2.09]	Non-inferiority not shown	0.153
[TKR] Maxim Cement, unconstrained fixed	715	1.76	0.79	[-0.20 , 1.78]	Non-inferiority not shown	0.059
[TKR] NRG Cement, posterior-stabilised fixed	2,394	1.63	0.66	[0.10 , 1.22]	Non-inferiority not shown	0.011
[TKR] NRG Cement, unconstrained fixed	3,377	1.22	0.24	[-0.20 , 0.68]	Non-inferiority not shown	0.140
[TKR] Natural Knee II Cement, unconstrained fixed	1,485	1.60	0.63	[-0.05 , 1.31]	Non-inferiority not shown	0.035
[TKR] NexGen Cement, posterior-stabilised mobile	490	2.31	1.33	[0.10 , 2.57]	Non-inferiority not shown	0.017
[TKR] NexGen Cement, posterior-stabilised fixed	24,309	1.38	0.40	[0.10 , 0.71]	Non-inferiority not shown	0.005
[TKR] NexGen Cement, unconstrained fixed	20,162	0.99	0.02	[-0.28 , 0.32]	Non-inferiority not shown	0.452
[TKR] NexGen Hybrid, unconstrained fixed	507	1.30	0.33	[-0.67 , 1.33]	Non-inferiority not shown	0.259
[TKR] NexGen Uncemented, posterior-stabilised fixed	650	2.15	1.18	[0.12 , 2.23]	Non-inferiority not shown	0.014
[TKR] NexGen Uncemented, unconstrained fixed	1,577	2.66	1.69	[0.87 , 2.51]	Inferior by ≥20%	<0.001
[TKR] NexGen monobloc polyethylene tibia	314	0.46	-0.51	[-1.22 , 0.20]	Non-inferiority not shown	0.080
[TKR] Optetrak Cement, posterior-stabilised fixed	882	3.11	2.13	[0.99 , 3.28]	Inferior by ≥100%	<0.001
[TKR] PFC Sigma Bicondylar Knee Cement, posterior-stabilised mobile	3,081	2.08	1.10	[0.56 , 1.65]	Inferior by ≥20%	<0.001
[TKR] PFC Sigma Bicondylar Knee Cement, constrained condylar	310	1.62	0.64	[-0.58 , 1.87]	Non-inferiority not shown	0.152

[TKR] PFC Sigma Bicondylar Knee Cement, posterior-stabilised fixed	32,691	1.42	0.45	[0.15 , 0.75]	Non-inferiority not shown	0.002
[TKR] PFC Sigma Bicondylar Knee Cement, unconstrained fixed	72,974	1.13	0.16	[-0.12 , 0.45]	Non-inferiority not shown	0.134
[TKR] PFC Sigma Bicondylar Knee Cement, unconstrained mobile	3,424	1.75	0.78	[0.28 , 1.28]	Inferior by ≥20%	0.001
[TKR] PFC Sigma Bicondylar Knee Hybrid, unconstrained fixed	833	1.38	0.40	[-0.42 , 1.23]	Non-inferiority not shown	0.167
[TKR] PFC Sigma Bicondylar Knee Uncemented, unconstrained mobile	324	1.28	0.31	[-0.85 , 1.47]	Non-inferiority not shown	0.299
[TKR] PFC Sigma Bicondylar Knee monobloc polyethylene tibia	3,257	1.22	0.24	[-0.17 , 0.66]	Non-inferiority not shown	0.126
[TKR] Profix Cement, unconstrained fixed	492	1.96	0.99	[-0.25 , 2.22]	Non-inferiority not shown	0.059
[TKR] Profix Oxinium monobloc polyethylene tibia	327	2.68	1.71	[-0.04 , 3.45]	Non-inferiority not shown	0.028
[TKR] Profix Uncemented, unconstrained fixed	1,184	1.46	0.49	[-0.24 , 1.22]	Non-inferiority not shown	0.093
[TKR] Profix monobloc polyethylene tibia	395	0.96	-0.01	[-0.99 , 0.97]	Non-inferiority not shown	0.493
[TKR] Rotaglide + Cement, unconstrained mobile	909	2.42	1.45	[0.43 , 2.46]	Inferior by ≥20%	0.003
[TKR] Rotaglide Cement, unconstrained mobile	649	1.62	0.64	[-0.31 , 1.60]	Non-inferiority not shown	0.092
[TKR] Scorpio Cement, posterior-stabilised mobile	725	1.33	0.36	[-0.51 , 1.23]	Non-inferiority not shown	0.208
[TKR] Scorpio Cement, posterior-stabilised fixed	3,409	1.33	0.36	[-0.11 , 0.82]	Non-inferiority not shown	0.068
[TKR] Scorpio Cement, unconstrained fixed	5,993	1.58	0.60	[0.19 , 1.02]	Non-inferiority not shown	0.002
[TKR] Scorpio Cement, unconstrained mobile	631	2.45	1.48	[0.26 , 2.69]	Inferior by ≥20%	0.009
[TKR] Scorpio Hybrid, unconstrained fixed	601	1.44	0.46	[-0.51 , 1.43]	Non-inferiority not shown	0.176
[TKR] Scorpio Uncemented, unconstrained fixed	1,885	1.83	0.86	[0.20 , 1.51]	Inferior by ≥20%	0.005
[TKR] TC Plus Cement, unconstrained fixed	4,106	1.53	0.55	[0.09 , 1.01]	Non-inferiority not shown	0.009
[TKR] TC Plus Cement, unconstrained mobile	2,452	1.02	0.04	[-0.43 , 0.51]	Non-inferiority not shown	0.428
[TKR] TC Plus Uncemented, unconstrained mobile	890	1.56	0.59	[-0.22 , 1.40]	Non-inferiority not shown	0.077
[TKR] Triathlon Cement, posterior-stabilised fixed	4,914	1.70	0.72	[0.31 , 1.14]	Inferior by ≥20%	<0.001
[TKR] Triathlon Cement, unconstrained fixed	16,831	1.53	0.56	[0.24 , 0.87]	Inferior by ≥20%	<0.001
[TKR] Triathlon Uncemented, unconstrained fixed	411	2.97	1.99	[0.51 , 3.47]	Inferior by ≥20%	0.004
[TKR] Vanguard Cement, constrained condylar	260	0.39	-0.59	[-1.11 , -0.06]	Non-inferior	0.014
[TKR] Vanguard Cement, posterior-stabilised fixed	1,764	2.01	1.04	[0.45 , 1.63]	Inferior by ≥20%	<0.001
[TKR] Vanguard Cement, unconstrained fixed	11,272	1.27	0.30	[-0.03 , 0.62]	Non-inferiority not shown	0.037
[UNI] AMC/Uniglide Unicondylar, fixed	571	3.27	2.30	[0.89 , 3.70]	Inferior by ≥20%	0.001
[UNI] AMC/Uniglide Unicondylar, mobile	568	9.00	8.03	[5.82 , 10.24]	Inferior by ≥100%	<0.001
[UNI] MG Uni Unicondylar, fixed	1,004	4.54	3.57	[2.28 , 4.85]	Inferior by ≥100%	<0.001
[UNI] Oxford Partial Knee Unicondylar, mobile	17,891	4.18	3.20	[2.82 , 3.59]	Inferior by ≥100%	<0.001
[UNI] Physica ZUK Unicondylar, fixed	2,153	2.59	1.61	[0.98 , 2.25]	Inferior by ≥100%	<0.001
[UNI] Preservation Unicondylar, fixed	506	8.29	7.32	[5.01 , 9.63]	Inferior by ≥100%	<0.001
[UNI] Sigma HP Unicondylar, fixed	1,499	4.15	3.17	[2.30 , 4.05]	Inferior by ≥100%	<0.001
[UNI] Sled Unicondylar, fixed	375	5.85	4.87	[2.63 , 7.12]	Inferior by ≥100%	<0.001

Supplementary table 9: Difference in cumulative percentage revision of knee implants compared to a contemporary benchmark at 7 years post primary in women for knee replacements with ≥250 procedures remaining at risk

Knee brand, bearing and constraint	Number at risk	Cumulative failure (%)	Difference in failure (%)	95% CI	Equivalence status	p-value
[TKR] Genesis 2 Cement, unconstrained fixed	4,697	1.77	[REFERENCE]			
[TKR] AGC Cement, unconstrained fixed	16,170	2.29	0.52	[0.23 , 0.80]	Non-inferiority not shown	<0.001
[TKR] AGC Hybrid, unconstrained fixed	292	3.33	1.56	[-0.06 , 3.18]	Non-inferiority not shown	0.030
[TKR] AGC Uncemented, unconstrained fixed	387	6.37	4.59	[2.54 , 6.65]	Inferior by ≥100%	<0.001
[TKR] Advance MP Cement, unconstrained fixed	1,537	3.17	1.40	[0.72 , 2.08]	Inferior by ≥20%	<0.001
[TKR] Columbus Cement, unconstrained fixed	858	2.87	1.10	[0.42 , 1.77]	Inferior by ≥20%	0.001
[TKR] E-Motion Bicondylar Knee Uncemented, unconstrained mobile	381	4.50	2.73	[1.11 , 4.35]	Inferior by ≥20%	<0.001
[TKR] FS Cement, unconstrained fixed	368	3.10	1.32	[-0.35 , 3.00]	Non-inferiority not shown	0.061
[TKR] Genesis 2 Cement, posterior-stabilised fixed	1,428	2.50	0.73	[0.22 , 1.24]	Non-inferiority not shown	0.003
[TKR] Genesis 2 Cement, unconstrained mobile	328	1.97	0.20	[-1.04 , 1.44]	Non-inferiority not shown	0.376
[TKR] Genesis 2 Oxinium Cement, posterior-stabilised fixed	324	5.72	3.94	[2.18 , 5.70]	Inferior by ≥100%	<0.001
[TKR] Genesis 2 Oxinium Cement, unconstrained fixed	906	3.15	1.38	[0.57 , 2.19]	Inferior by ≥20%	<0.001
[TKR] Insall-Burstein 2 Cement, posterior-stabilised fixed	1,086	3.31	1.54	[0.51 , 2.56]	Inferior by ≥20%	0.002
[TKR] Kinemax Cement, unconstrained fixed	4,958	3.28	1.51	[0.99 , 2.02]	Inferior by ≥20%	<0.001
[TKR] LCS Cement, unconstrained mobile	312	2.47	0.70	[-0.92 , 2.31]	Non-inferiority not shown	0.199
[TKR] LCS Complete Cement, unconstrained mobile	2,442	3.42	1.64	[1.06 , 2.22]	Inferior by ≥20%	<0.001
[TKR] LCS Complete Uncemented, unconstrained mobile	2,619	2.93	1.15	[0.65 , 1.65]	Inferior by ≥20%	<0.001
[TKR] LCS Uncemented, unconstrained mobile	636	2.16	0.39	[-0.68 , 1.46]	Non-inferiority not shown	0.238
[TKR] MRK Cement, unconstrained fixed	1,720	1.83	0.05	[-0.45 , 0.56]	Non-inferiority not shown	0.416
[TKR] Maxim Cement, unconstrained fixed	502	3.22	1.44	[0.09 , 2.80]	Non-inferiority not shown	0.018
[TKR] NRG Cement, posterior-stabilised fixed	859	2.66	0.89	[0.17 , 1.60]	Non-inferiority not shown	0.008
[TKR] NRG Cement, unconstrained fixed	1,301	2.15	0.38	[-0.18 , 0.94]	Non-inferiority not shown	0.092
[TKR] Natural Knee II Cement, unconstrained fixed	879	3.04	1.26	[0.32 , 2.21]	Non-inferiority not shown	0.005
[TKR] NexGen Cement, posterior-stabilised mobile	337	5.09	3.32	[1.36 , 5.28]	Inferior by ≥20%	<0.001
[TKR] NexGen Cement, posterior-stabilised fixed	10,683	2.97	1.20	[0.88 , 1.52]	Inferior by ≥20%	<0.001
[TKR] NexGen Cement, unconstrained fixed	7,469	1.98	0.21	[-0.10 , 0.51]	Non-inferiority not shown	0.095
[TKR] NexGen Hybrid, unconstrained fixed	333	2.18	0.40	[-0.89 , 1.70]	Non-inferiority not shown	0.271
[TKR] NexGen Uncemented, posterior-stabilised fixed	366	3.11	1.34	[0.00 , 2.67]	Non-inferiority not shown	0.025
[TKR] NexGen Uncemented, unconstrained fixed	1,261	3.67	1.90	[0.96 , 2.84]	Inferior by ≥20%	<0.001
[TKR] Optetrak Cement, posterior-stabilised fixed	474	5.87	4.10	[2.52 , 5.67]	Inferior by ≥100%	<0.001
[TKR] PFC Sigma Bicondylar Knee Cement, posterior-stabilised mobile	1,460	3.46	1.69	[0.99 , 2.39]	Inferior by ≥20%	<0.001
[TKR] PFC Sigma Bicondylar Knee Cement, posterior-stabilised fixed	14,968	2.39	0.61	[0.33 , 0.90]	Non-inferiority not shown	<0.001
[TKR] PFC Sigma Bicondylar Knee Cement, unconstrained fixed	32,901	1.89	0.11	[-0.13 , 0.36]	Non-inferiority not shown	0.183
[TKR] PFC Sigma Bicondylar Knee Cement, unconstrained mobile	2,012	3.07	1.30	[0.68 , 1.92]	Inferior by ≥20%	<0.001
[TKR] PFC Sigma Bicondylar Knee Hybrid, unconstrained fixed	648	1.88	0.10	[-0.84 , 1.04]	Non-inferiority not shown	0.414
[TKR] PFC Sigma Bicondylar Knee monobloc polyethylene tibia	523	1.78	0.00	[-0.56 , 0.57]	Non-inferiority not shown	0.496
[TKR] Profix Cement, unconstrained fixed	388	3.48	1.71	[0.06 , 3.36]	Non-inferiority not shown	0.021
[TKR] Profix Oxinium monobloc polyethylene tibia	276	3.90	2.13	[0.04 , 4.22]	Non-inferiority not shown	0.023
[TKR] Profix Uncemented, unconstrained fixed	827	1.81	0.04	[-0.75 , 0.82]	Non-inferiority not shown	0.461
[TKR] Profix monobloc polyethylene tibia	323	1.47	-0.30	[-1.49 , 0.89]	Non-inferiority not shown	0.309

[TKR] Rotaglide + Cement, unconstrained mobile	715	3.45	1.68	[0.48 , 2.88]	Inferior by ≥20%	0.003
[TKR] Rotaglide Cement, unconstrained mobile	318	3.32	1.55	[0.10 , 3.00]	Non-inferiority not shown	0.018
[TKR] Scorpio Cement, posterior-stabilised mobile	577	2.50	0.72	[-0.44 , 1.89]	Non-inferiority not shown	0.112
[TKR] Scorpio Cement, posterior-stabilised fixed	2,841	2.53	0.76	[0.18 , 1.33]	Non-inferiority not shown	0.005
[TKR] Scorpio Cement, unconstrained fixed	4,356	2.72	0.95	[0.47 , 1.42]	Inferior by ≥20%	<0.001
[TKR] Scorpio Cement, unconstrained mobile	559	4.37	2.60	[1.00 , 4.20]	Inferior by ≥20%	0.001
[TKR] Scorpio Hybrid, unconstrained fixed	499	2.69	0.92	[-0.41 , 2.24]	Non-inferiority not shown	0.087
[TKR] Scorpio Uncemented, unconstrained fixed	1,219	3.07	1.29	[0.47 , 2.11]	Inferior by ≥20%	0.001
[TKR] TC Plus Cement, unconstrained fixed	2,962	2.62	0.85	[0.31 , 1.38]	Non-inferiority not shown	0.001
[TKR] TC Plus Cement, unconstrained mobile	1,850	1.85	0.08	[-0.50 , 0.66]	Non-inferiority not shown	0.394
[TKR] TC Plus Uncemented, unconstrained mobile	502	2.56	0.79	[-0.26 , 1.84]	Non-inferiority not shown	0.070
[TKR] Triathlon Cement, posterior-stabilised fixed	966	2.94	1.17	[0.60 , 1.74]	Inferior by ≥20%	<0.001
[TKR] Triathlon Cement, unconstrained fixed	2,850	2.49	0.72	[0.37 , 1.06]	Inferior by ≥20%	<0.001
[TKR] Vanguard Cement, unconstrained fixed	1,475	2.44	0.67	[0.25 , 1.10]	Non-inferiority not shown	0.001
[UNI] AMC/Uniglide Unicondylar, mobile	338	14.31	12.54	[9.70 , 15.38]	Inferior by ≥100%	<0.001
[UNI] MG Uni Unicondylar, fixed	840	8.68	6.91	[5.18 , 8.64]	Inferior by ≥100%	<0.001
[UNI] Oxford Partial Knee Unicondylar, mobile	9,099	8.78	7.00	[6.52 , 7.49]	Inferior by ≥100%	<0.001
[UNI] Physica ZUK Unicondylar, fixed	497	6.06	4.29	[3.03 , 5.55]	Inferior by ≥100%	<0.001
[UNI] Preservation Unicondylar, fixed	437	14.95	13.18	[10.18 , 16.18]	Inferior by ≥100%	<0.001
[UNI] Sled Unicondylar, fixed	266	10.30	8.53	[5.51 , 11.55]	Inferior by ≥100%	<0.001

Supplementary table 10: Difference in cumulative percentage revision of knee implants compared to a contemporary benchmark at 10 years post primary in women for knee replacements with ≥250 procedures remaining at risk

Knee brand, bearing and constraint	Number at risk	Cumulative failure (%)	Difference in failure (%)	95% CI	Equivalence status	p-value
[TKR] Genesis 2 Cement, unconstrained fixed	1,175	2.13	[REFERENCE]			
[TKR] AGC Cement, unconstrained fixed	5,614	2.97	0.84	[0.45 , 1.23]	Inferior by ≥20%	<0.001
[TKR] Advance MP Cement, unconstrained fixed	443	4.02	1.90	[0.93 , 2.86]	Inferior by ≥20%	<0.001
[TKR] Genesis 2 Oxinium Cement, unconstrained fixed	259	4.33	2.20	[0.99 , 3.41]	Inferior by ≥20%	<0.001
[TKR] Insall-Burstein 2 Cement, posterior-stabilised fixed	670	4.25	2.13	[0.92 , 3.33]	Inferior by ≥20%	<0.001
[TKR] Kinemax Cement, unconstrained fixed	2,936	4.47	2.35	[1.70 , 2.99]	Inferior by ≥20%	<0.001
[TKR] LCS Cement, unconstrained mobile	269	3.15	1.02	[-0.85 , 2.89]	Non-inferiority not shown	0.142
[TKR] LCS Complete Cement, unconstrained mobile	479	4.29	2.16	[1.34 , 2.98]	Inferior by ≥20%	<0.001
[TKR] LCS Complete Uncemented, unconstrained mobile	738	3.30	1.17	[0.57 , 1.77]	Inferior by ≥20%	<0.001
[TKR] LCS Uncemented, unconstrained mobile	565	2.16	0.04	[-1.06 , 1.13]	Non-inferiority not shown	0.473
[TKR] MRK Cement, unconstrained fixed	341	2.19	0.07	[-0.67 , 0.80]	Non-inferiority not shown	0.431
[TKR] Natural Knee II Cement, unconstrained fixed	362	3.65	1.53	[0.38 , 2.67]	Non-inferiority not shown	0.004
[TKR] NexGen Cement, posterior-stabilised fixed	3,498	3.98	1.85	[1.40 , 2.30]	Inferior by ≥20%	<0.001
[TKR] NexGen Cement, unconstrained fixed	1,721	2.45	0.33	[-0.11 , 0.76]	Non-inferiority not shown	0.069
[TKR] NexGen Uncemented, unconstrained fixed	450	4.30	2.17	[1.08 , 3.26]	Inferior by ≥20%	<0.001
[TKR] PFC Sigma Bicondylar Knee Cement, posterior-stabilised mobile	444	3.87	1.75	[0.88 , 2.61]	Inferior by ≥20%	<0.001
[TKR] PFC Sigma Bicondylar Knee Cement, posterior-stabilised fixed	4,825	2.90	0.78	[0.40 , 1.16]	Non-inferiority not shown	<0.001
[TKR] PFC Sigma Bicondylar Knee Cement, unconstrained fixed	11,524	2.24	0.11	[-0.22 , 0.44]	Non-inferiority not shown	0.258
[TKR] PFC Sigma Bicondylar Knee Cement, unconstrained mobile	467	3.82	1.69	[0.87 , 2.51]	Inferior by ≥20%	<0.001
[TKR] PFC Sigma Bicondylar Knee Hybrid, unconstrained fixed	363	2.06	-0.07	[-1.09 , 0.96]	Non-inferiority not shown	0.450
[TKR] Profix Uncemented, unconstrained fixed	287	2.10	-0.03	[-0.93 , 0.88]	Non-inferiority not shown	0.477
[TKR] Rotaglide + Cement, unconstrained mobile	407	4.62	2.49	[1.01 , 3.97]	Inferior by ≥20%	<0.001
[TKR] Scorpio Cement, posterior-stabilised mobile	274	3.07	0.94	[-0.40 , 2.28]	Non-inferiority not shown	0.084
[TKR] Scorpio Cement, posterior-stabilised fixed	1,201	3.32	1.19	[0.47 , 1.91]	Inferior by ≥20%	0.001
[TKR] Scorpio Cement, unconstrained fixed	1,974	3.41	1.29	[0.70 , 1.87]	Inferior by ≥20%	<0.001
[TKR] Scorpio Uncemented, unconstrained fixed	283	4.01	1.89	[0.69 , 3.08]	Inferior by ≥20%	0.001
[TKR] TC Plus Cement, unconstrained fixed	730	3.03	0.90	[0.23 , 1.57]	Non-inferiority not shown	0.004
[TKR] TC Plus Cement, unconstrained mobile	825	2.40	0.28	[-0.45 , 1.01]	Non-inferiority not shown	0.226
[UNI] MG Uni Unicondylar, fixed	408	11.71	9.58	[7.46 , 11.71]	Inferior by ≥100%	<0.001
[UNI] Oxford Partial Knee Unicondylar, mobile	3,066	12.51	10.38	[9.68 , 11.09]	Inferior by ≥100%	<0.001

Supplementary table 11: Difference in cumulative percentage revision of knee implants compared to a contemporary benchmark at 3 years post primary in men less than 55 years for knee replacements with ≥250 procedures remaining at risk

Knee brand, bearing and constraint	Number at risk	Cumulative failure (%)	Difference in failure (%)	95% CI	Equivalence status	p-value
[TKR] PFC Sigma Bicondylar Knee Cement, unconstrained fixed	2,538	2.85	[REFERENCE]			
[TKR] AGC Cement, unconstrained fixed	915	3.55	0.70	[-0.57 , 1.98]	Non-inferiority not shown	0.140
[TKR] Genesis 2 Cement, unconstrained fixed	311	3.41	0.57	[-1.24 , 2.38]	Non-inferiority not shown	0.270
[TKR] Genesis 2 Oxinium Cement, unconstrained fixed	458	3.15	0.30	[-1.28 , 1.89]	Non-inferiority not shown	0.354
[TKR] LCS Complete Uncemented, unconstrained mobile	331	4.59	1.74	[-0.41 , 3.90]	Non-inferiority not shown	0.057
[TKR] NexGen Cement, posterior-stabilised fixed	761	4.36	1.52	[0.11 , 2.93]	Non-inferiority not shown	0.017
[TKR] NexGen Cement, unconstrained fixed	781	2.92	0.07	[-1.09 , 1.23]	Non-inferiority not shown	0.452
[TKR] PFC Sigma Bicondylar Knee Cement, posterior-stabilised mobile	408	3.30	0.46	[-1.28 , 2.20]	Non-inferiority not shown	0.304
[TKR] PFC Sigma Bicondylar Knee Cement, posterior-stabilised fixed	1,137	3.48	0.64	[-0.49 , 1.76]	Non-inferiority not shown	0.134
[TKR] PFC Sigma Bicondylar Knee Cement, unconstrained mobile	528	3.42	0.57	[-1.01 , 2.15]	Non-inferiority not shown	0.240
[TKR] Triathlon Cement, posterior-stabilised fixed	256	2.79	-0.05	[-1.88 , 1.78]	Non-inferiority not shown	0.477
[TKR] Triathlon Cement, unconstrained fixed	865	2.63	-0.21	[-1.26 , 0.84]	Non-inferiority not shown	0.346
[TKR] Vanguard Cement, unconstrained fixed	584	3.61	0.77	[-0.68 , 2.22]	Non-inferiority not shown	0.150
[UNI] Oxford Partial Knee Unicondylar, mobile	2,693	6.80	3.95	[2.94 , 4.97]	Inferior by ≥100%	<0.001
[UNI] Physica ZUK Unicondylar, fixed	551	2.61	-0.24	[-1.48 , 1.00]	Non-inferiority not shown	0.353
[UNI] Sigma HP Unicondylar, fixed	481	3.99	1.14	[-0.43 , 2.71]	Non-inferiority not shown	0.078

Supplementary table 12: Difference in cumulative percentage revision of knee implants compared to a contemporary benchmark at 5 years post primary in men less than 55 years for knee replacements with ≥250 procedures remaining at risk

Knee brand, bearing and constraint	Number at risk	Cumulative failure (%)	Difference in failure (%)	95% CI	Equivalence status	p-value
[TKR] PFC Sigma Bicondylar Knee Cement, unconstrained fixed	1,753	4.00	[REFERENCE]			
[TKR] AGC Cement, unconstrained fixed	740	5.29	1.29	[-0.30 , 2.88]	Non-inferiority not shown	0.056
[TKR] Genesis 2 Oxinium Cement, unconstrained fixed	325	4.65	0.65	[-1.37 , 2.67]	Non-inferiority not shown	0.263
[TKR] NexGen Cement, posterior-stabilised fixed	520	6.80	2.80	[0.93 , 4.67]	Inferior by ≥20%	0.002
[TKR] NexGen Cement, unconstrained fixed	454	3.75	-0.25	[-1.68 , 1.19]	Non-inferiority not shown	0.368
[TKR] PFC Sigma Bicondylar Knee Cement, posterior-stabilised mobile	306	4.31	0.31	[-1.72 , 2.34]	Non-inferiority not shown	0.383
[TKR] PFC Sigma Bicondylar Knee Cement, posterior-stabilised fixed	796	4.85	0.85	[-0.54 , 2.25]	Non-inferiority not shown	0.115
[TKR] PFC Sigma Bicondylar Knee Cement, unconstrained mobile	415	4.63	0.63	[-1.26 , 2.51]	Non-inferiority not shown	0.258
[TKR] Triathlon Cement, unconstrained fixed	452	3.69	-0.31	[-1.69 , 1.07]	Non-inferiority not shown	0.331
[TKR] Vanguard Cement, unconstrained fixed	255	4.79	0.79	[-0.98 , 2.56]	Non-inferiority not shown	0.190
[UNI] Oxford Partial Knee Unicondylar, mobile	1,972	9.71	5.71	[4.44 , 6.98]	Inferior by ≥100%	<0.001
[UNI] Physica ZUK Unicondylar, fixed	289	4.17	0.17	[-1.57 , 1.92]	Non-inferiority not shown	0.423

Supplementary table 13: Difference in cumulative percentage revision of knee implants compared to a contemporary benchmark at 7 years post primary in men less than 55 years for knee replacements with ≥250 procedures remaining at risk

Knee brand, bearing and constraint	Number at risk	Cumulative failure (%)	Difference in failure (%)	95% CI	Equivalence status	p-value
[TKR] PFC Sigma Bicondylar Knee Cement, unconstrained fixed	1,100	5.11	[REFERENCE]			
[TKR] AGC Cement, unconstrained fixed	500	7.67	2.56	[0.54 , 4.58]	Non-inferiority not shown	0.006
[TKR] NexGen Cement, posterior-stabilised fixed	320	9.92	4.82	[2.32 , 7.31]	Inferior by ≥20%	<0.001
[TKR] PFC Sigma Bicondylar Knee Cement, posterior-stabilised fixed	468	5.92	0.81	[-0.87 , 2.50]	Non-inferiority not shown	0.172
[TKR] PFC Sigma Bicondylar Knee Cement, unconstrained mobile	283	5.48	0.37	[-1.80 , 2.54]	Non-inferiority not shown	0.370
[UNI] Oxford Partial Knee Unicondylar, mobile	1,254	12.89	7.78	[6.21 , 9.35]	Inferior by ≥100%	<0.001

Supplementary table 14: Difference in cumulative percentage revision of knee implants compared to a contemporary benchmark at 3 years post primary in women less than 55 years for knee replacements with ≥250 procedures remaining at risk

Knee brand, bearing and constraint	Number at risk	Cumulative failure (%)	Difference in failure (%)	95% CI	Equivalence status	p-value
[TKR] NexGen Cement, unconstrained fixed	1,105	2.18	[REFERENCE]			
[TKR] AGC Cement, unconstrained fixed	1,331	2.56	0.39	[-0.72 , 1.50]	Non-inferiority not shown	0.248
[TKR] Genesis 2 Cement, unconstrained fixed	472	1.79	-0.38	[-1.65 , 0.89]	Non-inferiority not shown	0.278
[TKR] Genesis 2 Oxinium Cement, posterior-stabilised fixed	301	3.11	0.93	[-0.97 , 2.83]	Non-inferiority not shown	0.168
[TKR] Genesis 2 Oxinium Cement, unconstrained fixed	556	2.66	0.48	[-0.98 , 1.95]	Non-inferiority not shown	0.260
[TKR] Kinemax Cement, unconstrained fixed	266	2.92	0.74	[-1.39 , 2.88]	Non-inferiority not shown	0.248
[TKR] LCS Complete Uncemented, unconstrained mobile	412	3.54	1.36	[-0.46 , 3.19]	Non-inferiority not shown	0.072
[TKR] NexGen Cement, posterior-stabilised fixed	1,253	2.99	0.81	[-0.32 , 1.94]	Non-inferiority not shown	0.080
[TKR] PFC Sigma Bicondylar Knee Cement, posterior-stabilised mobile	436	4.36	2.18	[0.21 , 4.16]	Non-inferiority not shown	0.015
[TKR] PFC Sigma Bicondylar Knee Cement, posterior-stabilised fixed	1,637	3.08	0.90	[-0.18 , 1.99]	Non-inferiority not shown	0.052
[TKR] PFC Sigma Bicondylar Knee Cement, unconstrained fixed	3,827	2.27	0.10	[-0.78 , 0.97]	Non-inferiority not shown	0.415
[TKR] PFC Sigma Bicondylar Knee Cement, unconstrained mobile	520	3.29	1.11	[-0.53 , 2.76]	Non-inferiority not shown	0.092
[TKR] Triathlon Cement, posterior-stabilised fixed	364	3.91	1.74	[-0.17 , 3.64]	Non-inferiority not shown	0.037
[TKR] Triathlon Cement, unconstrained fixed	1,260	2.53	0.35	[-0.71 , 1.42]	Non-inferiority not shown	0.258
[TKR] Vanguard Cement, unconstrained fixed	807	1.39	-0.79	[-1.80 , 0.22]	Non-inferior	0.064
[UNI] Oxford Partial Knee Unicondylar, mobile	3,251	5.92	3.75	[2.70 , 4.80]	Inferior by ≥100%	<0.001
[UNI] Physica ZUK Unicondylar, fixed	526	4.48	2.30	[0.64 , 3.96]	Inferior by ≥20%	0.003
[UNI] Sigma HP Unicondylar, fixed	395	6.28	4.10	[1.96 , 6.25]	Inferior by ≥20%	<0.001

Supplementary table 15: Difference in cumulative percentage revision of knee implants compared to a contemporary benchmark at 5 years post primary in women less than 55 years for knee replacements with ≥250 procedures remaining at risk

Knee brand, bearing and constraint	Number at risk	Cumulative failure (%)	Difference in failure (%)	95% CI	Equivalence status	p-value
[TKR] PFC Sigma Bicondylar Knee Cement, unconstrained fixed	2,522	3.60	[REFERENCE]			
[TKR] AGC Cement, unconstrained fixed	1,072	4.19	0.59	[-0.62 , 1.80]	Non-inferiority not shown	0.168
[TKR] Genesis 2 Cement, unconstrained fixed	282	2.51	-1.09	[-2.51 , 0.32]	Non-inferior	0.065
[TKR] Genesis 2 Oxinium Cement, unconstrained fixed	392	4.68	1.08	[-0.76 , 2.92]	Non-inferiority not shown	0.125
[TKR] Kinemax Cement, unconstrained fixed	258	4.77	1.17	[-1.43 , 3.76]	Non-inferiority not shown	0.189
[TKR] LCS Complete Uncemented, unconstrained mobile	316	5.70	2.10	[-0.17 , 4.37]	Non-inferiority not shown	0.035
[TKR] NexGen Cement, posterior-stabilised fixed	859	4.62	1.02	[-0.23 , 2.27]	Non-inferiority not shown	0.054
[TKR] NexGen Cement, unconstrained fixed	642	3.94	0.34	[-0.92 , 1.60]	Non-inferiority not shown	0.300
[TKR] PFC Sigma Bicondylar Knee Cement, posterior-stabilised mobile	333	5.51	1.91	[-0.23 , 4.06]	Non-inferiority not shown	0.040
[TKR] PFC Sigma Bicondylar Knee Cement, posterior-stabilised fixed	1,153	4.93	1.33	[0.16 , 2.51]	Non-inferiority not shown	0.013
[TKR] PFC Sigma Bicondylar Knee Cement, unconstrained mobile	423	5.73	2.13	[0.08 , 4.18]	Non-inferiority not shown	0.021
[TKR] Triathlon Cement, unconstrained fixed	681	4.15	0.55	[-0.68 , 1.77]	Non-inferiority not shown	0.191
[TKR] Vanguard Cement, unconstrained fixed	341	2.57	-1.03	[-2.27 , 0.21]	Non-inferior	0.052
[UNI] Oxford Partial Knee Unicondylar, mobile	2,358	9.86	6.26	[5.12 , 7.39]	Inferior by ≥100%	<0.001
[UNI] Physica ZUK Unicondylar, fixed	256	6.21	2.61	[0.65 , 4.57]	Non-inferiority not shown	0.005

Supplementary table 16: Difference in cumulative percentage revision of knee implants compared to a contemporary benchmark at 7 years post primary in women less than 55 years for knee replacements with ≥250 procedures remaining at risk

Knee brand, bearing and constraint	Number at risk	Cumulative failure (%)	Difference in failure (%)	95% CI	Equivalence status	p-value
[TKR] PFC Sigma Bicondylar Knee Cement, unconstrained fixed	1,538	4.39	[REFERENCE]			
[TKR] AGC Cement, unconstrained fixed	735	5.15	0.75	[-0.65 , 2.16]	Non-inferiority not shown	0.147
[TKR] NexGen Cement, posterior-stabilised fixed	551	6.60	2.21	[0.55 , 3.86]	Non-inferiority not shown	0.004
[TKR] NexGen Cement, unconstrained fixed	334	5.20	0.81	[-0.84 , 2.46]	Non-inferiority not shown	0.169
[TKR] PFC Sigma Bicondylar Knee Cement, posterior-stabilised fixed	720	5.57	1.18	[-0.16 , 2.51]	Non-inferiority not shown	0.042
[TKR] PFC Sigma Bicondylar Knee Cement, unconstrained mobile	285	7.06	2.67	[0.30 , 5.03]	Non-inferiority not shown	0.014
[TKR] Triathlon Cement, unconstrained fixed	268	5.80	1.40	[-0.32 , 3.13]	Non-inferiority not shown	0.056
[UNI] Oxford Partial Knee Unicondylar, mobile	1,495	13.01	8.62	[7.24 , 10.00]	Inferior by ≥100%	<0.001

Supplementary table 17: Difference in cumulative percentage revision of knee implants compared to a contemporary benchmark at 3 years post primary in men between 55 and 75 years for knee replacements with ≥250 procedures remaining at risk

Knee brand, bearing and constraint	Number at risk	Cumulative failure (%)	Difference in failure (%)	95% CI	Equivalence status	p-value
[TKR] NexGen Cement, unconstrained fixed	10,041	1.34	[REFERENCE]			
[TKR] AGC Cement, unconstrained fixed	14,760	1.71	0.37	[0.09 , 0.65]	Non-inferiority not shown	0.005
[TKR] AGC Hybrid, unconstrained fixed	274	2.77	1.43	[-0.47 , 3.33]	Non-inferiority not shown	0.070
[TKR] AGC Uncemented, unconstrained fixed	402	3.74	2.40	[0.59 , 4.21]	Inferior by ≥20%	0.005
[TKR] Advance MP Cement, unconstrained fixed	1,736	2.43	1.09	[0.41 , 1.77]	Inferior by ≥20%	0.001
[TKR] Columbus Cement, unconstrained fixed	1,543	1.73	0.39	[-0.20 , 0.97]	Non-inferiority not shown	0.096
[TKR] E-Motion Bicondylar Knee Uncemented, unconstrained mobile	516	1.61	0.27	[-0.74 , 1.29]	Non-inferiority not shown	0.299
[TKR] Genesis 2 Cement, posterior-stabilised fixed	2,118	2.39	1.05	[0.45 , 1.64]	Inferior by ≥20%	<0.001
[TKR] Genesis 2 Cement, unconstrained fixed	7,447	1.61	0.27	[-0.05 , 0.59]	Non-inferiority not shown	0.047
[TKR] Genesis 2 Oxinium Cement, posterior-stabilised fixed	447	3.07	1.73	[0.27 , 3.19]	Non-inferiority not shown	0.010
[TKR] Genesis 2 Oxinium Cement, unconstrained fixed	1,105	2.22	0.88	[0.04 , 1.72]	Non-inferiority not shown	0.020
[TKR] Insall-Burstein 2 Cement, posterior-stabilised fixed	684	2.25	0.91	[-0.20 , 2.01]	Non-inferiority not shown	0.054
[TKR] Journey Oxinium Cement, posterior-stabilised fixed	252	3.43	2.09	[-0.12 , 4.29]	Non-inferiority not shown	0.032
[TKR] Kinemax Cement, unconstrained fixed	2,948	2.12	0.78	[0.23 , 1.32]	Non-inferiority not shown	0.003
[TKR] LCS Complete Cement, unconstrained mobile	2,624	1.76	0.42	[-0.09 , 0.94]	Non-inferiority not shown	0.053
[TKR] LCS Complete Uncemented, unconstrained mobile	3,377	1.87	0.53	[0.07 , 0.99]	Non-inferiority not shown	0.012
[TKR] LCS Uncemented, unconstrained mobile	376	2.31	0.97	[-0.53 , 2.47]	Non-inferiority not shown	0.103
[TKR] MRK Cement, unconstrained fixed	1,730	1.62	0.28	[-0.29 , 0.84]	Non-inferiority not shown	0.166
[TKR] Maxim Cement, unconstrained fixed	406	1.44	0.10	[-1.06 , 1.26]	Non-inferiority not shown	0.432
[TKR] NRG Cement, posterior-stabilised fixed	1,179	1.84	0.50	[-0.25 , 1.26]	Non-inferiority not shown	0.096
[TKR] NRG Cement, unconstrained fixed	1,693	2.06	0.72	[0.07 , 1.38]	Non-inferiority not shown	0.015
[TKR] Natural Knee II Cement, unconstrained fixed	712	1.33	-0.01	[-0.85 , 0.83]	Non-inferiority not shown	0.490
[TKR] NexGen Cement, posterior-stabilised fixed	11,497	2.14	0.80	[0.49 , 1.11]	Inferior by ≥20%	<0.001
[TKR] NexGen Hybrid, unconstrained fixed	332	0.56	-0.78	[-1.58 , 0.01]	Non-inferior	0.027
[TKR] NexGen Uncemented, posterior-stabilised fixed	564	1.27	-0.07	[-0.92 , 0.78]	Non-inferiority not shown	0.437
[TKR] NexGen Uncemented, unconstrained fixed	1,469	2.24	0.90	[0.15 , 1.65]	Non-inferiority not shown	0.009
[TKR] Optetrak Cement, posterior-stabilised fixed	417	2.72	1.38	[-0.15 , 2.92]	Non-inferiority not shown	0.038
[TKR] PFC Sigma Bicondylar Knee Cement, posterior-stabilised mobile	1,927	1.93	0.60	[-0.02 , 1.21]	Non-inferiority not shown	0.028
[TKR] PFC Sigma Bicondylar Knee Cement, posterior-stabilised fixed	15,069	1.72	0.38	[0.11 , 0.66]	Non-inferiority not shown	0.003
[TKR] PFC Sigma Bicondylar Knee Cement, unconstrained fixed	37,425	1.49	0.15	[-0.07 , 0.38]	Non-inferiority not shown	0.089
[TKR] PFC Sigma Bicondylar Knee Cement, unconstrained mobile	2,249	1.94	0.60	[0.02 , 1.17]	Non-inferiority not shown	0.021
[TKR] PFC Sigma Bicondylar Knee Hybrid, unconstrained fixed	499	0.97	-0.37	[-1.24 , 0.50]	Non-inferiority not shown	0.202
[TKR] PFC Sigma Bicondylar Knee monobloc polyethylene tibia	1,086	1.39	0.05	[-0.56 , 0.66]	Non-inferiority not shown	0.439
[TKR] Profix Cement, unconstrained fixed	304	2.18	0.84	[-0.77 , 2.44]	Non-inferiority not shown	0.154
[TKR] Profix Uncemented, unconstrained fixed	631	1.23	-0.11	[-0.98 , 0.75]	Non-inferiority not shown	0.399
[TKR] Rotaglide + Cement, unconstrained mobile	468	4.00	2.67	[0.93 , 4.40]	Inferior by ≥20%	0.001
[TKR] Rotaglide Cement, unconstrained mobile	264	3.61	2.27	[0.17 , 4.38]	Non-inferiority not shown	0.017
[TKR] Scorpio Cement, posterior-stabilised mobile	388	1.98	0.64	[-0.73 , 2.02]	Non-inferiority not shown	0.179
[TKR] Scorpio Cement, posterior-stabilised fixed	1,531	2.37	1.03	[0.26 , 1.81]	Non-inferiority not shown	0.004
[TKR] Scorpio Cement, unconstrained fixed	2,891	2.60	1.27	[0.67 , 1.86]	Inferior by ≥20%	<0.001

[TKR] Scorpio Cement, unconstrained mobile	341	1.97	0.63	[-0.83 , 2.09]	Non-inferiority not shown	0.198
[TKR] Scorpio Hybrid, unconstrained fixed	269	2.13	0.79	[-0.91 , 2.49]	Non-inferiority not shown	0.181
[TKR] Scorpio Uncemented, unconstrained fixed	1,184	1.96	0.63	[-0.18 , 1.43]	Non-inferiority not shown	0.063
[TKR] TC Plus Cement, unconstrained fixed	2,427	2.45	1.11	[0.48 , 1.75]	Inferior by ≥20%	<0.001
[TKR] TC Plus Cement, unconstrained mobile	1,442	2.13	0.79	[0.05 , 1.54]	Non-inferiority not shown	0.019
[TKR] TC Plus Uncemented, unconstrained mobile	373	1.03	-0.30	[-1.23 , 0.62]	Non-inferiority not shown	0.260
[TKR] Triathlon Cement, posterior-stabilised fixed	2,263	2.00	0.66	[0.14 , 1.18]	Non-inferiority not shown	0.006
[TKR] Triathlon Cement, unconstrained fixed	8,595	1.44	0.10	[-0.18 , 0.39]	Non-inferiority not shown	0.244
[TKR] Vanguard Cement, posterior-stabilised fixed	833	1.96	0.62	[-0.14 , 1.38]	Non-inferiority not shown	0.054
[TKR] Vanguard Cement, unconstrained fixed	5,651	1.50	0.16	[-0.17 , 0.48]	Non-inferiority not shown	0.169
[UNI] AMC/Uniglide Unicondylar, fixed	335	2.93	1.59	[-0.13 , 3.30]	Non-inferiority not shown	0.035
[UNI] AMC/Uniglide Unicondylar, mobile	458	8.38	7.04	[4.69 , 9.40]	Inferior by ≥100%	<0.001
[UNI] MG Uni Unicondylar, fixed	888	3.13	1.79	[0.65 , 2.93]	Inferior by ≥20%	0.001
[UNI] Oxford Partial Knee Unicondylar, mobile	14,444	3.69	2.35	[2.01 , 2.69]	Inferior by ≥100%	<0.001
[UNI] Physica ZUK Unicondylar, fixed	1,765	2.33	0.99	[0.36 , 1.62]	Inferior by ≥20%	0.001
[UNI] Preservation Unicondylar, fixed	455	4.16	2.82	[1.03 , 4.62]	Inferior by ≥20%	0.001
[UNI] Sigma HP Unicondylar, fixed	1,404	2.97	1.64	[0.89 , 2.38]	Inferior by ≥20%	<0.001

Supplementary table 18: Difference in cumulative percentage revision of knee implants compared to a contemporary benchmark at 5 years post primary in men between 55 and 75 years for knee replacements with ≥250 procedures remaining at risk

Knee brand, bearing and constraint	Number at risk	Cumulative failure (%)	Difference in failure (%)	95% CI	Equivalence status	p-value
[TKR] NexGen Cement, unconstrained fixed	6,119	1.92	[REFERENCE]			
[TKR] AGC Cement, unconstrained fixed	11,861	2.48	0.56	[0.21 , 0.91]	Non-inferiority not shown	0.001
[TKR] AGC Hybrid, unconstrained fixed	258	3.14	1.23	[-0.81 , 3.27]	Non-inferiority not shown	0.119
[TKR] AGC Uncemented, unconstrained fixed	350	4.52	2.60	[0.60 , 4.60]	Inferior by ≥20%	0.005
[TKR] Advance MP Cement, unconstrained fixed	1,248	3.28	1.37	[0.53 , 2.20]	Inferior by ≥20%	0.001
[TKR] Columbus Cement, unconstrained fixed	879	2.61	0.70	[-0.10 , 1.49]	Non-inferiority not shown	0.044
[TKR] E-Motion Bicondylar Knee Uncemented, unconstrained mobile	382	2.28	0.36	[-0.90 , 1.63]	Non-inferiority not shown	0.287
[TKR] Genesis 2 Cement, posterior-stabilised fixed	1,221	3.38	1.47	[0.69 , 2.24]	Inferior by ≥20%	<0.001
[TKR] Genesis 2 Cement, unconstrained fixed	4,599	2.39	0.47	[0.05 , 0.89]	Non-inferiority not shown	0.014
[TKR] Genesis 2 Oxinium Cement, posterior-stabilised fixed	259	4.21	2.29	[0.45 , 4.13]	Inferior by ≥20%	0.007
[TKR] Genesis 2 Oxinium Cement, unconstrained fixed	841	2.94	1.02	[0.02 , 2.03]	Non-inferiority not shown	0.023
[TKR] Insall-Burstein 2 Cement, posterior-stabilised fixed	643	3.70	1.79	[0.37 , 3.21]	Non-inferiority not shown	0.007
[TKR] Kinemax Cement, unconstrained fixed	2,804	3.37	1.46	[0.76 , 2.15]	Inferior by ≥20%	<0.001
[TKR] LCS Complete Cement, unconstrained mobile	2,077	3.22	1.30	[0.58 , 2.02]	Inferior by ≥20%	<0.001
[TKR] LCS Complete Uncemented, unconstrained mobile	2,561	2.43	0.51	[-0.04 , 1.07]	Non-inferiority not shown	0.035
[TKR] LCS Uncemented, unconstrained mobile	359	3.10	1.19	[-0.56 , 2.93]	Non-inferiority not shown	0.092
[TKR] MRK Cement, unconstrained fixed	1,182	2.35	0.43	[-0.30 , 1.16]	Non-inferiority not shown	0.123
[TKR] Maxim Cement, unconstrained fixed	375	2.19	0.27	[-1.17 , 1.71]	Non-inferiority not shown	0.356
[TKR] NRG Cement, posterior-stabilised fixed	893	2.58	0.66	[-0.26 , 1.58]	Non-inferiority not shown	0.080
[TKR] NRG Cement, unconstrained fixed	1,162	3.42	1.51	[0.61 , 2.40]	Inferior by ≥20%	<0.001
[TKR] Natural Knee II Cement, unconstrained fixed	563	1.91	-0.01	[-1.03 , 1.02]	Non-inferiority not shown	0.496
[TKR] NexGen Cement, posterior-stabilised fixed	8,138	3.17	1.25	[0.85 , 1.65]	Inferior by ≥20%	<0.001
[TKR] NexGen Hybrid, unconstrained fixed	282	1.47	-0.45	[-1.75 , 0.86]	Non-inferiority not shown	0.251
[TKR] NexGen Uncemented, posterior-stabilised fixed	415	1.69	-0.22	[-1.27 , 0.82]	Non-inferiority not shown	0.336
[TKR] NexGen Uncemented, unconstrained fixed	1,234	2.97	1.05	[0.17 , 1.94]	Non-inferiority not shown	0.010
[TKR] Optetrak Cement, posterior-stabilised fixed	380	4.38	2.47	[0.52 , 4.41]	Inferior by ≥20%	0.006
[TKR] PFC Sigma Bicondylar Knee Cement, posterior-stabilised mobile	1,449	2.32	0.41	[-0.29 , 1.10]	Non-inferiority not shown	0.125
[TKR] PFC Sigma Bicondylar Knee Cement, posterior-stabilised fixed	10,973	2.29	0.37	[0.03 , 0.72]	Non-inferiority not shown	0.016
[TKR] PFC Sigma Bicondylar Knee Cement, unconstrained fixed	26,348	1.97	0.05	[-0.24 , 0.34]	Non-inferior	0.364
[TKR] PFC Sigma Bicondylar Knee Cement, unconstrained mobile	1,860	2.36	0.45	[-0.21 , 1.11]	Non-inferiority not shown	0.092
[TKR] PFC Sigma Bicondylar Knee Hybrid, unconstrained fixed	454	1.60	-0.32	[-1.45 , 0.81]	Non-inferiority not shown	0.289
[TKR] PFC Sigma Bicondylar Knee monobloc polyethylene tibia	458	1.75	-0.16	[-0.92 , 0.59]	Non-inferiority not shown	0.336
[TKR] Profix Cement, unconstrained fixed	283	4.50	2.59	[0.27 , 4.91]	Non-inferiority not shown	0.014
[TKR] Profix Uncemented, unconstrained fixed	583	1.38	-0.53	[-1.47 , 0.40]	Non-inferiority not shown	0.131
[TKR] Rotaglide + Cement, unconstrained mobile	444	4.84	2.92	[1.02 , 4.83]	Inferior by ≥20%	0.001
[TKR] Scorpio Cement, posterior-stabilised mobile	366	3.00	1.08	[-0.61 , 2.77]	Non-inferiority not shown	0.105
[TKR] Scorpio Cement, posterior-stabilised fixed	1,441	3.55	1.63	[0.68 , 2.58]	Inferior by ≥20%	<0.001
[TKR] Scorpio Cement, unconstrained fixed	2,655	3.57	1.66	[0.94 , 2.37]	Inferior by ≥20%	<0.001
[TKR] Scorpio Cement, unconstrained mobile	325	3.43	1.51	[-0.41 , 3.43]	Non-inferiority not shown	0.062
[TKR] Scorpio Hybrid, unconstrained fixed	258	2.13	0.21	[-1.49 , 1.92]	Non-inferiority not shown	0.403

[TKR] Scorpio Uncemented, unconstrained fixed	1,053	2.82	0.90	[-0.07 , 1.87]	Non-inferiority not shown	0.034
[TKR] TC Plus Cement, unconstrained fixed	2,283	3.23	1.31	[0.58 , 2.05]	Inferior by ≥20%	<0.001
[TKR] TC Plus Cement, unconstrained mobile	1,340	2.89	0.97	[0.09 , 1.85]	Non-inferiority not shown	0.015
[TKR] TC Plus Uncemented, unconstrained mobile	303	1.58	-0.34	[-1.54 , 0.86]	Non-inferiority not shown	0.290
[TKR] Triathlon Cement, posterior-stabilised fixed	1,289	2.64	0.73	[0.07 , 1.38]	Non-inferiority not shown	0.015
[TKR] Triathlon Cement, unconstrained fixed	4,224	1.95	0.04	[-0.33 , 0.41]	Non-inferiority not shown	0.423
[TKR] Vanguard Cement, posterior-stabilised fixed	326	2.90	0.98	[-0.10 , 2.07]	Non-inferiority not shown	0.037
[TKR] Vanguard Cement, unconstrained fixed	2,489	2.10	0.18	[-0.25 , 0.61]	Non-inferiority not shown	0.207
[UNI] AMC/Uniglide Unicondylar, mobile	381	9.21	7.30	[4.82 , 9.77]	Inferior by ≥100%	<0.001
[UNI] MG Uni Unicondylar, fixed	845	4.35	2.43	[1.09 , 3.77]	Inferior by ≥20%	<0.001
[UNI] Oxford Partial Knee Unicondylar, mobile	10,935	5.34	3.42	[2.99 , 3.86]	Inferior by ≥100%	<0.001
[UNI] Physica ZUK Unicondylar, fixed	890	3.82	1.91	[0.99 , 2.83]	Inferior by ≥20%	<0.001
[UNI] Preservation Unicondylar, fixed	428	7.58	5.66	[3.27 , 8.06]	Inferior by ≥100%	<0.001
[UNI] Sigma HP Unicondylar, fixed	642	4.53	2.61	[1.55 , 3.67]	Inferior by ≥20%	<0.001

Supplementary table 19: Difference in cumulative percentage revision of knee implants compared to a contemporary benchmark at 7 years post primary in men between 55 and 75 years for knee replacements with ≥250 procedures remaining at risk

Knee brand, bearing and constraint	Number at risk	Cumulative failure (%)	Difference in failure (%)	95% CI	Equivalence status	p-value
[TKR] PFC Sigma Bicondylar Knee Cement, unconstrained fixed	16,697	2.31	[REFERENCE]			
[TKR] AGC Cement, unconstrained fixed	8,081	3.16	0.85	[0.52 , 1.18]	Inferior by ≥20%	<0.001
[TKR] Advance MP Cement, unconstrained fixed	777	3.82	1.51	[0.60 , 2.43]	Inferior by ≥20%	0.001
[TKR] Columbus Cement, unconstrained fixed	462	2.89	0.58	[-0.28 , 1.45]	Non-inferiority not shown	0.093
[TKR] E-Motion Bicondylar Knee Uncemented, unconstrained mobile	270	2.88	0.57	[-0.92 , 2.07]	Non-inferiority not shown	0.227
[TKR] Genesis 2 Cement, posterior-stabilised fixed	641	4.09	1.78	[0.87 , 2.70]	Inferior by ≥20%	<0.001
[TKR] Genesis 2 Cement, unconstrained fixed	2,429	2.96	0.65	[0.21 , 1.09]	Non-inferiority not shown	0.002
[TKR] Genesis 2 Oxinium Cement, unconstrained fixed	537	3.34	1.03	[-0.04 , 2.11]	Non-inferiority not shown	0.030
[TKR] Insall-Burstein 2 Cement, posterior-stabilised fixed	604	4.47	2.16	[0.61 , 3.71]	Inferior by ≥20%	0.003
[TKR] Kinemax Cement, unconstrained fixed	2,644	4.22	1.91	[1.17 , 2.65]	Inferior by ≥20%	<0.001
[TKR] LCS Complete Cement, unconstrained mobile	1,342	3.84	1.53	[0.76 , 2.30]	Inferior by ≥20%	<0.001
[TKR] LCS Complete Uncemented, unconstrained mobile	1,584	2.97	0.66	[0.05 , 1.27]	Non-inferiority not shown	0.016
[TKR] LCS Uncemented, unconstrained mobile	345	3.10	0.80	[-0.94 , 2.53]	Non-inferiority not shown	0.184
[TKR] MRK Cement, unconstrained fixed	762	3.31	1.00	[0.09 , 1.92]	Non-inferiority not shown	0.016
[TKR] Maxim Cement, unconstrained fixed	314	3.08	0.77	[-0.96 , 2.50]	Non-inferiority not shown	0.192
[TKR] NRG Cement, posterior-stabilised fixed	413	3.08	0.77	[-0.25 , 1.79]	Non-inferiority not shown	0.070
[TKR] NRG Cement, unconstrained fixed	666	3.68	1.37	[0.43 , 2.32]	Non-inferiority not shown	0.002
[TKR] Natural Knee II Cement, unconstrained fixed	417	3.49	1.18	[-0.29 , 2.66]	Non-inferiority not shown	0.057
[TKR] Nexgen Cement, posterior-stabilised fixed	5,295	4.16	1.85	[1.44 , 2.27]	Inferior by ≥20%	<0.001
[TKR] Nexgen Cement, unconstrained fixed	3,522	2.48	0.17	[-0.19 , 0.53]	Non-inferiority not shown	0.180
[TKR] Nexgen Uncemented, ps fixed	313	2.01	-0.30	[-1.49 , 0.89]	Non-inferiority not shown	0.309
[TKR] Nexgen Uncemented, unconstrained fixed	968	3.30	0.99	[0.07 , 1.91]	Non-inferiority not shown	0.017
[TKR] PFC Sigma Bicondylar Knee Cement, PS mobile	915	2.80	0.49	[-0.27 , 1.26]	Non-inferiority not shown	0.103
[TKR] PFC Sigma Bicondylar Knee Cement, posterior-stabilised fixed	6,928	2.64	0.33	[0.03 , 0.63]	Non-inferiority not shown	0.016
[TKR] PFC Sigma Bicondylar Knee Cement, unconstrained mobile	1,328	2.85	0.54	[-0.17 , 1.25]	Non-inferiority not shown	0.067
[TKR] PFC Sigma Bicondylar Knee Hybrid, unconstrained fixed	391	1.60	-0.71	[-1.82 , 0.40]	Non-inferior	0.104
[TKR] Profix Cement, unconstrained fixed	251	5.23	2.92	[0.41 , 5.42]	Non-inferiority not shown	0.011
[TKR] Profix Uncemented, unconstrained fixed	469	1.95	-0.36	[-1.47 , 0.74]	Non-inferiority not shown	0.260
[TKR] Rotaglide + Cement, unconstrained mobile	377	5.78	3.47	[1.38 , 5.56]	Inferior by ≥20%	0.001
[TKR] Scorpio Cement, PS mobile	320	3.00	0.69	[-0.99 , 2.37]	Non-inferiority not shown	0.210
[TKR] Scorpio Cement, posterior-stabilised fixed	1,320	4.58	2.27	[1.22 , 3.33]	Inferior by ≥20%	<0.001
[TKR] Scorpio Cement, unconstrained fixed	2,216	4.36	2.05	[1.29 , 2.81]	Inferior by ≥20%	<0.001
[TKR] Scorpio Cement, unconstrained mobile	302	4.34	2.03	[-0.12 , 4.19]	Non-inferiority not shown	0.032
[TKR] Scorpio Uncemented, unconstrained fixed	740	3.50	1.19	[0.10 , 2.28]	Non-inferiority not shown	0.016
[TKR] TC Plus Cement, unconstrained fixed	1,784	3.69	1.38	[0.62 , 2.15]	Inferior by ≥20%	<0.001
[TKR] TC Plus Cement, unconstrained mobile	1,101	3.50	1.20	[0.24 , 2.15]	Non-inferiority not shown	0.007
[TKR] Triathlon Cement, posterior-stabilised fixed	443	2.81	0.50	[-0.16 , 1.16]	Non-inferiority not shown	0.070
[TKR] Triathlon Cement, unconstrained fixed	1,424	2.43	0.12	[-0.29 , 0.54]	Non-inferiority not shown	0.280
[TKR] Vanguard Cement, unconstrained fixed	749	2.28	-0.03	[-0.46 , 0.40]	Non-inferior	0.449
[UNI] AMC/Uniglide Unicondylar, mobile	253	11.09	8.78	[5.99 , 11.56]	Inferior by ≥100%	<0.001

[UNI] MG Uni Unicondylar, fixed	753	5.64	3.33	[1.82 , 4.85]	Inferior by ≥20%	<0.001
[UNI] Oxford Partial Knee Unicondylar, mobile	7,296	6.84	4.54	[4.09 , 4.98]	Inferior by ≥100%	<0.001
[UNI] Physica ZUK Unicondylar, fixed	432	4.54	2.23	[1.14 , 3.32]	Inferior by ≥20%	<0.001
[UNI] Preservation Unicondylar, fixed	388	9.77	7.46	[4.77 , 10.15]	Inferior by ≥100%	<0.001

Supplementary table 20: Difference in cumulative percentage revision of knee implants compared to a contemporary benchmark at 10 years post primary in men between 55 and 75 years for knee replacements with ≥250 procedures remaining at risk

Knee brand, bearing and constraint	Number at risk	Cumulative failure (%)	Difference in failure (%)	95% CI	Equivalence status	p-value
[TKR] PFC Sigma Bicondylar Knee Cement, unconstrained fixed	6,049	2.72	[REFERENCE]			
[TKR] AGC Cement, unconstrained fixed	2,803	4.43	1.71	[1.24 , 2.18]	Inferior by ≥20%	<0.001
[TKR] Genesis 2 Cement, unconstrained fixed	621	3.54	0.82	[0.22 , 1.42]	Non-inferiority not shown	0.004
[TKR] Insall-Burstein 2 Cement, posterior-stabilised fixed	362	7.30	4.58	[2.50 , 6.66]	Inferior by ≥20%	<0.001
[TKR] Kinemax Cement, unconstrained fixed	1,648	5.26	2.54	[1.69 , 3.38]	Inferior by ≥20%	<0.001
[TKR] LCS Complete Uncemented, unconstrained mobile	471	3.52	0.80	[0.06 , 1.54]	Non-inferiority not shown	0.017
[TKR] LCS Uncemented, unconstrained mobile	317	3.10	0.38	[-1.36 , 2.12]	Non-inferiority not shown	0.333
[TKR] Nexgen Cement, posterior-stabilised fixed	1,814	5.64	2.92	[2.33 , 3.51]	Inferior by ≥20%	<0.001
[TKR] Nexgen Cement, unconstrained fixed	890	2.85	0.13	[-0.34 , 0.61]	Non-inferiority not shown	0.293
[TKR] Nexgen Uncemented, unconstrained fixed	265	3.42	0.70	[-0.25 , 1.66]	Non-inferiority not shown	0.075
[TKR] PFC Sigma Bicondylar Knee Cement, posterior-stabilised fixed	2,369	3.13	0.41	[0.03 , 0.78]	Non-inferiority not shown	0.018
[TKR] PFC Sigma Bicondylar Knee Cement, unconstrained mobile	322	3.19	0.47	[-0.32 , 1.26]	Non-inferiority not shown	0.122
[TKR] Scorpio Cement, posterior-stabilised fixed	611	5.47	2.75	[1.56 , 3.95]	Inferior by ≥20%	<0.001
[TKR] Scorpio Cement, unconstrained fixed	1,044	4.99	2.27	[1.43 , 3.12]	Inferior by ≥20%	<0.001
[TKR] TC Plus Cement, unconstrained fixed	456	4.51	1.79	[0.81 , 2.78]	Inferior by ≥20%	<0.001
[TKR] TC Plus Cement, unconstrained mobile	527	4.48	1.76	[0.61 , 2.90]	Inferior by ≥20%	0.001
[UNI] MG Uni Unicondylar, fixed	346	7.79	5.07	[3.18 , 6.97]	Inferior by ≥100%	<0.001
[UNI] Oxford Partial Knee Unicondylar, mobile	2,454	9.97	7.25	[6.57 , 7.93]	Inferior by ≥100%	<0.001

Supplementary table 21: Difference in cumulative percentage revision of knee implants compared to a contemporary benchmark at 3 years post primary in women between 55 and 75 years for knee replacements with ≥250 procedures remaining at risk

Knee brand, bearing and constraint	Number at risk	Cumulative failure (%)	Difference in failure (%)	95% CI	Equivalence status	p-value
[TKR] TC Plus Cement, unconstrained mobile	1,695	1.000	[REFERENCE]			
[TKR] AGC Cement, unconstrained fixed	18,655	1.500	0.510	[0.02 , 0.99]	Non-inferiority not shown	0.021
[TKR] AGC Hybrid, unconstrained fixed	297	2.600	1.600	[-0.24 , 3.43]	Non-inferiority not shown	0.044
[TKR] AGC Uncemented, unconstrained fixed	377	5.720	4.730	[2.41 , 7.04]	Inferior by ≥100%	<0.001
[TKR] Advance MP Cement, unconstrained fixed	2,023	1.800	0.810	[0.10 , 1.52]	Non-inferiority not shown	0.013
[TKR] Advance MP Stature Cement, unconstrained fixed	494	1.540	0.540	[-0.51 , 1.59]	Non-inferiority not shown	0.158
[TKR] Advance PS Cement, posterior-stabilised fixed	282	2.730	1.730	[-0.09 , 3.55]	Non-inferiority not shown	0.031
[TKR] Columbus Cement, unconstrained fixed	1,895	1.810	0.810	[0.12 , 1.50]	Non-inferiority not shown	0.011
[TKR] E-Motion Bicondylar Knee Cement, unconstrained mobile	311	3.320	2.320	[0.47 , 4.16]	Inferior by ≥20%	0.007
[TKR] E-Motion Bicondylar Knee Uncemented, unconstrained mobile	495	2.220	1.220	[-0.06 , 2.51]	Non-inferiority not shown	0.031
[TKR] FS Cement, unconstrained fixed	253	1.160	0.160	[-1.22 , 1.55]	Non-inferiority not shown	0.408
[TKR] Genesis 2 Cement, posterior-stabilised fixed	3,026	1.770	0.770	[0.16 , 1.39]	Non-inferiority not shown	0.006
[TKR] Genesis 2 Cement, unconstrained fixed	9,401	1.200	0.200	[-0.30 , 0.70]	Non-inferiority not shown	0.213
[TKR] Genesis 2 Cement, unconstrained mobile	331	1.480	0.480	[-0.89 , 1.85]	Non-inferiority not shown	0.245
[TKR] Genesis 2 Oxinium Cement, posterior-stabilised fixed	615	2.210	1.210	[0.09 , 2.32]	Non-inferiority not shown	0.017
[TKR] Genesis 2 Oxinium Cement, unconstrained fixed	1,426	1.160	0.160	[-0.52 , 0.84]	Non-inferiority not shown	0.324
[TKR] Insall-Burstein 2 Cement, posterior-stabilised fixed	812	1.450	0.450	[-0.49 , 1.38]	Non-inferiority not shown	0.174
[TKR] Journey Oxinium Cement, posterior-stabilised fixed	311	4.310	3.310	[1.05 , 5.56]	Inferior by ≥100%	0.002
[TKR] Kinemax Cement, unconstrained fixed	3,607	2.160	1.170	[0.51 , 1.82]	Inferior by ≥20%	<0.001
[TKR] LCS Complete Cement, unconstrained mobile	3,170	1.850	0.850	[0.21 , 1.49]	Inferior by ≥20%	0.005
[TKR] LCS Complete Uncemented, unconstrained mobile	3,736	1.750	0.750	[0.15 , 1.35]	Non-inferiority not shown	0.007
[TKR] LCS Uncemented, unconstrained mobile	458	2.330	1.330	[-0.11 , 2.76]	Non-inferiority not shown	0.035
[TKR] MRK Cement, unconstrained fixed	2,339	1.070	0.070	[-0.52 , 0.66]	Non-inferiority not shown	0.407
[TKR] Maxim Cement, unconstrained fixed	478	1.840	0.840	[-0.44 , 2.11]	Non-inferiority not shown	0.099
[TKR] NRG Cement, posterior-stabilised fixed	1,574	2.030	1.030	[0.21 , 1.84]	Inferior by ≥20%	0.007
[TKR] NRG Cement, unconstrained fixed	2,181	1.410	0.410	[-0.24 , 1.06]	Non-inferiority not shown	0.107
[TKR] Natural Knee II Cement, unconstrained fixed	965	2.070	1.070	[0.08 , 2.06]	Non-inferiority not shown	0.017
[TKR] NexGen Cement, posterior-stabilised mobile	324	2.070	1.070	[-0.42 , 2.57]	Non-inferiority not shown	0.080
[TKR] NexGen Cement, posterior-stabilised fixed	15,772	1.490	0.490	[-0.00 , 0.98]	Non-inferiority not shown	0.025
[TKR] NexGen Cement, unconstrained fixed	13,193	1.030	0.030	[-0.45 , 0.52]	Non-inferiority not shown	0.447
[TKR] NexGen Hybrid, unconstrained fixed	333	1.150	0.160	[-1.06 , 1.37]	Non-inferiority not shown	0.401
[TKR] NexGen Uncemented, posterior-stabilised fixed	419	1.420	0.420	[-0.72 , 1.57]	Non-inferiority not shown	0.235
[TKR] NexGen Uncemented, unconstrained fixed	1,110	2.720	1.720	[0.69 , 2.76]	Inferior by ≥20%	0.001
[TKR] Optetrak Cement, posterior-stabilised fixed	579	3.140	2.140	[0.68 , 3.60]	Inferior by ≥20%	0.002
[TKR] PFC Sigma Bicondylar Knee Cement, posterior-stabilised mobile	2,132	1.940	0.940	[0.22 , 1.65]	Inferior by ≥20%	0.005
[TKR] PFC Sigma Bicondylar Knee Cement, posterior-stabilised fixed	20,665	1.600	0.600	[0.12 , 1.09]	Non-inferiority not shown	0.007
[TKR] PFC Sigma Bicondylar Knee Cement, unconstrained fixed	47,826	1.240	0.240	[-0.23 , 0.71]	Non-inferiority not shown	0.158
[TKR] PFC Sigma Bicondylar Knee Cement, unconstrained mobile	2,332	1.510	0.520	[-0.14 , 1.17]	Non-inferiority not shown	0.062
[TKR] PFC Sigma Bicondylar Knee Hybrid, unconstrained fixed	557	1.570	0.570	[-0.54 , 1.69]	Non-inferiority not shown	0.158
[TKR] PFC Sigma Bicondylar Knee monobloc polyethylene tibia	1,703	1.490	0.490	[-0.18 , 1.15]	Non-inferiority not shown	0.077

[TKR] Profix Cement, unconstrained fixed	335	1.170	0.180	[-1.06 , 1.41]	Non-inferiority not shown	0.390
[TKR] Profix Oxinium monobloc polyethylene tibia	270	2.180	1.180	[-0.60 , 2.96]	Non-inferiority not shown	0.098
[TKR] Profix Uncemented, unconstrained fixed	680	1.710	0.720	[-0.35 , 1.78]	Non-inferiority not shown	0.094
[TKR] Rotaglide + Cement, unconstrained mobile	597	2.120	1.120	[-0.11 , 2.35]	Non-inferiority not shown	0.037
[TKR] Rotaglide Cement, unconstrained mobile	397	1.760	0.760	[-0.53 , 2.06]	Non-inferiority not shown	0.124
[TKR] Scorpio Cement, posterior-stabilised mobile	483	1.210	0.210	[-0.85 , 1.28]	Non-inferiority not shown	0.349
[TKR] Scorpio Cement, posterior-stabilised fixed	2,156	1.450	0.450	[-0.23 , 1.13]	Non-inferiority not shown	0.097
[TKR] Scorpio Cement, unconstrained fixed	3,812	1.580	0.580	[-0.02 , 1.18]	Non-inferiority not shown	0.029
[TKR] Scorpio Cement, unconstrained mobile	462	2.310	1.310	[-0.11 , 2.74]	Non-inferiority not shown	0.035
[TKR] Scorpio Hybrid, unconstrained fixed	373	1.810	0.810	[-0.59 , 2.22]	Non-inferiority not shown	0.129
[TKR] Scorpio Uncemented, unconstrained fixed	1,215	1.900	0.900	[0.02 , 1.79]	Non-inferiority not shown	0.022
[TKR] TC Plus Cement, unconstrained fixed	2,740	1.660	0.670	[0.01 , 1.32]	Non-inferiority not shown	0.024
[TKR] TC Plus Uncemented, unconstrained mobile	565	1.810	0.810	[-0.31 , 1.92]	Non-inferiority not shown	0.078
[TKR] Triathlon Cement, posterior-stabilised fixed	3,116	1.680	0.680	[0.08 , 1.29]	Non-inferiority not shown	0.013
[TKR] Triathlon Cement, unconstrained fixed	11,030	1.580	0.580	[0.08 , 1.08]	Non-inferiority not shown	0.011
[TKR] Triathlon Uncemented, unconstrained fixed	262	2.860	1.860	[-0.05 , 3.77]	Non-inferiority not shown	0.028
[TKR] Vanguard Cement, posterior-stabilised fixed	1,116	2.040	1.040	[0.22 , 1.85]	Inferior by ≥20%	0.006
[TKR] Vanguard Cement, unconstrained fixed	7,465	1.380	0.380	[-0.13 , 0.89]	Non-inferiority not shown	0.072
[UNI] AMC/Uniglide Unicondylar, fixed	390	3.200	2.200	[0.49 , 3.92]	Inferior by ≥20%	0.006
[UNI] AMC/Uniglide Unicondylar, mobile	407	7.070	6.070	[3.66 , 8.47]	Inferior by ≥100%	<0.001
[UNI] MG Uni Unicondylar, fixed	673	3.990	2.990	[1.47 , 4.51]	Inferior by ≥100%	<0.001
[UNI] Oxford Partial Knee Unicondylar, mobile	12,307	3.830	2.830	[2.28 , 3.38]	Inferior by ≥100%	<0.001
[UNI] Physica ZUK Unicondylar, fixed	1,431	2.090	1.090	[0.30 , 1.89]	Inferior by ≥20%	0.003
[UNI] Preservation Unicondylar, fixed	333	8.230	7.230	[4.37 , 10.09]	Inferior by ≥100%	<0.001
[UNI] Sigma HP Unicondylar, fixed	959	3.090	2.090	[1.09 , 3.09]	Inferior by ≥100%	<0.001
[UNI] Sled Unicondylar, fixed	264	6.670	5.680	[2.81 , 8.54]	Inferior by ≥100%	<0.001

Supplementary table 22: Difference in cumulative percentage revision of knee implants compared to a contemporary benchmark at 5 years post primary in women between 55 and 75 years for knee replacements with ≥250 procedures remaining at risk

Knee brand, bearing and constraint	Number at risk	Cumulative failure (%)	Difference in failure (%)	95% CI	Equivalence status	p-value
[TKR] MRK Cement, unconstrained fixed	1,647	1.47	[REFERENCE]			
[TKR] AGC Cement, unconstrained fixed	15,179	2.11	0.64	[0.14 , 1.14]	Non-inferiority not shown	0.006
[TKR] AGC Hybrid, unconstrained fixed	282	2.93	1.46	[-0.48 , 3.40]	Non-inferiority not shown	0.071
[TKR] AGC Uncemented, unconstrained fixed	358	6.74	5.27	[2.77 , 7.77]	Inferior by ≥100%	<0.001
[TKR] Advance MP Cement, unconstrained fixed	1,520	2.45	0.98	[0.19 , 1.78]	Non-inferiority not shown	0.008
[TKR] Advance MP Stature Cement, unconstrained fixed	267	2.49	1.02	[-0.38 , 2.42]	Non-inferiority not shown	0.077
[TKR] Columbus Cement, unconstrained fixed	1,052	2.83	1.36	[0.50 , 2.23]	Inferior by ≥20%	0.001
[TKR] E-Motion Bicondylar Knee Uncemented, unconstrained mobile	387	2.88	1.41	[-0.07 , 2.88]	Non-inferiority not shown	0.031
[TKR] Genesis 2 Cement, posterior-stabilised fixed	1,842	2.39	0.92	[0.24 , 1.60]	Non-inferiority not shown	0.004
[TKR] Genesis 2 Cement, unconstrained fixed	5,902	1.59	0.12	[-0.39 , 0.64]	Non-inferiority not shown	0.320
[TKR] Genesis 2 Cement, unconstrained mobile	301	1.78	0.31	[-1.18 , 1.79]	Non-inferiority not shown	0.342
[TKR] Genesis 2 Oxinium Cement, posterior-stabilised fixed	391	3.33	1.86	[0.44 , 3.28]	Inferior by ≥20%	0.005
[TKR] Genesis 2 Oxinium Cement, unconstrained fixed	1,039	2.11	0.63	[-0.23 , 1.50]	Non-inferiority not shown	0.075
[TKR] Insall-Burstein 2 Cement, posterior-stabilised fixed	775	3.28	1.81	[0.51 , 3.11]	Inferior by ≥20%	0.003
[TKR] Kinemax Cement, unconstrained fixed	3,479	2.96	1.49	[0.78 , 2.20]	Inferior by ≥20%	<0.001
[TKR] LCS Complete Cement, unconstrained mobile	2,534	2.91	1.44	[0.70 , 2.17]	Inferior by ≥20%	<0.001
[TKR] LCS Complete Uncemented, unconstrained mobile	2,848	2.79	1.32	[0.63 , 2.01]	Inferior by ≥20%	<0.001
[TKR] LCS Uncemented, unconstrained mobile	438	2.98	1.51	[-0.10 , 3.11]	Non-inferiority not shown	0.033
[TKR] Maxim Cement, unconstrained fixed	432	2.48	1.01	[-0.45 , 2.47]	Non-inferiority not shown	0.088
[TKR] NRG Cement, posterior-stabilised fixed	1,187	2.73	1.26	[0.34 , 2.18]	Inferior by ≥20%	0.004
[TKR] NRG Cement, unconstrained fixed	1,577	2.12	0.65	[-0.10 , 1.40]	Non-inferiority not shown	0.044
[TKR] Natural Knee II Cement, unconstrained fixed	773	3.08	1.61	[0.43 , 2.80]	Inferior by ≥20%	0.004
[TKR] NexGen Cement, posterior-stabilised mobile	275	3.40	1.93	[-0.03 , 3.90]	Non-inferiority not shown	0.027
[TKR] NexGen Cement, posterior-stabilised fixed	11,170	2.61	1.14	[0.62 , 1.66]	Inferior by ≥20%	<0.001
[TKR] NexGen Cement, unconstrained fixed	8,293	1.58	0.11	[-0.40 , 0.61]	Non-inferiority not shown	0.338
[TKR] NexGen Hybrid, unconstrained fixed	303	1.46	-0.01	[-1.36 , 1.34]	Non-inferiority not shown	0.493
[TKR] NexGen Uncemented, posterior-stabilised fixed	339	1.68	0.21	[-1.04 , 1.47]	Non-inferiority not shown	0.370
[TKR] NexGen Uncemented, unconstrained fixed	1,007	3.46	1.98	[0.84 , 3.13]	Inferior by ≥20%	<0.001
[TKR] Optetrak Cement, posterior-stabilised fixed	508	5.04	3.57	[1.75 , 5.38]	Inferior by ≥100%	<0.001
[TKR] PFC Sigma Bicondylar Knee Cement, posterior-stabilised mobile	1,587	2.74	1.27	[0.45 , 2.08]	Inferior by ≥20%	0.001
[TKR] PFC Sigma Bicondylar Knee Cement, posterior-stabilised fixed	15,223	2.26	0.78	[0.29 , 1.28]	Non-inferiority not shown	0.001
[TKR] PFC Sigma Bicondylar Knee Cement, unconstrained fixed	34,361	1.75	0.28	[-0.19 , 0.75]	Non-inferiority not shown	0.121
[TKR] PFC Sigma Bicondylar Knee Cement, unconstrained mobile	1,974	2.28	0.81	[0.06 , 1.56]	Non-inferiority not shown	0.017
[TKR] PFC Sigma Bicondylar Knee Hybrid, unconstrained fixed	523	1.76	0.28	[-0.89 , 1.46]	Non-inferiority not shown	0.318
[TKR] PFC Sigma Bicondylar Knee monobloc polyethylene tibia	721	2.00	0.53	[-0.25 , 1.32]	Non-inferiority not shown	0.092
[TKR] Profix Cement, unconstrained fixed	315	2.08	0.61	[-0.98 , 2.20]	Non-inferiority not shown	0.228
[TKR] Profix Oxinium monobloc polyethylene tibia	262	2.91	1.44	[-0.60 , 3.48]	Non-inferiority not shown	0.083
[TKR] Profix Uncemented, unconstrained fixed	619	2.16	0.69	[-0.49 , 1.86]	Non-inferiority not shown	0.125
[TKR] Rotaglide + Cement, unconstrained mobile	571	2.78	1.31	[-0.07 , 2.69]	Non-inferiority not shown	0.032
[TKR] Rotaglide Cement, unconstrained mobile	299	3.20	1.72	[-0.07 , 3.52]	Non-inferiority not shown	0.030

[TKR] Scorpio Cement, posterior-stabilised mobile	455	2.04	0.57	[-0.76 , 1.90]	Non-inferiority not shown	0.201
[TKR] Scorpio Cement, posterior-stabilised fixed	2,061	2.23	0.76	[-0.01 , 1.53]	Non-inferiority not shown	0.026
[TKR] Scorpio Cement, unconstrained fixed	3,488	2.43	0.96	[0.29 , 1.62]	Non-inferiority not shown	0.002
[TKR] Scorpio Cement, unconstrained mobile	445	3.17	1.70	[0.05 , 3.34]	Non-inferiority not shown	0.022
[TKR] Scorpio Hybrid, unconstrained fixed	356	2.35	0.88	[-0.71 , 2.47]	Non-inferiority not shown	0.138
[TKR] Scorpio Uncemented, unconstrained fixed	1,106	2.49	1.02	[0.04 , 2.00]	Non-inferiority not shown	0.021
[TKR] TC Plus Cement, unconstrained fixed	2,588	2.39	0.92	[0.19 , 1.65]	Non-inferiority not shown	0.007
[TKR] TC Plus Cement, unconstrained mobile	1,584	1.48	0.01	[-0.72 , 0.74]	Non-inferiority not shown	0.488
[TKR] TC Plus Uncemented, unconstrained mobile	456	2.74	1.27	[-0.11 , 2.64]	Non-inferiority not shown	0.035
[TKR] Triathlon Cement, posterior-stabilised fixed	1,765	2.61	1.14	[0.43 , 1.85]	Inferior by ≥20%	0.001
[TKR] Triathlon Cement, unconstrained fixed	5,497	2.07	0.60	[0.08 , 1.12]	Non-inferiority not shown	0.011
[TKR] Vanguard Cement, posterior-stabilised fixed	426	2.94	1.46	[0.44 , 2.49]	Inferior by ≥20%	0.003
[TKR] Vanguard Cement, unconstrained fixed	3,246	2.20	0.72	[0.16 , 1.28]	Non-inferiority not shown	0.006
[UNI] AMC/Uniglide Unicondylar, fixed	275	4.59	3.12	[1.04 , 5.20]	Inferior by ≥20%	0.002
[UNI] AMC/Uniglide Unicondylar, mobile	346	9.71	8.23	[5.43 , 11.04]	Inferior by ≥100%	<0.001
[UNI] MG Uni Unicondylar, fixed	651	5.28	3.81	[2.09 , 5.53]	Inferior by ≥100%	<0.001
[UNI] Oxford Partial Knee Unicondylar, mobile	9,400	6.04	4.57	[3.96 , 5.18]	Inferior by ≥100%	<0.001
[UNI] Physica ZUK Unicondylar, fixed	754	3.52	2.05	[1.00 , 3.11]	Inferior by ≥20%	<0.001
[UNI] Preservation Unicondylar, fixed	318	11.84	10.37	[7.01 , 13.72]	Inferior by ≥100%	<0.001
[UNI] Sigma HP Unicondylar, fixed	446	5.02	3.54	[2.09 , 5.00]	Inferior by ≥100%	<0.001

Supplementary table 23: Difference in cumulative percentage revision of knee implants compared to a contemporary benchmark at 7 years post primary in women between 55 and 75 years for knee replacements with ≥250 procedures remaining at risk

Knee brand, bearing and constraint	Number at risk	Cumulative failure (%)	Difference in failure (%)	95% CI	Equivalence status	p-value
[TKR] TC Plus Cement, unconstrained mobile	1,341	1.95	[REFERENCE]			
[TKR] AGC Cement, unconstrained fixed	10,578	2.61	0.66	[-0.04 , 1.36]	Non-inferiority not shown	0.032
[TKR] AGC Uncemented, unconstrained fixed	278	8.16	6.21	[3.41 , 9.00]	Inferior by ≥100%	<0.001
[TKR] Advance MP Cement, unconstrained fixed	1,054	3.35	1.40	[0.35 , 2.45]	Non-inferiority not shown	0.005
[TKR] Columbus Cement, unconstrained fixed	585	2.95	1.00	[-0.01 , 2.01]	Non-inferiority not shown	0.026
[TKR] E-Motion Bicondylar Knee Uncemented, unconstrained mobile	279	4.06	2.11	[0.19 , 4.03]	Non-inferiority not shown	0.015
[TKR] Genesis 2 Cement, posterior-stabilised fixed	938	2.62	0.67	[-0.19 , 1.53]	Non-inferiority not shown	0.063
[TKR] Genesis 2 Cement, unconstrained fixed	3,140	2.06	0.11	[-0.62 , 0.84]	Non-inferiority not shown	0.383
[TKR] Genesis 2 Oxinium Cement, unconstrained fixed	632	2.69	0.74	[-0.37 , 1.85]	Non-inferiority not shown	0.096
[TKR] Insall-Burstein 2 Cement, posterior-stabilised fixed	738	4.30	2.35	[0.81 , 3.89]	Inferior by ≥20%	0.001
[TKR] Kinemax Cement, unconstrained fixed	3,294	3.96	2.01	[1.10 , 2.93]	Inferior by ≥20%	<0.001
[TKR] LCS Complete Cement, unconstrained mobile	1,676	3.88	1.94	[0.97 , 2.90]	Inferior by ≥20%	<0.001
[TKR] LCS Complete Uncemented, unconstrained mobile	1,824	3.09	1.14	[0.28 , 2.01]	Non-inferiority not shown	0.005
[TKR] LCS Uncemented, unconstrained mobile	425	2.98	1.03	[-0.64 , 2.70]	Non-inferiority not shown	0.114
[TKR] MRK Cement, unconstrained fixed	1,125	2.02	0.07	[-0.82 , 0.96]	Non-inferiority not shown	0.438
[TKR] Maxim Cement, unconstrained fixed	344	3.50	1.55	[-0.27 , 3.36]	Non-inferiority not shown	0.047
[TKR] NRG Cement, posterior-stabilised fixed	572	3.26	1.31	[0.17 , 2.45]	Non-inferiority not shown	0.012
[TKR] NRG Cement, unconstrained fixed	920	2.41	0.46	[-0.47 , 1.39]	Non-inferiority not shown	0.165
[TKR] Natural Knee II Cement, unconstrained fixed	584	3.82	1.87	[0.45 , 3.29]	Inferior by ≥20%	0.005
[TKR] NexGen Cement, posterior-stabilised fixed	7,194	3.32	1.37	[0.65 , 2.10]	Inferior by ≥20%	<0.001
[TKR] NexGen Cement, unconstrained fixed	5,031	2.15	0.21	[-0.51 , 0.92]	Non-inferiority not shown	0.286
[TKR] NexGen Uncemented, posterior-stabilised fixed	251	2.36	0.41	[-1.22 , 2.04]	Non-inferiority not shown	0.310
[TKR] NexGen Uncemented, unconstrained fixed	909	4.05	2.10	[0.78 , 3.43]	Inferior by ≥20%	0.001
[TKR] Optetrak Cement, posterior-stabilised fixed	319	5.23	3.28	[1.37 , 5.20]	Inferior by ≥20%	<0.001
[TKR] PFC Sigma Bicondylar Knee Cement, posterior-stabilised mobile	1,016	3.32	1.37	[0.35 , 2.40]	Non-inferiority not shown	0.004
[TKR] PFC Sigma Bicondylar Knee Cement, posterior-stabilised fixed	9,914	2.66	0.72	[0.02 , 1.41]	Non-inferiority not shown	0.022
[TKR] PFC Sigma Bicondylar Knee Cement, unconstrained fixed	22,205	2.06	0.11	[-0.56 , 0.78]	Non-inferiority not shown	0.375
[TKR] PFC Sigma Bicondylar Knee Cement, unconstrained mobile	1,414	2.45	0.50	[-0.40 , 1.41]	Non-inferiority not shown	0.138
[TKR] PFC Sigma Bicondylar Knee Hybrid, unconstrained fixed	453	1.76	-0.19	[-1.46 , 1.07]	Non-inferiority not shown	0.383
[TKR] PFC Sigma Bicondylar Knee monobloc polyethylene tibia	265	2.00	0.06	[-0.86 , 0.98]	Non-inferiority not shown	0.451
[TKR] Profix Cement, unconstrained fixed	289	2.73	0.78	[-1.10 , 2.66]	Non-inferiority not shown	0.208
[TKR] Profix Uncemented, unconstrained fixed	508	2.16	0.21	[-1.05 , 1.48]	Non-inferiority not shown	0.370
[TKR] Rotaglide + Cement, unconstrained mobile	497	3.13	1.19	[-0.35 , 2.72]	Non-inferiority not shown	0.065
[TKR] Scorpio Cement, posterior-stabilised mobile	393	2.50	0.55	[-1.00 , 2.09]	Non-inferiority not shown	0.243
[TKR] Scorpio Cement, posterior-stabilised fixed	1,896	2.92	0.97	[0.00 , 1.94]	Non-inferiority not shown	0.025
[TKR] Scorpio Cement, unconstrained fixed	2,905	2.86	0.92	[0.07 , 1.77]	Non-inferiority not shown	0.017
[TKR] Scorpio Cement, unconstrained mobile	415	4.49	2.54	[0.55 , 4.53]	Inferior by ≥20%	0.006
[TKR] Scorpio Hybrid, unconstrained fixed	324	2.94	0.99	[-0.85 , 2.82]	Non-inferiority not shown	0.145
[TKR] Scorpio Uncemented, unconstrained fixed	820	3.17	1.22	[0.03 , 2.41]	Non-inferiority not shown	0.023
[TKR] TC Plus Cement, unconstrained fixed	2,030	2.99	1.04	[0.12 , 1.96]	Non-inferiority not shown	0.013

[TKR] TC Plus Uncemented, unconstrained mobile	330	2.98	1.03	[-0.49 , 2.56]	Non-inferiority not shown	0.092
[TKR] Triathlon Cement, posterior-stabilised fixed	633	2.91	0.96	[0.04 , 1.88]	Non-inferiority not shown	0.020
[TKR] Triathlon Cement, unconstrained fixed	1,918	2.48	0.53	[-0.20 , 1.26]	Non-inferiority not shown	0.079
[TKR] Vanguard Cement, unconstrained fixed	1,009	2.74	0.80	[-0.02 , 1.61]	Non-inferiority not shown	0.027
[UNI] AMC/Uniglide Unicondylar, mobile	254	11.38	9.44	[6.34 , 12.53]	Inferior by ≥100%	<0.001
[UNI] MG Uni Unicondylar, fixed	584	6.64	4.69	[2.72 , 6.66]	Inferior by ≥100%	<0.001
[UNI] Oxford Partial Knee Unicondylar, mobile	6,459	8.09	6.14	[5.31 , 6.97]	Inferior by ≥100%	<0.001
[UNI] Physica ZUK Unicondylar, fixed	327	6.01	4.06	[2.32 , 5.81]	Inferior by ≥100%	<0.001
[UNI] Preservation Unicondylar, fixed	292	14.69	12.74	[9.02 , 16.45]	Inferior by ≥100%	<0.001

Supplementary table 24: Difference in cumulative percentage revision of knee implants compared to a contemporary benchmark at 10 years post primary in women between 55 and 75 years for knee replacements with ≥250 procedures remaining at risk

Knee brand, bearing and constraint	Number at risk	Cumulative failure (%)	Difference in failure (%)	95% CI	Equivalence status	p-value
[TKR] PFC Sigma Bicondylar Knee Cement, unconstrained fixed	8,221	2.4	[REFERENCE]			
[TKR] AGC Cement, unconstrained fixed	3,879	3.4	1.0	[0.65 , 1.35]	Inferior by ≥20%	<0.001
[TKR] Advance MP Cement, unconstrained fixed	299	4.4	2.0	[0.77 , 3.13]	Inferior by ≥20%	0.001
[TKR] Genesis 2 Cement, unconstrained fixed	853	2.4	0.0	[-0.45 , 0.41]	Non-inferior	0.462
[TKR] Insall-Burstein 2 Cement, posterior-stabilised fixed	489	5.3	2.9	[1.34 , 4.49]	Inferior by ≥20%	<0.001
[TKR] Kinemax Cement, unconstrained fixed	2,094	5.4	3.0	[2.20 , 3.76]	Inferior by ≥20%	<0.001
[TKR] LCS Complete Cement, unconstrained mobile	352	4.9	2.5	[1.50 , 3.53]	Inferior by ≥20%	<0.001
[TKR] LCS Complete Uncemented, unconstrained mobile	531	3.5	1.1	[0.43 , 1.76]	Non-inferiority not shown	0.001
[TKR] LCS Uncemented, unconstrained mobile	393	3.0	0.6	[-0.96 , 2.13]	Non-inferiority not shown	0.231
[TKR] NexGen Cement, posterior-stabilised fixed	2,484	4.4	2.0	[1.51 , 2.40]	Inferior by ≥20%	<0.001
[TKR] NexGen Cement, unconstrained fixed	1,217	2.7	0.3	[-0.14 , 0.72]	Non-inferiority not shown	0.095
[TKR] NexGen Uncemented, unconstrained fixed	322	4.5	2.1	[0.83 , 3.34]	Inferior by ≥20%	0.001
[TKR] PFC Sigma Bicondylar Knee Cement, posterior-stabilised mobile	313	3.3	0.9	[0.13 , 1.72]	Non-inferiority not shown	0.012
[TKR] PFC Sigma Bicondylar Knee Cement, posterior-stabilised fixed	3,360	3.3	0.9	[0.52 , 1.19]	Inferior by ≥20%	<0.001
[TKR] PFC Sigma Bicondylar Knee Cement, unconstrained mobile	338	3.1	0.7	[-0.15 , 1.45]	Non-inferiority not shown	0.056
[TKR] PFC Sigma Bicondylar Knee Hybrid, unconstrained fixed	270	2.0	-0.4	[-1.58 , 0.81]	Non-inferiority not shown	0.263
[TKR] Rotaglide + Cement, unconstrained mobile	289	4.8	2.4	[0.56 , 4.24]	Inferior by ≥20%	0.005
[TKR] Scorpio Cement, posterior-stabilised fixed	842	3.7	1.3	[0.42 , 2.13]	Non-inferiority not shown	0.002
[TKR] Scorpio Cement, unconstrained fixed	1,414	3.6	1.2	[0.52 , 1.83]	Inferior by ≥20%	<0.001
[TKR] TC Plus Cement, unconstrained fixed	504	3.6	1.2	[0.35 , 2.00]	Non-inferiority not shown	0.003
[TKR] TC Plus Cement, unconstrained mobile	635	2.5	0.1	[-0.71 , 0.92]	Non-inferiority not shown	0.399
[UNI] MG Uni Unicondylar, fixed	287	8.8	6.4	[4.12 , 8.61]	Inferior by ≥100%	<0.001
[UNI] Oxford Partial Knee Unicondylar, mobile	2,271	11.9	9.5	[8.75 , 10.29]	Inferior by ≥100%	<0.001

Supplementary table 25: Difference in cumulative percentage revision of knee implants compared to a contemporary benchmark at 3 years post primary in men over 75 years for knee replacements with ≥250 procedures remaining at risk

Knee brand, bearing and constraint	Number at risk	Cumulative failure (%)	Difference in failure (%)	95% CI	Equivalence status	p-value
[TKR] NexGen Cement, unconstrained fixed	3,575	0.63	[REFERENCE]			
[TKR] AGC Cement, unconstrained fixed	5,857	1.06	0.43	[0.11 , 0.76]	Non-inferiority not shown	0.004
[TKR] Advance MP Cement, unconstrained fixed	586	1.71	1.08	[0.13 , 2.02]	Inferior by ≥20%	0.013
[TKR] Columbus Cement, unconstrained fixed	541	1.58	0.96	[0.07 , 1.85]	Non-inferiority not shown	0.018
[TKR] Genesis 2 Cement, posterior-stabilised fixed	845	0.61	-0.02	[-0.49 , 0.46]	Non-inferiority not shown	0.468
[TKR] Genesis 2 Cement, unconstrained fixed	3,120	0.92	0.29	[-0.06 , 0.64]	Non-inferiority not shown	0.050
[TKR] Insall-Burstein 2 Cement, posterior-stabilised fixed	270	0.35	-0.28	[-0.99 , 0.44]	Non-inferiority not shown	0.223
[TKR] Kinemax Cement, unconstrained fixed	1,108	1.07	0.44	[-0.18 , 1.06]	Non-inferiority not shown	0.081
[TKR] LCS Complete Cement, unconstrained mobile	825	0.71	0.08	[-0.49 , 0.65]	Non-inferiority not shown	0.391
[TKR] LCS Complete Uncemented, unconstrained mobile	999	0.81	0.19	[-0.34 , 0.71]	Non-inferiority not shown	0.244
[TKR] MRK Cement, unconstrained fixed	644	1.02	0.39	[-0.31 , 1.10]	Non-inferiority not shown	0.138
[TKR] NRG Cement, posterior-stabilised fixed	447	1.60	0.97	[-0.15 , 2.09]	Non-inferiority not shown	0.045
[TKR] NRG Cement, unconstrained fixed	646	0.84	0.21	[-0.44 , 0.86]	Non-inferiority not shown	0.265
[TKR] Natural Knee II Cement, unconstrained fixed	250	0.00	--	[-.-- , -.--]	No failures to date	
[TKR] NexGen Cement, posterior-stabilised fixed	4,136	1.00	0.37	[0.03 , 0.71]	Non-inferiority not shown	0.016
[TKR] NexGen Uncemented, unconstrained fixed	267	0.68	0.05	[-0.91 , 1.02]	Non-inferiority not shown	0.456
[TKR] PFC Sigma Bicondylar Knee Cement, posterior-stabilised mobile	318	2.09	1.46	[0.01 , 2.91]	Non-inferiority not shown	0.024
[TKR] PFC Sigma Bicondylar Knee Cement, posterior-stabilised fixed	6,056	1.11	0.49	[0.17 , 0.81]	Inferior by ≥20%	0.001
[TKR] PFC Sigma Bicondylar Knee Cement, unconstrained fixed	13,197	0.84	0.22	[-0.04 , 0.47]	Non-inferiority not shown	0.048
[TKR] PFC Sigma Bicondylar Knee Cement, unconstrained mobile	375	1.53	0.90	[-0.25 , 2.05]	Non-inferiority not shown	0.062
[TKR] PFC Sigma Bicondylar Knee monobloc polyethylene tibia	1,004	1.26	0.64	[0.01 , 1.26]	Non-inferiority not shown	0.023
[TKR] Profix Uncemented, unconstrained fixed	296	0.63	0.00	[-0.89 , 0.89]	Non-inferiority not shown	0.498
[TKR] Scorpio Cement, posterior-stabilised fixed	624	0.89	0.26	[-0.48 , 1.00]	Non-inferiority not shown	0.246
[TKR] Scorpio Cement, unconstrained fixed	1,038	1.15	0.53	[-0.13 , 1.19]	Non-inferiority not shown	0.059
[TKR] Scorpio Uncemented, unconstrained fixed	373	0.52	-0.11	[-0.86 , 0.64]	Non-inferiority not shown	0.386
[TKR] TC Plus Cement, unconstrained fixed	831	1.54	0.91	[0.08 , 1.74]	Non-inferiority not shown	0.016
[TKR] TC Plus Cement, unconstrained mobile	418	1.02	0.39	[-0.52 , 1.31]	Non-inferiority not shown	0.201
[TKR] Triathlon Cement, posterior-stabilised fixed	764	0.78	0.15	[-0.38 , 0.68]	Non-inferiority not shown	0.289
[TKR] Triathlon Cement, unconstrained fixed	2,911	1.01	0.38	[0.02 , 0.74]	Non-inferiority not shown	0.018
[TKR] Vanguard Cement, posterior-stabilised fixed	291	0.36	-0.26	[-0.82 , 0.30]	Non-inferiority not shown	0.178
[TKR] Vanguard Cement, unconstrained fixed	1,887	0.97	0.34	[-0.07 , 0.75]	Non-inferiority not shown	0.053
[UNI] Oxford Partial Knee Unicondylar, mobile	2,355	2.45	1.82	[1.23 , 2.42]	Inferior by ≥100%	<0.001

Supplementary table 26: Difference in cumulative percentage revision of knee implants compared to a contemporary benchmark at 5 years post primary in men over 75 years for knee replacements with ≥250 procedures remaining at risk

Knee brand, bearing and constraint	Number at risk	Cumulative failure (%)	Difference in failure (%)	95% CI	Equivalence status	p-value
[TKR] Nexgen Cement, unconstrained fixed	2,068	0.90	[REFERENCE]			
[TKR] AGC Cement, unconstrained fixed	4,270	1.31	0.41	[0.01 , 0.81]	Non-inferiority not shown	0.022
[TKR] Advance MP Cement, unconstrained fixed	362	2.15	1.25	[0.11 , 2.40]	Non-inferiority not shown	0.016
[TKR] Columbus Cement, unconstrained fixed	273	1.58	0.69	[-0.22 , 1.60]	Non-inferiority not shown	0.070
[TKR] Genesis 2 Cement, posterior-stabilised fixed	445	1.04	0.14	[-0.56 , 0.84]	Non-inferiority not shown	0.350
[TKR] Genesis 2 Cement, unconstrained fixed	1,766	1.51	0.62	[0.11 , 1.12]	Non-inferiority not shown	0.008
[TKR] Kinemax Cement, unconstrained fixed	949	1.35	0.46	[-0.26 , 1.18]	Non-inferiority not shown	0.107
[TKR] LCS Complete Cement, unconstrained mobile	585	1.14	0.25	[-0.53 , 1.02]	Non-inferiority not shown	0.266
[TKR] LCS Complete Uncemented, unconstrained mobile	681	1.02	0.12	[-0.50 , 0.75]	Non-inferiority not shown	0.348
[TKR] MRK Cement, unconstrained fixed	413	1.18	0.28	[-0.51 , 1.07]	Non-inferiority not shown	0.245
[TKR] NRG Cement, posterior-stabilised fixed	303	2.34	1.44	[0.04 , 2.85]	Non-inferiority not shown	0.022
[TKR] NRG Cement, unconstrained fixed	378	0.84	-0.06	[-0.74 , 0.62]	Non-inferiority not shown	0.432
[TKR] Nexgen Cement, posterior-stabilised fixed	2,698	1.37	0.47	[0.03 , 0.91]	Non-inferiority not shown	0.017
[TKR] PFC Sigma Bicondylar Knee Cement, posterior-stabilised fixed	4,060	1.31	0.41	[0.02 , 0.80]	Non-inferiority not shown	0.019
[TKR] PFC Sigma Bicondylar Knee Cement, unconstrained fixed	8,777	1.02	0.12	[-0.20 , 0.44]	Non-inferiority not shown	0.234
[TKR] PFC Sigma Bicondylar Knee Cement, unconstrained mobile	292	2.41	1.51	[-0.01 , 3.03]	Non-inferiority not shown	0.026
[TKR] PFC Sigma Bicondylar Knee monobloc polyethylene tibia	431	1.40	0.51	[-0.20 , 1.21]	Non-inferiority not shown	0.080
[TKR] Scorpio Cement, posterior-stabilised fixed	543	1.39	0.49	[-0.46 , 1.44]	Non-inferiority not shown	0.154
[TKR] Scorpio Cement, unconstrained fixed	861	1.56	0.67	[-0.13 , 1.46]	Non-inferiority not shown	0.050
[TKR] Scorpio Uncemented, unconstrained fixed	304	0.79	-0.11	[-1.04 , 0.82]	Non-inferiority not shown	0.409
[TKR] TC Plus Cement, unconstrained fixed	719	1.79	0.89	[-0.02 , 1.81]	Non-inferiority not shown	0.028
[TKR] TC Plus Cement, unconstrained mobile	353	1.26	0.36	[-0.68 , 1.40]	Non-inferiority not shown	0.249
[TKR] Triathlon Cement, posterior-stabilised fixed	423	1.34	0.44	[-0.41 , 1.29]	Non-inferiority not shown	0.153
[TKR] Triathlon Cement, unconstrained fixed	1,328	1.39	0.49	[0.01 , 0.97]	Non-inferiority not shown	0.024
[TKR] Vanguard Cement, unconstrained fixed	749	1.34	0.44	[-0.10 , 0.99]	Non-inferiority not shown	0.054
[UNI] Oxford Partial Knee Unicondylar, mobile	1,681	3.30	2.40	[1.67 , 3.14]	Inferior by ≥100%	<0.001

Supplementary table 27: Difference in cumulative percentage revision of knee implants compared to a contemporary benchmark at 7 years post primary in men over 75 years for knee replacements with ≥250 procedures remaining at risk

Knee brand, bearing and constraint	Number at risk	Cumulative failure (%)	Difference in failure (%)	95% CI	Equivalence status	p-value
[TKR] NexGen Cement, unconstrained fixed	1,099	1.11	[REFERENCE]			
[TKR] AGC Cement, unconstrained fixed	2,540	1.54	0.43	[-0.05 , 0.91]	Non-inferiority not shown	0.040
[TKR] Genesis 2 Cement, unconstrained fixed	846	1.78	0.67	[0.06 , 1.28]	Non-inferiority not shown	0.015
[TKR] Kinemax Cement, unconstrained fixed	790	1.82	0.71	[-0.17 , 1.58]	Non-inferiority not shown	0.057
[TKR] LCS Complete Cement, unconstrained mobile	356	1.32	0.21	[-0.66 , 1.09]	Non-inferiority not shown	0.317
[TKR] LCS Complete Uncemented, unconstrained mobile	376	1.53	0.42	[-0.46 , 1.31]	Non-inferiority not shown	0.174
[TKR] NexGen Cement, posterior-stabilised fixed	1,561	1.60	0.49	[-0.03 , 1.02]	Non-inferiority not shown	0.033
[TKR] PFC Sigma Bicondylar Knee Cement, posterior-stabilised fixed	2,298	1.59	0.48	[-0.00 , 0.96]	Non-inferiority not shown	0.026
[TKR] PFC Sigma Bicondylar Knee Cement, unconstrained fixed	4,935	1.15	0.04	[-0.35 , 0.44]	Non-inferiority not shown	0.417
[TKR] Scorpio Cement, posterior-stabilised fixed	418	1.83	0.72	[-0.42 , 1.86]	Non-inferiority not shown	0.109
[TKR] Scorpio Cement, unconstrained fixed	634	1.56	0.45	[-0.37 , 1.27]	Non-inferiority not shown	0.140
[TKR] TC Plus Cement, unconstrained fixed	499	1.79	0.68	[-0.26 , 1.62]	Non-inferiority not shown	0.079
[TKR] TC Plus Cement, unconstrained mobile	261	1.58	0.47	[-0.76 , 1.70]	Non-inferiority not shown	0.228
[TKR] Triathlon Cement, unconstrained fixed	381	1.85	0.74	[-0.01 , 1.48]	Non-inferiority not shown	0.027
[UNI] Oxford Partial Knee Unicondylar, mobile	975	4.75	3.64	[2.64 , 4.64]	Inferior by ≥100%	<0.001

Supplementary table 28: Difference in cumulative percentage revision of knee implants compared to a contemporary benchmark at 10 years post primary in men over 75 years for knee replacements with ≥250 procedures remaining at risk

Knee brand, bearing and constraint	Number at risk	Cumulative failure (%)	Difference in failure (%)	95% CI	Equivalence status	p-value
[TKR] PFC Sigma Bicondylar Knee Cement, unconstrained fixed	1,319	1.25	[REFERENCE]			
[TKR] AGC Cement, unconstrained fixed	660	1.79	0.54	[0.08 , 1.00]	Non-inferiority not shown	0.011
[TKR] Kinemax Cement, unconstrained fixed	359	2.30	1.05	[0.05 , 2.06]	Non-inferiority not shown	0.019
[TKR] NexGen Cement, posterior-stabilised fixed	385	2.00	0.75	[0.12 , 1.39]	Non-inferiority not shown	0.010
[TKR] PFC Sigma Bicondylar Knee Cement, posterior-stabilised fixed	590	2.04	0.80	[0.23 , 1.36]	Non-inferiority not shown	0.003
[UNI] Oxford Partial Knee Unicondylar, mobile	290	5.90	4.65	[3.41 , 5.90]	Inferior by ≥100%	<0.001

Supplementary table 29: Difference in cumulative percentage revision of knee implants compared to a contemporary benchmark at 3 years post primary in women over 75 years for knee replacements with ≥250 procedures remaining at risk

Knee brand, bearing and constraint	Number at risk	Cumulative failure (%)	Difference in failure (%)	95% CI	Equivalence status	p-value
[TKR] Scorpio Cement, posterior-stabilised fixed	1,132	0.58	[REFERENCE]			
[TKR] AGC Cement, unconstrained fixed	9,844	0.75	0.17	[-0.28 , 0.63]	Non-inferiority not shown	0.229
[TKR] Advance MP Cement, unconstrained fixed	846	1.21	0.63	[-0.15 , 1.41]	Non-inferiority not shown	0.057
[TKR] Columbus Cement, unconstrained fixed	858	1.56	0.99	[0.18 , 1.80]	Inferior by ≥20%	0.009
[TKR] Genesis 2 Cement, posterior-stabilised fixed	1,628	0.96	0.39	[-0.19 , 0.96]	Non-inferiority not shown	0.095
[TKR] Genesis 2 Cement, unconstrained fixed	4,807	0.91	0.33	[-0.16 , 0.81]	Non-inferiority not shown	0.092
[TKR] Insall-Burstein 2 Cement, posterior-stabilised fixed	378	1.01	0.43	[-0.64 , 1.51]	Non-inferiority not shown	0.214
[TKR] Kinemax Cement, unconstrained fixed	1,824	0.63	0.05	[-0.50 , 0.61]	Non-inferiority not shown	0.426
[TKR] LCS Complete Cement, unconstrained mobile	1,434	1.12	0.54	[-0.11 , 1.19]	Non-inferiority not shown	0.050
[TKR] LCS Complete Uncemented, unconstrained mobile	1,444	0.97	0.39	[-0.23 , 1.02]	Non-inferiority not shown	0.110
[TKR] MRK Cement, unconstrained fixed	1,124	0.62	0.04	[-0.53 , 0.62]	Non-inferiority not shown	0.443
[TKR] NRG Cement, posterior-stabilised fixed	692	0.65	0.07	[-0.64 , 0.78]	Non-inferiority not shown	0.421
[TKR] NRG Cement, unconstrained fixed	991	0.42	-0.16	[-0.72 , 0.41]	Non-inferiority not shown	0.295
[TKR] Natural Knee II Cement, unconstrained fixed	439	0.44	-0.13	[-0.88 , 0.61]	Non-inferiority not shown	0.361
[TKR] NexGen Cement, posterior-stabilised fixed	7,284	0.84	0.27	[-0.20 , 0.73]	Non-inferiority not shown	0.129
[TKR] NexGen Cement, unconstrained fixed	5,865	0.67	0.10	[-0.37 , 0.56]	Non-inferiority not shown	0.343
[TKR] NexGen Uncemented, unconstrained fixed	259	1.09	0.51	[-0.78 , 1.80]	Non-inferiority not shown	0.220
[TKR] Optetrak Cement, posterior-stabilised fixed	253	1.46	0.88	[-0.60 , 2.37]	Non-inferiority not shown	0.121
[TKR] PFC Sigma Bicondylar Knee Cement, posterior-stabilised mobile	514	0.82	0.24	[-0.59 , 1.07]	Non-inferiority not shown	0.287
[TKR] PFC Sigma Bicondylar Knee Cement, posterior-stabilised fixed	10,390	0.80	0.22	[-0.23 , 0.68]	Non-inferiority not shown	0.167
[TKR] PFC Sigma Bicondylar Knee Cement, unconstrained fixed	21,321	0.69	0.12	[-0.32 , 0.55]	Non-inferiority not shown	0.299
[TKR] PFC Sigma Bicondylar Knee Cement, unconstrained mobile	573	1.29	0.72	[-0.22 , 1.66]	Non-inferiority not shown	0.068
[TKR] PFC Sigma Bicondylar Knee monobloc polyethylene tibia	1,500	0.82	0.24	[-0.33 , 0.81]	Non-inferiority not shown	0.205
[TKR] Profix Uncemented, unconstrained fixed	479	0.98	0.41	[-0.55 , 1.37]	Non-inferiority not shown	0.202
[TKR] Rotaglide + Cement, unconstrained mobile	276	2.03	1.46	[-0.21 , 3.12]	Non-inferiority not shown	0.043
[TKR] Scorpio Cement, unconstrained fixed	1,946	1.08	0.50	[-0.12 , 1.12]	Non-inferiority not shown	0.055
[TKR] Scorpio Uncemented, unconstrained fixed	602	1.27	0.69	[-0.28 , 1.66]	Non-inferiority not shown	0.082
[TKR] TC Plus Cement, unconstrained fixed	1,222	0.78	0.21	[-0.44 , 0.85]	Non-inferiority not shown	0.265
[TKR] TC Plus Cement, unconstrained mobile	684	0.66	0.09	[-0.63 , 0.80]	Non-inferiority not shown	0.408
[TKR] TC Plus Uncemented, unconstrained mobile	278	1.33	0.75	[-0.61 , 2.12]	Non-inferiority not shown	0.140
[TKR] Triathlon Cement, posterior-stabilised fixed	1,437	1.17	0.59	[-0.04 , 1.22]	Non-inferiority not shown	0.033
[TKR] Triathlon Cement, unconstrained fixed	4,541	1.14	0.56	[0.07 , 1.06]	Non-inferiority not shown	0.013
[TKR] Vanguard Cement, posterior-stabilised fixed	524	1.67	1.10	[0.13 , 2.06]	Inferior by ≥20%	0.013
[TKR] Vanguard Cement, unconstrained fixed	3,003	0.97	0.39	[-0.11 , 0.90]	Non-inferiority not shown	0.064
[UNI] Oxford Partial Knee Unicondylar, mobile	2,333	3.46	2.89	[2.10 , 3.68]	Inferior by ≥100%	<0.001

Supplementary table 30: Difference in cumulative percentage revision of knee implants compared to a contemporary benchmark at 5 years post primary in women over 75 years for knee replacements with ≥250 procedures remaining at risk

Knee brand, bearing and constraint	Number at risk	Cumulative failure (%)	Difference in failure (%)	95% CI	Equivalence status	p-value
[TKR] NexGen Cement, unconstrained fixed	3,677	0.77	[REFERENCE]			
[TKR] AGC Cement, unconstrained fixed	7,591	1.03	0.27	[-0.01 , 0.54]	Non-inferiority not shown	0.031
[TKR] Advance MP Cement, unconstrained fixed	598	2.15	1.39	[0.41 , 2.36]	Inferior by ≥20%	0.003
[TKR] Columbus Cement, unconstrained fixed	453	1.68	0.91	[0.16 , 1.67]	Inferior by ≥20%	0.009
[TKR] Genesis 2 Cement, posterior-stabilised fixed	928	1.35	0.58	[0.03 , 1.13]	Non-inferiority not shown	0.019
[TKR] Genesis 2 Cement, unconstrained fixed	2,845	1.04	0.27	[-0.06 , 0.60]	Non-inferiority not shown	0.052
[TKR] Insall-Burstein 2 Cement, posterior-stabilised fixed	349	1.55	0.79	[-0.46 , 2.03]	Non-inferiority not shown	0.109
[TKR] Kinemax Cement, unconstrained fixed	1,634	0.97	0.20	[-0.29 , 0.69]	Non-inferiority not shown	0.209
[TKR] LCS Complete Cement, unconstrained mobile	1,039	1.75	0.98	[0.30 , 1.66]	Inferior by ≥20%	0.002
[TKR] LCS Complete Uncemented, unconstrained mobile	1,043	1.21	0.44	[-0.13 , 1.01]	Non-inferiority not shown	0.064
[TKR] MRK Cement, unconstrained fixed	772	0.89	0.13	[-0.41 , 0.66]	Non-inferiority not shown	0.321
[TKR] NRG Cement, posterior-stabilised fixed	485	0.82	0.05	[-0.63 , 0.74]	Non-inferiority not shown	0.442
[TKR] NRG Cement, unconstrained fixed	626	0.56	-0.20	[-0.71 , 0.30]	Non-inferiority not shown	0.218
[TKR] Natural Knee II Cement, unconstrained fixed	348	0.44	-0.32	[-0.97 , 0.32]	Non-inferiority not shown	0.161
[TKR] NexGen Cement, posterior-stabilised fixed	4,965	1.19	0.42	[0.11 , 0.73]	Non-inferiority not shown	0.004
[TKR] PFC Sigma Bicondylar Knee Cement, posterior-stabilised mobile	363	1.55	0.78	[-0.33 , 1.89]	Non-inferiority not shown	0.083
[TKR] PFC Sigma Bicondylar Knee Cement, posterior-stabilised fixed	7,316	1.13	0.36	[0.08 , 0.64]	Non-inferiority not shown	0.006
[TKR] PFC Sigma Bicondylar Knee Cement, unconstrained fixed	14,883	0.94	0.17	[-0.06 , 0.40]	Non-inferiority not shown	0.077
[TKR] PFC Sigma Bicondylar Knee Cement, unconstrained mobile	457	1.68	0.91	[-0.10 , 1.93]	Non-inferiority not shown	0.039
[TKR] PFC Sigma Bicondylar Knee monobloc polyethylene tibia	747	1.09	0.32	[-0.21 , 0.85]	Non-inferiority not shown	0.116
[TKR] Profix Uncemented, unconstrained fixed	413	1.21	0.44	[-0.54 , 1.43]	Non-inferiority not shown	0.188
[TKR] Scorpio Cement, posterior-stabilised fixed	1,004	0.77	0.00	[-0.54 , 0.54]	Non-inferiority not shown	0.499
[TKR] Scorpio Cement, unconstrained fixed	1,668	1.45	0.69	[0.12 , 1.25]	Non-inferiority not shown	0.008
[TKR] Scorpio Uncemented, unconstrained fixed	532	1.97	1.20	[0.08 , 2.33]	Non-inferiority not shown	0.018
[TKR] TC Plus Cement, unconstrained fixed	1,097	1.20	0.44	[-0.20 , 1.07]	Non-inferiority not shown	0.090
[TKR] TC Plus Cement, unconstrained mobile	588	1.27	0.50	[-0.35 , 1.36]	Non-inferiority not shown	0.123
[TKR] Triathlon Cement, posterior-stabilised fixed	780	1.87	1.10	[0.40 , 1.81]	Inferior by ≥20%	0.001
[TKR] Triathlon Cement, unconstrained fixed	2,200	1.45	0.68	[0.31 , 1.05]	Inferior by ≥20%	<0.001
[TKR] Vanguard Cement, unconstrained fixed	1,253	1.18	0.42	[0.03 , 0.81]	Non-inferiority not shown	0.018
[UNI] Oxford Partial Knee Unicondylar, mobile	1,802	4.68	3.91	[3.09 , 4.74]	Inferior by ≥100%	<0.001

Supplementary table 31: Difference in cumulative percentage revision of knee implants compared to a contemporary benchmark at 7 years post primary in women over 75 years for knee replacements with ≥250 procedures remaining at risk

Knee brand, bearing and constraint	Number at risk	Cumulative failure (%)	Difference in failure (%)	95% CI	Equivalence status	p-value
[TKR] NexGen Cement, unconstrained fixed	2,110	0.98	[REFERENCE]			
[TKR] AGC Cement, unconstrained fixed	4,857	1.28	0.30	[-0.04 , 0.65]	Non-inferiority not shown	0.043
[TKR] Advance MP Cement, unconstrained fixed	383	2.15	1.17	[0.18 , 2.16]	Non-inferiority not shown	0.010
[TKR] Genesis 2 Cement, posterior-stabilised fixed	452	1.99	1.01	[0.15 , 1.87]	Non-inferiority not shown	0.011
[TKR] Genesis 2 Cement, unconstrained fixed	1,415	1.11	0.13	[-0.25 , 0.51]	Non-inferiority not shown	0.252
[TKR] Insall-Burstein 2 Cement, posterior-stabilised fixed	295	1.55	0.57	[-0.69 , 1.83]	Non-inferiority not shown	0.188
[TKR] Kinemax Cement, unconstrained fixed	1,416	1.23	0.24	[-0.33 , 0.82]	Non-inferiority not shown	0.201
[TKR] LCS Complete Cement, unconstrained mobile	653	1.85	0.87	[0.14 , 1.60]	Non-inferiority not shown	0.010
[TKR] LCS Complete Uncemented, unconstrained mobile	591	1.44	0.46	[-0.22 , 1.13]	Non-inferiority not shown	0.092
[TKR] MRK Cement, unconstrained fixed	494	1.03	0.05	[-0.57 , 0.66]	Non-inferiority not shown	0.440
[TKR] NRG Cement, unconstrained fixed	295	0.56	-0.42	[-0.95 , 0.11]	Non-inferior	0.062
[TKR] Natural Knee II Cement, unconstrained fixed	251	0.84	-0.14	[-1.16 , 0.87]	Non-inferiority not shown	0.391
[TKR] NexGen Cement, posterior-stabilised fixed	2,942	1.54	0.56	[0.16 , 0.96]	Non-inferiority not shown	0.003
[TKR] PFC Sigma Bicondylar Knee Cement, posterior-stabilised fixed	4,344	1.31	0.33	[-0.01 , 0.67]	Non-inferiority not shown	0.029
[TKR] PFC Sigma Bicondylar Knee Cement, unconstrained fixed	9,161	1.05	0.07	[-0.22 , 0.36]	Non-inferiority not shown	0.320
[TKR] PFC Sigma Bicondylar Knee Cement, unconstrained mobile	314	1.95	0.96	[-0.18 , 2.11]	Non-inferiority not shown	0.050
[TKR] PFC Sigma Bicondylar Knee monobloc polyethylene tibia	252	1.43	0.45	[-0.42 , 1.31]	Non-inferiority not shown	0.156
[TKR] Profix Uncemented, unconstrained fixed	300	1.21	0.23	[-0.77 , 1.23]	Non-inferiority not shown	0.327
[TKR] Scorpio Cement, posterior-stabilised fixed	842	0.97	-0.01	[-0.64 , 0.61]	Non-inferiority not shown	0.482
[TKR] Scorpio Cement, unconstrained fixed	1,272	1.65	0.67	[0.04 , 1.29]	Non-inferiority not shown	0.018
[TKR] Scorpio Uncemented, unconstrained fixed	350	1.97	0.99	[-0.15 , 2.12]	Non-inferiority not shown	0.044
[TKR] TC Plus Cement, unconstrained fixed	824	1.29	0.31	[-0.37 , 0.99]	Non-inferiority not shown	0.184
[TKR] TC Plus Cement, unconstrained mobile	456	1.27	0.29	[-0.58 , 1.16]	Non-inferiority not shown	0.257
[TKR] Triathlon Cement, posterior-stabilised fixed	260	2.31	1.33	[0.38 , 2.27]	Inferior by ≥20%	0.003
[TKR] Triathlon Cement, unconstrained fixed	665	1.51	0.52	[0.11 , 0.94]	Non-inferiority not shown	0.007
[TKR] Vanguard Cement, unconstrained fixed	366	1.39	0.41	[-0.10 , 0.92]	Non-inferiority not shown	0.059
[UNI] Oxford Partial Knee Unicondylar, mobile	1,146	6.42	5.44	[4.38 , 6.49]	Inferior by ≥100%	<0.001

Supplementary table 32: Difference in cumulative percentage revision of knee implants compared to a contemporary benchmark at 10 years post primary in women over 75 years for knee replacements with ≥250 procedures remaining at risk

Knee brand, bearing and constraint	Number at risk	Cumulative failure (%)	Difference in failure (%)	95% CI	Equivalence status	p-value
[TKR] PFC Sigma Bicondylar Knee Cement, unconstrained fixed	2,749	1.29	[REFERENCE]			
[TKR] AGC Cement, unconstrained fixed	1,448	1.48	0.19	[-0.15 , 0.54]	Non-inferiority not shown	0.136
[TKR] Genesis 2 Cement, unconstrained fixed	280	1.41	0.12	[-0.36 , 0.61]	Non-inferiority not shown	0.310
[TKR] Kinemax Cement, unconstrained fixed	691	1.55	0.27	[-0.40 , 0.93]	Non-inferiority not shown	0.216
[TKR] NexGen Cement, posterior-stabilised fixed	851	1.77	0.48	[0.07 , 0.90]	Non-inferiority not shown	0.011
[TKR] NexGen Cement, unconstrained fixed	447	1.08	-0.21	[-0.56 , 0.14]	Non-inferior	0.117
[TKR] PFC Sigma Bicondylar Knee Cement, posterior-stabilised fixed	1,202	1.46	0.18	[-0.15 , 0.50]	Non-inferiority not shown	0.148
[TKR] Scorpio Cement, posterior-stabilised fixed	305	1.72	0.44	[-0.54 , 1.41]	Non-inferiority not shown	0.191
[TKR] Scorpio Cement, unconstrained fixed	449	2.07	0.78	[0.04 , 1.52]	Non-inferiority not shown	0.019
[UNI] Oxford Partial Knee Unicondylar, mobile	356	8.61	7.32	[5.87 , 8.78]	Inferior by ≥100%	<0.001
