# Supplementary material for: Assessing the non-inferiority of prosthesis constructs used in total and unicondylar knee replacements using data from the National Joint Registry of England, Wales, Northern Ireland and the Isle of Man: a benchmarking study
Source: BMJ Open. 2019 Apr 29;9(4):e026736. doi: 10.1136/bmjopen-2018-026736 (PMC6502008; doi:10.1136/bmjopen-2018-026736)
Supplement: Author's manuscript [file bmjopen-2018-026736.draft_revisions.pdf]

BMJ Open

BMJ Open is committed to open peer review. As part of this commitment we make the peer review history of every article we publish publicly available.

When an article is published we post the peer reviewers' comments and the authors' responses online. We also post the versions of the paper that were used during peer review. These are the versions that the peer review comments apply to.

The versions of the paper that follow are the versions that were submitted during the peer review process. They are not the versions of record or the final published versions. They should not be cited or distributed as the published version of this manuscript.

BMJ Open is an open access journal and the full, final, typeset and author-corrected version of record of the manuscript is available on our site with no access controls, subscription charges or pay-per-view fees (http://bmjopen.bmj.com).

If you have any questions on BMJ Open's open peer review process please email info.bmjopen@bmj.com

BMJ Open

Assessing the non-inferiority of prosthesis used in total and unicondylar knee replacements using data from the National Joint Registry of England, Wales, Northern Ireland and the Isle of Man: A benchmarking study.

Journal:	*BMJ Open*
Manuscript ID	bmjopen-2018-026736
Article Type:	Research
Date Submitted by the Author:	19-Sep-2018

Complete List of Authors:	Deere, Kevin; University of Bristol, School of Social & Community Medicine<br>Whitehouse, Michael; University of Bristol, Musculoskeletal Research Unit<br>Porter, Martyn; Wrightington Hospital<br>Blom, AW; University of Bristol,<br>Sayers, Adrian; University of Bristol, Muscloskeletal Research Unit

Keywords:	knee arthroplasty, non-inferiority, National Joint Registry, benchmarking, medical devices

SCHOLARONE™
Manuscripts

Assessing the non-inferiority of prosthesis used in total and unicondylar knee replacements using data from the National Joint Registry of England, Wales, Northern Ireland and the Isle of Man: A benchmarking study.

Kevin Deere, Michael R Whitehouse, Martyn L Porter, Ashley W Blom, Adrian Sayers

Musculoskeletal Research Unit, Bristol Medical School, 1st Floor Learning & Research Building, Southmead Hospital, Bristol, BS10 5NB, UK

Kevin Deere
Senior research Associate

Michael R Whitehouse
Consultant Senior Lecturer in Trauma and Orthopaedics

Ashley W Blom
Professor of Orthopaedic Surgery and Head of Translational Health Sciences

Adrian Sayers
Senior Research Fellow

National Institute for Health Research Bristol Biomedical Research Centre, University of Bristol

Michael R Whitehouse
Consultant Senior Lecturer in Trauma and Orthopaedics

Ashley W Blom
Professor of Orthopaedic Surgery and Head of Translational Health Sciences

Centre for Hip Surgery, Wrightington Hospital, Lancashire, WN6 9EP, UK

Martyn L Porter
Consultant Orthopaedic Surgeon

Bristol Medical School, Population Health Sciences, University of Bristol, Bristol, UK

Adrian Sayers
Senior Research Fellow

Corresponding author: Adrian Sayers

Musculoskeletal Research Unit,

1st Floor Learning & Research Building,

Southmead Hospital,

Bristol,

BS10 5NB, UK

Email: adrian.sayers@bristol.ac.uk

Tel: 0117 4147880

Word count: 3,992

For peer review only

ABSTRACT

Objectives To investigate the relative performance of knee replacement implants compared to the best performing implant and illustrate the substantial variability in performance.

Design A non-inferiority study.

Setting England and Wales

Participants All primary total and unicondylar knee replacements performed and registered in the National Joint Registry (NJR) between 1st April 2003 and 31st December 2016.

Main outcome measures Kaplan-Meier failure function for knee replacement implants. Failure difference between best performing implant and other implants.

Methods Using a non-inferiority analysis, the performance of knee replacement implants by brand were compared to the best performing implant. Implant failure was estimated using the 1-Kaplan Meier method, i.e. an estimate of net failure. The difference in failure between the contemporary benchmark and all other implants were tested.

Results Of the 449 different knee replacement implants used, only 27 had ≥500 procedures at risk at 10 years post-surgery, 18 of which were classified as inferior to the benchmark by at least 20% relative risk of failure. In men, aged 55-75, 12 of 27 (44%) implants were inferior by at least 20% to the reference at 7 years post-surgery. In women, aged 55-75, eight of 32 (25%) implants were inferior at 7 years post-surgery. Very few implants were classified as non-inferior to the contemporary benchmark.

Conclusions There are few knee replacement implants that can be shown to be non-inferior to a contemporary benchmark. Unicondylar knee implants have, almost universally, at least 100% worse revision outcomes compared to the best performing total knee replacement. We have a duty to inform patients, clinicians and commissioners of this variability in performance of knee implants.

For peer review only - http://bmjopen.bmj.com/site/about/guidelines.xhtml

ARTICLE SUMMARY

Strengths and limitations of this study

- Data presented from the largest joint registry in the world
- A novel and systematic comparison of the performance of knee implants to a contemporary reference knee implant
- Unambiguous presentation of data will facilitate the consenting process for patients and allows surgeons and policy makers to be more informed with respect to success and failure of different implants options available in knee replacement
- Residual and unmeasured confounding factors are likely to be present.
- Potential for selection bias whereby certain implants may be implanted for particular indications and in particular groups with different risks

For peer review only

For peer review only - http://bmjopen.bmj.com/site/about/guidelines.xhtml

INTRODUCTION

Over 90,000 knee replacements are performed annually in England and Wales and there is a bewildering choice of total and unicondylar knee replacement options available from which clinicians and patients can choose. When patients are considering a knee replacement, it is understandable that many assume that the different implant implants function equally. However, all implants are not equal as evidenced by variation in revision rates between brands and knee implant types.[1] The National Joint Registry for England, Wales, Northern Ireland and the Isle of Man (NJR) is the largest arthroplasty database in the world and publishes the unadjusted cumulative failure rates of the most commonly used implants in knee replacement surgery. This is a useful format for measuring absolute failure but does not allow easy head-to-head comparison of implants. Bench-marking bodies such as the Orthopaedic Device Evaluation Panel (ODEP) in the UK,[2] NOV in the Netherlands,[3] and the Australian superior clinical performance program[4] compare implant performance against externally set benchmarks, but do not perform head-to-head comparison. Although it is reassuring a certain standard has been met, this simple dichotomisation does not facilitate head-to-head comparison and the sample sizes used are arbitrarily set. Sayers et al. recently proposed a method of comparison for joint replacement implants using a non-inferiority design against an external benchmark.[5] However, the primary limitation of this method remains the arbitrary requirement for an externally specified benchmark.

In a non-inferiority clinical trial[6] investigating failure, two treatments (comparator and reference) can be directly compared to ensure that the comparator treatment is within a clinically acceptable range (non-inferiority margin) of the performance of the reference treatment at a specified point in time.[7, 8] Standard methods for assessing non-inferiority could be applied in an orthopaedic benchmarking setting, assuming an appropriate comparator, non-inferiority margin, and time of interest can be identified. Choosing an appropriate contemporary reference is difficult. There is no gold standard or evidence from randomised trials that suggests any implant outperforms all others,

For peer review only - http://bmjopen.bmj.com/site/about/guidelines.xhtml

therefore the choice is more heuristic. Patients would like to receive the best available care and clinicians would like to provide the best possible care, or at least care that it is non-inferior to the best. Therefore, the natural choice of reference is the implant with the lowest failure rate. However, in order to protect against chance the implant should be used in large enough numbers to mitigate sampling variability. The failure rate of implants is influenced by both age and gender, therefore the choice of reference should reflect this specificity. The selection of an appropriate time and non-inferiority margin to assess implant performance is more subjective. For example, implant survivorship in the long term is less relevant to older patients with shorter life expectancy.

The aim of this study is to investigate the relative performance of knee replacement implants as compared to the best performing implant using a non-inferiority study design and to illustrate the substantial variability in performance of widely used implants. This research focuses on total knee replacements (TKRs) and unicondylar knee replacements (UKRs) as these are commonly used and therefore there is sufficient data to make robust comparisons. TKRs and UKRs are examined against non-inferiority margins of 20% relative risk and 100% relative risk at 3, 5, 7 and 10 years following surgery. This is predicated on our belief that patients would at least want reassurance that the implant they are to receive is proven to not be 100% worse than the best alternatives when used in patients with the same gender and age as them.

For peer review only

For peer review only - http://bmjopen.bmj.com/site/about/guidelines.xhtml

METHODS

Patients and Data Sources

We identified all patients with a primary TKR or UKR registered in the NJR between 1st April 2003 and December 31st 2016. All patients were consented to be included in the NJR as part of the standard NJR process.

Procedures were excluded if the patient age or gender were missing, or the National Health Service (NHS) number was untraceable and therefore mortality unknown. Procedures where the constraint or fixation type were unknown were excluded from the analysis. Patellofemoral replacements were also removed owing to the low number in the sample.

Brands of implants are usually subdivided by fixation, mobility of bearing and constraint. NJR Annual Report data has shown that these characteristics influence revision and thus we treated each subdivision as a separate implant.

Patient involvement

Patient representatives sit on the committee structure of the National Joint Registry. The research priorities of the National Joint Registry are identified by this committee structure and approved by the patient representatives.

Statistical methods

Using a non-inferiority analysis, the performance of knee implants were compared to an internally identified reference group. Implant failure was estimated using the 1-Kaplan Meier method i.e. an estimate of net failure, which is appropriate when the risk of revision is considered.[9]

Failure is defined using the first linked surgical revision. Patients were censored at death or administratively censored on 31st December 2016. The difference in stratum specific failure

For peer review only - http://bmjopen.bmj.com/site/about/guidelines.xhtml

probabilities compared to the reference were calculated at 3, 5, 7 and 10 years for all implants, stratified by gender, and stratified by gender and age group (<55 years, 55 to 75 years, >75 years).

The difference and 95% confidence interval (CI) of the difference between the comparator implant and the reference implant was estimated at the specified time points. The standard error of the difference was constructed using a pooled estimate of the Greenwood Standard error,[10]

$$SE(\widehat{Diff}) = \sqrt{GSE_x^2 + GSE_{ref}^2},$$

and a z-test comparing the difference between the reference and test implant was then constructed using,

$$Z = \left((\widehat{F_{test}} - \widehat{F_{ref}}) + \delta \right) / SE(\widehat{Diff}).$$

The stratum specific contemporaneous reference implant was selected as the knee implant (TKR or UKR) with the lowest failure rate with at least 1,000 patients at risk at the time point of interest.

Two non-inferiority margins were chosen to illustrate the sensitivity of the choice. The first margin was conservatively set at a 20% increase in relative risk of failure compared to the reference, in line with clinical trials using this methodology, albeit towards the upper end.[11] The second was a 100% increase in relative risk, i.e. a doubling in cumulative probability of failure, as this is an easily interpretable outcome.

Results are graphically reported for all comparator implants with at least 500 patients still at risk at the beginning of the time point of interest. Results are also reported in a tabular format for all comparator implants with at least 250 patients at risk at the beginning of the time point of interest (See supplementary tables).

Implants were either classified as non-inferior, inconclusive, or inferior. If the upper confidence interval is less than or equal to the 20% non-inferiority margin, the implant was non-inferior. If the lower confidence interval of the difference was greater than the non-inferiority margin at either 20%

For peer review only - http://bmjopen.bmj.com/site/about/guidelines.xhtml

or 100% the implant was classed as inferior at 20% or 100% respectively. If the lower confidence limit is less than the non-inferiority margin, and the upper confidence greater than non-inferiority margin the evidence against the implant was described as inconclusive (See figure 1 for graphical representation of the classification). All analyses were carried out using Stata 15.1 (College Station, Texas, USA).

Sensitivity analysis

We repeated all analyses using the best performing knee implant at 10 years with at least 1,000 still at risk in the stratum of interest as the reference at the 3, 5, and 7-year time points.

For peer review only

RESULTS

There were 975,739 primary knee replacements included in the NJR between 1st April 2003 and 31st December 2016, utilising 649 different combinations of brand, fixation, constraint and bearing type Following the application of the exclusion criteria (See supplementary fig 1), 947,686 procedures were included in the final analysis (863,551 (91.1%) TKRs), utilising 449 different combinations of brand, fixation, constraint and bearing type (405 TKRs and 44 UKRs).

A detailed description of non-inferiority across all procedures is provided. Due to the large number of clinically relevant subdivisions and sensitivity analyses, results will be described more broadly. Each stratification of age group, gender and time point of interest are provided in supplemental material.

Implants are described by brand, fixation, the degree of constraint for TKR and whether the bearing was fixed or mobile. The vast majority of reference implants were cemented. In each subdivision of our analyses the implant that met our reference criteria was a TKR that was unconstrained with a fixed bearing. However, not all total knees that were unconstrained with fixed bearings performed well as there were 15 separate brands of this type that were found to be inferior to the reference by at least 20% relative risk in at least one subdivision analysis.

Non-inferiority - All procedures

The reference implant at 3 years was identified as the NexGen cemented, unconstrained TKR with a fixed bearing. There were 34,558 procedures remaining at risk at 3 years for this implant and the failure rate was 1.10% (95%CI 1.01 – 1.20). There were 73 implants with ≥500 procedures at risk. Thirty-nine implants were classified as inferior to the reference by at least 20% relative risk of failure. Nine of the 73 were shown to be inferior by at least 100% relative risk (supplementary figure 2). One prosthesis, the NexGen TKR with a monobloc polyethylene tibia, was non-inferior.

For peer review only - http://bmjopen.bmj.com/site/about/guidelines.xhtml

The reference implant at 5 years was identified as the Profix uncemented unconstrained TKR with a fixed bearing. There were 1,910 procedures remaining at risk and the failure rate was 1.54% (95%CI 1.10 – 2.15). There were 65 implants with ≥500 procedures at risk. Thirty-six were classified as inferior to the reference by at least 20% relative risk of failure. Twelve of the 36 were shown to be inferior by at least 100% relative risk (figure 2). All of the unicondylar implants with ≥500 procedures at risk (n=8) were inferior by at least 100% relative risk. No implant could be described as non-inferior.

The reference implant at 7 years was identified, again, as the Profix uncemented unconstrained TKR with a fixed bearing. There were 1,501 procedures remaining at risk and the failure rate was 1.77% (95%CI 1.29 – 2.43). There were 57 implants with ≥500 procedures at risk. Thirty implants were classified as inferior to the reference by at least 20% relative risk of failure. Eight of the 30 were shown to be inferior by at least 100% relative risk (supplementary figure 3). All of the unicondylar implants with ≥500 procedures at risk (n=5) were inferior by at least 100% relative risk. No implant could be described as non-inferior.

The reference implant at 10 years was identified as the TKR PFC Sigma Bicondylar Knee cemented unconstrained TKR with a fixed bearing. There were 19,284 procedures remaining at risk and the failure rate was 2.37% (95%CI 2.27 – 2.47). There were 27 implants with ≥500 procedures at risk. Eighteen implants were classified as inferior to the reference by at least 20% relative risk of failure. Two of the 18 were shown to be inferior by at least 100% relative risk (figure 3). There were only 2 unicondylar knee replacements with ≥500 procedures at risk at 10 years, both of which were inferior to the reference by at least 100% relative risk. Two implants were identified as non-inferior, the NexGen cemented unconstrained TKR with fixed bearing and the TKR PFC Sigma Bicondylar Knee hybrid uncemented with a fixed bearing.

Estimates for the difference in failure between the reference and comparator implants with ≥250 procedure at risk at the time of interest are shown in supplementary tables 1-4.

For peer review only - http://bmjopen.bmj.com/site/about/guidelines.xhtml

Non-inferiority - Gender specific

Gender specific non-inferiority analyses were also performed at 3, 5, 7, and 10 years after the primary operation.

At 3 years there were no implants that demonstrated non-inferiority in comparison to the reference prosthesis. Most striking is the general similarity in implants used and their performance regardless of gender. There were 56 different implants with at least 500 procedures still at risk in females versus 50 in males. There were 25 implants with at least a 20% increase in relative risk in females versus 18 in males although the increased number of inferior implants demonstrated in females is likely owing to the slightly better performing reference group than in males. Three implants were inferior by at least 100% in males, each a UKR, and eight implants were inferior by at least 100% in females (6 UKRs and 2 TKRs).

At 5 years, in females, there were 49 implants with ≥500 procedures at risk. Twenty-nine of these implants were classified as inferior to the reference by at least 20% relative risk of failure. Six of the 29 were shown to be inferior by at least 100% relative risk, four of which were unicondylar replacements. Similarly, in males there were 43 implants used with 19 inferior by at least a 20% increase in relative risk. Four of the 19 were inferior by at least 100% relative risk, all of which were unicondylar replacements. Results for males and females at 5 years with ≥250 procedures at risk can be seen in tables 1 and 2 respectively.

For peer review only - http://bmjopen.bmj.com/site/about/guidelines.xhtml

Table 1: Difference in cumulative percentage revision of knee implants compared to a contemporary benchmark at 5 years post primary in men for knee replacements with ≥250 procedures remaining at risk

Knee brand, bearing and constraint	Number at risk	Cumulative failure (%)	Difference in failure (%)	95% CI	Equivalence status	p-value
[TKR] NexGen Cement, unconstrained fixed	8,639	1.76	[REFERENCE]			
[TKR] AGC Cement, unconstrained fixed	16,871	2.28	0.52	[0.24, 0.80]	Non-inferiority not shown	<0.001
[TKR] AGC Hybrid, unconstrained fixed	369	2.59	0.83	[-0.70, 2.36]	Non-inferiority not shown	0.143
[TKR] AGC Uncemented, unconstrained fixed	468	4.87	3.11	[1.33, 4.88]	Inferior by ≥20%	<0.001
[TKR] Advance MP Cement, unconstrained fixed	1,725	3.18	1.42	[0.74, 2.11]	Inferior by ≥20%	<0.001
[TKR] Advance PS Cement, posterior-stabilised fixed	266	3.48	1.72	[-0.11, 3.55]	Non-inferiority not shown	0.033
[TKR] Columbus Cement, unconstrained fixed	1,224	2.49	0.73	[0.08, 1.38]	Non-inferiority not shown	0.013
[TKR] E-Motion Bicondylar Knee Uncemented, unconstrained mobile	519	2.49	0.73	[-0.41, 1.88]	Non-inferiority not shown	0.105
[TKR] FS Cement, unconstrained fixed	305	1.76	0.00	[-1.41, 1.40]	Non-inferiority not shown	0.497
[TKR] Genesis 2 Cement, posterior-stabilised fixed	1,723	2.97	1.21	[0.61, 1.82]	Inferior by ≥20%	<0.001
[TKR] Genesis 2 Cement, unconstrained fixed	6,545	2.17	0.41	[0.08, 0.74]	Non-inferiority not shown	0.008
[TKR] Genesis 2 Cement, unconstrained mobile	310	3.52	1.76	[-0.13, 3.65]	Non-inferiority not shown	0.034
[TKR] Genesis 2 Oxinium Cement, posterior-stabilised fixed	411	5.27	3.51	[1.89, 5.13]	Inferior by ≥100%	<0.001
[TKR] Genesis 2 Oxinium Cement, unconstrained fixed	1,175	3.42	1.65	[0.76, 2.55]	Inferior by ≥20%	<0.001
[TKR] Insall-Burstein 2 Cement, posterior-stabilised fixed	892	2.88	1.12	[0.06, 2.17]	Non-inferiority not shown	0.019
[TKR] Kinemax Cement, unconstrained fixed	3,927	3.08	1.32	[0.76, 1.87]	Inferior by ≥20%	<0.001
[TKR] LCS Complete Cement, unconstrained mobile	2,774	2.88	1.12	[0.54, 1.70]	Inferior by ≥20%	<0.001
[TKR] LCS Complete Uncemented, unconstrained mobile	3,472	2.36	0.60	[0.14, 1.06]	Non-inferiority not shown	0.005
[TKR] LCS Uncemented, unconstrained mobile	485	2.82	1.06	[-0.36, 2.48]	Non-inferiority not shown	0.072
[TKR] MRK Cement, unconstrained fixed	1,681	2.07	0.31	[-0.26, 0.87]	Non-inferiority not shown	0.143
[TKR] Maxim Cement, unconstrained fixed	481	1.86	0.10	[-1.06, 1.25]	Non-inferiority not shown	0.436
[TKR] NRG Cement, posterior-stabilised fixed	1,291	2.62	0.86	[0.10, 1.62]	Non-inferiority not shown	0.014
[TKR] NRG Cement, unconstrained fixed	1,649	3.14	1.38	[0.67, 2.09]	Inferior by ≥20%	<0.001
[TKR] Natural Knee II Cement, unconstrained fixed	767	2.02	0.26	[-0.65, 1.16]	Non-inferiority not shown	0.288
[TKR] NexGen Cement, posterior-stabilised mobile	267	3.93	2.17	[0.20, 4.14]	Non-inferiority not shown	0.016
[TKR] NexGen Cement, posterior-stabilised fixed	11,354	2.89	1.13	[0.81, 1.45]	Inferior by ≥20%	<0.001
[TKR] NexGen Hybrid, unconstrained fixed	379	1.47	-0.29	[-1.39, 0.81]	Non-inferiority not shown	0.303
[TKR] NexGen Uncemented, posterior-stabilised fixed	568	1.81	0.05	[-0.87, 0.97]	Non-inferiority not shown	0.456
[TKR] NexGen Uncemented, unconstrained fixed	1,639	3.13	1.36	[0.59, 2.14]	Inferior by ≥20%	<0.001
[TKR] Optetrak Cement, posterior-stabilised fixed	533	3.51	1.75	[0.29, 3.20]	Non-inferiority not shown	0.009
[TKR] PFC Sigma Bicondylar Knee Cement, posterior-stabilised mobile	1,976	2.65	0.89	[0.27, 1.51]	Non-inferiority not shown	0.002
[TKR] PFC Sigma Bicondylar Knee Cement, posterior-stabilised fixed	15,828	2.16	0.40	[0.13, 0.67]	Non-inferiority not shown	0.002
[TKR] PFC Sigma Bicondylar Knee Cement, unconstrained fixed	36,856	1.83	0.07	[-0.16, 0.30]	Non-inferior	0.279
[TKR] PFC Sigma Bicondylar Knee Cement, unconstrained mobile	2,565	2.75	0.98	[0.40, 1.57]	Inferior by ≥20%	0.001
[TKR] PFC Sigma Bicondylar Knee Hybrid, unconstrained fixed	619	1.86	0.10	[-0.93, 1.12]	Non-inferiority not shown	0.428
[TKR] PFC Sigma Bicondylar Knee monobloc polyethylene tibia	898	1.69	-0.07	[-0.61, 0.46]	Non-inferiority not shown	0.394

For peer review only

For peer review only - http://bmjopen.bmj.com/site/about/guidelines.xhtml

[TKR] Profix Cement, unconstrained fixed	360	3.97	2.21	[0.29, 4.12]	Non-inferiority not shown	0.012
[TKR] Profix Uncemented, unconstrained fixed	856	1.21	-0.55	[-1.26, 0.16]	Non-inferior	0.064
[TKR] Rotaglide + Cement, unconstrained mobile	610	4.40	2.64	[1.11, 4.17]	Inferior by ≥20%	<0.001
[TKR] Rotaglide Cement, unconstrained mobile	291	4.30	2.54	[0.62, 4.45]	Inferior by ≥20%	0.005
[TKR] Scorpio Cement, posterior-stabilised mobile	517	2.39	0.63	[-0.62, 1.89]	Non-inferiority not shown	0.162
[TKR] Scorpio Cement, posterior-stabilised fixed	2,090	2.91	1.14	[0.43, 1.86]	Inferior by ≥20%	0.001
[TKR] Scorpio Cement, unconstrained fixed	3,667	3.06	1.30	[0.75, 1.86]	Inferior by ≥20%	<0.001
[TKR] Scorpio Cement, unconstrained mobile	436	3.94	2.18	[0.43, 3.93]	Inferior by ≥20%	0.007
[TKR] Scorpio Hybrid, unconstrained fixed	374	3.33	1.57	[-0.16, 3.30]	Non-inferiority not shown	0.038
[TKR] Scorpio Uncemented, unconstrained fixed	1,440	2.50	0.74	[-0.04, 1.51]	Non-inferiority not shown	0.031
[TKR] TC Plus Cement, unconstrained fixed	3,133	3.00	1.23	[0.64, 1.83]	Inferior by ≥20%	<0.001
[TKR] TC Plus Cement, unconstrained mobile	1,758	2.61	0.84	[0.13, 1.56]	Non-inferiority not shown	0.010
[TKR] TC Plus Uncemented, unconstrained mobile	434	1.80	0.04	[-1.00, 1.07]	Non-inferiority not shown	0.473
[TKR] Triathlon Cement, posterior-stabilised fixed	1,851	2.53	0.77	[0.23, 1.31]	Non-inferiority not shown	0.003
[TKR] Triathlon Cement, unconstrained fixed	5,995	1.94	0.18	[-0.13, 0.48]	Non-inferiority not shown	0.126
[TKR] Vanguard Cement, posterior-stabilised fixed	471	2.57	0.81	[-0.05, 1.67]	Non-inferiority not shown	0.033
[TKR] Vanguard Cement, unconstrained fixed	3,489	2.10	0.34	[-0.01, 0.70]	Non-inferiority not shown	0.028
[UNI] AMC/Uniglide Unicondylar, fixed	348	3.97	2.21	[0.51, 3.90]	Inferior by ≥20%	0.005
[UNI] AMC/Uniglide Unicondylar, mobile	519	8.90	7.14	[5.06, 9.22]	Inferior by ≥100%	<0.001
[UNI] MG Uni Unicondylar, fixed	1,130	5.36	3.60	[2.33, 4.86]	Inferior by ≥100%	<0.001
[UNI] Oxford Partial Knee Unicondylar, mobile	14,581	5.72	3.96	[3.59, 4.33]	Inferior by ≥100%	<0.001
[UNI] Physica ZUK Unicondylar, fixed	1,280	3.88	2.12	[1.35, 2.89]	Inferior by ≥20%	<0.001
[UNI] Preservation Unicondylar, fixed	566	8.55	6.79	[4.61, 8.96]	Inferior by ≥100%	<0.001
[UNI] Sigma HP Unicondylar, fixed	948	4.36	2.59	[1.75, 3.44]	Inferior by ≥20%	<0.001
[UNI] Sled Unicondylar, fixed	261	11.47	9.71	[6.30, 13.12]	Inferior by ≥100%	<0.001

Note: TKR = total knee replacement, UNI = Unicondylar knee replacement

For peer review only

For peer review only - http://bmjopen.bmj.com/site/about/guidelines.xhtml

Table 2: Difference in cumulative percentage revision of knee implants compared to a contemporary benchmark at 5 years post primary in women for knee replacements with ≥250 procedures remaining at risk

Knee brand, bearing and constraint	Number at risk	Cumulative failure (%)	Difference in failure (%)	95% CI	Equivalence status	p-value
[TKR] MRK Cement, unconstrained fixed	2,570	1.35	[REFERENCE]			
[TKR] AGC Cement, posterior-stabilised fixed	264	2.65	1.31	[-0.54, 3.16]	Non-inferiority not shown	0.083
[TKR] AGC Cement, unconstrained fixed	23,829	1.85	0.50	[0.13, 0.88]	Non-inferiority not shown	0.004
[TKR] AGC Hybrid, unconstrained fixed	431	3.11	1.76	[0.17, 3.35]	Non-inferiority not shown	0.015
[TKR] AGC Uncemented, unconstrained fixed	502	5.34	4.00	[2.10, 5.89]	Inferior by ≥100%	<0.001
[TKR] Advance MP Cement, unconstrained fixed	2,264	2.46	1.11	[0.48, 1.75]	Inferior by ≥20%	<0.001
[TKR] Advance MP Stature Cement, unconstrained fixed	378	2.93	1.58	[0.31, 2.86]	Inferior by ≥20%	0.007
[TKR] Advance PS Cement, posterior-stabilised fixed	354	2.97	1.62	[0.09, 3.15]	Non-inferiority not shown	0.019
[TKR] Columbus Cement, unconstrained fixed	1,595	2.53	1.19	[0.54, 1.83]	Inferior by ≥20%	<0.001
[TKR] E-Motion Bicondylar Knee Uncemented, unconstrained mobile	544	3.22	1.87	[0.57, 3.17]	Inferior by ≥20%	0.002
[TKR] FS Cement, unconstrained fixed	397	2.34	0.99	[-0.48, 2.47]	Non-inferiority not shown	0.093
[TKR] Genesis 2 Cement, posterior-stabilised fixed	2,848	2.15	0.80	[0.29, 1.32]	Inferior by ≥20%	0.001
[TKR] Genesis 2 Cement, unconstrained fixed	9,021	1.44	0.10	[-0.29, 0.49]	Non-inferiority not shown	0.311
[TKR] Genesis 2 Cement, unconstrained mobile	464	1.48	0.14	[-0.94, 1.22]	Non-inferiority not shown	0.401
[TKR] Genesis 2 Oxinium Cement, posterior-stabilised fixed	620	3.49	2.14	[1.00, 3.29]	Inferior by ≥20%	<0.001
[TKR] Genesis 2 Oxinium Cement, unconstrained fixed	1,485	2.75	1.41	[0.63, 2.19]	Inferior by ≥20%	<0.001
[TKR] Insall-Burstein 2 Cement, posterior-stabilised fixed	1,177	2.63	1.28	[0.33, 2.23]	Inferior by ≥20%	0.004
[TKR] Journey Oxinium Cement, posterior-stabilised fixed	283	7.30	5.96	[3.45, 8.47]	Inferior by ≥100%	<0.001
[TKR] Kinemax Cement, unconstrained fixed	5,369	2.41	1.07	[0.54, 1.59]	Inferior by ≥20%	<0.001
[TKR] LCS Cement, unconstrained mobile	340	1.85	0.51	[-0.90, 1.91]	Non-inferiority not shown	0.239
[TKR] LCS Complete Cement, unconstrained mobile	3,759	2.62	1.28	[0.72, 1.84]	Inferior by ≥20%	<0.001
[TKR] LCS Complete Uncemented, unconstrained mobile	4,205	2.61	1.26	[0.73, 1.80]	Inferior by ≥20%	<0.001
[TKR] LCS Uncemented, unconstrained mobile	675	2.16	0.82	[-0.29, 1.92]	Non-inferiority not shown	0.073
[TKR] Maxim Cement, posterior-stabilised fixed	282	3.24	1.90	[-0.02, 3.82]	Non-inferiority not shown	0.026
[TKR] Maxim Cement, unconstrained fixed	629	2.34	1.00	[-0.16, 2.15]	Non-inferiority not shown	0.045
[TKR] NRG Cement, posterior-stabilised fixed	1,784	2.24	0.90	[0.22, 1.58]	Non-inferiority not shown	0.005
[TKR] NRG Cement, unconstrained fixed	2,350	1.79	0.44	[-0.11, 1.00]	Non-inferiority not shown	0.059
[TKR] Natural Knee II Cement, unconstrained fixed	1,183	2.34	1.00	[0.15, 1.84]	Non-inferiority not shown	0.010
[TKR] NexGen Cement, posterior-stabilised mobile	414	3.39	2.04	[0.49, 3.60]	Inferior by ≥20%	0.005
[TKR] NexGen Cement, posterior-stabilised fixed	16,989	2.30	0.95	[0.56, 1.34]	Inferior by ≥20%	<0.001
[TKR] NexGen Cement, unconstrained fixed	12,603	1.47	0.13	[-0.25, 0.51]	Non-inferiority not shown	0.253
[TKR] NexGen Hybrid, unconstrained fixed	454	1.72	0.37	[-0.79, 1.54]	Non-inferiority not shown	0.266
[TKR] NexGen Uncemented, posterior-stabilised fixed	514	2.66	1.31	[0.10, 2.52]	Non-inferiority not shown	0.017
[TKR] NexGen Uncemented, unconstrained fixed	1,421	3.25	1.90	[0.98, 2.83]	Inferior by ≥20%	<0.001
[TKR] Optetrak Cement, posterior-stabilised fixed	746	5.29	3.94	[2.45, 5.44]	Inferior by ≥100%	<0.001

For peer review only

[TKR] PFC Sigma Bicondylar Knee Cement, posterior-stabilised mobile	2,280	2.92	1.57	[0.90, 2.24]	Inferior by ≥20%	<0.001
[TKR] PFC Sigma Bicondylar Knee Cement, posterior-stabilised fixed	23,690	2.03	0.69	[0.31, 1.06]	Inferior by ≥20%	<0.001
[TKR] PFC Sigma Bicondylar Knee Cement, unconstrained fixed	51,762	1.61	0.27	[-0.09, 0.63]	Non-inferiority not shown	0.071
[TKR] PFC Sigma Bicondylar Knee Cement, unconstrained mobile	2,852	2.72	1.37	[0.73, 2.01]	Inferior by ≥20%	<0.001
[TKR] PFC Sigma Bicondylar Knee Hybrid, unconstrained fixed	771	1.75	0.40	[-0.54, 1.34]	Non-inferiority not shown	0.202
[TKR] PFC Sigma Bicondylar Knee Uncemented, unconstrained mobile	254	1.60	0.25	[-1.07, 1.57]	Non-inferiority not shown	0.355
[TKR] PFC Sigma Bicondylar Knee monobloc polyethylene tibia	1,486	1.61	0.27	[-0.27, 0.80]	Non-inferiority not shown	0.163
[TKR] Profix Cement, unconstrained fixed	454	2.58	1.23	[-0.19, 2.66]	Non-inferiority not shown	0.045
[TKR] Profix Oxinium monobloc polyethylene tibia	317	3.58	2.24	[0.22, 4.26]	Non-inferiority not shown	0.015
[TKR] Profix Uncemented, unconstrained fixed	1,055	1.81	0.47	[-0.36, 1.29]	Non-inferiority not shown	0.134
[TKR] Profix monobloc polyethylene tibia	371	1.47	0.13	[-1.09, 1.34]	Non-inferiority not shown	0.420
[TKR] Rotaglide + Cement, unconstrained mobile	845	3.08	1.74	[0.58, 2.89]	Inferior by ≥20%	0.002
[TKR] Rotaglide Cement, unconstrained mobile	487	2.65	1.31	[0.04, 2.58]	Non-inferiority not shown	0.022
[TKR] Scorpio Cement, posterior-stabilised mobile	662	2.03	0.68	[-0.39, 1.76]	Non-inferiority not shown	0.106
[TKR] Scorpio Cement, posterior-stabilised fixed	3,175	2.01	0.67	[0.09, 1.25]	Non-inferiority not shown	0.012
[TKR] Scorpio Cement, unconstrained fixed	5,380	2.29	0.94	[0.43, 1.45]	Inferior by ≥20%	<0.001
[TKR] Scorpio Cement, unconstrained mobile	601	3.39	2.05	[0.61, 3.49]	Inferior by ≥20%	0.003
[TKR] Scorpio Hybrid, unconstrained fixed	565	1.95	0.60	[-0.54, 1.75]	Non-inferiority not shown	0.152
[TKR] Scorpio Uncemented, unconstrained fixed	1,702	2.48	1.14	[0.36, 1.91]	Inferior by ≥20%	0.002
[TKR] TC Plus Cement, unconstrained fixed	3,820	2.19	0.84	[0.28, 1.40]	Inferior by ≥20%	0.002
[TKR] TC Plus Cement, unconstrained mobile	2,242	1.52	0.18	[-0.41, 0.76]	Non-inferiority not shown	0.279
[TKR] TC Plus Uncemented, unconstrained mobile	712	2.40	1.06	[0.02, 2.09]	Non-inferiority not shown	0.023
[TKR] Triathlon Cement, posterior-stabilised fixed	2,754	2.52	1.18	[0.63, 1.72]	Inferior by ≥20%	<0.001
[TKR] Triathlon Cement, unconstrained fixed	8,378	2.07	0.72	[0.32, 1.12]	Inferior by ≥20%	<0.001
[TKR] Triathlon Uncemented, unconstrained fixed	257	3.51	2.17	[0.50, 3.84]	Inferior by ≥20%	0.005
[TKR] Vanguard Cement, posterior-stabilised fixed	665	2.87	1.53	[0.71, 2.35]	Inferior by ≥20%	<0.001
[TKR] Vanguard Cement, unconstrained fixed	4,840	1.96	0.61	[0.19, 1.04]	Non-inferiority not shown	0.002
[UNI] AMC/Uniglide Unicondylar, fixed	409	4.80	3.45	[1.70, 5.21]	Inferior by ≥100%	<0.001
[UNI] AMC/Uniglide Unicondylar, mobile	479	11.54	10.19	[7.69, 12.69]	Inferior by ≥100%	<0.001
[UNI] MG Uni Unicondylar, fixed	953	6.66	5.32	[3.77, 6.87]	Inferior by ≥100%	<0.001
[UNI] Oxford Partial Knee Unicondylar, mobile	13,555	6.58	5.23	[4.74, 5.72]	Inferior by ≥100%	<0.001
[UNI] Physica ZUK Unicondylar, fixed	1,113	4.02	2.67	[1.80, 3.54]	Inferior by ≥100%	<0.001
[UNI] Preservation Unicondylar, fixed	480	12.12	10.78	[8.04, 13.52]	Inferior by ≥100%	<0.001
[UNI] Sigma HP Unicondylar, fixed	688	5.89	4.55	[3.35, 5.75]	Inferior by ≥100%	<0.001
[UNI] Sled Unicondylar, fixed	325	8.48	7.13	[4.41, 9.85]	Inferior by ≥100%	<0.001

Note: TKR = total knee replacement, UNI = Unicondylar knee replacement

For peer review only - http://bmjopen.bmj.com/site/about/guidelines.xhtml

At 7 years the results were again similar between male and females. There were 40 different implants used in females and 36 in males. In females there were 19 implants that were inferior by at least 20% relative risk, 2 of which were inferior by at least 100%. These were both UKRs. In males 18 of the 36 implants with ≥500 procedures still at risk were deemed to be inferior to the reference by at least 20% relative risk. Three of these 18 were inferior by at least 100% relative risk, all of which were UKRs. Two implants were demonstrated to be non-inferior to the reference.

At 10 years, in both males and females there was only one brand of UKR that had at least 500 procedures still at risk. In both instances this was inferior by at least 100% relative risk and was the only implant to be classified as such. There were no brands found to be non-inferior to the reference in either males or females. In females there were 8 of 15 implants that were inferior to the reference by at least 20% relative risk. In males 8 out of 13 implants that were inferior to the reference by at least 20% relative risk. Data for gender specific stratification can be viewed in supplementary figures 4, 5, 6 and 7 for males at 3, 5, 7 and 10 years respectively and supplementary figures 8, 9, 10 and 11 for females at 3, 5, 7 and 10 years respectively.

Non-inferiority- Gender and Age specific

Sub-dividing procedures by age and gender highlights the paucity of information available for male or female patients <55 years compared to those ≥55 years. There is little data at 7 years and no suitable reference could be found for females at 10 years in the <55 age group. There is a similar mix of implant types in each of the age groups in both males and females with cemented unconstrained total knee replacements with a fixed bearing the most popular type. Data for males under 55 years of age at 3, 5 and 7 years can be viewed in supplementary figures 12, 13 and 14 respectively. Data for females under 55 years of age at 3, 5 and 7 years can be viewed in supplementary figures 15, 16 and 17 respectively.

For peer review only - http://bmjopen.bmj.com/site/about/guidelines.xhtml

For peer review only

At 7 years in females aged 55-75 there were 32 different implants that met the threshold of 500 cases for analysis with 8 being classified as inferior by at least 20% relative risk (Figure 4). Two of these were inferior by at least 100% relative risk and both were UKRs. No implants were demonstrated to be non-inferior to the reference. In males there were 27 implants meeting the threshold with 12 being classified as inferior by at least 20% relative risk (Figure 5). A single prosthesis, 1 of the 2 UKRs with at least 500 procedures still at risk, was classified as being inferior by at least 100% relative risk. One implant was found to be non-inferior to the reference. Data for males between 55-75 years of age at 3, 5 and 10 years post primary can be viewed in supplementary figures 18, 19 and 20 respectively. Data for females between 55-75 years of age at 3, 5 and 10 years post primary can be viewed in supplementary figures 21,22 and 23 respectively.

In the >75 years age group we found only 1 inferior implant in both males and females at 7 years. Non-inferiority was not shown for any other prostheses, but very few met the threshold of 500 cases needed for analysis. At 10 years only 3 implants with ≥500 procedures still at risk were present in males and 5 in females. Non-inferiority was not demonstrated in any of these. At this time point and age group subdivision there were no UKRs with enough procedures still at risk to be included in the analysis. Data for males >75 years of age at 3, 5, 7 and 10 years post primary can be viewed in supplementary figure 24,25,26 and 27 respectively. Data for females >75 years of age at 3, 5, 7 and 10 years post primary can be viewed in supplementary figure 28,29,30 and 31 respectively.

Data for each level of stratification for comparator implants with at least 250 patients at risk at the beginning of the time point of interest can be viewed in the following supplemental tables. Data for all males at 3, 7 and 10 years can be seen in supplemental tables 5, 6 and 7 respectively and for all females at 3, 7 and 10 years in supplemental tables 8, 9 and 10. Data for males <55 years of age at 3, 5 and 7 years can be seen in supplemental tables 11, 12 and 13 respectively and females <55 at 3, 5 and 7 years can be seen in supplemental tables 14, 15 and 16 respectively. Data for males aged between 55 and 75 at 3, 5, 7 and 10 years can be seen in supplemental tables 17, 18, 19 and 20

For peer review only - http://bmjopen.bmj.com/site/about/guidelines.xhtml

respectively, and for females aged between 55 and 75 years at 3, 5, 7 and 10 years data can be seen in supplemental tables 21, 22, 23 and 24 respectively. Data for males aged >75 years at 3, 5, 7 and 10 years post primary can be seen in supplemental tables 25, 26, 27 and 28 respectively, and for females >75 years at 3, 5, 7 and 10 years post primary data can be seen in supplemental tables 29, 30, 31 and 32 respectively.

Sensitivity Analysis

Using the reference implant at 10 years as the reference at 3, 5, and 7 years, illustrates the temporal improvements in failure and the selective trajectory tracking of new implants. The reference implant in all procedures at 10 years is the PFC Sigma Bicondylar Knee cemented unconstrained TKR with a fixed bearing which had a failure rate of 2.37% (95%CI 2.27 – 2.47). At 3, 5 and 7 years, the contemporary reference has a 0.15%, a 0.17% and 0.23% lower point-estimate failure rate than the historical reference respectively. The performance of the implants appears to track in a consistent manner from each time point to the next.

For peer review only

For peer review only - http://bmjopen.bmj.com/site/about/guidelines.xhtml

DISCUSSION

We have demonstrated in 947,686 primary TKRs and UKRs the relative performance of knee implants in comparison to an internally selected relevant contemporary reference. There are two striking findings from the data analysed here. First, UKR brands almost universally have 100% worse revision outcomes in all ages, gender and time points of interest compared to the reference standard brand of knee replacement. Second, very few brands of knee replacement can be proven not to be at least 20% worse than the reference brands despite 449 brand implants having been implanted in England and Wales since 2003. Between one and two thirds of the knee implants for which sufficient data was available at each time point were at least 20% inferior to the contemporary benchmark. The vast majority of implants are implanted in too few cases to allow meaningful analysis. Many of those implanted in sufficient number have here been demonstrated to be inferior in terms of implant survivorship, whilst very few TKR's have been demonstrated to be non-inferior to a contemporary reference.

It is known that TKRs as a class have lower revision rates than UKRs which poses the question, should these two classes be directly compared? Since every patient who received a UKR could have received a TKR instead, this comparison is justified. There is no evidence to suggest that the subsequent observed revision rates would be any different if those receiving a UKR had received a TKR instead. Furthermore, ODEP (currently) do not provide a rating for UKRs so this method provides extra transparency and previously unavailable information for patients undergoing knee replacement.

Comparison with other studies and implications in light of existing evidence

No other published study has performed a direct head-to-head comparison of all available knee replacement implants. The closest available data is from national registry annual reports such as the NJR[1] which reports absolute failure of the commonest implants by age and gender. This shows that low failure rates are achieved by a number of implants, but this does not facilitate easy direct

For peer review only - http://bmjopen.bmj.com/site/about/guidelines.xhtml

comparison between them. Using the data presented here alongside the annual report data will greatly enhance the information available to surgeons, commissioners and to inform patient choice.

Consideration of the difference in outcome of UKR compared to TKR is complex. Using propensity score matching of registry data Liddle et al. showed that UKRs have higher revision rates than TKRs, but lower risks of complications.[12] Hunt et al. and Liddle et al. showed UKRs to be associated with lower early post-operative mortality.[12, 13] Kleeblad et al. performed a systematic review and meta-analysis of 49 cohort studies and found no difference in function as measured by Hospital for Special Surgery Score, Knee Society Score, Oxford Knee Score, Visual Analog Pain Scale and Western Ontario and McMaster Universities Osteoarthritis Index Score, but did show higher revision rates with UKRs.[14] Liddle et al. also showed no clinically important difference in Oxford Knee Scores (1 point difference favouring UKR), but UKR patients were more likely to be highly satisfied 6 months after surgery.[15] A recent study by the same group has shown that UKR is less costly than TKR particularly in older patients, who are less likely to require revision, and when performed by higher volume surgeons.[16] Our data adds to this complex picture by clearly demonstrating the increased risk of revision associated with almost all brands of UKRs at all time points in both genders and all age groups when compared with the best performing TKRs.

The major weakness of all registry studies is selection bias whereby certain implants may be implanted for particular indications and in particular groups with different risks. We have mitigated against this by analysing data by age and gender, the two variables with the greatest association with revision rates. Furthermore, revision thresholds may be lower in certain groups or for certain modes of failure. This study has looked at a single, but extremely important, outcome measure, revision. Other outcomes of interest such as mortality and PROMs need to be considered when making choices about treatment. With over 900,000 subjects this is the largest study of knee replacement published to date and comes from the largest implant registry in the world. Data entry is mandated and data capture is extremely high (over 95%),[17] thus the findings are highly likely to

For peer review only - http://bmjopen.bmj.com/site/about/guidelines.xhtml

be generalisable. The methods used are novel and for the first time allow a meaningfully direct comparison between all available implants.

Conclusions, policy and future research implications

Patients should be actively involved in decision making about their treatment. Here we provide, for the first time, data which allows patients and clinicians to directly compare revision rates associated with the use of different UKR and TKR implants. The information provided should be used to inform patient choice, surgical decision making and commissioning.

For peer review only

For peer review only - http://bmjopen.bmj.com/site/about/guidelines.xhtml

Acknowledgments

We thank the patients and staff of all the hospitals who have contributed data to the National Joint Registry. We are grateful to the Healthcare Quality Improvement Partnership, the National Joint Registry Steering Committee, and staff at the National Joint Registry for facilitating this work. The views expressed represent those of the authors and do not necessarily reflect those of the NHS, the National Institute for Health Research, the Department of Health, the National Joint Registry Steering Committee or Healthcare Quality Improvement Partnership, who do not vouch for how the information is presented.

Funding

Posts of members of the research team are funded by a contract grant from the National Joint Registry for England, Wales, Northern Ireland and the Isle of Man. This study was also supported by the NIHR Biomedical Research Centre at the University Hospitals Bristol NHS Foundation Trust and the University of Bristol. AS is funded by an MRC Strategic Skills Fellowship MR/L01226X/1.

Competing interest statement

All authors have completed the Unified Competing Interest form at www.icmje.org/coi_disclosure.pdf (available on request from the corresponding author) and declare: funding was received for the submitted work as described in the Funding statement; AWB and MRW are co-applicants on a grant from Stryker investigating the outcome of the Triathlon total knee replacement; no other financial relationships with any organisations that might have an interest in the submitted work in the previous three years, no other relationships or activities that could appear to have influenced the submitted work.

Author Contributions

AS, MRW and AWB conceived the study. KD, AS and AWB designed the study. The data were extracted by Northgate (Hemel Hempstead, UK). KD and AS managed and analysed data. MRW, AS, MLP and AWB reviewed the published work. All authors critically inputted into final design of the study, interpreted the data and co-wrote the manuscript. AS is the guarantor and attests on behalf of the authors that the manuscript is a true and transparent reflection of the study without omissions.

Role of the funding source

The sponsor of the study had no role in the study design, data collection, analysis, interpretation, or writing of the final report. KD and AS had full access to all of the data and AS had final responsibility for manuscript submission.

For peer review only

For peer review only - http://bmjopen.bmj.com/site/about/guidelines.xhtml

Ethical approval

Not required for this study. Patient data in the NJR is only available by consent of the patient.

Data sharing

Access to data is available from the National Joint Registry for England and Wales.

For peer review only

For peer review only - http://bmjopen.bmj.com/site/about/guidelines.xhtml

Figures

Figure 1: Schematic representation of inferiority and non-inferiority

Figure 2: Difference in cumulative revision of knee implants compared to a contemporary reference at 5 years, using all total knee and unicondylar replacements with ≥500 procedures remaining at risk.

Figure 3: Difference in cumulative revision of knee implants compared to a contemporary reference at 10 years, using all total knee and unicondylar replacements with ≥500 procedures remaining at risk.

Figure 4: Difference in cumulative revision of knee implants compared to a contemporary reference at 7 years in women aged between 55 and 75 years, using all total knee and unicondylar replacements with ≥500 procedures remaining at risk.

Figure 5: Difference in cumulative revision of knee implants compared to a contemporary reference at 7 years in men aged between 55 and 75 years, using all total knee and unicondylar replacements with ≥500 procedures remaining at risk.

For peer review only

REFERENCES

1. National Joint Registry for England W, Northern Ireland and the Isle of Man. 14th Annual report.http://www.njrreports.org.uk/Portals/0/PDFdownloads/NJR%2014th%20Annual%20Report%202017.pdf
2. Tucker K. Orthopaedic Data Evaluation Panel: ODEP. Northgate Public Services (UK) Ltd. 2017 [Available from: http://www.odep.org.uk/ODEPProcess/ODEPStatement.aspx.
3. Nederlandse Orthopaedische Vereniging. Classification Orthopaedic Implants: NOV Total Knee Prosthesis 2016-2017 [Available from: https://www.orthopeden.org/downloads/146/classificatie-knie-2017.pdf.
4. Prostheses and Devices Committee (PDC) and Prostheses List Advisory Committee (PLAC) Bulletins. Australian Government; Department of Health [Available from: http://www.health.gov.au/internet/main/publishing.nsf/Content/health-privatehealth-prostheseslist.htm.
5. Sayers A, Crowther MJ, Judge A, et al. Determining the sample size required to establish whether a medical device is non-inferior to an external benchmark. *BMJ Open* 2017;7(8):e015397. doi: 10.1136/bmjopen-2016-015397 [published Online First: 2017/08/31]
6. Schumi J, Wittes JT. Through the looking glass: understanding non-inferiority. *Trials* 2011;12:106. doi: 10.1186/1745-6215-12-106
7. Tunes da Silva G, Logan BR, Klein JP. Methods for equivalence and noninferiority testing. *Biol Blood Marrow Transplant* 2009;15(1 Suppl):120-7. doi: 10.1016/j.bbmt.2008.10.004
8. Com-Nougue C, Rodary C, Patte C. How to establish equivalence when data are censored: a randomized trial of treatments for B non-Hodgkin lymphoma. *Stat Med* 1993;12(14):1353-64.
9. Sayers A, Evans JT, Whitehouse MR, et al. Are competing risks models appropriate to describe implant failure? *Acta Orthop* 2018;89(3):256-58. doi: 10.1080/17453674.2018.1444876 [published Online First: 2018/03/10]
10. Greenwood M. The natural duration of cancer. *Rep* ***Public Health*** *Med Subj (Lond)* 1926;33: 1-26
11. Donken R, de Melker HE, Rots NY, et al. Comparing vaccines: a systematic review of the use of the non-inferiority margin in vaccine trials. *Vaccine* 2015;33(12):1426-32. doi: 10.1016/j.vaccine.2015.01.072
12. Liddle AD, Judge A, Pandit H, et al. Adverse outcomes after total and unicompartmental knee replacement in 101,330 matched patients: a study of data from the National Joint Registry for England and Wales. *Lancet* 2014;384(9952):1437-45. doi: 10.1016/S0140-6736(14)60419-0 [published Online First: 2014/07/12]
13. Hunt LP, Ben-Shlomo Y, Clark EM, et al. 45-day mortality after 467,779 knee replacements for osteoarthritis from the National Joint Registry for England and Wales: an observational study. *Lancet* 2014;384(9952):1429-36. doi: 10.1016/S0140-6736(14)60540-7 [published Online First: 2014/07/12]
14. Kleeblad LJ, van der List JP, Zuiderbaan HA, et al. Larger range of motion and increased return to activity, but higher revision rates following unicompartmental versus total knee arthroplasty in patients under 65: a systematic review. *Knee Surg Sports Traumatol Arthrosc* 2017 doi: 10.1007/s00167-017-4817-y [published Online First: 2017/12/01]
15. Liddle AD, Pandit H, Judge A, et al. Patient-reported outcomes after total and unicompartmental knee arthroplasty: a study of 14,076 matched patients from the National Joint Registry for England and Wales. *Bone Joint J* 2015;97-B(6):793-801. doi: 10.1302/0301-620X.97B6.35155 [published Online First: 2015/06/03]
16. Burn E, Liddle AD, Hamilton TW, et al. Cost-effectiveness of unicompartmental compared with total knee replacement: a population-based study using data from the National Joint Registry for England and Wales. *BMJ Open* 2018;8(4):e020977. doi: 10.1136/bmjopen-2017-020977 [published Online First: 2018/05/01]

For peer review only - http://bmjopen.bmj.com/site/about/guidelines.xhtml

17. NJR Data Quality Audit 2017 [Available from: http://www.njrcentre.org.uk/njrcentre/NewsandEvents/DataQualityAudit-updateSeptember2017/tabid/1451/Default.aspx.

For peer review only

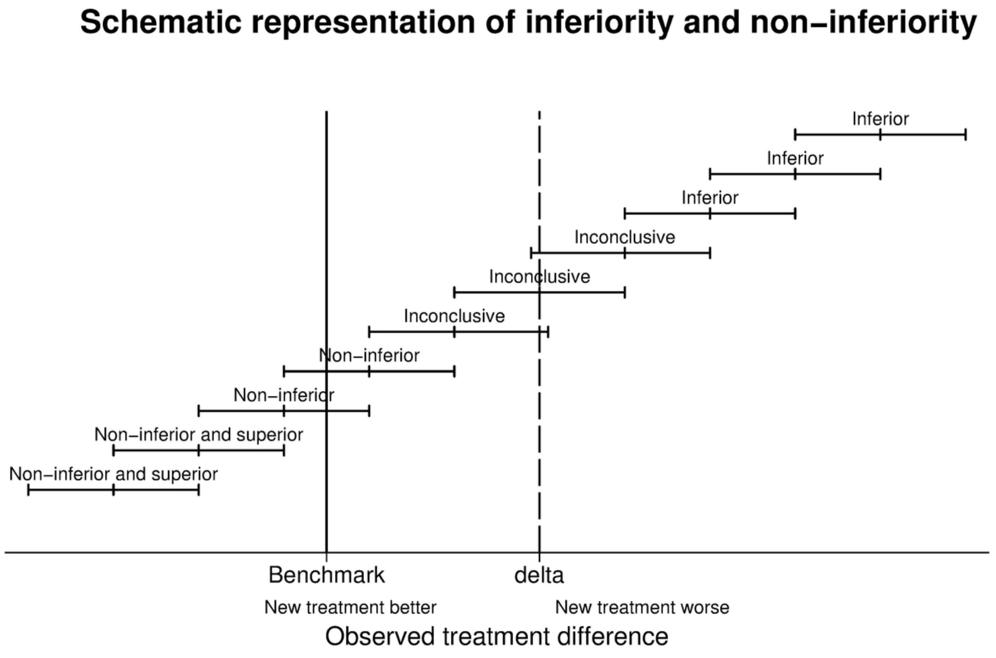

101x67mm (300 x 300 DPI)

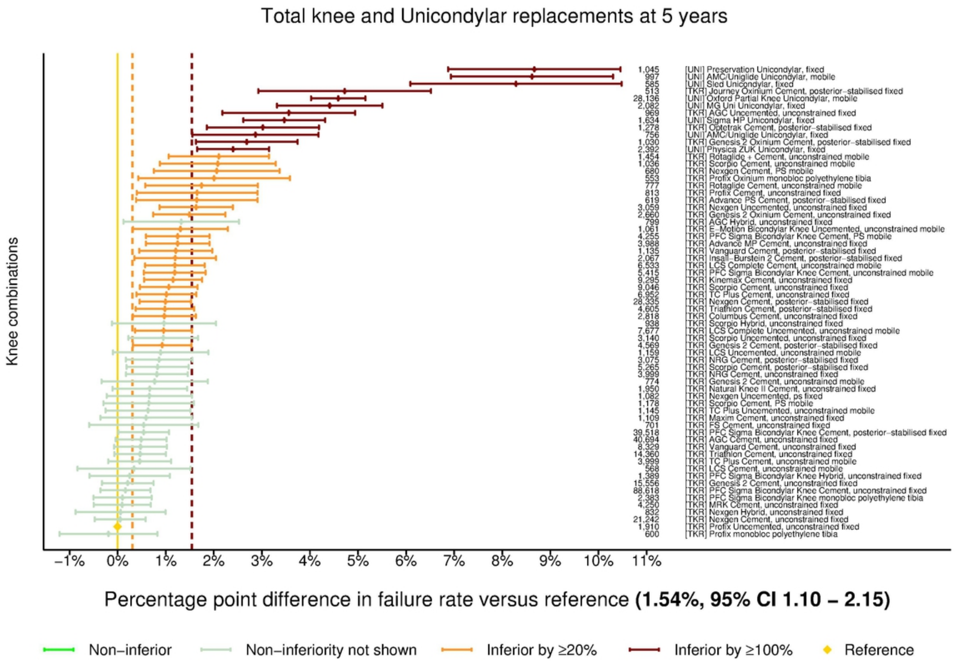

101x73mm (300 x 300 DPI)

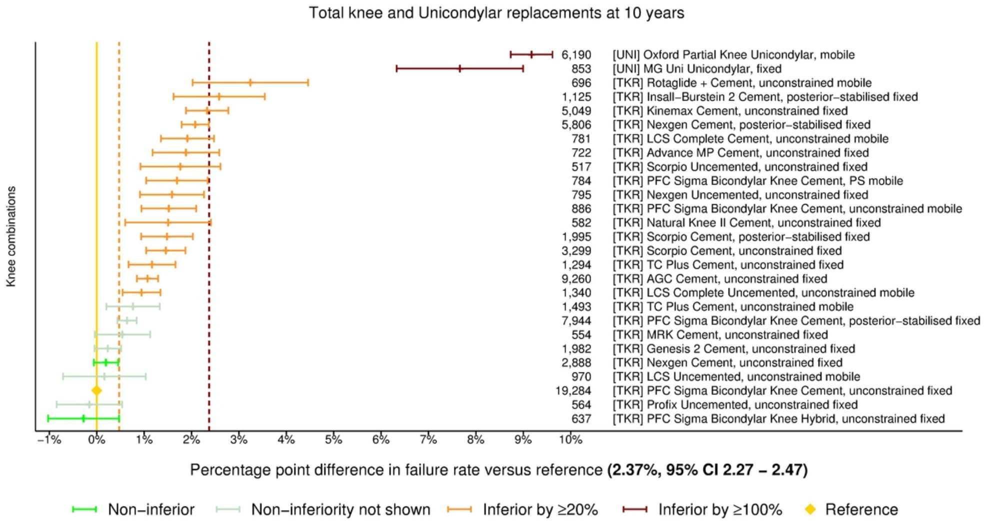

101x58mm (300 x 300 DPI)

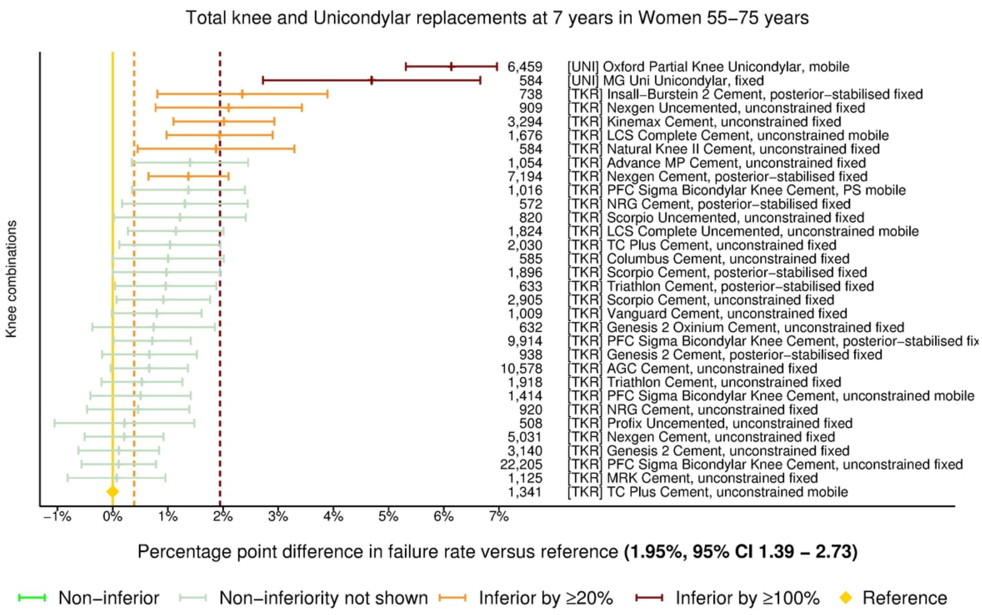

101x67mm (300 x 300 DPI)

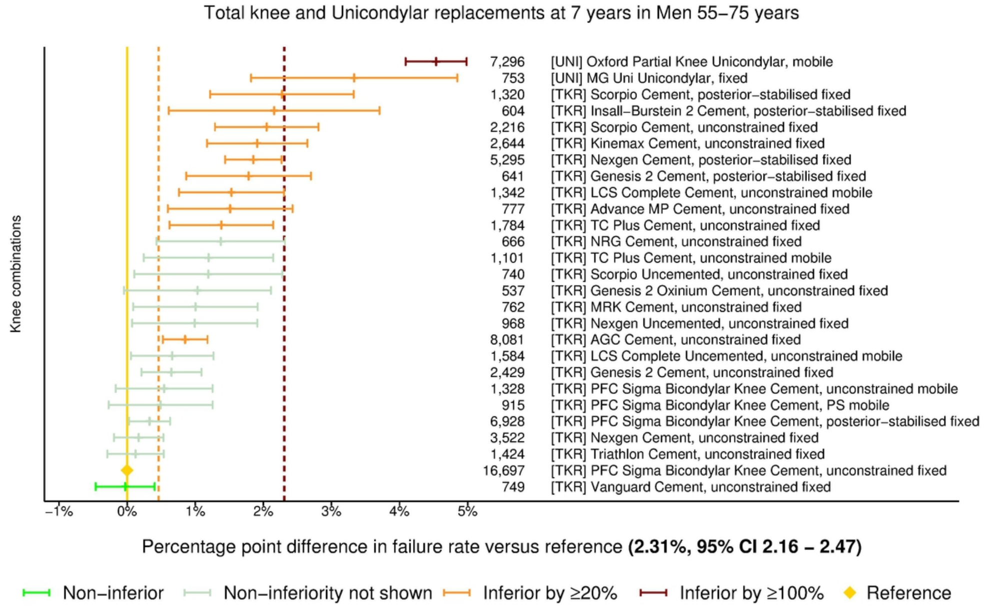

101x67mm (300 x 300 DPI)

Supplementary Figure 1: Flow diagram of showing derivation of procedures used in analyses

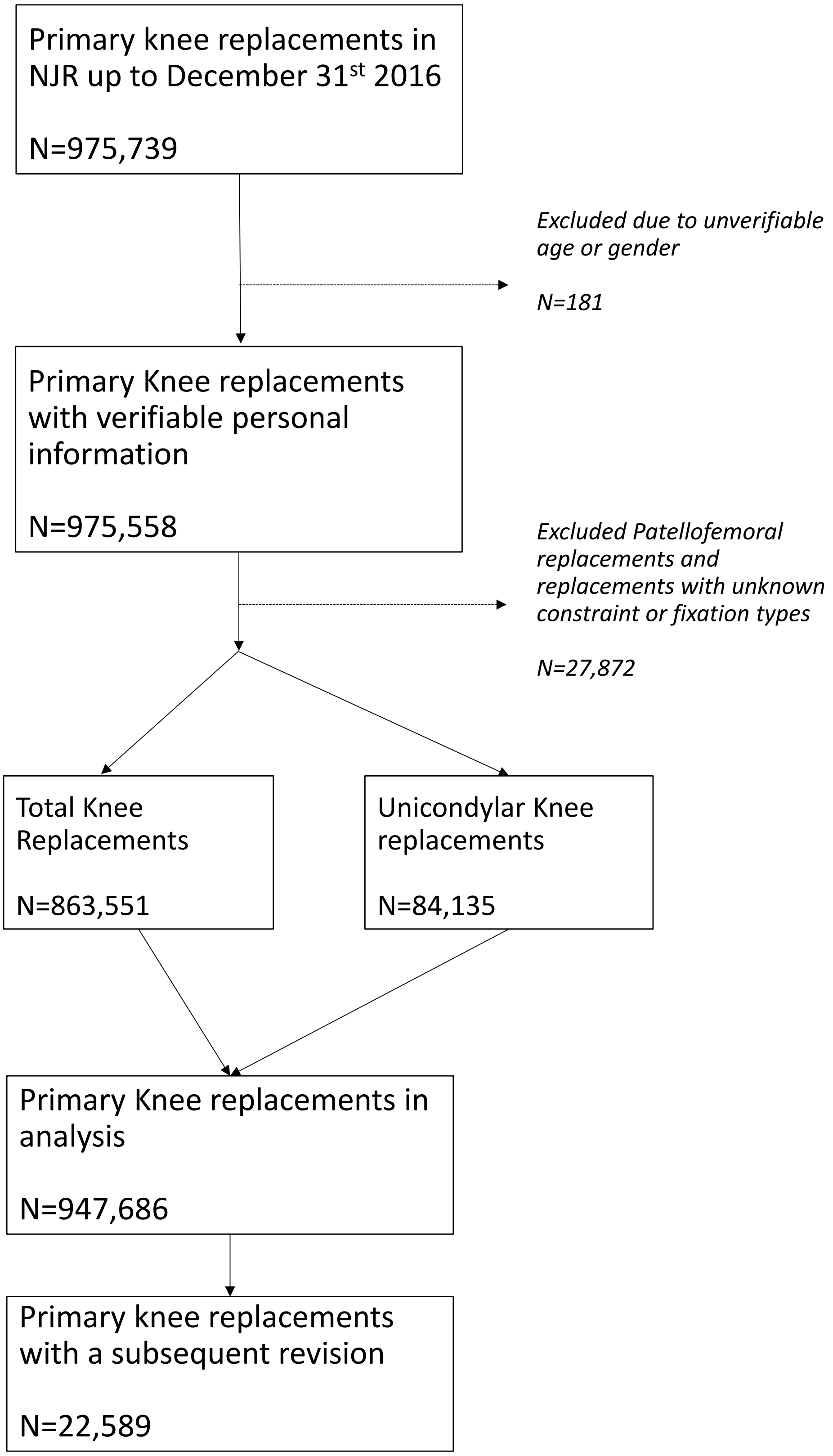

Supplementary Figure 2: Difference in cumulative revision of knee implants compared to a contemporary reference at 3 years, using all total knee and unicondylar replacements with ≥500 procedures remaining at risk.

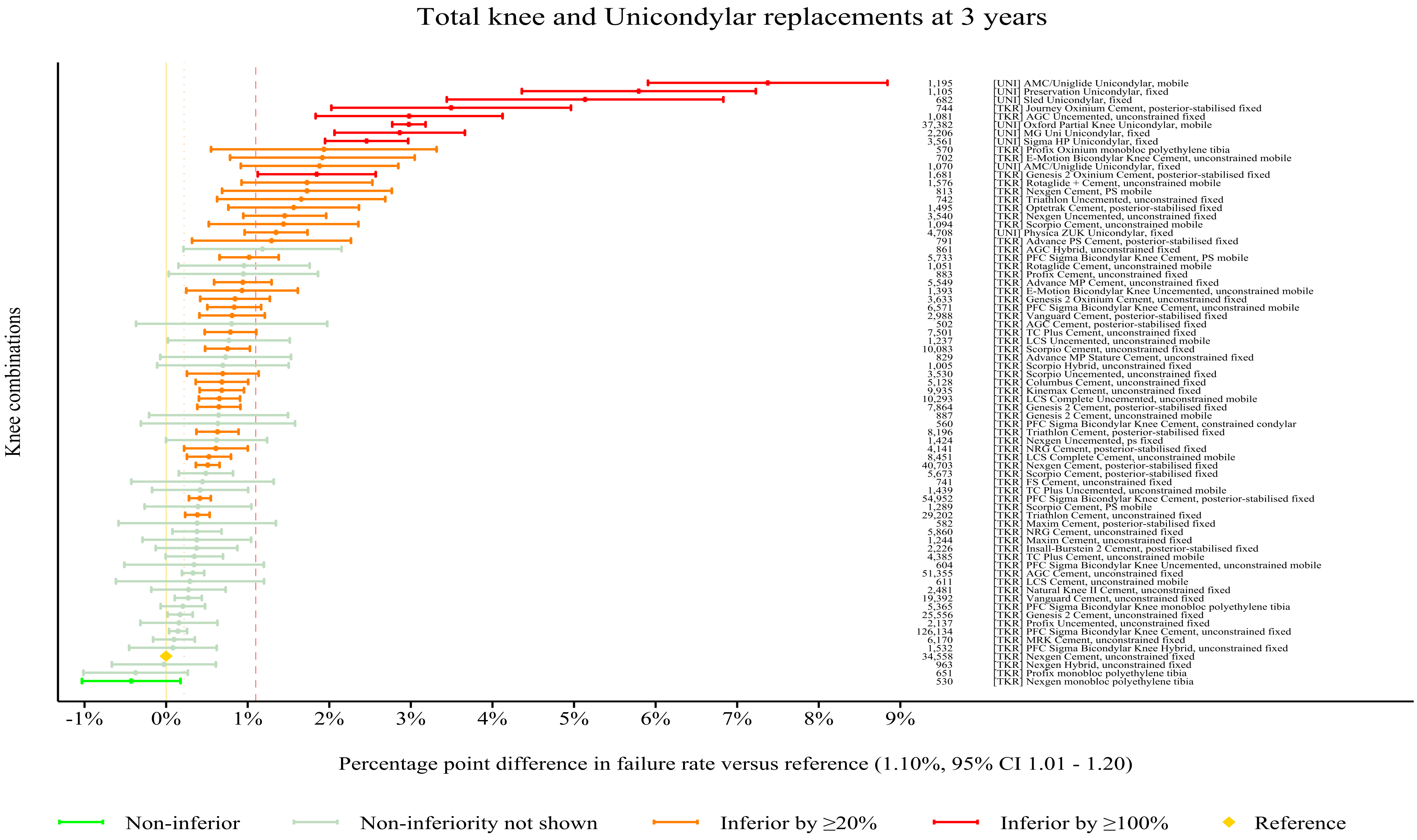

Supplementary Figure 3: Difference in cumulative revision of knee implants compared to a contemporary reference at 7 years, using all total knee and unicondylar replacements with ≥500 procedures remaining at risk.

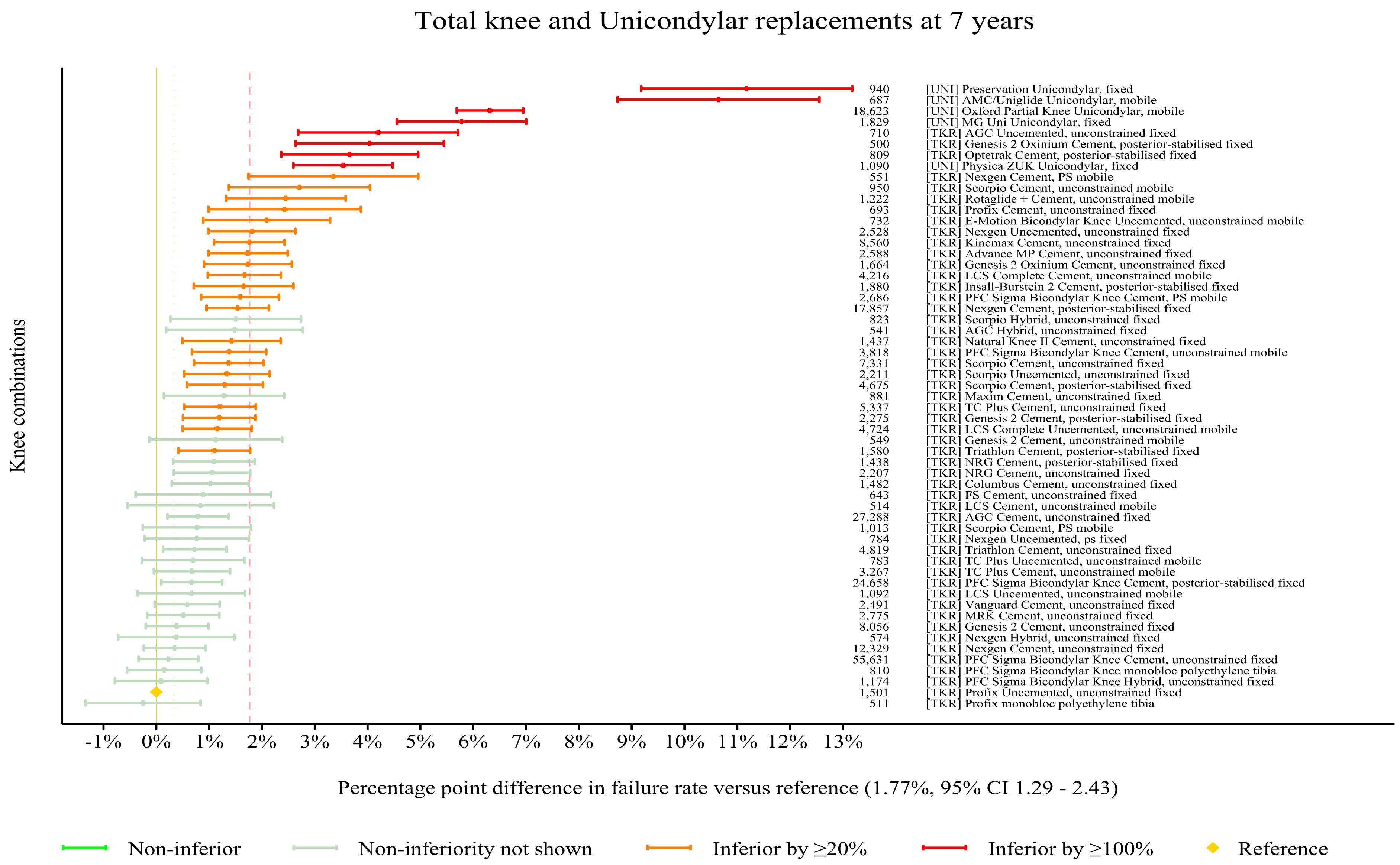

Supplementary Figure 4: Difference in cumulative revision of knee implants compared to a contemporary reference at 3 years in men, using all total knee and unicondylar replacements with ≥500 procedures remaining at risk.

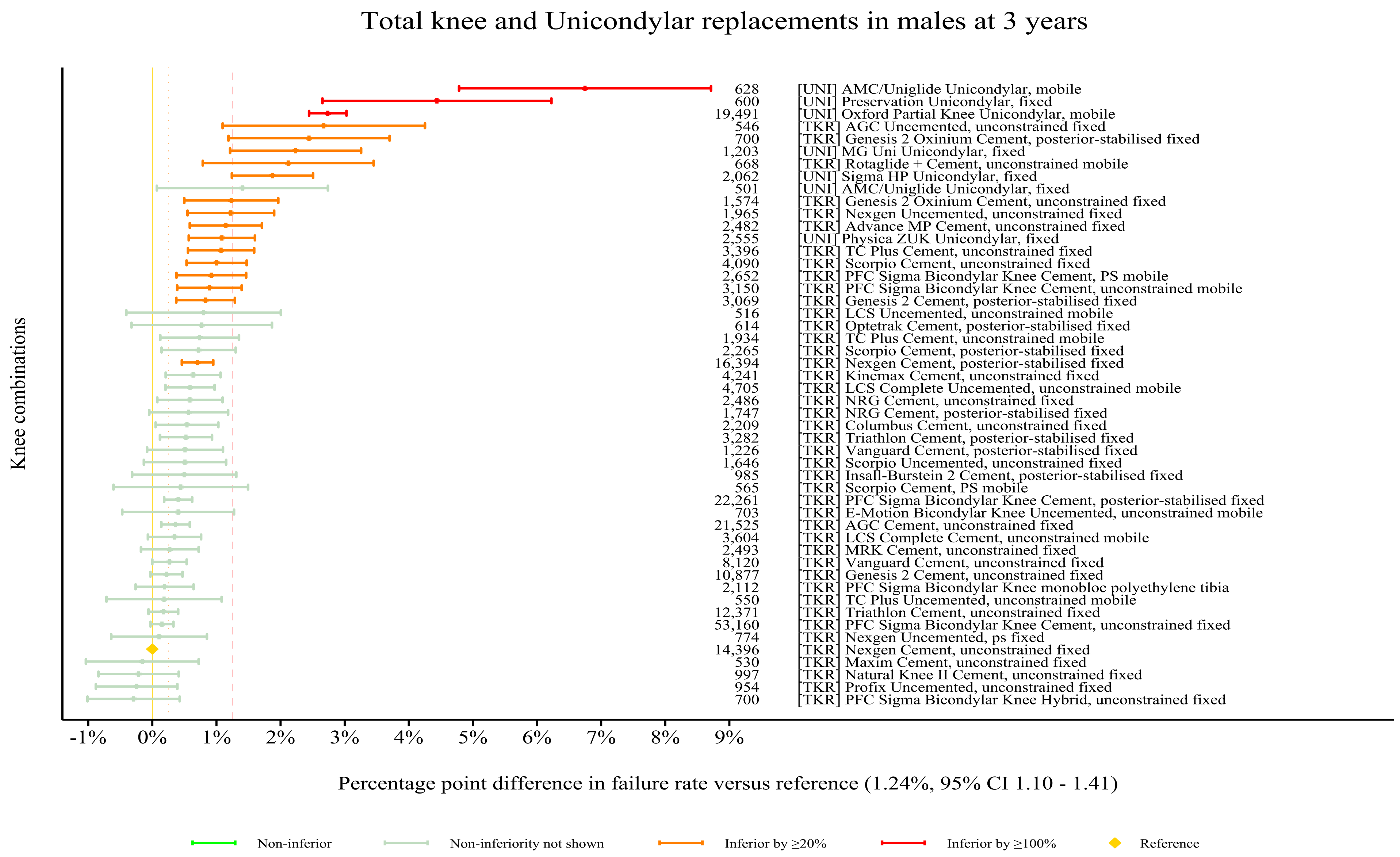

Supplementary Figure 5: Difference in cumulative revision of knee implants compared to a contemporary reference at 5 years in men, using all total knee and unicondylar replacements with ≥500 procedures remaining at risk.

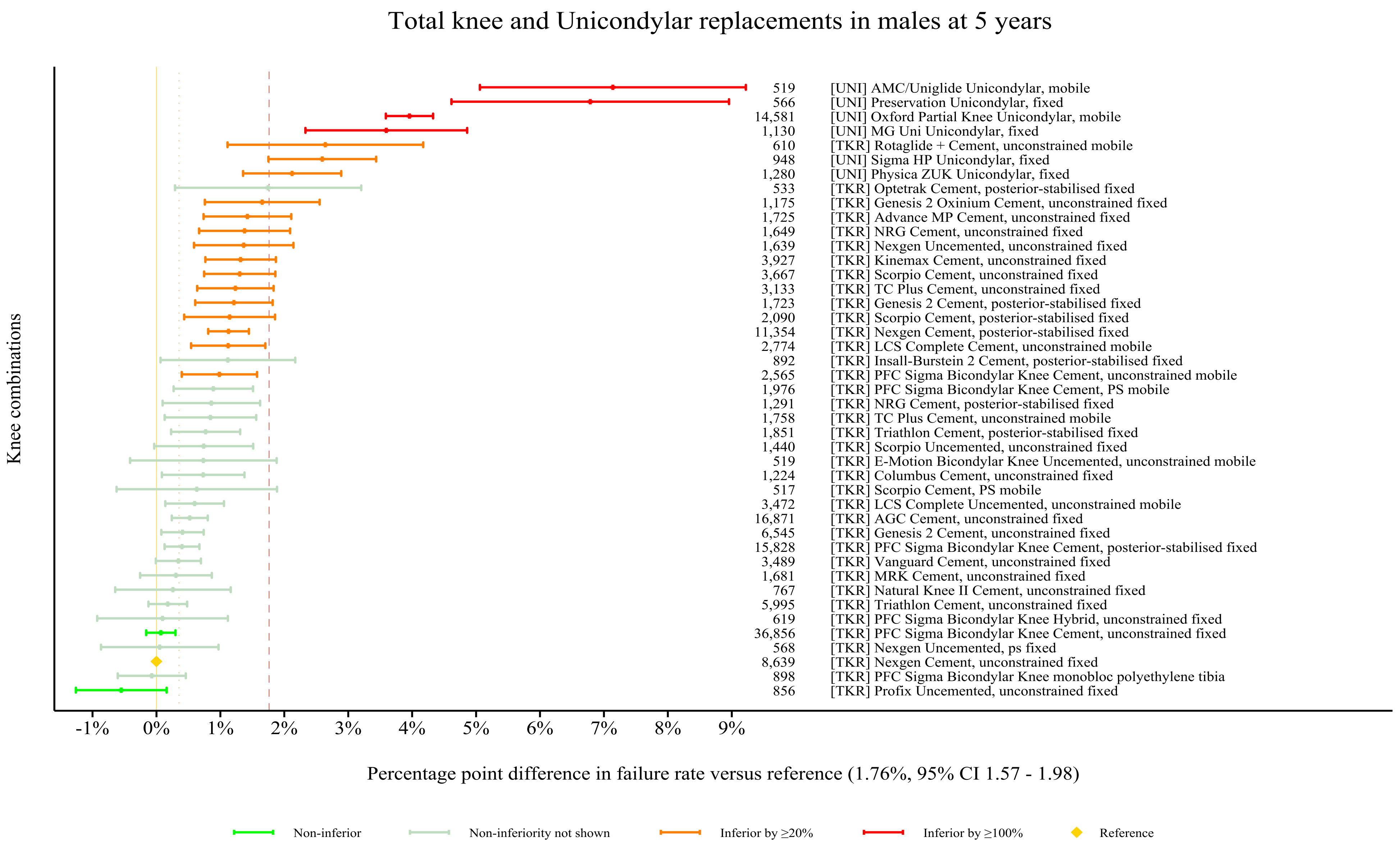

Supplementary Figure 6: Difference in cumulative revision of knee implants compared to a contemporary reference at 7 years in men, using all total knee and unicondylar replacements with ≥500 procedures remaining at risk.

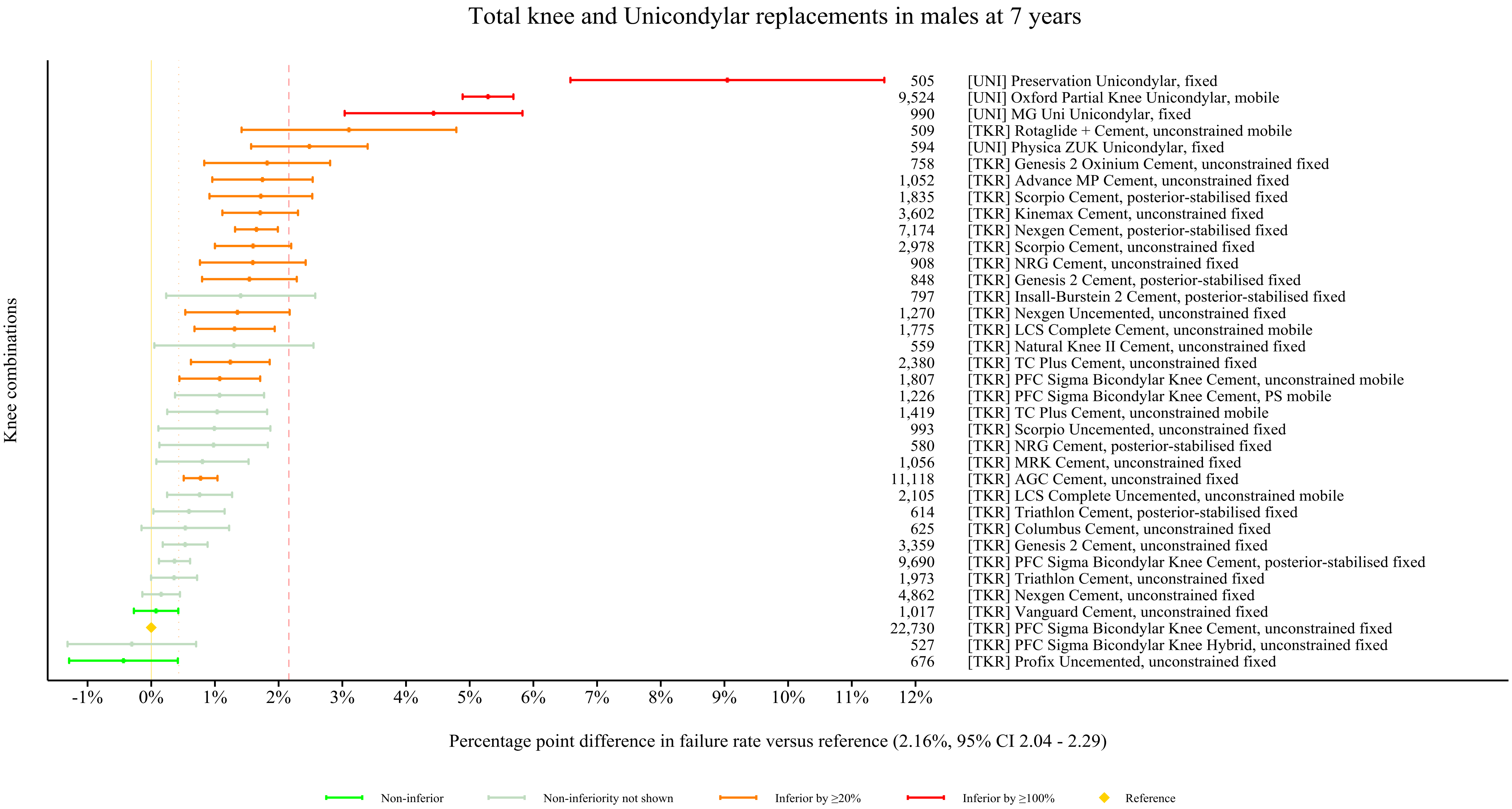

Supplementary Figure 7: Difference in cumulative revision of knee implants compared to a contemporary reference at 10 years in men, using all total knee and unicondylar replacements with ≥500 procedures remaining at risk.

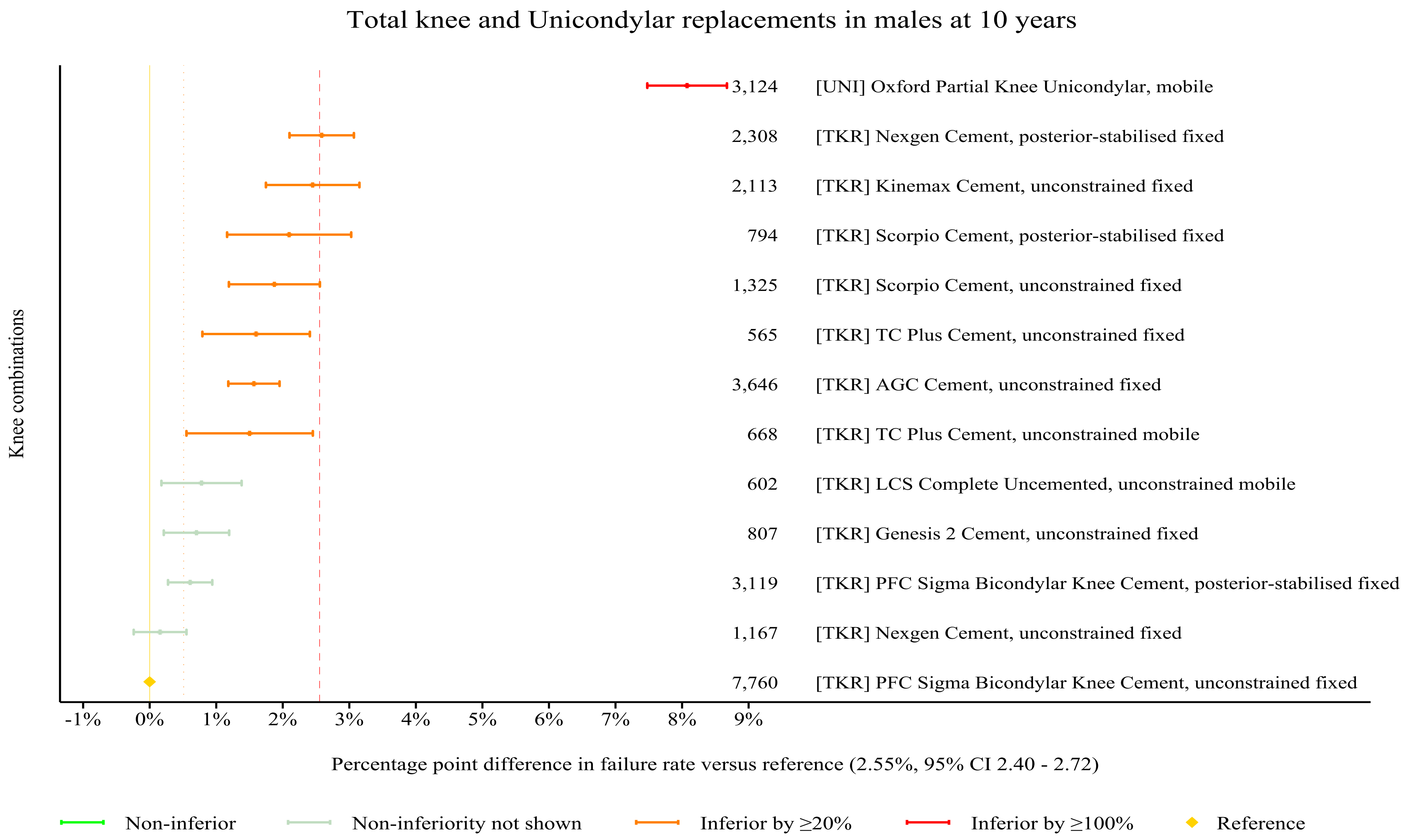

Supplementary Figure 8: Difference in cumulative revision of knee implants compared to a contemporary reference at 3 years in women, using all total knee and unicondylar replacements with ≥500 procedures remaining at risk.

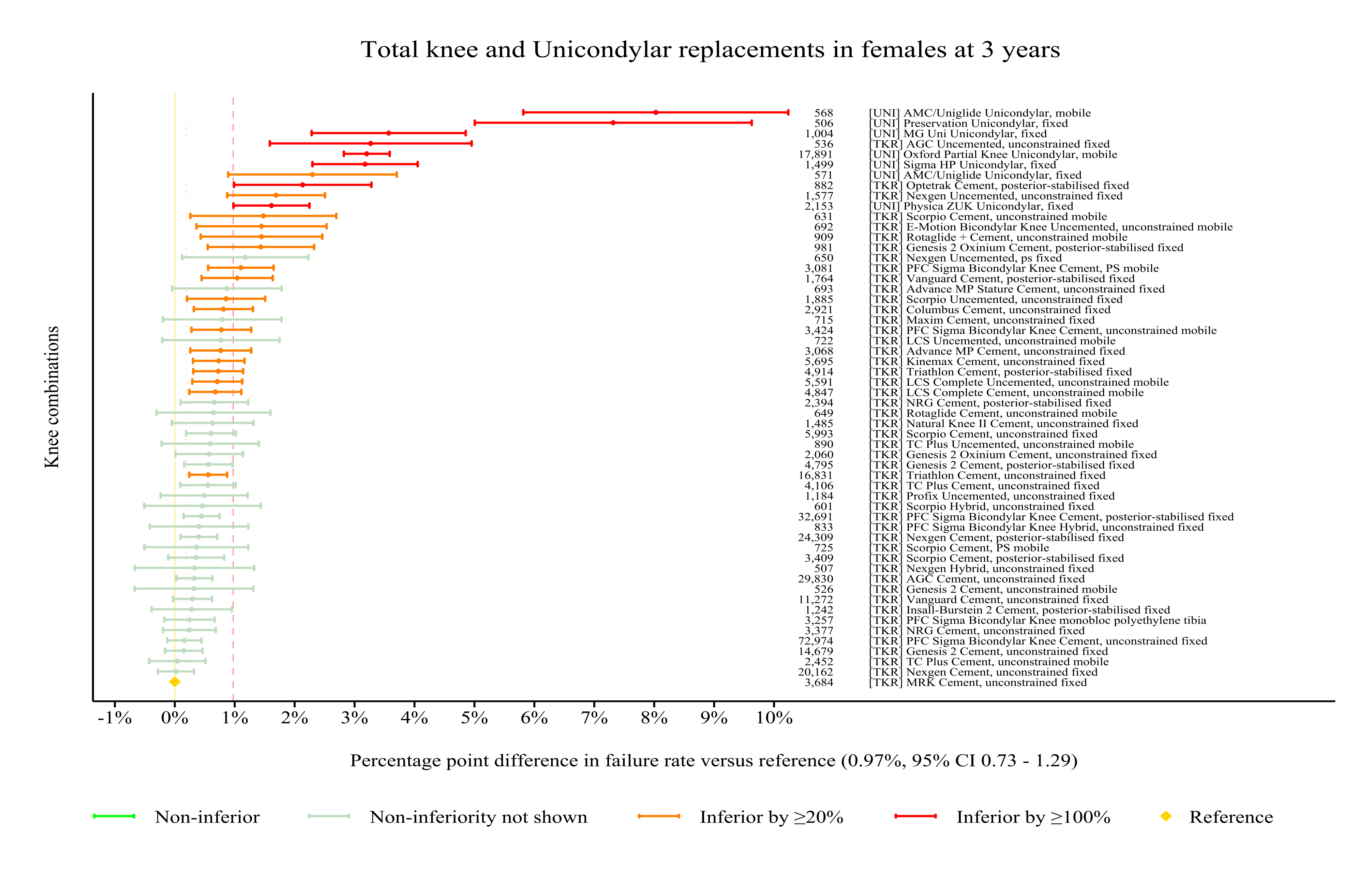

Supplementary Figure 9: Difference in cumulative revision of knee implants compared to a contemporary reference at 5 years in women, using all total knee and unicondylar replacements with ≥500 procedures remaining at risk.

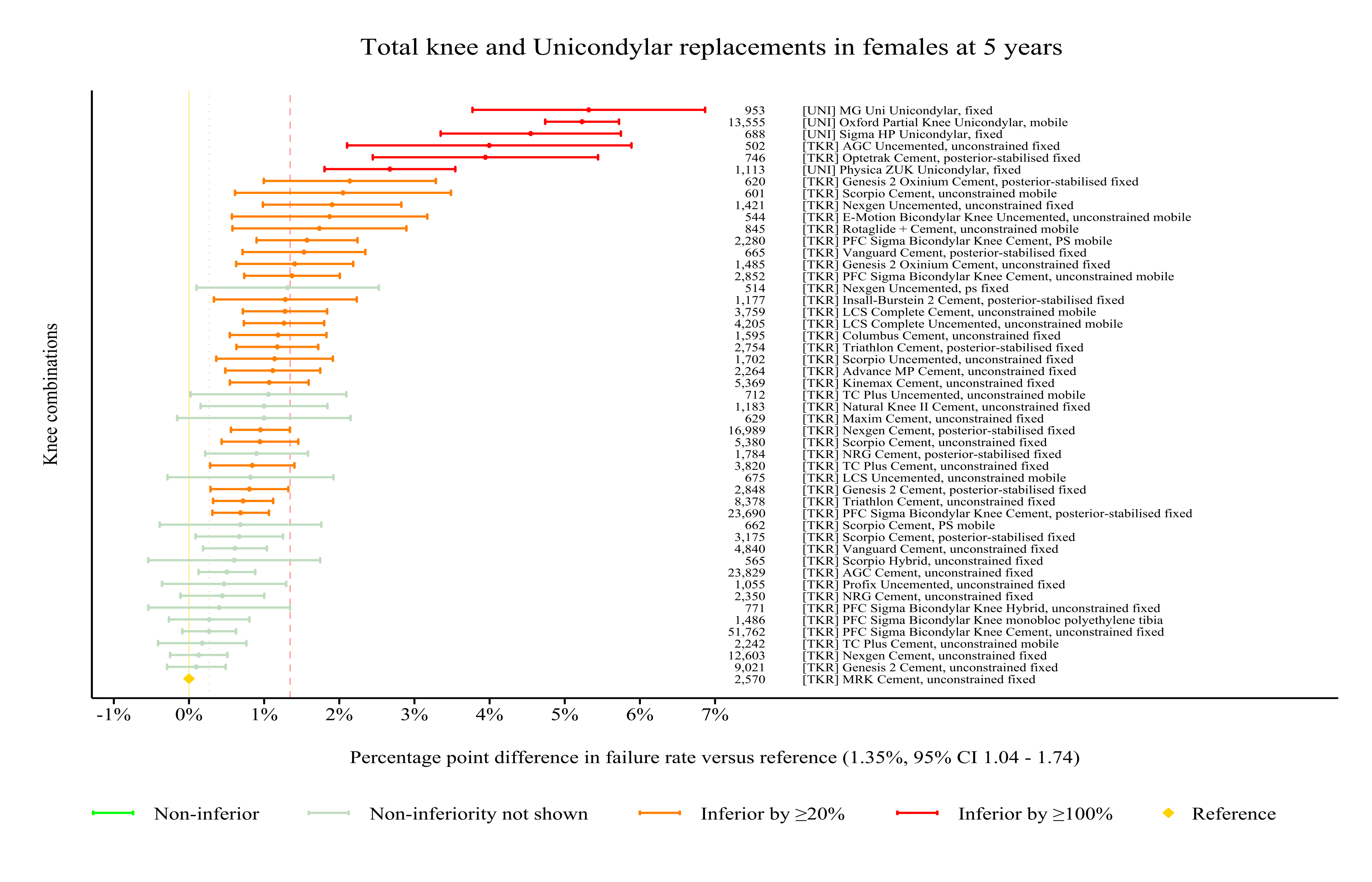

Supplementary Figure 10: Difference in cumulative revision of knee implants compared to a contemporary reference at 7 years in women, using all total knee and unicondylar replacements with ≥500 procedures remaining at risk.

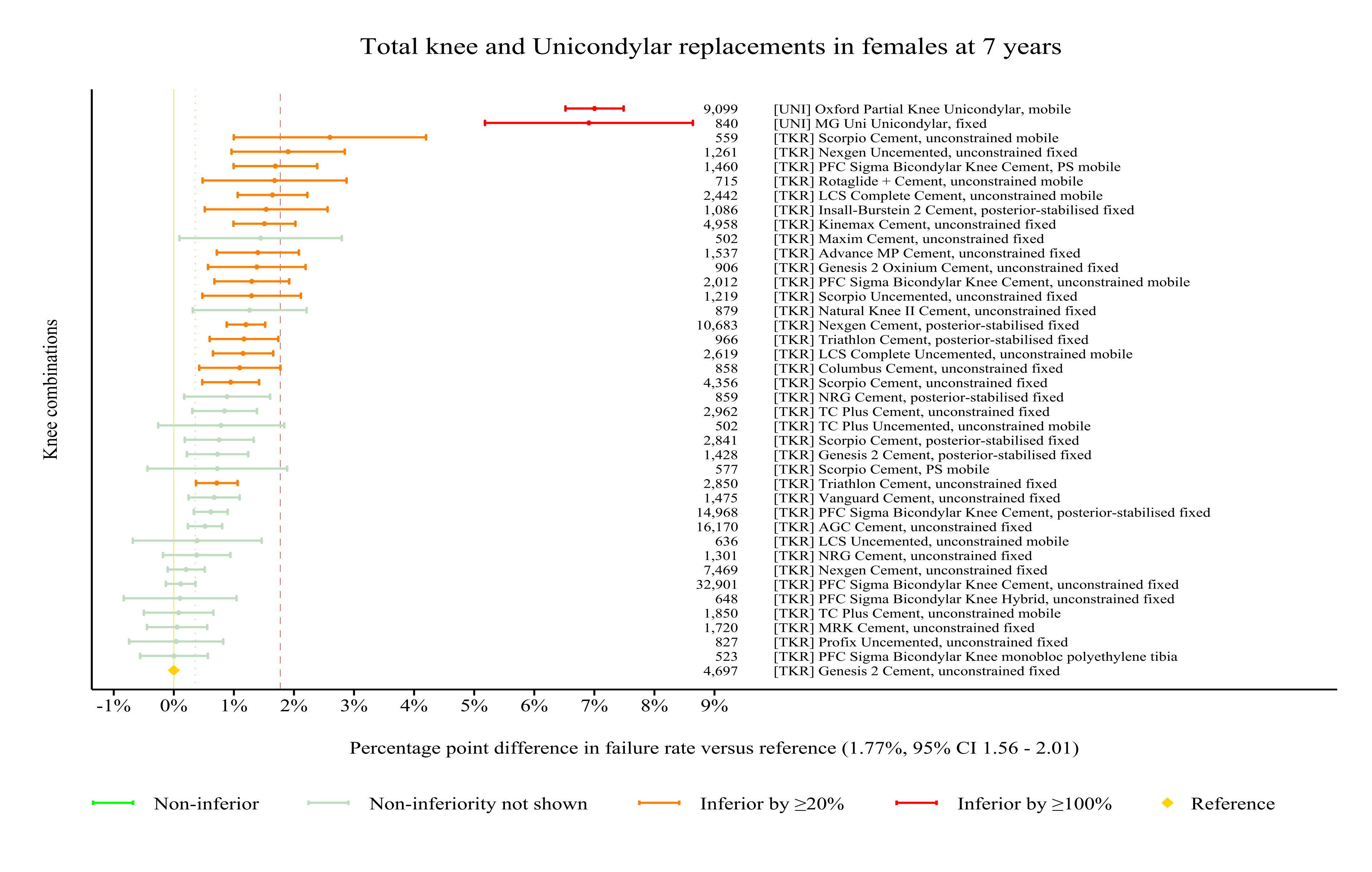

Supplementary Figure 11: Difference in cumulative revision of knee implants compared to a contemporary reference at 10 years in women, using all total knee and unicondylar replacements with ≥500 procedures remaining at risk.

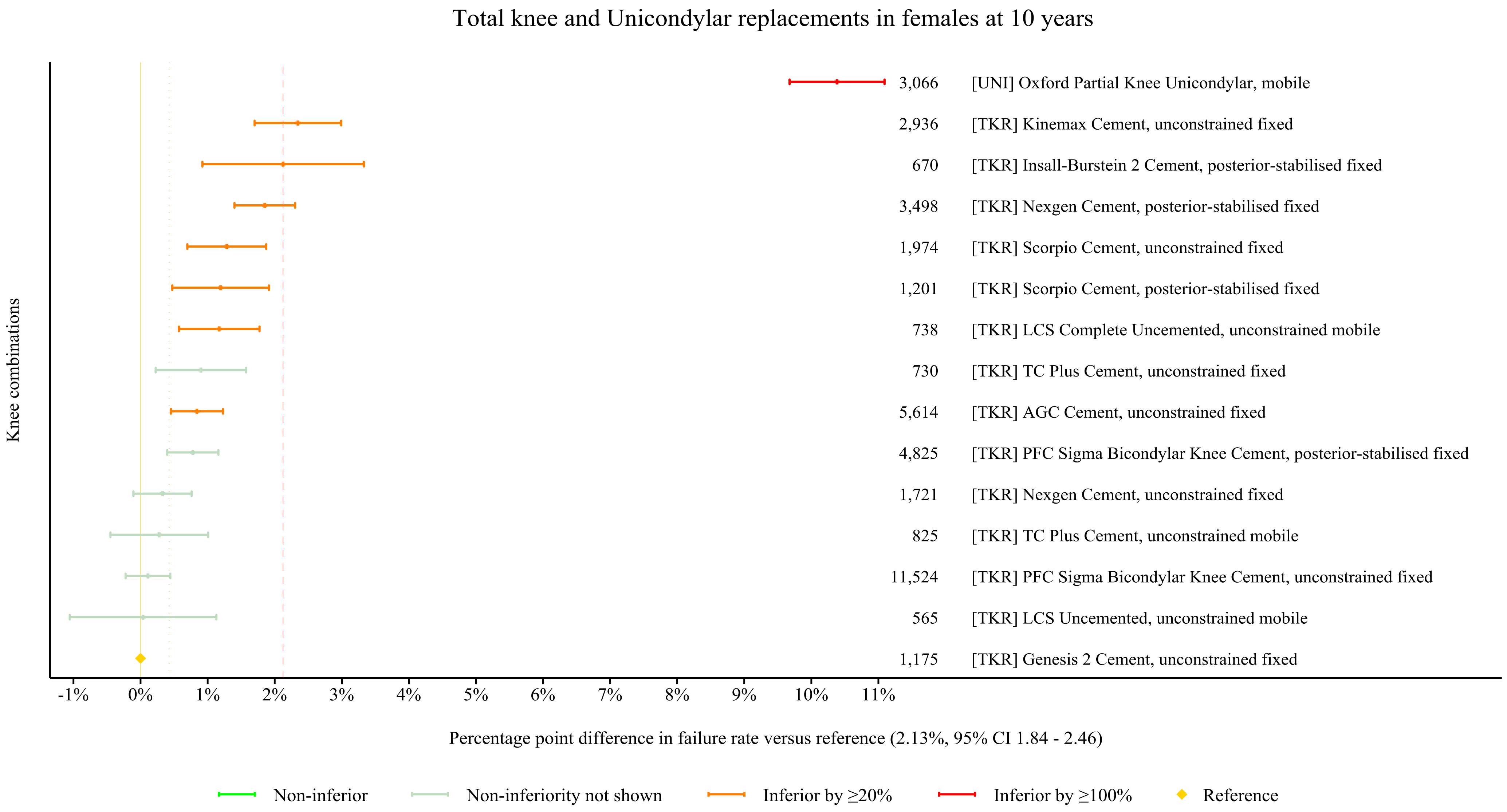

Supplementary Figure 12: Difference in cumulative revision of knee implants compared to a contemporary reference at 3 years in men less than 55 years, using all total knee and unicondylar replacements with ≥500 procedures remaining at risk.

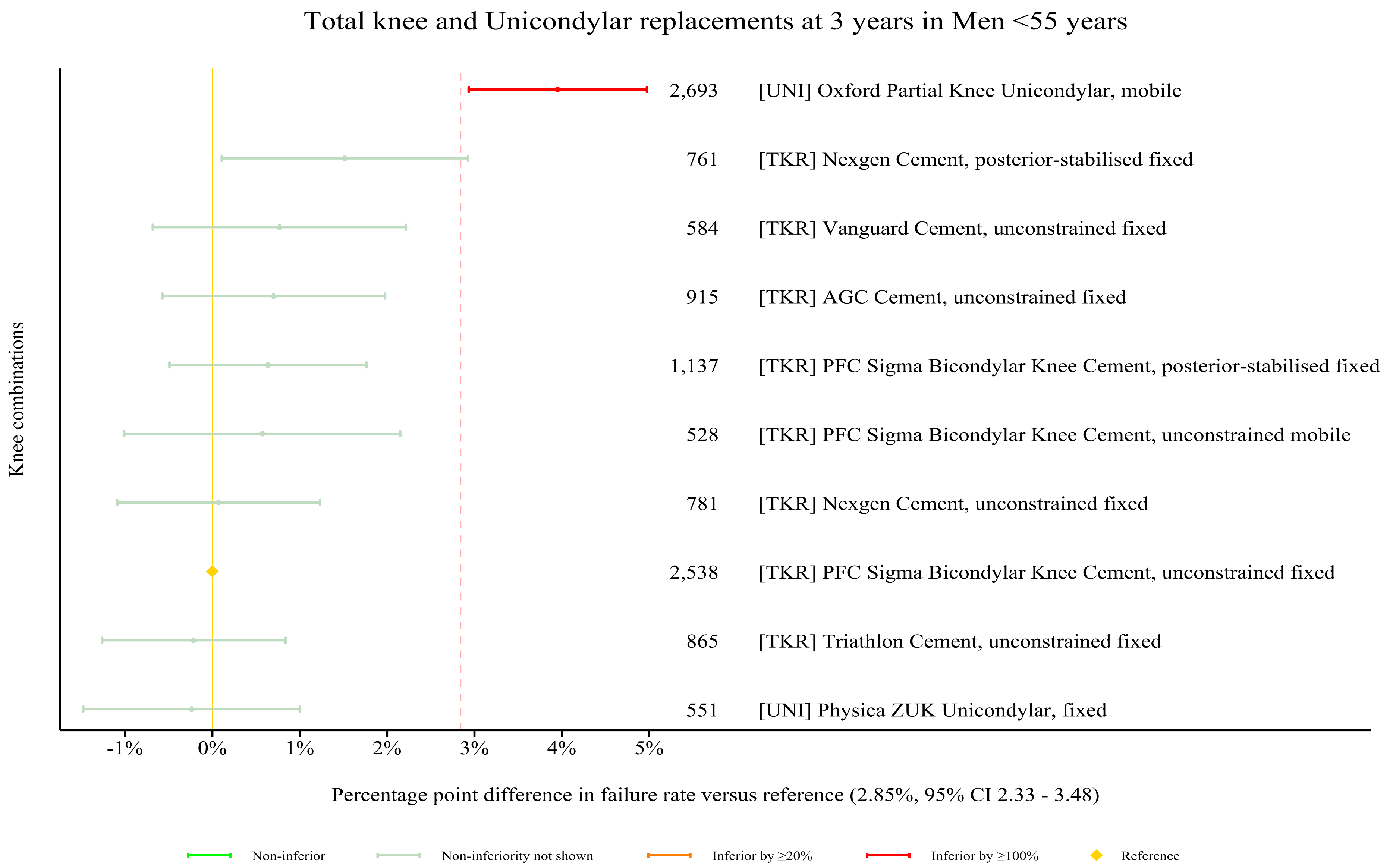

Supplementary Figure 13: Difference in cumulative revision of knee implants compared to a contemporary reference at 5 years in men less than 55 years, using all total knee and unicondylar replacements with ≥500 procedures remaining at risk.

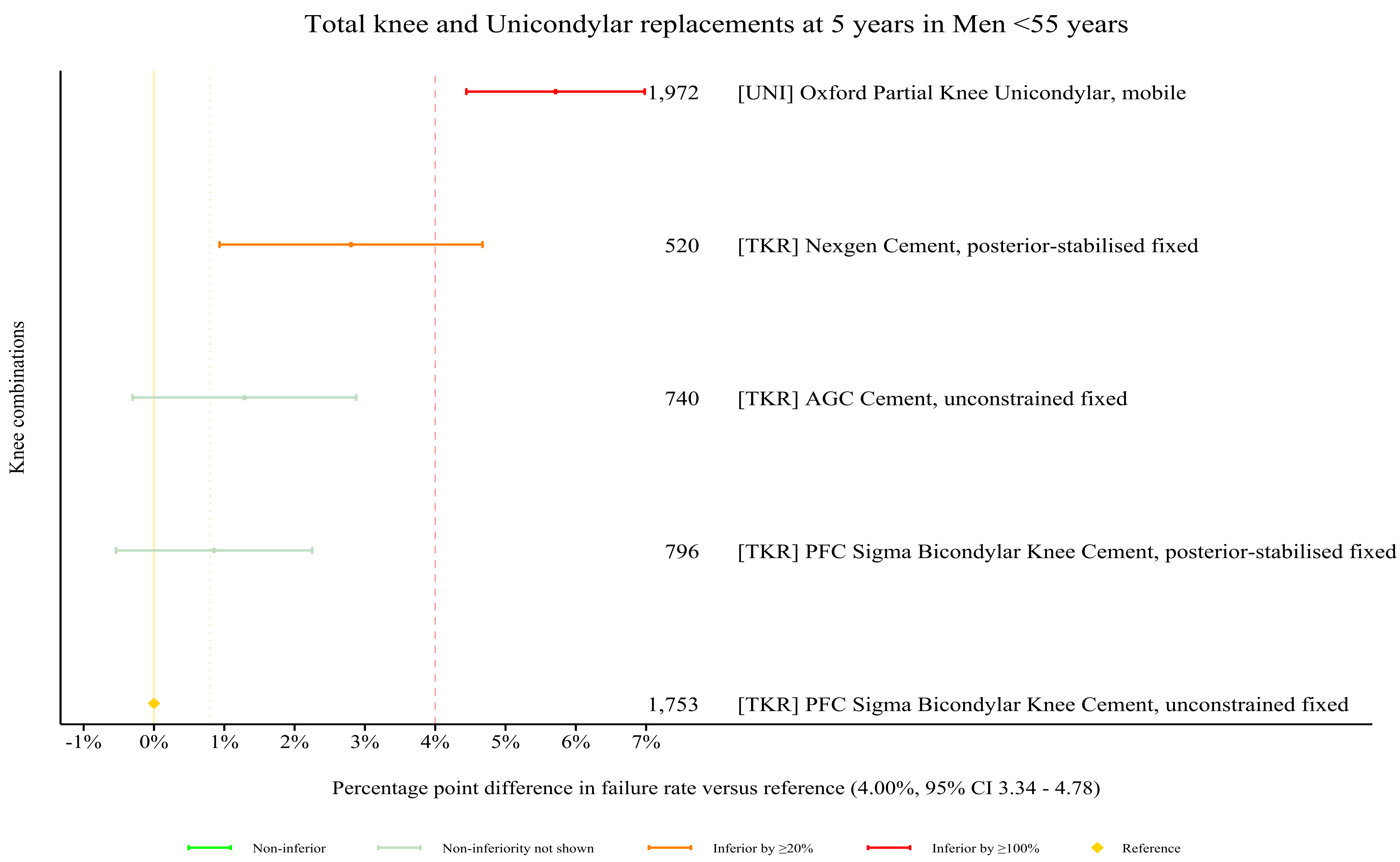

Supplementary Figure 14: Difference in cumulative revision of knee implants compared to a contemporary reference at 7 years in men less than 55 years, using all total knee and unicondylar replacements with ≥500 procedures remaining at risk.

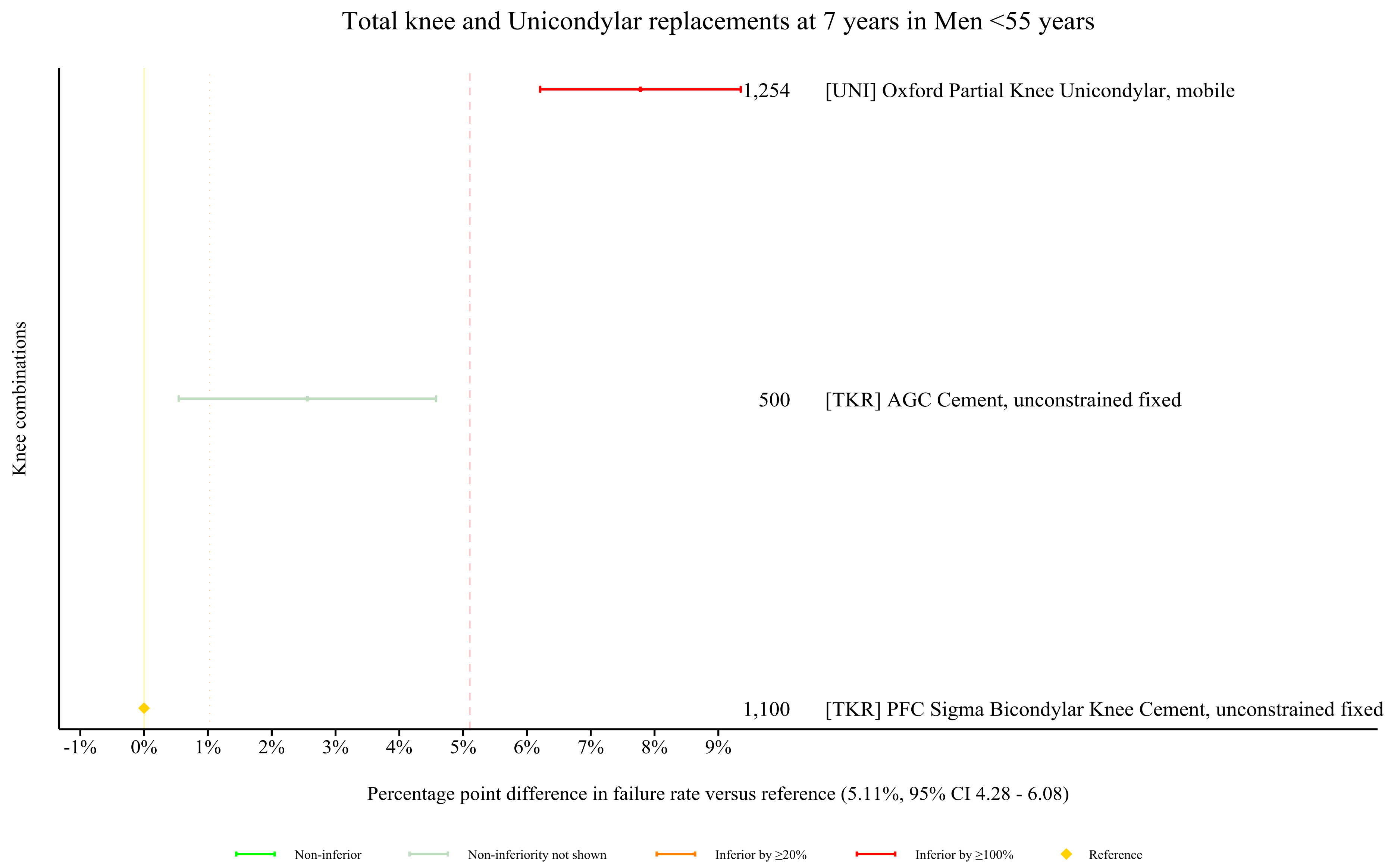

Supplementary Figure 15: Difference in cumulative revision of knee implants compared to a contemporary reference at 3 years in women less than 55 years, using all total knee and unicondylar replacements with ≥500 procedures remaining at risk.

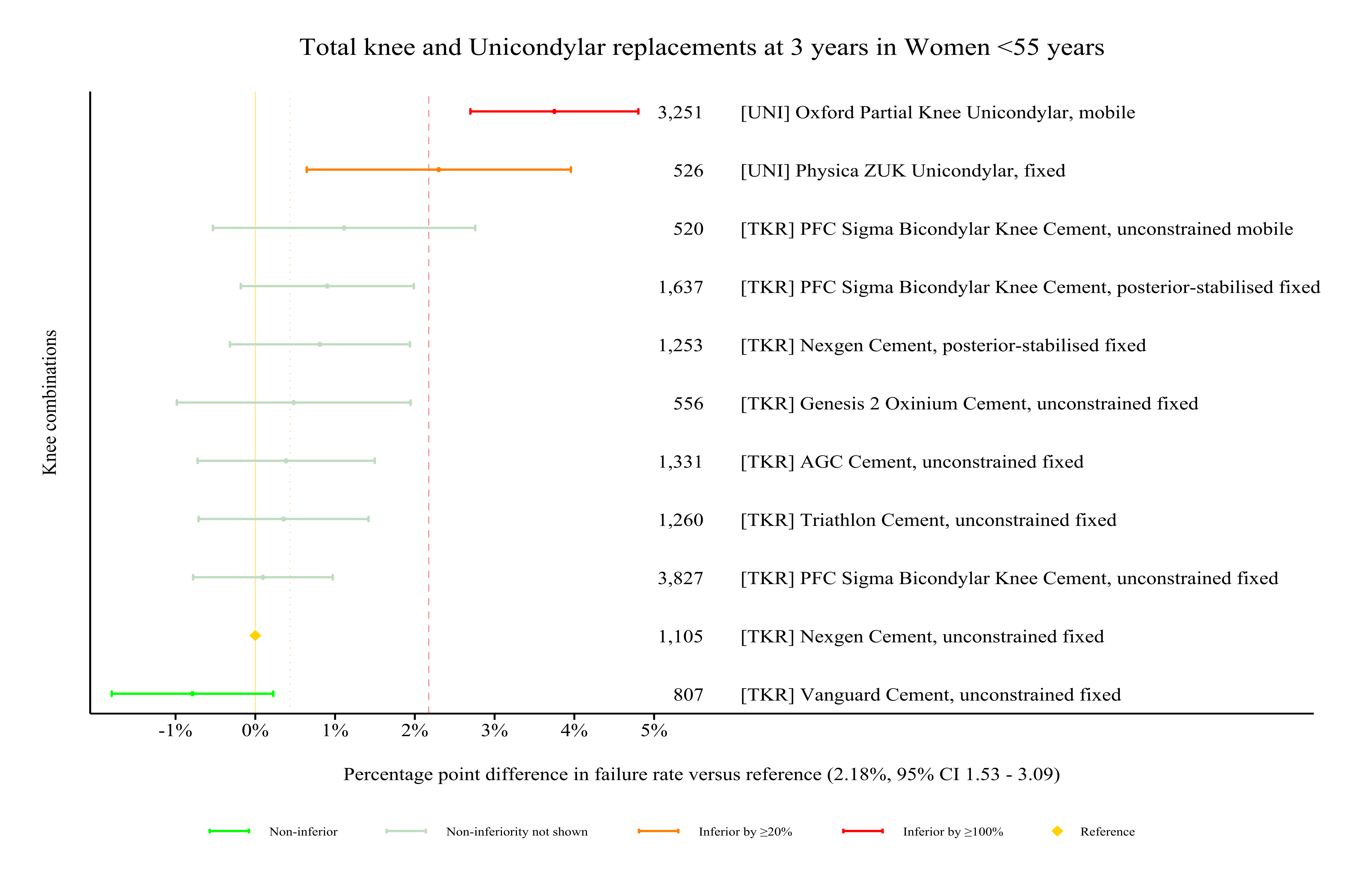

Supplementary Figure 16: Difference in cumulative revision of knee implants compared to a contemporary reference at 5 years in women less than 55 years, using all total knee and unicondylar replacements with ≥500 procedures remaining at risk.

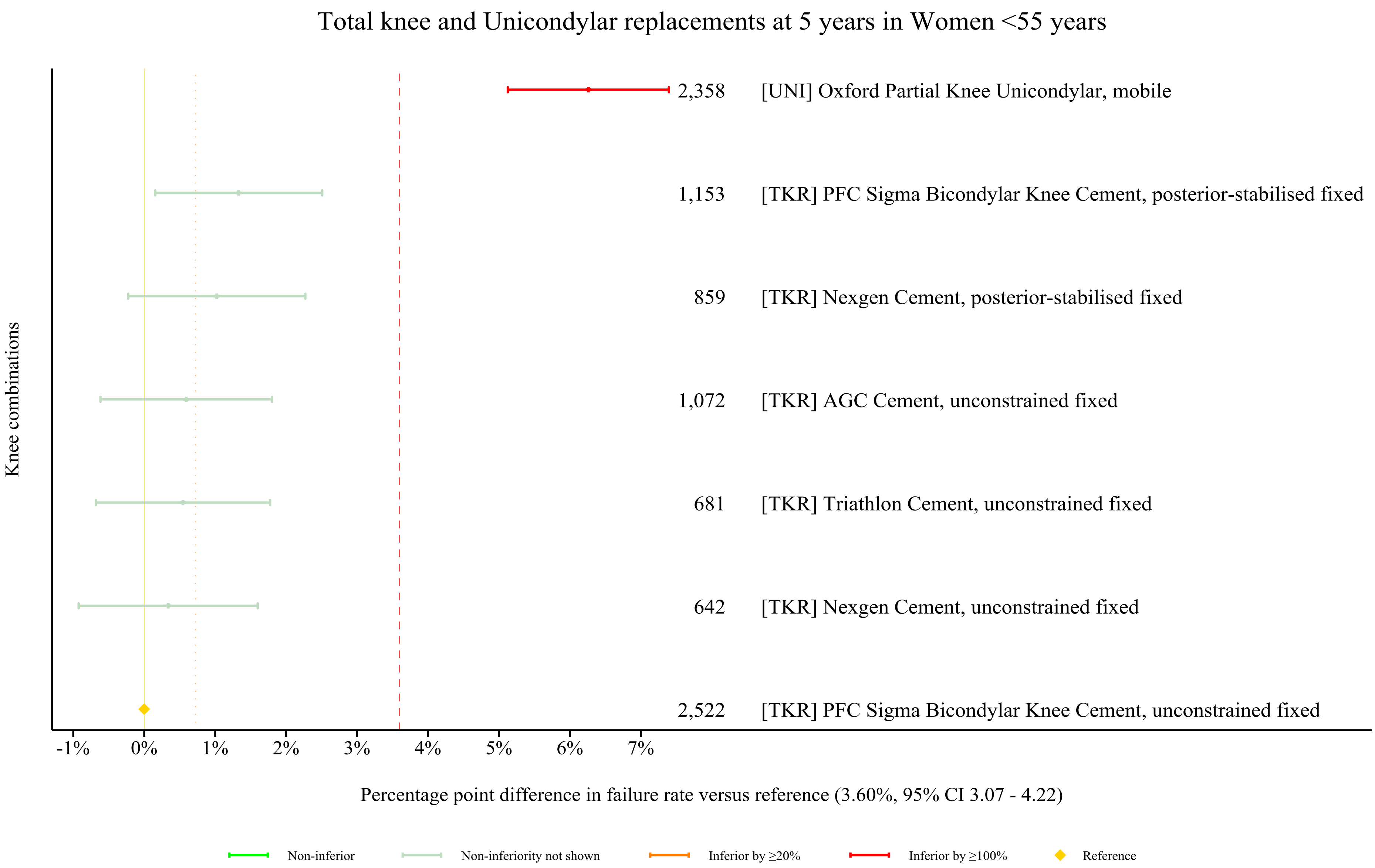

Supplementary Figure 17: Difference in cumulative revision of knee implants compared to a contemporary reference at 7 years in women less than 55 years, using all total knee and unicondylar replacements with ≥500 procedures remaining at risk.

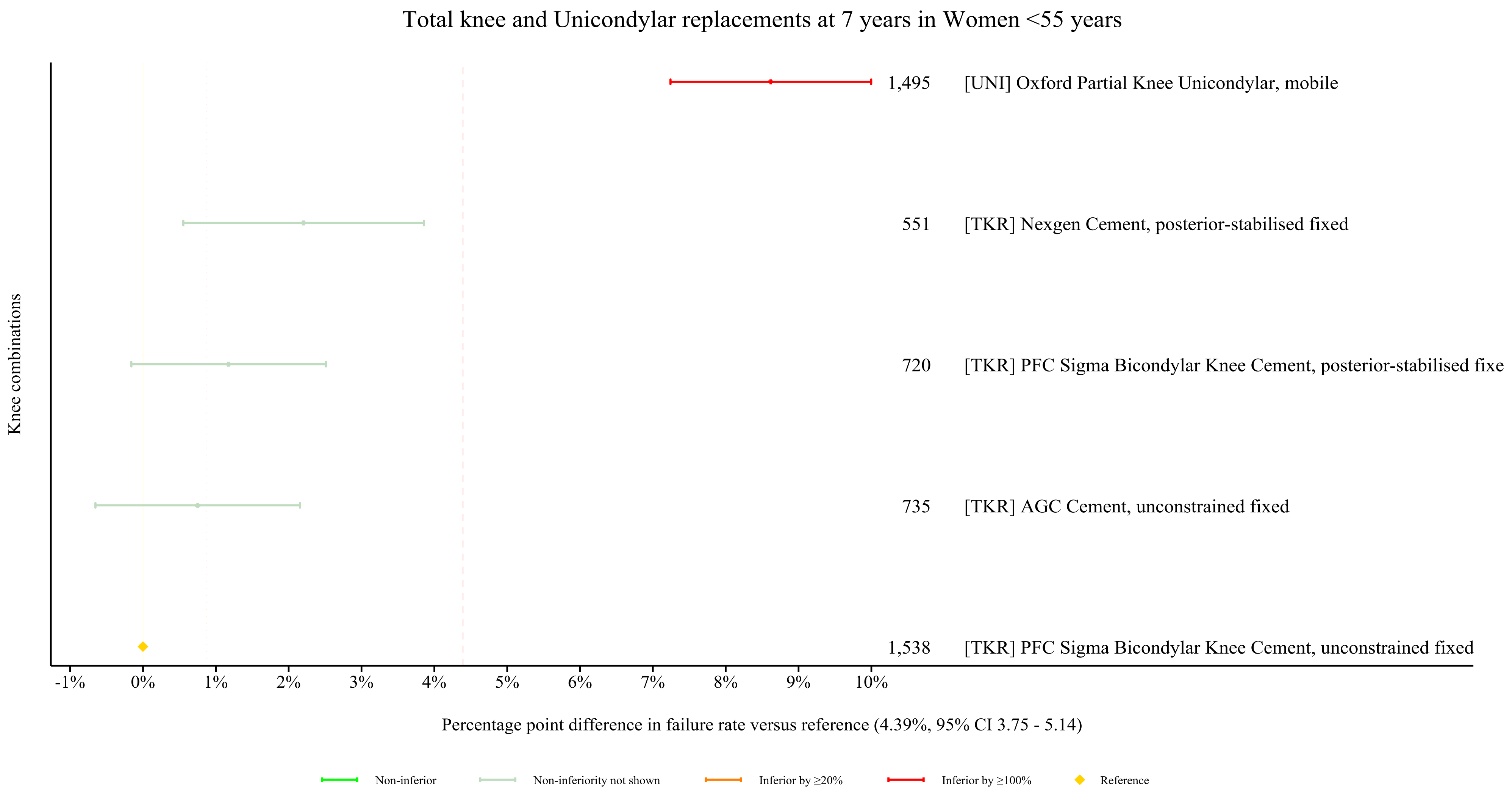

Supplementary Figure 18: Difference in cumulative revision of knee implants compared to a contemporary reference at 3 years in men aged between 55 and 75 years, using all total knee and unicondylar replacements with ≥500 procedures remaining at risk.

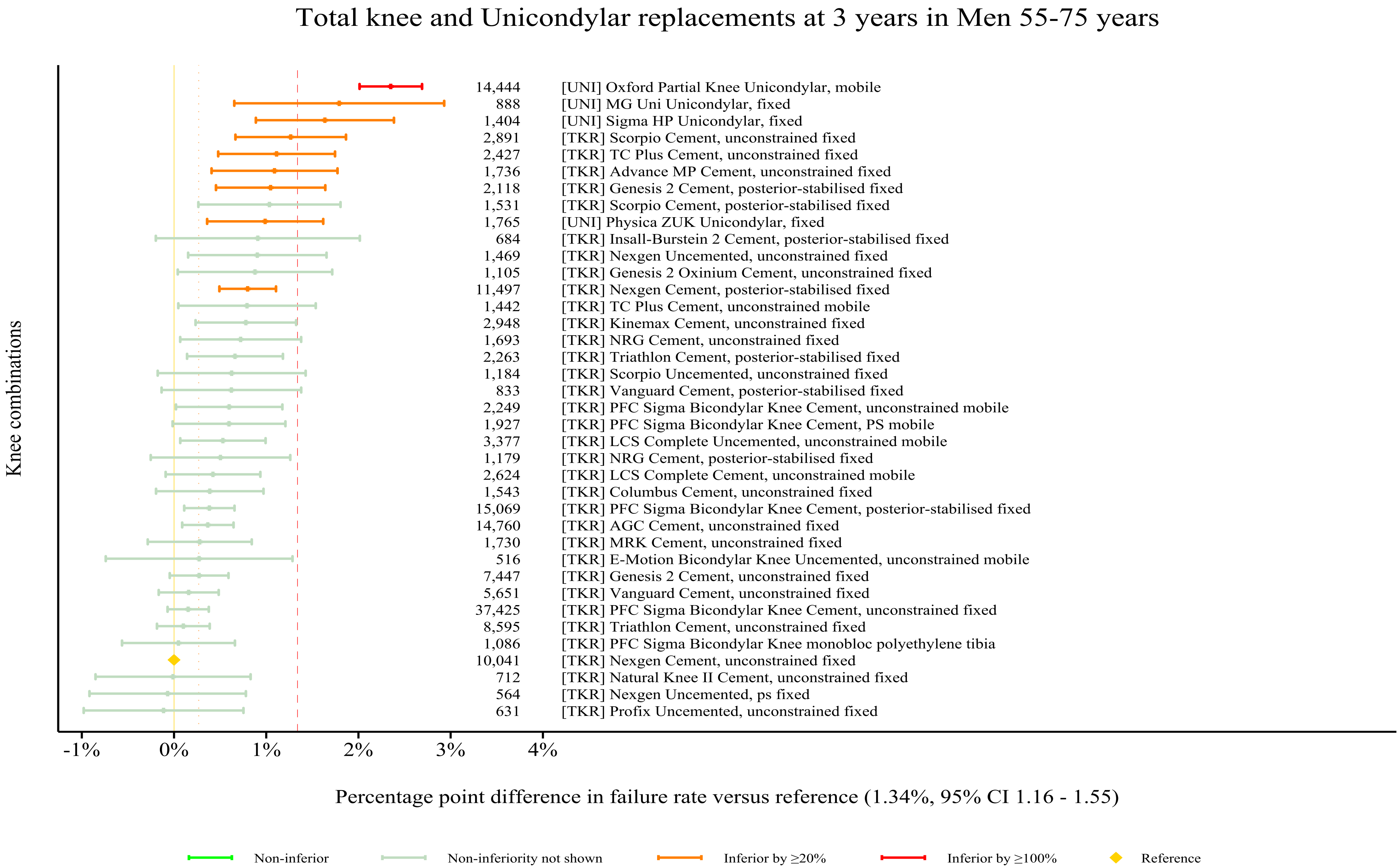

Supplementary Figure 19: Difference in cumulative revision of knee implants compared to a contemporary reference at 5 years in men aged between 55 and 75 years, using all total knee and unicondylar replacements with ≥500 procedures remaining at risk.

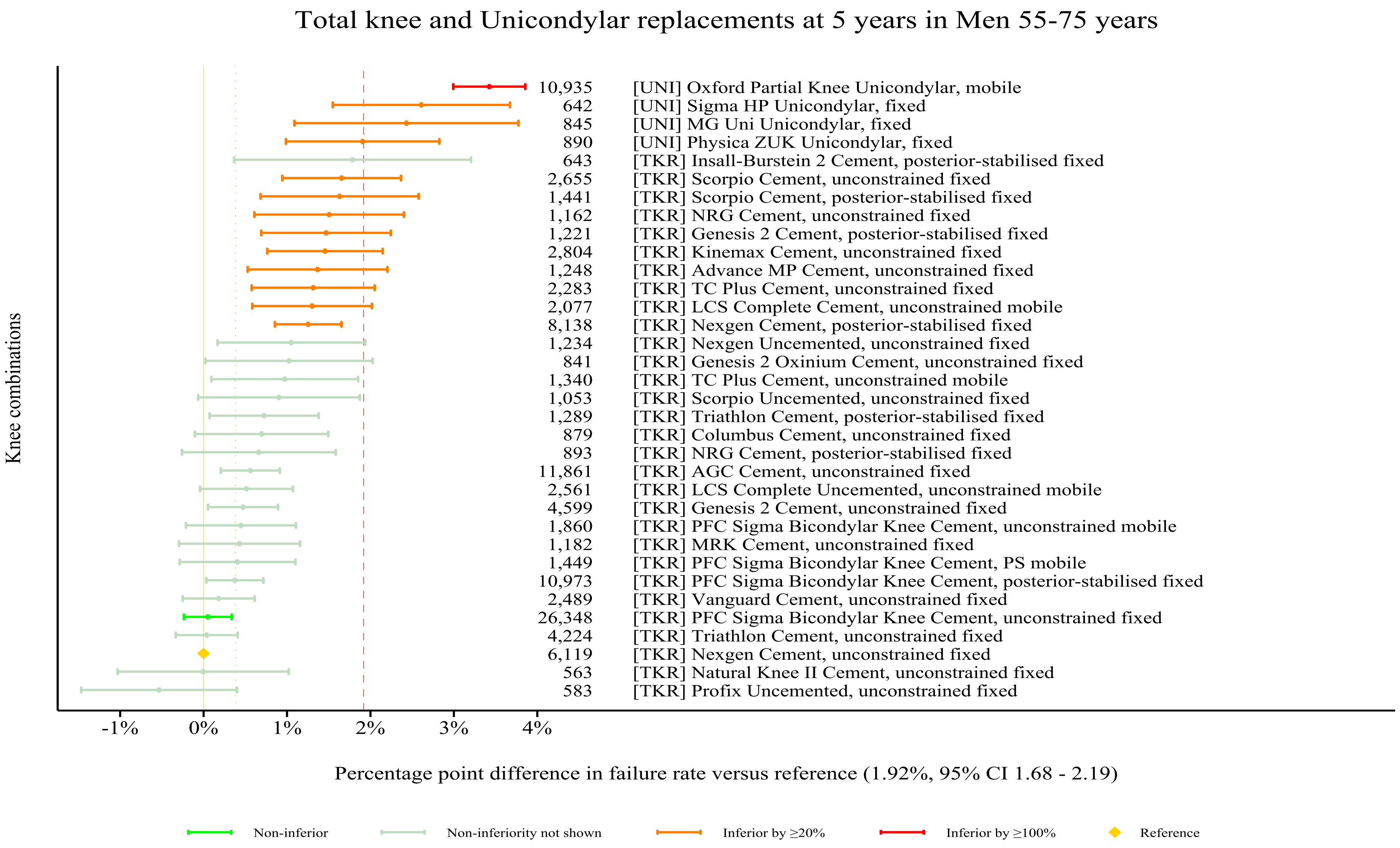

Supplementary Figure 20: Difference in cumulative revision of knee implants compared to a contemporary reference at 10 years in men aged between 55 and 75 years, using all total knee and unicondylar replacements with ≥500 procedures remaining at risk.

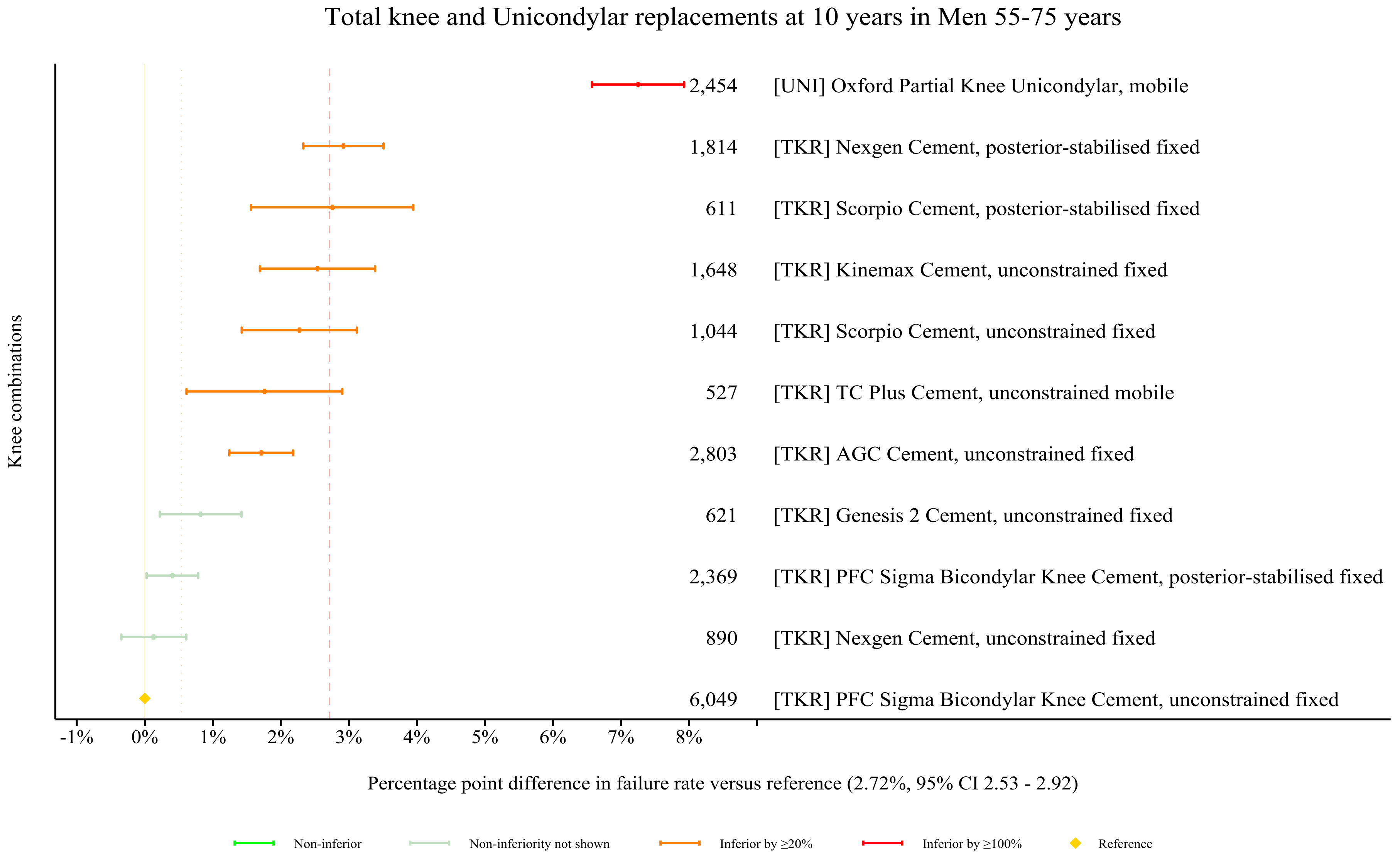

Supplementary Figure 21: Difference in cumulative revision of knee implants compared to a contemporary reference at 3 years in women aged between 55 and 75 years, using all total knee and unicondylar replacements with ≥500 procedures remaining at risk.

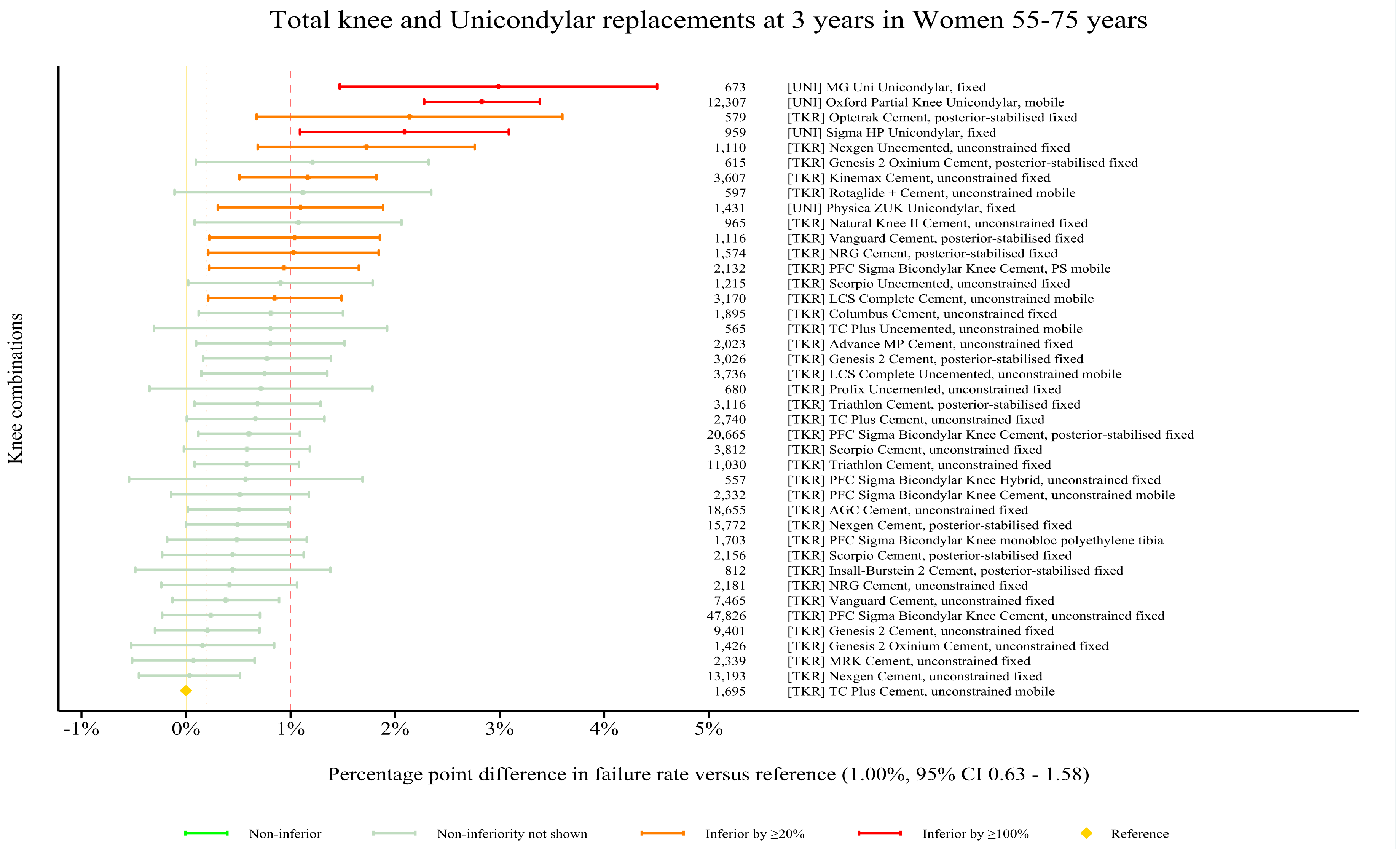

Supplementary Figure 22: Difference in cumulative revision of knee implants compared to a contemporary reference at 5 years in women aged between 55 and 75 years, using all total knee and unicondylar replacements with ≥500 procedures remaining at risk.

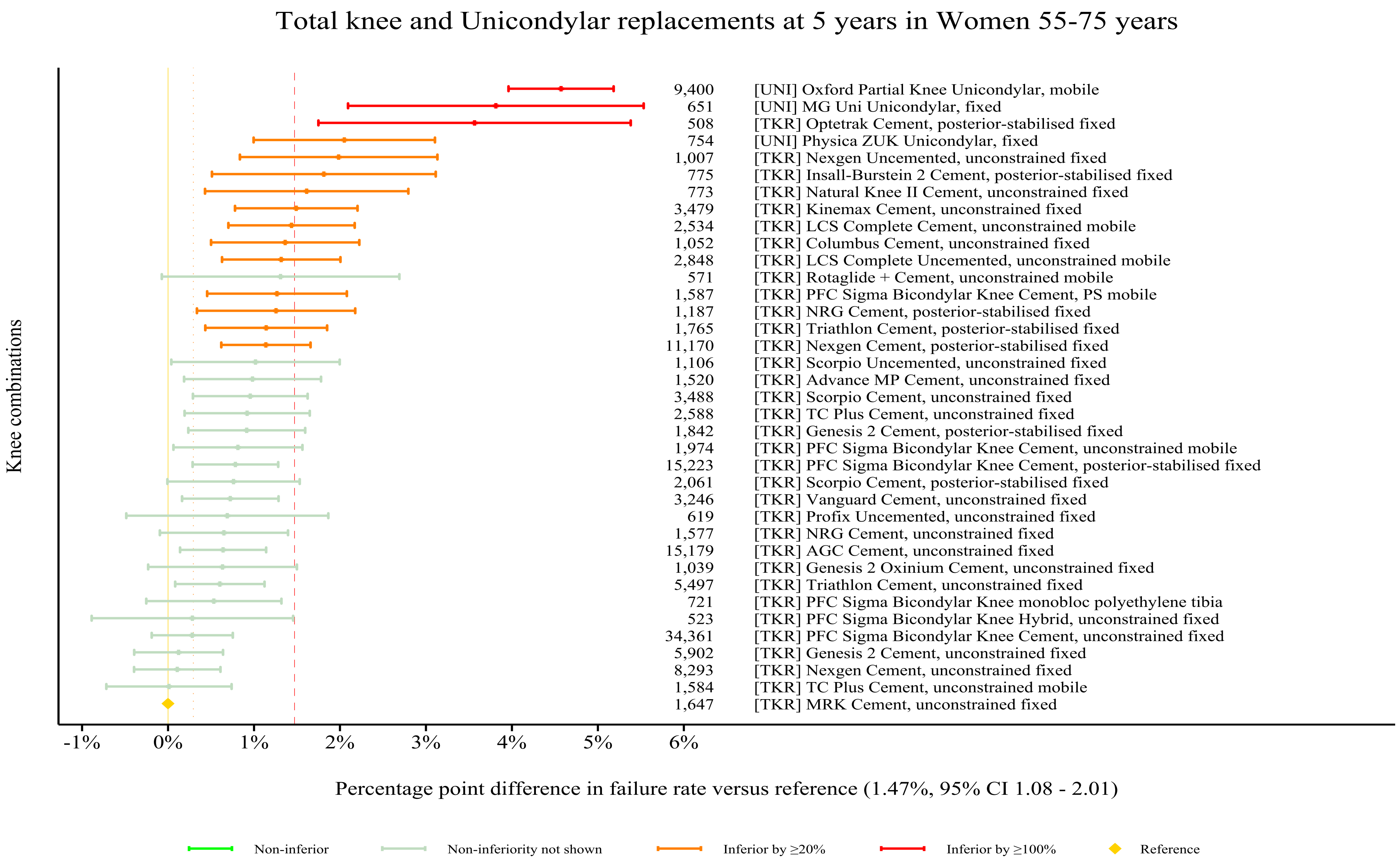

Supplementary Figure 23: Difference in cumulative revision of knee implants compared to a contemporary reference at 10 years in women aged between 55 and 75 years, using all total knee and unicondylar replacements with ≥500 procedures remaining at risk.

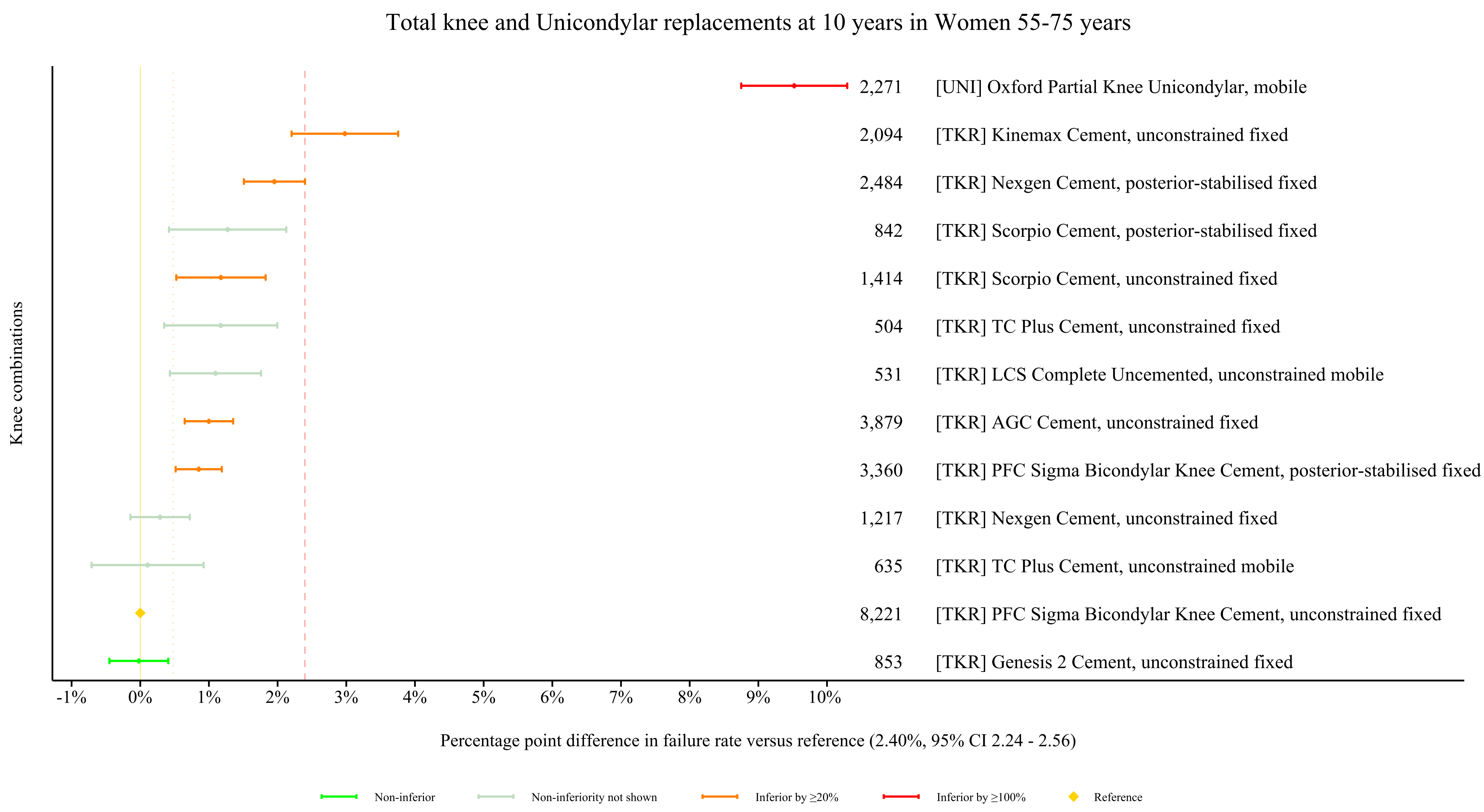

Supplementary Figure 24: Difference in cumulative revision of knee implants compared to a contemporary reference at 3 years in men aged over 75 years, using all total knee and unicondylar replacements with ≥500 procedures remaining at risk.

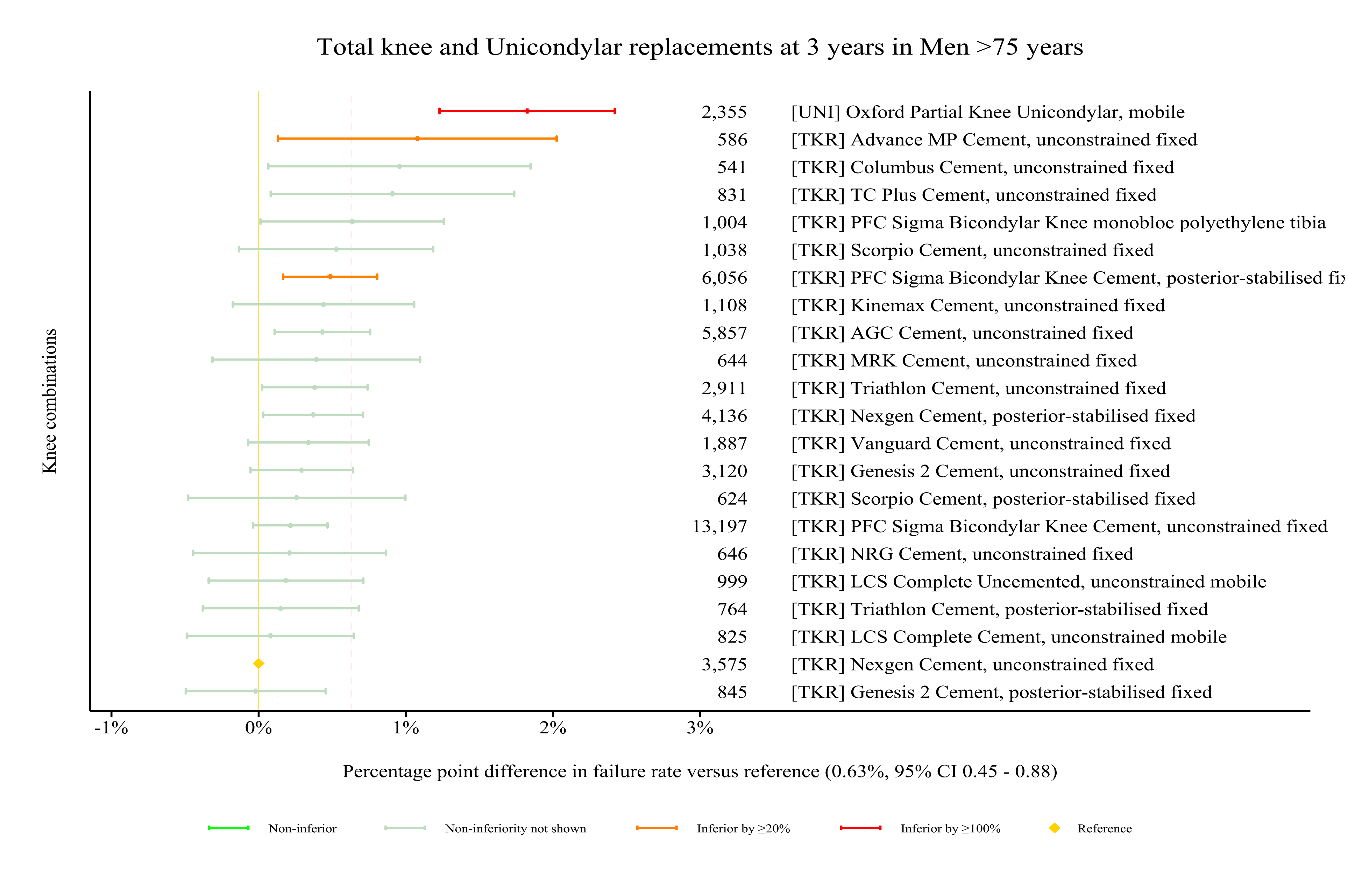

Supplementary Figure 25: Difference in cumulative revision of knee implants compared to a contemporary reference at 5 years in men aged over 75 years, using all total knee and unicondylar replacements with ≥500 procedures remaining at risk.

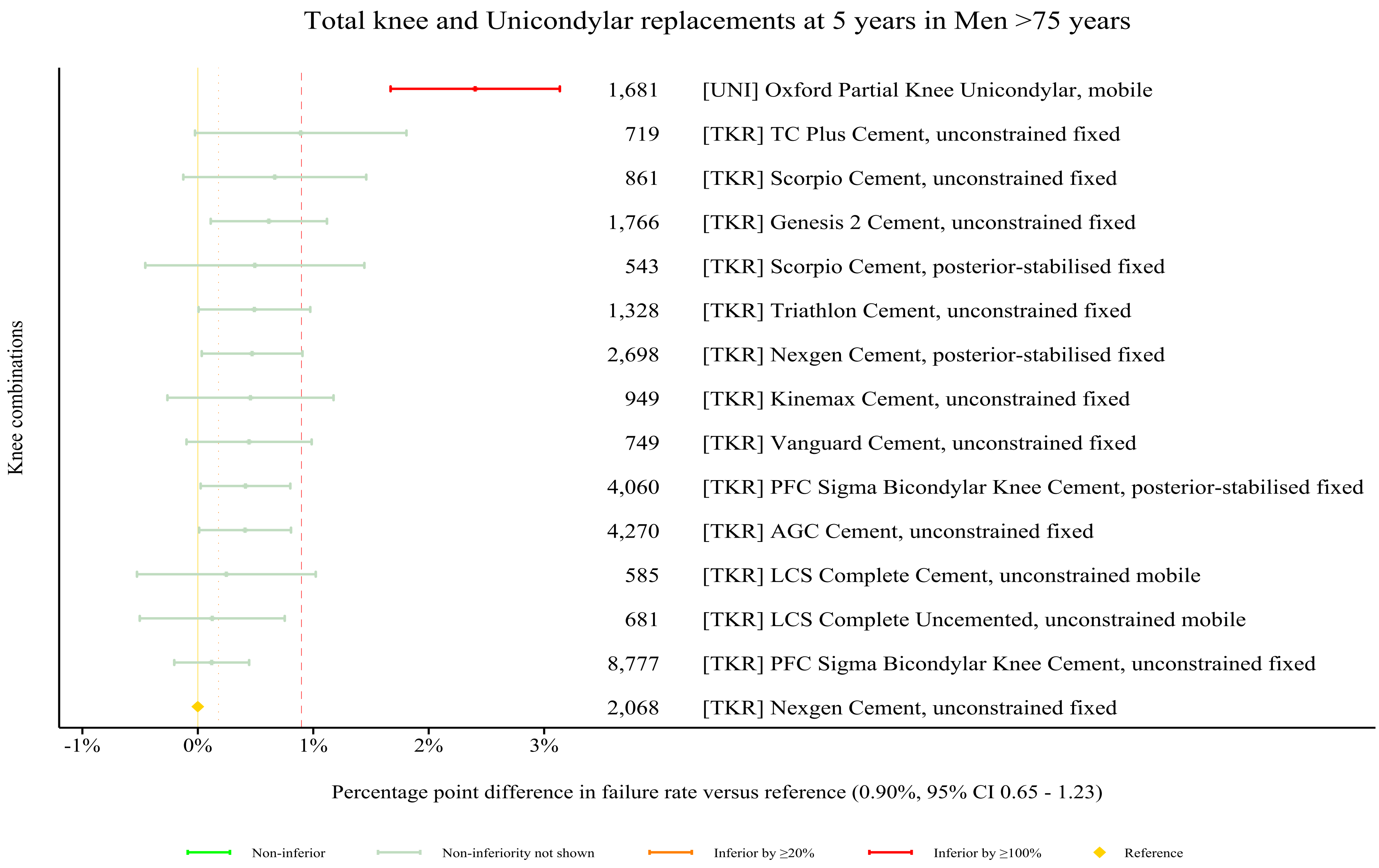

Supplementary Figure 26: Difference in cumulative revision of knee implants compared to a contemporary reference at 7 years in men aged over 75 years, using all total knee and unicondylar replacements with ≥500 procedures remaining at risk.

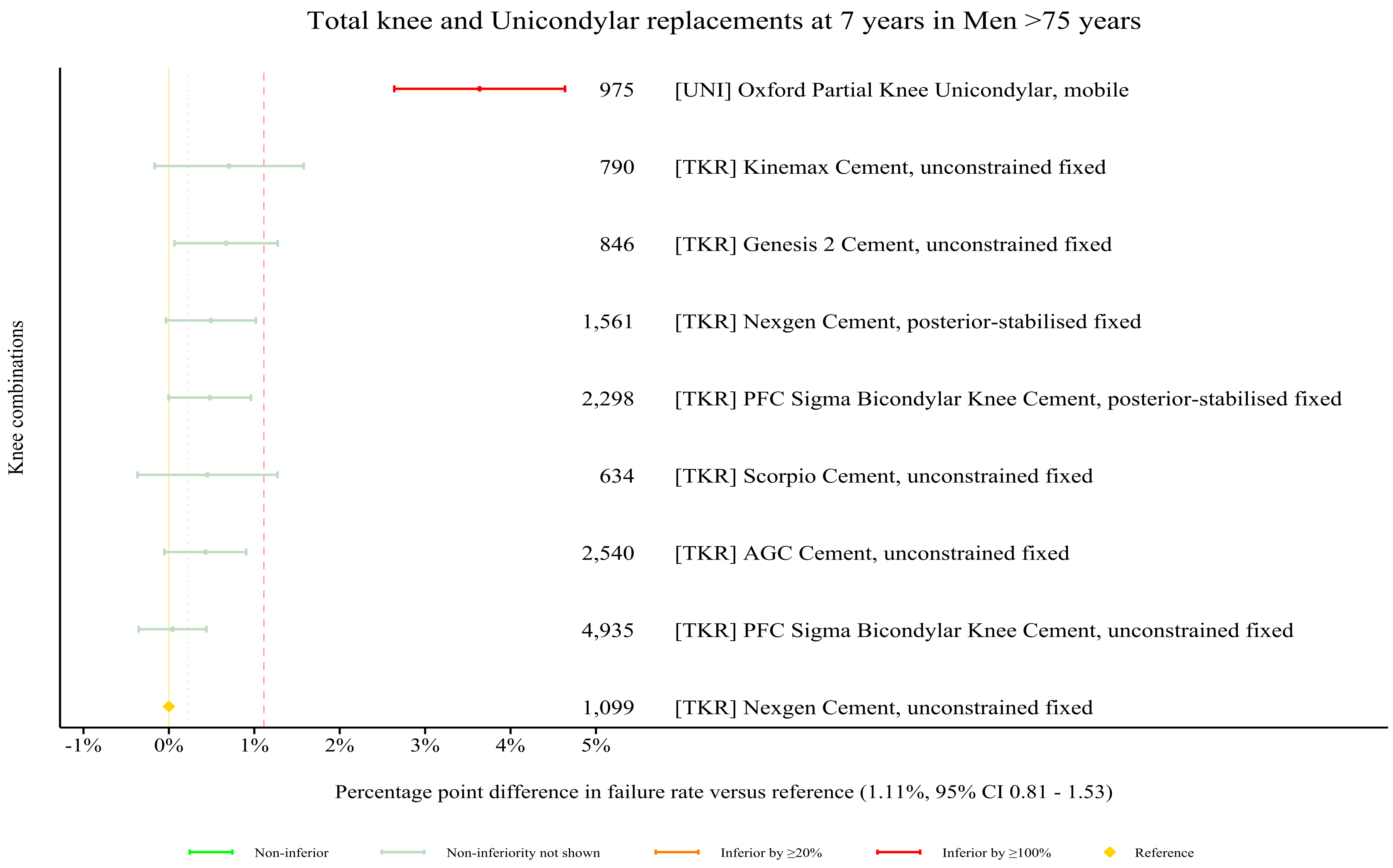

Supplementary Figure 27: Difference in cumulative revision of knee implants compared to a contemporary reference at 10 years in men aged over 75 years, using all total knee and unicondylar replacements with ≥500 procedures remaining at risk.

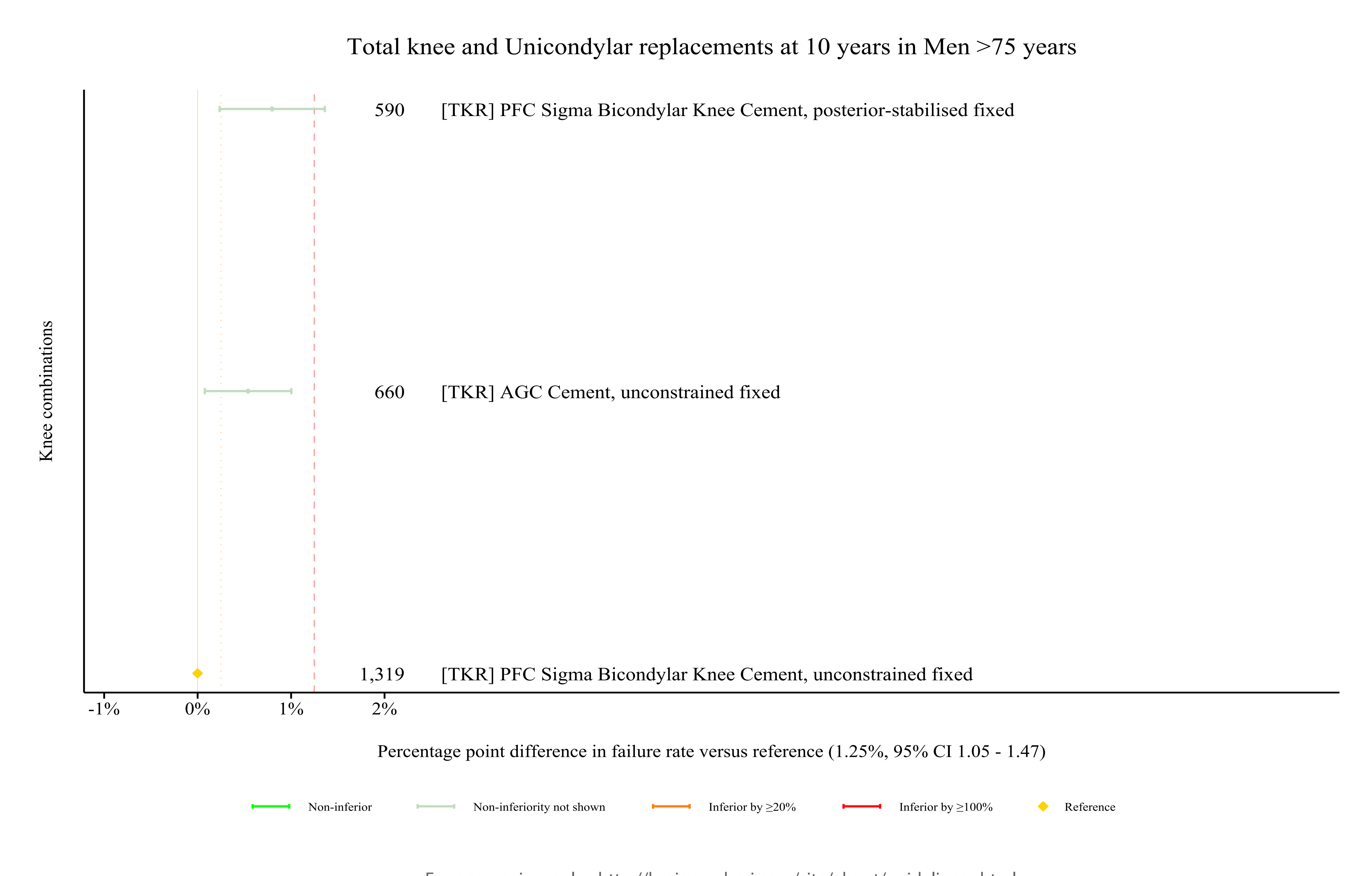

Supplementary Figure 28: Difference in cumulative revision of knee implants compared to a contemporary reference at 3 years in women aged over 75 years, using all total knee and unicondylar replacements with ≥500 procedures remaining at risk.

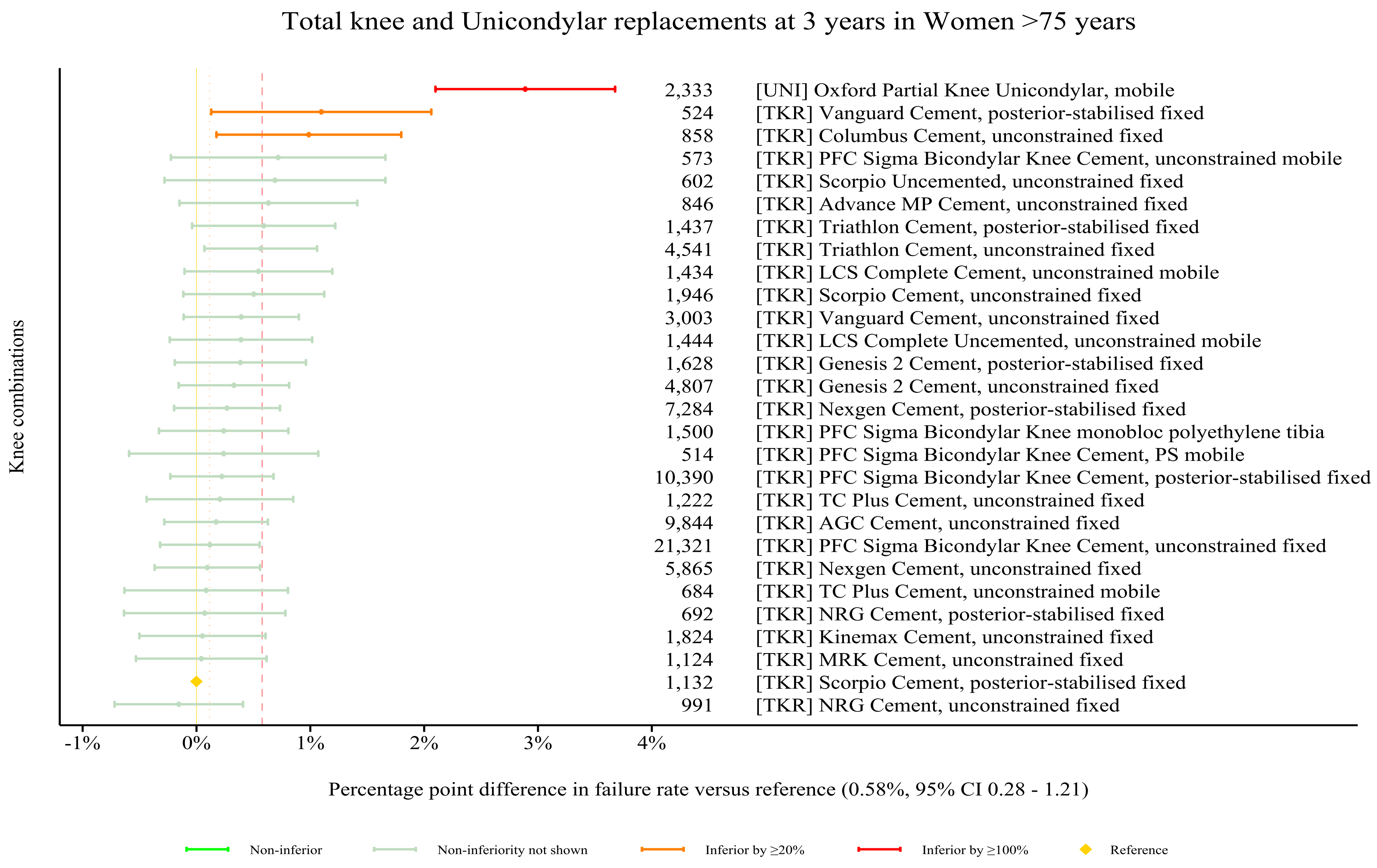

Supplementary Figure 29: Difference in cumulative revision of knee implants compared to a contemporary reference at 5 years in women aged over 75 years, using all total knee and unicondylar replacements with ≥500 procedures remaining at risk.

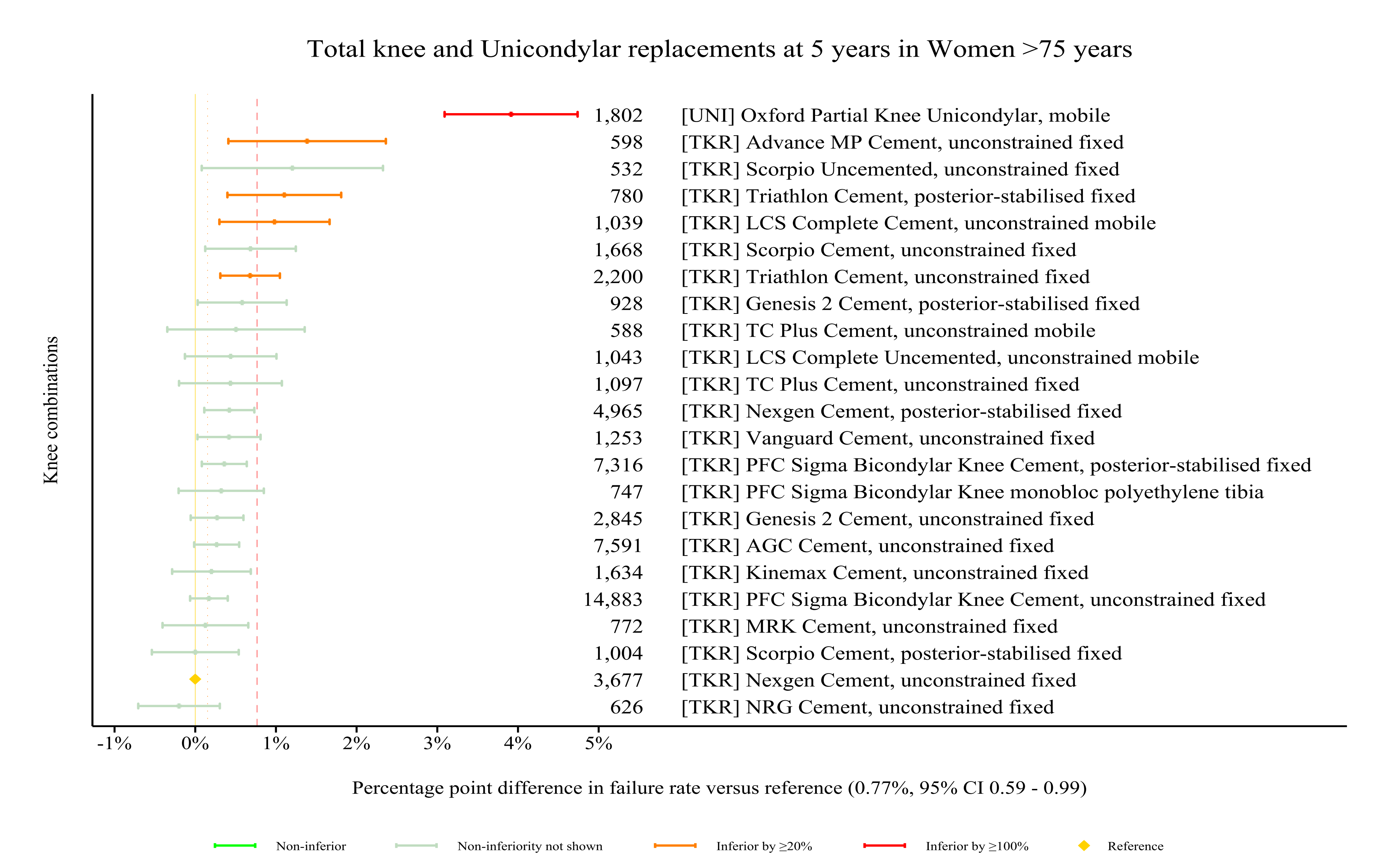

Supplementary Figure 30: Difference in cumulative revision of knee implants compared to a contemporary reference at 7 years in women aged over 75 years, using all total knee and unicondylar replacements with ≥500 procedures remaining at risk.

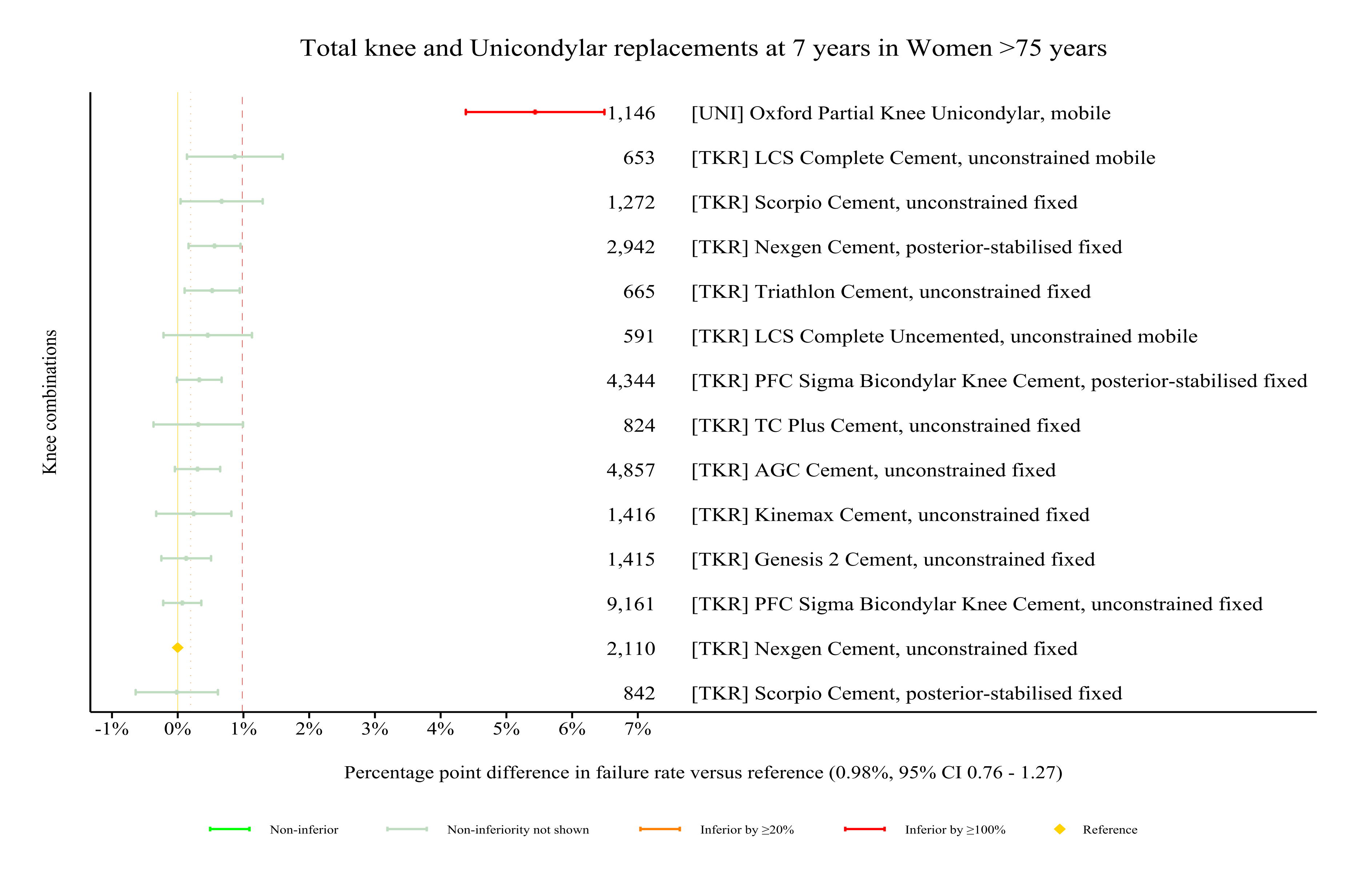

Supplementary Figure 31: Difference in cumulative revision of knee implants compared to a contemporary reference at 10 years in women aged over 75 years, using all total knee and unicondylar replacements with ≥500 procedures remaining at risk.

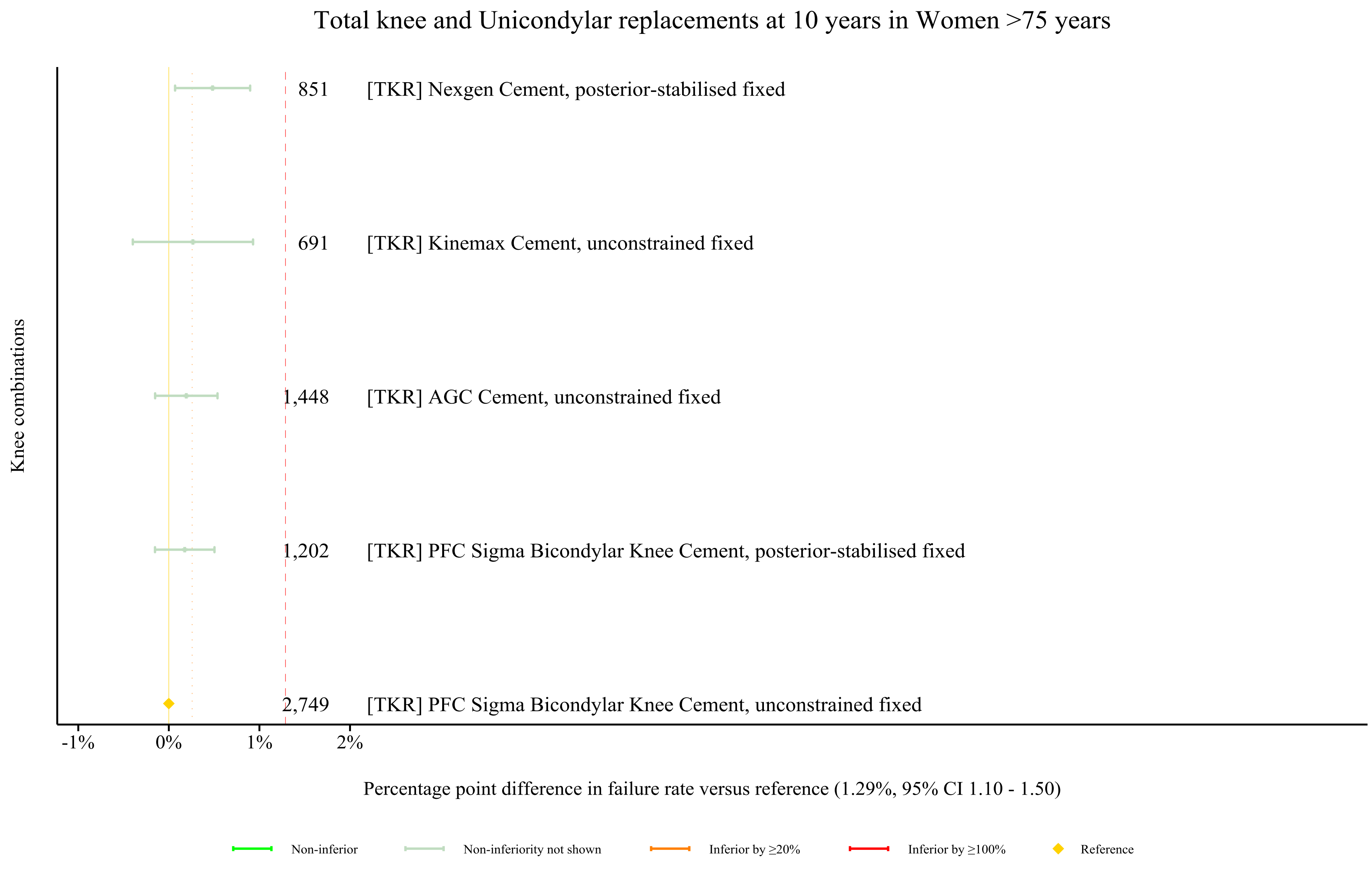

Supplementary table 1: Difference in cumulative percentage revision of knee implants compared to a contemporary benchmark at 3 years post primary for knee replacements with ≥250 procedures remaining at risk

Knee brand, bearing and constraint	Number at risk	Cumulative failure (%)	Difference in failure (%)	95% CI	Equivalence status	p-value
[TKR] NexGen Cement, unconstrained fixed	34,558	1.10	[REFERENCE]			
[TKR] ACS Uncemented, unconstrained fixed	448	3.42	2.32	[0.67 , 3.97]	Inferior by ≥20%	0.003
[TKR] ACS Uncemented, unconstrained mobile	428	3.20	2.10	[0.55 , 3.65]	Inferior by ≥20%	0.004
[TKR] AGC Cement, posterior-stabilised fixed	502	1.90	0.80	[-0.37 , 1.98]	Non-inferiority not shown	0.090
[TKR] AGC Cement, unconstrained fixed	51,355	1.43	0.33	[0.19 , 0.47]	Non-inferiority not shown	<0.001
[TKR] AGC Hybrid, unconstrained fixed	861	2.28	1.18	[0.21 , 2.15]	Non-inferiority not shown	0.008
[TKR] AGC Uncemented, unconstrained fixed	1,081	4.08	2.98	[1.83 , 4.12]	Inferior by ≥100%	<0.001
[TKR] AMK Cement, unconstrained fixed	378	0.48	-0.61	[-1.29 , 0.06]	Non-inferior	0.037
[TKR] Advance MP Cement, unconstrained fixed	5,549	2.04	0.94	[0.59 , 1.29]	Inferior by ≥20%	<0.001
[TKR] Advance MP Stature Cement, unconstrained fixed	829	1.83	0.73	[-0.07 , 1.53]	Non-inferiority not shown	0.037
[TKR] Advance PS Cement, posterior-stabilised fixed	791	2.39	1.29	[0.32 , 2.27]	Inferior by ≥20%	0.005
[TKR] Alpina Hybrid, unconstrained fixed	441	1.94	0.84	[-0.42 , 2.10]	Non-inferiority not shown	0.095
[TKR] Birmingham Knee Replacement Cement, posterior-stabilised mobile	420	1.86	0.76	[-0.30 , 1.81]	Non-inferiority not shown	0.080
[TKR] Columbus Cement, unconstrained fixed	5,128	1.78	0.68	[0.36 , 1.01]	Inferior by ≥20%	<0.001
[TKR] E-Motion Bicondylar Knee Cement, unconstrained mobile	702	3.01	1.92	[0.78 , 3.05]	Inferior by ≥20%	<0.001
[TKR] E-Motion Bicondylar Knee Uncemented, unconstrained mobile	1,393	2.03	0.93	[0.25 , 1.61]	Inferior by ≥20%	0.004
[TKR] FS Cement, unconstrained fixed	741	1.54	0.45	[-0.43 , 1.32]	Non-inferiority not shown	0.158
[TKR] Genesis 2 Cement, posterior-stabilised fixed	7,864	1.75	0.65	[0.38 , 0.91]	Inferior by ≥20%	<0.001
[TKR] Genesis 2 Cement, unconstrained fixed	25,556	1.27	0.17	[0.02 , 0.33]	Non-inferiority not shown	0.014
[TKR] Genesis 2 Cement, unconstrained mobile	887	1.74	0.64	[-0.21 , 1.49]	Non-inferiority not shown	0.070
[TKR] Genesis 2 Oxinium Cement, posterior-stabilised fixed	1,681	2.95	1.85	[1.12 , 2.57]	Inferior by ≥100%	<0.001
[TKR] Genesis 2 Oxinium Cement, unconstrained fixed	3,633	1.94	0.84	[0.42 , 1.27]	Inferior by ≥20%	<0.001
[TKR] Innex Uncemented, unconstrained mobile	358	1.62	0.52	[-0.77 , 1.80]	Non-inferiority not shown	0.216
[TKR] Insall-Burstein 2 Cement, posterior-stabilised fixed	2,226	1.47	0.37	[-0.13 , 0.87]	Non-inferiority not shown	0.072
[TKR] Journey Oxinium Cement, posterior-stabilised fixed	744	4.59	3.49	[2.03 , 4.96]	Inferior by ≥100%	<0.001
[TKR] Kinemax Cement, unconstrained fixed	9,935	1.78	0.68	[0.41 , 0.95]	Inferior by ≥20%	<0.001
[TKR] LCS Cement, unconstrained mobile	611	1.39	0.29	[-0.62 , 1.20]	Non-inferiority not shown	0.264
[TKR] LCS Complete Cement, unconstrained mobile	8,451	1.62	0.52	[0.25 , 0.80]	Inferior by ≥20%	<0.001
[TKR] LCS Complete Hybrid, unconstrained mobile	381	1.68	0.59	[-0.47 , 1.64]	Non-inferiority not shown	0.137

For peer review only

[TKR] LCS Complete Uncemented, unconstrained mobile	10,293	1.75	0.65	[0.40 , 0.91]	Inferior by ≥20%	<0.001
[TKR] LCS Uncemented, unconstrained mobile	1,237	1.87	0.77	[0.02 , 1.52]	Non-inferiority not shown	0.022
[TKR] Legion Cement, unconstrained fixed	395	1.50	0.40	[-0.53 , 1.33]	Non-inferiority not shown	0.202
[TKR] MBK Cement, unconstrained mobile	372	1.30	0.20	[-0.94 , 1.33]	Non-inferiority not shown	0.365
[TKR] MRK Cement, unconstrained fixed	6,170	1.20	0.10	[-0.16 , 0.35]	Non-inferiority not shown	0.231
[TKR] Maxim Cement, posterior-stabilised fixed	582	1.48	0.38	[-0.58 , 1.35]	Non-inferiority not shown	0.220
[TKR] Maxim Cement, unconstrained fixed	1,244	1.48	0.38	[-0.29 , 1.04]	Non-inferiority not shown	0.134
[TKR] NRG Cement, posterior-stabilised fixed	4,141	1.71	0.61	[0.22 , 1.00]	Inferior by ≥20%	0.001
[TKR] NRG Cement, unconstrained fixed	5,860	1.48	0.38	[0.08 , 0.68]	Non-inferiority not shown	0.007
[TKR] Natural Knee II Cement, unconstrained fixed	2,481	1.37	0.27	[-0.18 , 0.73]	Non-inferiority not shown	0.120
[TKR] NexGen Cement, posterior-stabilised mobile	813	2.83	1.73	[0.69 , 2.77]	Inferior by ≥20%	0.001
[TKR] NexGen Cement, posterior-stabilised fixed	40,703	1.61	0.51	[0.36 , 0.65]	Inferior by ≥20%	<0.001
[TKR] NexGen Hinge Type Cement, unconstrained mobile	292	1.65	0.56	[-0.69 , 1.80]	Non-inferiority not shown	0.191
[TKR] NexGen Hybrid, unconstrained fixed	963	1.07	-0.03	[-0.66 , 0.61]	Non-inferiority not shown	0.466
[TKR] NexGen LCCK Cement, constrained condylar	282	2.77	1.67	[0.01 , 3.33]	Non-inferiority not shown	0.024
[TKR] NexGen Uncemented, posterior-stabilised fixed	1,424	1.72	0.62	[-0.00 , 1.24]	Non-inferiority not shown	0.025
[TKR] NexGen Uncemented, unconstrained fixed	3,540	2.55	1.45	[0.95 , 1.96]	Inferior by ≥20%	<0.001
[TKR] NexGen monobloc polyethylene tibia	530	0.67	-0.43	[-1.03 , 0.18]	Non-inferior	0.083
[TKR] Optetrak Cement, posterior-stabilised fixed	1,495	2.66	1.56	[0.76 , 2.36]	Inferior by ≥20%	<0.001
[TKR] Optetrak Cement, unconstrained fixed	360	3.73	2.63	[0.77 , 4.48]	Inferior by ≥20%	0.003
[TKR] PFC Sigma Bicondylar Knee Cement, posterior-stabilised mobile	5,733	2.12	1.02	[0.65 , 1.38]	Inferior by ≥20%	<0.001
[TKR] PFC Sigma Bicondylar Knee Cement, constrained condylar	560	1.73	0.64	[-0.31 , 1.58]	Non-inferiority not shown	0.094
[TKR] PFC Sigma Bicondylar Knee Cement, posterior-stabilised fixed	54,952	1.51	0.41	[0.28 , 0.55]	Inferior by ≥20%	<0.001
[TKR] PFC Sigma Bicondylar Knee Cement, unconstrained fixed	126,134	1.25	0.15	[0.04 , 0.26]	Non-inferiority not shown	0.005
[TKR] PFC Sigma Bicondylar Knee Cement, unconstrained mobile	6,571	1.93	0.83	[0.51 , 1.16]	Inferior by ≥20%	<0.001
[TKR] PFC Sigma Bicondylar Knee Hybrid, unconstrained fixed	1,532	1.18	0.08	[-0.45 , 0.62]	Non-inferiority not shown	0.381
[TKR] PFC Sigma Bicondylar Knee Uncemented, unconstrained mobile	604	1.44	0.34	[-0.51 , 1.20]	Non-inferiority not shown	0.216
[TKR] PFC Sigma Bicondylar Knee monobloc polyethylene tibia	5,365	1.30	0.21	[-0.07 , 0.48]	Non-inferiority not shown	0.070
[TKR] Profix Cement, unconstrained fixed	883	2.05	0.95	[0.03 , 1.86]	Non-inferiority not shown	0.021
[TKR] Profix Oxinium monobloc polyethylene tibia	570	3.03	1.93	[0.55 , 3.32]	Inferior by ≥20%	0.003
[TKR] Profix Uncemented, unconstrained fixed	2,137	1.26	0.16	[-0.32 , 0.63]	Non-inferiority not shown	0.258
[TKR] Profix monobloc polyethylene tibia	651	0.72	-0.37	[-1.01 , 0.27]	Non-inferiority not shown	0.126
[TKR] Rotaglide + Cement, unconstrained mobile	1,576	2.83	1.73	[0.92 , 2.53]	Inferior by ≥20%	<0.001
[TKR] Rotaglide Cement, unconstrained mobile	1,051	2.05	0.96	[0.15 , 1.76]	Non-inferiority not shown	0.010
[TKR] Scorpio Cement, posterior-stabilised mobile	1,289	1.49	0.39	[-0.26 , 1.05]	Non-inferiority not shown	0.121
[TKR] Scorpio Cement, posterior-stabilised fixed	5,673	1.59	0.49	[0.16 , 0.82]	Non-inferiority not shown	0.002

For peer review only

[TKR] Scorpio Cement, unconstrained fixed	10,083	1.85	0.75	[0.48 , 1.03]	Inferior by ≥20%	<0.001
[TKR] Scorpio Cement, unconstrained mobile	1,094	2.54	1.44	[0.52 , 2.36]	Inferior by ≥20%	0.001
[TKR] Scorpio Hybrid, unconstrained fixed	1,005	1.80	0.70	[-0.11 , 1.50]	Non-inferiority not shown	0.045
[TKR] Scorpio Uncemented, unconstrained fixed	3,530	1.79	0.69	[0.26 , 1.13]	Inferior by ≥20%	0.001
[TKR] TC Plus Cement, unconstrained fixed	7,501	1.89	0.79	[0.47 , 1.10]	Inferior by ≥20%	<0.001
[TKR] TC Plus Cement, unconstrained mobile	4,385	1.44	0.35	[-0.01 , 0.70]	Non-inferiority not shown	0.027
[TKR] TC Plus Uncemented, unconstrained mobile	1,439	1.52	0.42	[-0.17 , 1.00]	Non-inferiority not shown	0.083
[TKR] Triathlon Cement, posterior-stabilised fixed	8,196	1.73	0.63	[0.37 , 0.89]	Inferior by ≥20%	<0.001
[TKR] Triathlon Cement, unconstrained fixed	29,202	1.48	0.38	[0.23 , 0.53]	Inferior by ≥20%	<0.001
[TKR] Triathlon Uncemented, unconstrained fixed	742	2.75	1.66	[0.63 , 2.69]	Inferior by ≥20%	0.001
[TKR] Vanguard Cement, constrained condylar	436	1.08	-0.02	[-0.72 , 0.69]	Non-inferiority not shown	0.479
[TKR] Vanguard Cement, posterior-stabilised fixed	2,988	1.91	0.81	[0.41 , 1.21]	Inferior by ≥20%	<0.001
[TKR] Vanguard Cement, unconstrained fixed	19,392	1.37	0.27	[0.11 , 0.44]	Non-inferiority not shown	0.001
[TKR] balanSys CR cemented Cement, unconstrained fixed	293	1.72	0.63	[-0.75 , 2.00]	Non-inferiority not shown	0.186
[UNI] AMC/Uniglide Unicondylar, fixed	1,070	2.98	1.88	[0.92 , 2.85]	Inferior by ≥20%	<0.001
[UNI] AMC/Uniglide Unicondylar, mobile	1,195	8.47	7.37	[5.91 , 8.84]	Inferior by ≥100%	<0.001
[UNI] EIUS Unicondylar, fixed	314	9.39	8.29	[5.24 , 11.34]	Inferior by ≥100%	<0.001
[UNI] Genesis Uni Oxinium Unicondylar, fixed	439	3.61	2.51	[0.87 , 4.15]	Inferior by ≥20%	0.001
[UNI] MG Uni Unicondylar, fixed	2,206	3.96	2.86	[2.06 , 3.66]	Inferior by ≥100%	<0.001
[UNI] Oxford Partial Knee Unicondylar, fixed	378	3.57	2.47	[0.79 , 4.16]	Inferior by ≥20%	0.002
[UNI] Oxford Partial Knee Unicondylar, mobile	37,382	4.07	2.98	[2.77 , 3.18]	Inferior by ≥100%	<0.001
[UNI] Physica ZUK Unicondylar, fixed	4,708	2.45	1.35	[0.96 , 1.73]	Inferior by ≥20%	<0.001
[UNI] Preservation Unicondylar, fixed	1,105	6.89	5.79	[4.36 , 7.23]	Inferior by ≥100%	<0.001
[UNI] Sigma HP Unicondylar, fixed	3,561	3.56	2.46	[1.95 , 2.97]	Inferior by ≥100%	<0.001
[UNI] Sled Unicondylar, fixed	682	6.23	5.14	[3.44 , 6.83]	Inferior by ≥100%	<0.001
[UNI] Triathlon Uni Unicondylar, fixed	433	5.69	4.59	[2.76 , 6.43]	Inferior by ≥100%	<0.001

For peer review only

Supplementary table 2: Difference in cumulative percentage revision of knee implants compared to a contemporary benchmark at 5 years post primary for knee replacements with ≥250 procedures remaining at risk

Knee brand, bearing and constraint	Number at risk	Cumulative failure (%)	Difference in failure (%)	95% CI	Equivalence status	p-value
[TKR] Profix Uncemented, unconstrained fixed	1,910	1.54	[REFERENCE]			
[TKR] ACS Uncemented, unconstrained fixed	431	3.86	2.32	[0.49 , 4.14]	Inferior by ≥20%	0.006
[TKR] ACS Uncemented, unconstrained mobile	347	3.66	2.12	[0.37 , 3.87]	Inferior by ≥20%	0.009
[TKR] AGC Cement, posterior-stabilised fixed	440	2.71	1.17	[-0.33 , 2.66]	Non-inferiority not shown	0.063
[TKR] AGC Cement, unconstrained fixed	40,694	2.03	0.49	[-0.04 , 1.02]	Non-inferiority not shown	0.035
[TKR] AGC Hybrid, unconstrained fixed	799	2.86	1.32	[0.12 , 2.52]	Non-inferiority not shown	0.016
[TKR] AGC Uncemented, unconstrained fixed	969	5.10	3.56	[2.18 , 4.94]	Inferior by ≥100%	<0.001
[TKR] AMK Cement, unconstrained fixed	350	1.02	-0.52	[-1.64 , 0.60]	Non-inferiority not shown	0.180
[TKR] Advance MP Cement, unconstrained fixed	3,988	2.79	1.25	[0.59 , 1.91]	Inferior by ≥20%	<0.001
[TKR] Advance MP Stature Cement, unconstrained fixed	452	3.20	1.66	[0.36 , 2.96]	Inferior by ≥20%	0.006
[TKR] Advance PS Cement, posterior-stabilised fixed	619	3.19	1.65	[0.38 , 2.91]	Inferior by ≥20%	0.005
[TKR] Alpina Hybrid, unconstrained fixed	390	2.19	0.65	[-0.79 , 2.09]	Non-inferiority not shown	0.189
[TKR] Birmingham Knee Replacement Cement, posterior-stabilised mobile	270	2.76	1.21	[-0.34 , 2.76]	Non-inferiority not shown	0.062
[TKR] Columbus Cement, unconstrained fixed	2,818	2.51	0.97	[0.31 , 1.63]	Inferior by ≥20%	0.002
[TKR] E-Motion Bicondylar Knee Cement, unconstrained mobile	354	3.92	2.38	[0.91 , 3.85]	Inferior by ≥20%	0.001
[TKR] E-Motion Bicondylar Knee Uncemented, unconstrained mobile	1,061	2.85	1.31	[0.32 , 2.29]	Inferior by ≥20%	0.005
[TKR] FS Cement, unconstrained fixed	701	2.09	0.54	[-0.59 , 1.68]	Non-inferiority not shown	0.174
[TKR] Genesis 2 Cement, posterior-stabilised fixed	4,569	2.47	0.93	[0.32 , 1.54]	Inferior by ≥20%	0.001
[TKR] Genesis 2 Cement, unconstrained fixed	15,556	1.75	0.21	[-0.33 , 0.75]	Non-inferiority not shown	0.222
[TKR] Genesis 2 Cement, unconstrained mobile	774	2.31	0.77	[-0.34 , 1.88]	Non-inferiority not shown	0.086
[TKR] Genesis 2 Oxinium Cement, posterior-stabilised fixed	1,030	4.23	2.69	[1.63 , 3.74]	Inferior by ≥100%	<0.001
[TKR] Genesis 2 Oxinium Cement, unconstrained fixed	2,660	3.03	1.49	[0.74 , 2.24]	Inferior by ≥20%	<0.001
[TKR] Innex Uncemented, unconstrained mobile	348	2.45	0.91	[-0.76 , 2.57]	Non-inferiority not shown	0.142
[TKR] Insall-Burstein 2 Cement, posterior-stabilised fixed	2,067	2.74	1.20	[0.35 , 2.05]	Inferior by ≥20%	0.003
[TKR] Journey Oxinium Cement, posterior-stabilised fixed	513	6.26	4.72	[2.93 , 6.52]	Inferior by ≥100%	<0.001
[TKR] Kinemax Cement, unconstrained fixed	9,295	2.70	1.15	[0.55 , 1.76]	Inferior by ≥20%	<0.001
[TKR] LCS Cement, unconstrained mobile	568	1.88	0.34	[-0.83 , 1.52]	Non-inferiority not shown	0.285
[TKR] LCS Complete Cement, unconstrained mobile	6,533	2.73	1.19	[0.57 , 1.81]	Inferior by ≥20%	<0.001
[TKR] LCS Complete Hybrid, unconstrained mobile	268	2.35	0.81	[-0.68 , 2.29]	Non-inferiority not shown	0.143

For peer review only

[TKR] LCS Complete Uncemented, unconstrained mobile	7,677	2.50	0.95	[0.36 , 1.55]	Inferior by ≥20%	0.001
[TKR] LCS Uncemented, unconstrained mobile	1,159	2.44	0.89	[-0.10 , 1.89]	Non-inferiority not shown	0.039
[TKR] MBK Cement, unconstrained mobile	360	2.63	1.09	[-0.60 , 2.78]	Non-inferiority not shown	0.103
[TKR] MRK Cement, unconstrained fixed	4,250	1.64	0.09	[-0.50 , 0.69]	Non-inferiority not shown	0.377
[TKR] Maxim Cement, posterior-stabilised fixed	480	3.12	1.58	[0.06 , 3.09]	Non-inferiority not shown	0.021
[TKR] Maxim Cement, unconstrained fixed	1,109	2.13	0.59	[-0.36 , 1.54]	Non-inferiority not shown	0.111
[TKR] NRG Cement, posterior-stabilised fixed	3,075	2.40	0.86	[0.17 , 1.55]	Non-inferiority not shown	0.007
[TKR] NRG Cement, unconstrained fixed	3,999	2.36	0.82	[0.17 , 1.46]	Non-inferiority not shown	0.006
[TKR] Natural Knee II Cement, unconstrained fixed	1,950	2.21	0.67	[-0.11 , 1.44]	Non-inferiority not shown	0.046
[TKR] NexGen Cement, posterior-stabilised mobile	680	3.60	2.06	[0.75 , 3.37]	Inferior by ≥20%	0.001
[TKR] NexGen Cement, posterior-stabilised fixed	28,335	2.54	0.99	[0.46 , 1.53]	Inferior by ≥20%	<0.001
[TKR] NexGen Cement, unconstrained fixed	21,242	1.60	0.05	[-0.48 , 0.58]	Non-inferiority not shown	0.423
[TKR] NexGen Hybrid, unconstrained fixed	832	1.60	0.06	[-0.87 , 1.00]	Non-inferiority not shown	0.450
[TKR] NexGen Uncemented, posterior-stabilised fixed	1,082	2.20	0.66	[-0.23 , 1.54]	Non-inferiority not shown	0.073
[TKR] NexGen Uncemented, unconstrained fixed	3,059	3.18	1.63	[0.87 , 2.40]	Inferior by ≥20%	<0.001
[TKR] Optetrak Cement, posterior-stabilised fixed	1,278	4.56	3.02	[1.85 , 4.19]	Inferior by ≥100%	<0.001
[TKR] PFC Sigma Bicondylar Knee Cement, posterior-stabilised mobile	4,255	2.79	1.25	[0.59 , 1.91]	Inferior by ≥20%	<0.001
[TKR] PFC Sigma Bicondylar Knee Cement, constrained condylar	366	2.56	1.02	[-0.32 , 2.36]	Non-inferiority not shown	0.068
[TKR] PFC Sigma Bicondylar Knee Cement, posterior-stabilised fixed	39,518	2.09	0.54	[0.01 , 1.07]	Non-inferiority not shown	0.022
[TKR] PFC Sigma Bicondylar Knee Cement, unconstrained fixed	88,618	1.71	0.16	[-0.36 , 0.68]	Non-inferiority not shown	0.270
[TKR] PFC Sigma Bicondylar Knee Cement, unconstrained mobile	5,415	2.73	1.19	[0.54 , 1.83]	Inferior by ≥20%	<0.001
[TKR] PFC Sigma Bicondylar Knee Hybrid, unconstrained fixed	1,389	1.79	0.25	[-0.59 , 1.08]	Non-inferiority not shown	0.280
[TKR] PFC Sigma Bicondylar Knee Uncemented, unconstrained mobile	450	1.78	0.24	[-0.86 , 1.34]	Non-inferiority not shown	0.335
[TKR] PFC Sigma Bicondylar Knee monobloc polyethylene tibia	2,383	1.65	0.10	[-0.50 , 0.71]	Non-inferiority not shown	0.370
[TKR] Profix Cement, unconstrained fixed	813	3.20	1.65	[0.40 , 2.91]	Inferior by ≥20%	0.005
[TKR] Profix Oxinium monobloc polyethylene tibia	553	3.55	2.01	[0.43 , 3.58]	Inferior by ≥20%	0.006
[TKR] Profix monobloc polyethylene tibia	600	1.35	-0.19	[-1.21 , 0.83]	Non-inferiority not shown	0.356
[TKR] Rotaglide + Cement, unconstrained mobile	1,454	3.65	2.10	[1.06 , 3.15]	Inferior by ≥20%	<0.001
[TKR] Rotaglide Cement, unconstrained mobile	777	3.29	1.75	[0.57 , 2.92]	Inferior by ≥20%	0.002
[TKR] Scorpio Cement, posterior-stabilised mobile	1,178	2.19	0.65	[-0.30 , 1.59]	Non-inferiority not shown	0.089
[TKR] Scorpio Cement, posterior-stabilised fixed	5,265	2.37	0.83	[0.18 , 1.48]	Non-inferiority not shown	0.006
[TKR] Scorpio Cement, unconstrained fixed	9,046	2.61	1.06	[0.46 , 1.66]	Inferior by ≥20%	<0.001
[TKR] Scorpio Cement, unconstrained mobile	1,036	3.63	2.08	[0.88 , 3.29]	Inferior by ≥20%	<0.001
[TKR] Scorpio Hybrid, unconstrained fixed	938	2.51	0.96	[-0.12 , 2.05]	Non-inferiority not shown	0.040
[TKR] Scorpio Uncemented, unconstrained fixed	3,140	2.49	0.95	[0.22 , 1.67]	Non-inferiority not shown	0.005
[TKR] TC Plus Cement, unconstrained fixed	6,952	2.56	1.01	[0.39 , 1.64]	Inferior by ≥20%	0.001

For peer review only

[TKR] TC Plus Cement, unconstrained mobile	3,999	2.00	0.46	[-0.20 , 1.11]	Non-inferiority not shown	0.085
[TKR] TC Plus Uncemented, unconstrained mobile	1,145	2.18	0.63	[-0.25 , 1.52]	Non-inferiority not shown	0.081
[TKR] Triathlon Cement, posterior-stabilised fixed	4,605	2.53	0.98	[0.38 , 1.59]	Inferior by ≥20%	0.001
[TKR] Triathlon Cement, unconstrained fixed	14,360	2.01	0.47	[-0.07 , 1.00]	Non-inferiority not shown	0.044
[TKR] Triathlon Uncemented, unconstrained fixed	418	3.39	1.85	[0.54 , 3.15]	Inferior by ≥20%	0.003
[TKR] Vanguard Cement, posterior-stabilised fixed	1,135	2.75	1.21	[0.45 , 1.97]	Inferior by ≥20%	0.001
[TKR] Vanguard Cement, unconstrained fixed	8,329	2.02	0.48	[-0.07 , 1.02]	Non-inferiority not shown	0.045
[UNI] AMC/Uniglide Unicondylar, fixed	756	4.41	2.87	[1.55 , 4.18]	Inferior by ≥100%	<0.001
[UNI] AMC/Uniglide Unicondylar, mobile	997	10.16	8.62	[6.93 , 10.30]	Inferior by ≥100%	<0.001
[UNI] EIUS Unicondylar, fixed	289	14.92	13.38	[9.60 , 17.16]	Inferior by ≥100%	<0.001
[UNI] Genesis Uni Oxinium Unicondylar, fixed	330	7.05	5.51	[3.07 , 7.94]	Inferior by ≥100%	<0.001
[UNI] MG Uni Unicondylar, fixed	2,082	5.95	4.41	[3.31 , 5.51]	Inferior by ≥100%	<0.001
[UNI] Oxford Partial Knee Unicondylar, fixed	284	4.65	3.10	[1.07 , 5.13]	Inferior by ≥20%	0.001
[UNI] Oxford Partial Knee Unicondylar, mobile	28,136	6.13	4.59	[4.02 , 5.15]	Inferior by ≥100%	<0.001
[UNI] Physica ZUK Unicondylar, fixed	2,392	3.94	2.40	[1.65 , 3.15]	Inferior by ≥100%	<0.001
[UNI] Preservation Unicondylar, fixed	1,045	10.21	8.67	[6.87 , 10.46]	Inferior by ≥100%	<0.001
[UNI] Sigma HP Unicondylar, fixed	1,634	5.01	3.47	[2.61 , 4.32]	Inferior by ≥100%	<0.001
[UNI] Sled Unicondylar, fixed	585	9.83	8.29	[6.09 , 10.48]	Inferior by ≥100%	<0.001

Supplementary table 3: Difference in cumulative percentage revision of knee implants compared to a contemporary benchmark at 7 years post primary for knee replacements with ≥250 procedures remaining at risk

Knee brand, bearing and constraint	Number at risk	Cumulative failure (%)	Difference in failure (%)	95% CI	Equivalence status	p-value
[TKR] Profix Uncemented, unconstrained fixed	1,501	1.77	[REFERENCE]			
[TKR] ACS Uncemented, unconstrained fixed	389	5.01	3.24	[1.16 , 5.31]	Inferior by ≥20%	0.001
[TKR] AGC Cement, posterior-stabilised fixed	355	3.97	2.19	[0.34 , 4.05]	Non-inferiority not shown	0.010
[TKR] AGC Cement, unconstrained fixed	27,288	2.56	0.79	[0.21 , 1.37]	Non-inferiority not shown	0.004
[TKR] AGC Hybrid, unconstrained fixed	541	3.25	1.48	[0.19 , 2.78]	Non-inferiority not shown	0.013
[TKR] AGC Uncemented, unconstrained fixed	710	5.97	4.20	[2.69 , 5.71]	Inferior by ≥100%	<0.001
[TKR] AMK Cement, unconstrained fixed	318	1.02	-0.75	[-1.90 , 0.39]	Non-inferiority not shown	0.098
[TKR] Advance MP Cement, unconstrained fixed	2,588	3.51	1.74	[0.99 , 2.49]	Inferior by ≥20%	<0.001
[TKR] Advance PS Cement, posterior-stabilised fixed	425	4.29	2.52	[0.97 , 4.07]	Inferior by ≥20%	0.001
[TKR] Alpina Hybrid, unconstrained fixed	317	3.84	2.07	[0.13 , 4.01]	Non-inferiority not shown	0.018
[TKR] Columbus Cement, unconstrained fixed	1,482	2.79	1.02	[0.29 , 1.75]	Non-inferiority not shown	0.003
[TKR] E-Motion Bicondylar Knee Uncemented, unconstrained mobile	732	3.86	2.09	[0.89 , 3.29]	Inferior by ≥20%	<0.001
[TKR] FS Cement, unconstrained fixed	643	2.66	0.89	[-0.39 , 2.17]	Non-inferiority not shown	0.087
[TKR] Genesis 2 Cement, posterior-stabilised fixed	2,275	2.96	1.19	[0.50 , 1.88]	Inferior by ≥20%	<0.001
[TKR] Genesis 2 Cement, unconstrained fixed	8,056	2.17	0.39	[-0.20 , 0.99]	Non-inferiority not shown	0.097
[TKR] Genesis 2 Cement, unconstrained mobile	549	2.90	1.12	[-0.14 , 2.38]	Non-inferiority not shown	0.040
[TKR] Genesis 2 Oxinium Cement, posterior-stabilised fixed	500	5.81	4.04	[2.64 , 5.44]	Inferior by ≥100%	<0.001
[TKR] Genesis 2 Oxinium Cement, unconstrained fixed	1,664	3.51	1.74	[0.90 , 2.57]	Inferior by ≥20%	<0.001
[TKR] Innex Uncemented, unconstrained mobile	314	2.45	0.68	[-1.00 , 2.36]	Non-inferiority not shown	0.214
[TKR] Insall-Burstein 2 Cement, posterior-stabilised fixed	1,880	3.43	1.65	[0.71 , 2.60]	Inferior by ≥20%	<0.001
[TKR] Journey Oxinium Cement, posterior-stabilised fixed	334	6.26	4.49	[2.68 , 6.30]	Inferior by ≥100%	<0.001
[TKR] Kinemax Cement, unconstrained fixed	8,560	3.53	1.76	[1.09 , 2.43]	Inferior by ≥20%	<0.001
[TKR] LCS Cement, unconstrained mobile	514	2.61	0.84	[-0.55 , 2.23]	Non-inferiority not shown	0.117
[TKR] LCS Complete Cement, unconstrained mobile	4,216	3.44	1.67	[0.98 , 2.36]	Inferior by ≥20%	<0.001
[TKR] LCS Complete Uncemented, unconstrained mobile	4,724	2.92	1.15	[0.50 , 1.80]	Inferior by ≥20%	<0.001
[TKR] LCS Uncemented, unconstrained mobile	1,092	2.44	0.67	[-0.35 , 1.68]	Non-inferiority not shown	0.100
[TKR] MBK Cement, unconstrained mobile	347	3.18	1.41	[-0.45 , 3.26]	Non-inferiority not shown	0.069
[TKR] MRK Cement, unconstrained fixed	2,775	2.28	0.51	[-0.17 , 1.19]	Non-inferiority not shown	0.072
[TKR] Maxim Cement, posterior-stabilised fixed	353	3.60	1.83	[0.16 , 3.49]	Non-inferiority not shown	0.016

For peer review only

[TKR] Maxim Cement, unconstrained fixed	881	3.05	1.28	[0.14 , 2.42]	Non-inferiority not shown	0.014
[TKR] NRG Cement, posterior-stabilised fixed	1,438	2.86	1.09	[0.32 , 1.86]	Non-inferiority not shown	0.003
[TKR] NRG Cement, unconstrained fixed	2,207	2.83	1.06	[0.33 , 1.78]	Non-inferiority not shown	0.002
[TKR] Natural Knee II Cement, unconstrained fixed	1,437	3.20	1.42	[0.50 , 2.35]	Inferior by ≥20%	0.001
[TKR] Nexgen Cement, PS mobile	551	5.12	3.35	[1.74 , 4.96]	Inferior by ≥20%	<0.001
[TKR] Nexgen Cement, posterior-stabilised fixed	17,857	3.31	1.54	[0.95 , 2.13]	Inferior by ≥20%	<0.001
[TKR] Nexgen Cement, unconstrained fixed	12,329	2.12	0.35	[-0.24 , 0.93]	Non-inferiority not shown	0.122
[TKR] Nexgen Hybrid, unconstrained fixed	574	2.15	0.38	[-0.72 , 1.48]	Non-inferiority not shown	0.250
[TKR] Nexgen Uncemented, ps fixed	784	2.54	0.77	[-0.22 , 1.75]	Non-inferiority not shown	0.064
[TKR] Nexgen Uncemented, unconstrained fixed	2,528	3.58	1.81	[0.98 , 2.64]	Inferior by ≥20%	<0.001
[TKR] Optetrak Cement, posterior-stabilised fixed	809	5.43	3.66	[2.37 , 4.96]	Inferior by ≥100%	<0.001
[TKR] PFC Sigma Bicondylar Knee Cement, PS mobile	2,686	3.36	1.59	[0.85 , 2.32]	Inferior by ≥20%	<0.001
[TKR] PFC Sigma Bicondylar Knee Cement, posterior-stabilised fixed	24,658	2.44	0.67	[0.10 , 1.25]	Non-inferiority not shown	0.011
[TKR] PFC Sigma Bicondylar Knee Cement, unconstrained fixed	55,631	2.00	0.23	[-0.34 , 0.80]	Non-inferiority not shown	0.212
[TKR] PFC Sigma Bicondylar Knee Cement, unconstrained mobile	3,818	3.15	1.38	[0.67 , 2.08]	Inferior by ≥20%	<0.001
[TKR] PFC Sigma Bicondylar Knee Hybrid, unconstrained fixed	1,174	1.86	0.09	[-0.78 , 0.97]	Non-inferiority not shown	0.418
[TKR] PFC Sigma Bicondylar Knee Uncemented, unconstrained mobile	258	2.02	0.25	[-0.96 , 1.46]	Non-inferiority not shown	0.344
[TKR] PFC Sigma Bicondylar Knee monobloc polyethylene tibia	810	1.92	0.15	[-0.55 , 0.85]	Non-inferiority not shown	0.340
[TKR] Profix Cement, unconstrained fixed	693	4.20	2.43	[0.99 , 3.87]	Inferior by ≥20%	<0.001
[TKR] Profix Oxinium monobloc polyethylene tibia	471	4.10	2.32	[0.62 , 4.03]	Inferior by ≥20%	0.004
[TKR] Profix monobloc polyethylene tibia	511	1.52	-0.25	[-1.34 , 0.84]	Non-inferiority not shown	0.325
[TKR] Rotaglide + Cement, unconstrained mobile	1,222	4.22	2.45	[1.32 , 3.59]	Inferior by ≥20%	<0.001
[TKR] Rotaglide Cement, unconstrained mobile	488	3.71	1.94	[0.66 , 3.22]	Inferior by ≥20%	0.002
[TKR] Scorpio Cement, PS mobile	1,013	2.54	0.77	[-0.26 , 1.79]	Non-inferiority not shown	0.071
[TKR] Scorpio Cement, posterior-stabilised fixed	4,675	3.07	1.30	[0.58 , 2.02]	Inferior by ≥20%	<0.001
[TKR] Scorpio Cement, unconstrained fixed	7,331	3.14	1.37	[0.71 , 2.03]	Inferior by ≥20%	<0.001
[TKR] Scorpio Cement, unconstrained mobile	950	4.48	2.71	[1.37 , 4.04]	Inferior by ≥20%	<0.001
[TKR] Scorpio Hybrid, unconstrained fixed	823	3.28	1.50	[0.27 , 2.74]	Non-inferiority not shown	0.009
[TKR] Scorpio Uncemented, unconstrained fixed	2,211	3.11	1.33	[0.52 , 2.14]	Inferior by ≥20%	0.001
[TKR] TC Plus Cement, unconstrained fixed	5,337	2.98	1.20	[0.52 , 1.88]	Inferior by ≥20%	<0.001
[TKR] TC Plus Cement, unconstrained mobile	3,267	2.45	0.68	[-0.05 , 1.40]	Non-inferiority not shown	0.033
[TKR] TC Plus Uncemented, unconstrained mobile	783	2.47	0.70	[-0.27 , 1.67]	Non-inferiority not shown	0.079
[TKR] Triathlon Cement, posterior-stabilised fixed	1,580	2.87	1.10	[0.42 , 1.78]	Inferior by ≥20%	0.001
[TKR] Triathlon Cement, unconstrained fixed	4,819	2.50	0.73	[0.13 , 1.33]	Non-inferiority not shown	0.009
[TKR] Vanguard Cement, posterior-stabilised fixed	360	3.58	1.81	[0.76 , 2.86]	Inferior by ≥20%	<0.001

For peer review only

For peer review only - http://bmjopen.bmj.com/site/about/guidelines.xhtml

[TKR] Vanguard Cement, unconstrained fixed	2,491	2.36	0.59	[-0.03 , 1.20]	Non-inferiority not shown	0.031
[UNI] AMC/Uniglide Unicondylar, fixed	401	6.44	4.67	[2.95 , 6.38]	Inferior by ≥100%	<0.001
[UNI] AMC/Uniglide Unicondylar, mobile	687	12.42	10.64	[8.74 , 12.55]	Inferior by ≥100%	<0.001
[UNI] EIUS Unicondylar, fixed	280	16.41	14.64	[10.70 , 18.58]	Inferior by ≥100%	<0.001
[UNI] MG Uni Unicondylar, fixed	1,829	7.55	5.78	[4.55 , 7.01]	Inferior by ≥100%	<0.001
[UNI] Oxford Partial Knee Unicondylar, mobile	18,623	8.09	6.32	[5.69 , 6.95]	Inferior by ≥100%	<0.001
[UNI] Physica ZUK Unicondylar, fixed	1,090	5.31	3.54	[2.59 , 4.48]	Inferior by ≥100%	<0.001
[UNI] Preservation Unicondylar, fixed	940	12.95	11.18	[9.18 , 13.18]	Inferior by ≥100%	<0.001
[UNI] Sled Unicondylar, fixed	476	12.39	10.62	[8.12 , 13.12]	Inferior by ≥100%	<0.001

For peer review only

Supplementary table 4: Difference in cumulative percentage revision of knee implants compared to a contemporary benchmark at 10 years post primary for knee replacements with ≥250 procedures remaining at risk

Knee brand, bearing and constraint	Number at risk	Cumulative failure (%)	Difference in failure (%)	95% CI	Equivalence status	p-value
[TKR] PFC Sigma Bicondylar Knee Cement, unconstrained fixed	19,284	2.37	[REFERENCE]			
[TKR] AGC Cement, unconstrained fixed	9,260	3.44	1.07	[0.85 , 1.29]	Inferior by ≥20%	<0.001
[TKR] AGC Uncemented, unconstrained fixed	253	7.76	5.39	[3.55 , 7.24]	Inferior by ≥100%	<0.001
[TKR] AMK Cement, unconstrained fixed	270	1.35	-1.02	[-2.21 , 0.17]	Non-inferior	0.047
[TKR] Advance MP Cement, unconstrained fixed	722	4.25	1.88	[1.18 , 2.59]	Inferior by ≥20%	<0.001
[TKR] Genesis 2 Cement, posterior-stabilised fixed	324	3.85	1.48	[0.66 , 2.30]	Inferior by ≥20%	<0.001
[TKR] Genesis 2 Cement, unconstrained fixed	1,982	2.60	0.23	[-0.05 , 0.52]	Non-inferiority not shown	0.052
[TKR] Genesis 2 Oxinium Cement, unconstrained fixed	486	4.95	2.58	[1.63 , 3.53]	Inferior by ≥20%	<0.001
[TKR] Insall-Burstein 2 Cement, posterior-stabilised fixed	1,125	4.95	2.58	[1.62 , 3.54]	Inferior by ≥20%	<0.001
[TKR] Kinemax Cement, unconstrained fixed	5,049	4.70	2.33	[1.88 , 2.78]	Inferior by ≥20%	<0.001
[TKR] LCS Cement, unconstrained mobile	442	3.62	1.25	[-0.28 , 2.79]	Non-inferiority not shown	0.055
[TKR] LCS Complete Cement, unconstrained mobile	781	4.28	1.91	[1.35 , 2.47]	Inferior by ≥20%	<0.001
[TKR] LCS Complete Uncemented, unconstrained mobile	1,340	3.31	0.94	[0.55 , 1.34]	Inferior by ≥20%	<0.001
[TKR] LCS Uncemented, unconstrained mobile	970	2.53	0.16	[-0.71 , 1.03]	Non-inferiority not shown	0.358
[TKR] MRK Cement, unconstrained fixed	554	2.91	0.54	[-0.04 , 1.13]	Non-inferiority not shown	0.035
[TKR] Maxim Cement, unconstrained fixed	408	4.32	1.95	[0.67 , 3.24]	Inferior by ≥20%	0.001
[TKR] Natural Knee II Cement, unconstrained fixed	582	3.88	1.51	[0.60 , 2.41]	Inferior by ≥20%	0.001
[TKR] NexGen Cement, posterior-stabilised mobile	268	7.13	4.76	[2.83 , 6.69]	Inferior by ≥100%	<0.001
[TKR] NexGen Cement, posterior-stabilised fixed	5,806	4.45	2.08	[1.79 , 2.37]	Inferior by ≥20%	<0.001
[TKR] NexGen Cement, unconstrained fixed	2,888	2.56	0.19	[-0.06 , 0.45]	Non-inferior	0.069
[TKR] NexGen Hybrid, unconstrained fixed	280	2.15	-0.22	[-1.17 , 0.73]	Non-inferiority not shown	0.326
[TKR] NexGen Uncemented, posterior-stabilised fixed	251	3.12	0.75	[-0.24 , 1.75]	Non-inferiority not shown	0.069
[TKR] NexGen Uncemented, unconstrained fixed	795	3.96	1.59	[0.91 , 2.26]	Inferior by ≥20%	<0.001
[TKR] PFC Sigma Bicondylar Knee Cement, posterior-stabilised mobile	784	4.06	1.69	[1.04 , 2.34]	Inferior by ≥20%	<0.001
[TKR] PFC Sigma Bicondylar Knee Cement, posterior-stabilised fixed	7,944	3.01	0.64	[0.43 , 0.84]	Non-inferiority not shown	<0.001
[TKR] PFC Sigma Bicondylar Knee Cement, unconstrained mobile	886	3.89	1.52	[0.94 , 2.10]	Inferior by ≥20%	<0.001
[TKR] PFC Sigma Bicondylar Knee Hybrid, unconstrained fixed	637	2.09	-0.28	[-1.03 , 0.47]	Non-inferior	0.234
[TKR] Profix Cement, unconstrained fixed	295	5.03	2.66	[1.14 , 4.18]	Inferior by ≥20%	<0.001
[TKR] Profix Uncemented, unconstrained fixed	564	2.21	-0.16	[-0.85 , 0.53]	Non-inferiority not shown	0.329

For peer review only

[TKR] Rotaglide + Cement, unconstrained mobile	696	5.61	3.24	[2.02 , 4.46]	Inferior by ≥20%	<0.001
[TKR] Scorpio Cement, posterior-stabilised mobile	480	3.27	0.90	[-0.14 , 1.94]	Non-inferiority not shown	0.045
[TKR] Scorpio Cement, posterior-stabilised fixed	1,995	3.85	1.48	[0.94 , 2.03]	Inferior by ≥20%	<0.001
[TKR] Scorpio Cement, unconstrained fixed	3,299	3.83	1.46	[1.04 , 1.87]	Inferior by ≥20%	<0.001
[TKR] Scorpio Hybrid, unconstrained fixed	316	4.27	1.90	[0.53 , 3.26]	Inferior by ≥20%	0.003
[TKR] Scorpio Uncemented, unconstrained fixed	517	4.13	1.76	[0.92 , 2.61]	Inferior by ≥20%	<0.001
[TKR] TC Plus Cement, unconstrained fixed	1,294	3.54	1.17	[0.67 , 1.66]	Inferior by ≥20%	<0.001
[TKR] TC Plus Cement, unconstrained mobile	1,493	3.13	0.76	[0.20 , 1.33]	Non-inferiority not shown	0.004
[TKR] Triathlon Cement, unconstrained fixed	334	3.20	0.83	[0.38 , 1.27]	Non-inferiority not shown	<0.001
[UNI] MG Uni Unicondylar, fixed	853	10.03	7.66	[6.33 , 9.00]	Inferior by ≥100%	<0.001
[UNI] Oxford Partial Knee Unicondylar, mobile	6,190	11.54	9.17	[8.73 , 9.62]	Inferior by ≥100%	<0.001
[UNI] Preservation Unicondylar, fixed	398	15.09	12.72	[10.59 , 14.86]	Inferior by ≥100%	<0.001

For peer review only

Supplementary table 5: Difference in cumulative percentage revision of knee implants compared to a contemporary benchmark at 3 years post primary in men for knee replacements with ≥250 procedures remaining at risk

Knee brand, bearing and constraint	Number at risk	Cumulative failure (%)	Difference in failure (%)	95% CI	Equivalence status	p-value
[TKR] NexGen Cement, unconstrained fixed	14,396	1.24	[REFERENCE]			
[TKR] AGC Cement, unconstrained fixed	21,525	1.61	0.36	[0.14 , 0.58]	Non-inferiority not shown	0.001
[TKR] AGC Hybrid, unconstrained fixed	400	2.07	0.83	[-0.52 , 2.17]	Non-inferiority not shown	0.115
[TKR] AGC Uncemented, unconstrained fixed	546	3.92	2.67	[1.10 , 4.25]	Inferior by ≥20%	<0.001
[TKR] Advance MP Cement, unconstrained fixed	2,482	2.39	1.15	[0.58 , 1.71]	Inferior by ≥20%	<0.001
[TKR] Advance PS Cement, posterior-stabilised fixed	342	2.55	1.30	[-0.20 , 2.80]	Non-inferiority not shown	0.045
[TKR] Columbus Cement, unconstrained fixed	2,209	1.78	0.54	[0.05 , 1.03]	Non-inferiority not shown	0.015
[TKR] E-Motion Bicondylar Knee Cement, unconstrained mobile	252	3.17	1.92	[-0.02 , 3.87]	Non-inferiority not shown	0.026
[TKR] E-Motion Bicondylar Knee Uncemented, unconstrained mobile	703	1.65	0.40	[-0.47 , 1.27]	Non-inferiority not shown	0.183
[TKR] FS Cement, unconstrained fixed	325	1.45	0.20	[-1.07 , 1.47]	Non-inferiority not shown	0.376
[TKR] Genesis 2 Cement, posterior-stabilised fixed	3,069	2.07	0.83	[0.37 , 1.29]	Inferior by ≥20%	<0.001
[TKR] Genesis 2 Cement, unconstrained fixed	10,877	1.47	0.22	[-0.03 , 0.47]	Non-inferiority not shown	0.042
[TKR] Genesis 2 Cement, unconstrained mobile	362	2.39	1.14	[-0.41 , 2.69]	Non-inferiority not shown	0.074
[TKR] Genesis 2 Oxinium Cement, posterior-stabilised fixed	700	3.69	2.44	[1.19 , 3.70]	Inferior by ≥20%	<0.001
[TKR] Genesis 2 Oxinium Cement, unconstrained fixed	1,574	2.48	1.23	[0.50 , 1.96]	Inferior by ≥20%	0.001
[TKR] Insall-Burstein 2 Cement, posterior-stabilised fixed	985	1.74	0.50	[-0.32 , 1.31]	Non-inferiority not shown	0.115
[TKR] Journey Oxinium Cement, posterior-stabilised fixed	338	3.13	1.89	[0.06 , 3.72]	Non-inferiority not shown	0.021
[TKR] Kinemax Cement, unconstrained fixed	4,241	1.88	0.64	[0.21 , 1.06]	Non-inferiority not shown	0.002
[TKR] LCS Complete Cement, unconstrained mobile	3,604	1.59	0.35	[-0.07 , 0.76]	Non-inferiority not shown	0.051
[TKR] LCS Complete Uncemented, unconstrained mobile	4,705	1.83	0.59	[0.21 , 0.97]	Non-inferiority not shown	0.001
[TKR] LCS Uncemented, unconstrained mobile	516	2.04	0.80	[-0.41 , 2.01]	Non-inferiority not shown	0.097
[TKR] MRK Cement, unconstrained fixed	2,493	1.52	0.27	[-0.18 , 0.72]	Non-inferiority not shown	0.117
[TKR] Maxim Cement, unconstrained fixed	530	1.09	-0.16	[-1.04 , 0.72]	Non-inferiority not shown	0.364
[TKR] NRG Cement, posterior-stabilised fixed	1,747	1.81	0.57	[-0.05 , 1.18]	Non-inferiority not shown	0.035
[TKR] NRG Cement, unconstrained fixed	2,486	1.83	0.59	[0.08 , 1.10]	Non-inferiority not shown	0.012
[TKR] Natural Knee II Cement, unconstrained fixed	997	1.03	-0.21	[-0.84 , 0.41]	Non-inferiority not shown	0.251
[TKR] NexGen Cement, posterior-stabilised mobile	324	3.61	2.36	[0.50 , 4.23]	Inferior by ≥20%	0.007
[TKR] NexGen Cement, posterior-stabilised fixed	16,394	1.95	0.71	[0.46 , 0.95]	Inferior by ≥20%	<0.001
[TKR] NexGen Hybrid, unconstrained fixed	457	0.81	-0.43	[-1.24 , 0.37]	Non-inferiority not shown	0.147
[TKR] NexGen Uncemented, posterior-stabilised fixed	774	1.35	0.10	[-0.64 , 0.85]	Non-inferiority not shown	0.392
[TKR] NexGen Uncemented, unconstrained fixed	1,965	2.47	1.22	[0.55 , 1.90]	Inferior by ≥20%	<0.001
[TKR] Optetrak Cement, posterior-stabilised fixed	614	2.01	0.77	[-0.32 , 1.87]	Non-inferiority not shown	0.084
[TKR] PFC Sigma Bicondylar Knee Cement, posterior-stabilised mobile	2,652	2.16	0.92	[0.38 , 1.46]	Inferior by ≥20%	<0.001
[TKR] PFC Sigma Bicondylar Knee Cement, constrained condylar	251	1.88	0.63	[-0.87 , 2.13]	Non-inferiority not shown	0.205
[TKR] PFC Sigma Bicondylar Knee Cement, posterior-stabilised fixed	22,261	1.65	0.40	[0.18 , 0.62]	Non-inferiority not shown	<0.001
[TKR] PFC Sigma Bicondylar Knee Cement, unconstrained fixed	53,160	1.39	0.15	[-0.03 , 0.33]	Non-inferiority not shown	0.050
[TKR] PFC Sigma Bicondylar Knee Cement, unconstrained mobile	3,150	2.14	0.89	[0.39 , 1.39]	Inferior by ≥20%	<0.001
[TKR] PFC Sigma Bicondylar Knee Hybrid, unconstrained fixed	700	0.95	-0.29	[-1.01 , 0.43]	Non-inferiority not shown	0.213
[TKR] PFC Sigma Bicondylar Knee Uncemented, unconstrained mobile	280	1.61	0.36	[-0.93 , 1.65]	Non-inferiority not shown	0.292
[TKR] PFC Sigma Bicondylar Knee monobloc polyethylene tibia	2,112	1.44	0.19	[-0.26 , 0.64]	Non-inferiority not shown	0.204

For peer review only

[TKR] Profix Cement, unconstrained fixed	392	2.15	0.90	[-0.49 , 2.30]	Non-inferiority not shown	0.102
[TKR] Profix Uncemented, unconstrained fixed	954	1.00	-0.25	[-0.88 , 0.39]	Non-inferiority not shown	0.225
[TKR] Profix monobloc polyethylene tibia	257	0.36	-0.88	[-1.61 , -0.15]	Non-inferior	0.009
[TKR] Rotaglide + Cement, unconstrained mobile	668	3.36	2.12	[0.79 , 3.45]	Inferior by ≥20%	0.001
[TKR] Rotaglide Cement, unconstrained mobile	403	2.75	1.51	[0.02 , 2.99]	Non-inferiority not shown	0.024
[TKR] Scorpio Cement, posterior-stabilised mobile	565	1.69	0.44	[-0.61 , 1.49]	Non-inferiority not shown	0.204
[TKR] Scorpio Cement, posterior-stabilised fixed	2,265	1.97	0.72	[0.14 , 1.30]	Non-inferiority not shown	0.007
[TKR] Scorpio Cement, unconstrained fixed	4,090	2.25	1.00	[0.53 , 1.47]	Inferior by ≥20%	<0.001
[TKR] Scorpio Cement, unconstrained mobile	464	2.66	1.41	[-0.02 , 2.84]	Non-inferiority not shown	0.027
[TKR] Scorpio Hybrid, unconstrained fixed	405	2.32	1.07	[-0.35 , 2.50]	Non-inferiority not shown	0.070
[TKR] Scorpio Uncemented, unconstrained fixed	1,646	1.75	0.51	[-0.13 , 1.15]	Non-inferiority not shown	0.060
[TKR] TC Plus Cement, unconstrained fixed	3,396	2.32	1.07	[0.56 , 1.59]	Inferior by ≥20%	<0.001
[TKR] TC Plus Cement, unconstrained mobile	1,934	1.98	0.74	[0.12 , 1.35]	Non-inferiority not shown	0.009
[TKR] TC Plus Uncemented, unconstrained mobile	550	1.43	0.18	[-0.71 , 1.08]	Non-inferiority not shown	0.345
[TKR] Triathlon Cement, posterior-stabilised fixed	3,282	1.77	0.52	[0.12 , 0.93]	Non-inferiority not shown	0.006
[TKR] Triathlon Cement, unconstrained fixed	12,371	1.42	0.17	[-0.06 , 0.40]	Non-inferiority not shown	0.073
[TKR] Triathlon Uncemented, unconstrained fixed	332	2.49	1.24	[-0.19 , 2.67]	Non-inferiority not shown	0.044
[TKR] Vanguard Cement, posterior-stabilised fixed	1,226	1.75	0.51	[-0.08 , 1.10]	Non-inferiority not shown	0.046
[TKR] Vanguard Cement, unconstrained fixed	8,120	1.51	0.27	[-0.00 , 0.54]	Non-inferiority not shown	0.025
[UNI] AMC/Uniglide Unicondylar, fixed	501	2.65	1.40	[0.07 , 2.74]	Non-inferiority not shown	0.020
[UNI] AMC/Uniglide Unicondylar, mobile	628	7.99	6.75	[4.78 , 8.71]	Inferior by ≥100%	<0.001
[UNI] MG Uni Unicondylar, fixed	1,203	3.48	2.23	[1.21 , 3.26]	Inferior by ≥20%	<0.001
[UNI] Oxford Partial Knee Unicondylar, mobile	19,491	3.98	2.74	[2.45 , 3.03]	Inferior by ≥100%	<0.001
[UNI] Physica ZUK Unicondylar, fixed	2,555	2.33	1.08	[0.57 , 1.60]	Inferior by ≥20%	<0.001
[UNI] Preservation Unicondylar, fixed	600	5.68	4.44	[2.65 , 6.22]	Inferior by ≥100%	<0.001
[UNI] Sigma HP Unicondylar, fixed	2,062	3.12	1.87	[1.24 , 2.51]	Inferior by ≥20%	<0.001
[UNI] Sled Unicondylar, fixed	308	6.69	5.45	[2.85 , 8.04]	Inferior by ≥100%	<0.001

For peer review only

Supplementary table 6: Difference in cumulative percentage revision of knee implants compared to a contemporary benchmark at 7 years post primary in men for knee replacements with ≥250 procedures remaining at risk

Knee brand, bearing and constraint	Number at risk	Cumulative failure (%)	Difference in failure (%)	95% CI	Equivalence status	p-value
[TKR] PFC Sigma Bicondylar Knee Cement, unconstrained fixed	22,730	2.16	[REFERENCE]			
[TKR] AGC Cement, unconstrained fixed	11,118	2.94	0.78	[0.51 , 1.04]	Inferior by ≥20%	<0.001
[TKR] AGC Hybrid, unconstrained fixed	250	3.18	1.02	[-0.70 , 2.73]	Non-inferiority not shown	0.123
[TKR] AGC Uncemented, unconstrained fixed	324	5.56	3.40	[1.48 , 5.33]	Inferior by ≥20%	<0.001
[TKR] Advance MP Cement, unconstrained fixed	1,052	3.91	1.75	[0.96 , 2.53]	Inferior by ≥20%	<0.001
[TKR] Columbus Cement, unconstrained fixed	625	2.70	0.53	[-0.15 , 1.22]	Non-inferiority not shown	0.064
[TKR] E-Motion Bicondylar Knee Uncemented, unconstrained mobile	352	3.20	1.04	[-0.34 , 2.43]	Non-inferiority not shown	0.070
[TKR] FS Cement, unconstrained fixed	276	2.10	-0.06	[-1.61 , 1.48]	Non-inferiority not shown	0.468
[TKR] Genesis 2 Cement, posterior-stabilised fixed	848	3.70	1.54	[0.80 , 2.28]	Inferior by ≥20%	<0.001
[TKR] Genesis 2 Cement, unconstrained fixed	3,359	2.69	0.53	[0.18 , 0.88]	Non-inferiority not shown	0.002
[TKR] Genesis 2 Oxinium Cement, unconstrained fixed	758	3.98	1.82	[0.83 , 2.81]	Inferior by ≥20%	<0.001
[TKR] Insall-Burstein 2 Cement, posterior-stabilised fixed	797	3.57	1.40	[0.23 , 2.57]	Non-inferiority not shown	0.009
[TKR] Kinemax Cement, unconstrained fixed	3,602	3.87	1.71	[1.12 , 2.30]	Inferior by ≥20%	<0.001
[TKR] LCS Complete Cement, unconstrained mobile	1,775	3.47	1.31	[0.68 , 1.94]	Inferior by ≥20%	<0.001
[TKR] LCS Complete Uncemented, unconstrained mobile	2,105	2.92	0.76	[0.25 , 1.27]	Non-inferiority not shown	0.002
[TKR] LCS Uncemented, unconstrained mobile	457	2.82	0.66	[-0.75 , 2.07]	Non-inferiority not shown	0.181
[TKR] MRK Cement, unconstrained fixed	1,056	2.97	0.80	[0.08 , 1.53]	Non-inferiority not shown	0.015
[TKR] Maxim Cement, unconstrained fixed	380	2.84	0.68	[-0.81 , 2.16]	Non-inferiority not shown	0.186
[TKR] NRG Cement, posterior-stabilised fixed	580	3.14	0.98	[0.13 , 1.83]	Non-inferiority not shown	0.012
[TKR] NRG Cement, unconstrained fixed	908	3.76	1.60	[0.77 , 2.43]	Inferior by ≥20%	<0.001
[TKR] Natural Knee II Cement, unconstrained fixed	559	3.46	1.30	[0.05 , 2.55]	Non-inferiority not shown	0.021
[TKR] NexGen Cement, posterior-stabilised fixed	7,174	3.81	1.65	[1.32 , 1.99]	Inferior by ≥20%	<0.001
[TKR] NexGen Cement, unconstrained fixed	4,862	2.32	0.16	[-0.14 , 0.45]	Non-inferiority not shown	0.151
[TKR] NexGen Uncemented, posterior-stabilised fixed	419	2.04	-0.12	[-1.13 , 0.89]	Non-inferiority not shown	0.410
[TKR] NexGen Uncemented, unconstrained fixed	1,270	3.52	1.35	[0.53 , 2.17]	Inferior by ≥20%	0.001
[TKR] Optetrak Cement, posterior-stabilised fixed	335	4.79	2.63	[0.87 , 4.38]	Inferior by ≥20%	0.002
[TKR] PFC Sigma Bicondylar Knee Cement, posterior-stabilised mobile	1,226	3.23	1.07	[0.37 , 1.77]	Non-inferiority not shown	0.001
[TKR] PFC Sigma Bicondylar Knee Cement, posterior-stabilised fixed	9,690	2.53	0.37	[0.12 , 0.61]	Non-inferiority not shown	0.002
[TKR] PFC Sigma Bicondylar Knee Cement, unconstrained mobile	1,807	3.24	1.08	[0.44 , 1.71]	Inferior by ≥20%	<0.001
[TKR] PFC Sigma Bicondylar Knee Hybrid, unconstrained fixed	527	1.86	-0.31	[-1.31 , 0.70]	Non-inferiority not shown	0.277
[TKR] PFC Sigma Bicondylar Knee monobloc polyethylene tibia	287	2.16	0.00	[-0.75 , 0.75]	Non-inferiority not shown	0.498
[TKR] Profix Cement, unconstrained fixed	306	5.09	2.93	[0.74 , 5.11]	Inferior by ≥20%	0.004
[TKR] Profix Uncemented, unconstrained fixed	676	1.73	-0.44	[-1.29 , 0.42]	Non-inferior	0.158
[TKR] Rotaglide + Cement, unconstrained mobile	509	5.27	3.10	[1.42 , 4.79]	Inferior by ≥20%	<0.001
[TKR] Scorpio Cement, posterior-stabilised mobile	437	2.60	0.44	[-0.87 , 1.74]	Non-inferiority not shown	0.256
[TKR] Scorpio Cement, posterior-stabilised fixed	1,835	3.88	1.72	[0.91 , 2.53]	Inferior by ≥20%	<0.001
[TKR] Scorpio Cement, unconstrained fixed	2,978	3.76	1.60	[1.00 , 2.20]	Inferior by ≥20%	<0.001
[TKR] Scorpio Cement, unconstrained mobile	392	4.62	2.46	[0.57 , 4.35]	Inferior by ≥20%	0.005
[TKR] Scorpio Hybrid, unconstrained fixed	326	4.13	1.97	[0.04 , 3.91]	Non-inferiority not shown	0.023
[TKR] Scorpio Uncemented, unconstrained fixed	993	3.15	0.99	[0.11 , 1.87]	Non-inferiority not shown	0.014

For peer review only

[TKR] TC Plus Cement, unconstrained fixed	2,380	3.40	1.24	[0.62 , 1.86]	Inferior by ≥20%	<0.001
[TKR] TC Plus Cement, unconstrained mobile	1,419	3.20	1.04	[0.25 , 1.82]	Non-inferiority not shown	0.005
[TKR] TC Plus Uncemented, unconstrained mobile	281	2.32	0.16	[-1.10 , 1.42]	Non-inferiority not shown	0.400
[TKR] Triathlon Cement, posterior-stabilised fixed	614	2.75	0.59	[0.03 , 1.15]	Non-inferiority not shown	0.019
[TKR] Triathlon Cement, unconstrained fixed	1,973	2.52	0.36	[-0.00 , 0.72]	Non-inferiority not shown	0.026
[TKR] Vanguard Cement, unconstrained fixed	1,017	2.24	0.08	[-0.27 , 0.42]	Non-inferior	0.336
[UNI] AMC/Uniglide Unicondylar, mobile	350	10.65	8.49	[6.16 , 10.82]	Inferior by ≥100%	<0.001
[UNI] MG Uni Unicondylar, fixed	990	6.59	4.43	[3.04 , 5.83]	Inferior by ≥100%	<0.001
[UNI] Oxford Partial Knee Unicondylar, mobile	9,524	7.45	5.29	[4.89 , 5.69]	Inferior by ≥100%	<0.001
[UNI] Physica ZUK Unicondylar, fixed	594	4.64	2.48	[1.57 , 3.40]	Inferior by ≥20%	<0.001
[UNI] Preservation Unicondylar, fixed	505	11.21	9.05	[6.58 , 11.51]	Inferior by ≥100%	<0.001

For peer review only

Supplementary table 7: Difference in cumulative percentage revision of knee implants compared to a contemporary benchmark at 10 years post primary in men for knee replacements with ≥250 procedures remaining at risk

Knee brand, bearing and constraint	Number at risk	Cumulative failure (%)	Difference in failure (%)	95% CI	Equivalence status	p-value
[TKR] PFC Sigma Bicondylar Knee Cement, unconstrained fixed	7,760	2.55	[REFERENCE]			
[TKR] AGC Cement, unconstrained fixed	3,646	4.12	1.57	[1.18 , 1.95]	Inferior by ≥20%	<0.001
[TKR] Advance MP Cement, unconstrained fixed	279	4.50	1.94	[0.85 , 3.03]	Inferior by ≥20%	<0.001
[TKR] Genesis 2 Cement, unconstrained fixed	807	3.26	0.70	[0.21 , 1.19]	Non-inferiority not shown	0.003
[TKR] Insall-Burstein 2 Cement, posterior-stabilised fixed	457	5.91	3.35	[1.73 , 4.98]	Inferior by ≥20%	<0.001
[TKR] Kinemax Cement, unconstrained fixed	2,113	5.00	2.45	[1.75 , 3.15]	Inferior by ≥20%	<0.001
[TKR] LCS Complete Cement, unconstrained mobile	303	4.25	1.70	[0.91 , 2.49]	Inferior by ≥20%	<0.001
[TKR] LCS Complete Uncemented, unconstrained mobile	602	3.33	0.78	[0.18 , 1.38]	Non-inferiority not shown	0.006
[TKR] LCS Uncemented, unconstrained mobile	406	3.04	0.49	[-0.99 , 1.97]	Non-inferiority not shown	0.258
[TKR] NexGen Cement, posterior-stabilised fixed	2,308	5.14	2.58	[2.10 , 3.07]	Inferior by ≥20%	<0.001
[TKR] NexGen Cement, unconstrained fixed	1,167	2.71	0.16	[-0.24 , 0.55]	Non-inferiority not shown	0.220
[TKR] NexGen Uncemented, unconstrained fixed	348	3.61	1.05	[0.21 , 1.90]	Non-inferiority not shown	0.007
[TKR] PFC Sigma Bicondylar Knee Cement, posterior-stabilised mobile	340	4.31	1.75	[0.71 , 2.80]	Inferior by ≥20%	0.001
[TKR] PFC Sigma Bicondylar Knee Cement, posterior-stabilised fixed	3,119	3.16	0.61	[0.28 , 0.94]	Non-inferiority not shown	<0.001
[TKR] PFC Sigma Bicondylar Knee Cement, unconstrained mobile	419	3.97	1.41	[0.55 , 2.27]	Inferior by ≥20%	0.001
[TKR] PFC Sigma Bicondylar Knee Hybrid, unconstrained fixed	276	2.14	-0.41	[-1.57 , 0.74]	Non-inferiority not shown	0.241
[TKR] Profix Uncemented, unconstrained fixed	278	2.35	-0.20	[-1.32 , 0.91]	Non-inferiority not shown	0.361
[TKR] Rotaglide + Cement, unconstrained mobile	289	6.95	4.40	[2.31 , 6.48]	Inferior by ≥20%	<0.001
[TKR] Scorpio Cement, posterior-stabilised fixed	794	4.65	2.10	[1.16 , 3.03]	Inferior by ≥20%	<0.001
[TKR] Scorpio Cement, unconstrained fixed	1,325	4.43	1.87	[1.19 , 2.56]	Inferior by ≥20%	<0.001
[TKR] TC Plus Cement, unconstrained fixed	565	4.15	1.60	[0.79 , 2.41]	Inferior by ≥20%	<0.001
[TKR] TC Plus Cement, unconstrained mobile	668	4.06	1.50	[0.55 , 2.45]	Inferior by ≥20%	0.001
[UNI] MG Uni Unicondylar, fixed	445	8.58	6.02	[4.34 , 7.70]	Inferior by ≥100%	<0.001
[UNI] Oxford Partial Knee Unicondylar, mobile	3,124	10.63	8.08	[7.48 , 8.67]	Inferior by ≥100%	<0.001

For peer review only

Supplementary table 8: Difference in cumulative percentage revision of knee implants compared to a contemporary benchmark at 3 years post primary in women for knee replacements with ≥250 procedures remaining at risk

Knee brand, bearing and constraint	Number at risk	Cumulative failure (%)	Difference in failure (%)	95% CI	Equivalence status	p-value
[TKR] MRK Cement, unconstrained fixed	3,684	0.97	[REFERENCE]			
[TKR] AGC Cement, posterior-stabilised fixed	292	2.31	1.33	[-0.38 , 3.05]	Non-inferiority not shown	0.063
[TKR] AGC Cement, unconstrained fixed	29,830	1.30	0.33	[0.02 , 0.63]	Non-inferiority not shown	0.017
[TKR] AGC Hybrid, unconstrained fixed	462	2.46	1.49	[0.09 , 2.90]	Non-inferiority not shown	0.019
[TKR] AGC Uncemented, unconstrained fixed	536	4.24	3.27	[1.59 , 4.95]	Inferior by ≥100%	<0.001
[TKR] Advance MP Cement, unconstrained fixed	3,068	1.74	0.77	[0.26 , 1.27]	Inferior by ≥20%	0.002
[TKR] Advance MP Stature Cement, unconstrained fixed	693	1.84	0.87	[-0.04 , 1.78]	Non-inferiority not shown	0.031
[TKR] Advance PS Cement, posterior-stabilised fixed	449	2.27	1.29	[-0.01 , 2.60]	Non-inferiority not shown	0.026
[TKR] Alpina Hybrid, unconstrained fixed	278	1.74	0.77	[-0.77 , 2.31]	Non-inferiority not shown	0.164
[TKR] Columbus Cement, unconstrained fixed	2,921	1.78	0.81	[0.32 , 1.30]	Inferior by ≥20%	0.001
[TKR] E-Motion Bicondylar Knee Cement, unconstrained mobile	451	2.94	1.96	[0.55 , 3.38]	Inferior by ≥20%	0.003
[TKR] E-Motion Bicondylar Knee Uncemented, unconstrained mobile	692	2.42	1.45	[0.36 , 2.53]	Inferior by ≥20%	0.004
[TKR] FS Cement, unconstrained fixed	417	1.62	0.65	[-0.58 , 1.87]	Non-inferiority not shown	0.150
[TKR] Genesis 2 Cement, posterior-stabilised fixed	4,795	1.53	0.56	[0.16 , 0.96]	Non-inferiority not shown	0.003
[TKR] Genesis 2 Cement, unconstrained fixed	14,679	1.12	0.15	[-0.16 , 0.46]	Non-inferiority not shown	0.174
[TKR] Genesis 2 Cement, unconstrained mobile	526	1.29	0.32	[-0.67 , 1.31]	Non-inferiority not shown	0.263
[TKR] Genesis 2 Oxinium Cement, posterior-stabilised fixed	981	2.41	1.44	[0.55 , 2.32]	Inferior by ≥20%	0.001
[TKR] Genesis 2 Oxinium Cement, unconstrained fixed	2,060	1.55	0.57	[0.01 , 1.14]	Non-inferiority not shown	0.023
[TKR] Insall-Burstein 2 Cement, posterior-stabilised fixed	1,242	1.25	0.28	[-0.39 , 0.95]	Non-inferiority not shown	0.205
[TKR] Journey Oxinium Cement, posterior-stabilised fixed	407	5.77	4.80	[2.59 , 7.01]	Inferior by ≥100%	<0.001
[TKR] Kinemax Cement, unconstrained fixed	5,695	1.71	0.73	[0.30 , 1.16]	Inferior by ≥20%	<0.001
[TKR] LCS Cement, unconstrained mobile	363	1.58	0.60	[-0.68 , 1.89]	Non-inferiority not shown	0.178
[TKR] LCS Complete Cement, unconstrained mobile	4,847	1.65	0.68	[0.24 , 1.11]	Inferior by ≥20%	0.001
[TKR] LCS Complete Uncemented, unconstrained mobile	5,591	1.68	0.71	[0.29 , 1.13]	Inferior by ≥20%	<0.001
[TKR] LCS Uncemented, unconstrained mobile	722	1.74	0.77	[-0.21 , 1.75]	Non-inferiority not shown	0.062
[TKR] Maxim Cement, posterior-stabilised fixed	340	1.69	0.72	[-0.65 , 2.09]	Non-inferiority not shown	0.153
[TKR] Maxim Cement, unconstrained fixed	715	1.76	0.79	[-0.20 , 1.78]	Non-inferiority not shown	0.059
[TKR] NRG Cement, posterior-stabilised fixed	2,394	1.63	0.66	[0.10 , 1.22]	Non-inferiority not shown	0.011
[TKR] NRG Cement, unconstrained fixed	3,377	1.22	0.24	[-0.20 , 0.68]	Non-inferiority not shown	0.140
[TKR] Natural Knee II Cement, unconstrained fixed	1,485	1.60	0.63	[-0.05 , 1.31]	Non-inferiority not shown	0.035
[TKR] NexGen Cement, posterior-stabilised mobile	490	2.31	1.33	[0.10 , 2.57]	Non-inferiority not shown	0.017
[TKR] NexGen Cement, posterior-stabilised fixed	24,309	1.38	0.40	[0.10 , 0.71]	Non-inferiority not shown	0.005
[TKR] NexGen Cement, unconstrained fixed	20,162	0.99	0.02	[-0.28 , 0.32]	Non-inferiority not shown	0.452
[TKR] NexGen Hybrid, unconstrained fixed	507	1.30	0.33	[-0.67 , 1.33]	Non-inferiority not shown	0.259
[TKR] NexGen Uncemented, posterior-stabilised fixed	650	2.15	1.18	[0.12 , 2.23]	Non-inferiority not shown	0.014
[TKR] NexGen Uncemented, unconstrained fixed	1,577	2.66	1.69	[0.87 , 2.51]	Inferior by ≥20%	<0.001
[TKR] NexGen monobloc polyethylene tibia	314	0.46	-0.51	[-1.22 , 0.20]	Non-inferiority not shown	0.080
[TKR] Optetrak Cement, posterior-stabilised fixed	882	3.11	2.13	[0.99 , 3.28]	Inferior by ≥100%	<0.001
[TKR] PFC Sigma Bicondylar Knee Cement, posterior-stabilised mobile	3,081	2.08	1.10	[0.56 , 1.65]	Inferior by ≥20%	<0.001
[TKR] PFC Sigma Bicondylar Knee Cement, constrained condylar	310	1.62	0.64	[-0.58 , 1.87]	Non-inferiority not shown	0.152

For peer review only - http://bmjopen.bmj.com/site/about/guidelines.xhtml

[TKR] PFC Sigma Bicondylar Knee Cement, posterior-stabilised fixed	32,691	1.42	0.45	[0.15 , 0.75]	Non-inferiority not shown	0.002
[TKR] PFC Sigma Bicondylar Knee Cement, unconstrained fixed	72,974	1.13	0.16	[-0.12 , 0.45]	Non-inferiority not shown	0.134
[TKR] PFC Sigma Bicondylar Knee Cement, unconstrained mobile	3,424	1.75	0.78	[0.28 , 1.28]	Inferior by ≥20%	0.001
[TKR] PFC Sigma Bicondylar Knee Hybrid, unconstrained fixed	833	1.38	0.40	[-0.42 , 1.23]	Non-inferiority not shown	0.167
[TKR] PFC Sigma Bicondylar Knee Uncemented, unconstrained mobile	324	1.28	0.31	[-0.85 , 1.47]	Non-inferiority not shown	0.299
[TKR] PFC Sigma Bicondylar Knee monobloc polyethylene tibia	3,257	1.22	0.24	[-0.17 , 0.66]	Non-inferiority not shown	0.126
[TKR] Profix Cement, unconstrained fixed	492	1.96	0.99	[-0.25 , 2.22]	Non-inferiority not shown	0.059
[TKR] Profix Oxinium monobloc polyethylene tibia	327	2.68	1.71	[-0.04 , 3.45]	Non-inferiority not shown	0.028
[TKR] Profix Uncemented, unconstrained fixed	1,184	1.46	0.49	[-0.24 , 1.22]	Non-inferiority not shown	0.093
[TKR] Profix monobloc polyethylene tibia	395	0.96	-0.01	[-0.99 , 0.97]	Non-inferiority not shown	0.493
[TKR] Rotaglide + Cement, unconstrained mobile	909	2.42	1.45	[0.43 , 2.46]	Inferior by ≥20%	0.003
[TKR] Rotaglide Cement, unconstrained mobile	649	1.62	0.64	[-0.31 , 1.60]	Non-inferiority not shown	0.092
[TKR] Scorpio Cement, posterior-stabilised mobile	725	1.33	0.36	[-0.51 , 1.23]	Non-inferiority not shown	0.208
[TKR] Scorpio Cement, posterior-stabilised fixed	3,409	1.33	0.36	[-0.11 , 0.82]	Non-inferiority not shown	0.068
[TKR] Scorpio Cement, unconstrained fixed	5,993	1.58	0.60	[0.19 , 1.02]	Non-inferiority not shown	0.002
[TKR] Scorpio Cement, unconstrained mobile	631	2.45	1.48	[0.26 , 2.69]	Inferior by ≥20%	0.009
[TKR] Scorpio Hybrid, unconstrained fixed	601	1.44	0.46	[-0.51 , 1.43]	Non-inferiority not shown	0.176
[TKR] Scorpio Uncemented, unconstrained fixed	1,885	1.83	0.86	[0.20 , 1.51]	Inferior by ≥20%	0.005
[TKR] TC Plus Cement, unconstrained fixed	4,106	1.53	0.55	[0.09 , 1.01]	Non-inferiority not shown	0.009
[TKR] TC Plus Cement, unconstrained mobile	2,452	1.02	0.04	[-0.43 , 0.51]	Non-inferiority not shown	0.428
[TKR] TC Plus Uncemented, unconstrained mobile	890	1.56	0.59	[-0.22 , 1.40]	Non-inferiority not shown	0.077
[TKR] Triathlon Cement, posterior-stabilised fixed	4,914	1.70	0.72	[0.31 , 1.14]	Inferior by ≥20%	<0.001
[TKR] Triathlon Cement, unconstrained fixed	16,831	1.53	0.56	[0.24 , 0.87]	Inferior by ≥20%	<0.001
[TKR] Triathlon Uncemented, unconstrained fixed	411	2.97	1.99	[0.51 , 3.47]	Inferior by ≥20%	0.004
[TKR] Vanguard Cement, constrained condylar	260	0.39	-0.59	[-1.11 , -0.06]	Non-inferior	0.014
[TKR] Vanguard Cement, posterior-stabilised fixed	1,764	2.01	1.04	[0.45 , 1.63]	Inferior by ≥20%	<0.001
[TKR] Vanguard Cement, unconstrained fixed	11,272	1.27	0.30	[-0.03 , 0.62]	Non-inferiority not shown	0.037
[UNI] AMC/Uniglide Unicondylar, fixed	571	3.27	2.30	[0.89 , 3.70]	Inferior by ≥20%	0.001
[UNI] AMC/Uniglide Unicondylar, mobile	568	9.00	8.03	[5.82 , 10.24]	Inferior by ≥100%	<0.001
[UNI] MG Uni Unicondylar, fixed	1,004	4.54	3.57	[2.28 , 4.85]	Inferior by ≥100%	<0.001
[UNI] Oxford Partial Knee Unicondylar, mobile	17,891	4.18	3.20	[2.82 , 3.59]	Inferior by ≥100%	<0.001
[UNI] Physica ZUK Unicondylar, fixed	2,153	2.59	1.61	[0.98 , 2.25]	Inferior by ≥100%	<0.001
[UNI] Preservation Unicondylar, fixed	506	8.29	7.32	[5.01 , 9.63]	Inferior by ≥100%	<0.001
[UNI] Sigma HP Unicondylar, fixed	1,499	4.15	3.17	[2.30 , 4.05]	Inferior by ≥100%	<0.001
[UNI] Sled Unicondylar, fixed	375	5.85	4.87	[2.63 , 7.12]	Inferior by ≥100%	<0.001

For peer review only

Supplementary table 9: Difference in cumulative percentage revision of knee implants compared to a contemporary benchmark at 7 years post primary in women for knee replacements with ≥250 procedures remaining at risk

Knee brand, bearing and constraint	Number at risk	Cumulative failure (%)	Difference in failure (%)	95% CI	Equivalence status	p-value
[TKR] Genesis 2 Cement, unconstrained fixed	4,697	1.77	[REFERENCE]			
[TKR] AGC Cement, unconstrained fixed	16,170	2.29	0.52	[0.23 , 0.80]	Non-inferiority not shown	<0.001
[TKR] AGC Hybrid, unconstrained fixed	292	3.33	1.56	[-0.06 , 3.18]	Non-inferiority not shown	0.030
[TKR] AGC Uncemented, unconstrained fixed	387	6.37	4.59	[2.54 , 6.65]	Inferior by ≥100%	<0.001
[TKR] Advance MP Cement, unconstrained fixed	1,537	3.17	1.40	[0.72 , 2.08]	Inferior by ≥20%	<0.001
[TKR] Columbus Cement, unconstrained fixed	858	2.87	1.10	[0.42 , 1.77]	Inferior by ≥20%	0.001
[TKR] E-Motion Bicondylar Knee Uncemented, unconstrained mobile	381	4.50	2.73	[1.11 , 4.35]	Inferior by ≥20%	<0.001
[TKR] FS Cement, unconstrained fixed	368	3.10	1.32	[-0.35 , 3.00]	Non-inferiority not shown	0.061
[TKR] Genesis 2 Cement, posterior-stabilised fixed	1,428	2.50	0.73	[0.22 , 1.24]	Non-inferiority not shown	0.003
[TKR] Genesis 2 Cement, unconstrained mobile	328	1.97	0.20	[-1.04 , 1.44]	Non-inferiority not shown	0.376
[TKR] Genesis 2 Oxinium Cement, posterior-stabilised fixed	324	5.72	3.94	[2.18 , 5.70]	Inferior by ≥100%	<0.001
[TKR] Genesis 2 Oxinium Cement, unconstrained fixed	906	3.15	1.38	[0.57 , 2.19]	Inferior by ≥20%	<0.001
[TKR] Insall-Burstein 2 Cement, posterior-stabilised fixed	1,086	3.31	1.54	[0.51 , 2.56]	Inferior by ≥20%	0.002
[TKR] Kinemax Cement, unconstrained fixed	4,958	3.28	1.51	[0.99 , 2.02]	Inferior by ≥20%	<0.001
[TKR] LCS Cement, unconstrained mobile	312	2.47	0.70	[-0.92 , 2.31]	Non-inferiority not shown	0.199
[TKR] LCS Complete Cement, unconstrained mobile	2,442	3.42	1.64	[1.06 , 2.22]	Inferior by ≥20%	<0.001
[TKR] LCS Complete Uncemented, unconstrained mobile	2,619	2.93	1.15	[0.65 , 1.65]	Inferior by ≥20%	<0.001
[TKR] LCS Uncemented, unconstrained mobile	636	2.16	0.39	[-0.68 , 1.46]	Non-inferiority not shown	0.238
[TKR] MRK Cement, unconstrained fixed	1,720	1.83	0.05	[-0.45 , 0.56]	Non-inferiority not shown	0.416
[TKR] Maxim Cement, unconstrained fixed	502	3.22	1.44	[0.09 , 2.80]	Non-inferiority not shown	0.018
[TKR] NRG Cement, posterior-stabilised fixed	859	2.66	0.89	[0.17 , 1.60]	Non-inferiority not shown	0.008
[TKR] NRG Cement, unconstrained fixed	1,301	2.15	0.38	[-0.18 , 0.94]	Non-inferiority not shown	0.092
[TKR] Natural Knee II Cement, unconstrained fixed	879	3.04	1.26	[0.32 , 2.21]	Non-inferiority not shown	0.005
[TKR] NexGen Cement, posterior-stabilised mobile	337	5.09	3.32	[1.36 , 5.28]	Inferior by ≥20%	<0.001
[TKR] NexGen Cement, posterior-stabilised fixed	10,683	2.97	1.20	[0.88 , 1.52]	Inferior by ≥20%	<0.001
[TKR] NexGen Cement, unconstrained fixed	7,469	1.98	0.21	[-0.10 , 0.51]	Non-inferiority not shown	0.095
[TKR] NexGen Hybrid, unconstrained fixed	333	2.18	0.40	[-0.89 , 1.70]	Non-inferiority not shown	0.271
[TKR] NexGen Uncemented, posterior-stabilised fixed	366	3.11	1.34	[0.00 , 2.67]	Non-inferiority not shown	0.025
[TKR] NexGen Uncemented, unconstrained fixed	1,261	3.67	1.90	[0.96 , 2.84]	Inferior by ≥20%	<0.001
[TKR] Optetrak Cement, posterior-stabilised fixed	474	5.87	4.10	[2.52 , 5.67]	Inferior by ≥100%	<0.001
[TKR] PFC Sigma Bicondylar Knee Cement, posterior-stabilised mobile	1,460	3.46	1.69	[0.99 , 2.39]	Inferior by ≥20%	<0.001
[TKR] PFC Sigma Bicondylar Knee Cement, posterior-stabilised fixed	14,968	2.39	0.61	[0.33 , 0.90]	Non-inferiority not shown	<0.001
[TKR] PFC Sigma Bicondylar Knee Cement, unconstrained fixed	32,901	1.89	0.11	[-0.13 , 0.36]	Non-inferiority not shown	0.183
[TKR] PFC Sigma Bicondylar Knee Cement, unconstrained mobile	2,012	3.07	1.30	[0.68 , 1.92]	Inferior by ≥20%	<0.001
[TKR] PFC Sigma Bicondylar Knee Hybrid, unconstrained fixed	648	1.88	0.10	[-0.84 , 1.04]	Non-inferiority not shown	0.414
[TKR] PFC Sigma Bicondylar Knee monobloc polyethylene tibia	523	1.78	0.00	[-0.56 , 0.57]	Non-inferiority not shown	0.496
[TKR] Profix Cement, unconstrained fixed	388	3.48	1.71	[0.06 , 3.36]	Non-inferiority not shown	0.021
[TKR] Profix Oxinium monobloc polyethylene tibia	276	3.90	2.13	[0.04 , 4.22]	Non-inferiority not shown	0.023
[TKR] Profix Uncemented, unconstrained fixed	827	1.81	0.04	[-0.75 , 0.82]	Non-inferiority not shown	0.461
[TKR] Profix monobloc polyethylene tibia	323	1.47	-0.30	[-1.49 , 0.89]	Non-inferiority not shown	0.309

For peer review only

For peer review only - http://bmjopen.bmj.com/site/about/guidelines.xhtml

[TKR] Rotaglide + Cement, unconstrained mobile	715	3.45	1.68	[0.48 , 2.88]	Inferior by ≥20%	0.003
[TKR] Rotaglide Cement, unconstrained mobile	318	3.32	1.55	[0.10 , 3.00]	Non-inferiority not shown	0.018
[TKR] Scorpio Cement, posterior-stabilised mobile	577	2.50	0.72	[-0.44 , 1.89]	Non-inferiority not shown	0.112
[TKR] Scorpio Cement, posterior-stabilised fixed	2,841	2.53	0.76	[0.18 , 1.33]	Non-inferiority not shown	0.005
[TKR] Scorpio Cement, unconstrained fixed	4,356	2.72	0.95	[0.47 , 1.42]	Inferior by ≥20%	<0.001
[TKR] Scorpio Cement, unconstrained mobile	559	4.37	2.60	[1.00 , 4.20]	Inferior by ≥20%	0.001
[TKR] Scorpio Hybrid, unconstrained fixed	499	2.69	0.92	[-0.41 , 2.24]	Non-inferiority not shown	0.087
[TKR] Scorpio Uncemented, unconstrained fixed	1,219	3.07	1.29	[0.47 , 2.11]	Inferior by ≥20%	0.001
[TKR] TC Plus Cement, unconstrained fixed	2,962	2.62	0.85	[0.31 , 1.38]	Non-inferiority not shown	0.001
[TKR] TC Plus Cement, unconstrained mobile	1,850	1.85	0.08	[-0.50 , 0.66]	Non-inferiority not shown	0.394
[TKR] TC Plus Uncemented, unconstrained mobile	502	2.56	0.79	[-0.26 , 1.84]	Non-inferiority not shown	0.070
[TKR] Triathlon Cement, posterior-stabilised fixed	966	2.94	1.17	[0.60 , 1.74]	Inferior by ≥20%	<0.001
[TKR] Triathlon Cement, unconstrained fixed	2,850	2.49	0.72	[0.37 , 1.06]	Inferior by ≥20%	<0.001
[TKR] Vanguard Cement, unconstrained fixed	1,475	2.44	0.67	[0.25 , 1.10]	Non-inferiority not shown	0.001
[UNI] AMC/Uniglide Unicondylar, mobile	338	14.31	12.54	[9.70 , 15.38]	Inferior by ≥100%	<0.001
[UNI] MG Uni Unicondylar, fixed	840	8.68	6.91	[5.18 , 8.64]	Inferior by ≥100%	<0.001
[UNI] Oxford Partial Knee Unicondylar, mobile	9,099	8.78	7.00	[6.52 , 7.49]	Inferior by ≥100%	<0.001
[UNI] Physica ZUK Unicondylar, fixed	497	6.06	4.29	[3.03 , 5.55]	Inferior by ≥100%	<0.001
[UNI] Preservation Unicondylar, fixed	437	14.95	13.18	[10.18 , 16.18]	Inferior by ≥100%	<0.001
[UNI] Sled Unicondylar, fixed	266	10.30	8.53	[5.51 , 11.55]	Inferior by ≥100%	<0.001

For peer review only

Supplementary table 10: Difference in cumulative percentage revision of knee implants compared to a contemporary benchmark at 10 years post primary in women for knee replacements with ≥250 procedures remaining at risk

Knee brand, bearing and constraint	Number at risk	Cumulative failure (%)	Difference in failure (%)	95% CI	Equivalence status	p-value
[TKR] Genesis 2 Cement, unconstrained fixed	1,175	2.13	[REFERENCE]			
[TKR] AGC Cement, unconstrained fixed	5,614	2.97	0.84	[0.45 , 1.23]	Inferior by ≥20%	<0.001
[TKR] Advance MP Cement, unconstrained fixed	443	4.02	1.90	[0.93 , 2.86]	Inferior by ≥20%	<0.001
[TKR] Genesis 2 Oxinium Cement, unconstrained fixed	259	4.33	2.20	[0.99 , 3.41]	Inferior by ≥20%	<0.001
[TKR] Insall-Burstein 2 Cement, posterior-stabilised fixed	670	4.25	2.13	[0.92 , 3.33]	Inferior by ≥20%	<0.001
[TKR] Kinemax Cement, unconstrained fixed	2,936	4.47	2.35	[1.70 , 2.99]	Inferior by ≥20%	<0.001
[TKR] LCS Cement, unconstrained mobile	269	3.15	1.02	[-0.85 , 2.89]	Non-inferiority not shown	0.142
[TKR] LCS Complete Cement, unconstrained mobile	479	4.29	2.16	[1.34 , 2.98]	Inferior by ≥20%	<0.001
[TKR] LCS Complete Uncemented, unconstrained mobile	738	3.30	1.17	[0.57 , 1.77]	Inferior by ≥20%	<0.001
[TKR] LCS Uncemented, unconstrained mobile	565	2.16	0.04	[-1.06 , 1.13]	Non-inferiority not shown	0.473
[TKR] MRK Cement, unconstrained fixed	341	2.19	0.07	[-0.67 , 0.80]	Non-inferiority not shown	0.431
[TKR] Natural Knee II Cement, unconstrained fixed	362	3.65	1.53	[0.38 , 2.67]	Non-inferiority not shown	0.004
[TKR] NexGen Cement, posterior-stabilised fixed	3,498	3.98	1.85	[1.40 , 2.30]	Inferior by ≥20%	<0.001
[TKR] NexGen Cement, unconstrained fixed	1,721	2.45	0.33	[-0.11 , 0.76]	Non-inferiority not shown	0.069
[TKR] NexGen Uncemented, unconstrained fixed	450	4.30	2.17	[1.08 , 3.26]	Inferior by ≥20%	<0.001
[TKR] PFC Sigma Bicondylar Knee Cement, posterior-stabilised mobile	444	3.87	1.75	[0.88 , 2.61]	Inferior by ≥20%	<0.001
[TKR] PFC Sigma Bicondylar Knee Cement, posterior-stabilised fixed	4,825	2.90	0.78	[0.40 , 1.16]	Non-inferiority not shown	<0.001
[TKR] PFC Sigma Bicondylar Knee Cement, unconstrained fixed	11,524	2.24	0.11	[-0.22 , 0.44]	Non-inferiority not shown	0.258
[TKR] PFC Sigma Bicondylar Knee Cement, unconstrained mobile	467	3.82	1.69	[0.87 , 2.51]	Inferior by ≥20%	<0.001
[TKR] PFC Sigma Bicondylar Knee Hybrid, unconstrained fixed	363	2.06	-0.07	[-1.09 , 0.96]	Non-inferiority not shown	0.450
[TKR] Profix Uncemented, unconstrained fixed	287	2.10	-0.03	[-0.93 , 0.88]	Non-inferiority not shown	0.477
[TKR] Rotaglide + Cement, unconstrained mobile	407	4.62	2.49	[1.01 , 3.97]	Inferior by ≥20%	<0.001
[TKR] Scorpio Cement, posterior-stabilised mobile	274	3.07	0.94	[-0.40 , 2.28]	Non-inferiority not shown	0.084
[TKR] Scorpio Cement, posterior-stabilised fixed	1,201	3.32	1.19	[0.47 , 1.91]	Inferior by ≥20%	0.001
[TKR] Scorpio Cement, unconstrained fixed	1,974	3.41	1.29	[0.70 , 1.87]	Inferior by ≥20%	<0.001
[TKR] Scorpio Uncemented, unconstrained fixed	283	4.01	1.89	[0.69 , 3.08]	Inferior by ≥20%	0.001
[TKR] TC Plus Cement, unconstrained fixed	730	3.03	0.90	[0.23 , 1.57]	Non-inferiority not shown	0.004
[TKR] TC Plus Cement, unconstrained mobile	825	2.40	0.28	[-0.45 , 1.01]	Non-inferiority not shown	0.226
[UNI] MG Uni Unicondylar, fixed	408	11.71	9.58	[7.46 , 11.71]	Inferior by ≥100%	<0.001
[UNI] Oxford Partial Knee Unicondylar, mobile	3,066	12.51	10.38	[9.68 , 11.09]	Inferior by ≥100%	<0.001

For peer review only

Supplementary table 11: Difference in cumulative percentage revision of knee implants compared to a contemporary benchmark at 3 years post primary in men less than 55 years for knee replacements with ≥250 procedures remaining at risk

Knee brand, bearing and constraint	Number at risk	Cumulative failure (%)	Difference in failure (%)	95% CI	Equivalence status	p-value
[TKR] PFC Sigma Bicondylar Knee Cement, unconstrained fixed	2,538	2.85	[REFERENCE]			
[TKR] AGC Cement, unconstrained fixed	915	3.55	0.70	[-0.57 , 1.98]	Non-inferiority not shown	0.140
[TKR] Genesis 2 Cement, unconstrained fixed	311	3.41	0.57	[-1.24 , 2.38]	Non-inferiority not shown	0.270
[TKR] Genesis 2 Oxinium Cement, unconstrained fixed	458	3.15	0.30	[-1.28 , 1.89]	Non-inferiority not shown	0.354
[TKR] LCS Complete Uncemented, unconstrained mobile	331	4.59	1.74	[-0.41 , 3.90]	Non-inferiority not shown	0.057
[TKR] NexGen Cement, posterior-stabilised fixed	761	4.36	1.52	[0.11 , 2.93]	Non-inferiority not shown	0.017
[TKR] NexGen Cement, unconstrained fixed	781	2.92	0.07	[-1.09 , 1.23]	Non-inferiority not shown	0.452
[TKR] PFC Sigma Bicondylar Knee Cement, posterior-stabilised mobile	408	3.30	0.46	[-1.28 , 2.20]	Non-inferiority not shown	0.304
[TKR] PFC Sigma Bicondylar Knee Cement, posterior-stabilised fixed	1,137	3.48	0.64	[-0.49 , 1.76]	Non-inferiority not shown	0.134
[TKR] PFC Sigma Bicondylar Knee Cement, unconstrained mobile	528	3.42	0.57	[-1.01 , 2.15]	Non-inferiority not shown	0.240
[TKR] Triathlon Cement, posterior-stabilised fixed	256	2.79	-0.05	[-1.88 , 1.78]	Non-inferiority not shown	0.477
[TKR] Triathlon Cement, unconstrained fixed	865	2.63	-0.21	[-1.26 , 0.84]	Non-inferiority not shown	0.346
[TKR] Vanguard Cement, unconstrained fixed	584	3.61	0.77	[-0.68 , 2.22]	Non-inferiority not shown	0.150
[UNI] Oxford Partial Knee Unicondylar, mobile	2,693	6.80	3.95	[2.94 , 4.97]	Inferior by ≥100%	<0.001
[UNI] Physica ZUK Unicondylar, fixed	551	2.61	-0.24	[-1.48 , 1.00]	Non-inferiority not shown	0.353
[UNI] Sigma HP Unicondylar, fixed	481	3.99	1.14	[-0.43 , 2.71]	Non-inferiority not shown	0.078

For peer review only

Supplementary table 12: Difference in cumulative percentage revision of knee implants compared to a contemporary benchmark at 5 years post primary in men less than 55 years for knee replacements with ≥250 procedures remaining at risk

Knee brand, bearing and constraint	Number at risk	Cumulative failure (%)	Difference in failure (%)	95% CI	Equivalence status	p-value
[TKR] PFC Sigma Bicondylar Knee Cement, unconstrained fixed	1,753	4.00	[REFERENCE]			
[TKR] AGC Cement, unconstrained fixed	740	5.29	1.29	[-0.30 , 2.88]	Non-inferiority not shown	0.056
[TKR] Genesis 2 Oxinium Cement, unconstrained fixed	325	4.65	0.65	[-1.37 , 2.67]	Non-inferiority not shown	0.263
[TKR] NexGen Cement, posterior-stabilised fixed	520	6.80	2.80	[0.93 , 4.67]	Inferior by ≥20%	0.002
[TKR] NexGen Cement, unconstrained fixed	454	3.75	-0.25	[-1.68 , 1.19]	Non-inferiority not shown	0.368
[TKR] PFC Sigma Bicondylar Knee Cement, posterior-stabilised mobile	306	4.31	0.31	[-1.72 , 2.34]	Non-inferiority not shown	0.383
[TKR] PFC Sigma Bicondylar Knee Cement, posterior-stabilised fixed	796	4.85	0.85	[-0.54 , 2.25]	Non-inferiority not shown	0.115
[TKR] PFC Sigma Bicondylar Knee Cement, unconstrained mobile	415	4.63	0.63	[-1.26 , 2.51]	Non-inferiority not shown	0.258
[TKR] Triathlon Cement, unconstrained fixed	452	3.69	-0.31	[-1.69 , 1.07]	Non-inferiority not shown	0.331
[TKR] Vanguard Cement, unconstrained fixed	255	4.79	0.79	[-0.98 , 2.56]	Non-inferiority not shown	0.190
[UNI] Oxford Partial Knee Unicondylar, mobile	1,972	9.71	5.71	[4.44 , 6.98]	Inferior by ≥100%	<0.001
[UNI] Physica ZUK Unicondylar, fixed	289	4.17	0.17	[-1.57 , 1.92]	Non-inferiority not shown	0.423

For peer review only

Supplementary table 13: Difference in cumulative percentage revision of knee implants compared to a contemporary benchmark at 7 years post primary in men less than 55 years for knee replacements with ≥250 procedures remaining at risk

Knee brand, bearing and constraint	Number at risk	Cumulative failure (%)	Difference in failure (%)	95% CI	Equivalence status	p-value
[TKR] PFC Sigma Bicondylar Knee Cement, unconstrained fixed	1,100	5.11	[REFERENCE]			
[TKR] AGC Cement, unconstrained fixed	500	7.67	2.56	[0.54 , 4.58]	Non-inferiority not shown	0.006
[TKR] NexGen Cement, posterior-stabilised fixed	320	9.92	4.82	[2.32 , 7.31]	Inferior by ≥20%	<0.001
[TKR] PFC Sigma Bicondylar Knee Cement, posterior-stabilised fixed	468	5.92	0.81	[-0.87 , 2.50]	Non-inferiority not shown	0.172
[TKR] PFC Sigma Bicondylar Knee Cement, unconstrained mobile	283	5.48	0.37	[-1.80 , 2.54]	Non-inferiority not shown	0.370
[UNI] Oxford Partial Knee Unicondylar, mobile	1,254	12.89	7.78	[6.21 , 9.35]	Inferior by ≥100%	<0.001

For peer review only

Supplementary table 14: Difference in cumulative percentage revision of knee implants compared to a contemporary benchmark at 3 years post primary in women less than 55 years for knee replacements with ≥250 procedures remaining at risk

Knee brand, bearing and constraint	Number at risk	Cumulative failure (%)	Difference in failure (%)	95% CI	Equivalence status	p-value
[TKR] NexGen Cement, unconstrained fixed	1,105	2.18	[REFERENCE]			
[TKR] AGC Cement, unconstrained fixed	1,331	2.56	0.39	[-0.72 , 1.50]	Non-inferiority not shown	0.248
[TKR] Genesis 2 Cement, unconstrained fixed	472	1.79	-0.38	[-1.65 , 0.89]	Non-inferiority not shown	0.278
[TKR] Genesis 2 Oxinium Cement, posterior-stabilised fixed	301	3.11	0.93	[-0.97 , 2.83]	Non-inferiority not shown	0.168
[TKR] Genesis 2 Oxinium Cement, unconstrained fixed	556	2.66	0.48	[-0.98 , 1.95]	Non-inferiority not shown	0.260
[TKR] Kinemax Cement, unconstrained fixed	266	2.92	0.74	[-1.39 , 2.88]	Non-inferiority not shown	0.248
[TKR] LCS Complete Uncemented, unconstrained mobile	412	3.54	1.36	[-0.46 , 3.19]	Non-inferiority not shown	0.072
[TKR] NexGen Cement, posterior-stabilised fixed	1,253	2.99	0.81	[-0.32 , 1.94]	Non-inferiority not shown	0.080
[TKR] PFC Sigma Bicondylar Knee Cement, posterior-stabilised mobile	436	4.36	2.18	[0.21 , 4.16]	Non-inferiority not shown	0.015
[TKR] PFC Sigma Bicondylar Knee Cement, posterior-stabilised fixed	1,637	3.08	0.90	[-0.18 , 1.99]	Non-inferiority not shown	0.052
[TKR] PFC Sigma Bicondylar Knee Cement, unconstrained fixed	3,827	2.27	0.10	[-0.78 , 0.97]	Non-inferiority not shown	0.415
[TKR] PFC Sigma Bicondylar Knee Cement, unconstrained mobile	520	3.29	1.11	[-0.53 , 2.76]	Non-inferiority not shown	0.092
[TKR] Triathlon Cement, posterior-stabilised fixed	364	3.91	1.74	[-0.17 , 3.64]	Non-inferiority not shown	0.037
[TKR] Triathlon Cement, unconstrained fixed	1,260	2.53	0.35	[-0.71 , 1.42]	Non-inferiority not shown	0.258
[TKR] Vanguard Cement, unconstrained fixed	807	1.39	-0.79	[-1.80 , 0.22]	Non-inferior	0.064
[UNI] Oxford Partial Knee Unicondylar, mobile	3,251	5.92	3.75	[2.70 , 4.80]	Inferior by ≥100%	<0.001
[UNI] Physica ZUK Unicondylar, fixed	526	4.48	2.30	[0.64 , 3.96]	Inferior by ≥20%	0.003
[UNI] Sigma HP Unicondylar, fixed	395	6.28	4.10	[1.96 , 6.25]	Inferior by ≥20%	<0.001

For peer review only

Supplementary table 15: Difference in cumulative percentage revision of knee implants compared to a contemporary benchmark at 5 years post primary in women less than 55 years for knee replacements with ≥250 procedures remaining at risk

Knee brand, bearing and constraint	Number at risk	Cumulative failure (%)	Difference in failure (%)	95% CI	Equivalence status	p-value
[TKR] PFC Sigma Bicondylar Knee Cement, unconstrained fixed	2,522	3.60	[REFERENCE]			
[TKR] AGC Cement, unconstrained fixed	1,072	4.19	0.59	[-0.62 , 1.80]	Non-inferiority not shown	0.168
[TKR] Genesis 2 Cement, unconstrained fixed	282	2.51	-1.09	[-2.51 , 0.32]	Non-inferior	0.065
[TKR] Genesis 2 Oxinium Cement, unconstrained fixed	392	4.68	1.08	[-0.76 , 2.92]	Non-inferiority not shown	0.125
[TKR] Kinemax Cement, unconstrained fixed	258	4.77	1.17	[-1.43 , 3.76]	Non-inferiority not shown	0.189
[TKR] LCS Complete Uncemented, unconstrained mobile	316	5.70	2.10	[-0.17 , 4.37]	Non-inferiority not shown	0.035
[TKR] NexGen Cement, posterior-stabilised fixed	859	4.62	1.02	[-0.23 , 2.27]	Non-inferiority not shown	0.054
[TKR] NexGen Cement, unconstrained fixed	642	3.94	0.34	[-0.92 , 1.60]	Non-inferiority not shown	0.300
[TKR] PFC Sigma Bicondylar Knee Cement, posterior-stabilised mobile	333	5.51	1.91	[-0.23 , 4.06]	Non-inferiority not shown	0.040
[TKR] PFC Sigma Bicondylar Knee Cement, posterior-stabilised fixed	1,153	4.93	1.33	[0.16 , 2.51]	Non-inferiority not shown	0.013
[TKR] PFC Sigma Bicondylar Knee Cement, unconstrained mobile	423	5.73	2.13	[0.08 , 4.18]	Non-inferiority not shown	0.021
[TKR] Triathlon Cement, unconstrained fixed	681	4.15	0.55	[-0.68 , 1.77]	Non-inferiority not shown	0.191
[TKR] Vanguard Cement, unconstrained fixed	341	2.57	-1.03	[-2.27 , 0.21]	Non-inferior	0.052
[UNI] Oxford Partial Knee Unicondylar, mobile	2,358	9.86	6.26	[5.12 , 7.39]	Inferior by ≥100%	<0.001
[UNI] Physica ZUK Unicondylar, fixed	256	6.21	2.61	[0.65 , 4.57]	Non-inferiority not shown	0.005

For peer review only

Supplementary table 16: Difference in cumulative percentage revision of knee implants compared to a contemporary benchmark at 7 years post primary in women less than 55 years for knee replacements with ≥250 procedures remaining at risk

Knee brand, bearing and constraint	Number at risk	Cumulative failure (%)	Difference in failure (%)	95% CI	Equivalence status	p-value
[TKR] PFC Sigma Bicondylar Knee Cement, unconstrained fixed	1,538	4.39	[REFERENCE]			
[TKR] AGC Cement, unconstrained fixed	735	5.15	0.75	[-0.65 , 2.16]	Non-inferiority not shown	0.147
[TKR] NexGen Cement, posterior-stabilised fixed	551	6.60	2.21	[0.55 , 3.86]	Non-inferiority not shown	0.004
[TKR] NexGen Cement, unconstrained fixed	334	5.20	0.81	[-0.84 , 2.46]	Non-inferiority not shown	0.169
[TKR] PFC Sigma Bicondylar Knee Cement, posterior-stabilised fixed	720	5.57	1.18	[-0.16 , 2.51]	Non-inferiority not shown	0.042
[TKR] PFC Sigma Bicondylar Knee Cement, unconstrained mobile	285	7.06	2.67	[0.30 , 5.03]	Non-inferiority not shown	0.014
[TKR] Triathlon Cement, unconstrained fixed	268	5.80	1.40	[-0.32 , 3.13]	Non-inferiority not shown	0.056
[UNI] Oxford Partial Knee Unicondylar, mobile	1,495	13.01	8.62	[7.24 , 10.00]	Inferior by ≥100%	<0.001

For peer review only

Supplementary table 17: Difference in cumulative percentage revision of knee implants compared to a contemporary benchmark at 3 years post primary in men between 55 and 75 years for knee replacements with ≥250 procedures remaining at risk

Knee brand, bearing and constraint	Number at risk	Cumulative failure (%)	Difference in failure (%)	95% CI	Equivalence status	p-value
[TKR] NexGen Cement, unconstrained fixed	10,041	1.34	[REFERENCE]			
[TKR] AGC Cement, unconstrained fixed	14,760	1.71	0.37	[0.09 , 0.65]	Non-inferiority not shown	0.005
[TKR] AGC Hybrid, unconstrained fixed	274	2.77	1.43	[-0.47 , 3.33]	Non-inferiority not shown	0.070
[TKR] AGC Uncemented, unconstrained fixed	402	3.74	2.40	[0.59 , 4.21]	Inferior by ≥20%	0.005
[TKR] Advance MP Cement, unconstrained fixed	1,736	2.43	1.09	[0.41 , 1.77]	Inferior by ≥20%	0.001
[TKR] Columbus Cement, unconstrained fixed	1,543	1.73	0.39	[-0.20 , 0.97]	Non-inferiority not shown	0.096
[TKR] E-Motion Bicondylar Knee Uncemented, unconstrained mobile	516	1.61	0.27	[-0.74 , 1.29]	Non-inferiority not shown	0.299
[TKR] Genesis 2 Cement, posterior-stabilised fixed	2,118	2.39	1.05	[0.45 , 1.64]	Inferior by ≥20%	<0.001
[TKR] Genesis 2 Cement, unconstrained fixed	7,447	1.61	0.27	[-0.05 , 0.59]	Non-inferiority not shown	0.047
[TKR] Genesis 2 Oxinium Cement, posterior-stabilised fixed	447	3.07	1.73	[0.27 , 3.19]	Non-inferiority not shown	0.010
[TKR] Genesis 2 Oxinium Cement, unconstrained fixed	1,105	2.22	0.88	[0.04 , 1.72]	Non-inferiority not shown	0.020
[TKR] Insall-Burstein 2 Cement, posterior-stabilised fixed	684	2.25	0.91	[-0.20 , 2.01]	Non-inferiority not shown	0.054
[TKR] Journey Oxinium Cement, posterior-stabilised fixed	252	3.43	2.09	[-0.12 , 4.29]	Non-inferiority not shown	0.032
[TKR] Kinemax Cement, unconstrained fixed	2,948	2.12	0.78	[0.23 , 1.32]	Non-inferiority not shown	0.003
[TKR] LCS Complete Cement, unconstrained mobile	2,624	1.76	0.42	[-0.09 , 0.94]	Non-inferiority not shown	0.053
[TKR] LCS Complete Uncemented, unconstrained mobile	3,377	1.87	0.53	[0.07 , 0.99]	Non-inferiority not shown	0.012
[TKR] LCS Uncemented, unconstrained mobile	376	2.31	0.97	[-0.53 , 2.47]	Non-inferiority not shown	0.103
[TKR] MRK Cement, unconstrained fixed	1,730	1.62	0.28	[-0.29 , 0.84]	Non-inferiority not shown	0.166
[TKR] Maxim Cement, unconstrained fixed	406	1.44	0.10	[-1.06 , 1.26]	Non-inferiority not shown	0.432
[TKR] NRG Cement, posterior-stabilised fixed	1,179	1.84	0.50	[-0.25 , 1.26]	Non-inferiority not shown	0.096
[TKR] NRG Cement, unconstrained fixed	1,693	2.06	0.72	[0.07 , 1.38]	Non-inferiority not shown	0.015
[TKR] Natural Knee II Cement, unconstrained fixed	712	1.33	-0.01	[-0.85 , 0.83]	Non-inferiority not shown	0.490
[TKR] NexGen Cement, posterior-stabilised fixed	11,497	2.14	0.80	[0.49 , 1.11]	Inferior by ≥20%	<0.001
[TKR] NexGen Hybrid, unconstrained fixed	332	0.56	-0.78	[-1.58 , 0.01]	Non-inferior	0.027
[TKR] NexGen Uncemented, posterior-stabilised fixed	564	1.27	-0.07	[-0.92 , 0.78]	Non-inferiority not shown	0.437
[TKR] NexGen Uncemented, unconstrained fixed	1,469	2.24	0.90	[0.15 , 1.65]	Non-inferiority not shown	0.009
[TKR] Optetrak Cement, posterior-stabilised fixed	417	2.72	1.38	[-0.15 , 2.92]	Non-inferiority not shown	0.038
[TKR] PFC Sigma Bicondylar Knee Cement, posterior-stabilised mobile	1,927	1.93	0.60	[-0.02 , 1.21]	Non-inferiority not shown	0.028
[TKR] PFC Sigma Bicondylar Knee Cement, posterior-stabilised fixed	15,069	1.72	0.38	[0.11 , 0.66]	Non-inferiority not shown	0.003
[TKR] PFC Sigma Bicondylar Knee Cement, unconstrained fixed	37,425	1.49	0.15	[-0.07 , 0.38]	Non-inferiority not shown	0.089
[TKR] PFC Sigma Bicondylar Knee Cement, unconstrained mobile	2,249	1.94	0.60	[0.02 , 1.17]	Non-inferiority not shown	0.021
[TKR] PFC Sigma Bicondylar Knee Hybrid, unconstrained fixed	499	0.97	-0.37	[-1.24 , 0.50]	Non-inferiority not shown	0.202
[TKR] PFC Sigma Bicondylar Knee monobloc polyethylene tibia	1,086	1.39	0.05	[-0.56 , 0.66]	Non-inferiority not shown	0.439
[TKR] Profix Cement, unconstrained fixed	304	2.18	0.84	[-0.77 , 2.44]	Non-inferiority not shown	0.154
[TKR] Profix Uncemented, unconstrained fixed	631	1.23	-0.11	[-0.98 , 0.75]	Non-inferiority not shown	0.399
[TKR] Rotaglide + Cement, unconstrained mobile	468	4.00	2.67	[0.93 , 4.40]	Inferior by ≥20%	0.001
[TKR] Rotaglide Cement, unconstrained mobile	264	3.61	2.27	[0.17 , 4.38]	Non-inferiority not shown	0.017
[TKR] Scorpio Cement, posterior-stabilised mobile	388	1.98	0.64	[-0.73 , 2.02]	Non-inferiority not shown	0.179
[TKR] Scorpio Cement, posterior-stabilised fixed	1,531	2.37	1.03	[0.26 , 1.81]	Non-inferiority not shown	0.004
[TKR] Scorpio Cement, unconstrained fixed	2,891	2.60	1.27	[0.67 , 1.86]	Inferior by ≥20%	<0.001

For peer review only

[TKR] Scorpio Cement, unconstrained mobile	341	1.97	0.63	[-0.83 , 2.09]	Non-inferiority not shown	0.198
[TKR] Scorpio Hybrid, unconstrained fixed	269	2.13	0.79	[-0.91 , 2.49]	Non-inferiority not shown	0.181
[TKR] Scorpio Uncemented, unconstrained fixed	1,184	1.96	0.63	[-0.18 , 1.43]	Non-inferiority not shown	0.063
[TKR] TC Plus Cement, unconstrained fixed	2,427	2.45	1.11	[0.48 , 1.75]	Inferior by ≥20%	<0.001
[TKR] TC Plus Cement, unconstrained mobile	1,442	2.13	0.79	[0.05 , 1.54]	Non-inferiority not shown	0.019
[TKR] TC Plus Uncemented, unconstrained mobile	373	1.03	-0.30	[-1.23 , 0.62]	Non-inferiority not shown	0.260
[TKR] Triathlon Cement, posterior-stabilised fixed	2,263	2.00	0.66	[0.14 , 1.18]	Non-inferiority not shown	0.006
[TKR] Triathlon Cement, unconstrained fixed	8,595	1.44	0.10	[-0.18 , 0.39]	Non-inferiority not shown	0.244
[TKR] Vanguard Cement, posterior-stabilised fixed	833	1.96	0.62	[-0.14 , 1.38]	Non-inferiority not shown	0.054
[TKR] Vanguard Cement, unconstrained fixed	5,651	1.50	0.16	[-0.17 , 0.48]	Non-inferiority not shown	0.169
[UNI] AMC/Uniglide Unicondylar, fixed	335	2.93	1.59	[-0.13 , 3.30]	Non-inferiority not shown	0.035
[UNI] AMC/Uniglide Unicondylar, mobile	458	8.38	7.04	[4.69 , 9.40]	Inferior by ≥100%	<0.001
[UNI] MG Uni Unicondylar, fixed	888	3.13	1.79	[0.65 , 2.93]	Inferior by ≥20%	0.001
[UNI] Oxford Partial Knee Unicondylar, mobile	14,444	3.69	2.35	[2.01 , 2.69]	Inferior by ≥100%	<0.001
[UNI] Physica ZUK Unicondylar, fixed	1,765	2.33	0.99	[0.36 , 1.62]	Inferior by ≥20%	0.001
[UNI] Preservation Unicondylar, fixed	455	4.16	2.82	[1.03 , 4.62]	Inferior by ≥20%	0.001
[UNI] Sigma HP Unicondylar, fixed	1,404	2.97	1.64	[0.89 , 2.38]	Inferior by ≥20%	<0.001

For peer review only

Supplementary table 18: Difference in cumulative percentage revision of knee implants compared to a contemporary benchmark at 5 years post primary in men between 55 and 75 years for knee replacements with ≥250 procedures remaining at risk

Knee brand, bearing and constraint	Number at risk	Cumulative failure (%)	Difference in failure (%)	95% CI	Equivalence status	p-value
[TKR] NexGen Cement, unconstrained fixed	6,119	1.92	[REFERENCE]			
[TKR] AGC Cement, unconstrained fixed	11,861	2.48	0.56	[0.21 , 0.91]	Non-inferiority not shown	0.001
[TKR] AGC Hybrid, unconstrained fixed	258	3.14	1.23	[-0.81 , 3.27]	Non-inferiority not shown	0.119
[TKR] AGC Uncemented, unconstrained fixed	350	4.52	2.60	[0.60 , 4.60]	Inferior by ≥20%	0.005
[TKR] Advance MP Cement, unconstrained fixed	1,248	3.28	1.37	[0.53 , 2.20]	Inferior by ≥20%	0.001
[TKR] Columbus Cement, unconstrained fixed	879	2.61	0.70	[-0.10 , 1.49]	Non-inferiority not shown	0.044
[TKR] E-Motion Bicondylar Knee Uncemented, unconstrained mobile	382	2.28	0.36	[-0.90 , 1.63]	Non-inferiority not shown	0.287
[TKR] Genesis 2 Cement, posterior-stabilised fixed	1,221	3.38	1.47	[0.69 , 2.24]	Inferior by ≥20%	<0.001
[TKR] Genesis 2 Cement, unconstrained fixed	4,599	2.39	0.47	[0.05 , 0.89]	Non-inferiority not shown	0.014
[TKR] Genesis 2 Oxinium Cement, posterior-stabilised fixed	259	4.21	2.29	[0.45 , 4.13]	Inferior by ≥20%	0.007
[TKR] Genesis 2 Oxinium Cement, unconstrained fixed	841	2.94	1.02	[0.02 , 2.03]	Non-inferiority not shown	0.023
[TKR] Insall-Burstein 2 Cement, posterior-stabilised fixed	643	3.70	1.79	[0.37 , 3.21]	Non-inferiority not shown	0.007
[TKR] Kinemax Cement, unconstrained fixed	2,804	3.37	1.46	[0.76 , 2.15]	Inferior by ≥20%	<0.001
[TKR] LCS Complete Cement, unconstrained mobile	2,077	3.22	1.30	[0.58 , 2.02]	Inferior by ≥20%	<0.001
[TKR] LCS Complete Uncemented, unconstrained mobile	2,561	2.43	0.51	[-0.04 , 1.07]	Non-inferiority not shown	0.035
[TKR] LCS Uncemented, unconstrained mobile	359	3.10	1.19	[-0.56 , 2.93]	Non-inferiority not shown	0.092
[TKR] MRK Cement, unconstrained fixed	1,182	2.35	0.43	[-0.30 , 1.16]	Non-inferiority not shown	0.123
[TKR] Maxim Cement, unconstrained fixed	375	2.19	0.27	[-1.17 , 1.71]	Non-inferiority not shown	0.356
[TKR] NRG Cement, posterior-stabilised fixed	893	2.58	0.66	[-0.26 , 1.58]	Non-inferiority not shown	0.080
[TKR] NRG Cement, unconstrained fixed	1,162	3.42	1.51	[0.61 , 2.40]	Inferior by ≥20%	<0.001
[TKR] Natural Knee II Cement, unconstrained fixed	563	1.91	-0.01	[-1.03 , 1.02]	Non-inferiority not shown	0.496
[TKR] NexGen Cement, posterior-stabilised fixed	8,138	3.17	1.25	[0.85 , 1.65]	Inferior by ≥20%	<0.001
[TKR] NexGen Hybrid, unconstrained fixed	282	1.47	-0.45	[-1.75 , 0.86]	Non-inferiority not shown	0.251
[TKR] NexGen Uncemented, posterior-stabilised fixed	415	1.69	-0.22	[-1.27 , 0.82]	Non-inferiority not shown	0.336
[TKR] NexGen Uncemented, unconstrained fixed	1,234	2.97	1.05	[0.17 , 1.94]	Non-inferiority not shown	0.010
[TKR] Optetrak Cement, posterior-stabilised fixed	380	4.38	2.47	[0.52 , 4.41]	Inferior by ≥20%	0.006
[TKR] PFC Sigma Bicondylar Knee Cement, posterior-stabilised mobile	1,449	2.32	0.41	[-0.29 , 1.10]	Non-inferiority not shown	0.125
[TKR] PFC Sigma Bicondylar Knee Cement, posterior-stabilised fixed	10,973	2.29	0.37	[0.03 , 0.72]	Non-inferiority not shown	0.016
[TKR] PFC Sigma Bicondylar Knee Cement, unconstrained fixed	26,348	1.97	0.05	[-0.24 , 0.34]	Non-inferior	0.364
[TKR] PFC Sigma Bicondylar Knee Cement, unconstrained mobile	1,860	2.36	0.45	[-0.21 , 1.11]	Non-inferiority not shown	0.092
[TKR] PFC Sigma Bicondylar Knee Hybrid, unconstrained fixed	454	1.60	-0.32	[-1.45 , 0.81]	Non-inferiority not shown	0.289
[TKR] PFC Sigma Bicondylar Knee monobloc polyethylene tibia	458	1.75	-0.16	[-0.92 , 0.59]	Non-inferiority not shown	0.336
[TKR] Profix Cement, unconstrained fixed	283	4.50	2.59	[0.27 , 4.91]	Non-inferiority not shown	0.014
[TKR] Profix Uncemented, unconstrained fixed	583	1.38	-0.53	[-1.47 , 0.40]	Non-inferiority not shown	0.131
[TKR] Rotaglide + Cement, unconstrained mobile	444	4.84	2.92	[1.02 , 4.83]	Inferior by ≥20%	0.001
[TKR] Scorpio Cement, posterior-stabilised mobile	366	3.00	1.08	[-0.61 , 2.77]	Non-inferiority not shown	0.105
[TKR] Scorpio Cement, posterior-stabilised fixed	1,441	3.55	1.63	[0.68 , 2.58]	Inferior by ≥20%	<0.001
[TKR] Scorpio Cement, unconstrained fixed	2,655	3.57	1.66	[0.94 , 2.37]	Inferior by ≥20%	<0.001
[TKR] Scorpio Cement, unconstrained mobile	325	3.43	1.51	[-0.41 , 3.43]	Non-inferiority not shown	0.062
[TKR] Scorpio Hybrid, unconstrained fixed	258	2.13	0.21	[-1.49 , 1.92]	Non-inferiority not shown	0.403

For peer review only

[TKR] Scorpio Uncemented, unconstrained fixed	1,053	2.82	0.90	[-0.07 , 1.87]	Non-inferiority not shown	0.034
[TKR] TC Plus Cement, unconstrained fixed	2,283	3.23	1.31	[0.58 , 2.05]	Inferior by ≥20%	<0.001
[TKR] TC Plus Cement, unconstrained mobile	1,340	2.89	0.97	[0.09 , 1.85]	Non-inferiority not shown	0.015
[TKR] TC Plus Uncemented, unconstrained mobile	303	1.58	-0.34	[-1.54 , 0.86]	Non-inferiority not shown	0.290
[TKR] Triathlon Cement, posterior-stabilised fixed	1,289	2.64	0.73	[0.07 , 1.38]	Non-inferiority not shown	0.015
[TKR] Triathlon Cement, unconstrained fixed	4,224	1.95	0.04	[-0.33 , 0.41]	Non-inferiority not shown	0.423
[TKR] Vanguard Cement, posterior-stabilised fixed	326	2.90	0.98	[-0.10 , 2.07]	Non-inferiority not shown	0.037
[TKR] Vanguard Cement, unconstrained fixed	2,489	2.10	0.18	[-0.25 , 0.61]	Non-inferiority not shown	0.207
[UNI] AMC/Uniglide Unicondylar, mobile	381	9.21	7.30	[4.82 , 9.77]	Inferior by ≥100%	<0.001
[UNI] MG Uni Unicondylar, fixed	845	4.35	2.43	[1.09 , 3.77]	Inferior by ≥20%	<0.001
[UNI] Oxford Partial Knee Unicondylar, mobile	10,935	5.34	3.42	[2.99 , 3.86]	Inferior by ≥100%	<0.001
[UNI] Physica ZUK Unicondylar, fixed	890	3.82	1.91	[0.99 , 2.83]	Inferior by ≥20%	<0.001
[UNI] Preservation Unicondylar, fixed	428	7.58	5.66	[3.27 , 8.06]	Inferior by ≥100%	<0.001
[UNI] Sigma HP Unicondylar, fixed	642	4.53	2.61	[1.55 , 3.67]	Inferior by ≥20%	<0.001

For peer review only

Supplementary table 19: Difference in cumulative percentage revision of knee implants compared to a contemporary benchmark at 7 years post primary in men between 55 and 75 years for knee replacements with ≥250 procedures remaining at risk

Knee brand, bearing and constraint	Number at risk	Cumulative failure (%)	Difference in failure (%)	95% CI	Equivalence status	p-value
[TKR] PFC Sigma Bicondylar Knee Cement, unconstrained fixed	16,697	2.31	[REFERENCE]			
[TKR] AGC Cement, unconstrained fixed	8,081	3.16	0.85	[0.52 , 1.18]	Inferior by ≥20%	<0.001
[TKR] Advance MP Cement, unconstrained fixed	777	3.82	1.51	[0.60 , 2.43]	Inferior by ≥20%	0.001
[TKR] Columbus Cement, unconstrained fixed	462	2.89	0.58	[-0.28 , 1.45]	Non-inferiority not shown	0.093
[TKR] E-Motion Bicondylar Knee Uncemented, unconstrained mobile	270	2.88	0.57	[-0.92 , 2.07]	Non-inferiority not shown	0.227
[TKR] Genesis 2 Cement, posterior-stabilised fixed	641	4.09	1.78	[0.87 , 2.70]	Inferior by ≥20%	<0.001
[TKR] Genesis 2 Cement, unconstrained fixed	2,429	2.96	0.65	[0.21 , 1.09]	Non-inferiority not shown	0.002
[TKR] Genesis 2 Oxinium Cement, unconstrained fixed	537	3.34	1.03	[-0.04 , 2.11]	Non-inferiority not shown	0.030
[TKR] Insall-Burstein 2 Cement, posterior-stabilised fixed	604	4.47	2.16	[0.61 , 3.71]	Inferior by ≥20%	0.003
[TKR] Kinemax Cement, unconstrained fixed	2,644	4.22	1.91	[1.17 , 2.65]	Inferior by ≥20%	<0.001
[TKR] LCS Complete Cement, unconstrained mobile	1,342	3.84	1.53	[0.76 , 2.30]	Inferior by ≥20%	<0.001
[TKR] LCS Complete Uncemented, unconstrained mobile	1,584	2.97	0.66	[0.05 , 1.27]	Non-inferiority not shown	0.016
[TKR] LCS Uncemented, unconstrained mobile	345	3.10	0.80	[-0.94 , 2.53]	Non-inferiority not shown	0.184
[TKR] MRK Cement, unconstrained fixed	762	3.31	1.00	[0.09 , 1.92]	Non-inferiority not shown	0.016
[TKR] Maxim Cement, unconstrained fixed	314	3.08	0.77	[-0.96 , 2.50]	Non-inferiority not shown	0.192
[TKR] NRG Cement, posterior-stabilised fixed	413	3.08	0.77	[-0.25 , 1.79]	Non-inferiority not shown	0.070
[TKR] NRG Cement, unconstrained fixed	666	3.68	1.37	[0.43 , 2.32]	Non-inferiority not shown	0.002
[TKR] Natural Knee II Cement, unconstrained fixed	417	3.49	1.18	[-0.29 , 2.66]	Non-inferiority not shown	0.057
[TKR] Nexgen Cement, posterior-stabilised fixed	5,295	4.16	1.85	[1.44 , 2.27]	Inferior by ≥20%	<0.001
[TKR] Nexgen Cement, unconstrained fixed	3,522	2.48	0.17	[-0.19 , 0.53]	Non-inferiority not shown	0.180
[TKR] Nexgen Uncemented, ps fixed	313	2.01	-0.30	[-1.49 , 0.89]	Non-inferiority not shown	0.309
[TKR] Nexgen Uncemented, unconstrained fixed	968	3.30	0.99	[0.07 , 1.91]	Non-inferiority not shown	0.017
[TKR] PFC Sigma Bicondylar Knee Cement, PS mobile	915	2.80	0.49	[-0.27 , 1.26]	Non-inferiority not shown	0.103
[TKR] PFC Sigma Bicondylar Knee Cement, posterior-stabilised fixed	6,928	2.64	0.33	[0.03 , 0.63]	Non-inferiority not shown	0.016
[TKR] PFC Sigma Bicondylar Knee Cement, unconstrained mobile	1,328	2.85	0.54	[-0.17 , 1.25]	Non-inferiority not shown	0.067
[TKR] PFC Sigma Bicondylar Knee Hybrid, unconstrained fixed	391	1.60	-0.71	[-1.82 , 0.40]	Non-inferior	0.104
[TKR] Profix Cement, unconstrained fixed	251	5.23	2.92	[0.41 , 5.42]	Non-inferiority not shown	0.011
[TKR] Profix Uncemented, unconstrained fixed	469	1.95	-0.36	[-1.47 , 0.74]	Non-inferiority not shown	0.260
[TKR] Rotaglide + Cement, unconstrained mobile	377	5.78	3.47	[1.38 , 5.56]	Inferior by ≥20%	0.001
[TKR] Scorpio Cement, PS mobile	320	3.00	0.69	[-0.99 , 2.37]	Non-inferiority not shown	0.210
[TKR] Scorpio Cement, posterior-stabilised fixed	1,320	4.58	2.27	[1.22 , 3.33]	Inferior by ≥20%	<0.001
[TKR] Scorpio Cement, unconstrained fixed	2,216	4.36	2.05	[1.29 , 2.81]	Inferior by ≥20%	<0.001
[TKR] Scorpio Cement, unconstrained mobile	302	4.34	2.03	[-0.12 , 4.19]	Non-inferiority not shown	0.032
[TKR] Scorpio Uncemented, unconstrained fixed	740	3.50	1.19	[0.10 , 2.28]	Non-inferiority not shown	0.016
[TKR] TC Plus Cement, unconstrained fixed	1,784	3.69	1.38	[0.62 , 2.15]	Inferior by ≥20%	<0.001
[TKR] TC Plus Cement, unconstrained mobile	1,101	3.50	1.20	[0.24 , 2.15]	Non-inferiority not shown	0.007
[TKR] Triathlon Cement, posterior-stabilised fixed	443	2.81	0.50	[-0.16 , 1.16]	Non-inferiority not shown	0.070
[TKR] Triathlon Cement, unconstrained fixed	1,424	2.43	0.12	[-0.29 , 0.54]	Non-inferiority not shown	0.280
[TKR] Vanguard Cement, unconstrained fixed	749	2.28	-0.03	[-0.46 , 0.40]	Non-inferior	0.449
[UNI] AMC/Uniglide Unicondylar, mobile	253	11.09	8.78	[5.99 , 11.56]	Inferior by ≥100%	<0.001

For peer review only

[UNI] MG Uni Unicondylar, fixed	753	5.64	3.33	[1.82 , 4.85]	Inferior by ≥20%	<0.001
[UNI] Oxford Partial Knee Unicondylar, mobile	7,296	6.84	4.54	[4.09 , 4.98]	Inferior by ≥100%	<0.001
[UNI] Physica ZUK Unicondylar, fixed	432	4.54	2.23	[1.14 , 3.32]	Inferior by ≥20%	<0.001
[UNI] Preservation Unicondylar, fixed	388	9.77	7.46	[4.77 , 10.15]	Inferior by ≥100%	<0.001

For peer review only

Supplementary table 20: Difference in cumulative percentage revision of knee implants compared to a contemporary benchmark at 10 years post primary in men between 55 and 75 years for knee replacements with ≥250 procedures remaining at risk

Knee brand, bearing and constraint	Number at risk	Cumulative failure (%)	Difference in failure (%)	95% CI	Equivalence status	p-value
[TKR] PFC Sigma Bicondylar Knee Cement, unconstrained fixed	6,049	2.72	[REFERENCE]			
[TKR] AGC Cement, unconstrained fixed	2,803	4.43	1.71	[1.24 , 2.18]	Inferior by ≥20%	<0.001
[TKR] Genesis 2 Cement, unconstrained fixed	621	3.54	0.82	[0.22 , 1.42]	Non-inferiority not shown	0.004
[TKR] Insall-Burstein 2 Cement, posterior-stabilised fixed	362	7.30	4.58	[2.50 , 6.66]	Inferior by ≥20%	<0.001
[TKR] Kinemax Cement, unconstrained fixed	1,648	5.26	2.54	[1.69 , 3.38]	Inferior by ≥20%	<0.001
[TKR] LCS Complete Uncemented, unconstrained mobile	471	3.52	0.80	[0.06 , 1.54]	Non-inferiority not shown	0.017
[TKR] LCS Uncemented, unconstrained mobile	317	3.10	0.38	[-1.36 , 2.12]	Non-inferiority not shown	0.333
[TKR] Nexgen Cement, posterior-stabilised fixed	1,814	5.64	2.92	[2.33 , 3.51]	Inferior by ≥20%	<0.001
[TKR] Nexgen Cement, unconstrained fixed	890	2.85	0.13	[-0.34 , 0.61]	Non-inferiority not shown	0.293
[TKR] Nexgen Uncemented, unconstrained fixed	265	3.42	0.70	[-0.25 , 1.66]	Non-inferiority not shown	0.075
[TKR] PFC Sigma Bicondylar Knee Cement, posterior-stabilised fixed	2,369	3.13	0.41	[0.03 , 0.78]	Non-inferiority not shown	0.018
[TKR] PFC Sigma Bicondylar Knee Cement, unconstrained mobile	322	3.19	0.47	[-0.32 , 1.26]	Non-inferiority not shown	0.122
[TKR] Scorpio Cement, posterior-stabilised fixed	611	5.47	2.75	[1.56 , 3.95]	Inferior by ≥20%	<0.001
[TKR] Scorpio Cement, unconstrained fixed	1,044	4.99	2.27	[1.43 , 3.12]	Inferior by ≥20%	<0.001
[TKR] TC Plus Cement, unconstrained fixed	456	4.51	1.79	[0.81 , 2.78]	Inferior by ≥20%	<0.001
[TKR] TC Plus Cement, unconstrained mobile	527	4.48	1.76	[0.61 , 2.90]	Inferior by ≥20%	0.001
[UNI] MG Uni Unicondylar, fixed	346	7.79	5.07	[3.18 , 6.97]	Inferior by ≥100%	<0.001
[UNI] Oxford Partial Knee Unicondylar, mobile	2,454	9.97	7.25	[6.57 , 7.93]	Inferior by ≥100%	<0.001

For peer review only

Supplementary table 21: Difference in cumulative percentage revision of knee implants compared to a contemporary benchmark at 3 years post primary in women between 55 and 75 years for knee replacements with ≥250 procedures remaining at risk

Knee brand, bearing and constraint	Number at risk	Cumulative failure (%)	Difference in failure (%)	95% CI	Equivalence status	p-value
[TKR] TC Plus Cement, unconstrained mobile	1,695	1.000	[REFERENCE]			
[TKR] AGC Cement, unconstrained fixed	18,655	1.500	0.510	[0.02 , 0.99]	Non-inferiority not shown	0.021
[TKR] AGC Hybrid, unconstrained fixed	297	2.600	1.600	[-0.24 , 3.43]	Non-inferiority not shown	0.044
[TKR] AGC Uncemented, unconstrained fixed	377	5.720	4.730	[2.41 , 7.04]	Inferior by ≥100%	<0.001
[TKR] Advance MP Cement, unconstrained fixed	2,023	1.800	0.810	[0.10 , 1.52]	Non-inferiority not shown	0.013
[TKR] Advance MP Stature Cement, unconstrained fixed	494	1.540	0.540	[-0.51 , 1.59]	Non-inferiority not shown	0.158
[TKR] Advance PS Cement, posterior-stabilised fixed	282	2.730	1.730	[-0.09 , 3.55]	Non-inferiority not shown	0.031
[TKR] Columbus Cement, unconstrained fixed	1,895	1.810	0.810	[0.12 , 1.50]	Non-inferiority not shown	0.011
[TKR] E-Motion Bicondylar Knee Cement, unconstrained mobile	311	3.320	2.320	[0.47 , 4.16]	Inferior by ≥20%	0.007
[TKR] E-Motion Bicondylar Knee Uncemented, unconstrained mobile	495	2.220	1.220	[-0.06 , 2.51]	Non-inferiority not shown	0.031
[TKR] FS Cement, unconstrained fixed	253	1.160	0.160	[-1.22 , 1.55]	Non-inferiority not shown	0.408
[TKR] Genesis 2 Cement, posterior-stabilised fixed	3,026	1.770	0.770	[0.16 , 1.39]	Non-inferiority not shown	0.006
[TKR] Genesis 2 Cement, unconstrained fixed	9,401	1.200	0.200	[-0.30 , 0.70]	Non-inferiority not shown	0.213
[TKR] Genesis 2 Cement, unconstrained mobile	331	1.480	0.480	[-0.89 , 1.85]	Non-inferiority not shown	0.245
[TKR] Genesis 2 Oxinium Cement, posterior-stabilised fixed	615	2.210	1.210	[0.09 , 2.32]	Non-inferiority not shown	0.017
[TKR] Genesis 2 Oxinium Cement, unconstrained fixed	1,426	1.160	0.160	[-0.52 , 0.84]	Non-inferiority not shown	0.324
[TKR] Insall-Burstein 2 Cement, posterior-stabilised fixed	812	1.450	0.450	[-0.49 , 1.38]	Non-inferiority not shown	0.174
[TKR] Journey Oxinium Cement, posterior-stabilised fixed	311	4.310	3.310	[1.05 , 5.56]	Inferior by ≥100%	0.002
[TKR] Kinemax Cement, unconstrained fixed	3,607	2.160	1.170	[0.51 , 1.82]	Inferior by ≥20%	<0.001
[TKR] LCS Complete Cement, unconstrained mobile	3,170	1.850	0.850	[0.21 , 1.49]	Inferior by ≥20%	0.005
[TKR] LCS Complete Uncemented, unconstrained mobile	3,736	1.750	0.750	[0.15 , 1.35]	Non-inferiority not shown	0.007
[TKR] LCS Uncemented, unconstrained mobile	458	2.330	1.330	[-0.11 , 2.76]	Non-inferiority not shown	0.035
[TKR] MRK Cement, unconstrained fixed	2,339	1.070	0.070	[-0.52 , 0.66]	Non-inferiority not shown	0.407
[TKR] Maxim Cement, unconstrained fixed	478	1.840	0.840	[-0.44 , 2.11]	Non-inferiority not shown	0.099
[TKR] NRG Cement, posterior-stabilised fixed	1,574	2.030	1.030	[0.21 , 1.84]	Inferior by ≥20%	0.007
[TKR] NRG Cement, unconstrained fixed	2,181	1.410	0.410	[-0.24 , 1.06]	Non-inferiority not shown	0.107
[TKR] Natural Knee II Cement, unconstrained fixed	965	2.070	1.070	[0.08 , 2.06]	Non-inferiority not shown	0.017
[TKR] NexGen Cement, posterior-stabilised mobile	324	2.070	1.070	[-0.42 , 2.57]	Non-inferiority not shown	0.080
[TKR] NexGen Cement, posterior-stabilised fixed	15,772	1.490	0.490	[-0.00 , 0.98]	Non-inferiority not shown	0.025
[TKR] NexGen Cement, unconstrained fixed	13,193	1.030	0.030	[-0.45 , 0.52]	Non-inferiority not shown	0.447
[TKR] NexGen Hybrid, unconstrained fixed	333	1.150	0.160	[-1.06 , 1.37]	Non-inferiority not shown	0.401
[TKR] NexGen Uncemented, posterior-stabilised fixed	419	1.420	0.420	[-0.72 , 1.57]	Non-inferiority not shown	0.235
[TKR] NexGen Uncemented, unconstrained fixed	1,110	2.720	1.720	[0.69 , 2.76]	Inferior by ≥20%	0.001
[TKR] Optetrak Cement, posterior-stabilised fixed	579	3.140	2.140	[0.68 , 3.60]	Inferior by ≥20%	0.002
[TKR] PFC Sigma Bicondylar Knee Cement, posterior-stabilised mobile	2,132	1.940	0.940	[0.22 , 1.65]	Inferior by ≥20%	0.005
[TKR] PFC Sigma Bicondylar Knee Cement, posterior-stabilised fixed	20,665	1.600	0.600	[0.12 , 1.09]	Non-inferiority not shown	0.007
[TKR] PFC Sigma Bicondylar Knee Cement, unconstrained fixed	47,826	1.240	0.240	[-0.23 , 0.71]	Non-inferiority not shown	0.158
[TKR] PFC Sigma Bicondylar Knee Cement, unconstrained mobile	2,332	1.510	0.520	[-0.14 , 1.17]	Non-inferiority not shown	0.062
[TKR] PFC Sigma Bicondylar Knee Hybrid, unconstrained fixed	557	1.570	0.570	[-0.54 , 1.69]	Non-inferiority not shown	0.158
[TKR] PFC Sigma Bicondylar Knee monobloc polyethylene tibia	1,703	1.490	0.490	[-0.18 , 1.15]	Non-inferiority not shown	0.077

For peer review only

[TKR] Profix Cement, unconstrained fixed	335	1.170	0.180	[-1.06 , 1.41]	Non-inferiority not shown	0.390
[TKR] Profix Oxinium monobloc polyethylene tibia	270	2.180	1.180	[-0.60 , 2.96]	Non-inferiority not shown	0.098
[TKR] Profix Uncemented, unconstrained fixed	680	1.710	0.720	[-0.35 , 1.78]	Non-inferiority not shown	0.094
[TKR] Rotaglide + Cement, unconstrained mobile	597	2.120	1.120	[-0.11 , 2.35]	Non-inferiority not shown	0.037
[TKR] Rotaglide Cement, unconstrained mobile	397	1.760	0.760	[-0.53 , 2.06]	Non-inferiority not shown	0.124
[TKR] Scorpio Cement, posterior-stabilised mobile	483	1.210	0.210	[-0.85 , 1.28]	Non-inferiority not shown	0.349
[TKR] Scorpio Cement, posterior-stabilised fixed	2,156	1.450	0.450	[-0.23 , 1.13]	Non-inferiority not shown	0.097
[TKR] Scorpio Cement, unconstrained fixed	3,812	1.580	0.580	[-0.02 , 1.18]	Non-inferiority not shown	0.029
[TKR] Scorpio Cement, unconstrained mobile	462	2.310	1.310	[-0.11 , 2.74]	Non-inferiority not shown	0.035
[TKR] Scorpio Hybrid, unconstrained fixed	373	1.810	0.810	[-0.59 , 2.22]	Non-inferiority not shown	0.129
[TKR] Scorpio Uncemented, unconstrained fixed	1,215	1.900	0.900	[0.02 , 1.79]	Non-inferiority not shown	0.022
[TKR] TC Plus Cement, unconstrained fixed	2,740	1.660	0.670	[0.01 , 1.32]	Non-inferiority not shown	0.024
[TKR] TC Plus Uncemented, unconstrained mobile	565	1.810	0.810	[-0.31 , 1.92]	Non-inferiority not shown	0.078
[TKR] Triathlon Cement, posterior-stabilised fixed	3,116	1.680	0.680	[0.08 , 1.29]	Non-inferiority not shown	0.013
[TKR] Triathlon Cement, unconstrained fixed	11,030	1.580	0.580	[0.08 , 1.08]	Non-inferiority not shown	0.011
[TKR] Triathlon Uncemented, unconstrained fixed	262	2.860	1.860	[-0.05 , 3.77]	Non-inferiority not shown	0.028
[TKR] Vanguard Cement, posterior-stabilised fixed	1,116	2.040	1.040	[0.22 , 1.85]	Inferior by ≥20%	0.006
[TKR] Vanguard Cement, unconstrained fixed	7,465	1.380	0.380	[-0.13 , 0.89]	Non-inferiority not shown	0.072
[UNI] AMC/Uniglide Unicondylar, fixed	390	3.200	2.200	[0.49 , 3.92]	Inferior by ≥20%	0.006
[UNI] AMC/Uniglide Unicondylar, mobile	407	7.070	6.070	[3.66 , 8.47]	Inferior by ≥100%	<0.001
[UNI] MG Uni Unicondylar, fixed	673	3.990	2.990	[1.47 , 4.51]	Inferior by ≥100%	<0.001
[UNI] Oxford Partial Knee Unicondylar, mobile	12,307	3.830	2.830	[2.28 , 3.38]	Inferior by ≥100%	<0.001
[UNI] Physica ZUK Unicondylar, fixed	1,431	2.090	1.090	[0.30 , 1.89]	Inferior by ≥20%	0.003
[UNI] Preservation Unicondylar, fixed	333	8.230	7.230	[4.37 , 10.09]	Inferior by ≥100%	<0.001
[UNI] Sigma HP Unicondylar, fixed	959	3.090	2.090	[1.09 , 3.09]	Inferior by ≥100%	<0.001
[UNI] Sled Unicondylar, fixed	264	6.670	5.680	[2.81 , 8.54]	Inferior by ≥100%	<0.001

For peer review only

Supplementary table 22: Difference in cumulative percentage revision of knee implants compared to a contemporary benchmark at 5 years post primary in women between 55 and 75 years for knee replacements with ≥250 procedures remaining at risk

Knee brand, bearing and constraint	Number at risk	Cumulative failure (%)	Difference in failure (%)	95% CI	Equivalence status	p-value
[TKR] MRK Cement, unconstrained fixed	1,647	1.47	[REFERENCE]			
[TKR] AGC Cement, unconstrained fixed	15,179	2.11	0.64	[0.14 , 1.14]	Non-inferiority not shown	0.006
[TKR] AGC Hybrid, unconstrained fixed	282	2.93	1.46	[-0.48 , 3.40]	Non-inferiority not shown	0.071
[TKR] AGC Uncemented, unconstrained fixed	358	6.74	5.27	[2.77 , 7.77]	Inferior by ≥100%	<0.001
[TKR] Advance MP Cement, unconstrained fixed	1,520	2.45	0.98	[0.19 , 1.78]	Non-inferiority not shown	0.008
[TKR] Advance MP Stature Cement, unconstrained fixed	267	2.49	1.02	[-0.38 , 2.42]	Non-inferiority not shown	0.077
[TKR] Columbus Cement, unconstrained fixed	1,052	2.83	1.36	[0.50 , 2.23]	Inferior by ≥20%	0.001
[TKR] E-Motion Bicondylar Knee Uncemented, unconstrained mobile	387	2.88	1.41	[-0.07 , 2.88]	Non-inferiority not shown	0.031
[TKR] Genesis 2 Cement, posterior-stabilised fixed	1,842	2.39	0.92	[0.24 , 1.60]	Non-inferiority not shown	0.004
[TKR] Genesis 2 Cement, unconstrained fixed	5,902	1.59	0.12	[-0.39 , 0.64]	Non-inferiority not shown	0.320
[TKR] Genesis 2 Cement, unconstrained mobile	301	1.78	0.31	[-1.18 , 1.79]	Non-inferiority not shown	0.342
[TKR] Genesis 2 Oxinium Cement, posterior-stabilised fixed	391	3.33	1.86	[0.44 , 3.28]	Inferior by ≥20%	0.005
[TKR] Genesis 2 Oxinium Cement, unconstrained fixed	1,039	2.11	0.63	[-0.23 , 1.50]	Non-inferiority not shown	0.075
[TKR] Insall-Burstein 2 Cement, posterior-stabilised fixed	775	3.28	1.81	[0.51 , 3.11]	Inferior by ≥20%	0.003
[TKR] Kinemax Cement, unconstrained fixed	3,479	2.96	1.49	[0.78 , 2.20]	Inferior by ≥20%	<0.001
[TKR] LCS Complete Cement, unconstrained mobile	2,534	2.91	1.44	[0.70 , 2.17]	Inferior by ≥20%	<0.001
[TKR] LCS Complete Uncemented, unconstrained mobile	2,848	2.79	1.32	[0.63 , 2.01]	Inferior by ≥20%	<0.001
[TKR] LCS Uncemented, unconstrained mobile	438	2.98	1.51	[-0.10 , 3.11]	Non-inferiority not shown	0.033
[TKR] Maxim Cement, unconstrained fixed	432	2.48	1.01	[-0.45 , 2.47]	Non-inferiority not shown	0.088
[TKR] NRG Cement, posterior-stabilised fixed	1,187	2.73	1.26	[0.34 , 2.18]	Inferior by ≥20%	0.004
[TKR] NRG Cement, unconstrained fixed	1,577	2.12	0.65	[-0.10 , 1.40]	Non-inferiority not shown	0.044
[TKR] Natural Knee II Cement, unconstrained fixed	773	3.08	1.61	[0.43 , 2.80]	Inferior by ≥20%	0.004
[TKR] NexGen Cement, posterior-stabilised mobile	275	3.40	1.93	[-0.03 , 3.90]	Non-inferiority not shown	0.027
[TKR] NexGen Cement, posterior-stabilised fixed	11,170	2.61	1.14	[0.62 , 1.66]	Inferior by ≥20%	<0.001
[TKR] NexGen Cement, unconstrained fixed	8,293	1.58	0.11	[-0.40 , 0.61]	Non-inferiority not shown	0.338
[TKR] NexGen Hybrid, unconstrained fixed	303	1.46	-0.01	[-1.36 , 1.34]	Non-inferiority not shown	0.493
[TKR] NexGen Uncemented, posterior-stabilised fixed	339	1.68	0.21	[-1.04 , 1.47]	Non-inferiority not shown	0.370
[TKR] NexGen Uncemented, unconstrained fixed	1,007	3.46	1.98	[0.84 , 3.13]	Inferior by ≥20%	<0.001
[TKR] Optetrak Cement, posterior-stabilised fixed	508	5.04	3.57	[1.75 , 5.38]	Inferior by ≥100%	<0.001
[TKR] PFC Sigma Bicondylar Knee Cement, posterior-stabilised mobile	1,587	2.74	1.27	[0.45 , 2.08]	Inferior by ≥20%	0.001
[TKR] PFC Sigma Bicondylar Knee Cement, posterior-stabilised fixed	15,223	2.26	0.78	[0.29 , 1.28]	Non-inferiority not shown	0.001
[TKR] PFC Sigma Bicondylar Knee Cement, unconstrained fixed	34,361	1.75	0.28	[-0.19 , 0.75]	Non-inferiority not shown	0.121
[TKR] PFC Sigma Bicondylar Knee Cement, unconstrained mobile	1,974	2.28	0.81	[0.06 , 1.56]	Non-inferiority not shown	0.017
[TKR] PFC Sigma Bicondylar Knee Hybrid, unconstrained fixed	523	1.76	0.28	[-0.89 , 1.46]	Non-inferiority not shown	0.318
[TKR] PFC Sigma Bicondylar Knee monobloc polyethylene tibia	721	2.00	0.53	[-0.25 , 1.32]	Non-inferiority not shown	0.092
[TKR] Profix Cement, unconstrained fixed	315	2.08	0.61	[-0.98 , 2.20]	Non-inferiority not shown	0.228
[TKR] Profix Oxinium monobloc polyethylene tibia	262	2.91	1.44	[-0.60 , 3.48]	Non-inferiority not shown	0.083
[TKR] Profix Uncemented, unconstrained fixed	619	2.16	0.69	[-0.49 , 1.86]	Non-inferiority not shown	0.125
[TKR] Rotaglide + Cement, unconstrained mobile	571	2.78	1.31	[-0.07 , 2.69]	Non-inferiority not shown	0.032
[TKR] Rotaglide Cement, unconstrained mobile	299	3.20	1.72	[-0.07 , 3.52]	Non-inferiority not shown	0.030

For peer review only

[TKR] Scorpio Cement, posterior-stabilised mobile	455	2.04	0.57	[-0.76 , 1.90]	Non-inferiority not shown	0.201
[TKR] Scorpio Cement, posterior-stabilised fixed	2,061	2.23	0.76	[-0.01 , 1.53]	Non-inferiority not shown	0.026
[TKR] Scorpio Cement, unconstrained fixed	3,488	2.43	0.96	[0.29 , 1.62]	Non-inferiority not shown	0.002
[TKR] Scorpio Cement, unconstrained mobile	445	3.17	1.70	[0.05 , 3.34]	Non-inferiority not shown	0.022
[TKR] Scorpio Hybrid, unconstrained fixed	356	2.35	0.88	[-0.71 , 2.47]	Non-inferiority not shown	0.138
[TKR] Scorpio Uncemented, unconstrained fixed	1,106	2.49	1.02	[0.04 , 2.00]	Non-inferiority not shown	0.021
[TKR] TC Plus Cement, unconstrained fixed	2,588	2.39	0.92	[0.19 , 1.65]	Non-inferiority not shown	0.007
[TKR] TC Plus Cement, unconstrained mobile	1,584	1.48	0.01	[-0.72 , 0.74]	Non-inferiority not shown	0.488
[TKR] TC Plus Uncemented, unconstrained mobile	456	2.74	1.27	[-0.11 , 2.64]	Non-inferiority not shown	0.035
[TKR] Triathlon Cement, posterior-stabilised fixed	1,765	2.61	1.14	[0.43 , 1.85]	Inferior by ≥20%	0.001
[TKR] Triathlon Cement, unconstrained fixed	5,497	2.07	0.60	[0.08 , 1.12]	Non-inferiority not shown	0.011
[TKR] Vanguard Cement, posterior-stabilised fixed	426	2.94	1.46	[0.44 , 2.49]	Inferior by ≥20%	0.003
[TKR] Vanguard Cement, unconstrained fixed	3,246	2.20	0.72	[0.16 , 1.28]	Non-inferiority not shown	0.006
[UNI] AMC/Uniglide Unicondylar, fixed	275	4.59	3.12	[1.04 , 5.20]	Inferior by ≥20%	0.002
[UNI] AMC/Uniglide Unicondylar, mobile	346	9.71	8.23	[5.43 , 11.04]	Inferior by ≥100%	<0.001
[UNI] MG Uni Unicondylar, fixed	651	5.28	3.81	[2.09 , 5.53]	Inferior by ≥100%	<0.001
[UNI] Oxford Partial Knee Unicondylar, mobile	9,400	6.04	4.57	[3.96 , 5.18]	Inferior by ≥100%	<0.001
[UNI] Physica ZUK Unicondylar, fixed	754	3.52	2.05	[1.00 , 3.11]	Inferior by ≥20%	<0.001
[UNI] Preservation Unicondylar, fixed	318	11.84	10.37	[7.01 , 13.72]	Inferior by ≥100%	<0.001
[UNI] Sigma HP Unicondylar, fixed	446	5.02	3.54	[2.09 , 5.00]	Inferior by ≥100%	<0.001

For peer review only

Supplementary table 23: Difference in cumulative percentage revision of knee implants compared to a contemporary benchmark at 7 years post primary in women between 55 and 75 years for knee replacements with ≥250 procedures remaining at risk

Knee brand, bearing and constraint	Number at risk	Cumulative failure (%)	Difference in failure (%)	95% CI	Equivalence status	p-value
[TKR] TC Plus Cement, unconstrained mobile	1,341	1.95	[REFERENCE]			
[TKR] AGC Cement, unconstrained fixed	10,578	2.61	0.66	[-0.04 , 1.36]	Non-inferiority not shown	0.032
[TKR] AGC Uncemented, unconstrained fixed	278	8.16	6.21	[3.41 , 9.00]	Inferior by ≥100%	<0.001
[TKR] Advance MP Cement, unconstrained fixed	1,054	3.35	1.40	[0.35 , 2.45]	Non-inferiority not shown	0.005
[TKR] Columbus Cement, unconstrained fixed	585	2.95	1.00	[-0.01 , 2.01]	Non-inferiority not shown	0.026
[TKR] E-Motion Bicondylar Knee Uncemented, unconstrained mobile	279	4.06	2.11	[0.19 , 4.03]	Non-inferiority not shown	0.015
[TKR] Genesis 2 Cement, posterior-stabilised fixed	938	2.62	0.67	[-0.19 , 1.53]	Non-inferiority not shown	0.063
[TKR] Genesis 2 Cement, unconstrained fixed	3,140	2.06	0.11	[-0.62 , 0.84]	Non-inferiority not shown	0.383
[TKR] Genesis 2 Oxinium Cement, unconstrained fixed	632	2.69	0.74	[-0.37 , 1.85]	Non-inferiority not shown	0.096
[TKR] Insall-Burstein 2 Cement, posterior-stabilised fixed	738	4.30	2.35	[0.81 , 3.89]	Inferior by ≥20%	0.001
[TKR] Kinemax Cement, unconstrained fixed	3,294	3.96	2.01	[1.10 , 2.93]	Inferior by ≥20%	<0.001
[TKR] LCS Complete Cement, unconstrained mobile	1,676	3.88	1.94	[0.97 , 2.90]	Inferior by ≥20%	<0.001
[TKR] LCS Complete Uncemented, unconstrained mobile	1,824	3.09	1.14	[0.28 , 2.01]	Non-inferiority not shown	0.005
[TKR] LCS Uncemented, unconstrained mobile	425	2.98	1.03	[-0.64 , 2.70]	Non-inferiority not shown	0.114
[TKR] MRK Cement, unconstrained fixed	1,125	2.02	0.07	[-0.82 , 0.96]	Non-inferiority not shown	0.438
[TKR] Maxim Cement, unconstrained fixed	344	3.50	1.55	[-0.27 , 3.36]	Non-inferiority not shown	0.047
[TKR] NRG Cement, posterior-stabilised fixed	572	3.26	1.31	[0.17 , 2.45]	Non-inferiority not shown	0.012
[TKR] NRG Cement, unconstrained fixed	920	2.41	0.46	[-0.47 , 1.39]	Non-inferiority not shown	0.165
[TKR] Natural Knee II Cement, unconstrained fixed	584	3.82	1.87	[0.45 , 3.29]	Inferior by ≥20%	0.005
[TKR] NexGen Cement, posterior-stabilised fixed	7,194	3.32	1.37	[0.65 , 2.10]	Inferior by ≥20%	<0.001
[TKR] NexGen Cement, unconstrained fixed	5,031	2.15	0.21	[-0.51 , 0.92]	Non-inferiority not shown	0.286
[TKR] NexGen Uncemented, posterior-stabilised fixed	251	2.36	0.41	[-1.22 , 2.04]	Non-inferiority not shown	0.310
[TKR] NexGen Uncemented, unconstrained fixed	909	4.05	2.10	[0.78 , 3.43]	Inferior by ≥20%	0.001
[TKR] Optetrak Cement, posterior-stabilised fixed	319	5.23	3.28	[1.37 , 5.20]	Inferior by ≥20%	<0.001
[TKR] PFC Sigma Bicondylar Knee Cement, posterior-stabilised mobile	1,016	3.32	1.37	[0.35 , 2.40]	Non-inferiority not shown	0.004
[TKR] PFC Sigma Bicondylar Knee Cement, posterior-stabilised fixed	9,914	2.66	0.72	[0.02 , 1.41]	Non-inferiority not shown	0.022
[TKR] PFC Sigma Bicondylar Knee Cement, unconstrained fixed	22,205	2.06	0.11	[-0.56 , 0.78]	Non-inferiority not shown	0.375
[TKR] PFC Sigma Bicondylar Knee Cement, unconstrained mobile	1,414	2.45	0.50	[-0.40 , 1.41]	Non-inferiority not shown	0.138
[TKR] PFC Sigma Bicondylar Knee Hybrid, unconstrained fixed	453	1.76	-0.19	[-1.46 , 1.07]	Non-inferiority not shown	0.383
[TKR] PFC Sigma Bicondylar Knee monobloc polyethylene tibia	265	2.00	0.06	[-0.86 , 0.98]	Non-inferiority not shown	0.451
[TKR] Profix Cement, unconstrained fixed	289	2.73	0.78	[-1.10 , 2.66]	Non-inferiority not shown	0.208
[TKR] Profix Uncemented, unconstrained fixed	508	2.16	0.21	[-1.05 , 1.48]	Non-inferiority not shown	0.370
[TKR] Rotaglide + Cement, unconstrained mobile	497	3.13	1.19	[-0.35 , 2.72]	Non-inferiority not shown	0.065
[TKR] Scorpio Cement, posterior-stabilised mobile	393	2.50	0.55	[-1.00 , 2.09]	Non-inferiority not shown	0.243
[TKR] Scorpio Cement, posterior-stabilised fixed	1,896	2.92	0.97	[0.00 , 1.94]	Non-inferiority not shown	0.025
[TKR] Scorpio Cement, unconstrained fixed	2,905	2.86	0.92	[0.07 , 1.77]	Non-inferiority not shown	0.017
[TKR] Scorpio Cement, unconstrained mobile	415	4.49	2.54	[0.55 , 4.53]	Inferior by ≥20%	0.006
[TKR] Scorpio Hybrid, unconstrained fixed	324	2.94	0.99	[-0.85 , 2.82]	Non-inferiority not shown	0.145
[TKR] Scorpio Uncemented, unconstrained fixed	820	3.17	1.22	[0.03 , 2.41]	Non-inferiority not shown	0.023
[TKR] TC Plus Cement, unconstrained fixed	2,030	2.99	1.04	[0.12 , 1.96]	Non-inferiority not shown	0.013

For peer review only

[TKR] TC Plus Uncemented, unconstrained mobile	330	2.98	1.03	[-0.49 , 2.56]	Non-inferiority not shown	0.092
[TKR] Triathlon Cement, posterior-stabilised fixed	633	2.91	0.96	[0.04 , 1.88]	Non-inferiority not shown	0.020
[TKR] Triathlon Cement, unconstrained fixed	1,918	2.48	0.53	[-0.20 , 1.26]	Non-inferiority not shown	0.079
[TKR] Vanguard Cement, unconstrained fixed	1,009	2.74	0.80	[-0.02 , 1.61]	Non-inferiority not shown	0.027
[UNI] AMC/Uniglide Unicondylar, mobile	254	11.38	9.44	[6.34 , 12.53]	Inferior by ≥100%	<0.001
[UNI] MG Uni Unicondylar, fixed	584	6.64	4.69	[2.72 , 6.66]	Inferior by ≥100%	<0.001
[UNI] Oxford Partial Knee Unicondylar, mobile	6,459	8.09	6.14	[5.31 , 6.97]	Inferior by ≥100%	<0.001
[UNI] Physica ZUK Unicondylar, fixed	327	6.01	4.06	[2.32 , 5.81]	Inferior by ≥100%	<0.001
[UNI] Preservation Unicondylar, fixed	292	14.69	12.74	[9.02 , 16.45]	Inferior by ≥100%	<0.001

For peer review only

Supplementary table 24: Difference in cumulative percentage revision of knee implants compared to a contemporary benchmark at 10 years post primary in women between 55 and 75 years for knee replacements with ≥250 procedures remaining at risk

Knee brand, bearing and constraint	Number at risk	Cumulative failure (%)	Difference in failure (%)	95% CI	Equivalence status	p-value
[TKR] PFC Sigma Bicondylar Knee Cement, unconstrained fixed	8,221	2.4	[REFERENCE]			
[TKR] AGC Cement, unconstrained fixed	3,879	3.4	1.0	[0.65 , 1.35]	Inferior by ≥20%	<0.001
[TKR] Advance MP Cement, unconstrained fixed	299	4.4	2.0	[0.77 , 3.13]	Inferior by ≥20%	0.001
[TKR] Genesis 2 Cement, unconstrained fixed	853	2.4	0.0	[-0.45 , 0.41]	Non-inferior	0.462
[TKR] Insall-Burstein 2 Cement, posterior-stabilised fixed	489	5.3	2.9	[1.34 , 4.49]	Inferior by ≥20%	<0.001
[TKR] Kinemax Cement, unconstrained fixed	2,094	5.4	3.0	[2.20 , 3.76]	Inferior by ≥20%	<0.001
[TKR] LCS Complete Cement, unconstrained mobile	352	4.9	2.5	[1.50 , 3.53]	Inferior by ≥20%	<0.001
[TKR] LCS Complete Uncemented, unconstrained mobile	531	3.5	1.1	[0.43 , 1.76]	Non-inferiority not shown	0.001
[TKR] LCS Uncemented, unconstrained mobile	393	3.0	0.6	[-0.96 , 2.13]	Non-inferiority not shown	0.231
[TKR] NexGen Cement, posterior-stabilised fixed	2,484	4.4	2.0	[1.51 , 2.40]	Inferior by ≥20%	<0.001
[TKR] NexGen Cement, unconstrained fixed	1,217	2.7	0.3	[-0.14 , 0.72]	Non-inferiority not shown	0.095
[TKR] NexGen Uncemented, unconstrained fixed	322	4.5	2.1	[0.83 , 3.34]	Inferior by ≥20%	0.001
[TKR] PFC Sigma Bicondylar Knee Cement, posterior-stabilised mobile	313	3.3	0.9	[0.13 , 1.72]	Non-inferiority not shown	0.012
[TKR] PFC Sigma Bicondylar Knee Cement, posterior-stabilised fixed	3,360	3.3	0.9	[0.52 , 1.19]	Inferior by ≥20%	<0.001
[TKR] PFC Sigma Bicondylar Knee Cement, unconstrained mobile	338	3.1	0.7	[-0.15 , 1.45]	Non-inferiority not shown	0.056
[TKR] PFC Sigma Bicondylar Knee Hybrid, unconstrained fixed	270	2.0	-0.4	[-1.58 , 0.81]	Non-inferiority not shown	0.263
[TKR] Rotaglide + Cement, unconstrained mobile	289	4.8	2.4	[0.56 , 4.24]	Inferior by ≥20%	0.005
[TKR] Scorpio Cement, posterior-stabilised fixed	842	3.7	1.3	[0.42 , 2.13]	Non-inferiority not shown	0.002
[TKR] Scorpio Cement, unconstrained fixed	1,414	3.6	1.2	[0.52 , 1.83]	Inferior by ≥20%	<0.001
[TKR] TC Plus Cement, unconstrained fixed	504	3.6	1.2	[0.35 , 2.00]	Non-inferiority not shown	0.003
[TKR] TC Plus Cement, unconstrained mobile	635	2.5	0.1	[-0.71 , 0.92]	Non-inferiority not shown	0.399
[UNI] MG Uni Unicondylar, fixed	287	8.8	6.4	[4.12 , 8.61]	Inferior by ≥100%	<0.001
[UNI] Oxford Partial Knee Unicondylar, mobile	2,271	11.9	9.5	[8.75 , 10.29]	Inferior by ≥100%	<0.001

For peer review only

Supplementary table 25: Difference in cumulative percentage revision of knee implants compared to a contemporary benchmark at 3 years post primary in men over 75 years for knee replacements with ≥250 procedures remaining at risk

Knee brand, bearing and constraint	Number at risk	Cumulative failure (%)	Difference in failure (%)	95% CI	Equivalence status	p-value
[TKR] NexGen Cement, unconstrained fixed	3,575	0.63	[REFERENCE]			
[TKR] AGC Cement, unconstrained fixed	5,857	1.06	0.43	[0.11 , 0.76]	Non-inferiority not shown	0.004
[TKR] Advance MP Cement, unconstrained fixed	586	1.71	1.08	[0.13 , 2.02]	Inferior by ≥20%	0.013
[TKR] Columbus Cement, unconstrained fixed	541	1.58	0.96	[0.07 , 1.85]	Non-inferiority not shown	0.018
[TKR] Genesis 2 Cement, posterior-stabilised fixed	845	0.61	-0.02	[-0.49 , 0.46]	Non-inferiority not shown	0.468
[TKR] Genesis 2 Cement, unconstrained fixed	3,120	0.92	0.29	[-0.06 , 0.64]	Non-inferiority not shown	0.050
[TKR] Insall-Burstein 2 Cement, posterior-stabilised fixed	270	0.35	-0.28	[-0.99 , 0.44]	Non-inferiority not shown	0.223
[TKR] Kinemax Cement, unconstrained fixed	1,108	1.07	0.44	[-0.18 , 1.06]	Non-inferiority not shown	0.081
[TKR] LCS Complete Cement, unconstrained mobile	825	0.71	0.08	[-0.49 , 0.65]	Non-inferiority not shown	0.391
[TKR] LCS Complete Uncemented, unconstrained mobile	999	0.81	0.19	[-0.34 , 0.71]	Non-inferiority not shown	0.244
[TKR] MRK Cement, unconstrained fixed	644	1.02	0.39	[-0.31 , 1.10]	Non-inferiority not shown	0.138
[TKR] NRG Cement, posterior-stabilised fixed	447	1.60	0.97	[-0.15 , 2.09]	Non-inferiority not shown	0.045
[TKR] NRG Cement, unconstrained fixed	646	0.84	0.21	[-0.44 , 0.86]	Non-inferiority not shown	0.265
[TKR] Natural Knee II Cement, unconstrained fixed	250	0.00	--	[-.-- , -.--]	No failures to date	
[TKR] NexGen Cement, posterior-stabilised fixed	4,136	1.00	0.37	[0.03 , 0.71]	Non-inferiority not shown	0.016
[TKR] NexGen Uncemented, unconstrained fixed	267	0.68	0.05	[-0.91 , 1.02]	Non-inferiority not shown	0.456
[TKR] PFC Sigma Bicondylar Knee Cement, posterior-stabilised mobile	318	2.09	1.46	[0.01 , 2.91]	Non-inferiority not shown	0.024
[TKR] PFC Sigma Bicondylar Knee Cement, posterior-stabilised fixed	6,056	1.11	0.49	[0.17 , 0.81]	Inferior by ≥20%	0.001
[TKR] PFC Sigma Bicondylar Knee Cement, unconstrained fixed	13,197	0.84	0.22	[-0.04 , 0.47]	Non-inferiority not shown	0.048
[TKR] PFC Sigma Bicondylar Knee Cement, unconstrained mobile	375	1.53	0.90	[-0.25 , 2.05]	Non-inferiority not shown	0.062
[TKR] PFC Sigma Bicondylar Knee monobloc polyethylene tibia	1,004	1.26	0.64	[0.01 , 1.26]	Non-inferiority not shown	0.023
[TKR] Profix Uncemented, unconstrained fixed	296	0.63	0.00	[-0.89 , 0.89]	Non-inferiority not shown	0.498
[TKR] Scorpio Cement, posterior-stabilised fixed	624	0.89	0.26	[-0.48 , 1.00]	Non-inferiority not shown	0.246
[TKR] Scorpio Cement, unconstrained fixed	1,038	1.15	0.53	[-0.13 , 1.19]	Non-inferiority not shown	0.059
[TKR] Scorpio Uncemented, unconstrained fixed	373	0.52	-0.11	[-0.86 , 0.64]	Non-inferiority not shown	0.386
[TKR] TC Plus Cement, unconstrained fixed	831	1.54	0.91	[0.08 , 1.74]	Non-inferiority not shown	0.016
[TKR] TC Plus Cement, unconstrained mobile	418	1.02	0.39	[-0.52 , 1.31]	Non-inferiority not shown	0.201
[TKR] Triathlon Cement, posterior-stabilised fixed	764	0.78	0.15	[-0.38 , 0.68]	Non-inferiority not shown	0.289
[TKR] Triathlon Cement, unconstrained fixed	2,911	1.01	0.38	[0.02 , 0.74]	Non-inferiority not shown	0.018
[TKR] Vanguard Cement, posterior-stabilised fixed	291	0.36	-0.26	[-0.82 , 0.30]	Non-inferiority not shown	0.178
[TKR] Vanguard Cement, unconstrained fixed	1,887	0.97	0.34	[-0.07 , 0.75]	Non-inferiority not shown	0.053
[UNI] Oxford Partial Knee Unicondylar, mobile	2,355	2.45	1.82	[1.23 , 2.42]	Inferior by ≥100%	<0.001

For peer review only

Supplementary table 26: Difference in cumulative percentage revision of knee implants compared to a contemporary benchmark at 5 years post primary in men over 75 years for knee replacements with ≥250 procedures remaining at risk

Knee brand, bearing and constraint	Number at risk	Cumulative failure (%)	Difference in failure (%)	95% CI	Equivalence status	p-value
[TKR] Nexgen Cement, unconstrained fixed	2,068	0.90	[REFERENCE]			
[TKR] AGC Cement, unconstrained fixed	4,270	1.31	0.41	[0.01 , 0.81]	Non-inferiority not shown	0.022
[TKR] Advance MP Cement, unconstrained fixed	362	2.15	1.25	[0.11 , 2.40]	Non-inferiority not shown	0.016
[TKR] Columbus Cement, unconstrained fixed	273	1.58	0.69	[-0.22 , 1.60]	Non-inferiority not shown	0.070
[TKR] Genesis 2 Cement, posterior-stabilised fixed	445	1.04	0.14	[-0.56 , 0.84]	Non-inferiority not shown	0.350
[TKR] Genesis 2 Cement, unconstrained fixed	1,766	1.51	0.62	[0.11 , 1.12]	Non-inferiority not shown	0.008
[TKR] Kinemax Cement, unconstrained fixed	949	1.35	0.46	[-0.26 , 1.18]	Non-inferiority not shown	0.107
[TKR] LCS Complete Cement, unconstrained mobile	585	1.14	0.25	[-0.53 , 1.02]	Non-inferiority not shown	0.266
[TKR] LCS Complete Uncemented, unconstrained mobile	681	1.02	0.12	[-0.50 , 0.75]	Non-inferiority not shown	0.348
[TKR] MRK Cement, unconstrained fixed	413	1.18	0.28	[-0.51 , 1.07]	Non-inferiority not shown	0.245
[TKR] NRG Cement, posterior-stabilised fixed	303	2.34	1.44	[0.04 , 2.85]	Non-inferiority not shown	0.022
[TKR] NRG Cement, unconstrained fixed	378	0.84	-0.06	[-0.74 , 0.62]	Non-inferiority not shown	0.432
[TKR] Nexgen Cement, posterior-stabilised fixed	2,698	1.37	0.47	[0.03 , 0.91]	Non-inferiority not shown	0.017
[TKR] PFC Sigma Bicondylar Knee Cement, posterior-stabilised fixed	4,060	1.31	0.41	[0.02 , 0.80]	Non-inferiority not shown	0.019
[TKR] PFC Sigma Bicondylar Knee Cement, unconstrained fixed	8,777	1.02	0.12	[-0.20 , 0.44]	Non-inferiority not shown	0.234
[TKR] PFC Sigma Bicondylar Knee Cement, unconstrained mobile	292	2.41	1.51	[-0.01 , 3.03]	Non-inferiority not shown	0.026
[TKR] PFC Sigma Bicondylar Knee monobloc polyethylene tibia	431	1.40	0.51	[-0.20 , 1.21]	Non-inferiority not shown	0.080
[TKR] Scorpio Cement, posterior-stabilised fixed	543	1.39	0.49	[-0.46 , 1.44]	Non-inferiority not shown	0.154
[TKR] Scorpio Cement, unconstrained fixed	861	1.56	0.67	[-0.13 , 1.46]	Non-inferiority not shown	0.050
[TKR] Scorpio Uncemented, unconstrained fixed	304	0.79	-0.11	[-1.04 , 0.82]	Non-inferiority not shown	0.409
[TKR] TC Plus Cement, unconstrained fixed	719	1.79	0.89	[-0.02 , 1.81]	Non-inferiority not shown	0.028
[TKR] TC Plus Cement, unconstrained mobile	353	1.26	0.36	[-0.68 , 1.40]	Non-inferiority not shown	0.249
[TKR] Triathlon Cement, posterior-stabilised fixed	423	1.34	0.44	[-0.41 , 1.29]	Non-inferiority not shown	0.153
[TKR] Triathlon Cement, unconstrained fixed	1,328	1.39	0.49	[0.01 , 0.97]	Non-inferiority not shown	0.024
[TKR] Vanguard Cement, unconstrained fixed	749	1.34	0.44	[-0.10 , 0.99]	Non-inferiority not shown	0.054
[UNI] Oxford Partial Knee Unicondylar, mobile	1,681	3.30	2.40	[1.67 , 3.14]	Inferior by ≥100%	<0.001

For peer review only

Supplementary table 27: Difference in cumulative percentage revision of knee implants compared to a contemporary benchmark at 7 years post primary in men over 75 years for knee replacements with ≥250 procedures remaining at risk

Knee brand, bearing and constraint	Number at risk	Cumulative failure (%)	Difference in failure (%)	95% CI	Equivalence status	p-value
[TKR] NexGen Cement, unconstrained fixed	1,099	1.11	[REFERENCE]			
[TKR] AGC Cement, unconstrained fixed	2,540	1.54	0.43	[-0.05 , 0.91]	Non-inferiority not shown	0.040
[TKR] Genesis 2 Cement, unconstrained fixed	846	1.78	0.67	[0.06 , 1.28]	Non-inferiority not shown	0.015
[TKR] Kinemax Cement, unconstrained fixed	790	1.82	0.71	[-0.17 , 1.58]	Non-inferiority not shown	0.057
[TKR] LCS Complete Cement, unconstrained mobile	356	1.32	0.21	[-0.66 , 1.09]	Non-inferiority not shown	0.317
[TKR] LCS Complete Uncemented, unconstrained mobile	376	1.53	0.42	[-0.46 , 1.31]	Non-inferiority not shown	0.174
[TKR] NexGen Cement, posterior-stabilised fixed	1,561	1.60	0.49	[-0.03 , 1.02]	Non-inferiority not shown	0.033
[TKR] PFC Sigma Bicondylar Knee Cement, posterior-stabilised fixed	2,298	1.59	0.48	[-0.00 , 0.96]	Non-inferiority not shown	0.026
[TKR] PFC Sigma Bicondylar Knee Cement, unconstrained fixed	4,935	1.15	0.04	[-0.35 , 0.44]	Non-inferiority not shown	0.417
[TKR] Scorpio Cement, posterior-stabilised fixed	418	1.83	0.72	[-0.42 , 1.86]	Non-inferiority not shown	0.109
[TKR] Scorpio Cement, unconstrained fixed	634	1.56	0.45	[-0.37 , 1.27]	Non-inferiority not shown	0.140
[TKR] TC Plus Cement, unconstrained fixed	499	1.79	0.68	[-0.26 , 1.62]	Non-inferiority not shown	0.079
[TKR] TC Plus Cement, unconstrained mobile	261	1.58	0.47	[-0.76 , 1.70]	Non-inferiority not shown	0.228
[TKR] Triathlon Cement, unconstrained fixed	381	1.85	0.74	[-0.01 , 1.48]	Non-inferiority not shown	0.027
[UNI] Oxford Partial Knee Unicondylar, mobile	975	4.75	3.64	[2.64 , 4.64]	Inferior by ≥100%	<0.001

For peer review only

Supplementary table 28: Difference in cumulative percentage revision of knee implants compared to a contemporary benchmark at 10 years post primary in men over 75 years for knee replacements with ≥250 procedures remaining at risk

Knee brand, bearing and constraint	Number at risk	Cumulative failure (%)	Difference in failure (%)	95% CI	Equivalence status	p-value
[TKR] PFC Sigma Bicondylar Knee Cement, unconstrained fixed	1,319	1.25	[REFERENCE]			
[TKR] AGC Cement, unconstrained fixed	660	1.79	0.54	[0.08 , 1.00]	Non-inferiority not shown	0.011
[TKR] Kinemax Cement, unconstrained fixed	359	2.30	1.05	[0.05 , 2.06]	Non-inferiority not shown	0.019
[TKR] NexGen Cement, posterior-stabilised fixed	385	2.00	0.75	[0.12 , 1.39]	Non-inferiority not shown	0.010
[TKR] PFC Sigma Bicondylar Knee Cement, posterior-stabilised fixed	590	2.04	0.80	[0.23 , 1.36]	Non-inferiority not shown	0.003
[UNI] Oxford Partial Knee Unicondylar, mobile	290	5.90	4.65	[3.41 , 5.90]	Inferior by ≥100%	<0.001

For peer review only

Supplementary table 29: Difference in cumulative percentage revision of knee implants compared to a contemporary benchmark at 3 years post primary in women over 75 years for knee replacements with ≥250 procedures remaining at risk

Knee brand, bearing and constraint	Number at risk	Cumulative failure (%)	Difference in failure (%)	95% CI	Equivalence status	p-value
[TKR] Scorpio Cement, posterior-stabilised fixed	1,132	0.58	[REFERENCE]			
[TKR] AGC Cement, unconstrained fixed	9,844	0.75	0.17	[-0.28 , 0.63]	Non-inferiority not shown	0.229
[TKR] Advance MP Cement, unconstrained fixed	846	1.21	0.63	[-0.15 , 1.41]	Non-inferiority not shown	0.057
[TKR] Columbus Cement, unconstrained fixed	858	1.56	0.99	[0.18 , 1.80]	Inferior by ≥20%	0.009
[TKR] Genesis 2 Cement, posterior-stabilised fixed	1,628	0.96	0.39	[-0.19 , 0.96]	Non-inferiority not shown	0.095
[TKR] Genesis 2 Cement, unconstrained fixed	4,807	0.91	0.33	[-0.16 , 0.81]	Non-inferiority not shown	0.092
[TKR] Insall-Burstein 2 Cement, posterior-stabilised fixed	378	1.01	0.43	[-0.64 , 1.51]	Non-inferiority not shown	0.214
[TKR] Kinemax Cement, unconstrained fixed	1,824	0.63	0.05	[-0.50 , 0.61]	Non-inferiority not shown	0.426
[TKR] LCS Complete Cement, unconstrained mobile	1,434	1.12	0.54	[-0.11 , 1.19]	Non-inferiority not shown	0.050
[TKR] LCS Complete Uncemented, unconstrained mobile	1,444	0.97	0.39	[-0.23 , 1.02]	Non-inferiority not shown	0.110
[TKR] MRK Cement, unconstrained fixed	1,124	0.62	0.04	[-0.53 , 0.62]	Non-inferiority not shown	0.443
[TKR] NRG Cement, posterior-stabilised fixed	692	0.65	0.07	[-0.64 , 0.78]	Non-inferiority not shown	0.421
[TKR] NRG Cement, unconstrained fixed	991	0.42	-0.16	[-0.72 , 0.41]	Non-inferiority not shown	0.295
[TKR] Natural Knee II Cement, unconstrained fixed	439	0.44	-0.13	[-0.88 , 0.61]	Non-inferiority not shown	0.361
[TKR] NexGen Cement, posterior-stabilised fixed	7,284	0.84	0.27	[-0.20 , 0.73]	Non-inferiority not shown	0.129
[TKR] NexGen Cement, unconstrained fixed	5,865	0.67	0.10	[-0.37 , 0.56]	Non-inferiority not shown	0.343
[TKR] NexGen Uncemented, unconstrained fixed	259	1.09	0.51	[-0.78 , 1.80]	Non-inferiority not shown	0.220
[TKR] Optetrak Cement, posterior-stabilised fixed	253	1.46	0.88	[-0.60 , 2.37]	Non-inferiority not shown	0.121
[TKR] PFC Sigma Bicondylar Knee Cement, posterior-stabilised mobile	514	0.82	0.24	[-0.59 , 1.07]	Non-inferiority not shown	0.287
[TKR] PFC Sigma Bicondylar Knee Cement, posterior-stabilised fixed	10,390	0.80	0.22	[-0.23 , 0.68]	Non-inferiority not shown	0.167
[TKR] PFC Sigma Bicondylar Knee Cement, unconstrained fixed	21,321	0.69	0.12	[-0.32 , 0.55]	Non-inferiority not shown	0.299
[TKR] PFC Sigma Bicondylar Knee Cement, unconstrained mobile	573	1.29	0.72	[-0.22 , 1.66]	Non-inferiority not shown	0.068
[TKR] PFC Sigma Bicondylar Knee monobloc polyethylene tibia	1,500	0.82	0.24	[-0.33 , 0.81]	Non-inferiority not shown	0.205
[TKR] Profix Uncemented, unconstrained fixed	479	0.98	0.41	[-0.55 , 1.37]	Non-inferiority not shown	0.202
[TKR] Rotaglide + Cement, unconstrained mobile	276	2.03	1.46	[-0.21 , 3.12]	Non-inferiority not shown	0.043
[TKR] Scorpio Cement, unconstrained fixed	1,946	1.08	0.50	[-0.12 , 1.12]	Non-inferiority not shown	0.055
[TKR] Scorpio Uncemented, unconstrained fixed	602	1.27	0.69	[-0.28 , 1.66]	Non-inferiority not shown	0.082
[TKR] TC Plus Cement, unconstrained fixed	1,222	0.78	0.21	[-0.44 , 0.85]	Non-inferiority not shown	0.265
[TKR] TC Plus Cement, unconstrained mobile	684	0.66	0.09	[-0.63 , 0.80]	Non-inferiority not shown	0.408
[TKR] TC Plus Uncemented, unconstrained mobile	278	1.33	0.75	[-0.61 , 2.12]	Non-inferiority not shown	0.140
[TKR] Triathlon Cement, posterior-stabilised fixed	1,437	1.17	0.59	[-0.04 , 1.22]	Non-inferiority not shown	0.033
[TKR] Triathlon Cement, unconstrained fixed	4,541	1.14	0.56	[0.07 , 1.06]	Non-inferiority not shown	0.013
[TKR] Vanguard Cement, posterior-stabilised fixed	524	1.67	1.10	[0.13 , 2.06]	Inferior by ≥20%	0.013
[TKR] Vanguard Cement, unconstrained fixed	3,003	0.97	0.39	[-0.11 , 0.90]	Non-inferiority not shown	0.064
[UNI] Oxford Partial Knee Unicondylar, mobile	2,333	3.46	2.89	[2.10 , 3.68]	Inferior by ≥100%	<0.001

For peer review only

Supplementary table 30: Difference in cumulative percentage revision of knee implants compared to a contemporary benchmark at 5 years post primary in women over 75 years for knee replacements with ≥250 procedures remaining at risk

Knee brand, bearing and constraint	Number at risk	Cumulative failure (%)	Difference in failure (%)	95% CI	Equivalence status	p-value
[TKR] NexGen Cement, unconstrained fixed	3,677	0.77	[REFERENCE]			
[TKR] AGC Cement, unconstrained fixed	7,591	1.03	0.27	[-0.01 , 0.54]	Non-inferiority not shown	0.031
[TKR] Advance MP Cement, unconstrained fixed	598	2.15	1.39	[0.41 , 2.36]	Inferior by ≥20%	0.003
[TKR] Columbus Cement, unconstrained fixed	453	1.68	0.91	[0.16 , 1.67]	Inferior by ≥20%	0.009
[TKR] Genesis 2 Cement, posterior-stabilised fixed	928	1.35	0.58	[0.03 , 1.13]	Non-inferiority not shown	0.019
[TKR] Genesis 2 Cement, unconstrained fixed	2,845	1.04	0.27	[-0.06 , 0.60]	Non-inferiority not shown	0.052
[TKR] Insall-Burstein 2 Cement, posterior-stabilised fixed	349	1.55	0.79	[-0.46 , 2.03]	Non-inferiority not shown	0.109
[TKR] Kinemax Cement, unconstrained fixed	1,634	0.97	0.20	[-0.29 , 0.69]	Non-inferiority not shown	0.209
[TKR] LCS Complete Cement, unconstrained mobile	1,039	1.75	0.98	[0.30 , 1.66]	Inferior by ≥20%	0.002
[TKR] LCS Complete Uncemented, unconstrained mobile	1,043	1.21	0.44	[-0.13 , 1.01]	Non-inferiority not shown	0.064
[TKR] MRK Cement, unconstrained fixed	772	0.89	0.13	[-0.41 , 0.66]	Non-inferiority not shown	0.321
[TKR] NRG Cement, posterior-stabilised fixed	485	0.82	0.05	[-0.63 , 0.74]	Non-inferiority not shown	0.442
[TKR] NRG Cement, unconstrained fixed	626	0.56	-0.20	[-0.71 , 0.30]	Non-inferiority not shown	0.218
[TKR] Natural Knee II Cement, unconstrained fixed	348	0.44	-0.32	[-0.97 , 0.32]	Non-inferiority not shown	0.161
[TKR] NexGen Cement, posterior-stabilised fixed	4,965	1.19	0.42	[0.11 , 0.73]	Non-inferiority not shown	0.004
[TKR] PFC Sigma Bicondylar Knee Cement, posterior-stabilised mobile	363	1.55	0.78	[-0.33 , 1.89]	Non-inferiority not shown	0.083
[TKR] PFC Sigma Bicondylar Knee Cement, posterior-stabilised fixed	7,316	1.13	0.36	[0.08 , 0.64]	Non-inferiority not shown	0.006
[TKR] PFC Sigma Bicondylar Knee Cement, unconstrained fixed	14,883	0.94	0.17	[-0.06 , 0.40]	Non-inferiority not shown	0.077
[TKR] PFC Sigma Bicondylar Knee Cement, unconstrained mobile	457	1.68	0.91	[-0.10 , 1.93]	Non-inferiority not shown	0.039
[TKR] PFC Sigma Bicondylar Knee monobloc polyethylene tibia	747	1.09	0.32	[-0.21 , 0.85]	Non-inferiority not shown	0.116
[TKR] Profix Uncemented, unconstrained fixed	413	1.21	0.44	[-0.54 , 1.43]	Non-inferiority not shown	0.188
[TKR] Scorpio Cement, posterior-stabilised fixed	1,004	0.77	0.00	[-0.54 , 0.54]	Non-inferiority not shown	0.499
[TKR] Scorpio Cement, unconstrained fixed	1,668	1.45	0.69	[0.12 , 1.25]	Non-inferiority not shown	0.008
[TKR] Scorpio Uncemented, unconstrained fixed	532	1.97	1.20	[0.08 , 2.33]	Non-inferiority not shown	0.018
[TKR] TC Plus Cement, unconstrained fixed	1,097	1.20	0.44	[-0.20 , 1.07]	Non-inferiority not shown	0.090
[TKR] TC Plus Cement, unconstrained mobile	588	1.27	0.50	[-0.35 , 1.36]	Non-inferiority not shown	0.123
[TKR] Triathlon Cement, posterior-stabilised fixed	780	1.87	1.10	[0.40 , 1.81]	Inferior by ≥20%	0.001
[TKR] Triathlon Cement, unconstrained fixed	2,200	1.45	0.68	[0.31 , 1.05]	Inferior by ≥20%	<0.001
[TKR] Vanguard Cement, unconstrained fixed	1,253	1.18	0.42	[0.03 , 0.81]	Non-inferiority not shown	0.018
[UNI] Oxford Partial Knee Unicondylar, mobile	1,802	4.68	3.91	[3.09 , 4.74]	Inferior by ≥100%	<0.001

For peer review only

Supplementary table 31: Difference in cumulative percentage revision of knee implants compared to a contemporary benchmark at 7 years post primary in women over 75 years for knee replacements with ≥250 procedures remaining at risk

Knee brand, bearing and constraint	Number at risk	Cumulative failure (%)	Difference in failure (%)	95% CI	Equivalence status	p-value
[TKR] NexGen Cement, unconstrained fixed	2,110	0.98	[REFERENCE]			
[TKR] AGC Cement, unconstrained fixed	4,857	1.28	0.30	[-0.04 , 0.65]	Non-inferiority not shown	0.043
[TKR] Advance MP Cement, unconstrained fixed	383	2.15	1.17	[0.18 , 2.16]	Non-inferiority not shown	0.010
[TKR] Genesis 2 Cement, posterior-stabilised fixed	452	1.99	1.01	[0.15 , 1.87]	Non-inferiority not shown	0.011
[TKR] Genesis 2 Cement, unconstrained fixed	1,415	1.11	0.13	[-0.25 , 0.51]	Non-inferiority not shown	0.252
[TKR] Insall-Burstein 2 Cement, posterior-stabilised fixed	295	1.55	0.57	[-0.69 , 1.83]	Non-inferiority not shown	0.188
[TKR] Kinemax Cement, unconstrained fixed	1,416	1.23	0.24	[-0.33 , 0.82]	Non-inferiority not shown	0.201
[TKR] LCS Complete Cement, unconstrained mobile	653	1.85	0.87	[0.14 , 1.60]	Non-inferiority not shown	0.010
[TKR] LCS Complete Uncemented, unconstrained mobile	591	1.44	0.46	[-0.22 , 1.13]	Non-inferiority not shown	0.092
[TKR] MRK Cement, unconstrained fixed	494	1.03	0.05	[-0.57 , 0.66]	Non-inferiority not shown	0.440
[TKR] NRG Cement, unconstrained fixed	295	0.56	-0.42	[-0.95 , 0.11]	Non-inferior	0.062
[TKR] Natural Knee II Cement, unconstrained fixed	251	0.84	-0.14	[-1.16 , 0.87]	Non-inferiority not shown	0.391
[TKR] NexGen Cement, posterior-stabilised fixed	2,942	1.54	0.56	[0.16 , 0.96]	Non-inferiority not shown	0.003
[TKR] PFC Sigma Bicondylar Knee Cement, posterior-stabilised fixed	4,344	1.31	0.33	[-0.01 , 0.67]	Non-inferiority not shown	0.029
[TKR] PFC Sigma Bicondylar Knee Cement, unconstrained fixed	9,161	1.05	0.07	[-0.22 , 0.36]	Non-inferiority not shown	0.320
[TKR] PFC Sigma Bicondylar Knee Cement, unconstrained mobile	314	1.95	0.96	[-0.18 , 2.11]	Non-inferiority not shown	0.050
[TKR] PFC Sigma Bicondylar Knee monobloc polyethylene tibia	252	1.43	0.45	[-0.42 , 1.31]	Non-inferiority not shown	0.156
[TKR] Profix Uncemented, unconstrained fixed	300	1.21	0.23	[-0.77 , 1.23]	Non-inferiority not shown	0.327
[TKR] Scorpio Cement, posterior-stabilised fixed	842	0.97	-0.01	[-0.64 , 0.61]	Non-inferiority not shown	0.482
[TKR] Scorpio Cement, unconstrained fixed	1,272	1.65	0.67	[0.04 , 1.29]	Non-inferiority not shown	0.018
[TKR] Scorpio Uncemented, unconstrained fixed	350	1.97	0.99	[-0.15 , 2.12]	Non-inferiority not shown	0.044
[TKR] TC Plus Cement, unconstrained fixed	824	1.29	0.31	[-0.37 , 0.99]	Non-inferiority not shown	0.184
[TKR] TC Plus Cement, unconstrained mobile	456	1.27	0.29	[-0.58 , 1.16]	Non-inferiority not shown	0.257
[TKR] Triathlon Cement, posterior-stabilised fixed	260	2.31	1.33	[0.38 , 2.27]	Inferior by ≥20%	0.003
[TKR] Triathlon Cement, unconstrained fixed	665	1.51	0.52	[0.11 , 0.94]	Non-inferiority not shown	0.007
[TKR] Vanguard Cement, unconstrained fixed	366	1.39	0.41	[-0.10 , 0.92]	Non-inferiority not shown	0.059
[UNI] Oxford Partial Knee Unicondylar, mobile	1,146	6.42	5.44	[4.38 , 6.49]	Inferior by ≥100%	<0.001

For peer review only

Supplementary table 32: Difference in cumulative percentage revision of knee implants compared to a contemporary benchmark at 10 years post primary in women over 75 years for knee replacements with ≥250 procedures remaining at risk

Knee brand, bearing and constraint	Number at risk	Cumulative failure (%)	Difference in failure (%)	95% CI	Equivalence status	p-value
[TKR] PFC Sigma Bicondylar Knee Cement, unconstrained fixed	2,749	1.29	[REFERENCE]			
[TKR] AGC Cement, unconstrained fixed	1,448	1.48	0.19	[-0.15 , 0.54]	Non-inferiority not shown	0.136
[TKR] Genesis 2 Cement, unconstrained fixed	280	1.41	0.12	[-0.36 , 0.61]	Non-inferiority not shown	0.310
[TKR] Kinemax Cement, unconstrained fixed	691	1.55	0.27	[-0.40 , 0.93]	Non-inferiority not shown	0.216
[TKR] NexGen Cement, posterior-stabilised fixed	851	1.77	0.48	[0.07 , 0.90]	Non-inferiority not shown	0.011
[TKR] NexGen Cement, unconstrained fixed	447	1.08	-0.21	[-0.56 , 0.14]	Non-inferior	0.117
[TKR] PFC Sigma Bicondylar Knee Cement, posterior-stabilised fixed	1,202	1.46	0.18	[-0.15 , 0.50]	Non-inferiority not shown	0.148
[TKR] Scorpio Cement, posterior-stabilised fixed	305	1.72	0.44	[-0.54 , 1.41]	Non-inferiority not shown	0.191
[TKR] Scorpio Cement, unconstrained fixed	449	2.07	0.78	[0.04 , 1.52]	Non-inferiority not shown	0.019
[UNI] Oxford Partial Knee Unicondylar, mobile	356	8.61	7.32	[5.87 , 8.78]	Inferior by ≥100%	<0.001

For peer review only

The RECORD statement – checklist of items, extended from the STROBE statement, that should be reported in observational studies using routinely collected health data.

	Item No.	STROBE items	Location in manuscript where items are reported	RECORD items	Location in manuscript where items are reported
**Title and abstract**					
	1	(a) Indicate the study's design with a commonly used term in the title or the abstract (b) Provide in the abstract an informative and balanced summary of what was done and what was found	Title page 1 and abstract page 2	RECORD 1.1: The type of data used should be specified in the title or abstract. When possible, the name of the databases used should be included.<br><br>RECORD 1.2: If applicable, the geographic region and timeframe within which the study took place should be reported in the title or abstract.<br><br>RECORD 1.3: If linkage between databases was conducted for the study, this should be clearly stated in the title or abstract.	Title, abstract page 2.<br><br>N/A
**Introduction**					
Background rationale	2	Explain the scientific background and rationale for the investigation being reported	Introduction Page 4 and 5		
Objectives	3	State specific objectives, including any prespecified hypotheses	Introduction Page 5		
**Methods**					
Study Design	4	Present key elements of study design early in the paper	Methods pages 6 to 7		
Setting	5	Describe the setting, locations, and relevant dates, including periods of recruitment, exposure, follow-up, and data collection	Methods page 6		

For peer review only

Participants	6	*(a) Cohort study* - Give the eligibility criteria, and the sources and methods of selection of participants. Describe methods of follow-up<br>*Case-control study* - Give the eligibility criteria, and the sources and methods of case ascertainment and control selection. Give the rationale for the choice of cases and controls<br>*Cross-sectional study* - Give the eligibility criteria, and the sources and methods of selection of participants<br><br>*(b) Cohort study* - For matched studies, give matching criteria and number of exposed and unexposed<br>*Case-control study* - For matched studies, give matching criteria and the number of controls per case	Methods page 6	RECORD 6.1: The methods of study population selection (such as codes or algorithms used to identify subjects) should be listed in detail. If this is not possible, an explanation should be provided.<br><br>RECORD 6.2: Any validation studies of the codes or algorithms used to select the population should be referenced. If validation was conducted for this study and not published elsewhere, detailed methods and results should be provided.<br><br>RECORD 6.3: If the study involved linkage of databases, consider use of a flow diagram or other graphical display to demonstrate the data linkage process, including the number of individuals with linked data at each stage.	Methods Page 6, supplemental figure 1<br><br>N/A<br><br>N/A
Variables	7	Clearly define all outcomes, exposures, predictors, potential confounders, and effect modifiers. Give diagnostic criteria, if applicable.	Methods page 6 and 7	RECORD 7.1: A complete list of codes and algorithms used to classify exposures, outcomes, confounders, and effect modifiers should be provided. If these cannot be reported, an explanation should be provided.	Methods page 6 (description of data selection, no codes)
Data sources/ measurement	8	For each variable of interest, give sources of data and details of methods of assessment (measurement).<br>Describe comparability of assessment methods if there is more than one group	Methods Page 6		
Bias	9	Describe any efforts to address			

For peer review only

		potential sources of bias			
Study size	10	Explain how the study size was arrived at	Supplemental figure 1		
Quantitative variables	11	Explain how quantitative variables were handled in the analyses. If applicable, describe which groupings were chosen, and why	Methods Page 6 and 7		
Statistical methods	12	(a) Describe all statistical methods, including those used to control for confounding<br>(b) Describe any methods used to examine subgroups and interactions<br>(c) Explain how missing data were addressed<br>(d) *Cohort study* - If applicable, explain how loss to follow-up was addressed<br>*Case-control study* - If applicable, explain how matching of cases and controls was addressed<br>*Cross-sectional study* - If applicable, describe analytical methods taking account of sampling strategy<br>(e) Describe any sensitivity analyses	Methods Page 6 and 7		
Data access and cleaning methods		..		RECORD 12.1: Authors should describe the extent to which the investigators had access to the database population used to create the study population.<br><br>RECORD 12.2: Authors should provide information on the data cleaning methods used in the study.	Acknowledgement section, page 21

For peer review only

					Methods Page 6
Linkage		..		RECORD 12.3: State whether the study included person-level, institutional-level, or other data linkage across two or more databases. The methods of linkage and methods of linkage quality evaluation should be provided.	No linkage
**Results**					
Participants	13	(a) Report the numbers of individuals at each stage of the study (*e.g.*, numbers potentially eligible, examined for eligibility, confirmed eligible, included in the study, completing follow-up, and analysed)<br>(b) Give reasons for non-participation at each stage.<br>(c) Consider use of a flow diagram	Supplemental figure 1 and Results page 9	RECORD 13.1: Describe in detail the selection of the persons included in the study (*i.e.,* study population selection) including filtering based on data quality, data availability and linkage. The selection of included persons can be described in the text and/or by means of the study flow diagram.	Methods; Results page 9 and supplemental figure 1
Descriptive data	14	(a) Give characteristics of study participants (*e.g.*, demographic, clinical, social) and information on exposures and potential confounders<br>(b) Indicate the number of participants with missing data for each variable of interest<br>(c) *Cohort study* - summarise follow-up time (*e.g.*, average and total amount)			
Outcome data	15	*Cohort study* - Report numbers of outcome events or summary measures over time<br>*Case-control study* - Report numbers in each exposure category, or summary measures of exposure	All supplemental tables		

For peer review only

		Cross-sectional study - Report numbers of outcome events or summary measures			
Main results	16	(a) Give unadjusted estimates and, if applicable, confounder-adjusted estimates and their precision (e.g., 95% confidence interval). Make clear which confounders were adjusted for and why they were included<br>(b) Report category boundaries when continuous variables were categorized<br>(c) If relevant, consider translating estimates of relative risk into absolute risk for a meaningful time period	Results Pages 9 to 17, embedded tables and Supplemental tables		
Other analyses	17	Report other analyses done—e.g., analyses of subgroups and interactions, and sensitivity analyses	Results Page 17		
**Discussion**					
Key results	18	Summarise key results with reference to study objectives	Discussion Page 18		
Limitations	19	Discuss limitations of the study, taking into account sources of potential bias or imprecision. Discuss both direction and magnitude of any potential bias	Discussion page 19	RECORD 19.1: Discuss the implications of using data that were not created or collected to answer the specific research question(s). Include discussion of misclassification bias, unmeasured confounding, missing data, and changing eligibility over time, as they pertain to the study being reported.	Discussion Page 19
Interpretation	20	Give a cautious overall interpretation of results considering objectives, limitations, multiplicity of analyses, results from similar	Discussion Pages 18-19		

For peer review only - http://bmjopen.bmj.com/site/about/guidelines.xhtml

		studies, and other relevant evidence			
Generalisability	21	Discuss the generalisability (external validity) of the study results	Discussion Page 19/20		
**Other Information**					
Funding	22	Give the source of funding and the role of the funders for the present study and, if applicable, for the original study on which the present article is based	Page 21		
Accessibility of protocol, raw data, and programming code		..	N/A	RECORD 22.1: Authors should provide information on how to access any supplemental information such as the study protocol, raw data, or programming code.	N/A

*Reference: Benchimol EI, Smeeth L, Guttmann A, Harron K, Moher D, Petersen I, Sørensen HT, von Elm E, Langan SM, the RECORD Working Committee. The REporting of studies Conducted using Observational Routinely-collected health Data (RECORD) Statement. *PLoS Medicine* 2015; https://doi.org/10.1371/journal.pmed.1001885.

*Checklist is protected under Creative Commons Attribution (CC BY) license.

For peer review only

Assessing the non-inferiority of prosthesis used in total and unicondylar knee replacements using data from the National Joint Registry of England, Wales, Northern Ireland and the Isle of Man: A benchmarking study.

Journal:	*BMJ Open*
Manuscript ID	bmjopen-2018-026736.R1
Article Type:	Research
Date Submitted by the Author:	25-Jan-2019
Complete List of Authors:	Deere, Kevin; University of Bristol, School of Social & Community Medicine<br>Whitehouse, Michael; University of Bristol, Musculoskeletal Research Unit<br>Porter, Martyn; Wrightington Hospital<br>Blom, AW; University of Bristol,<br>Sayers, Adrian; University of Bristol, Muscloskeletal Research Unit
**Primary Subject Heading**:	Surgery
Secondary Subject Heading:	Patient-centred medicine
Keywords:	knee arthroplasty, non-inferiority, National Joint Registry, benchmarking, medical devices

SCHOLARONE™
Manuscripts

Assessing the non-inferiority of prosthesis used in total and unicondylar knee replacements using data from the National Joint Registry of England, Wales, Northern Ireland and the Isle of Man: A benchmarking study.

Kevin Deere, Michael R Whitehouse, Martyn L Porter, Ashley W Blom, Adrian Sayers

Musculoskeletal Research Unit, Bristol Medical School, 1st Floor Learning & Research Building, Southmead Hospital, Bristol, BS10 5NB, UK

Kevin Deere
Senior Research Associate

Michael R Whitehouse
Reader and Consultant in Trauma and Orthopaedics

Ashley W Blom
Professor of Orthopaedic Surgery and Head of Translational Health Sciences

Adrian Sayers
Senior Research Fellow

National Institute for Health Research Bristol Biomedical Research Centre, University of Bristol

Michael R Whitehouse
Reader and Consultant in Trauma and Orthopaedics

Ashley W Blom
Professor of Orthopaedic Surgery and Head of Translational Health Sciences

Centre for Hip Surgery, Wrightington Hospital, Lancashire, WN6 9EP, UK

Martyn L Porter
Consultant Orthopaedic Surgeon

Bristol Medical School, Population Health Sciences, University of Bristol, Bristol, UK

Adrian Sayers
Senior Research Fellow

Corresponding author: Adrian Sayers

Musculoskeletal Research Unit,

1st Floor Learning & Research Building,

Southmead Hospital,

Bristol,

BS10 5NB, UK

Email: adrian.sayers@bristol.ac.uk

Tel: 0117 4147880

Word count: 4,296

For peer review only - http://bmjopen.bmj.com/site/about/guidelines.xhtml

For peer review only

ABSTRACT

Objectives To investigate the relative performance of knee replacement constructs compared to the best performing construct and illustrate the substantial variability in performance.

Design A non-inferiority study.

Setting England and Wales

Participants All primary total and unicondylar knee replacements performed and registered in the National Joint Registry (NJR) between 1st April 2003 and 31st December 2016**.**

Main outcome measures Kaplan-Meier failure function for knee replacement constructs. Failure difference between best performing construct (the benchmark) and other constructs.

Methods Using a non-inferiority analysis, the performance of knee replacement constructs by brand were compared to the best performing construct. Construct failure was estimated using the 1-Kaplan Meier method, i.e. an estimate of net failure. The difference in failure between the contemporary benchmark construct and all other constructs were tested.

Results Of the 449 different knee replacement constructs used, only 27 had ≥500 procedures at risk at 10 years post-surgery, 18 of which were classified as inferior to the benchmark by at least 20% relative risk of failure. Two of these 18 were unicondylar constructs that were inferior by at least 100% relative risk. In men, aged 55-75, 12 of 27 (44%) constructs were inferior by at least 20% to the benchmark at 7 years post-surgery. In women, aged 55-75, eight of 32 (25%) constructs were inferior at 7 years post-surgery. Very few constructs were classified as non-inferior to the contemporary benchmark.

Conclusions There are few knee replacement constructs that can be shown to be non-inferior to a contemporary benchmark. Unicondylar knee constructs have, almost universally, at least 100% worse revision outcomes compared to the best performing total knee replacement. We have a duty to inform patients, clinicians and commissioners of this variability in performance of knee constructs.

For peer review only

ARTICLE SUMMARY

Strengths and limitations of this study

- Data presented from the largest joint registry in the world
- A novel and systematic comparison of the performance of knee constructs to a contemporary benchmark knee construct
- Unambiguous presentation of data will facilitate the consenting process for patients and allows surgeons and policy makers to be more informed with respect to success and failure of different constructs options available in knee replacement
- Residual and unmeasured confounding factors are likely to be present.
- Potential for selection bias whereby certain constructs may be implanted for particular indications and in particular groups with different risks

For peer review only - http://bmjopen.bmj.com/site/about/guidelines.xhtml

INTRODUCTION

Over 90,000 knee replacements are performed annually in England and Wales and there is a bewildering choice of total and unicondylar knee replacement options available from which clinicians and patients can choose. When patients are considering a knee replacement, it is understandable that many assume that the different constructs function equally. However, all constructs are not equal as evidenced by variation in revision rates between brands and knee construct types.[1] The National Joint Registry for England, Wales, Northern Ireland and the Isle of Man (NJR) is the largest arthroplasty database in the world and publishes the unadjusted cumulative failure rates of the most commonly used constructs in knee replacement surgery. This is a useful format for measuring absolute failure but does not allow easy head-to-head comparison of constructs. Bench-marking bodies such as the Orthopaedic Device Evaluation Panel (ODEP) in the UK,[2] NOV in the Netherlands,[3] and the Australian superior clinical performance program[4] compare construct performance against externally set benchmarks, but do not perform head-to-head comparison. Although it is reassuring a certain standard has been met, this simple dichotomisation does not facilitate head-to-head comparison and the sample sizes used are arbitrarily set. Sayers et al. recently proposed a method of comparison for joint replacement constructs using a non-inferiority design against an external benchmark.[5] However, the primary limitation of this method remains the arbitrary requirement for an externally specified benchmark.

In a non-inferiority clinical trial[6] investigating failure, two treatments (comparator and reference) can be directly compared to ensure that the comparator treatment is within a clinically acceptable range (non-inferiority margin) of the performance of the reference treatment at a specified point in time.[7, 8] Standard methods for assessing non-inferiority could be applied in an orthopaedic benchmarking setting, assuming an appropriate comparator, non-inferiority margin, and time of interest can be identified. This is a method we have applied in a medical device setting, namely hip

For peer review only - http://bmjopen.bmj.com/site/about/guidelines.xhtml

replacements using NJR data, in which we assessed the non-inferiority of hip replacement constructs as compared to benchmark construct.[9]

Choosing an appropriate outcome and contemporary reference is difficult. There is no single outcome, no gold standard, or evidence from randomised trials that suggests any construct outperforms all others, therefore the choice is more heuristic. Patients would like to receive the best available care and clinicians would like to provide the best possible care, or at least care that it is non-inferior to the best. A binary, unambiguous, endpoint such as revision surgery represents one potential outcome. Therefore, the natural choice of reference is the construct with the lowest failure rate. However, in order to protect against chance the construct should be used in large enough numbers to mitigate sampling variability. The failure rate of a construct is influenced by both age and gender, therefore the choice of reference should reflect this specificity. The selection of an appropriate time and non-inferiority margin to assess construct performance is more subjective. For example, construct survivorship in the long term is less relevant to older patients with shorter life expectancy, where improved quality of life, reduction in pain or improved physical functioning maybe more relevant.

The aim of this study is to investigate the relative performance of knee replacement constructs as compared to the best performing contemporary construct, the benchmark, using a non-inferiority study design and to illustrate the substantial variability in performance of widely used constructs. This research focuses on total knee replacements (TKRs) and unicondylar knee replacements (UKRs) as these are commonly used and therefore there is sufficient data to make robust comparisons. TKRs and UKRs are examined against non-inferiority margins of 20% relative risk and 100% relative risk at 3, 5, 7 and 10 years following surgery. This is predicated on our belief that patients would at least want reassurance that the construct they are to receive is proven to not be 100% worse than the best alternatives when used in patients with the same gender and age as them.

For peer review only - http://bmjopen.bmj.com/site/about/guidelines.xhtml

METHODS

Patients and Data Sources

We identified all patients with a primary TKR or UKR registered in the NJR between 1st April 2003 and December 31st 2016. All patients were consented to be included in the NJR as part of the standard NJR process.

Procedures were excluded if the patient age or gender were missing, or the National Health Service (NHS) number was untraceable and therefore mortality unknown. Procedures where the constraint or fixation type were unknown were excluded from the analysis. Patellofemoral replacements were also removed owing to the low number in the sample.

Brands of constructs are usually subdivided by fixation, mobility of bearing and constraint. NJR Annual Report data has shown that these characteristics influence revision and thus we treated each subdivision as a separate construct.

Patient involvement

Patient representatives sit on the committee structure of the National Joint Registry. The research priorities of the National Joint Registry are identified by this committee structure and approved by the patient representatives. Patients were not involved in the setting of the research question or the outcome measures, nor were they involved in designing or implementing this work or interpretation of the results. We are unable to disseminate results of this study directly to study participants due to the anonymous nature of the data. We plan to disseminate our findings to the National Joint Registry, via their communications team, to consultations relevant to the provision of joint replacement and to the general population through the local and national press.

Statistical methods

For peer review only - http://bmjopen.bmj.com/site/about/guidelines.xhtml

Using a non-inferiority analysis, the performance of knee constructs were compared to an internally identified benchmark group. Construct failure was estimated using the 1-Kaplan Meier method i.e. an estimate of net failure, which is appropriate when the risk of revision is considered.[10]

Failure is defined using the first linked surgical revision, where revision was defined as any addition, removal or modification of an implant to a joint that had previously undergone a joint replacement. Patients were censored at death or administratively censored on 31st December 2016. The difference in stratum specific failure probabilities compared to the benchmark were calculated at 3, 5, 7 and 10 years for all constructs, stratified by gender, and stratified by gender and age group (<55 years, 55 to 75 years, >75 years).

The difference and 95% confidence interval (CI) of the difference between the comparator construct and the benchmark construct was estimated at the specified time points. We used a 95% CI, as is the convention in the majority if medical research. The standard error of the difference was constructed using a pooled estimate of the Greenwood Standard error,[11]

$$\widehat{SE(Diff)} = \sqrt{GSE_x^2 + GSE_{ref}^2} ,$$

and a z-test comparing the difference between the benchmark and test construct was then constructed using,

$$Z = \left(\left(\widehat{F_{test}} - \widehat{F_{ref}}\right) + \delta\right) / \widehat{SE(Diff)}.$$

The stratum specific contemporaneous benchmark construct was selected as the knee construct (TKR or UKR) with the lowest failure rate with at least 1,000 patients at risk at the time point of interest. The choice of 1,000 procedures of the same construct was based on simulation work by Sayers et al. which demonstrated that 1,000 procedures at risk will give rise to a confidence interval width of approximately 3% (±1.5%).[5] We believe this is the minimal level of accuracy to be considered a suitable reference standard.

For peer review only - http://bmjopen.bmj.com/site/about/guidelines.xhtml

Two non-inferiority margins were chosen to illustrate the sensitivity of the choice. The first margin was conservatively set at a 20% increase in relative risk of failure compared to the benchmark, in line with clinical trials using this methodology, albeit towards the upper end.[12] The second was a 100% increase in relative risk, i.e. a doubling in cumulative probability of failure, as this is an easily interpretable outcome.

Results are graphically reported for all comparator constructs with at least 500 patients still at risk at the beginning of the time point of interest. Results are also reported in a tabular format for all comparator constructs with at least 250 patients at risk at the beginning of the time point of interest (See supplementary tables).

Constructs were either classified as non-inferior, inconclusive, or inferior. If the upper confidence interval is less than or equal to the 20% non-inferiority margin, the construct was non-inferior. If the lower confidence interval of the difference was greater than the non-inferiority margin at either 20% or 100% the construct was classed as inferior at 20% or 100% respectively. If the lower confidence limit is less than the non-inferiority margin, and the upper confidence greater than non-inferiority margin the evidence against the construct was described as inconclusive (See figure 1 for graphical representation of the classification). All analyses were carried out using Stata 15.1 (College Station, Texas, USA).

Sensitivity analysis

We repeated all analyses using the best performing knee construct at 10 years with at least 1,000 still at risk in the stratum of interest as the benchmark at the 3, 5, and 7-year time points.

For peer review only - http://bmjopen.bmj.com/site/about/guidelines.xhtml

RESULTS

There were 975,739 primary knee replacements included in the NJR between 1st April 2003 and 31st December 2016, utilising 649 different combinations of brand, fixation, constraint and bearing type Following the application of the exclusion criteria (See supplementary fig 1), 947,686 procedures were included in the final analysis (863,551 (91.1%) TKRs), utilising 449 different combinations of brand, fixation, constraint and bearing type (405 TKRs and 44 UKRs).

A detailed description of non-inferiority across all procedures is provided. Due to the large number of clinically relevant subdivisions and sensitivity analyses, results will be described more broadly. Each stratification of age group, gender and time point of interest are provided in supplemental material.

Constructs are described by brand, fixation, the degree of constraint for TKR and whether the bearing was fixed or mobile. Constraint types were either unconstrained (cruciate retaining) or posterior-stabilised. The vast majority of benchmark constructs were cemented. In each subdivision of our analyses the construct that met our benchmark criteria was a TKR that was unconstrained with a fixed bearing. However, not all total knees that were unconstrained with fixed bearings performed well as there were 15 separate brands of this type that were found to be inferior to the benchmark by at least 20% relative risk in at least one subdivision analysis.

For peer review only

Non-inferiority - All procedures

The benchmark construct at 3 years was identified as the NexGen cemented, unconstrained TKR with a fixed bearing. There were 34,558 procedures remaining at risk at 3 years for this construct and the failure rate was 1.10% (95%CI 1.01 – 1.20). There were 73 constructs with ≥500 procedures at risk. Thirty-nine constructs were classified as inferior to the benchmark by at least 20% relative risk of failure. Nine of the 73 were shown to be inferior by at least 100% relative risk (supplementary

For peer review only - http://bmjopen.bmj.com/site/about/guidelines.xhtml

figure 2). One prosthesis, the NexGen TKR with a monobloc polyethylene tibia, was non-inferior. The remaining 32 constructs were classified as non-inferiority not shown.

The benchmark construct at 5 years was identified as the Profix uncemented unconstrained TKR with a fixed bearing. There were 1,910 procedures remaining at risk and the failure rate was 1.54% (95%CI 1.10 – 2.15). There were 65 constructs with ≥500 procedures at risk. Thirty-six were classified as inferior to the benchmark by at least 20% relative risk of failure. Twelve of the 36 were shown to be inferior by at least 100% relative risk (figure 2). All of the unicondylar constructs with ≥500 procedures at risk (n=8) were inferior by at least 100% relative risk. No construct could be described as non-inferior.

The benchmark construct at 7 years was identified, again, as the Profix uncemented unconstrained TKR with a fixed bearing. There were 1,501 procedures remaining at risk and the failure rate was 1.77% (95%CI 1.29 – 2.43). There were 57 constructs with ≥500 procedures at risk. Thirty constructs were classified as inferior to the benchmark by at least 20% relative risk of failure. Eight of the 30 were shown to be inferior by at least 100% relative risk (supplementary figure 3). All of the unicondylar constructs with ≥500 procedures at risk (n=5) were inferior by at least 100% relative risk. No construct could be described as non-inferior.

The benchmark construct at 10 years was identified as the TKR PFC Sigma Bicondylar Knee cemented unconstrained TKR with a fixed bearing. There were 19,284 procedures remaining at risk and the failure rate was 2.37% (95%CI 2.27 – 2.47). There were 27 constructs with ≥500 procedures at risk. Eighteen constructs were classified as inferior to the benchmark by at least 20% relative risk of failure. Two of the 18 were shown to be inferior by at least 100% relative risk (figure 3). There were only 2 unicondylar knee replacements with ≥500 procedures at risk at 10 years, both of which were inferior to the benchmark by at least 100% relative risk. Two constructs were identified as non-inferior, the NexGen cemented unconstrained TKR with fixed bearing and the TKR PFC Sigma Bicondylar Knee hybrid uncemented with a fixed bearing.

For peer review only - http://bmjopen.bmj.com/site/about/guidelines.xhtml

Estimates for the difference in failure between the benchmark and comparator constructs with ≥250 procedure at risk at the time of interest are shown in supplementary tables 1-4.

Non-inferiority - Gender specific

Gender specific non-inferiority analyses were also performed at 3, 5, 7, and 10 years after the primary operation.

At 3 years there were no constructs that demonstrated non-inferiority in comparison to the benchmark prosthesis. Most striking is the general similarity in constructs used and their performance regardless of gender. There were 56 different constructs with at least 500 procedures still at risk in females versus 50 in males. There were 25 constructs with at least a 20% increase in relative risk in females versus 18 in males although the increased number of inferior constructs demonstrated in females is likely owing to the slightly better performing benchmark group than in males. Three constructs were inferior by at least 100% in males, each a UKR, and eight constructs were inferior by at least 100% in females (6 UKRs and 2 TKRs).

At 5 years, in females, there were 49 constructs with ≥500 procedures at risk. Twenty-nine of these constructs were classified as inferior to the benchmark by at least 20% relative risk of failure. Six of the 29 were shown to be inferior by at least 100% relative risk, four of which were unicondylar replacements. Similarly, in males there were 43 constructs used with 19 inferior by at least a 20% increase in relative risk. Four of the 19 were inferior by at least 100% relative risk, all of which were unicondylar replacements. Results for males and females at 5 years with ≥250 procedures at risk can be seen in tables 1 and 2 respectively.

For peer review only - http://bmjopen.bmj.com/site/about/guidelines.xhtml

Table 1: Difference in cumulative percentage revision of knee constructs compared to a contemporary benchmark at 5 years post primary in men for knee replacements with ≥250 procedures remaining at risk

Knee brand, bearing and constraint	Number at risk	Cumulative failure (%)	Difference in failure (%)	95% CI	Equivalence status	p-value
[TKR] NexGen Cement, unconstrained fixed	8,639	1.76	[REFERENCE]			
[TKR] AGC Cement, unconstrained fixed	16,871	2.28	0.52	[0.24, 0.80]	Non-inferiority not shown	<0.001
[TKR] AGC Hybrid, unconstrained fixed	369	2.59	0.83	[-0.70, 2.36]	Non-inferiority not shown	0.143
[TKR] AGC Uncemented, unconstrained fixed	468	4.87	3.11	[1.33, 4.88]	Inferior by ≥20%	<0.001
[TKR] Advance MP Cement, unconstrained fixed	1,725	3.18	1.42	[0.74, 2.11]	Inferior by ≥20%	<0.001
[TKR] Advance PS Cement, posterior-stabilised fixed	266	3.48	1.72	[-0.11, 3.55]	Non-inferiority not shown	0.033
[TKR] Columbus Cement, unconstrained fixed	1,224	2.49	0.73	[0.08, 1.38]	Non-inferiority not shown	0.013
[TKR] E-Motion Bicondylar Knee Uncemented, unconstrained mobile	519	2.49	0.73	[-0.41, 1.88]	Non-inferiority not shown	0.105
[TKR] FS Cement, unconstrained fixed	305	1.76	0.00	[-1.41, 1.40]	Non-inferiority not shown	0.497
[TKR] Genesis 2 Cement, posterior-stabilised fixed	1,723	2.97	1.21	[0.61, 1.82]	Inferior by ≥20%	<0.001
[TKR] Genesis 2 Cement, unconstrained fixed	6,545	2.17	0.41	[0.08, 0.74]	Non-inferiority not shown	0.008
[TKR] Genesis 2 Cement, unconstrained mobile	310	3.52	1.76	[-0.13, 3.65]	Non-inferiority not shown	0.034
[TKR] Genesis 2 Oxinium Cement, posterior-stabilised fixed	411	5.27	3.51	[1.89, 5.13]	Inferior by ≥100%	<0.001
[TKR] Genesis 2 Oxinium Cement, unconstrained fixed	1,175	3.42	1.65	[0.76, 2.55]	Inferior by ≥20%	<0.001
[TKR] Insall-Burstein 2 Cement, posterior-stabilised fixed	892	2.88	1.12	[0.06, 2.17]	Non-inferiority not shown	0.019
[TKR] Kinemax Cement, unconstrained fixed	3,927	3.08	1.32	[0.76, 1.87]	Inferior by ≥20%	<0.001
[TKR] LCS Complete Cement, unconstrained mobile	2,774	2.88	1.12	[0.54, 1.70]	Inferior by ≥20%	<0.001
[TKR] LCS Complete Uncemented, unconstrained mobile	3,472	2.36	0.60	[0.14, 1.06]	Non-inferiority not shown	0.005
[TKR] LCS Uncemented, unconstrained mobile	485	2.82	1.06	[-0.36, 2.48]	Non-inferiority not shown	0.072
[TKR] MRK Cement, unconstrained fixed	1,681	2.07	0.31	[-0.26, 0.87]	Non-inferiority not shown	0.143
[TKR] Maxim Cement, unconstrained fixed	481	1.86	0.10	[-1.06, 1.25]	Non-inferiority not shown	0.436
[TKR] NRG Cement, posterior-stabilised fixed	1,291	2.62	0.86	[0.10, 1.62]	Non-inferiority not shown	0.014
[TKR] NRG Cement, unconstrained fixed	1,649	3.14	1.38	[0.67, 2.09]	Inferior by ≥20%	<0.001
[TKR] Natural Knee II Cement, unconstrained fixed	767	2.02	0.26	[-0.65, 1.16]	Non-inferiority not shown	0.288
[TKR] NexGen Cement, posterior-stabilised mobile	267	3.93	2.17	[0.20, 4.14]	Non-inferiority not shown	0.016
[TKR] NexGen Cement, posterior-stabilised fixed	11,354	2.89	1.13	[0.81, 1.45]	Inferior by ≥20%	<0.001
[TKR] NexGen Hybrid, unconstrained fixed	379	1.47	-0.29	[-1.39, 0.81]	Non-inferiority not shown	0.303
[TKR] NexGen Uncemented, posterior-stabilised fixed	568	1.81	0.05	[-0.87, 0.97]	Non-inferiority not shown	0.456
[TKR] NexGen Uncemented, unconstrained fixed	1,639	3.13	1.36	[0.59, 2.14]	Inferior by ≥20%	<0.001
[TKR] Optetrak Cement, posterior-stabilised fixed	533	3.51	1.75	[0.29, 3.20]	Non-inferiority not shown	0.009
[TKR] PFC Sigma Bicondylar Knee Cement, posterior-stabilised mobile	1,976	2.65	0.89	[0.27, 1.51]	Non-inferiority not shown	0.002
[TKR] PFC Sigma Bicondylar Knee Cement, posterior-stabilised fixed	15,828	2.16	0.40	[0.13, 0.67]	Non-inferiority not shown	0.002
[TKR] PFC Sigma Bicondylar Knee Cement, unconstrained fixed	36,856	1.83	0.07	[-0.16, 0.30]	Non-inferior	0.279
[TKR] PFC Sigma Bicondylar Knee Cement, unconstrained mobile	2,565	2.75	0.98	[0.40, 1.57]	Inferior by ≥20%	0.001
[TKR] PFC Sigma Bicondylar Knee Hybrid, unconstrained fixed	619	1.86	0.10	[-0.93, 1.12]	Non-inferiority not shown	0.428
[TKR] PFC Sigma Bicondylar Knee monobloc polyethylene tibia	898	1.69	-0.07	[-0.61, 0.46]	Non-inferiority not shown	0.394

For peer review only - http://bmjopen.bmj.com/site/about/guidelines.xhtml

For peer review only

[TKR] Profix Cement, unconstrained fixed	360	3.97	2.21	[0.29, 4.12]	Non-inferiority not shown	0.012
[TKR] Profix Uncemented, unconstrained fixed	856	1.21	-0.55	[-1.26, 0.16]	Non-inferior	0.064
[TKR] Rotaglide + Cement, unconstrained mobile	610	4.40	2.64	[1.11, 4.17]	Inferior by ≥20%	<0.001
[TKR] Rotaglide Cement, unconstrained mobile	291	4.30	2.54	[0.62, 4.45]	Inferior by ≥20%	0.005
[TKR] Scorpio Cement, posterior-stabilised mobile	517	2.39	0.63	[-0.62, 1.89]	Non-inferiority not shown	0.162
[TKR] Scorpio Cement, posterior-stabilised fixed	2,090	2.91	1.14	[0.43, 1.86]	Inferior by ≥20%	0.001
[TKR] Scorpio Cement, unconstrained fixed	3,667	3.06	1.30	[0.75, 1.86]	Inferior by ≥20%	<0.001
[TKR] Scorpio Cement, unconstrained mobile	436	3.94	2.18	[0.43, 3.93]	Inferior by ≥20%	0.007
[TKR] Scorpio Hybrid, unconstrained fixed	374	3.33	1.57	[-0.16, 3.30]	Non-inferiority not shown	0.038
[TKR] Scorpio Uncemented, unconstrained fixed	1,440	2.50	0.74	[-0.04, 1.51]	Non-inferiority not shown	0.031
[TKR] TC Plus Cement, unconstrained fixed	3,133	3.00	1.23	[0.64, 1.83]	Inferior by ≥20%	<0.001
[TKR] TC Plus Cement, unconstrained mobile	1,758	2.61	0.84	[0.13, 1.56]	Non-inferiority not shown	0.010
[TKR] TC Plus Uncemented, unconstrained mobile	434	1.80	0.04	[-1.00, 1.07]	Non-inferiority not shown	0.473
[TKR] Triathlon Cement, posterior-stabilised fixed	1,851	2.53	0.77	[0.23, 1.31]	Non-inferiority not shown	0.003
[TKR] Triathlon Cement, unconstrained fixed	5,995	1.94	0.18	[-0.13, 0.48]	Non-inferiority not shown	0.126
[TKR] Vanguard Cement, posterior-stabilised fixed	471	2.57	0.81	[-0.05, 1.67]	Non-inferiority not shown	0.033
[TKR] Vanguard Cement, unconstrained fixed	3,489	2.10	0.34	[-0.01, 0.70]	Non-inferiority not shown	0.028
[UNI] AMC/Uniglide Unicondylar, fixed	348	3.97	2.21	[0.51, 3.90]	Inferior by ≥20%	0.005
[UNI] AMC/Uniglide Unicondylar, mobile	519	8.90	7.14	[5.06, 9.22]	Inferior by ≥100%	<0.001
[UNI] MG Uni Unicondylar, fixed	1,130	5.36	3.60	[2.33, 4.86]	Inferior by ≥100%	<0.001
[UNI] Oxford Partial Knee Unicondylar, mobile	14,581	5.72	3.96	[3.59, 4.33]	Inferior by ≥100%	<0.001
[UNI] Physica ZUK Unicondylar, fixed	1,280	3.88	2.12	[1.35, 2.89]	Inferior by ≥20%	<0.001
[UNI] Preservation Unicondylar, fixed	566	8.55	6.79	[4.61, 8.96]	Inferior by ≥100%	<0.001
[UNI] Sigma HP Unicondylar, fixed	948	4.36	2.59	[1.75, 3.44]	Inferior by ≥20%	<0.001
[UNI] Sled Unicondylar, fixed	261	11.47	9.71	[6.30, 13.12]	Inferior by ≥100%	<0.001

Note: TKR = total knee replacement, UNI = Unicondylar knee replacement

For peer review only

Table 2: Difference in cumulative percentage revision of knee constructs compared to a contemporary benchmark at 5 years post primary in women for knee replacements with ≥250 procedures remaining at risk

Knee brand, bearing and constraint	Number at risk	Cumulative failure (%)	Difference in failure (%)	95% CI	Equivalence status	p-value
[TKR] MRK Cement, unconstrained fixed	2,570	1.35	[REFERENCE]			
[TKR] AGC Cement, posterior-stabilised fixed	264	2.65	1.31	[-0.54, 3.16]	Non-inferiority not shown	0.083
[TKR] AGC Cement, unconstrained fixed	23,829	1.85	0.50	[0.13, 0.88]	Non-inferiority not shown	0.004
[TKR] AGC Hybrid, unconstrained fixed	431	3.11	1.76	[0.17, 3.35]	Non-inferiority not shown	0.015
[TKR] AGC Uncemented, unconstrained fixed	502	5.34	4.00	[2.10, 5.89]	Inferior by ≥100%	<0.001
[TKR] Advance MP Cement, unconstrained fixed	2,264	2.46	1.11	[0.48, 1.75]	Inferior by ≥20%	<0.001
[TKR] Advance MP Stature Cement, unconstrained fixed	378	2.93	1.58	[0.31, 2.86]	Inferior by ≥20%	0.007
[TKR] Advance PS Cement, posterior-stabilised fixed	354	2.97	1.62	[0.09, 3.15]	Non-inferiority not shown	0.019
[TKR] Columbus Cement, unconstrained fixed	1,595	2.53	1.19	[0.54, 1.83]	Inferior by ≥20%	<0.001
[TKR] E-Motion Bicondylar Knee Uncemented, unconstrained mobile	544	3.22	1.87	[0.57, 3.17]	Inferior by ≥20%	0.002
[TKR] FS Cement, unconstrained fixed	397	2.34	0.99	[-0.48, 2.47]	Non-inferiority not shown	0.093
[TKR] Genesis 2 Cement, posterior-stabilised fixed	2,848	2.15	0.80	[0.29, 1.32]	Inferior by ≥20%	0.001
[TKR] Genesis 2 Cement, unconstrained fixed	9,021	1.44	0.10	[-0.29, 0.49]	Non-inferiority not shown	0.311
[TKR] Genesis 2 Cement, unconstrained mobile	464	1.48	0.14	[-0.94, 1.22]	Non-inferiority not shown	0.401
[TKR] Genesis 2 Oxinium Cement, posterior-stabilised fixed	620	3.49	2.14	[1.00, 3.29]	Inferior by ≥20%	<0.001
[TKR] Genesis 2 Oxinium Cement, unconstrained fixed	1,485	2.75	1.41	[0.63, 2.19]	Inferior by ≥20%	<0.001
[TKR] Insall-Burstein 2 Cement, posterior-stabilised fixed	1,177	2.63	1.28	[0.33, 2.23]	Inferior by ≥20%	0.004
[TKR] Journey Oxinium Cement, posterior-stabilised fixed	283	7.30	5.96	[3.45, 8.47]	Inferior by ≥100%	<0.001
[TKR] Kinemax Cement, unconstrained fixed	5,369	2.41	1.07	[0.54, 1.59]	Inferior by ≥20%	<0.001
[TKR] LCS Cement, unconstrained mobile	340	1.85	0.51	[-0.90, 1.91]	Non-inferiority not shown	0.239
[TKR] LCS Complete Cement, unconstrained mobile	3,759	2.62	1.28	[0.72, 1.84]	Inferior by ≥20%	<0.001
[TKR] LCS Complete Uncemented, unconstrained mobile	4,205	2.61	1.26	[0.73, 1.80]	Inferior by ≥20%	<0.001
[TKR] LCS Uncemented, unconstrained mobile	675	2.16	0.82	[-0.29, 1.92]	Non-inferiority not shown	0.073
[TKR] Maxim Cement, posterior-stabilised fixed	282	3.24	1.90	[-0.02, 3.82]	Non-inferiority not shown	0.026
[TKR] Maxim Cement, unconstrained fixed	629	2.34	1.00	[-0.16, 2.15]	Non-inferiority not shown	0.045
[TKR] NRG Cement, posterior-stabilised fixed	1,784	2.24	0.90	[0.22, 1.58]	Non-inferiority not shown	0.005
[TKR] NRG Cement, unconstrained fixed	2,350	1.79	0.44	[-0.11, 1.00]	Non-inferiority not shown	0.059
[TKR] Natural Knee II Cement, unconstrained fixed	1,183	2.34	1.00	[0.15, 1.84]	Non-inferiority not shown	0.010
[TKR] NexGen Cement, posterior-stabilised mobile	414	3.39	2.04	[0.49, 3.60]	Inferior by ≥20%	0.005
[TKR] NexGen Cement, posterior-stabilised fixed	16,989	2.30	0.95	[0.56, 1.34]	Inferior by ≥20%	<0.001
[TKR] NexGen Cement, unconstrained fixed	12,603	1.47	0.13	[-0.25, 0.51]	Non-inferiority not shown	0.253
[TKR] NexGen Hybrid, unconstrained fixed	454	1.72	0.37	[-0.79, 1.54]	Non-inferiority not shown	0.266
[TKR] NexGen Uncemented, posterior-stabilised fixed	514	2.66	1.31	[0.10, 2.52]	Non-inferiority not shown	0.017
[TKR] NexGen Uncemented, unconstrained fixed	1,421	3.25	1.90	[0.98, 2.83]	Inferior by ≥20%	<0.001
[TKR] Optetrak Cement, posterior-stabilised fixed	746	5.29	3.94	[2.45, 5.44]	Inferior by ≥100%	<0.001

For peer review only - http://bmjopen.bmj.com/site/about/guidelines.xhtml

[TKR] PFC Sigma Bicondylar Knee Cement, posterior-stabilised mobile	2,280	2.92	1.57	[0.90, 2.24]	Inferior by ≥20%	<0.001
[TKR] PFC Sigma Bicondylar Knee Cement, posterior-stabilised fixed	23,690	2.03	0.69	[0.31, 1.06]	Inferior by ≥20%	<0.001
[TKR] PFC Sigma Bicondylar Knee Cement, unconstrained fixed	51,762	1.61	0.27	[-0.09, 0.63]	Non-inferiority not shown	0.071
[TKR] PFC Sigma Bicondylar Knee Cement, unconstrained mobile	2,852	2.72	1.37	[0.73, 2.01]	Inferior by ≥20%	<0.001
[TKR] PFC Sigma Bicondylar Knee Hybrid, unconstrained fixed	771	1.75	0.40	[-0.54, 1.34]	Non-inferiority not shown	0.202
[TKR] PFC Sigma Bicondylar Knee Uncemented, unconstrained mobile	254	1.60	0.25	[-1.07, 1.57]	Non-inferiority not shown	0.355
[TKR] PFC Sigma Bicondylar Knee monobloc polyethylene tibia	1,486	1.61	0.27	[-0.27, 0.80]	Non-inferiority not shown	0.163
[TKR] Profix Cement, unconstrained fixed	454	2.58	1.23	[-0.19, 2.66]	Non-inferiority not shown	0.045
[TKR] Profix Oxinium monobloc polyethylene tibia	317	3.58	2.24	[0.22, 4.26]	Non-inferiority not shown	0.015
[TKR] Profix Uncemented, unconstrained fixed	1,055	1.81	0.47	[-0.36, 1.29]	Non-inferiority not shown	0.134
[TKR] Profix monobloc polyethylene tibia	371	1.47	0.13	[-1.09, 1.34]	Non-inferiority not shown	0.420
[TKR] Rotaglide + Cement, unconstrained mobile	845	3.08	1.74	[0.58, 2.89]	Inferior by ≥20%	0.002
[TKR] Rotaglide Cement, unconstrained mobile	487	2.65	1.31	[0.04, 2.58]	Non-inferiority not shown	0.022
[TKR] Scorpio Cement, posterior-stabilised mobile	662	2.03	0.68	[-0.39, 1.76]	Non-inferiority not shown	0.106
[TKR] Scorpio Cement, posterior-stabilised fixed	3,175	2.01	0.67	[0.09, 1.25]	Non-inferiority not shown	0.012
[TKR] Scorpio Cement, unconstrained fixed	5,380	2.29	0.94	[0.43, 1.45]	Inferior by ≥20%	<0.001
[TKR] Scorpio Cement, unconstrained mobile	601	3.39	2.05	[0.61, 3.49]	Inferior by ≥20%	0.003
[TKR] Scorpio Hybrid, unconstrained fixed	565	1.95	0.60	[-0.54, 1.75]	Non-inferiority not shown	0.152
[TKR] Scorpio Uncemented, unconstrained fixed	1,702	2.48	1.14	[0.36, 1.91]	Inferior by ≥20%	0.002
[TKR] TC Plus Cement, unconstrained fixed	3,820	2.19	0.84	[0.28, 1.40]	Inferior by ≥20%	0.002
[TKR] TC Plus Cement, unconstrained mobile	2,242	1.52	0.18	[-0.41, 0.76]	Non-inferiority not shown	0.279
[TKR] TC Plus Uncemented, unconstrained mobile	712	2.40	1.06	[0.02, 2.09]	Non-inferiority not shown	0.023
[TKR] Triathlon Cement, posterior-stabilised fixed	2,754	2.52	1.18	[0.63, 1.72]	Inferior by ≥20%	<0.001
[TKR] Triathlon Cement, unconstrained fixed	8,378	2.07	0.72	[0.32, 1.12]	Inferior by ≥20%	<0.001
[TKR] Triathlon Uncemented, unconstrained fixed	257	3.51	2.17	[0.50, 3.84]	Inferior by ≥20%	0.005
[TKR] Vanguard Cement, posterior-stabilised fixed	665	2.87	1.53	[0.71, 2.35]	Inferior by ≥20%	<0.001
[TKR] Vanguard Cement, unconstrained fixed	4,840	1.96	0.61	[0.19, 1.04]	Non-inferiority not shown	0.002
[UNI] AMC/Uniglide Unicondylar, fixed	409	4.80	3.45	[1.70, 5.21]	Inferior by ≥100%	<0.001
[UNI] AMC/Uniglide Unicondylar, mobile	479	11.54	10.19	[7.69, 12.69]	Inferior by ≥100%	<0.001
[UNI] MG Uni Unicondylar, fixed	953	6.66	5.32	[3.77, 6.87]	Inferior by ≥100%	<0.001
[UNI] Oxford Partial Knee Unicondylar, mobile	13,555	6.58	5.23	[4.74, 5.72]	Inferior by ≥100%	<0.001
[UNI] Physica ZUK Unicondylar, fixed	1,113	4.02	2.67	[1.80, 3.54]	Inferior by ≥100%	<0.001
[UNI] Preservation Unicondylar, fixed	480	12.12	10.78	[8.04, 13.52]	Inferior by ≥100%	<0.001
[UNI] Sigma HP Unicondylar, fixed	688	5.89	4.55	[3.35, 5.75]	Inferior by ≥100%	<0.001
[UNI] Sled Unicondylar, fixed	325	8.48	7.13	[4.41, 9.85]	Inferior by ≥100%	<0.001

Note: TKR = total knee replacement, UNI = Unicondylar knee replacement

For peer review only

At 7 years the results were again similar between male and females. There were 40 different constructs used in females and 36 in males. In females there were 19 constructs that were inferior by at least 20% relative risk, 2 of which were inferior by at least 100%. These were both UKRs. In males 18 of the 36 constructs with ≥500 procedures still at risk were deemed to be inferior to the benchmark by at least 20% relative risk. Three of these 18 were inferior by at least 100% relative risk, all of which were UKRs. Two constructs were demonstrated to be non-inferior to the benchmark.

At 10 years, in both males and females there was only one brand of UKR that had at least 500 procedures still at risk. In both instances this was inferior by at least 100% relative risk and was the only construct to be classified as such. There were no brands found to be non-inferior to the benchmark in either males or females. In females there were 8 of 15 constructs that were inferior to the benchmark by at least 20% relative risk. In males 8 out of 13 constructs that were inferior to the benchmark by at least 20% relative risk. Data for gender specific stratification can be viewed in supplementary figures 4, 5, 6 and 7 for males at 3, 5, 7 and 10 years respectively and supplementary figures 8, 9, 10 and 11 for females at 3, 5, 7 and 10 years respectively.

Non-inferiority- Gender and Age specific

Sub-dividing procedures by age and gender highlights the paucity of information available for male or female patients <55 years compared to those ≥55 years. There is little data at 7 years and no suitable benchmark could be found for females at 10 years in the <55 age group. There is a similar mix of construct types in each of the age groups in both males and females with cemented unconstrained total knee replacements with a fixed bearing the most popular type. Data for males under 55 years of age at 3, 5 and 7 years can be viewed in supplementary figures 12, 13 and 14 respectively. Data for females under 55 years of age at 3, 5 and 7 years can be viewed in supplementary figures 15, 16 and 17 respectively.

For peer review only - http://bmjopen.bmj.com/site/about/guidelines.xhtml

At 7 years in females aged 55-75 there were 32 different constructs that met the threshold of 500 cases for analysis with 8 being classified as inferior by at least 20% relative risk (Figure 4). Two of these were inferior by at least 100% relative risk and both were UKRs. No constructs were demonstrated to be non-inferior to the benchmark. In males there were 27 constructs meeting the threshold with 12 being classified as inferior by at least 20% relative risk (Figure 5). A single prosthesis, 1 of the 2 UKRs with at least 500 procedures still at risk, was classified as being inferior by at least 100% relative risk. One construct was found to be non-inferior to the benchmark. Data for males between 55-75 years of age at 3, 5 and 10 years post primary can be viewed in supplementary figures 18, 19 and 20 respectively. Data for females between 55-75 years of age at 3, 5 and 10 years post primary can be viewed in supplementary figures 21,22 and 23 respectively.

In the >75 years age group we found only 1 inferior construct in both males and females at 7 years. Non-inferiority was not shown for any other prostheses, but very few met the threshold of 500 cases needed for analysis. At 10 years only 3 constructs with ≥500 procedures still at risk were present in males and 5 in females. Non-inferiority was not demonstrated in any of these. At this time point and age group subdivision there were no UKRs with enough procedures still at risk to be included in the analysis. Data for males >75 years of age at 3, 5, 7 and 10 years post primary can be viewed in supplementary figure 24, 25, 26 and 27 respectively. Data for females >75 years of age at 3, 5, 7 and 10 years post primary can be viewed in supplementary figure 28, 29, 30 and 31 respectively.

Data for each level of stratification for comparator constructs with at least 250 patients at risk at the beginning of the time point of interest can be viewed in the following supplemental tables. Data for all males at 3, 7 and 10 years can be seen in supplemental tables 5, 6 and 7 respectively and for all females at 3, 7 and 10 years in supplemental tables 8, 9 and 10. Data for males <55 years of age at 3, 5 and 7 years can be seen in supplemental tables 11, 12 and 13 respectively and females <55 at 3, 5 and 7 years can be seen in supplemental tables 14, 15 and 16 respectively. Data for males aged between 55 and 75 at 3, 5, 7 and 10 years can be seen in supplemental tables 17, 18, 19 and 20

For peer review only - http://bmjopen.bmj.com/site/about/guidelines.xhtml

respectively, and for females aged between 55 and 75 years at 3, 5, 7 and 10 years data can be seen in supplemental tables 21, 22, 23 and 24 respectively. Data for males aged >75 years at 3, 5, 7 and 10 years post primary can be seen in supplemental tables 25, 26, 27 and 28 respectively, and for females >75 years at 3, 5, 7 and 10 years post primary data can be seen in supplemental tables 29, 30, 31 and 32 respectively.

Sensitivity Analysis

Using the benchmark construct at 10 years as the benchmark at 3, 5, and 7 years, illustrates the temporal improvements in failure and the selective trajectory tracking of new constructs. The benchmark construct in all procedures at 10 years is the PFC Sigma Bicondylar Knee cemented unconstrained TKR with a fixed bearing which had a failure rate of 2.37% (95%CI 2.27 – 2.47). At 3, 5 and 7 years, the contemporary benchmark has a 0.15%, a 0.17% and 0.23% lower point-estimate failure rate than the historical benchmark respectively. The performance of the constructs appears to track in a consistent manner from each time point to the next.

For peer review only

DISCUSSION

We have demonstrated in 947,686 primary TKRs and UKRs the relative performance of knee constructs in comparison to an internally selected relevant contemporary benchmark. There are two striking findings from the data analysed here. First, UKR brands almost universally have 100% worse revision outcomes in all ages, gender and time points of interest compared to the benchmark standard brand of knee replacement. Second, very few brands of knee replacement can be proven not to be at least 20% worse than the benchmark brands despite 449 brand constructs having been constructed in England and Wales since 2003. Between one and two thirds of the knee constructs for which sufficient data was available at each time point were at least 20% inferior to the contemporary benchmark. The vast majority of constructs are implanted in too few cases to allow meaningful analysis. Many of those implanted in sufficient number have here been demonstrated to be inferior in terms of construct survivorship, whilst very few TKR's have been demonstrated to be non-inferior to a contemporary benchmark.

It is known that TKRs as a class have lower revision rates than UKRs which poses the question, should these two classes be directly compared? Since every patient who received a UKR could have received a TKR instead, this comparison is justified. There is no evidence to suggest that the subsequent observed revision rates would be any different if those receiving a UKR had received a TKR instead. Furthermore, ODEP (currently) do not provide a rating for UKRs so this method provides extra transparency and previously unavailable information for patients undergoing knee replacement.

Comparison with other studies and implications in light of existing evidence

No other published study has performed a direct head-to-head comparison of all available knee replacement constructs. The closest available data is from national registry annual reports such as the NJR[1] which reports absolute failure of the commonest constructs by age and gender. This shows that low failure rates are achieved by a number of constructs, but this does not facilitate easy

For peer review only - http://bmjopen.bmj.com/site/about/guidelines.xhtml

direct comparison between them. Using the data presented here alongside the annual report data will greatly enhance the information available to surgeons, commissioners and to inform patient choice.

Consideration of the difference in outcome of UKR compared to TKR is complex. Using propensity score matching of registry data Liddle et al. showed that UKRs have higher revision rates than TKRs, but lower risks of complications.[13] Hunt et al. and Liddle et al. showed UKRs to be associated with lower early post-operative mortality.[13, 14] Kleeblad et al. performed a systematic review and meta-analysis of 49 cohort studies and found no difference in function as measured by Hospital for Special Surgery Score, Knee Society Score, Oxford Knee Score, Visual Analog Pain Scale and Western Ontario and McMaster Universities Osteoarthritis Index Score, but did show higher revision rates with UKRs.[15] Liddle et al. also showed no clinically important difference in Oxford Knee Scores (1 point difference favouring UKR), but UKR patients were more likely to be highly satisfied 6 months after surgery.[16] A recent study by the same group has shown that UKR is less costly than TKR particularly in older patients, who are less likely to require revision, and when performed by higher volume surgeons.[17] Our data adds to this complex picture by clearly demonstrating the increased risk of revision associated with almost all brands of UKRs at all time points in both genders and all age groups when compared with the best performing TKRs.

The major weakness of all registry studies is selection bias whereby certain constructs may be implanted for particular indications and in particular groups with different risks. We have mitigated against this by analysing data by age and gender, the two variables with the greatest association with revision rates. Furthermore, revision thresholds may be lower in certain groups or for certain modes of failure. This study has looked at a single, but extremely important, outcome measure, revision. Other outcomes of interest such as mortality and PROMs need to be considered when making choices about treatment. With over 900,000 subjects this is the largest study of knee replacement published to date and comes from the largest implant registry in the world. Data entry

For peer review only - http://bmjopen.bmj.com/site/about/guidelines.xhtml

is mandated and data capture is extremely high (over 95%),[18] thus the findings are highly likely to be generalisable. The methods used are novel and for the first time allow a meaningfully direct comparison between all available constructs.

Conclusions, policy and future research implications

The use of product benchmarking has the potential to be highly informative for patients, change the practice of surgeons, and influence policy makers if presented clearly and unambiguously. The implications of this research are far reaching. We are unable to definitively state which construct is the best choice for all patients, due to the presence of selection effects and residual confounding. However, we believe that the information presented here illustrates the variability, frequency and performance of different constructs currently used in clinical practice. Which in turn, should be used to further inform the consenting process between the patient and the surgeon, and facilitate implant selection. We believe commissioners and policy makers should consider the variability and performance of different implants in the commissioning of healthcare providers.

Patients should be actively involved in decision making about their treatment. Here we provide, for the first time, data which allows patients and clinicians to directly compare revision rates associated with the use of different UKR and TKR constructs. The information provided should be used to inform patient choice, surgical decision making and commissioning.

For peer review only

For peer review only - http://bmjopen.bmj.com/site/about/guidelines.xhtml

Acknowledgments

We thank the patients and staff of all the hospitals who have contributed data to the National Joint Registry. We are grateful to the Healthcare Quality Improvement Partnership, the National Joint Registry Steering Committee, and staff at the National Joint Registry for facilitating this work. The views expressed represent those of the authors and do not necessarily reflect those of the NHS, the National Institute for Health Research, the Department of Health, the National Joint Registry Steering Committee or Healthcare Quality Improvement Partnership, who do not vouch for how the information is presented.

Funding

Posts of members of the research team are funded by a contract grant from the National Joint Registry for England, Wales, Northern Ireland and the Isle of Man. This study was also supported by the NIHR Biomedical Research Centre at the University Hospitals Bristol NHS Foundation Trust and the University of Bristol. AS is funded by an MRC Strategic Skills Fellowship MR/L01226X/1.

Competing interest statement

All authors have completed the Unified Competing Interest form at www.icmje.org/coi_disclosure.pdf (available on request from the corresponding author) and declare: funding was received for the submitted work as described in the Funding statement; AWB and MRW are co-applicants on a grant from Stryker investigating the outcome of the Triathlon total knee replacement; no other financial relationships with any organisations that might have an interest in the submitted work in the previous three years, no other relationships or activities that could appear to have influenced the submitted work.

Author Contributions

AS, MRW and AWB conceived the study. KD, AS and AWB designed the study. The data were extracted by Northgate (Hemel Hempstead, UK). KD and AS managed and analysed data. MRW, AS, MLP and AWB reviewed the published work. All authors critically inputted into final design of the study, interpreted the data and co-wrote the manuscript. AS is the guarantor and attests on behalf of the authors that the manuscript is a true and transparent reflection of the study without omissions.

Role of the funding source

The sponsor of the study had no role in the study design, data collection, analysis, interpretation, or writing of the final report. KD and AS had full access to all of the data and AS had final responsibility for manuscript submission.

For peer review only - http://bmjopen.bmj.com/site/about/guidelines.xhtml

For peer review only

Ethical approval

Pseudo anonymised analysis of NJR data is considered as secondary use of clinical registry data, under HRA guidance this does not require formal ethical approval. However, all research projects are internally approved by the NJR. *The full NJR privacy notice can be found at* *http://www.njrcentre.org.uk/njrcentre/About-the-NJR/Privacy-Notice-GDPR*

Data sharing

Access to data is available from the National Joint Registry for England and Wales.

For peer review only

For peer review only - http://bmjopen.bmj.com/site/about/guidelines.xhtml

Figures

Figure 1: Schematic representation of inferiority and non-inferiority

Figure 2: Difference in cumulative revision of knee constructs compared to a contemporary benchmark at 5 years, using all total knee and unicondylar replacements with ≥500 procedures remaining at risk.

Figure 3: Difference in cumulative revision of knee constructs compared to a contemporary benchmark at 10 years, using all total knee and unicondylar replacements with ≥500 procedures remaining at risk.

Figure 4: Difference in cumulative revision of knee constructs compared to a contemporary benchmark at 7 years in women aged between 55 and 75 years, using all total knee and unicondylar replacements with ≥500 procedures remaining at risk.

Figure 5: Difference in cumulative revision of knee constructs compared to a contemporary benchmark at 7 years in men aged between 55 and 75 years, using all total knee and unicondylar replacements with ≥500 procedures remaining at risk.

For peer review only

For peer review only - http://bmjopen.bmj.com/site/about/guidelines.xhtml

REFERENCES

1. National Joint Registry for England W, Northern Ireland and the Isle of Man. 14th Annual report.http://www.njrreports.org.uk/Portals/0/PDFdownloads/NJR%2014th%20Annual%20Report%202017.pdf
2. Tucker K. Orthopaedic Data Evaluation Panel: ODEP. Northgate Public Services (UK) Ltd. 2017 [Available from: http://www.odep.org.uk/ODEPProcess/ODEPStatement.aspx.
3. Nederlandse Orthopaedische Vereniging. Classification Orthopaedic Implants: NOV Total Knee Prosthesis 2016-2017 [Available from: https://www.orthopeden.org/downloads/146/classificatie-knie-2017.pdf.
4. Prostheses and Devices Committee (PDC) and Prostheses List Advisory Committee (PLAC) Bulletins. Australian Government; Department of Health [Available from: http://www.health.gov.au/internet/main/publishing.nsf/Content/health-privatehealth-prostheseslist.htm.
5. Sayers A, Crowther MJ, Judge A, et al. Determining the sample size required to establish whether a medical device is non-inferior to an external benchmark. *BMJ Open* 2017;7(8):e015397. doi: 10.1136/bmjopen-2016-015397 [published Online First: 2017/08/31]
6. Schumi J, Wittes JT. Through the looking glass: understanding non-inferiority. *Trials* 2011;12:106. doi: 10.1186/1745-6215-12-106
7. Tunes da Silva G, Logan BR, Klein JP. Methods for equivalence and noninferiority testing. *Biol Blood Marrow Transplant* 2009;15(1 Suppl):120-7. doi: 10.1016/j.bbmt.2008.10.004
8. Com-Nougue C, Rodary C, Patte C. How to establish equivalence when data are censored: a randomized trial of treatments for B non-Hodgkin lymphoma. *Stat Med* 1993;12(14):1353-64.
9. Deere K, Whitehouse MR, Porter M, et al. Assessing the non-inferiority of prosthesis constructs used in hip replacement using data from the National Joint Registry of England, Wales, Northern Ireland and the Isle of Man: A Benchmarking study. *BMJ Open* 2019:xxx. doi: xxx/bmjopen-2019-xxx
10. Sayers A, Evans JT, Whitehouse MR, et al. Are competing risks models appropriate to describe implant failure? *Acta Orthop* 2018;89(3):256-58. doi: 10.1080/17453674.2018.1444876 [published Online First: 2018/03/10]
11. Greenwood M. The natural duration of cancer. *Rep **Public Health** Med Subj (Lond)* 1926;33: 1-26
12. Donken R, de Melker HE, Rots NY, et al. Comparing vaccines: a systematic review of the use of the non-inferiority margin in vaccine trials. *Vaccine* 2015;33(12):1426-32. doi: 10.1016/j.vaccine.2015.01.072
13. Liddle AD, Judge A, Pandit H, et al. Adverse outcomes after total and unicompartmental knee replacement in 101,330 matched patients: a study of data from the National Joint Registry for England and Wales. *Lancet* 2014;384(9952):1437-45. doi: 10.1016/S0140-6736(14)60419-0 [published Online First: 2014/07/12]
14. Hunt LP, Ben-Shlomo Y, Clark EM, et al. 45-day mortality after 467,779 knee replacements for osteoarthritis from the National Joint Registry for England and Wales: an observational study. *Lancet* 2014;384(9952):1429-36. doi: 10.1016/S0140-6736(14)60540-7 [published Online First: 2014/07/12]
15. Kleeblad LJ, van der List JP, Zuiderbaan HA, et al. Larger range of motion and increased return to activity, but higher revision rates following unicompartmental versus total knee arthroplasty in patients under 65: a systematic review. *Knee Surg Sports Traumatol Arthrosc* 2017 doi: 10.1007/s00167-017-4817-y [published Online First: 2017/12/01]
16. Liddle AD, Pandit H, Judge A, et al. Patient-reported outcomes after total and unicompartmental knee arthroplasty: a study of 14,076 matched patients from the National Joint Registry for England and Wales. *Bone Joint J* 2015;97-B(6):793-801. doi: 10.1302/0301-620X.97B6.35155 [published Online First: 2015/06/03]

For peer review only - http://bmjopen.bmj.com/site/about/guidelines.xhtml

17. Burn E, Liddle AD, Hamilton TW, et al. Cost-effectiveness of unicompartmental compared with total knee replacement: a population-based study using data from the National Joint Registry for England and Wales. *BMJ Open* 2018;8(4):e020977. doi: 10.1136/bmjopen-2017-020977 [published Online First: 2018/05/01]
18. NJR Data Quality Audit 2017 [Available from: http://www.njrcentre.org.uk/njrcentre/NewsandEvents/DataQualityAudit-updateSeptember2017/tabid/1451/Default.aspx.

For peer review only

For peer review only - http://bmjopen.bmj.com/site/about/guidelines.xhtml

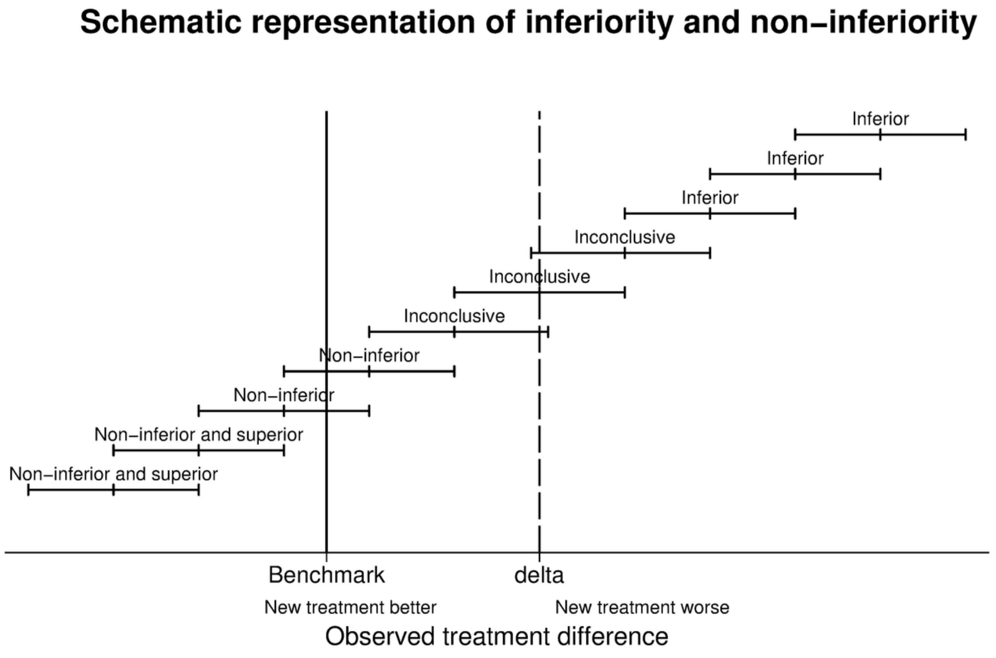

101x67mm (300 x 300 DPI)

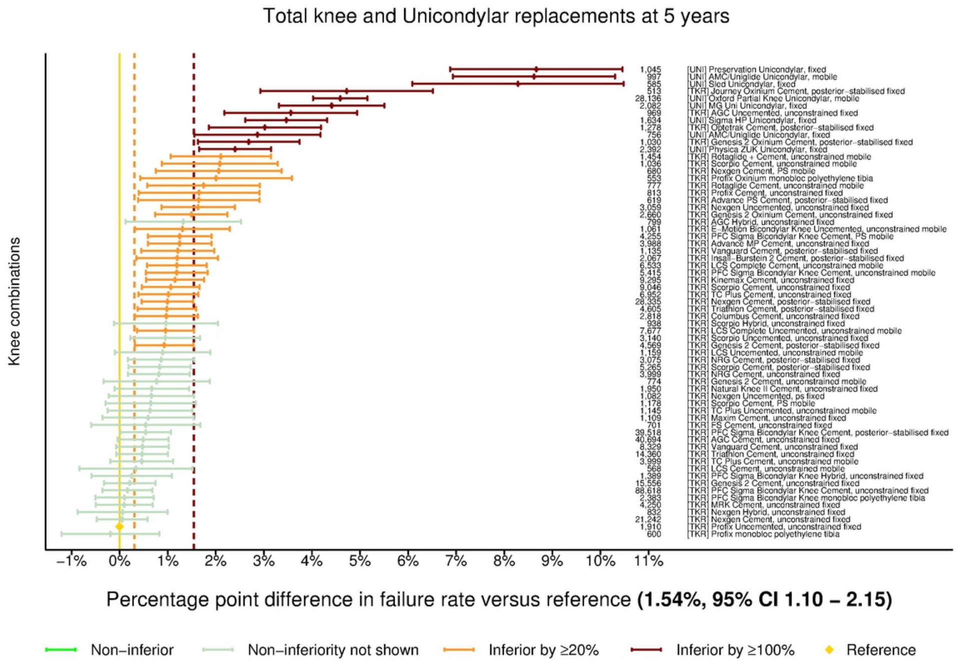

101x73mm (300 x 300 DPI)

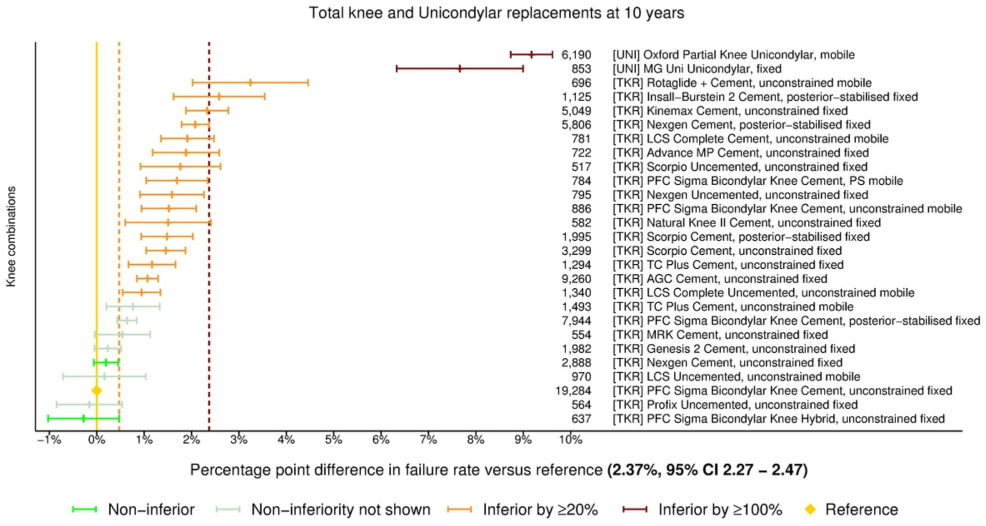

101x58mm (300 x 300 DPI)

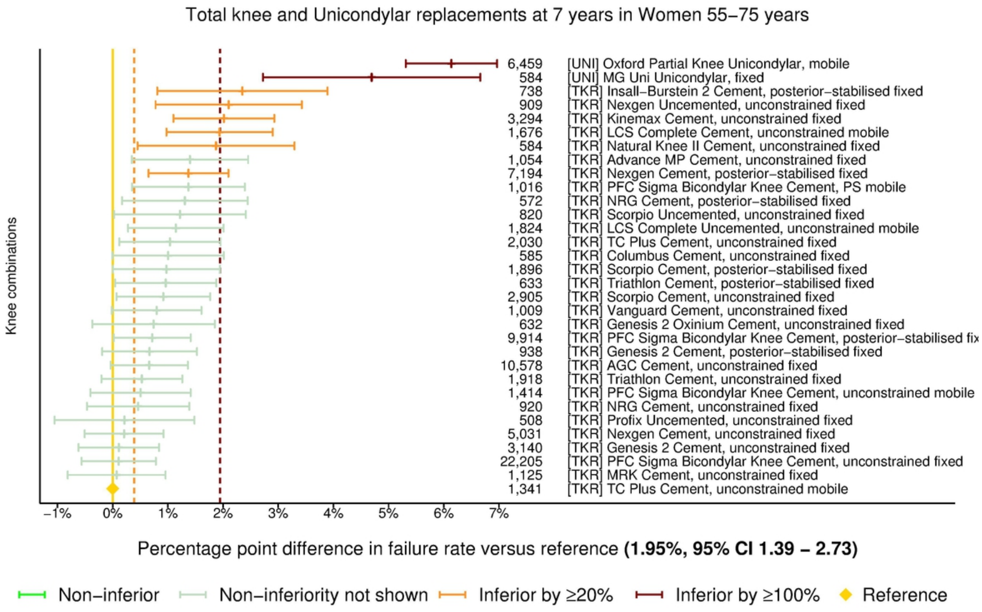

101x67mm (300 x 300 DPI)

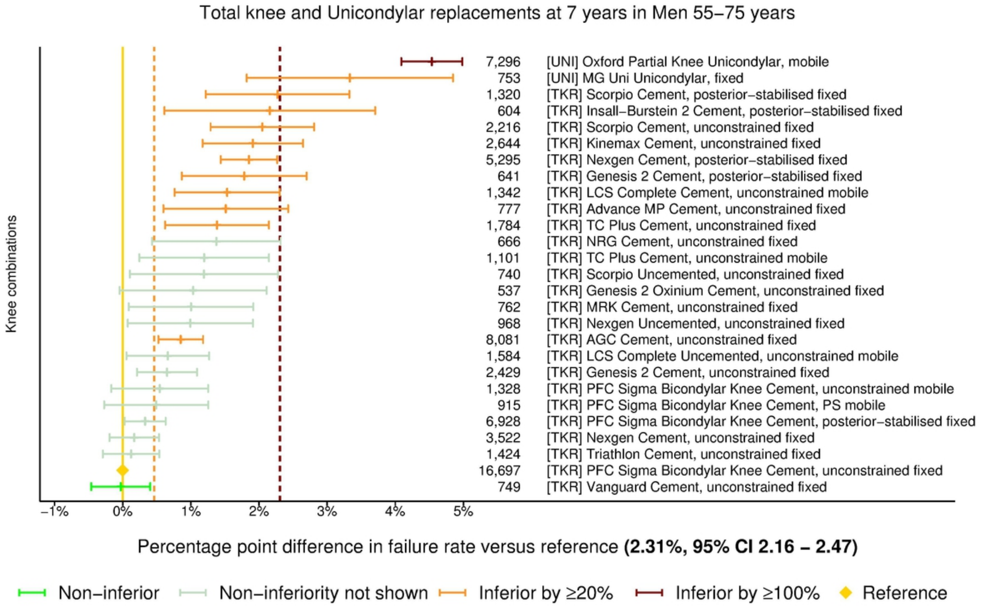

101x67mm (300 x 300 DPI)

Supplementary Figure 1: Flow diagram of showing derivation of procedures used in analyses

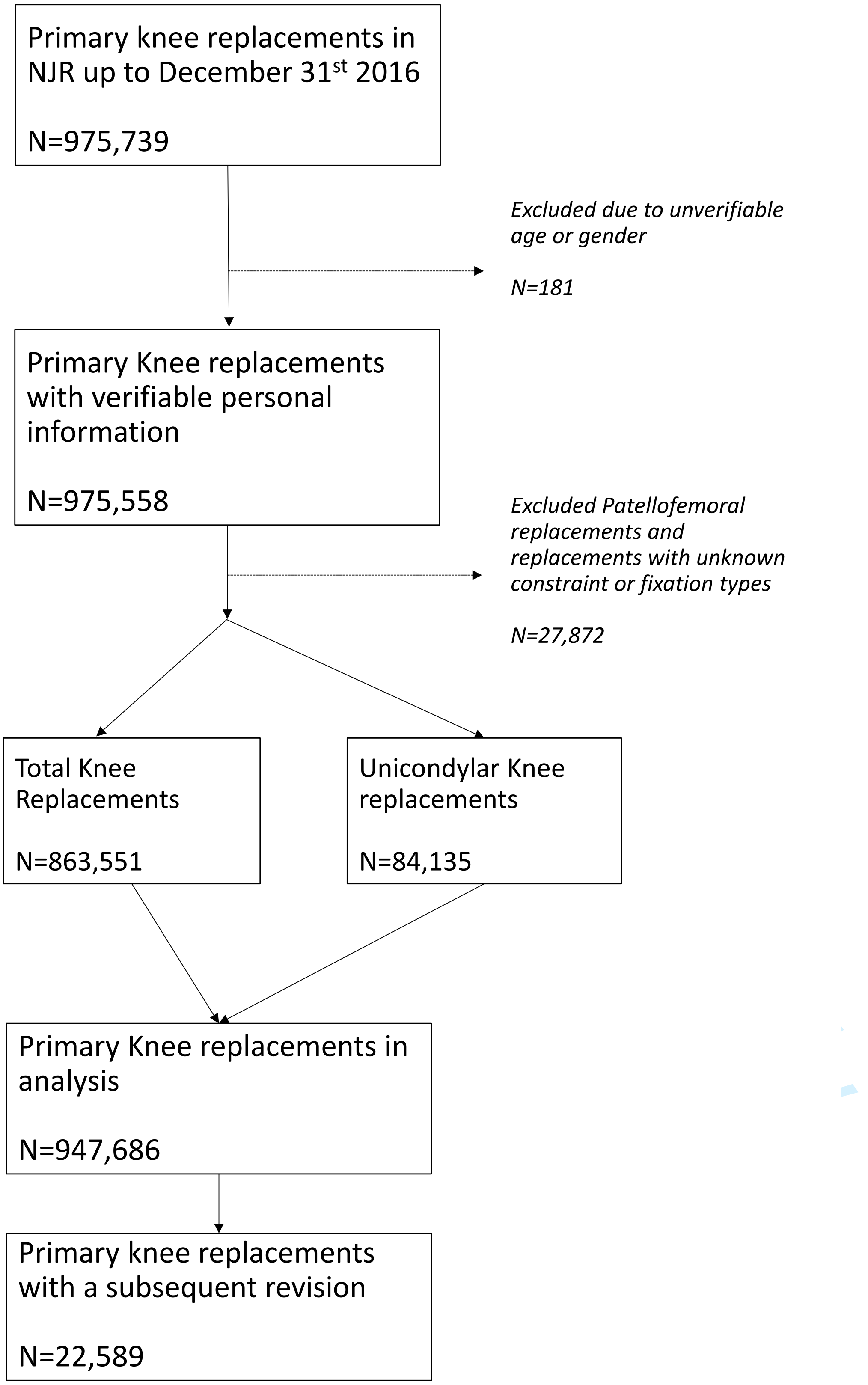

Supplementary Figure 2: Difference in cumulative revision of knee implants compared to a contemporary reference at 3 years, using all total knee and unicondylar replacements with ≥500 procedures remaining at risk.

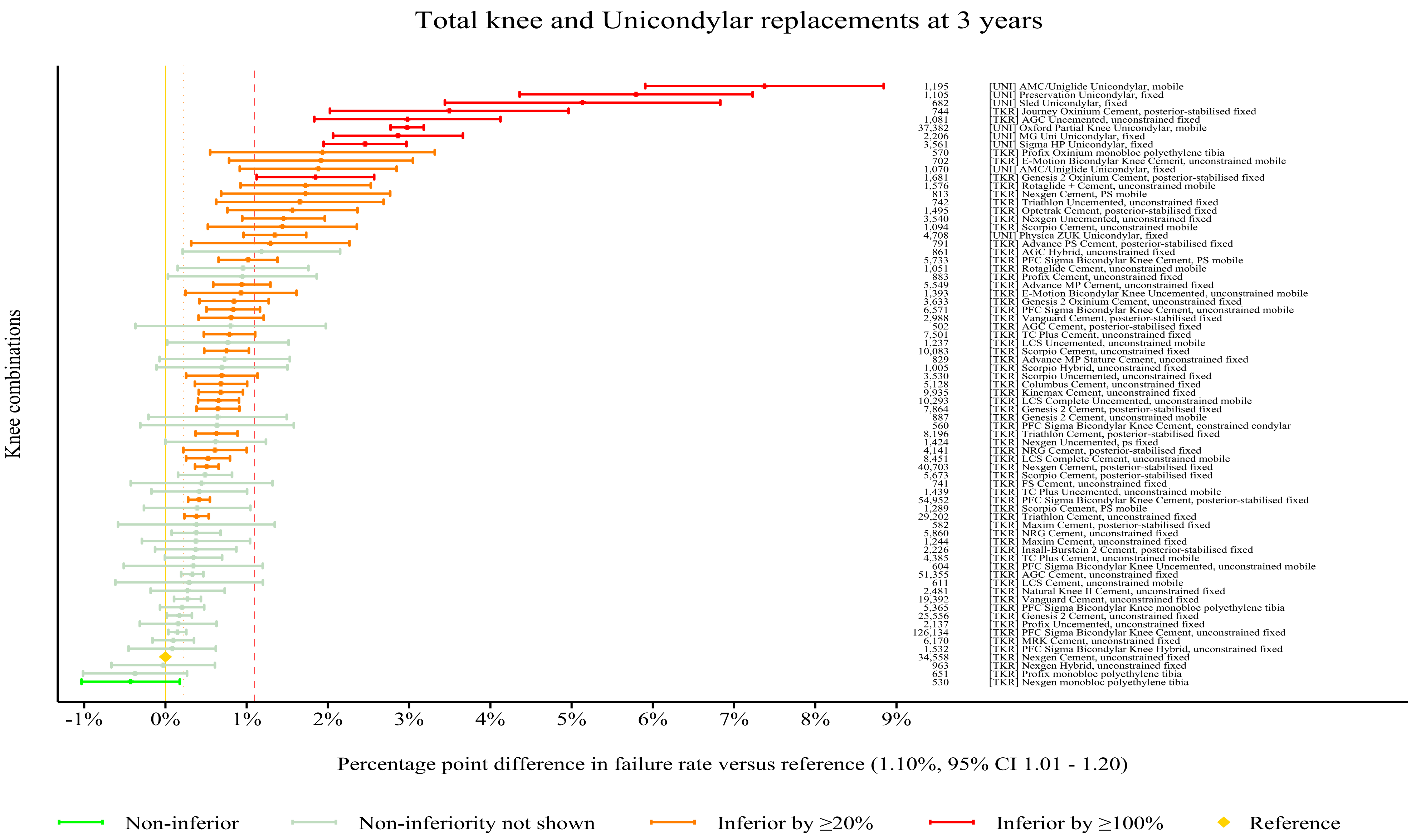

Supplementary Figure 3: Difference in cumulative revision of knee implants compared to a contemporary reference at 7 years, using all total knee and unicondylar replacements with ≥500 procedures remaining at risk.

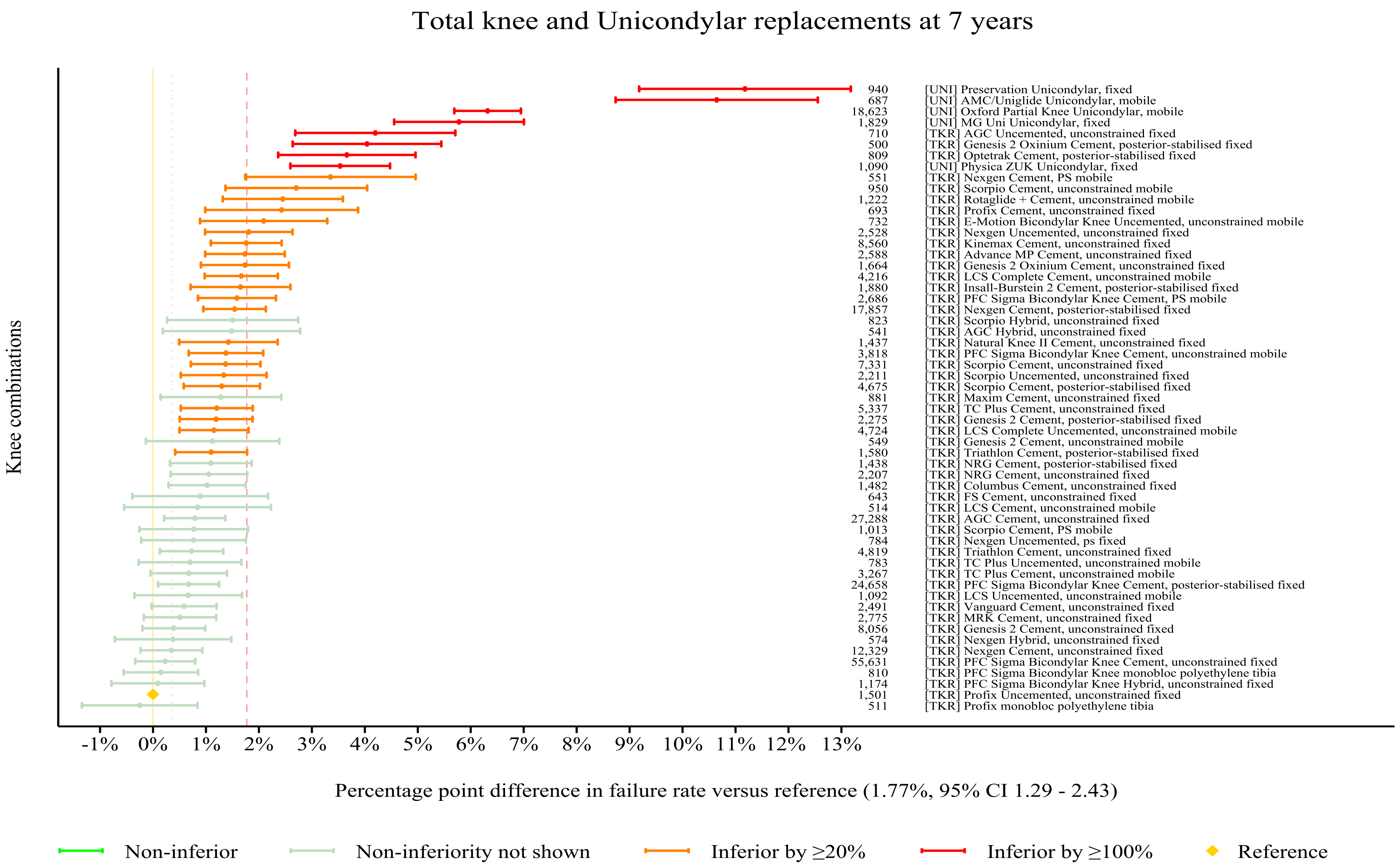

Supplementary Figure 4: Difference in cumulative revision of knee implants compared to a contemporary reference at 3 years in men, using all total knee and unicondylar replacements with ≥500 procedures remaining at risk.

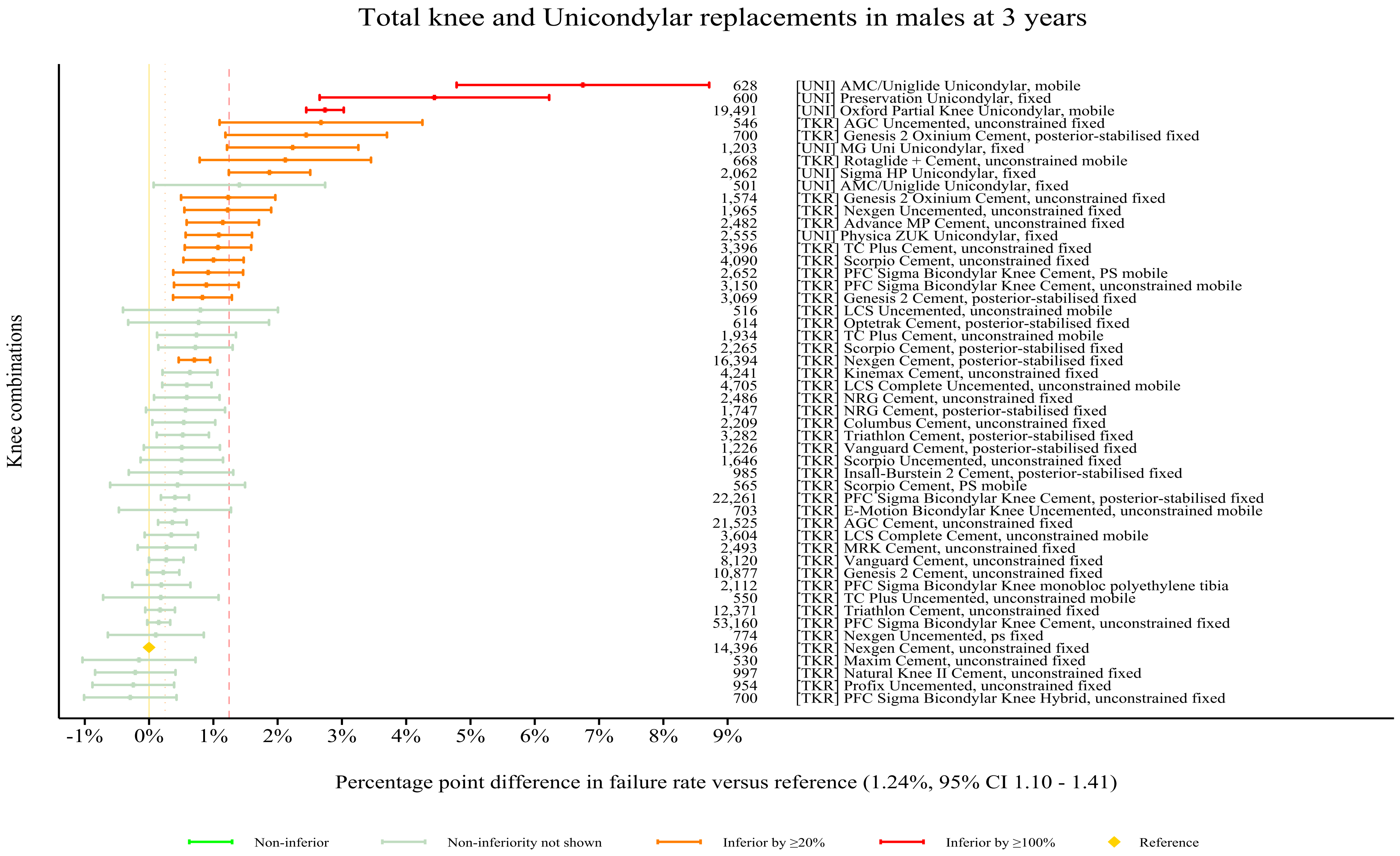

Supplementary Figure 5: Difference in cumulative revision of knee implants compared to a contemporary reference at 5 years in men, using all total knee and unicondylar replacements with ≥500 procedures remaining at risk.

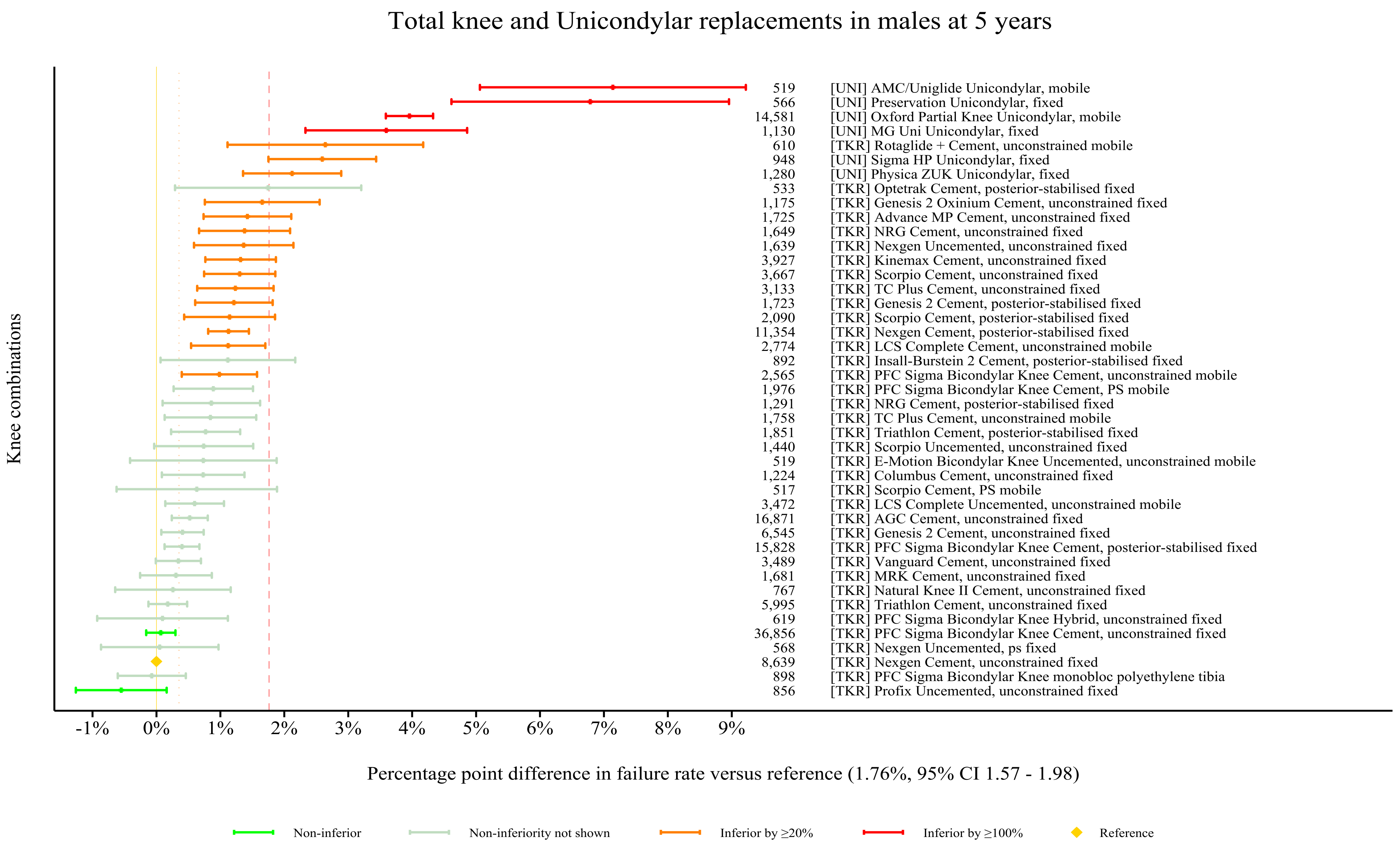

Supplementary Figure 6: Difference in cumulative revision of knee implants compared to a contemporary reference at 7 years in men, using all total knee and unicondylar replacements with ≥500 procedures remaining at risk.

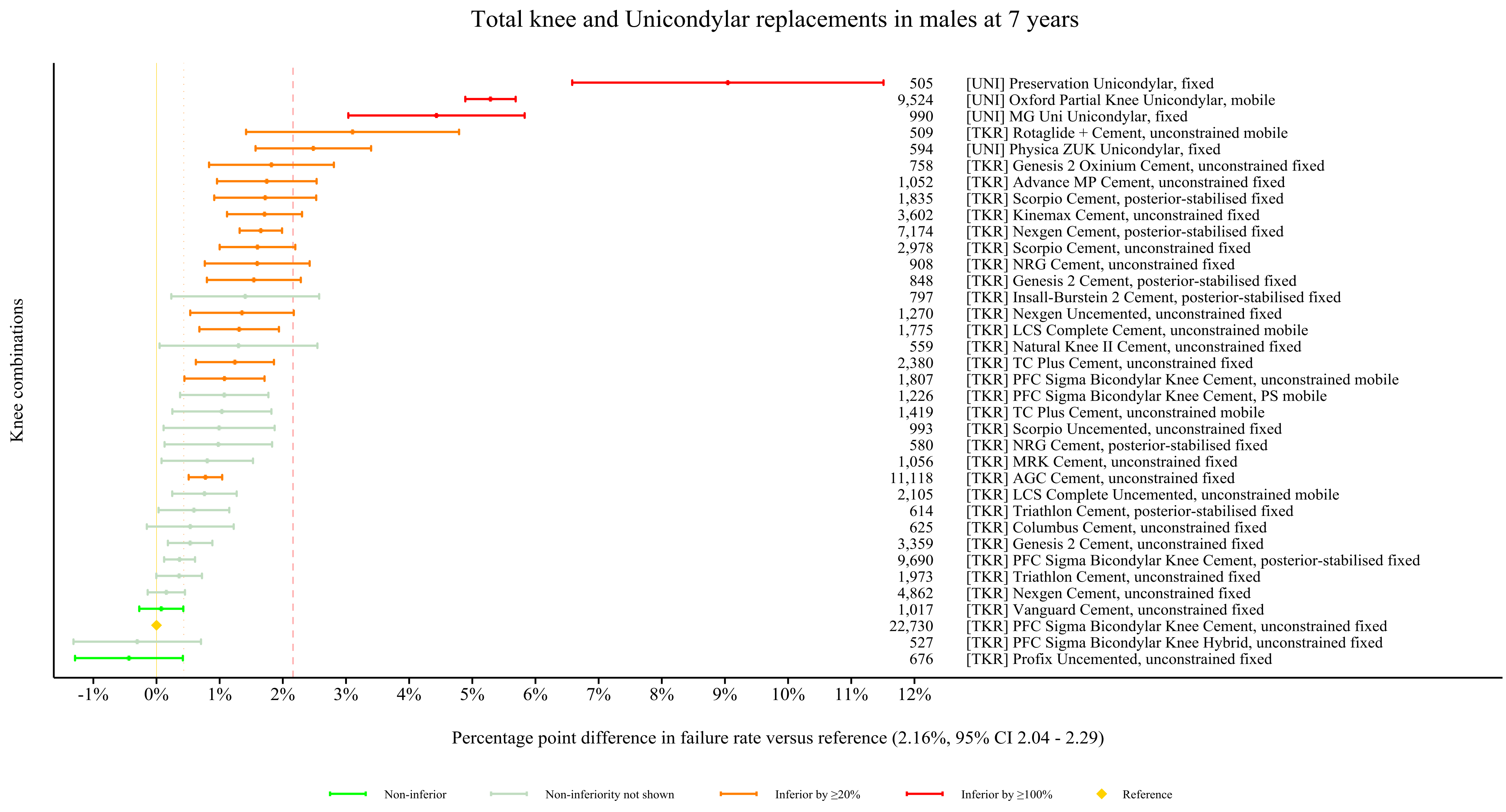

Supplementary Figure 7: Difference in cumulative revision of knee implants compared to a contemporary reference at 10 years in men, using all total knee and unicondylar replacements with ≥500 procedures remaining at risk.

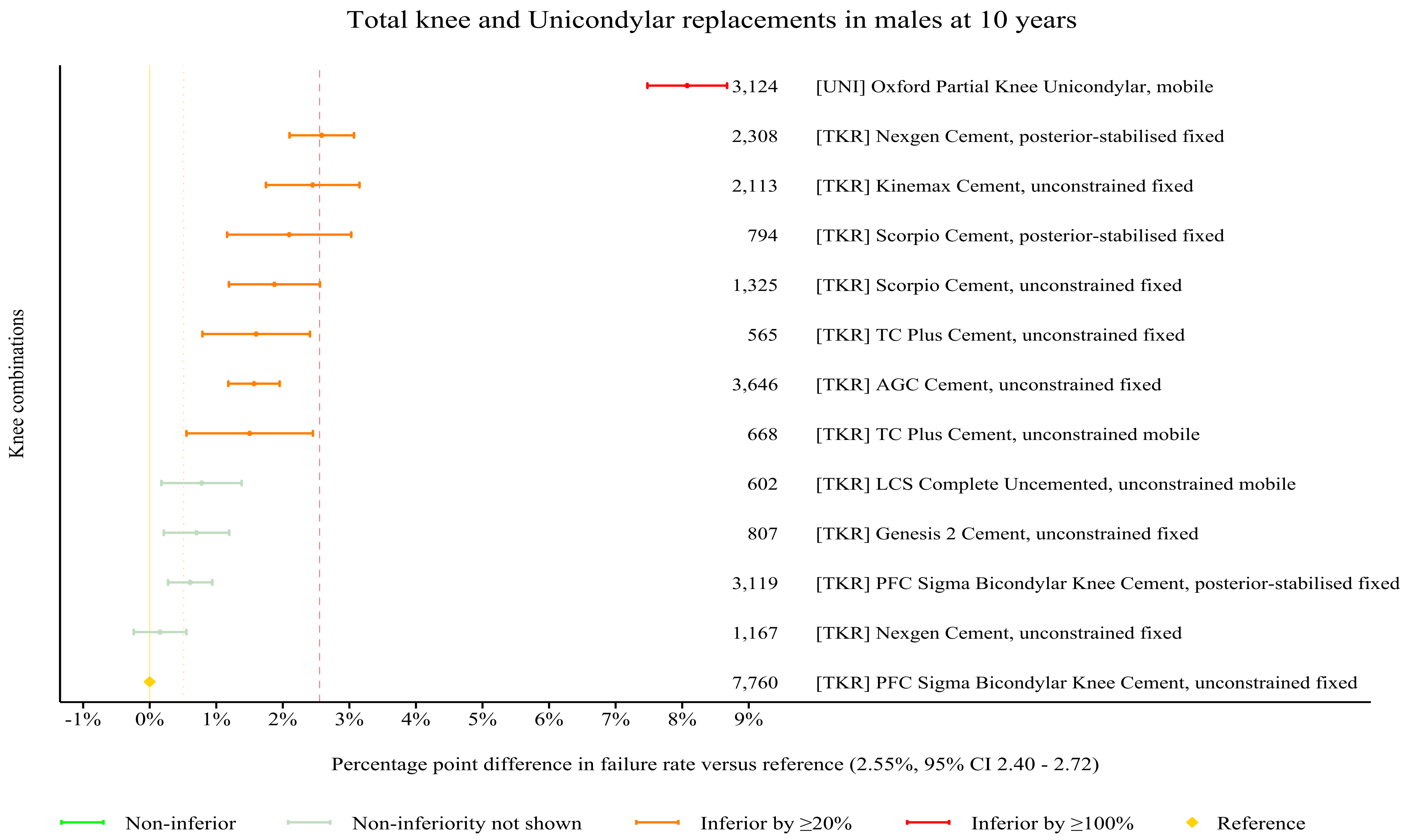

Supplementary Figure 8: Difference in cumulative revision of knee implants compared to a contemporary reference at 3 years in women, using all total knee and unicondylar replacements with ≥500 procedures remaining at risk.

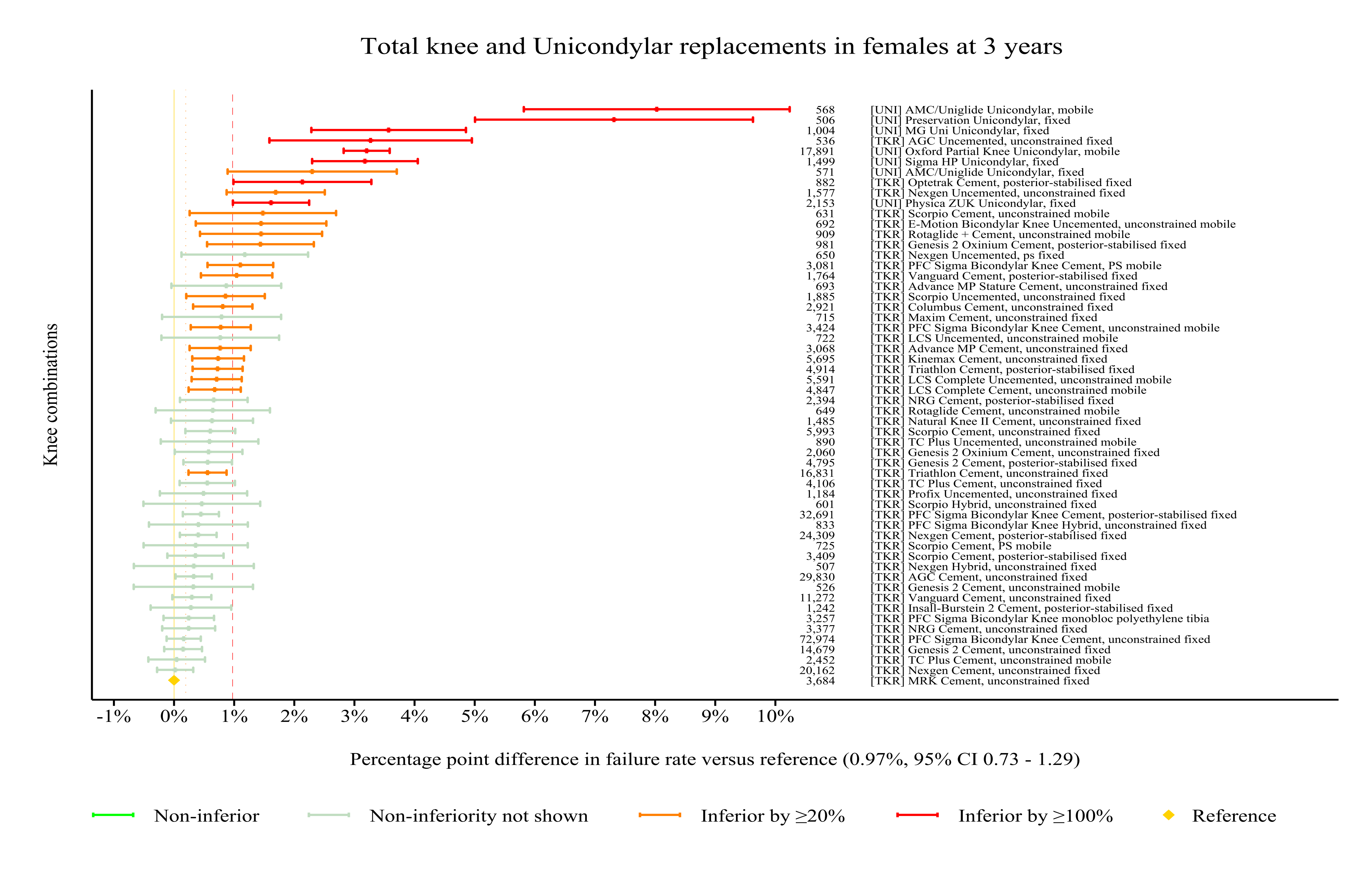

Supplementary Figure 9: Difference in cumulative revision of knee implants compared to a contemporary reference at 5 years in women, using all total knee and unicondylar replacements with ≥500 procedures remaining at risk.

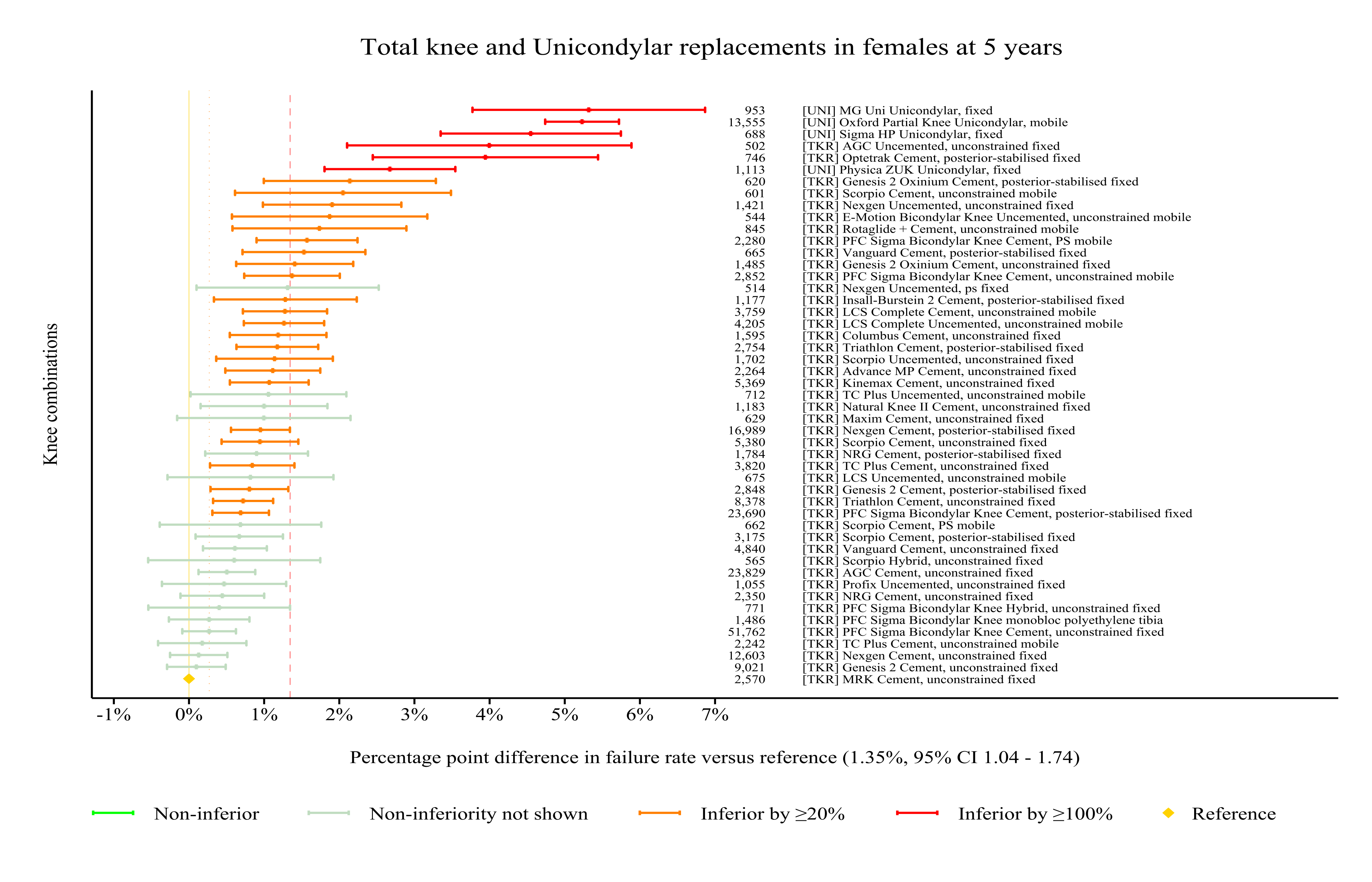

Supplementary Figure 10: Difference in cumulative revision of knee implants compared to a contemporary reference at 7 years in women, using all total knee and unicondylar replacements with ≥500 procedures remaining at risk.

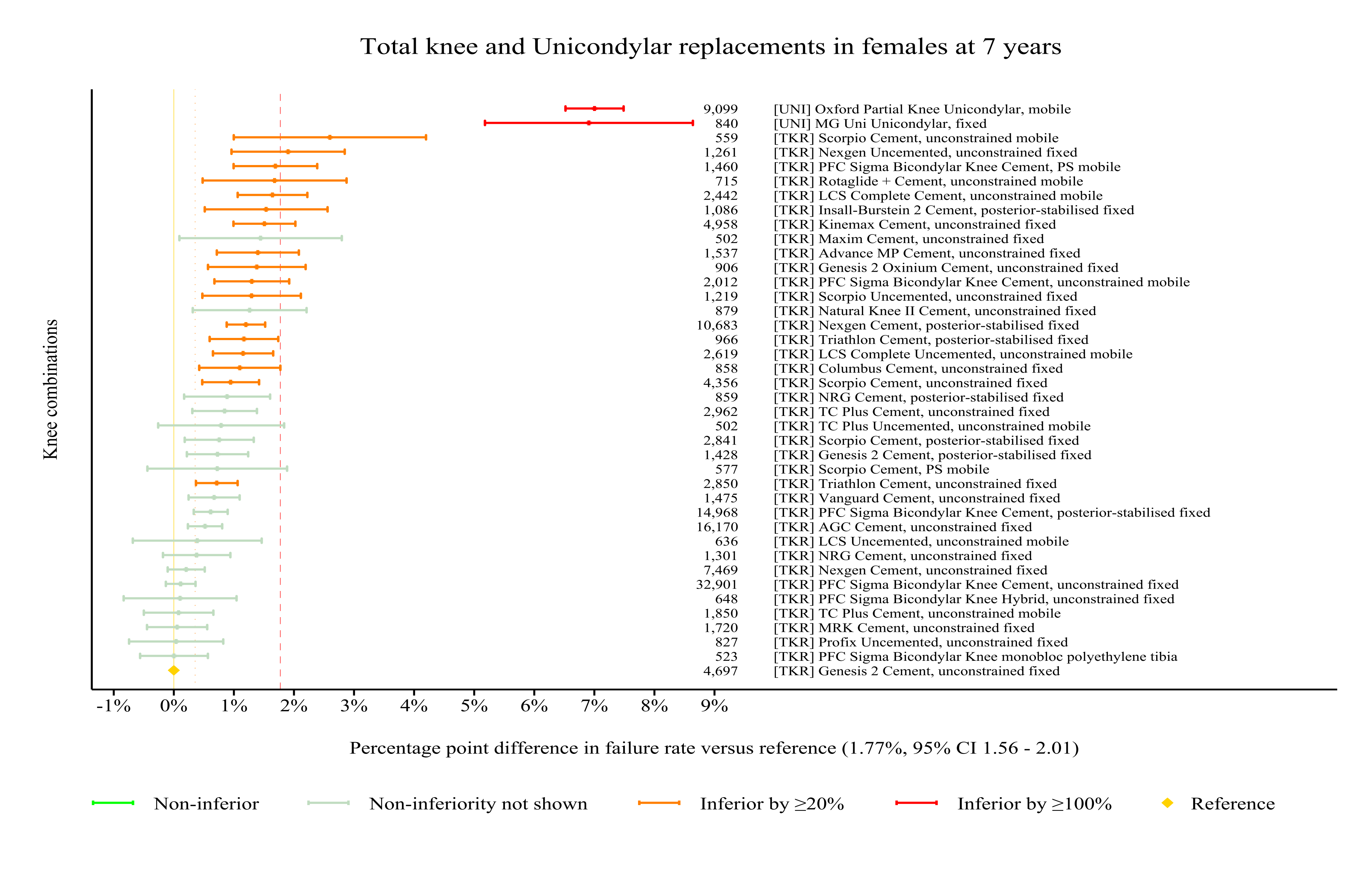

Supplementary Figure 11: Difference in cumulative revision of knee implants compared to a contemporary reference at 10 years in women, using all total knee and unicondylar replacements with ≥500 procedures remaining at risk.

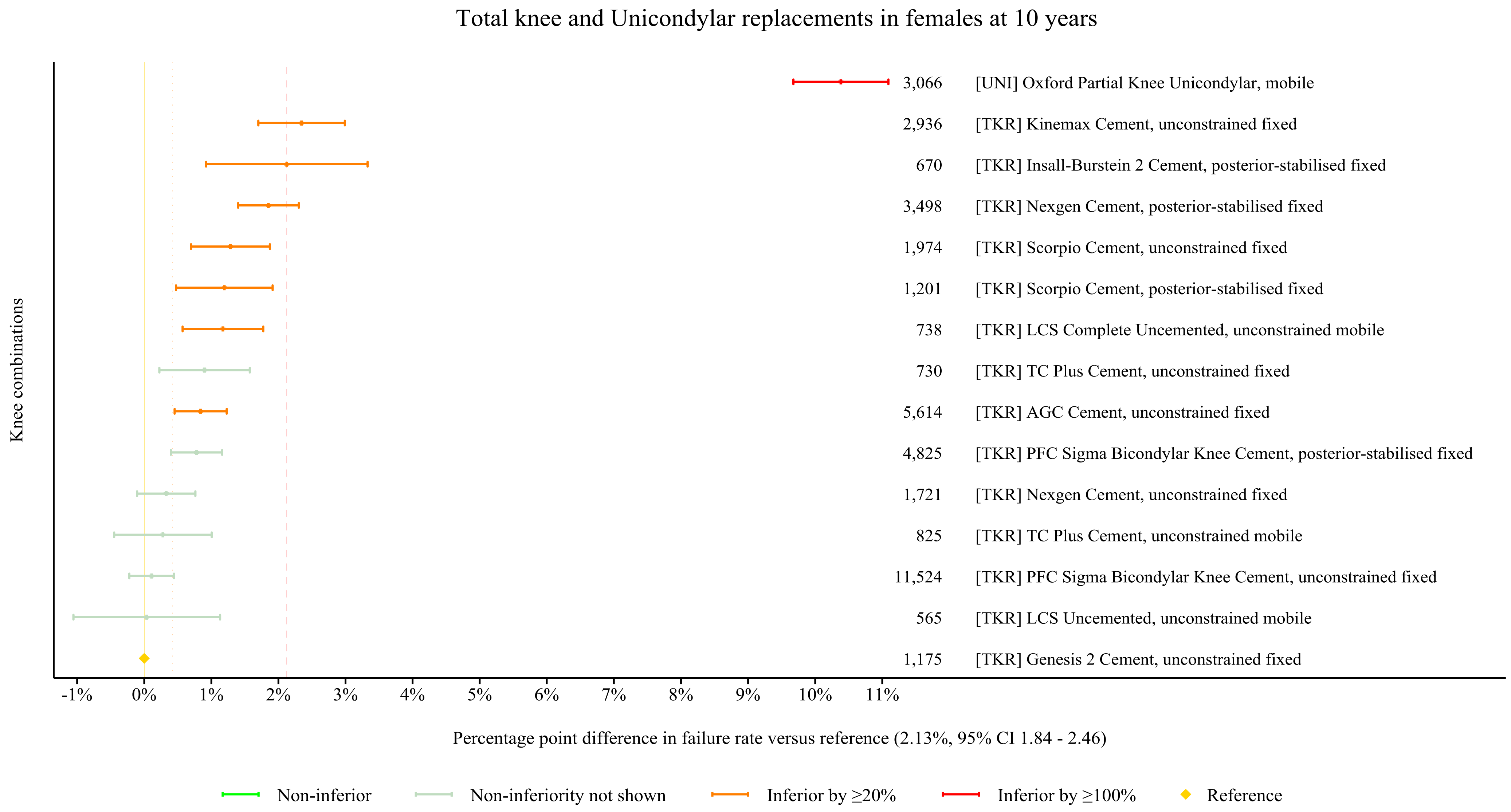

Supplementary Figure 12: Difference in cumulative revision of knee implants compared to a contemporary reference at 3 years in men less than 55 years, using all total knee and unicondylar replacements with ≥500 procedures remaining at risk.

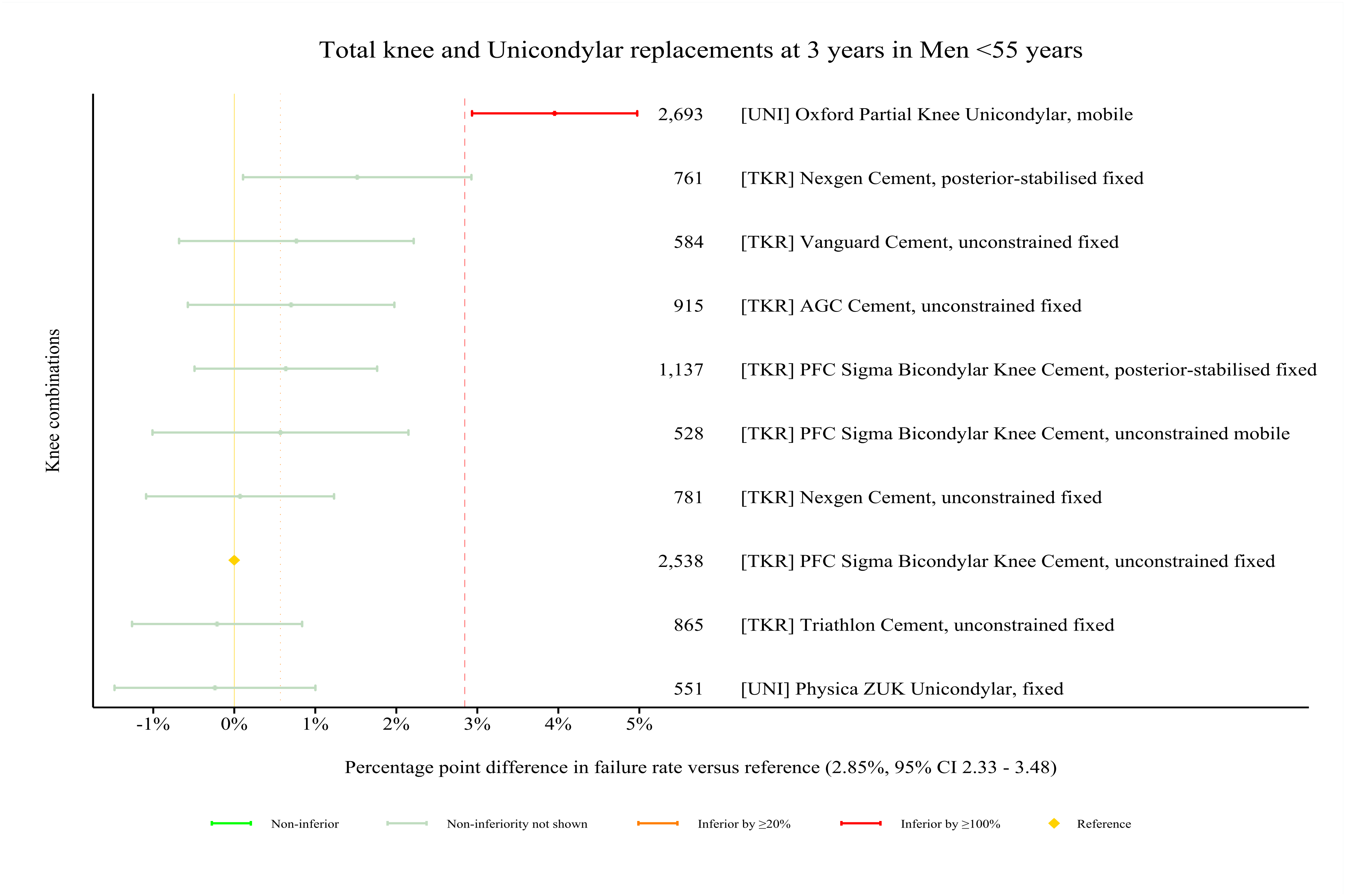

Supplementary Figure 13: Difference in cumulative revision of knee implants compared to a contemporary reference at 5 years in men less than 55 years, using all total knee and unicondylar replacements with ≥500 procedures remaining at risk.

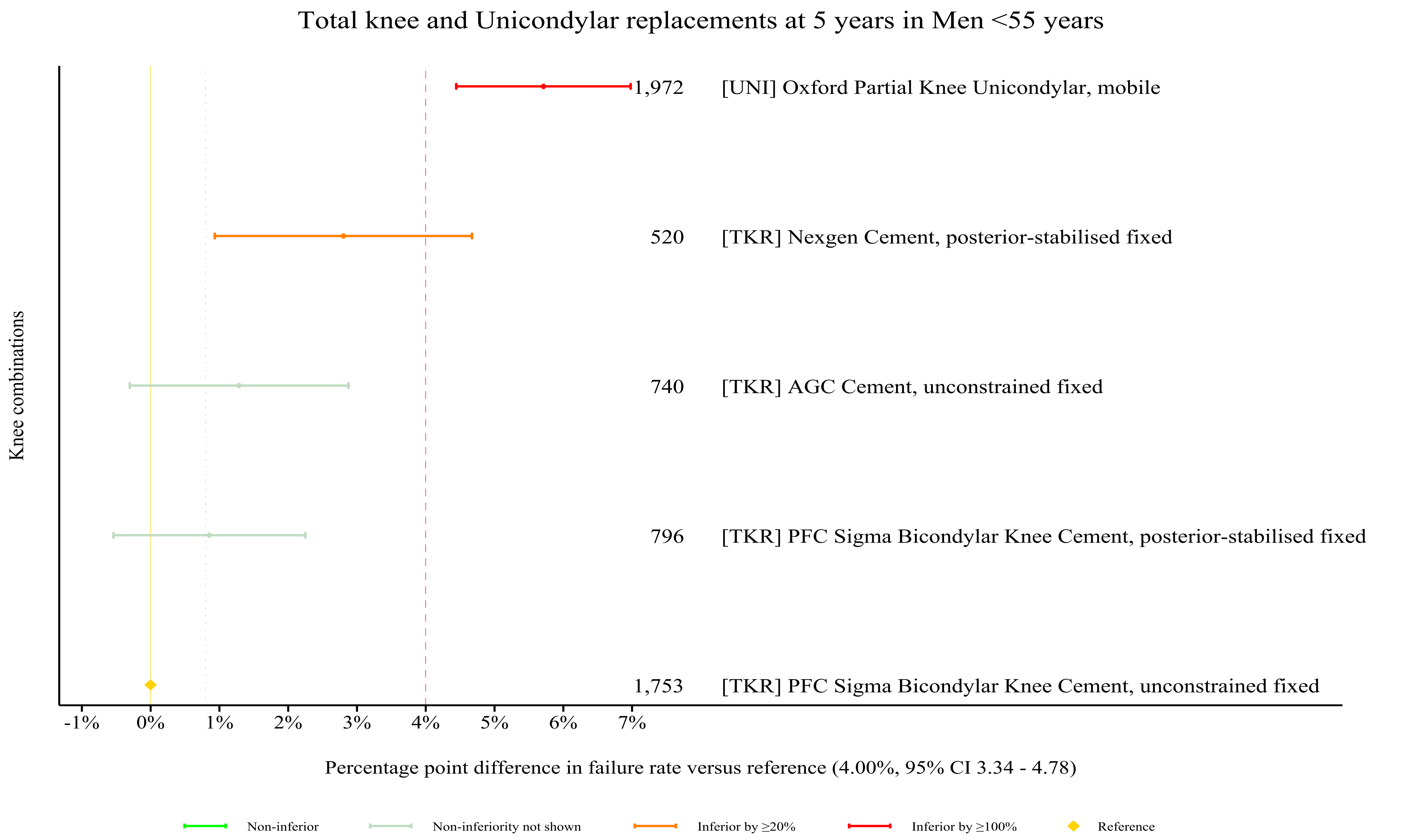

Supplementary Figure 14: Difference in cumulative revision of knee implants compared to a contemporary reference at 7 years in men less than 55 years, using all total knee and unicondylar replacements with ≥500 procedures remaining at risk.

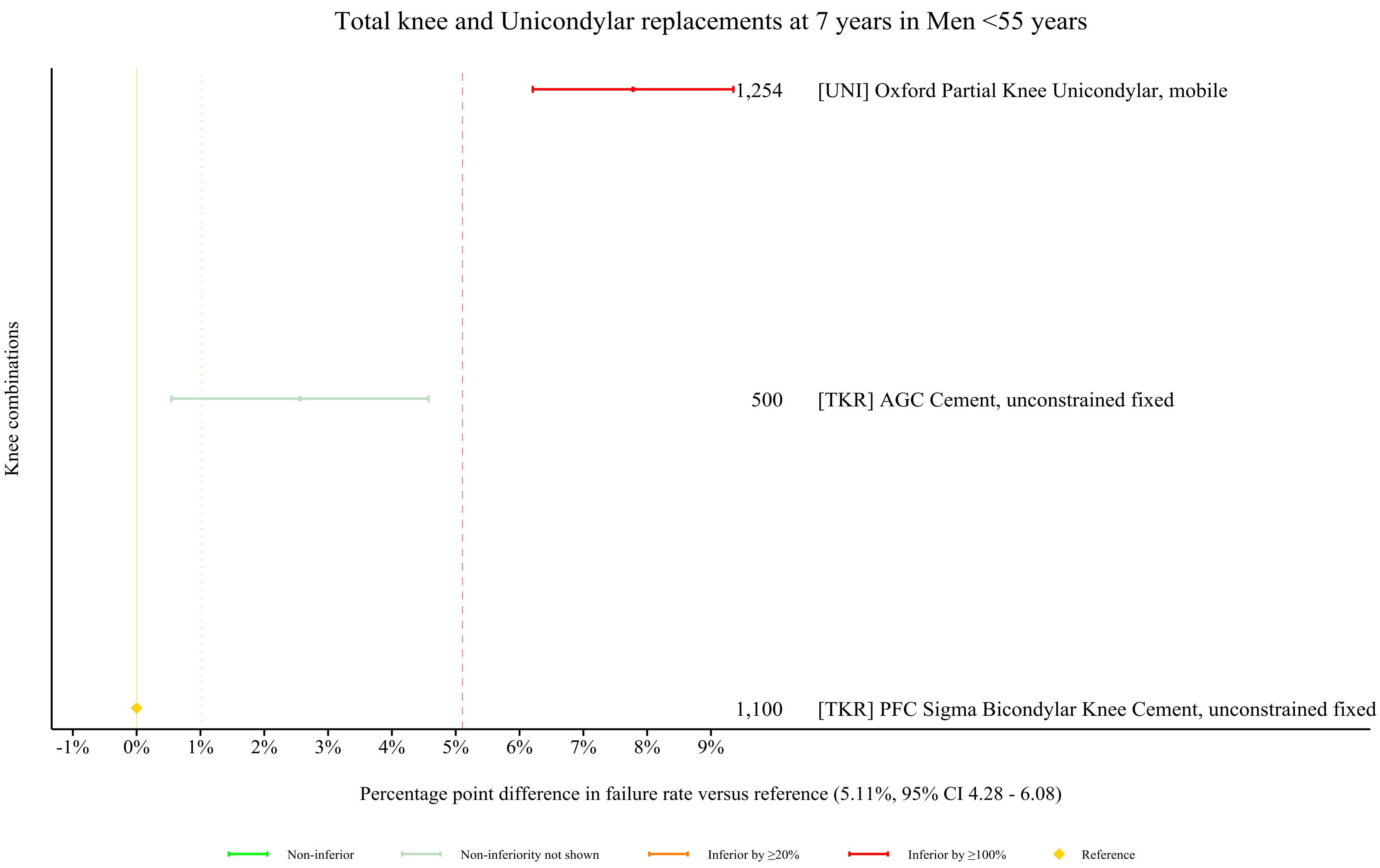

Supplementary Figure 15: Difference in cumulative revision of knee implants compared to a contemporary reference at 3 years in women less than 55 years, using all total knee and unicondylar replacements with ≥500 procedures remaining at risk.

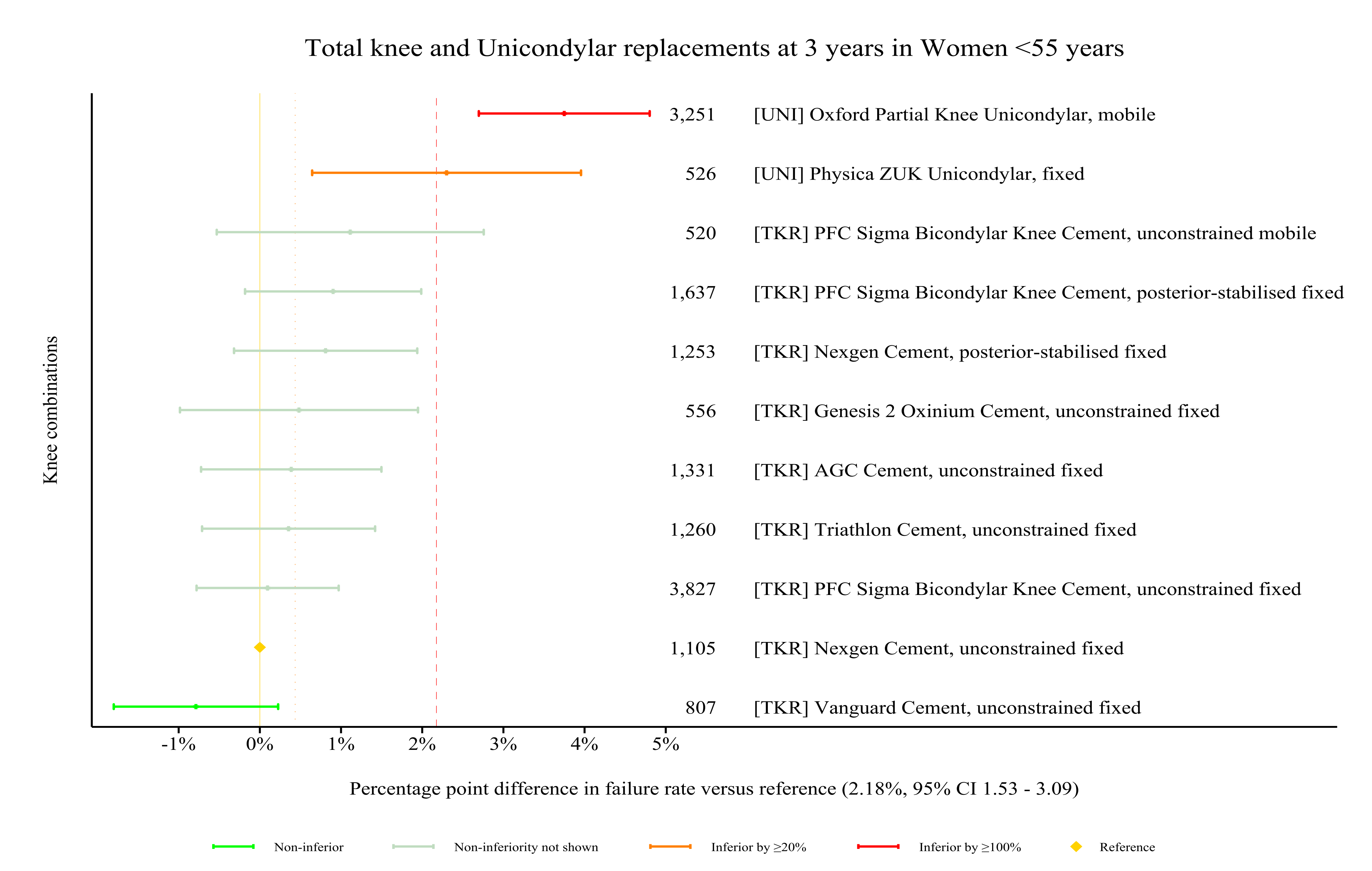

Supplementary Figure 16: Difference in cumulative revision of knee implants compared to a contemporary reference at 5 years in women less than 55 years, using all total knee and unicondylar replacements with ≥500 procedures remaining at risk.

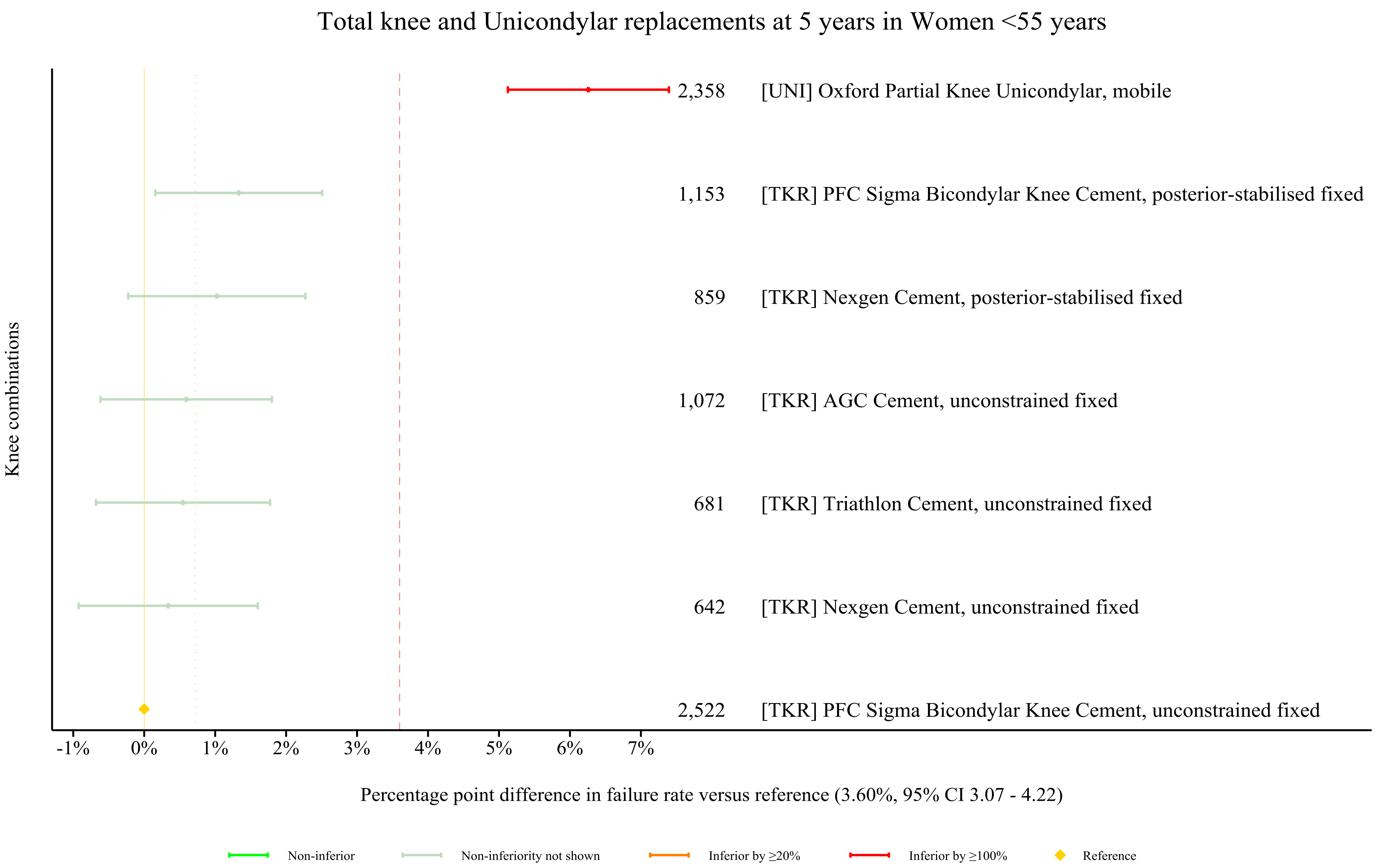

Supplementary Figure 17: Difference in cumulative revision of knee implants compared to a contemporary reference at 7 years in women less than 55 years, using all total knee and unicondylar replacements with ≥500 procedures remaining at risk.

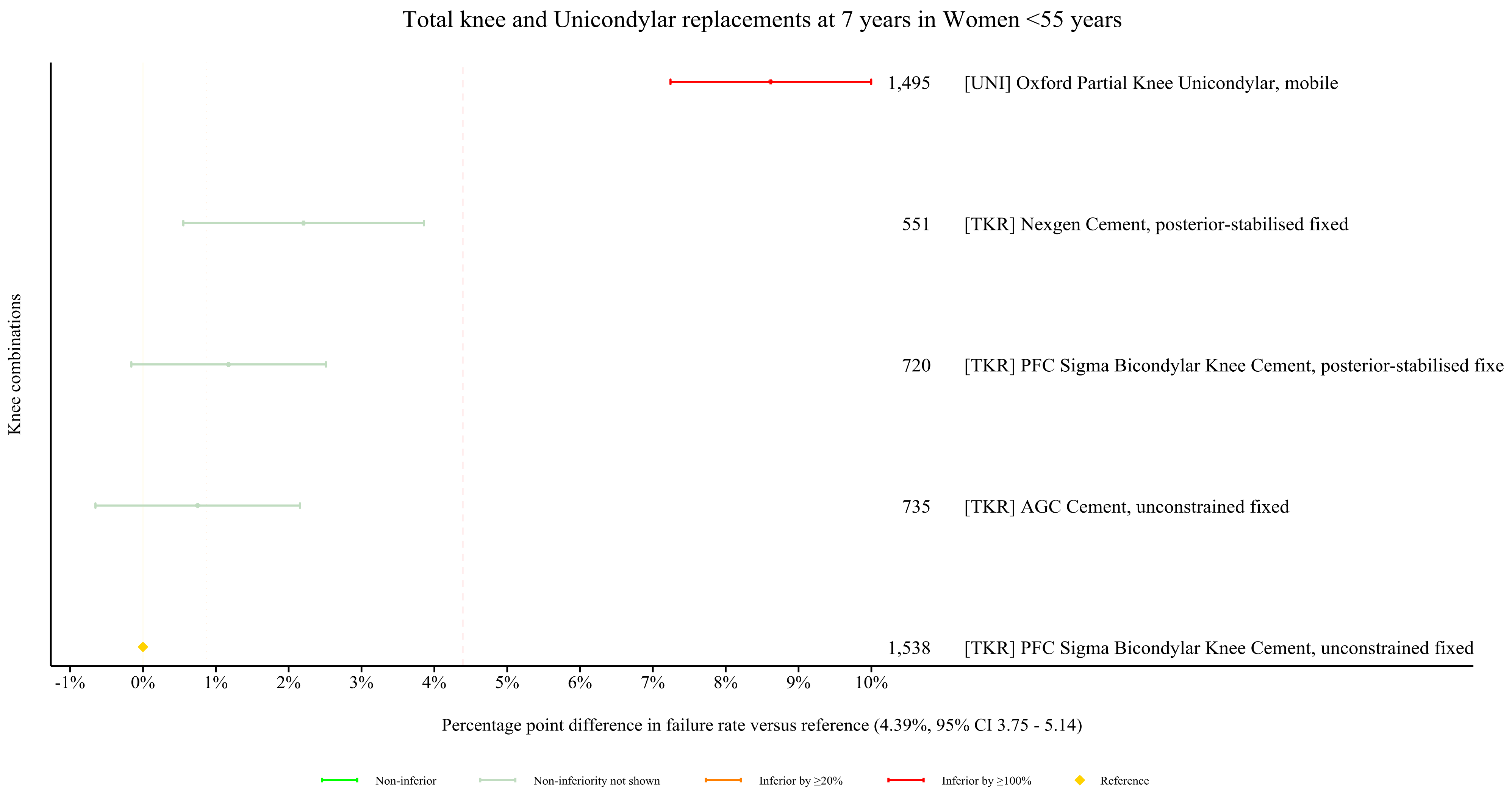

Supplementary Figure 18: Difference in cumulative revision of knee implants compared to a contemporary reference at 3 years in men aged between 55 and 75 years, using all total knee and unicondylar replacements with ≥500 procedures remaining at risk.

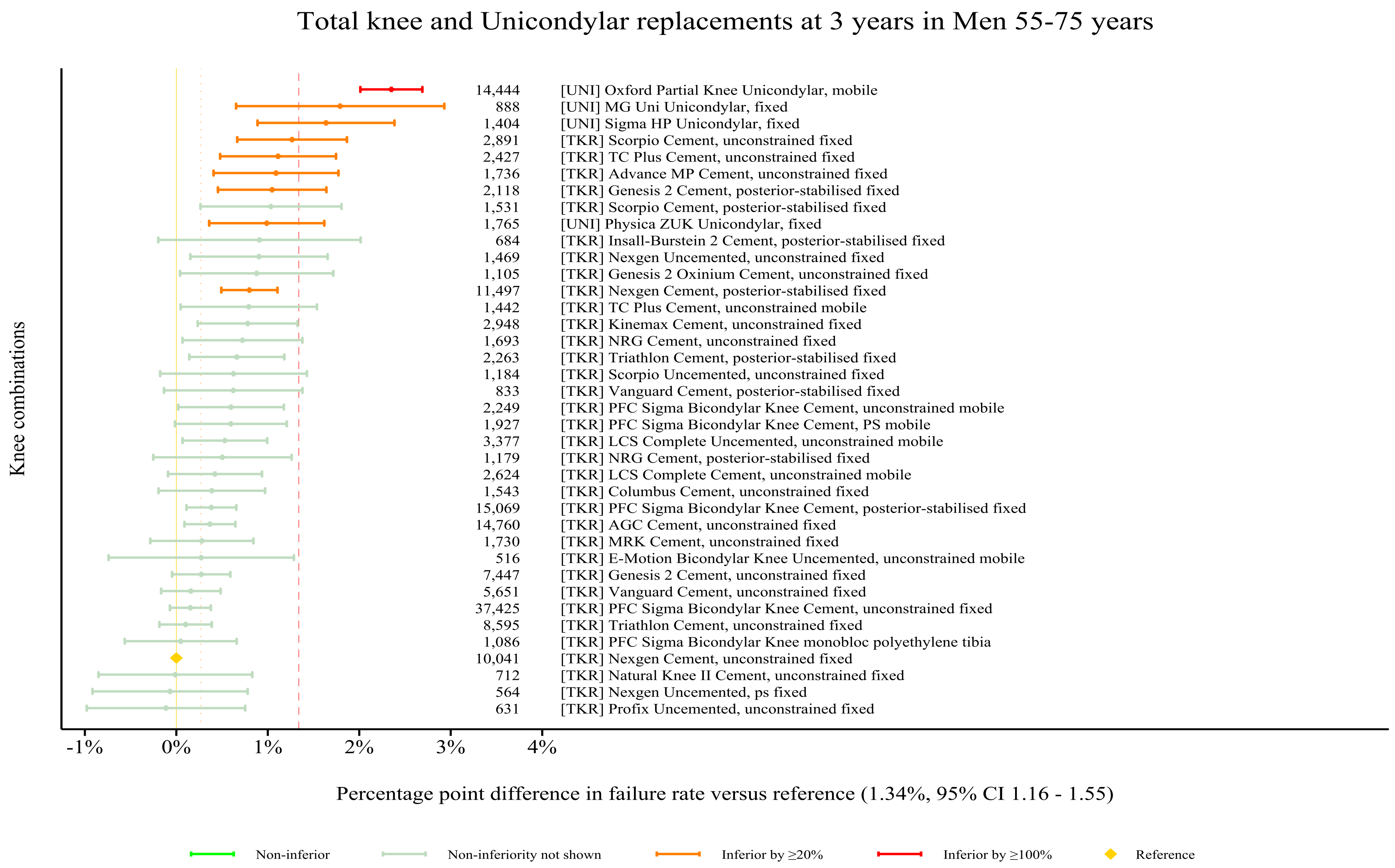

Supplementary Figure 19: Difference in cumulative revision of knee implants compared to a contemporary reference at 5 years in men aged between 55 and 75 years, using all total knee and unicondylar replacements with ≥500 procedures remaining at risk.

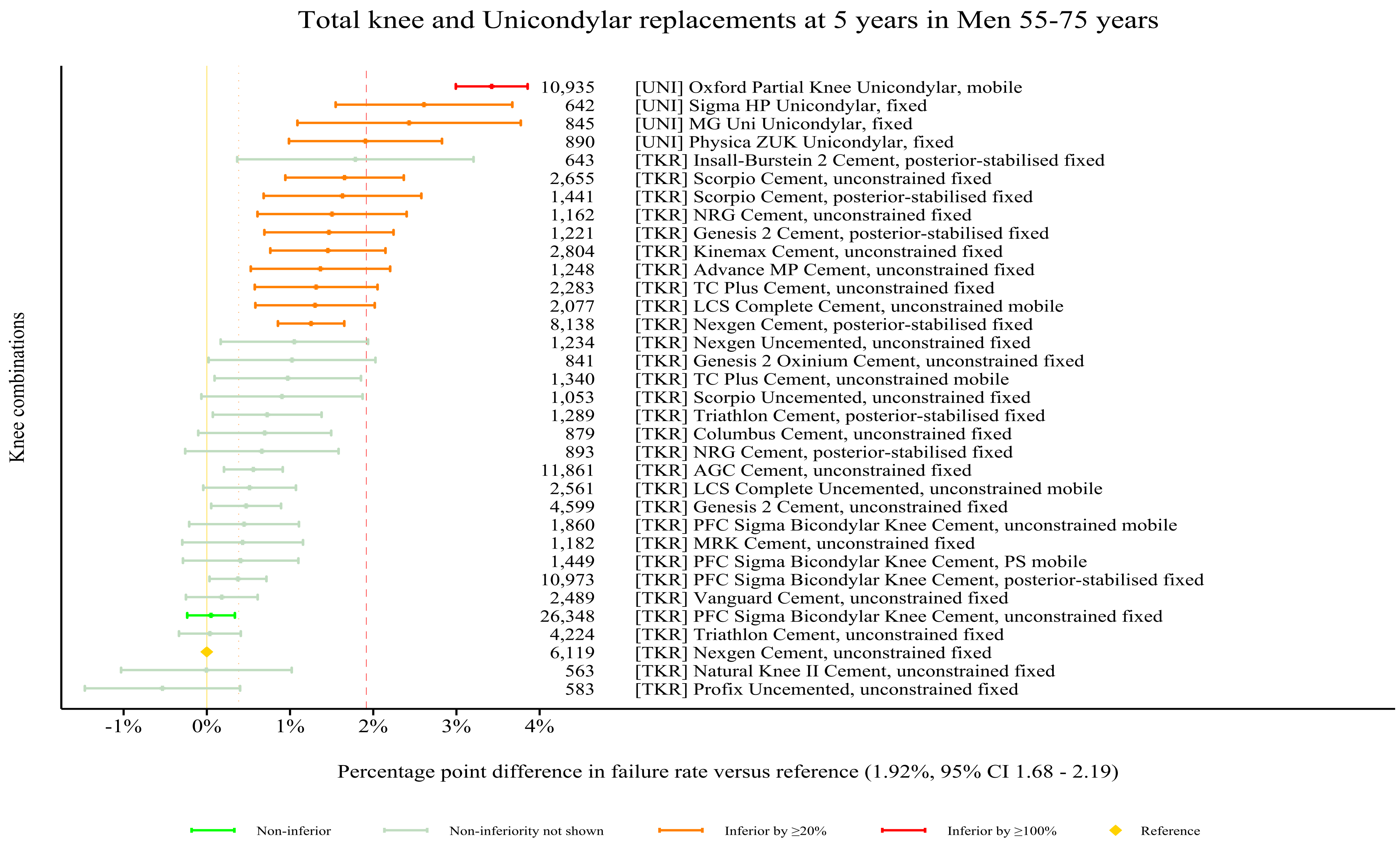

Supplementary Figure 20: Difference in cumulative revision of knee implants compared to a contemporary reference at 10 years in men aged between 55 and 75 years, using all total knee and unicondylar replacements with ≥500 procedures remaining at risk.

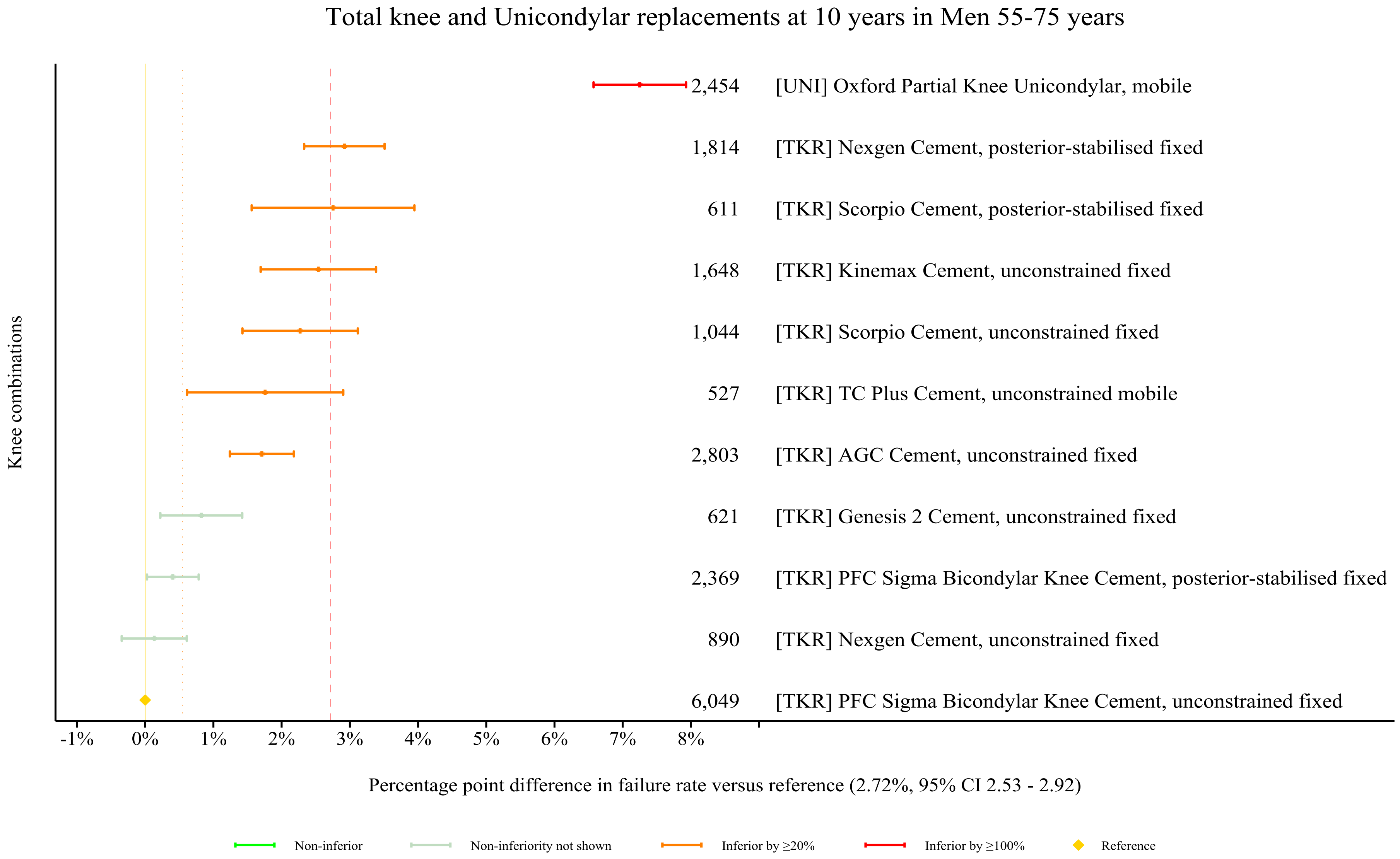

Supplementary Figure 21: Difference in cumulative revision of knee implants compared to a contemporary reference at 3 years in women aged between 55 and 75 years, using all total knee and unicondylar replacements with ≥500 procedures remaining at risk.

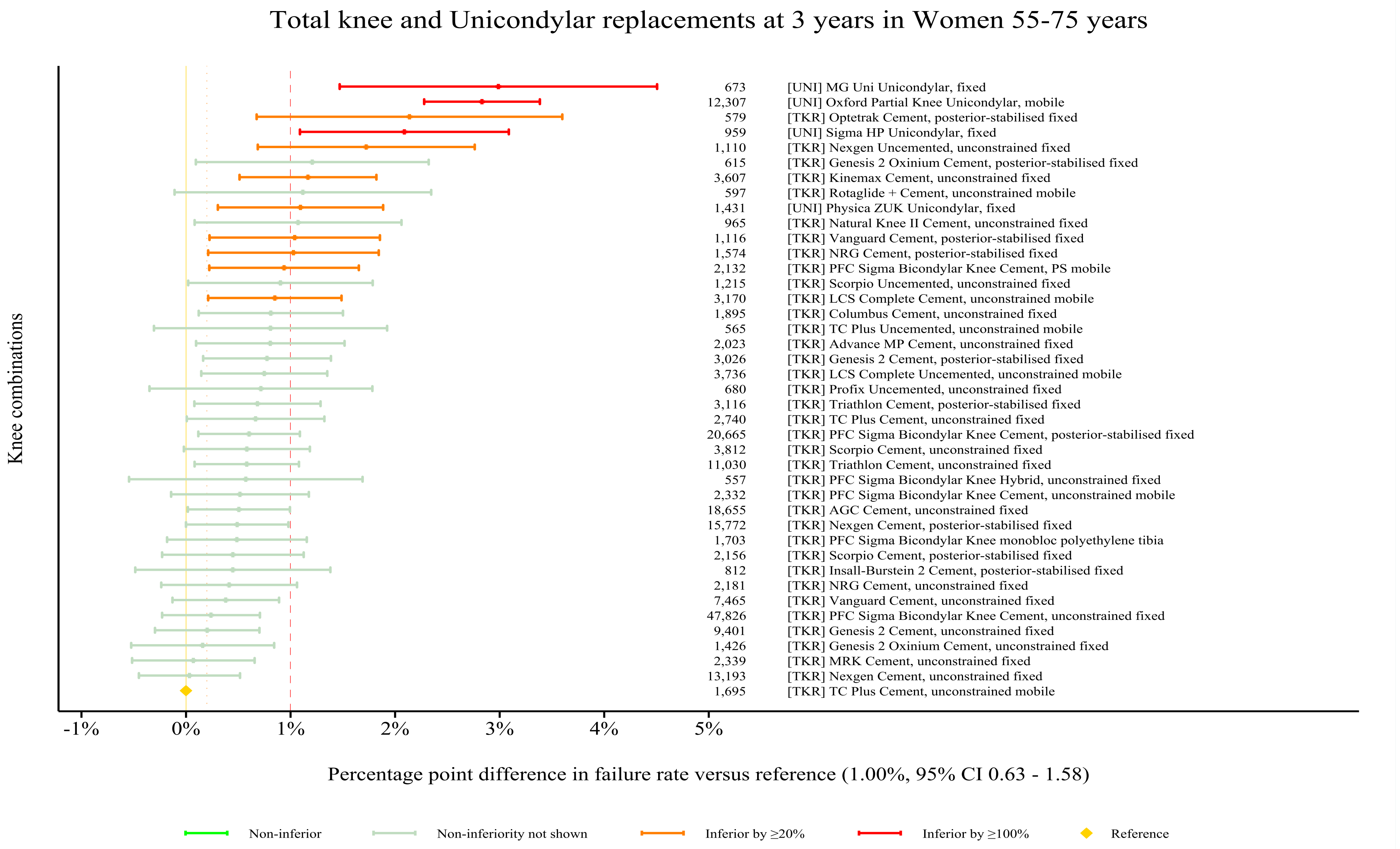

Supplementary Figure 22: Difference in cumulative revision of knee implants compared to a contemporary reference at 5 years in women aged between 55 and 75 years, using all total knee and unicondylar replacements with ≥500 procedures remaining at risk.

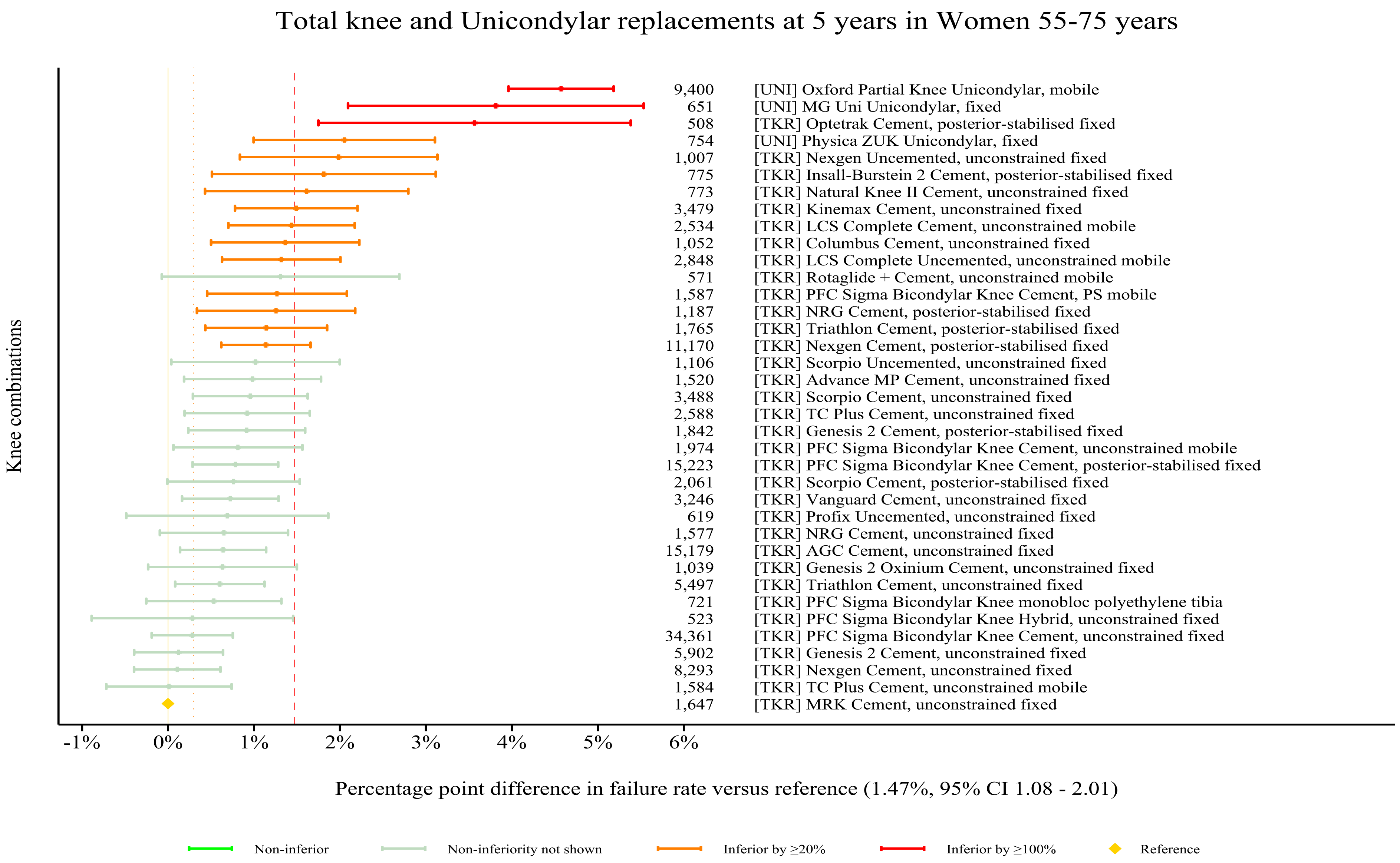

Supplementary Figure 23: Difference in cumulative revision of knee implants compared to a contemporary reference at 10 years in women aged between 55 and 75 years, using all total knee and unicondylar replacements with ≥500 procedures remaining at risk.

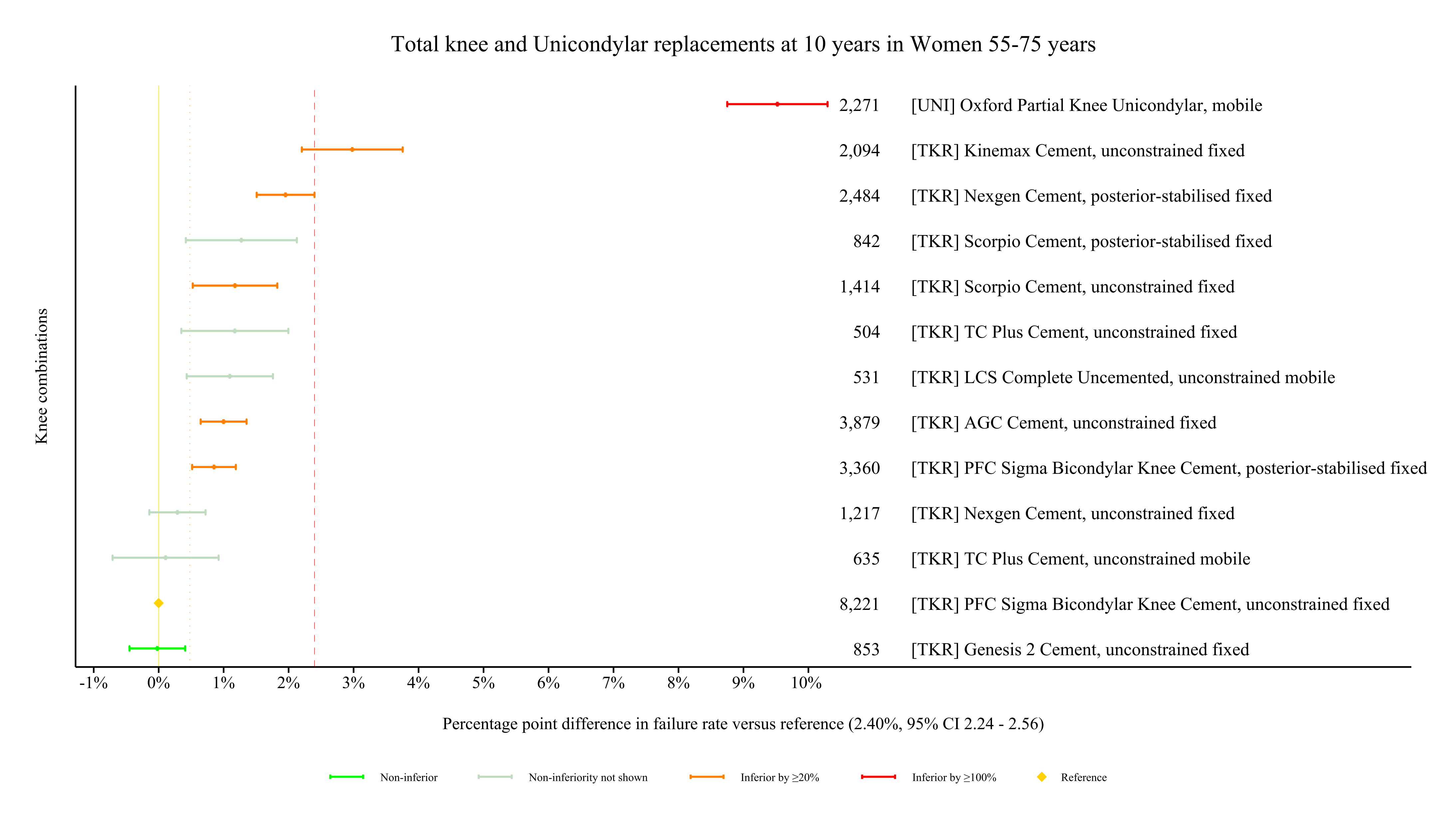

Supplementary Figure 24: Difference in cumulative revision of knee implants compared to a contemporary reference at 3 years in men aged over 75 years, using all total knee and unicondylar replacements with ≥500 procedures remaining at risk.

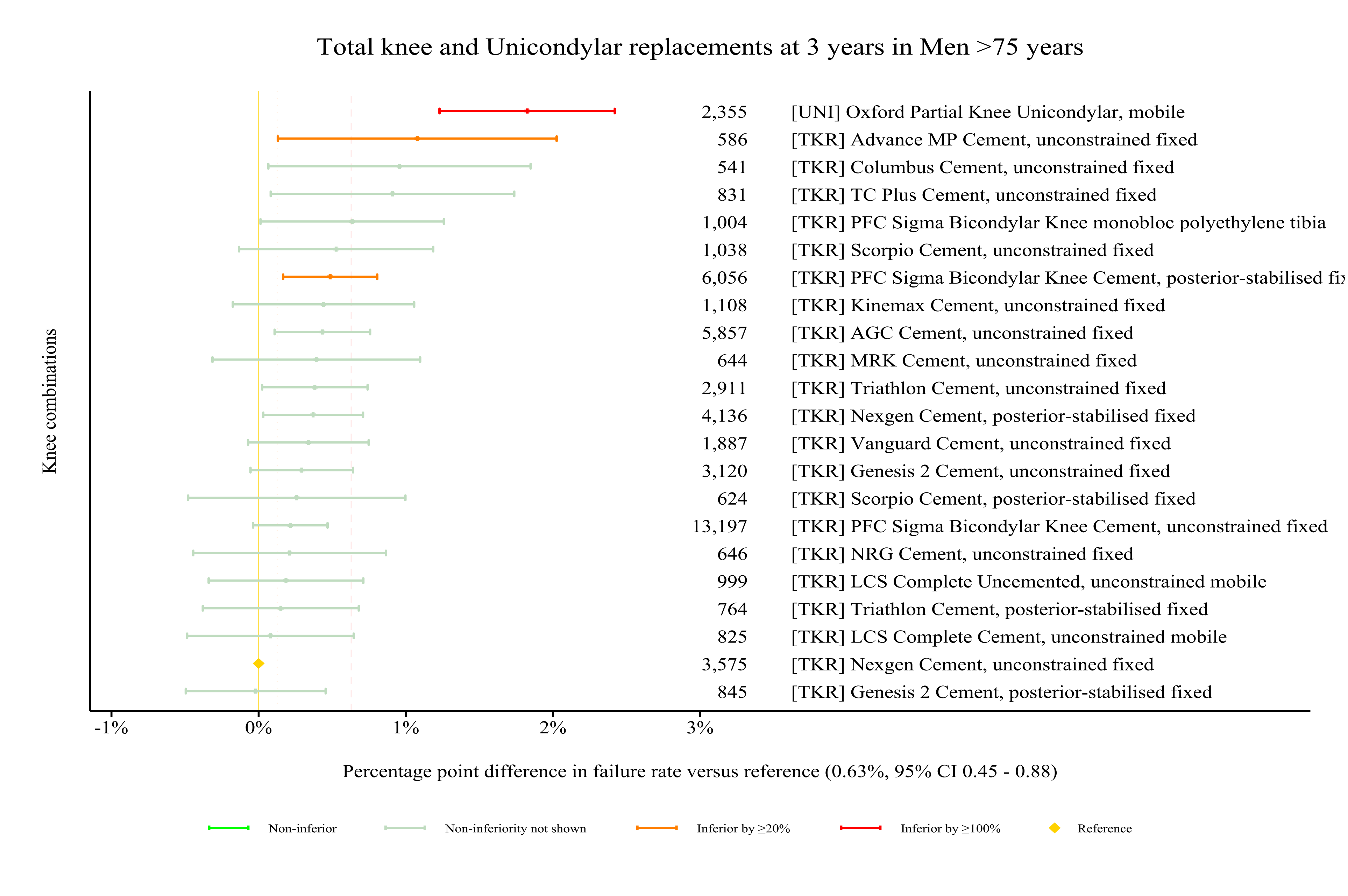

Supplementary Figure 25: Difference in cumulative revision of knee implants compared to a contemporary reference at 5 years in men aged over 75 years, using all total knee and unicondylar replacements with ≥500 procedures remaining at risk.

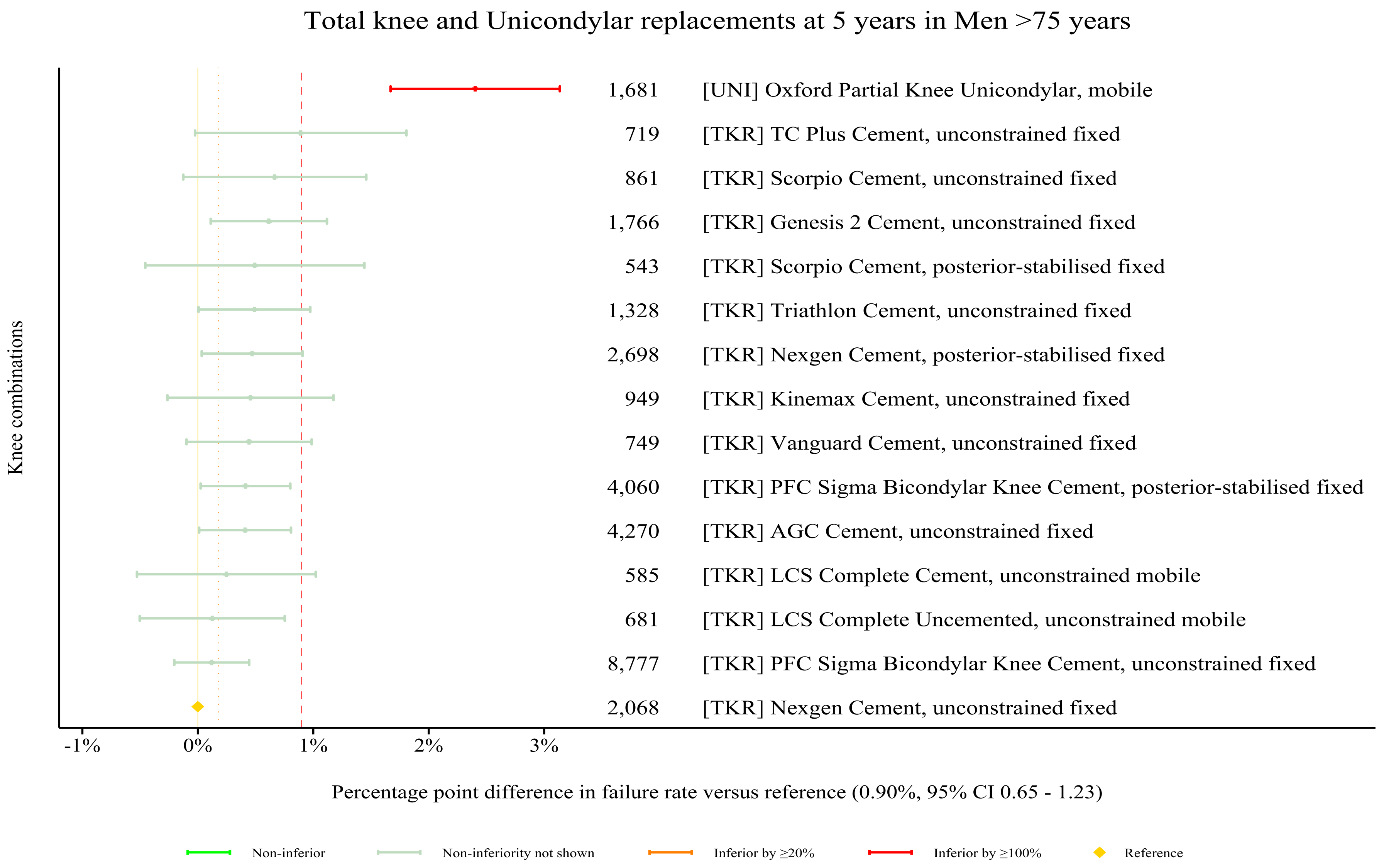

Supplementary Figure 26: Difference in cumulative revision of knee implants compared to a contemporary reference at 7 years in men aged over 75 years, using all total knee and unicondylar replacements with ≥500 procedures remaining at risk.

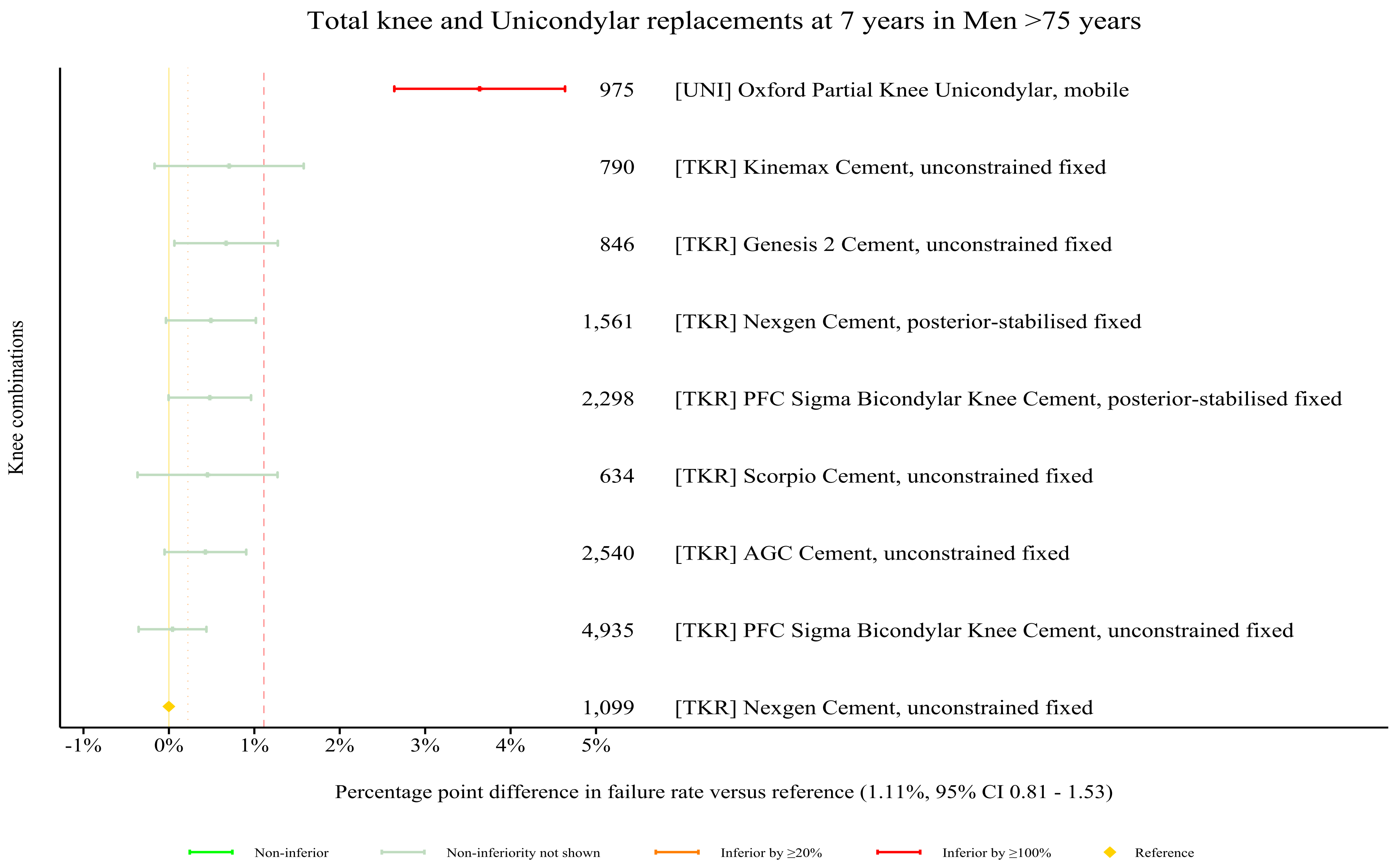

Supplementary Figure 27: Difference in cumulative revision of knee implants compared to a contemporary reference at 10 years in men aged over 75 years, using all total knee and unicondylar replacements with ≥500 procedures remaining at risk.

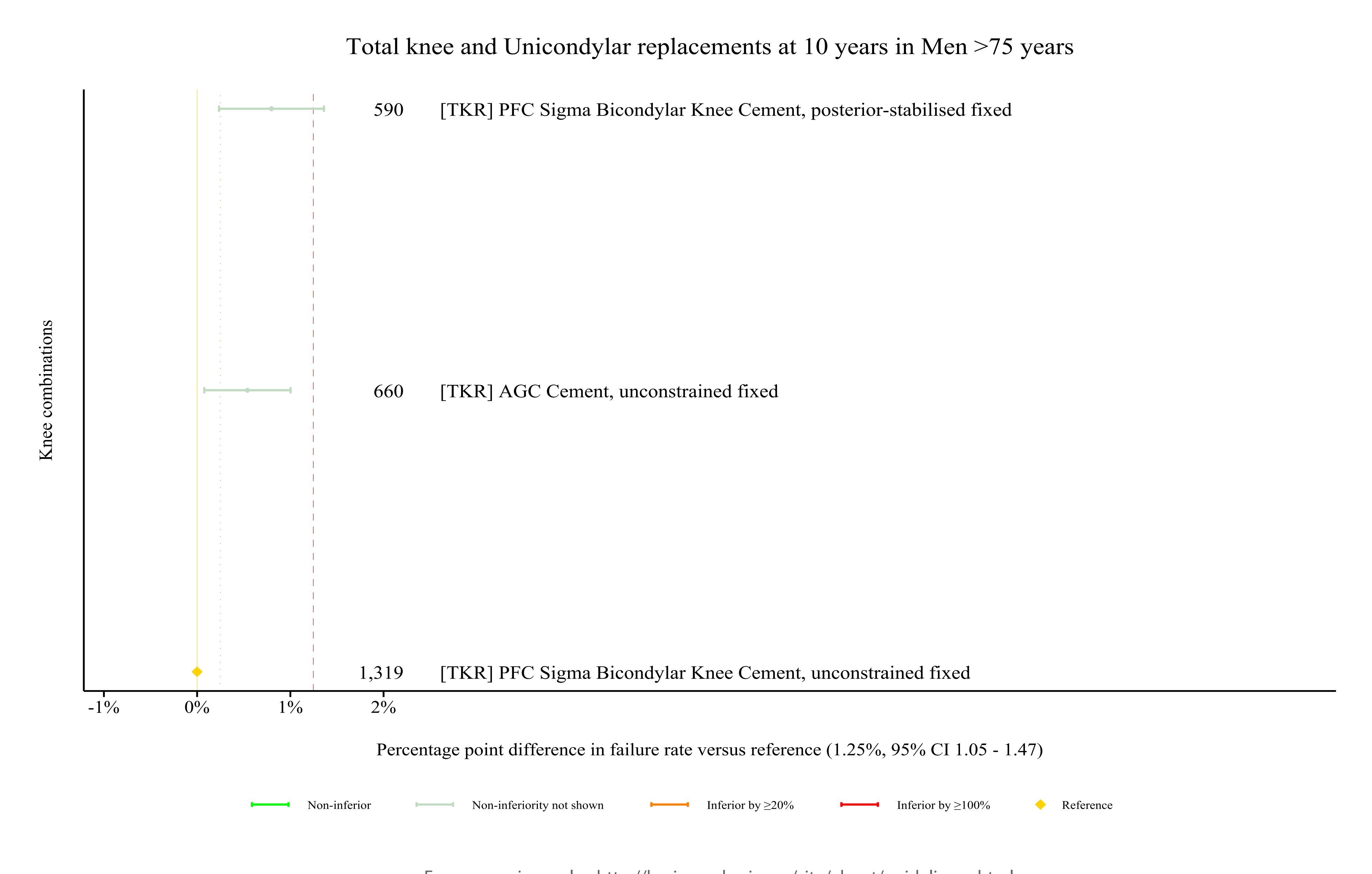

Supplementary Figure 28: Difference in cumulative revision of knee implants compared to a contemporary reference at 3 years in women aged over 75 years, using all total knee and unicondylar replacements with ≥500 procedures remaining at risk.

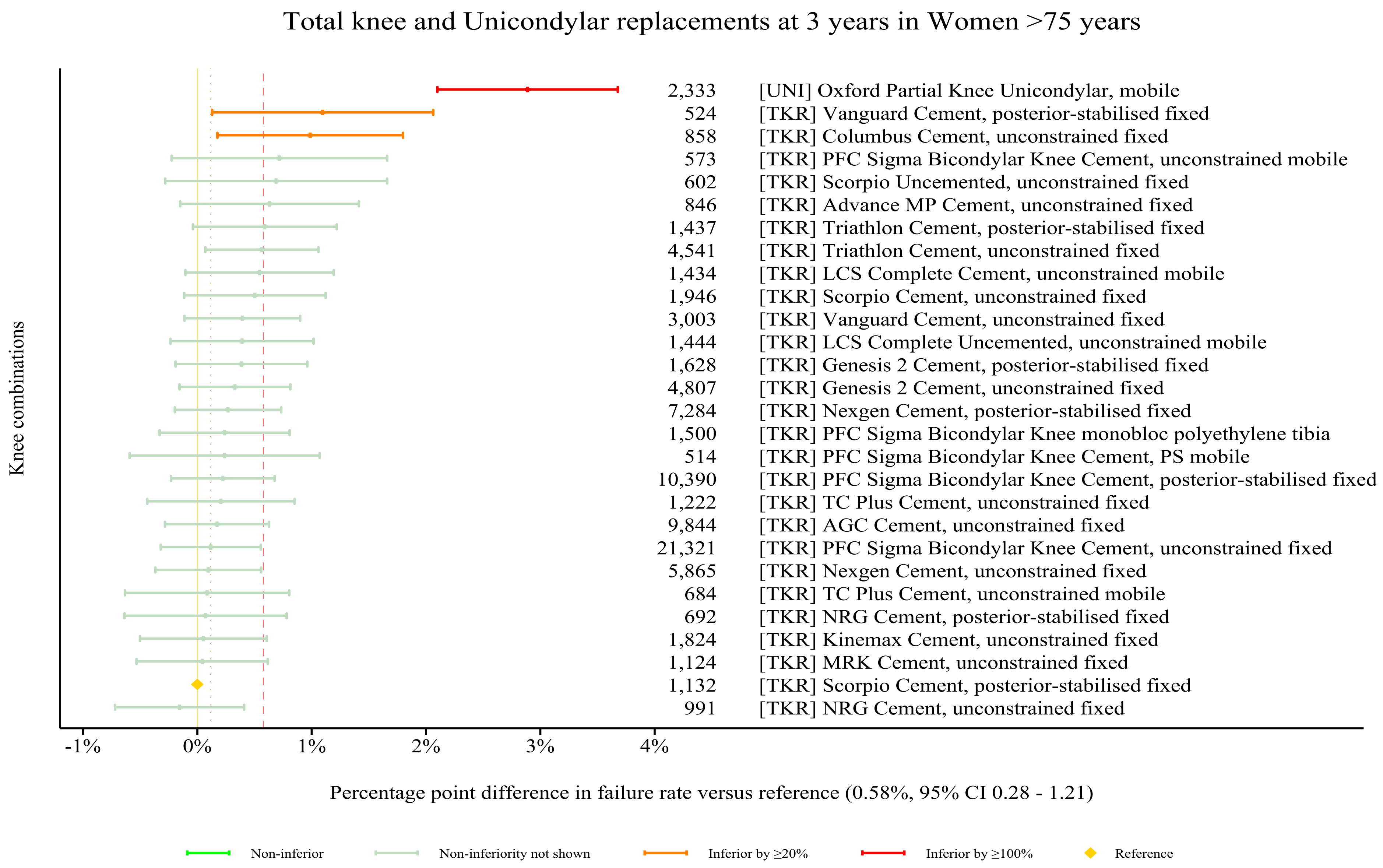

Supplementary Figure 29: Difference in cumulative revision of knee implants compared to a contemporary reference at 5 years in women aged over 75 years, using all total knee and unicondylar replacements with ≥500 procedures remaining at risk.

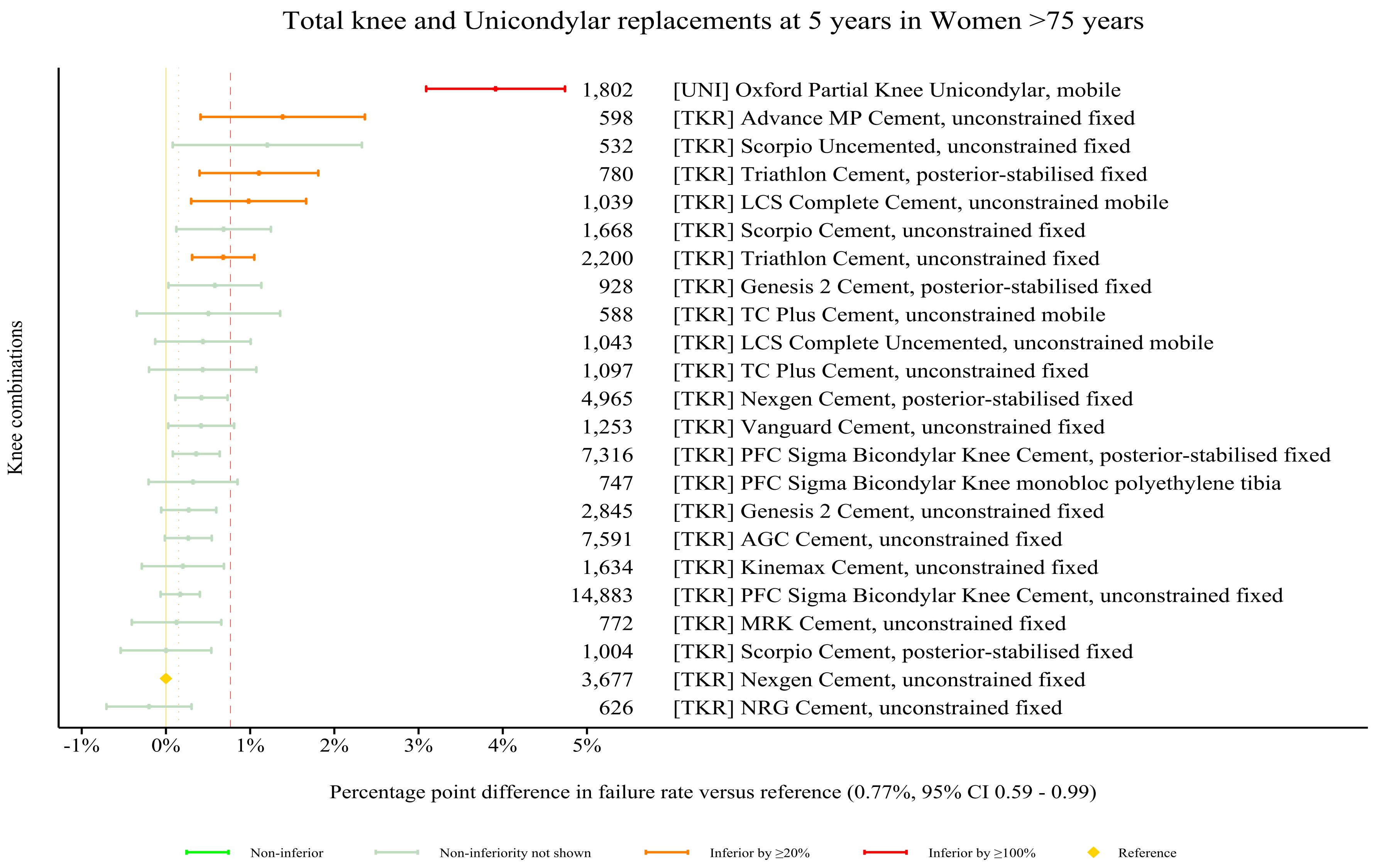

Supplementary Figure 30: Difference in cumulative revision of knee implants compared to a contemporary reference at 7 years in women aged over 75 years, using all total knee and unicondylar replacements with ≥500 procedures remaining at risk.

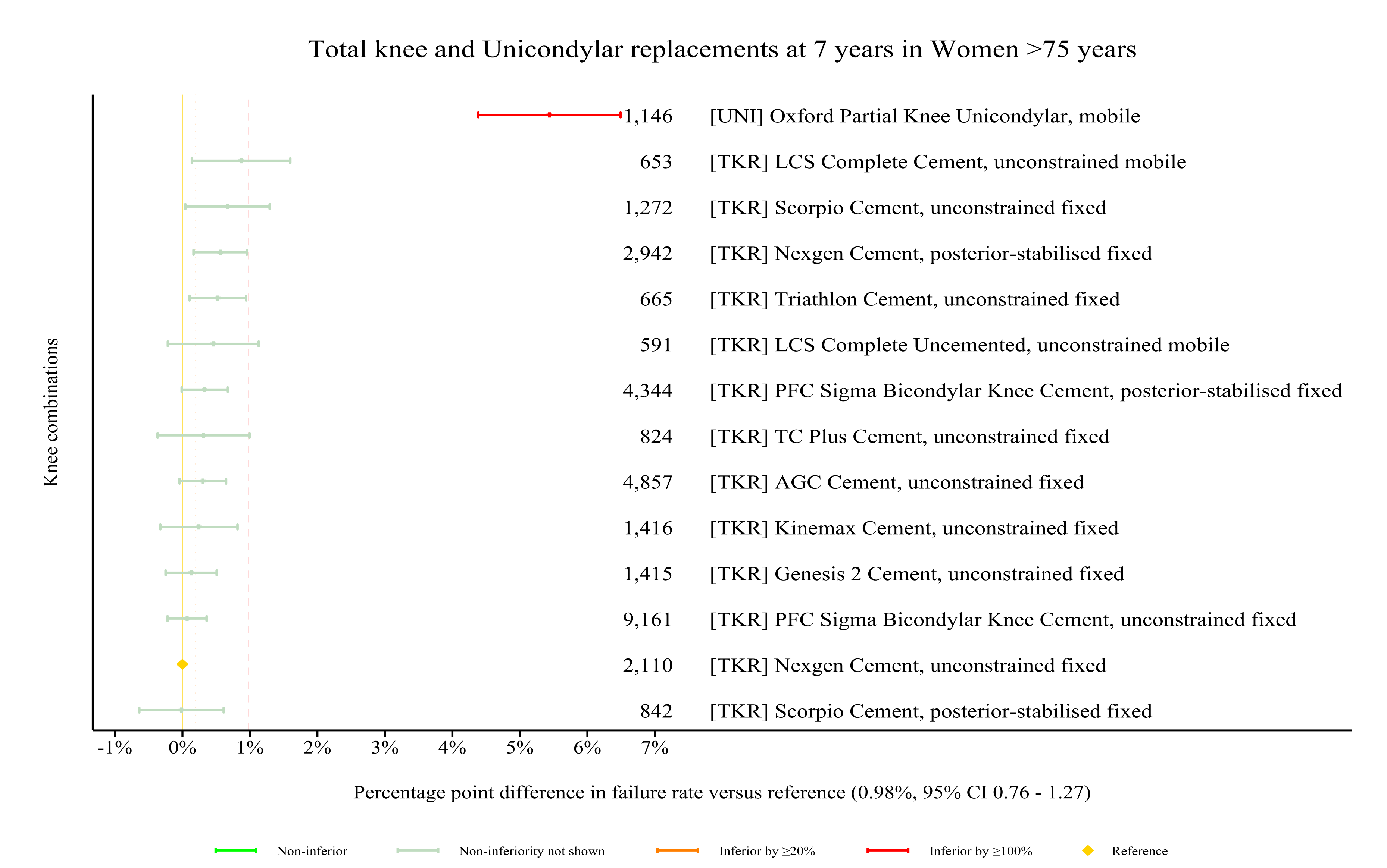

Supplementary Figure 31: Difference in cumulative revision of knee implants compared to a contemporary reference at 10 years in women aged over 75 years, using all total knee and unicondylar replacements with ≥500 procedures remaining at risk.

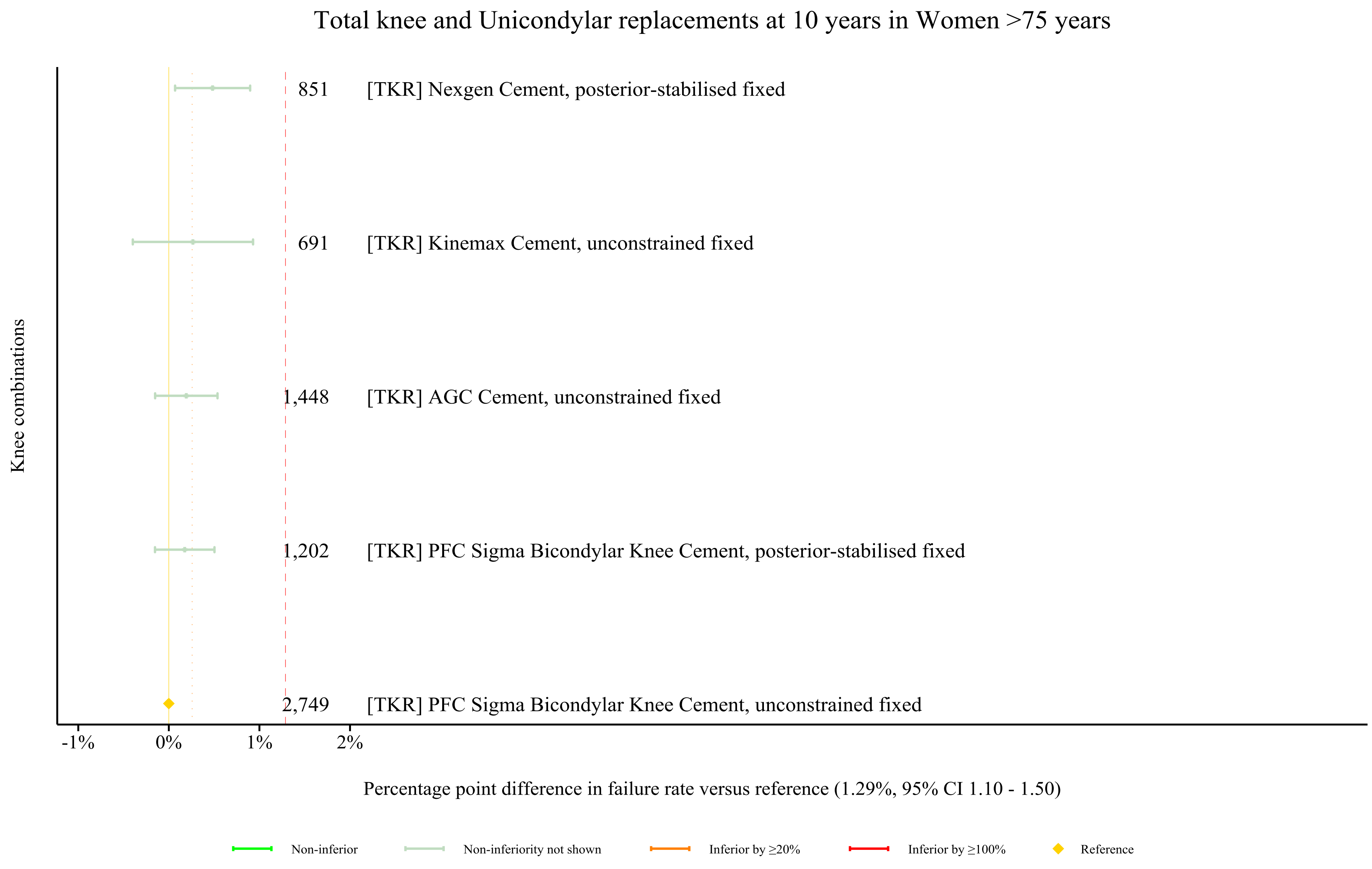

Supplementary table 1: Difference in cumulative percentage revision of knee implants compared to a contemporary benchmark at 3 years post primary for knee replacements with ≥250 procedures remaining at risk

Knee brand, bearing and constraint	Number at risk	Cumulative failure (%)	Difference in failure (%)	95% CI	Equivalence status	p-value
[TKR] NexGen Cement, unconstrained fixed	34,558	1.10	[REFERENCE]			
[TKR] ACS Uncemented, unconstrained fixed	448	3.42	2.32	[0.67 , 3.97]	Inferior by ≥20%	0.003
[TKR] ACS Uncemented, unconstrained mobile	428	3.20	2.10	[0.55 , 3.65]	Inferior by ≥20%	0.004
[TKR] AGC Cement, posterior-stabilised fixed	502	1.90	0.80	[-0.37 , 1.98]	Non-inferiority not shown	0.090
[TKR] AGC Cement, unconstrained fixed	51,355	1.43	0.33	[0.19 , 0.47]	Non-inferiority not shown	<0.001
[TKR] AGC Hybrid, unconstrained fixed	861	2.28	1.18	[0.21 , 2.15]	Non-inferiority not shown	0.008
[TKR] AGC Uncemented, unconstrained fixed	1,081	4.08	2.98	[1.83 , 4.12]	Inferior by ≥100%	<0.001
[TKR] AMK Cement, unconstrained fixed	378	0.48	-0.61	[-1.29 , 0.06]	Non-inferior	0.037
[TKR] Advance MP Cement, unconstrained fixed	5,549	2.04	0.94	[0.59 , 1.29]	Inferior by ≥20%	<0.001
[TKR] Advance MP Stature Cement, unconstrained fixed	829	1.83	0.73	[-0.07 , 1.53]	Non-inferiority not shown	0.037
[TKR] Advance PS Cement, posterior-stabilised fixed	791	2.39	1.29	[0.32 , 2.27]	Inferior by ≥20%	0.005
[TKR] Alpina Hybrid, unconstrained fixed	441	1.94	0.84	[-0.42 , 2.10]	Non-inferiority not shown	0.095
[TKR] Birmingham Knee Replacement Cement, posterior-stabilised mobile	420	1.86	0.76	[-0.30 , 1.81]	Non-inferiority not shown	0.080
[TKR] Columbus Cement, unconstrained fixed	5,128	1.78	0.68	[0.36 , 1.01]	Inferior by ≥20%	<0.001
[TKR] E-Motion Bicondylar Knee Cement, unconstrained mobile	702	3.01	1.92	[0.78 , 3.05]	Inferior by ≥20%	<0.001
[TKR] E-Motion Bicondylar Knee Uncemented, unconstrained mobile	1,393	2.03	0.93	[0.25 , 1.61]	Inferior by ≥20%	0.004
[TKR] FS Cement, unconstrained fixed	741	1.54	0.45	[-0.43 , 1.32]	Non-inferiority not shown	0.158
[TKR] Genesis 2 Cement, posterior-stabilised fixed	7,864	1.75	0.65	[0.38 , 0.91]	Inferior by ≥20%	<0.001
[TKR] Genesis 2 Cement, unconstrained fixed	25,556	1.27	0.17	[0.02 , 0.33]	Non-inferiority not shown	0.014
[TKR] Genesis 2 Cement, unconstrained mobile	887	1.74	0.64	[-0.21 , 1.49]	Non-inferiority not shown	0.070
[TKR] Genesis 2 Oxinium Cement, posterior-stabilised fixed	1,681	2.95	1.85	[1.12 , 2.57]	Inferior by ≥100%	<0.001
[TKR] Genesis 2 Oxinium Cement, unconstrained fixed	3,633	1.94	0.84	[0.42 , 1.27]	Inferior by ≥20%	<0.001
[TKR] Innex Uncemented, unconstrained mobile	358	1.62	0.52	[-0.77 , 1.80]	Non-inferiority not shown	0.216
[TKR] Insall-Burstein 2 Cement, posterior-stabilised fixed	2,226	1.47	0.37	[-0.13 , 0.87]	Non-inferiority not shown	0.072
[TKR] Journey Oxinium Cement, posterior-stabilised fixed	744	4.59	3.49	[2.03 , 4.96]	Inferior by ≥100%	<0.001
[TKR] Kinemax Cement, unconstrained fixed	9,935	1.78	0.68	[0.41 , 0.95]	Inferior by ≥20%	<0.001
[TKR] LCS Cement, unconstrained mobile	611	1.39	0.29	[-0.62 , 1.20]	Non-inferiority not shown	0.264
[TKR] LCS Complete Cement, unconstrained mobile	8,451	1.62	0.52	[0.25 , 0.80]	Inferior by ≥20%	<0.001
[TKR] LCS Complete Hybrid, unconstrained mobile	381	1.68	0.59	[-0.47 , 1.64]	Non-inferiority not shown	0.137

For peer review only

[TKR] LCS Complete Uncemented, unconstrained mobile	10,293	1.75	0.65	[0.40 , 0.91]	Inferior by ≥20%	<0.001
[TKR] LCS Uncemented, unconstrained mobile	1,237	1.87	0.77	[0.02 , 1.52]	Non-inferiority not shown	0.022
[TKR] Legion Cement, unconstrained fixed	395	1.50	0.40	[-0.53 , 1.33]	Non-inferiority not shown	0.202
[TKR] MBK Cement, unconstrained mobile	372	1.30	0.20	[-0.94 , 1.33]	Non-inferiority not shown	0.365
[TKR] MRK Cement, unconstrained fixed	6,170	1.20	0.10	[-0.16 , 0.35]	Non-inferiority not shown	0.231
[TKR] Maxim Cement, posterior-stabilised fixed	582	1.48	0.38	[-0.58 , 1.35]	Non-inferiority not shown	0.220
[TKR] Maxim Cement, unconstrained fixed	1,244	1.48	0.38	[-0.29 , 1.04]	Non-inferiority not shown	0.134
[TKR] NRG Cement, posterior-stabilised fixed	4,141	1.71	0.61	[0.22 , 1.00]	Inferior by ≥20%	0.001
[TKR] NRG Cement, unconstrained fixed	5,860	1.48	0.38	[0.08 , 0.68]	Non-inferiority not shown	0.007
[TKR] Natural Knee II Cement, unconstrained fixed	2,481	1.37	0.27	[-0.18 , 0.73]	Non-inferiority not shown	0.120
[TKR] NexGen Cement, posterior-stabilised mobile	813	2.83	1.73	[0.69 , 2.77]	Inferior by ≥20%	0.001
[TKR] NexGen Cement, posterior-stabilised fixed	40,703	1.61	0.51	[0.36 , 0.65]	Inferior by ≥20%	<0.001
[TKR] NexGen Hinge Type Cement, unconstrained mobile	292	1.65	0.56	[-0.69 , 1.80]	Non-inferiority not shown	0.191
[TKR] NexGen Hybrid, unconstrained fixed	963	1.07	-0.03	[-0.66 , 0.61]	Non-inferiority not shown	0.466
[TKR] NexGen LCCK Cement, constrained condylar	282	2.77	1.67	[0.01 , 3.33]	Non-inferiority not shown	0.024
[TKR] NexGen Uncemented, posterior-stabilised fixed	1,424	1.72	0.62	[-0.00 , 1.24]	Non-inferiority not shown	0.025
[TKR] NexGen Uncemented, unconstrained fixed	3,540	2.55	1.45	[0.95 , 1.96]	Inferior by ≥20%	<0.001
[TKR] NexGen monobloc polyethylene tibia	530	0.67	-0.43	[-1.03 , 0.18]	Non-inferior	0.083
[TKR] Optetrak Cement, posterior-stabilised fixed	1,495	2.66	1.56	[0.76 , 2.36]	Inferior by ≥20%	<0.001
[TKR] Optetrak Cement, unconstrained fixed	360	3.73	2.63	[0.77 , 4.48]	Inferior by ≥20%	0.003
[TKR] PFC Sigma Bicondylar Knee Cement, posterior-stabilised mobile	5,733	2.12	1.02	[0.65 , 1.38]	Inferior by ≥20%	<0.001
[TKR] PFC Sigma Bicondylar Knee Cement, constrained condylar	560	1.73	0.64	[-0.31 , 1.58]	Non-inferiority not shown	0.094
[TKR] PFC Sigma Bicondylar Knee Cement, posterior-stabilised fixed	54,952	1.51	0.41	[0.28 , 0.55]	Inferior by ≥20%	<0.001
[TKR] PFC Sigma Bicondylar Knee Cement, unconstrained fixed	126,134	1.25	0.15	[0.04 , 0.26]	Non-inferiority not shown	0.005
[TKR] PFC Sigma Bicondylar Knee Cement, unconstrained mobile	6,571	1.93	0.83	[0.51 , 1.16]	Inferior by ≥20%	<0.001
[TKR] PFC Sigma Bicondylar Knee Hybrid, unconstrained fixed	1,532	1.18	0.08	[-0.45 , 0.62]	Non-inferiority not shown	0.381
[TKR] PFC Sigma Bicondylar Knee Uncemented, unconstrained mobile	604	1.44	0.34	[-0.51 , 1.20]	Non-inferiority not shown	0.216
[TKR] PFC Sigma Bicondylar Knee monobloc polyethylene tibia	5,365	1.30	0.21	[-0.07 , 0.48]	Non-inferiority not shown	0.070
[TKR] Profix Cement, unconstrained fixed	883	2.05	0.95	[0.03 , 1.86]	Non-inferiority not shown	0.021
[TKR] Profix Oxinium monobloc polyethylene tibia	570	3.03	1.93	[0.55 , 3.32]	Inferior by ≥20%	0.003
[TKR] Profix Uncemented, unconstrained fixed	2,137	1.26	0.16	[-0.32 , 0.63]	Non-inferiority not shown	0.258
[TKR] Profix monobloc polyethylene tibia	651	0.72	-0.37	[-1.01 , 0.27]	Non-inferiority not shown	0.126
[TKR] Rotaglide + Cement, unconstrained mobile	1,576	2.83	1.73	[0.92 , 2.53]	Inferior by ≥20%	<0.001
[TKR] Rotaglide Cement, unconstrained mobile	1,051	2.05	0.96	[0.15 , 1.76]	Non-inferiority not shown	0.010
[TKR] Scorpio Cement, posterior-stabilised mobile	1,289	1.49	0.39	[-0.26 , 1.05]	Non-inferiority not shown	0.121
[TKR] Scorpio Cement, posterior-stabilised fixed	5,673	1.59	0.49	[0.16 , 0.82]	Non-inferiority not shown	0.002

For peer review only

For peer review only - http://bmjopen.bmj.com/site/about/guidelines.xhtml

[TKR] Scorpio Cement, unconstrained fixed	10,083	1.85	0.75	[0.48 , 1.03]	Inferior by ≥20%	<0.001
[TKR] Scorpio Cement, unconstrained mobile	1,094	2.54	1.44	[0.52 , 2.36]	Inferior by ≥20%	0.001
[TKR] Scorpio Hybrid, unconstrained fixed	1,005	1.80	0.70	[-0.11 , 1.50]	Non-inferiority not shown	0.045
[TKR] Scorpio Uncemented, unconstrained fixed	3,530	1.79	0.69	[0.26 , 1.13]	Inferior by ≥20%	0.001
[TKR] TC Plus Cement, unconstrained fixed	7,501	1.89	0.79	[0.47 , 1.10]	Inferior by ≥20%	<0.001
[TKR] TC Plus Cement, unconstrained mobile	4,385	1.44	0.35	[-0.01 , 0.70]	Non-inferiority not shown	0.027
[TKR] TC Plus Uncemented, unconstrained mobile	1,439	1.52	0.42	[-0.17 , 1.00]	Non-inferiority not shown	0.083
[TKR] Triathlon Cement, posterior-stabilised fixed	8,196	1.73	0.63	[0.37 , 0.89]	Inferior by ≥20%	<0.001
[TKR] Triathlon Cement, unconstrained fixed	29,202	1.48	0.38	[0.23 , 0.53]	Inferior by ≥20%	<0.001
[TKR] Triathlon Uncemented, unconstrained fixed	742	2.75	1.66	[0.63 , 2.69]	Inferior by ≥20%	0.001
[TKR] Vanguard Cement, constrained condylar	436	1.08	-0.02	[-0.72 , 0.69]	Non-inferiority not shown	0.479
[TKR] Vanguard Cement, posterior-stabilised fixed	2,988	1.91	0.81	[0.41 , 1.21]	Inferior by ≥20%	<0.001
[TKR] Vanguard Cement, unconstrained fixed	19,392	1.37	0.27	[0.11 , 0.44]	Non-inferiority not shown	0.001
[TKR] balanSys CR cemented Cement, unconstrained fixed	293	1.72	0.63	[-0.75 , 2.00]	Non-inferiority not shown	0.186
[UNI] AMC/Uniglide Unicondylar, fixed	1,070	2.98	1.88	[0.92 , 2.85]	Inferior by ≥20%	<0.001
[UNI] AMC/Uniglide Unicondylar, mobile	1,195	8.47	7.37	[5.91 , 8.84]	Inferior by ≥100%	<0.001
[UNI] EIUS Unicondylar, fixed	314	9.39	8.29	[5.24 , 11.34]	Inferior by ≥100%	<0.001
[UNI] Genesis Uni Oxinium Unicondylar, fixed	439	3.61	2.51	[0.87 , 4.15]	Inferior by ≥20%	0.001
[UNI] MG Uni Unicondylar, fixed	2,206	3.96	2.86	[2.06 , 3.66]	Inferior by ≥100%	<0.001
[UNI] Oxford Partial Knee Unicondylar, fixed	378	3.57	2.47	[0.79 , 4.16]	Inferior by ≥20%	0.002
[UNI] Oxford Partial Knee Unicondylar, mobile	37,382	4.07	2.98	[2.77 , 3.18]	Inferior by ≥100%	<0.001
[UNI] Physica ZUK Unicondylar, fixed	4,708	2.45	1.35	[0.96 , 1.73]	Inferior by ≥20%	<0.001
[UNI] Preservation Unicondylar, fixed	1,105	6.89	5.79	[4.36 , 7.23]	Inferior by ≥100%	<0.001
[UNI] Sigma HP Unicondylar, fixed	3,561	3.56	2.46	[1.95 , 2.97]	Inferior by ≥100%	<0.001
[UNI] Sled Unicondylar, fixed	682	6.23	5.14	[3.44 , 6.83]	Inferior by ≥100%	<0.001
[UNI] Triathlon Uni Unicondylar, fixed	433	5.69	4.59	[2.76 , 6.43]	Inferior by ≥100%	<0.001

For peer review only

Supplementary table 2: Difference in cumulative percentage revision of knee implants compared to a contemporary benchmark at 5 years post primary for knee replacements with ≥250 procedures remaining at risk

Knee brand, bearing and constraint	Number at risk	Cumulative failure (%)	Difference in failure (%)	95% CI	Equivalence status	p-value
[TKR] Profix Uncemented, unconstrained fixed	1,910	1.54	[REFERENCE]			
[TKR] ACS Uncemented, unconstrained fixed	431	3.86	2.32	[0.49 , 4.14]	Inferior by ≥20%	0.006
[TKR] ACS Uncemented, unconstrained mobile	347	3.66	2.12	[0.37 , 3.87]	Inferior by ≥20%	0.009
[TKR] AGC Cement, posterior-stabilised fixed	440	2.71	1.17	[-0.33 , 2.66]	Non-inferiority not shown	0.063
[TKR] AGC Cement, unconstrained fixed	40,694	2.03	0.49	[-0.04 , 1.02]	Non-inferiority not shown	0.035
[TKR] AGC Hybrid, unconstrained fixed	799	2.86	1.32	[0.12 , 2.52]	Non-inferiority not shown	0.016
[TKR] AGC Uncemented, unconstrained fixed	969	5.10	3.56	[2.18 , 4.94]	Inferior by ≥100%	<0.001
[TKR] AMK Cement, unconstrained fixed	350	1.02	-0.52	[-1.64 , 0.60]	Non-inferiority not shown	0.180
[TKR] Advance MP Cement, unconstrained fixed	3,988	2.79	1.25	[0.59 , 1.91]	Inferior by ≥20%	<0.001
[TKR] Advance MP Stature Cement, unconstrained fixed	452	3.20	1.66	[0.36 , 2.96]	Inferior by ≥20%	0.006
[TKR] Advance PS Cement, posterior-stabilised fixed	619	3.19	1.65	[0.38 , 2.91]	Inferior by ≥20%	0.005
[TKR] Alpina Hybrid, unconstrained fixed	390	2.19	0.65	[-0.79 , 2.09]	Non-inferiority not shown	0.189
[TKR] Birmingham Knee Replacement Cement, posterior-stabilised mobile	270	2.76	1.21	[-0.34 , 2.76]	Non-inferiority not shown	0.062
[TKR] Columbus Cement, unconstrained fixed	2,818	2.51	0.97	[0.31 , 1.63]	Inferior by ≥20%	0.002
[TKR] E-Motion Bicondylar Knee Cement, unconstrained mobile	354	3.92	2.38	[0.91 , 3.85]	Inferior by ≥20%	0.001
[TKR] E-Motion Bicondylar Knee Uncemented, unconstrained mobile	1,061	2.85	1.31	[0.32 , 2.29]	Inferior by ≥20%	0.005
[TKR] FS Cement, unconstrained fixed	701	2.09	0.54	[-0.59 , 1.68]	Non-inferiority not shown	0.174
[TKR] Genesis 2 Cement, posterior-stabilised fixed	4,569	2.47	0.93	[0.32 , 1.54]	Inferior by ≥20%	0.001
[TKR] Genesis 2 Cement, unconstrained fixed	15,556	1.75	0.21	[-0.33 , 0.75]	Non-inferiority not shown	0.222
[TKR] Genesis 2 Cement, unconstrained mobile	774	2.31	0.77	[-0.34 , 1.88]	Non-inferiority not shown	0.086
[TKR] Genesis 2 Oxinium Cement, posterior-stabilised fixed	1,030	4.23	2.69	[1.63 , 3.74]	Inferior by ≥100%	<0.001
[TKR] Genesis 2 Oxinium Cement, unconstrained fixed	2,660	3.03	1.49	[0.74 , 2.24]	Inferior by ≥20%	<0.001
[TKR] Innex Uncemented, unconstrained mobile	348	2.45	0.91	[-0.76 , 2.57]	Non-inferiority not shown	0.142
[TKR] Insall-Burstein 2 Cement, posterior-stabilised fixed	2,067	2.74	1.20	[0.35 , 2.05]	Inferior by ≥20%	0.003
[TKR] Journey Oxinium Cement, posterior-stabilised fixed	513	6.26	4.72	[2.93 , 6.52]	Inferior by ≥100%	<0.001
[TKR] Kinemax Cement, unconstrained fixed	9,295	2.70	1.15	[0.55 , 1.76]	Inferior by ≥20%	<0.001
[TKR] LCS Cement, unconstrained mobile	568	1.88	0.34	[-0.83 , 1.52]	Non-inferiority not shown	0.285
[TKR] LCS Complete Cement, unconstrained mobile	6,533	2.73	1.19	[0.57 , 1.81]	Inferior by ≥20%	<0.001
[TKR] LCS Complete Hybrid, unconstrained mobile	268	2.35	0.81	[-0.68 , 2.29]	Non-inferiority not shown	0.143

For peer review only

[TKR] LCS Complete Uncemented, unconstrained mobile	7,677	2.50	0.95	[0.36 , 1.55]	Inferior by ≥20%	0.001
[TKR] LCS Uncemented, unconstrained mobile	1,159	2.44	0.89	[-0.10 , 1.89]	Non-inferiority not shown	0.039
[TKR] MBK Cement, unconstrained mobile	360	2.63	1.09	[-0.60 , 2.78]	Non-inferiority not shown	0.103
[TKR] MRK Cement, unconstrained fixed	4,250	1.64	0.09	[-0.50 , 0.69]	Non-inferiority not shown	0.377
[TKR] Maxim Cement, posterior-stabilised fixed	480	3.12	1.58	[0.06 , 3.09]	Non-inferiority not shown	0.021
[TKR] Maxim Cement, unconstrained fixed	1,109	2.13	0.59	[-0.36 , 1.54]	Non-inferiority not shown	0.111
[TKR] NRG Cement, posterior-stabilised fixed	3,075	2.40	0.86	[0.17 , 1.55]	Non-inferiority not shown	0.007
[TKR] NRG Cement, unconstrained fixed	3,999	2.36	0.82	[0.17 , 1.46]	Non-inferiority not shown	0.006
[TKR] Natural Knee II Cement, unconstrained fixed	1,950	2.21	0.67	[-0.11 , 1.44]	Non-inferiority not shown	0.046
[TKR] NexGen Cement, posterior-stabilised mobile	680	3.60	2.06	[0.75 , 3.37]	Inferior by ≥20%	0.001
[TKR] NexGen Cement, posterior-stabilised fixed	28,335	2.54	0.99	[0.46 , 1.53]	Inferior by ≥20%	<0.001
[TKR] NexGen Cement, unconstrained fixed	21,242	1.60	0.05	[-0.48 , 0.58]	Non-inferiority not shown	0.423
[TKR] NexGen Hybrid, unconstrained fixed	832	1.60	0.06	[-0.87 , 1.00]	Non-inferiority not shown	0.450
[TKR] NexGen Uncemented, posterior-stabilised fixed	1,082	2.20	0.66	[-0.23 , 1.54]	Non-inferiority not shown	0.073
[TKR] NexGen Uncemented, unconstrained fixed	3,059	3.18	1.63	[0.87 , 2.40]	Inferior by ≥20%	<0.001
[TKR] Optetrak Cement, posterior-stabilised fixed	1,278	4.56	3.02	[1.85 , 4.19]	Inferior by ≥100%	<0.001
[TKR] PFC Sigma Bicondylar Knee Cement, posterior-stabilised mobile	4,255	2.79	1.25	[0.59 , 1.91]	Inferior by ≥20%	<0.001
[TKR] PFC Sigma Bicondylar Knee Cement, constrained condylar	366	2.56	1.02	[-0.32 , 2.36]	Non-inferiority not shown	0.068
[TKR] PFC Sigma Bicondylar Knee Cement, posterior-stabilised fixed	39,518	2.09	0.54	[0.01 , 1.07]	Non-inferiority not shown	0.022
[TKR] PFC Sigma Bicondylar Knee Cement, unconstrained fixed	88,618	1.71	0.16	[-0.36 , 0.68]	Non-inferiority not shown	0.270
[TKR] PFC Sigma Bicondylar Knee Cement, unconstrained mobile	5,415	2.73	1.19	[0.54 , 1.83]	Inferior by ≥20%	<0.001
[TKR] PFC Sigma Bicondylar Knee Hybrid, unconstrained fixed	1,389	1.79	0.25	[-0.59 , 1.08]	Non-inferiority not shown	0.280
[TKR] PFC Sigma Bicondylar Knee Uncemented, unconstrained mobile	450	1.78	0.24	[-0.86 , 1.34]	Non-inferiority not shown	0.335
[TKR] PFC Sigma Bicondylar Knee monobloc polyethylene tibia	2,383	1.65	0.10	[-0.50 , 0.71]	Non-inferiority not shown	0.370
[TKR] Profix Cement, unconstrained fixed	813	3.20	1.65	[0.40 , 2.91]	Inferior by ≥20%	0.005
[TKR] Profix Oxinium monobloc polyethylene tibia	553	3.55	2.01	[0.43 , 3.58]	Inferior by ≥20%	0.006
[TKR] Profix monobloc polyethylene tibia	600	1.35	-0.19	[-1.21 , 0.83]	Non-inferiority not shown	0.356
[TKR] Rotaglide + Cement, unconstrained mobile	1,454	3.65	2.10	[1.06 , 3.15]	Inferior by ≥20%	<0.001
[TKR] Rotaglide Cement, unconstrained mobile	777	3.29	1.75	[0.57 , 2.92]	Inferior by ≥20%	0.002
[TKR] Scorpio Cement, posterior-stabilised mobile	1,178	2.19	0.65	[-0.30 , 1.59]	Non-inferiority not shown	0.089
[TKR] Scorpio Cement, posterior-stabilised fixed	5,265	2.37	0.83	[0.18 , 1.48]	Non-inferiority not shown	0.006
[TKR] Scorpio Cement, unconstrained fixed	9,046	2.61	1.06	[0.46 , 1.66]	Inferior by ≥20%	<0.001
[TKR] Scorpio Cement, unconstrained mobile	1,036	3.63	2.08	[0.88 , 3.29]	Inferior by ≥20%	<0.001
[TKR] Scorpio Hybrid, unconstrained fixed	938	2.51	0.96	[-0.12 , 2.05]	Non-inferiority not shown	0.040
[TKR] Scorpio Uncemented, unconstrained fixed	3,140	2.49	0.95	[0.22 , 1.67]	Non-inferiority not shown	0.005
[TKR] TC Plus Cement, unconstrained fixed	6,952	2.56	1.01	[0.39 , 1.64]	Inferior by ≥20%	0.001

For peer review only

[TKR] TC Plus Cement, unconstrained mobile	3,999	2.00	0.46	[-0.20 , 1.11]	Non-inferiority not shown	0.085
[TKR] TC Plus Uncemented, unconstrained mobile	1,145	2.18	0.63	[-0.25 , 1.52]	Non-inferiority not shown	0.081
[TKR] Triathlon Cement, posterior-stabilised fixed	4,605	2.53	0.98	[0.38 , 1.59]	Inferior by ≥20%	0.001
[TKR] Triathlon Cement, unconstrained fixed	14,360	2.01	0.47	[-0.07 , 1.00]	Non-inferiority not shown	0.044
[TKR] Triathlon Uncemented, unconstrained fixed	418	3.39	1.85	[0.54 , 3.15]	Inferior by ≥20%	0.003
[TKR] Vanguard Cement, posterior-stabilised fixed	1,135	2.75	1.21	[0.45 , 1.97]	Inferior by ≥20%	0.001
[TKR] Vanguard Cement, unconstrained fixed	8,329	2.02	0.48	[-0.07 , 1.02]	Non-inferiority not shown	0.045
[UNI] AMC/Uniglide Unicondylar, fixed	756	4.41	2.87	[1.55 , 4.18]	Inferior by ≥100%	<0.001
[UNI] AMC/Uniglide Unicondylar, mobile	997	10.16	8.62	[6.93 , 10.30]	Inferior by ≥100%	<0.001
[UNI] EIUS Unicondylar, fixed	289	14.92	13.38	[9.60 , 17.16]	Inferior by ≥100%	<0.001
[UNI] Genesis Uni Oxinium Unicondylar, fixed	330	7.05	5.51	[3.07 , 7.94]	Inferior by ≥100%	<0.001
[UNI] MG Uni Unicondylar, fixed	2,082	5.95	4.41	[3.31 , 5.51]	Inferior by ≥100%	<0.001
[UNI] Oxford Partial Knee Unicondylar, fixed	284	4.65	3.10	[1.07 , 5.13]	Inferior by ≥20%	0.001
[UNI] Oxford Partial Knee Unicondylar, mobile	28,136	6.13	4.59	[4.02 , 5.15]	Inferior by ≥100%	<0.001
[UNI] Physica ZUK Unicondylar, fixed	2,392	3.94	2.40	[1.65 , 3.15]	Inferior by ≥100%	<0.001
[UNI] Preservation Unicondylar, fixed	1,045	10.21	8.67	[6.87 , 10.46]	Inferior by ≥100%	<0.001
[UNI] Sigma HP Unicondylar, fixed	1,634	5.01	3.47	[2.61 , 4.32]	Inferior by ≥100%	<0.001
[UNI] Sled Unicondylar, fixed	585	9.83	8.29	[6.09 , 10.48]	Inferior by ≥100%	<0.001

For peer review only

Supplementary table 3: Difference in cumulative percentage revision of knee implants compared to a contemporary benchmark at 7 years post primary for knee replacements with ≥250 procedures remaining at risk

Knee brand, bearing and constraint	Number at risk	Cumulative failure (%)	Difference in failure (%)	95% CI	Equivalence status	p-value
[TKR] Profix Uncemented, unconstrained fixed	1,501	1.77	[REFERENCE]			
[TKR] ACS Uncemented, unconstrained fixed	389	5.01	3.24	[1.16 , 5.31]	Inferior by ≥20%	0.001
[TKR] AGC Cement, posterior-stabilised fixed	355	3.97	2.19	[0.34 , 4.05]	Non-inferiority not shown	0.010
[TKR] AGC Cement, unconstrained fixed	27,288	2.56	0.79	[0.21 , 1.37]	Non-inferiority not shown	0.004
[TKR] AGC Hybrid, unconstrained fixed	541	3.25	1.48	[0.19 , 2.78]	Non-inferiority not shown	0.013
[TKR] AGC Uncemented, unconstrained fixed	710	5.97	4.20	[2.69 , 5.71]	Inferior by ≥100%	<0.001
[TKR] AMK Cement, unconstrained fixed	318	1.02	-0.75	[-1.90 , 0.39]	Non-inferiority not shown	0.098
[TKR] Advance MP Cement, unconstrained fixed	2,588	3.51	1.74	[0.99 , 2.49]	Inferior by ≥20%	<0.001
[TKR] Advance PS Cement, posterior-stabilised fixed	425	4.29	2.52	[0.97 , 4.07]	Inferior by ≥20%	0.001
[TKR] Alpina Hybrid, unconstrained fixed	317	3.84	2.07	[0.13 , 4.01]	Non-inferiority not shown	0.018
[TKR] Columbus Cement, unconstrained fixed	1,482	2.79	1.02	[0.29 , 1.75]	Non-inferiority not shown	0.003
[TKR] E-Motion Bicondylar Knee Uncemented, unconstrained mobile	732	3.86	2.09	[0.89 , 3.29]	Inferior by ≥20%	<0.001
[TKR] FS Cement, unconstrained fixed	643	2.66	0.89	[-0.39 , 2.17]	Non-inferiority not shown	0.087
[TKR] Genesis 2 Cement, posterior-stabilised fixed	2,275	2.96	1.19	[0.50 , 1.88]	Inferior by ≥20%	<0.001
[TKR] Genesis 2 Cement, unconstrained fixed	8,056	2.17	0.39	[-0.20 , 0.99]	Non-inferiority not shown	0.097
[TKR] Genesis 2 Cement, unconstrained mobile	549	2.90	1.12	[-0.14 , 2.38]	Non-inferiority not shown	0.040
[TKR] Genesis 2 Oxinium Cement, posterior-stabilised fixed	500	5.81	4.04	[2.64 , 5.44]	Inferior by ≥100%	<0.001
[TKR] Genesis 2 Oxinium Cement, unconstrained fixed	1,664	3.51	1.74	[0.90 , 2.57]	Inferior by ≥20%	<0.001
[TKR] Innex Uncemented, unconstrained mobile	314	2.45	0.68	[-1.00 , 2.36]	Non-inferiority not shown	0.214
[TKR] Insall-Burstein 2 Cement, posterior-stabilised fixed	1,880	3.43	1.65	[0.71 , 2.60]	Inferior by ≥20%	<0.001
[TKR] Journey Oxinium Cement, posterior-stabilised fixed	334	6.26	4.49	[2.68 , 6.30]	Inferior by ≥100%	<0.001
[TKR] Kinemax Cement, unconstrained fixed	8,560	3.53	1.76	[1.09 , 2.43]	Inferior by ≥20%	<0.001
[TKR] LCS Cement, unconstrained mobile	514	2.61	0.84	[-0.55 , 2.23]	Non-inferiority not shown	0.117
[TKR] LCS Complete Cement, unconstrained mobile	4,216	3.44	1.67	[0.98 , 2.36]	Inferior by ≥20%	<0.001
[TKR] LCS Complete Uncemented, unconstrained mobile	4,724	2.92	1.15	[0.50 , 1.80]	Inferior by ≥20%	<0.001
[TKR] LCS Uncemented, unconstrained mobile	1,092	2.44	0.67	[-0.35 , 1.68]	Non-inferiority not shown	0.100
[TKR] MBK Cement, unconstrained mobile	347	3.18	1.41	[-0.45 , 3.26]	Non-inferiority not shown	0.069
[TKR] MRK Cement, unconstrained fixed	2,775	2.28	0.51	[-0.17 , 1.19]	Non-inferiority not shown	0.072
[TKR] Maxim Cement, posterior-stabilised fixed	353	3.60	1.83	[0.16 , 3.49]	Non-inferiority not shown	0.016

For peer review only

[TKR] Maxim Cement, unconstrained fixed	881	3.05	1.28	[0.14 , 2.42]	Non-inferiority not shown	0.014
[TKR] NRG Cement, posterior-stabilised fixed	1,438	2.86	1.09	[0.32 , 1.86]	Non-inferiority not shown	0.003
[TKR] NRG Cement, unconstrained fixed	2,207	2.83	1.06	[0.33 , 1.78]	Non-inferiority not shown	0.002
[TKR] Natural Knee II Cement, unconstrained fixed	1,437	3.20	1.42	[0.50 , 2.35]	Inferior by ≥20%	0.001
[TKR] Nexgen Cement, PS mobile	551	5.12	3.35	[1.74 , 4.96]	Inferior by ≥20%	<0.001
[TKR] Nexgen Cement, posterior-stabilised fixed	17,857	3.31	1.54	[0.95 , 2.13]	Inferior by ≥20%	<0.001
[TKR] Nexgen Cement, unconstrained fixed	12,329	2.12	0.35	[-0.24 , 0.93]	Non-inferiority not shown	0.122
[TKR] Nexgen Hybrid, unconstrained fixed	574	2.15	0.38	[-0.72 , 1.48]	Non-inferiority not shown	0.250
[TKR] Nexgen Uncemented, ps fixed	784	2.54	0.77	[-0.22 , 1.75]	Non-inferiority not shown	0.064
[TKR] Nexgen Uncemented, unconstrained fixed	2,528	3.58	1.81	[0.98 , 2.64]	Inferior by ≥20%	<0.001
[TKR] Optetrak Cement, posterior-stabilised fixed	809	5.43	3.66	[2.37 , 4.96]	Inferior by ≥100%	<0.001
[TKR] PFC Sigma Bicondylar Knee Cement, PS mobile	2,686	3.36	1.59	[0.85 , 2.32]	Inferior by ≥20%	<0.001
[TKR] PFC Sigma Bicondylar Knee Cement, posterior-stabilised fixed	24,658	2.44	0.67	[0.10 , 1.25]	Non-inferiority not shown	0.011
[TKR] PFC Sigma Bicondylar Knee Cement, unconstrained fixed	55,631	2.00	0.23	[-0.34 , 0.80]	Non-inferiority not shown	0.212
[TKR] PFC Sigma Bicondylar Knee Cement, unconstrained mobile	3,818	3.15	1.38	[0.67 , 2.08]	Inferior by ≥20%	<0.001
[TKR] PFC Sigma Bicondylar Knee Hybrid, unconstrained fixed	1,174	1.86	0.09	[-0.78 , 0.97]	Non-inferiority not shown	0.418
[TKR] PFC Sigma Bicondylar Knee Uncemented, unconstrained mobile	258	2.02	0.25	[-0.96 , 1.46]	Non-inferiority not shown	0.344
[TKR] PFC Sigma Bicondylar Knee monobloc polyethylene tibia	810	1.92	0.15	[-0.55 , 0.85]	Non-inferiority not shown	0.340
[TKR] Profix Cement, unconstrained fixed	693	4.20	2.43	[0.99 , 3.87]	Inferior by ≥20%	<0.001
[TKR] Profix Oxinium monobloc polyethylene tibia	471	4.10	2.32	[0.62 , 4.03]	Inferior by ≥20%	0.004
[TKR] Profix monobloc polyethylene tibia	511	1.52	-0.25	[-1.34 , 0.84]	Non-inferiority not shown	0.325
[TKR] Rotaglide + Cement, unconstrained mobile	1,222	4.22	2.45	[1.32 , 3.59]	Inferior by ≥20%	<0.001
[TKR] Rotaglide Cement, unconstrained mobile	488	3.71	1.94	[0.66 , 3.22]	Inferior by ≥20%	0.002
[TKR] Scorpio Cement, PS mobile	1,013	2.54	0.77	[-0.26 , 1.79]	Non-inferiority not shown	0.071
[TKR] Scorpio Cement, posterior-stabilised fixed	4,675	3.07	1.30	[0.58 , 2.02]	Inferior by ≥20%	<0.001
[TKR] Scorpio Cement, unconstrained fixed	7,331	3.14	1.37	[0.71 , 2.03]	Inferior by ≥20%	<0.001
[TKR] Scorpio Cement, unconstrained mobile	950	4.48	2.71	[1.37 , 4.04]	Inferior by ≥20%	<0.001
[TKR] Scorpio Hybrid, unconstrained fixed	823	3.28	1.50	[0.27 , 2.74]	Non-inferiority not shown	0.009
[TKR] Scorpio Uncemented, unconstrained fixed	2,211	3.11	1.33	[0.52 , 2.14]	Inferior by ≥20%	0.001
[TKR] TC Plus Cement, unconstrained fixed	5,337	2.98	1.20	[0.52 , 1.88]	Inferior by ≥20%	<0.001
[TKR] TC Plus Cement, unconstrained mobile	3,267	2.45	0.68	[-0.05 , 1.40]	Non-inferiority not shown	0.033
[TKR] TC Plus Uncemented, unconstrained mobile	783	2.47	0.70	[-0.27 , 1.67]	Non-inferiority not shown	0.079
[TKR] Triathlon Cement, posterior-stabilised fixed	1,580	2.87	1.10	[0.42 , 1.78]	Inferior by ≥20%	0.001
[TKR] Triathlon Cement, unconstrained fixed	4,819	2.50	0.73	[0.13 , 1.33]	Non-inferiority not shown	0.009
[TKR] Vanguard Cement, posterior-stabilised fixed	360	3.58	1.81	[0.76 , 2.86]	Inferior by ≥20%	<0.001

For peer review only

[TKR] Vanguard Cement, unconstrained fixed	2,491	2.36	0.59	[-0.03 , 1.20]	Non-inferiority not shown	0.031
[UNI] AMC/Uniglide Unicondylar, fixed	401	6.44	4.67	[2.95 , 6.38]	Inferior by ≥100%	<0.001
[UNI] AMC/Uniglide Unicondylar, mobile	687	12.42	10.64	[8.74 , 12.55]	Inferior by ≥100%	<0.001
[UNI] EIUS Unicondylar, fixed	280	16.41	14.64	[10.70 , 18.58]	Inferior by ≥100%	<0.001
[UNI] MG Uni Unicondylar, fixed	1,829	7.55	5.78	[4.55 , 7.01]	Inferior by ≥100%	<0.001
[UNI] Oxford Partial Knee Unicondylar, mobile	18,623	8.09	6.32	[5.69 , 6.95]	Inferior by ≥100%	<0.001
[UNI] Physica ZUK Unicondylar, fixed	1,090	5.31	3.54	[2.59 , 4.48]	Inferior by ≥100%	<0.001
[UNI] Preservation Unicondylar, fixed	940	12.95	11.18	[9.18 , 13.18]	Inferior by ≥100%	<0.001
[UNI] Sled Unicondylar, fixed	476	12.39	10.62	[8.12 , 13.12]	Inferior by ≥100%	<0.001

For peer review only

Supplementary table 4: Difference in cumulative percentage revision of knee implants compared to a contemporary benchmark at 10 years post primary for knee replacements with ≥250 procedures remaining at risk

Knee brand, bearing and constraint	Number at risk	Cumulative failure (%)	Difference in failure (%)	95% CI	Equivalence status	p-value
[TKR] PFC Sigma Bicondylar Knee Cement, unconstrained fixed	19,284	2.37	[REFERENCE]			
[TKR] AGC Cement, unconstrained fixed	9,260	3.44	1.07	[0.85 , 1.29]	Inferior by ≥20%	<0.001
[TKR] AGC Uncemented, unconstrained fixed	253	7.76	5.39	[3.55 , 7.24]	Inferior by ≥100%	<0.001
[TKR] AMK Cement, unconstrained fixed	270	1.35	-1.02	[-2.21 , 0.17]	Non-inferior	0.047
[TKR] Advance MP Cement, unconstrained fixed	722	4.25	1.88	[1.18 , 2.59]	Inferior by ≥20%	<0.001
[TKR] Genesis 2 Cement, posterior-stabilised fixed	324	3.85	1.48	[0.66 , 2.30]	Inferior by ≥20%	<0.001
[TKR] Genesis 2 Cement, unconstrained fixed	1,982	2.60	0.23	[-0.05 , 0.52]	Non-inferiority not shown	0.052
[TKR] Genesis 2 Oxinium Cement, unconstrained fixed	486	4.95	2.58	[1.63 , 3.53]	Inferior by ≥20%	<0.001
[TKR] Insall-Burstein 2 Cement, posterior-stabilised fixed	1,125	4.95	2.58	[1.62 , 3.54]	Inferior by ≥20%	<0.001
[TKR] Kinemax Cement, unconstrained fixed	5,049	4.70	2.33	[1.88 , 2.78]	Inferior by ≥20%	<0.001
[TKR] LCS Cement, unconstrained mobile	442	3.62	1.25	[-0.28 , 2.79]	Non-inferiority not shown	0.055
[TKR] LCS Complete Cement, unconstrained mobile	781	4.28	1.91	[1.35 , 2.47]	Inferior by ≥20%	<0.001
[TKR] LCS Complete Uncemented, unconstrained mobile	1,340	3.31	0.94	[0.55 , 1.34]	Inferior by ≥20%	<0.001
[TKR] LCS Uncemented, unconstrained mobile	970	2.53	0.16	[-0.71 , 1.03]	Non-inferiority not shown	0.358
[TKR] MRK Cement, unconstrained fixed	554	2.91	0.54	[-0.04 , 1.13]	Non-inferiority not shown	0.035
[TKR] Maxim Cement, unconstrained fixed	408	4.32	1.95	[0.67 , 3.24]	Inferior by ≥20%	0.001
[TKR] Natural Knee II Cement, unconstrained fixed	582	3.88	1.51	[0.60 , 2.41]	Inferior by ≥20%	0.001
[TKR] NexGen Cement, posterior-stabilised mobile	268	7.13	4.76	[2.83 , 6.69]	Inferior by ≥100%	<0.001
[TKR] NexGen Cement, posterior-stabilised fixed	5,806	4.45	2.08	[1.79 , 2.37]	Inferior by ≥20%	<0.001
[TKR] NexGen Cement, unconstrained fixed	2,888	2.56	0.19	[-0.06 , 0.45]	Non-inferior	0.069
[TKR] NexGen Hybrid, unconstrained fixed	280	2.15	-0.22	[-1.17 , 0.73]	Non-inferiority not shown	0.326
[TKR] NexGen Uncemented, posterior-stabilised fixed	251	3.12	0.75	[-0.24 , 1.75]	Non-inferiority not shown	0.069
[TKR] NexGen Uncemented, unconstrained fixed	795	3.96	1.59	[0.91 , 2.26]	Inferior by ≥20%	<0.001
[TKR] PFC Sigma Bicondylar Knee Cement, posterior-stabilised mobile	784	4.06	1.69	[1.04 , 2.34]	Inferior by ≥20%	<0.001
[TKR] PFC Sigma Bicondylar Knee Cement, posterior-stabilised fixed	7,944	3.01	0.64	[0.43 , 0.84]	Non-inferiority not shown	<0.001
[TKR] PFC Sigma Bicondylar Knee Cement, unconstrained mobile	886	3.89	1.52	[0.94 , 2.10]	Inferior by ≥20%	<0.001
[TKR] PFC Sigma Bicondylar Knee Hybrid, unconstrained fixed	637	2.09	-0.28	[-1.03 , 0.47]	Non-inferior	0.234
[TKR] Profix Cement, unconstrained fixed	295	5.03	2.66	[1.14 , 4.18]	Inferior by ≥20%	<0.001
[TKR] Profix Uncemented, unconstrained fixed	564	2.21	-0.16	[-0.85 , 0.53]	Non-inferiority not shown	0.329

For peer review only

[TKR] Rotaglide + Cement, unconstrained mobile	696	5.61	3.24	[2.02 , 4.46]	Inferior by ≥20%	<0.001
[TKR] Scorpio Cement, posterior-stabilised mobile	480	3.27	0.90	[-0.14 , 1.94]	Non-inferiority not shown	0.045
[TKR] Scorpio Cement, posterior-stabilised fixed	1,995	3.85	1.48	[0.94 , 2.03]	Inferior by ≥20%	<0.001
[TKR] Scorpio Cement, unconstrained fixed	3,299	3.83	1.46	[1.04 , 1.87]	Inferior by ≥20%	<0.001
[TKR] Scorpio Hybrid, unconstrained fixed	316	4.27	1.90	[0.53 , 3.26]	Inferior by ≥20%	0.003
[TKR] Scorpio Uncemented, unconstrained fixed	517	4.13	1.76	[0.92 , 2.61]	Inferior by ≥20%	<0.001
[TKR] TC Plus Cement, unconstrained fixed	1,294	3.54	1.17	[0.67 , 1.66]	Inferior by ≥20%	<0.001
[TKR] TC Plus Cement, unconstrained mobile	1,493	3.13	0.76	[0.20 , 1.33]	Non-inferiority not shown	0.004
[TKR] Triathlon Cement, unconstrained fixed	334	3.20	0.83	[0.38 , 1.27]	Non-inferiority not shown	<0.001
[UNI] MG Uni Unicondylar, fixed	853	10.03	7.66	[6.33 , 9.00]	Inferior by ≥100%	<0.001
[UNI] Oxford Partial Knee Unicondylar, mobile	6,190	11.54	9.17	[8.73 , 9.62]	Inferior by ≥100%	<0.001
[UNI] Preservation Unicondylar, fixed	398	15.09	12.72	[10.59 , 14.86]	Inferior by ≥100%	<0.001

For peer review only

Supplementary table 5: Difference in cumulative percentage revision of knee implants compared to a contemporary benchmark at 3 years post primary in men for knee replacements with ≥250 procedures remaining at risk

Knee brand, bearing and constraint	Number at risk	Cumulative failure (%)	Difference in failure (%)	95% CI	Equivalence status	p-value
[TKR] NexGen Cement, unconstrained fixed	14,396	1.24	[REFERENCE]			
[TKR] AGC Cement, unconstrained fixed	21,525	1.61	0.36	[0.14 , 0.58]	Non-inferiority not shown	0.001
[TKR] AGC Hybrid, unconstrained fixed	400	2.07	0.83	[-0.52 , 2.17]	Non-inferiority not shown	0.115
[TKR] AGC Uncemented, unconstrained fixed	546	3.92	2.67	[1.10 , 4.25]	Inferior by ≥20%	<0.001
[TKR] Advance MP Cement, unconstrained fixed	2,482	2.39	1.15	[0.58 , 1.71]	Inferior by ≥20%	<0.001
[TKR] Advance PS Cement, posterior-stabilised fixed	342	2.55	1.30	[-0.20 , 2.80]	Non-inferiority not shown	0.045
[TKR] Columbus Cement, unconstrained fixed	2,209	1.78	0.54	[0.05 , 1.03]	Non-inferiority not shown	0.015
[TKR] E-Motion Bicondylar Knee Cement, unconstrained mobile	252	3.17	1.92	[-0.02 , 3.87]	Non-inferiority not shown	0.026
[TKR] E-Motion Bicondylar Knee Uncemented, unconstrained mobile	703	1.65	0.40	[-0.47 , 1.27]	Non-inferiority not shown	0.183
[TKR] FS Cement, unconstrained fixed	325	1.45	0.20	[-1.07 , 1.47]	Non-inferiority not shown	0.376
[TKR] Genesis 2 Cement, posterior-stabilised fixed	3,069	2.07	0.83	[0.37 , 1.29]	Inferior by ≥20%	<0.001
[TKR] Genesis 2 Cement, unconstrained fixed	10,877	1.47	0.22	[-0.03 , 0.47]	Non-inferiority not shown	0.042
[TKR] Genesis 2 Cement, unconstrained mobile	362	2.39	1.14	[-0.41 , 2.69]	Non-inferiority not shown	0.074
[TKR] Genesis 2 Oxinium Cement, posterior-stabilised fixed	700	3.69	2.44	[1.19 , 3.70]	Inferior by ≥20%	<0.001
[TKR] Genesis 2 Oxinium Cement, unconstrained fixed	1,574	2.48	1.23	[0.50 , 1.96]	Inferior by ≥20%	0.001
[TKR] Insall-Burstein 2 Cement, posterior-stabilised fixed	985	1.74	0.50	[-0.32 , 1.31]	Non-inferiority not shown	0.115
[TKR] Journey Oxinium Cement, posterior-stabilised fixed	338	3.13	1.89	[0.06 , 3.72]	Non-inferiority not shown	0.021
[TKR] Kinemax Cement, unconstrained fixed	4,241	1.88	0.64	[0.21 , 1.06]	Non-inferiority not shown	0.002
[TKR] LCS Complete Cement, unconstrained mobile	3,604	1.59	0.35	[-0.07 , 0.76]	Non-inferiority not shown	0.051
[TKR] LCS Complete Uncemented, unconstrained mobile	4,705	1.83	0.59	[0.21 , 0.97]	Non-inferiority not shown	0.001
[TKR] LCS Uncemented, unconstrained mobile	516	2.04	0.80	[-0.41 , 2.01]	Non-inferiority not shown	0.097
[TKR] MRK Cement, unconstrained fixed	2,493	1.52	0.27	[-0.18 , 0.72]	Non-inferiority not shown	0.117
[TKR] Maxim Cement, unconstrained fixed	530	1.09	-0.16	[-1.04 , 0.72]	Non-inferiority not shown	0.364
[TKR] NRG Cement, posterior-stabilised fixed	1,747	1.81	0.57	[-0.05 , 1.18]	Non-inferiority not shown	0.035
[TKR] NRG Cement, unconstrained fixed	2,486	1.83	0.59	[0.08 , 1.10]	Non-inferiority not shown	0.012
[TKR] Natural Knee II Cement, unconstrained fixed	997	1.03	-0.21	[-0.84 , 0.41]	Non-inferiority not shown	0.251
[TKR] NexGen Cement, posterior-stabilised mobile	324	3.61	2.36	[0.50 , 4.23]	Inferior by ≥20%	0.007
[TKR] NexGen Cement, posterior-stabilised fixed	16,394	1.95	0.71	[0.46 , 0.95]	Inferior by ≥20%	<0.001
[TKR] NexGen Hybrid, unconstrained fixed	457	0.81	-0.43	[-1.24 , 0.37]	Non-inferiority not shown	0.147
[TKR] NexGen Uncemented, posterior-stabilised fixed	774	1.35	0.10	[-0.64 , 0.85]	Non-inferiority not shown	0.392
[TKR] NexGen Uncemented, unconstrained fixed	1,965	2.47	1.22	[0.55 , 1.90]	Inferior by ≥20%	<0.001
[TKR] Optetrak Cement, posterior-stabilised fixed	614	2.01	0.77	[-0.32 , 1.87]	Non-inferiority not shown	0.084
[TKR] PFC Sigma Bicondylar Knee Cement, posterior-stabilised mobile	2,652	2.16	0.92	[0.38 , 1.46]	Inferior by ≥20%	<0.001
[TKR] PFC Sigma Bicondylar Knee Cement, constrained condylar	251	1.88	0.63	[-0.87 , 2.13]	Non-inferiority not shown	0.205
[TKR] PFC Sigma Bicondylar Knee Cement, posterior-stabilised fixed	22,261	1.65	0.40	[0.18 , 0.62]	Non-inferiority not shown	<0.001
[TKR] PFC Sigma Bicondylar Knee Cement, unconstrained fixed	53,160	1.39	0.15	[-0.03 , 0.33]	Non-inferiority not shown	0.050
[TKR] PFC Sigma Bicondylar Knee Cement, unconstrained mobile	3,150	2.14	0.89	[0.39 , 1.39]	Inferior by ≥20%	<0.001
[TKR] PFC Sigma Bicondylar Knee Hybrid, unconstrained fixed	700	0.95	-0.29	[-1.01 , 0.43]	Non-inferiority not shown	0.213
[TKR] PFC Sigma Bicondylar Knee Uncemented, unconstrained mobile	280	1.61	0.36	[-0.93 , 1.65]	Non-inferiority not shown	0.292
[TKR] PFC Sigma Bicondylar Knee monobloc polyethylene tibia	2,112	1.44	0.19	[-0.26 , 0.64]	Non-inferiority not shown	0.204

For peer review only

[TKR] Profix Cement, unconstrained fixed	392	2.15	0.90	[-0.49 , 2.30]	Non-inferiority not shown	0.102
[TKR] Profix Uncemented, unconstrained fixed	954	1.00	-0.25	[-0.88 , 0.39]	Non-inferiority not shown	0.225
[TKR] Profix monobloc polyethylene tibia	257	0.36	-0.88	[-1.61 , -0.15]	Non-inferior	0.009
[TKR] Rotaglide + Cement, unconstrained mobile	668	3.36	2.12	[0.79 , 3.45]	Inferior by ≥20%	0.001
[TKR] Rotaglide Cement, unconstrained mobile	403	2.75	1.51	[0.02 , 2.99]	Non-inferiority not shown	0.024
[TKR] Scorpio Cement, posterior-stabilised mobile	565	1.69	0.44	[-0.61 , 1.49]	Non-inferiority not shown	0.204
[TKR] Scorpio Cement, posterior-stabilised fixed	2,265	1.97	0.72	[0.14 , 1.30]	Non-inferiority not shown	0.007
[TKR] Scorpio Cement, unconstrained fixed	4,090	2.25	1.00	[0.53 , 1.47]	Inferior by ≥20%	<0.001
[TKR] Scorpio Cement, unconstrained mobile	464	2.66	1.41	[-0.02 , 2.84]	Non-inferiority not shown	0.027
[TKR] Scorpio Hybrid, unconstrained fixed	405	2.32	1.07	[-0.35 , 2.50]	Non-inferiority not shown	0.070
[TKR] Scorpio Uncemented, unconstrained fixed	1,646	1.75	0.51	[-0.13 , 1.15]	Non-inferiority not shown	0.060
[TKR] TC Plus Cement, unconstrained fixed	3,396	2.32	1.07	[0.56 , 1.59]	Inferior by ≥20%	<0.001
[TKR] TC Plus Cement, unconstrained mobile	1,934	1.98	0.74	[0.12 , 1.35]	Non-inferiority not shown	0.009
[TKR] TC Plus Uncemented, unconstrained mobile	550	1.43	0.18	[-0.71 , 1.08]	Non-inferiority not shown	0.345
[TKR] Triathlon Cement, posterior-stabilised fixed	3,282	1.77	0.52	[0.12 , 0.93]	Non-inferiority not shown	0.006
[TKR] Triathlon Cement, unconstrained fixed	12,371	1.42	0.17	[-0.06 , 0.40]	Non-inferiority not shown	0.073
[TKR] Triathlon Uncemented, unconstrained fixed	332	2.49	1.24	[-0.19 , 2.67]	Non-inferiority not shown	0.044
[TKR] Vanguard Cement, posterior-stabilised fixed	1,226	1.75	0.51	[-0.08 , 1.10]	Non-inferiority not shown	0.046
[TKR] Vanguard Cement, unconstrained fixed	8,120	1.51	0.27	[-0.00 , 0.54]	Non-inferiority not shown	0.025
[UNI] AMC/Uniglide Unicondylar, fixed	501	2.65	1.40	[0.07 , 2.74]	Non-inferiority not shown	0.020
[UNI] AMC/Uniglide Unicondylar, mobile	628	7.99	6.75	[4.78 , 8.71]	Inferior by ≥100%	<0.001
[UNI] MG Uni Unicondylar, fixed	1,203	3.48	2.23	[1.21 , 3.26]	Inferior by ≥20%	<0.001
[UNI] Oxford Partial Knee Unicondylar, mobile	19,491	3.98	2.74	[2.45 , 3.03]	Inferior by ≥100%	<0.001
[UNI] Physica ZUK Unicondylar, fixed	2,555	2.33	1.08	[0.57 , 1.60]	Inferior by ≥20%	<0.001
[UNI] Preservation Unicondylar, fixed	600	5.68	4.44	[2.65 , 6.22]	Inferior by ≥100%	<0.001
[UNI] Sigma HP Unicondylar, fixed	2,062	3.12	1.87	[1.24 , 2.51]	Inferior by ≥20%	<0.001
[UNI] Sled Unicondylar, fixed	308	6.69	5.45	[2.85 , 8.04]	Inferior by ≥100%	<0.001

For peer review only

Supplementary table 6: Difference in cumulative percentage revision of knee implants compared to a contemporary benchmark at 7 years post primary in men for knee replacements with ≥250 procedures remaining at risk

Knee brand, bearing and constraint	Number at risk	Cumulative failure (%)	Difference in failure (%)	95% CI	Equivalence status	p-value
[TKR] PFC Sigma Bicondylar Knee Cement, unconstrained fixed	22,730	2.16	[REFERENCE]			
[TKR] AGC Cement, unconstrained fixed	11,118	2.94	0.78	[0.51 , 1.04]	Inferior by ≥20%	<0.001
[TKR] AGC Hybrid, unconstrained fixed	250	3.18	1.02	[-0.70 , 2.73]	Non-inferiority not shown	0.123
[TKR] AGC Uncemented, unconstrained fixed	324	5.56	3.40	[1.48 , 5.33]	Inferior by ≥20%	<0.001
[TKR] Advance MP Cement, unconstrained fixed	1,052	3.91	1.75	[0.96 , 2.53]	Inferior by ≥20%	<0.001
[TKR] Columbus Cement, unconstrained fixed	625	2.70	0.53	[-0.15 , 1.22]	Non-inferiority not shown	0.064
[TKR] E-Motion Bicondylar Knee Uncemented, unconstrained mobile	352	3.20	1.04	[-0.34 , 2.43]	Non-inferiority not shown	0.070
[TKR] FS Cement, unconstrained fixed	276	2.10	-0.06	[-1.61 , 1.48]	Non-inferiority not shown	0.468
[TKR] Genesis 2 Cement, posterior-stabilised fixed	848	3.70	1.54	[0.80 , 2.28]	Inferior by ≥20%	<0.001
[TKR] Genesis 2 Cement, unconstrained fixed	3,359	2.69	0.53	[0.18 , 0.88]	Non-inferiority not shown	0.002
[TKR] Genesis 2 Oxinium Cement, unconstrained fixed	758	3.98	1.82	[0.83 , 2.81]	Inferior by ≥20%	<0.001
[TKR] Insall-Burstein 2 Cement, posterior-stabilised fixed	797	3.57	1.40	[0.23 , 2.57]	Non-inferiority not shown	0.009
[TKR] Kinemax Cement, unconstrained fixed	3,602	3.87	1.71	[1.12 , 2.30]	Inferior by ≥20%	<0.001
[TKR] LCS Complete Cement, unconstrained mobile	1,775	3.47	1.31	[0.68 , 1.94]	Inferior by ≥20%	<0.001
[TKR] LCS Complete Uncemented, unconstrained mobile	2,105	2.92	0.76	[0.25 , 1.27]	Non-inferiority not shown	0.002
[TKR] LCS Uncemented, unconstrained mobile	457	2.82	0.66	[-0.75 , 2.07]	Non-inferiority not shown	0.181
[TKR] MRK Cement, unconstrained fixed	1,056	2.97	0.80	[0.08 , 1.53]	Non-inferiority not shown	0.015
[TKR] Maxim Cement, unconstrained fixed	380	2.84	0.68	[-0.81 , 2.16]	Non-inferiority not shown	0.186
[TKR] NRG Cement, posterior-stabilised fixed	580	3.14	0.98	[0.13 , 1.83]	Non-inferiority not shown	0.012
[TKR] NRG Cement, unconstrained fixed	908	3.76	1.60	[0.77 , 2.43]	Inferior by ≥20%	<0.001
[TKR] Natural Knee II Cement, unconstrained fixed	559	3.46	1.30	[0.05 , 2.55]	Non-inferiority not shown	0.021
[TKR] NexGen Cement, posterior-stabilised fixed	7,174	3.81	1.65	[1.32 , 1.99]	Inferior by ≥20%	<0.001
[TKR] NexGen Cement, unconstrained fixed	4,862	2.32	0.16	[-0.14 , 0.45]	Non-inferiority not shown	0.151
[TKR] NexGen Uncemented, posterior-stabilised fixed	419	2.04	-0.12	[-1.13 , 0.89]	Non-inferiority not shown	0.410
[TKR] NexGen Uncemented, unconstrained fixed	1,270	3.52	1.35	[0.53 , 2.17]	Inferior by ≥20%	0.001
[TKR] Optetrak Cement, posterior-stabilised fixed	335	4.79	2.63	[0.87 , 4.38]	Inferior by ≥20%	0.002
[TKR] PFC Sigma Bicondylar Knee Cement, posterior-stabilised mobile	1,226	3.23	1.07	[0.37 , 1.77]	Non-inferiority not shown	0.001
[TKR] PFC Sigma Bicondylar Knee Cement, posterior-stabilised fixed	9,690	2.53	0.37	[0.12 , 0.61]	Non-inferiority not shown	0.002
[TKR] PFC Sigma Bicondylar Knee Cement, unconstrained mobile	1,807	3.24	1.08	[0.44 , 1.71]	Inferior by ≥20%	<0.001
[TKR] PFC Sigma Bicondylar Knee Hybrid, unconstrained fixed	527	1.86	-0.31	[-1.31 , 0.70]	Non-inferiority not shown	0.277
[TKR] PFC Sigma Bicondylar Knee monobloc polyethylene tibia	287	2.16	0.00	[-0.75 , 0.75]	Non-inferiority not shown	0.498
[TKR] Profix Cement, unconstrained fixed	306	5.09	2.93	[0.74 , 5.11]	Inferior by ≥20%	0.004
[TKR] Profix Uncemented, unconstrained fixed	676	1.73	-0.44	[-1.29 , 0.42]	Non-inferior	0.158
[TKR] Rotaglide + Cement, unconstrained mobile	509	5.27	3.10	[1.42 , 4.79]	Inferior by ≥20%	<0.001
[TKR] Scorpio Cement, posterior-stabilised mobile	437	2.60	0.44	[-0.87 , 1.74]	Non-inferiority not shown	0.256
[TKR] Scorpio Cement, posterior-stabilised fixed	1,835	3.88	1.72	[0.91 , 2.53]	Inferior by ≥20%	<0.001
[TKR] Scorpio Cement, unconstrained fixed	2,978	3.76	1.60	[1.00 , 2.20]	Inferior by ≥20%	<0.001
[TKR] Scorpio Cement, unconstrained mobile	392	4.62	2.46	[0.57 , 4.35]	Inferior by ≥20%	0.005
[TKR] Scorpio Hybrid, unconstrained fixed	326	4.13	1.97	[0.04 , 3.91]	Non-inferiority not shown	0.023
[TKR] Scorpio Uncemented, unconstrained fixed	993	3.15	0.99	[0.11 , 1.87]	Non-inferiority not shown	0.014

For peer review only

[TKR] TC Plus Cement, unconstrained fixed	2,380	3.40	1.24	[0.62 , 1.86]	Inferior by ≥20%	<0.001
[TKR] TC Plus Cement, unconstrained mobile	1,419	3.20	1.04	[0.25 , 1.82]	Non-inferiority not shown	0.005
[TKR] TC Plus Uncemented, unconstrained mobile	281	2.32	0.16	[-1.10 , 1.42]	Non-inferiority not shown	0.400
[TKR] Triathlon Cement, posterior-stabilised fixed	614	2.75	0.59	[0.03 , 1.15]	Non-inferiority not shown	0.019
[TKR] Triathlon Cement, unconstrained fixed	1,973	2.52	0.36	[-0.00 , 0.72]	Non-inferiority not shown	0.026
[TKR] Vanguard Cement, unconstrained fixed	1,017	2.24	0.08	[-0.27 , 0.42]	Non-inferior	0.336
[UNI] AMC/Uniglide Unicondylar, mobile	350	10.65	8.49	[6.16 , 10.82]	Inferior by ≥100%	<0.001
[UNI] MG Uni Unicondylar, fixed	990	6.59	4.43	[3.04 , 5.83]	Inferior by ≥100%	<0.001
[UNI] Oxford Partial Knee Unicondylar, mobile	9,524	7.45	5.29	[4.89 , 5.69]	Inferior by ≥100%	<0.001
[UNI] Physica ZUK Unicondylar, fixed	594	4.64	2.48	[1.57 , 3.40]	Inferior by ≥20%	<0.001
[UNI] Preservation Unicondylar, fixed	505	11.21	9.05	[6.58 , 11.51]	Inferior by ≥100%	<0.001

For peer review only

Supplementary table 7: Difference in cumulative percentage revision of knee implants compared to a contemporary benchmark at 10 years post primary in men for knee replacements with ≥250 procedures remaining at risk

Knee brand, bearing and constraint	Number at risk	Cumulative failure (%)	Difference in failure (%)	95% CI	Equivalence status	p-value
[TKR] PFC Sigma Bicondylar Knee Cement, unconstrained fixed	7,760	2.55	[REFERENCE]			
[TKR] AGC Cement, unconstrained fixed	3,646	4.12	1.57	[1.18 , 1.95]	Inferior by ≥20%	<0.001
[TKR] Advance MP Cement, unconstrained fixed	279	4.50	1.94	[0.85 , 3.03]	Inferior by ≥20%	<0.001
[TKR] Genesis 2 Cement, unconstrained fixed	807	3.26	0.70	[0.21 , 1.19]	Non-inferiority not shown	0.003
[TKR] Insall-Burstein 2 Cement, posterior-stabilised fixed	457	5.91	3.35	[1.73 , 4.98]	Inferior by ≥20%	<0.001
[TKR] Kinemax Cement, unconstrained fixed	2,113	5.00	2.45	[1.75 , 3.15]	Inferior by ≥20%	<0.001
[TKR] LCS Complete Cement, unconstrained mobile	303	4.25	1.70	[0.91 , 2.49]	Inferior by ≥20%	<0.001
[TKR] LCS Complete Uncemented, unconstrained mobile	602	3.33	0.78	[0.18 , 1.38]	Non-inferiority not shown	0.006
[TKR] LCS Uncemented, unconstrained mobile	406	3.04	0.49	[-0.99 , 1.97]	Non-inferiority not shown	0.258
[TKR] NexGen Cement, posterior-stabilised fixed	2,308	5.14	2.58	[2.10 , 3.07]	Inferior by ≥20%	<0.001
[TKR] NexGen Cement, unconstrained fixed	1,167	2.71	0.16	[-0.24 , 0.55]	Non-inferiority not shown	0.220
[TKR] NexGen Uncemented, unconstrained fixed	348	3.61	1.05	[0.21 , 1.90]	Non-inferiority not shown	0.007
[TKR] PFC Sigma Bicondylar Knee Cement, posterior-stabilised mobile	340	4.31	1.75	[0.71 , 2.80]	Inferior by ≥20%	0.001
[TKR] PFC Sigma Bicondylar Knee Cement, posterior-stabilised fixed	3,119	3.16	0.61	[0.28 , 0.94]	Non-inferiority not shown	<0.001
[TKR] PFC Sigma Bicondylar Knee Cement, unconstrained mobile	419	3.97	1.41	[0.55 , 2.27]	Inferior by ≥20%	0.001
[TKR] PFC Sigma Bicondylar Knee Hybrid, unconstrained fixed	276	2.14	-0.41	[-1.57 , 0.74]	Non-inferiority not shown	0.241
[TKR] Profix Uncemented, unconstrained fixed	278	2.35	-0.20	[-1.32 , 0.91]	Non-inferiority not shown	0.361
[TKR] Rotaglide + Cement, unconstrained mobile	289	6.95	4.40	[2.31 , 6.48]	Inferior by ≥20%	<0.001
[TKR] Scorpio Cement, posterior-stabilised fixed	794	4.65	2.10	[1.16 , 3.03]	Inferior by ≥20%	<0.001
[TKR] Scorpio Cement, unconstrained fixed	1,325	4.43	1.87	[1.19 , 2.56]	Inferior by ≥20%	<0.001
[TKR] TC Plus Cement, unconstrained fixed	565	4.15	1.60	[0.79 , 2.41]	Inferior by ≥20%	<0.001
[TKR] TC Plus Cement, unconstrained mobile	668	4.06	1.50	[0.55 , 2.45]	Inferior by ≥20%	0.001
[UNI] MG Uni Unicondylar, fixed	445	8.58	6.02	[4.34 , 7.70]	Inferior by ≥100%	<0.001
[UNI] Oxford Partial Knee Unicondylar, mobile	3,124	10.63	8.08	[7.48 , 8.67]	Inferior by ≥100%	<0.001

For peer review only

Supplementary table 8: Difference in cumulative percentage revision of knee implants compared to a contemporary benchmark at 3 years post primary in women for knee replacements with ≥250 procedures remaining at risk

Knee brand, bearing and constraint	Number at risk	Cumulative failure (%)	Difference in failure (%)	95% CI	Equivalence status	p-value
[TKR] MRK Cement, unconstrained fixed	3,684	0.97	[REFERENCE]			
[TKR] AGC Cement, posterior-stabilised fixed	292	2.31	1.33	[-0.38 , 3.05]	Non-inferiority not shown	0.063
[TKR] AGC Cement, unconstrained fixed	29,830	1.30	0.33	[0.02 , 0.63]	Non-inferiority not shown	0.017
[TKR] AGC Hybrid, unconstrained fixed	462	2.46	1.49	[0.09 , 2.90]	Non-inferiority not shown	0.019
[TKR] AGC Uncemented, unconstrained fixed	536	4.24	3.27	[1.59 , 4.95]	Inferior by ≥100%	<0.001
[TKR] Advance MP Cement, unconstrained fixed	3,068	1.74	0.77	[0.26 , 1.27]	Inferior by ≥20%	0.002
[TKR] Advance MP Stature Cement, unconstrained fixed	693	1.84	0.87	[-0.04 , 1.78]	Non-inferiority not shown	0.031
[TKR] Advance PS Cement, posterior-stabilised fixed	449	2.27	1.29	[-0.01 , 2.60]	Non-inferiority not shown	0.026
[TKR] Alpina Hybrid, unconstrained fixed	278	1.74	0.77	[-0.77 , 2.31]	Non-inferiority not shown	0.164
[TKR] Columbus Cement, unconstrained fixed	2,921	1.78	0.81	[0.32 , 1.30]	Inferior by ≥20%	0.001
[TKR] E-Motion Bicondylar Knee Cement, unconstrained mobile	451	2.94	1.96	[0.55 , 3.38]	Inferior by ≥20%	0.003
[TKR] E-Motion Bicondylar Knee Uncemented, unconstrained mobile	692	2.42	1.45	[0.36 , 2.53]	Inferior by ≥20%	0.004
[TKR] FS Cement, unconstrained fixed	417	1.62	0.65	[-0.58 , 1.87]	Non-inferiority not shown	0.150
[TKR] Genesis 2 Cement, posterior-stabilised fixed	4,795	1.53	0.56	[0.16 , 0.96]	Non-inferiority not shown	0.003
[TKR] Genesis 2 Cement, unconstrained fixed	14,679	1.12	0.15	[-0.16 , 0.46]	Non-inferiority not shown	0.174
[TKR] Genesis 2 Cement, unconstrained mobile	526	1.29	0.32	[-0.67 , 1.31]	Non-inferiority not shown	0.263
[TKR] Genesis 2 Oxinium Cement, posterior-stabilised fixed	981	2.41	1.44	[0.55 , 2.32]	Inferior by ≥20%	0.001
[TKR] Genesis 2 Oxinium Cement, unconstrained fixed	2,060	1.55	0.57	[0.01 , 1.14]	Non-inferiority not shown	0.023
[TKR] Insall-Burstein 2 Cement, posterior-stabilised fixed	1,242	1.25	0.28	[-0.39 , 0.95]	Non-inferiority not shown	0.205
[TKR] Journey Oxinium Cement, posterior-stabilised fixed	407	5.77	4.80	[2.59 , 7.01]	Inferior by ≥100%	<0.001
[TKR] Kinemax Cement, unconstrained fixed	5,695	1.71	0.73	[0.30 , 1.16]	Inferior by ≥20%	<0.001
[TKR] LCS Cement, unconstrained mobile	363	1.58	0.60	[-0.68 , 1.89]	Non-inferiority not shown	0.178
[TKR] LCS Complete Cement, unconstrained mobile	4,847	1.65	0.68	[0.24 , 1.11]	Inferior by ≥20%	0.001
[TKR] LCS Complete Uncemented, unconstrained mobile	5,591	1.68	0.71	[0.29 , 1.13]	Inferior by ≥20%	<0.001
[TKR] LCS Uncemented, unconstrained mobile	722	1.74	0.77	[-0.21 , 1.75]	Non-inferiority not shown	0.062
[TKR] Maxim Cement, posterior-stabilised fixed	340	1.69	0.72	[-0.65 , 2.09]	Non-inferiority not shown	0.153
[TKR] Maxim Cement, unconstrained fixed	715	1.76	0.79	[-0.20 , 1.78]	Non-inferiority not shown	0.059
[TKR] NRG Cement, posterior-stabilised fixed	2,394	1.63	0.66	[0.10 , 1.22]	Non-inferiority not shown	0.011
[TKR] NRG Cement, unconstrained fixed	3,377	1.22	0.24	[-0.20 , 0.68]	Non-inferiority not shown	0.140
[TKR] Natural Knee II Cement, unconstrained fixed	1,485	1.60	0.63	[-0.05 , 1.31]	Non-inferiority not shown	0.035
[TKR] NexGen Cement, posterior-stabilised mobile	490	2.31	1.33	[0.10 , 2.57]	Non-inferiority not shown	0.017
[TKR] NexGen Cement, posterior-stabilised fixed	24,309	1.38	0.40	[0.10 , 0.71]	Non-inferiority not shown	0.005
[TKR] NexGen Cement, unconstrained fixed	20,162	0.99	0.02	[-0.28 , 0.32]	Non-inferiority not shown	0.452
[TKR] NexGen Hybrid, unconstrained fixed	507	1.30	0.33	[-0.67 , 1.33]	Non-inferiority not shown	0.259
[TKR] NexGen Uncemented, posterior-stabilised fixed	650	2.15	1.18	[0.12 , 2.23]	Non-inferiority not shown	0.014
[TKR] NexGen Uncemented, unconstrained fixed	1,577	2.66	1.69	[0.87 , 2.51]	Inferior by ≥20%	<0.001
[TKR] NexGen monobloc polyethylene tibia	314	0.46	-0.51	[-1.22 , 0.20]	Non-inferiority not shown	0.080
[TKR] Optetrak Cement, posterior-stabilised fixed	882	3.11	2.13	[0.99 , 3.28]	Inferior by ≥100%	<0.001
[TKR] PFC Sigma Bicondylar Knee Cement, posterior-stabilised mobile	3,081	2.08	1.10	[0.56 , 1.65]	Inferior by ≥20%	<0.001
[TKR] PFC Sigma Bicondylar Knee Cement, constrained condylar	310	1.62	0.64	[-0.58 , 1.87]	Non-inferiority not shown	0.152

For peer review only

[TKR] PFC Sigma Bicondylar Knee Cement, posterior-stabilised fixed	32,691	1.42	0.45	[0.15 , 0.75]	Non-inferiority not shown	0.002
[TKR] PFC Sigma Bicondylar Knee Cement, unconstrained fixed	72,974	1.13	0.16	[-0.12 , 0.45]	Non-inferiority not shown	0.134
[TKR] PFC Sigma Bicondylar Knee Cement, unconstrained mobile	3,424	1.75	0.78	[0.28 , 1.28]	Inferior by ≥20%	0.001
[TKR] PFC Sigma Bicondylar Knee Hybrid, unconstrained fixed	833	1.38	0.40	[-0.42 , 1.23]	Non-inferiority not shown	0.167
[TKR] PFC Sigma Bicondylar Knee Uncemented, unconstrained mobile	324	1.28	0.31	[-0.85 , 1.47]	Non-inferiority not shown	0.299
[TKR] PFC Sigma Bicondylar Knee monobloc polyethylene tibia	3,257	1.22	0.24	[-0.17 , 0.66]	Non-inferiority not shown	0.126
[TKR] Profix Cement, unconstrained fixed	492	1.96	0.99	[-0.25 , 2.22]	Non-inferiority not shown	0.059
[TKR] Profix Oxinium monobloc polyethylene tibia	327	2.68	1.71	[-0.04 , 3.45]	Non-inferiority not shown	0.028
[TKR] Profix Uncemented, unconstrained fixed	1,184	1.46	0.49	[-0.24 , 1.22]	Non-inferiority not shown	0.093
[TKR] Profix monobloc polyethylene tibia	395	0.96	-0.01	[-0.99 , 0.97]	Non-inferiority not shown	0.493
[TKR] Rotaglide + Cement, unconstrained mobile	909	2.42	1.45	[0.43 , 2.46]	Inferior by ≥20%	0.003
[TKR] Rotaglide Cement, unconstrained mobile	649	1.62	0.64	[-0.31 , 1.60]	Non-inferiority not shown	0.092
[TKR] Scorpio Cement, posterior-stabilised mobile	725	1.33	0.36	[-0.51 , 1.23]	Non-inferiority not shown	0.208
[TKR] Scorpio Cement, posterior-stabilised fixed	3,409	1.33	0.36	[-0.11 , 0.82]	Non-inferiority not shown	0.068
[TKR] Scorpio Cement, unconstrained fixed	5,993	1.58	0.60	[0.19 , 1.02]	Non-inferiority not shown	0.002
[TKR] Scorpio Cement, unconstrained mobile	631	2.45	1.48	[0.26 , 2.69]	Inferior by ≥20%	0.009
[TKR] Scorpio Hybrid, unconstrained fixed	601	1.44	0.46	[-0.51 , 1.43]	Non-inferiority not shown	0.176
[TKR] Scorpio Uncemented, unconstrained fixed	1,885	1.83	0.86	[0.20 , 1.51]	Inferior by ≥20%	0.005
[TKR] TC Plus Cement, unconstrained fixed	4,106	1.53	0.55	[0.09 , 1.01]	Non-inferiority not shown	0.009
[TKR] TC Plus Cement, unconstrained mobile	2,452	1.02	0.04	[-0.43 , 0.51]	Non-inferiority not shown	0.428
[TKR] TC Plus Uncemented, unconstrained mobile	890	1.56	0.59	[-0.22 , 1.40]	Non-inferiority not shown	0.077
[TKR] Triathlon Cement, posterior-stabilised fixed	4,914	1.70	0.72	[0.31 , 1.14]	Inferior by ≥20%	<0.001
[TKR] Triathlon Cement, unconstrained fixed	16,831	1.53	0.56	[0.24 , 0.87]	Inferior by ≥20%	<0.001
[TKR] Triathlon Uncemented, unconstrained fixed	411	2.97	1.99	[0.51 , 3.47]	Inferior by ≥20%	0.004
[TKR] Vanguard Cement, constrained condylar	260	0.39	-0.59	[-1.11 , -0.06]	Non-inferior	0.014
[TKR] Vanguard Cement, posterior-stabilised fixed	1,764	2.01	1.04	[0.45 , 1.63]	Inferior by ≥20%	<0.001
[TKR] Vanguard Cement, unconstrained fixed	11,272	1.27	0.30	[-0.03 , 0.62]	Non-inferiority not shown	0.037
[UNI] AMC/Uniglide Unicondylar, fixed	571	3.27	2.30	[0.89 , 3.70]	Inferior by ≥20%	0.001
[UNI] AMC/Uniglide Unicondylar, mobile	568	9.00	8.03	[5.82 , 10.24]	Inferior by ≥100%	<0.001
[UNI] MG Uni Unicondylar, fixed	1,004	4.54	3.57	[2.28 , 4.85]	Inferior by ≥100%	<0.001
[UNI] Oxford Partial Knee Unicondylar, mobile	17,891	4.18	3.20	[2.82 , 3.59]	Inferior by ≥100%	<0.001
[UNI] Physica ZUK Unicondylar, fixed	2,153	2.59	1.61	[0.98 , 2.25]	Inferior by ≥100%	<0.001
[UNI] Preservation Unicondylar, fixed	506	8.29	7.32	[5.01 , 9.63]	Inferior by ≥100%	<0.001
[UNI] Sigma HP Unicondylar, fixed	1,499	4.15	3.17	[2.30 , 4.05]	Inferior by ≥100%	<0.001
[UNI] Sled Unicondylar, fixed	375	5.85	4.87	[2.63 , 7.12]	Inferior by ≥100%	<0.001

For peer review only

Supplementary table 9: Difference in cumulative percentage revision of knee implants compared to a contemporary benchmark at 7 years post primary in women for knee replacements with ≥250 procedures remaining at risk

Knee brand, bearing and constraint	Number at risk	Cumulative failure (%)	Difference in failure (%)	95% CI	Equivalence status	p-value
[TKR] Genesis 2 Cement, unconstrained fixed	4,697	1.77	[REFERENCE]			
[TKR] AGC Cement, unconstrained fixed	16,170	2.29	0.52	[0.23 , 0.80]	Non-inferiority not shown	<0.001
[TKR] AGC Hybrid, unconstrained fixed	292	3.33	1.56	[-0.06 , 3.18]	Non-inferiority not shown	0.030
[TKR] AGC Uncemented, unconstrained fixed	387	6.37	4.59	[2.54 , 6.65]	Inferior by ≥100%	<0.001
[TKR] Advance MP Cement, unconstrained fixed	1,537	3.17	1.40	[0.72 , 2.08]	Inferior by ≥20%	<0.001
[TKR] Columbus Cement, unconstrained fixed	858	2.87	1.10	[0.42 , 1.77]	Inferior by ≥20%	0.001
[TKR] E-Motion Bicondylar Knee Uncemented, unconstrained mobile	381	4.50	2.73	[1.11 , 4.35]	Inferior by ≥20%	<0.001
[TKR] FS Cement, unconstrained fixed	368	3.10	1.32	[-0.35 , 3.00]	Non-inferiority not shown	0.061
[TKR] Genesis 2 Cement, posterior-stabilised fixed	1,428	2.50	0.73	[0.22 , 1.24]	Non-inferiority not shown	0.003
[TKR] Genesis 2 Cement, unconstrained mobile	328	1.97	0.20	[-1.04 , 1.44]	Non-inferiority not shown	0.376
[TKR] Genesis 2 Oxinium Cement, posterior-stabilised fixed	324	5.72	3.94	[2.18 , 5.70]	Inferior by ≥100%	<0.001
[TKR] Genesis 2 Oxinium Cement, unconstrained fixed	906	3.15	1.38	[0.57 , 2.19]	Inferior by ≥20%	<0.001
[TKR] Insall-Burstein 2 Cement, posterior-stabilised fixed	1,086	3.31	1.54	[0.51 , 2.56]	Inferior by ≥20%	0.002
[TKR] Kinemax Cement, unconstrained fixed	4,958	3.28	1.51	[0.99 , 2.02]	Inferior by ≥20%	<0.001
[TKR] LCS Cement, unconstrained mobile	312	2.47	0.70	[-0.92 , 2.31]	Non-inferiority not shown	0.199
[TKR] LCS Complete Cement, unconstrained mobile	2,442	3.42	1.64	[1.06 , 2.22]	Inferior by ≥20%	<0.001
[TKR] LCS Complete Uncemented, unconstrained mobile	2,619	2.93	1.15	[0.65 , 1.65]	Inferior by ≥20%	<0.001
[TKR] LCS Uncemented, unconstrained mobile	636	2.16	0.39	[-0.68 , 1.46]	Non-inferiority not shown	0.238
[TKR] MRK Cement, unconstrained fixed	1,720	1.83	0.05	[-0.45 , 0.56]	Non-inferiority not shown	0.416
[TKR] Maxim Cement, unconstrained fixed	502	3.22	1.44	[0.09 , 2.80]	Non-inferiority not shown	0.018
[TKR] NRG Cement, posterior-stabilised fixed	859	2.66	0.89	[0.17 , 1.60]	Non-inferiority not shown	0.008
[TKR] NRG Cement, unconstrained fixed	1,301	2.15	0.38	[-0.18 , 0.94]	Non-inferiority not shown	0.092
[TKR] Natural Knee II Cement, unconstrained fixed	879	3.04	1.26	[0.32 , 2.21]	Non-inferiority not shown	0.005
[TKR] NexGen Cement, posterior-stabilised mobile	337	5.09	3.32	[1.36 , 5.28]	Inferior by ≥20%	<0.001
[TKR] NexGen Cement, posterior-stabilised fixed	10,683	2.97	1.20	[0.88 , 1.52]	Inferior by ≥20%	<0.001
[TKR] NexGen Cement, unconstrained fixed	7,469	1.98	0.21	[-0.10 , 0.51]	Non-inferiority not shown	0.095
[TKR] NexGen Hybrid, unconstrained fixed	333	2.18	0.40	[-0.89 , 1.70]	Non-inferiority not shown	0.271
[TKR] NexGen Uncemented, posterior-stabilised fixed	366	3.11	1.34	[0.00 , 2.67]	Non-inferiority not shown	0.025
[TKR] NexGen Uncemented, unconstrained fixed	1,261	3.67	1.90	[0.96 , 2.84]	Inferior by ≥20%	<0.001
[TKR] Optetrak Cement, posterior-stabilised fixed	474	5.87	4.10	[2.52 , 5.67]	Inferior by ≥100%	<0.001
[TKR] PFC Sigma Bicondylar Knee Cement, posterior-stabilised mobile	1,460	3.46	1.69	[0.99 , 2.39]	Inferior by ≥20%	<0.001
[TKR] PFC Sigma Bicondylar Knee Cement, posterior-stabilised fixed	14,968	2.39	0.61	[0.33 , 0.90]	Non-inferiority not shown	<0.001
[TKR] PFC Sigma Bicondylar Knee Cement, unconstrained fixed	32,901	1.89	0.11	[-0.13 , 0.36]	Non-inferiority not shown	0.183
[TKR] PFC Sigma Bicondylar Knee Cement, unconstrained mobile	2,012	3.07	1.30	[0.68 , 1.92]	Inferior by ≥20%	<0.001
[TKR] PFC Sigma Bicondylar Knee Hybrid, unconstrained fixed	648	1.88	0.10	[-0.84 , 1.04]	Non-inferiority not shown	0.414
[TKR] PFC Sigma Bicondylar Knee monobloc polyethylene tibia	523	1.78	0.00	[-0.56 , 0.57]	Non-inferiority not shown	0.496
[TKR] Profix Cement, unconstrained fixed	388	3.48	1.71	[0.06 , 3.36]	Non-inferiority not shown	0.021
[TKR] Profix Oxinium monobloc polyethylene tibia	276	3.90	2.13	[0.04 , 4.22]	Non-inferiority not shown	0.023
[TKR] Profix Uncemented, unconstrained fixed	827	1.81	0.04	[-0.75 , 0.82]	Non-inferiority not shown	0.461
[TKR] Profix monobloc polyethylene tibia	323	1.47	-0.30	[-1.49 , 0.89]	Non-inferiority not shown	0.309

For peer review only

[TKR] Rotaglide + Cement, unconstrained mobile	715	3.45	1.68	[0.48 , 2.88]	Inferior by ≥20%	0.003
[TKR] Rotaglide Cement, unconstrained mobile	318	3.32	1.55	[0.10 , 3.00]	Non-inferiority not shown	0.018
[TKR] Scorpio Cement, posterior-stabilised mobile	577	2.50	0.72	[-0.44 , 1.89]	Non-inferiority not shown	0.112
[TKR] Scorpio Cement, posterior-stabilised fixed	2,841	2.53	0.76	[0.18 , 1.33]	Non-inferiority not shown	0.005
[TKR] Scorpio Cement, unconstrained fixed	4,356	2.72	0.95	[0.47 , 1.42]	Inferior by ≥20%	<0.001
[TKR] Scorpio Cement, unconstrained mobile	559	4.37	2.60	[1.00 , 4.20]	Inferior by ≥20%	0.001
[TKR] Scorpio Hybrid, unconstrained fixed	499	2.69	0.92	[-0.41 , 2.24]	Non-inferiority not shown	0.087
[TKR] Scorpio Uncemented, unconstrained fixed	1,219	3.07	1.29	[0.47 , 2.11]	Inferior by ≥20%	0.001
[TKR] TC Plus Cement, unconstrained fixed	2,962	2.62	0.85	[0.31 , 1.38]	Non-inferiority not shown	0.001
[TKR] TC Plus Cement, unconstrained mobile	1,850	1.85	0.08	[-0.50 , 0.66]	Non-inferiority not shown	0.394
[TKR] TC Plus Uncemented, unconstrained mobile	502	2.56	0.79	[-0.26 , 1.84]	Non-inferiority not shown	0.070
[TKR] Triathlon Cement, posterior-stabilised fixed	966	2.94	1.17	[0.60 , 1.74]	Inferior by ≥20%	<0.001
[TKR] Triathlon Cement, unconstrained fixed	2,850	2.49	0.72	[0.37 , 1.06]	Inferior by ≥20%	<0.001
[TKR] Vanguard Cement, unconstrained fixed	1,475	2.44	0.67	[0.25 , 1.10]	Non-inferiority not shown	0.001
[UNI] AMC/Uniglide Unicondylar, mobile	338	14.31	12.54	[9.70 , 15.38]	Inferior by ≥100%	<0.001
[UNI] MG Uni Unicondylar, fixed	840	8.68	6.91	[5.18 , 8.64]	Inferior by ≥100%	<0.001
[UNI] Oxford Partial Knee Unicondylar, mobile	9,099	8.78	7.00	[6.52 , 7.49]	Inferior by ≥100%	<0.001
[UNI] Physica ZUK Unicondylar, fixed	497	6.06	4.29	[3.03 , 5.55]	Inferior by ≥100%	<0.001
[UNI] Preservation Unicondylar, fixed	437	14.95	13.18	[10.18 , 16.18]	Inferior by ≥100%	<0.001
[UNI] Sled Unicondylar, fixed	266	10.30	8.53	[5.51 , 11.55]	Inferior by ≥100%	<0.001

For peer review only

Supplementary table 10: Difference in cumulative percentage revision of knee implants compared to a contemporary benchmark at 10 years post primary in women for knee replacements with ≥250 procedures remaining at risk

Knee brand, bearing and constraint	Number at risk	Cumulative failure (%)	Difference in failure (%)	95% CI	Equivalence status	p-value
[TKR] Genesis 2 Cement, unconstrained fixed	1,175	2.13	[REFERENCE]			
[TKR] AGC Cement, unconstrained fixed	5,614	2.97	0.84	[0.45 , 1.23]	Inferior by ≥20%	<0.001
[TKR] Advance MP Cement, unconstrained fixed	443	4.02	1.90	[0.93 , 2.86]	Inferior by ≥20%	<0.001
[TKR] Genesis 2 Oxinium Cement, unconstrained fixed	259	4.33	2.20	[0.99 , 3.41]	Inferior by ≥20%	<0.001
[TKR] Insall-Burstein 2 Cement, posterior-stabilised fixed	670	4.25	2.13	[0.92 , 3.33]	Inferior by ≥20%	<0.001
[TKR] Kinemax Cement, unconstrained fixed	2,936	4.47	2.35	[1.70 , 2.99]	Inferior by ≥20%	<0.001
[TKR] LCS Cement, unconstrained mobile	269	3.15	1.02	[-0.85 , 2.89]	Non-inferiority not shown	0.142
[TKR] LCS Complete Cement, unconstrained mobile	479	4.29	2.16	[1.34 , 2.98]	Inferior by ≥20%	<0.001
[TKR] LCS Complete Uncemented, unconstrained mobile	738	3.30	1.17	[0.57 , 1.77]	Inferior by ≥20%	<0.001
[TKR] LCS Uncemented, unconstrained mobile	565	2.16	0.04	[-1.06 , 1.13]	Non-inferiority not shown	0.473
[TKR] MRK Cement, unconstrained fixed	341	2.19	0.07	[-0.67 , 0.80]	Non-inferiority not shown	0.431
[TKR] Natural Knee II Cement, unconstrained fixed	362	3.65	1.53	[0.38 , 2.67]	Non-inferiority not shown	0.004
[TKR] NexGen Cement, posterior-stabilised fixed	3,498	3.98	1.85	[1.40 , 2.30]	Inferior by ≥20%	<0.001
[TKR] NexGen Cement, unconstrained fixed	1,721	2.45	0.33	[-0.11 , 0.76]	Non-inferiority not shown	0.069
[TKR] NexGen Uncemented, unconstrained fixed	450	4.30	2.17	[1.08 , 3.26]	Inferior by ≥20%	<0.001
[TKR] PFC Sigma Bicondylar Knee Cement, posterior-stabilised mobile	444	3.87	1.75	[0.88 , 2.61]	Inferior by ≥20%	<0.001
[TKR] PFC Sigma Bicondylar Knee Cement, posterior-stabilised fixed	4,825	2.90	0.78	[0.40 , 1.16]	Non-inferiority not shown	<0.001
[TKR] PFC Sigma Bicondylar Knee Cement, unconstrained fixed	11,524	2.24	0.11	[-0.22 , 0.44]	Non-inferiority not shown	0.258
[TKR] PFC Sigma Bicondylar Knee Cement, unconstrained mobile	467	3.82	1.69	[0.87 , 2.51]	Inferior by ≥20%	<0.001
[TKR] PFC Sigma Bicondylar Knee Hybrid, unconstrained fixed	363	2.06	-0.07	[-1.09 , 0.96]	Non-inferiority not shown	0.450
[TKR] Profix Uncemented, unconstrained fixed	287	2.10	-0.03	[-0.93 , 0.88]	Non-inferiority not shown	0.477
[TKR] Rotaglide + Cement, unconstrained mobile	407	4.62	2.49	[1.01 , 3.97]	Inferior by ≥20%	<0.001
[TKR] Scorpio Cement, posterior-stabilised mobile	274	3.07	0.94	[-0.40 , 2.28]	Non-inferiority not shown	0.084
[TKR] Scorpio Cement, posterior-stabilised fixed	1,201	3.32	1.19	[0.47 , 1.91]	Inferior by ≥20%	0.001
[TKR] Scorpio Cement, unconstrained fixed	1,974	3.41	1.29	[0.70 , 1.87]	Inferior by ≥20%	<0.001
[TKR] Scorpio Uncemented, unconstrained fixed	283	4.01	1.89	[0.69 , 3.08]	Inferior by ≥20%	0.001
[TKR] TC Plus Cement, unconstrained fixed	730	3.03	0.90	[0.23 , 1.57]	Non-inferiority not shown	0.004
[TKR] TC Plus Cement, unconstrained mobile	825	2.40	0.28	[-0.45 , 1.01]	Non-inferiority not shown	0.226
[UNI] MG Uni Unicondylar, fixed	408	11.71	9.58	[7.46 , 11.71]	Inferior by ≥100%	<0.001
[UNI] Oxford Partial Knee Unicondylar, mobile	3,066	12.51	10.38	[9.68 , 11.09]	Inferior by ≥100%	<0.001

For peer review only

Supplementary table 11: Difference in cumulative percentage revision of knee implants compared to a contemporary benchmark at 3 years post primary in men less than 55 years for knee replacements with ≥250 procedures remaining at risk

Knee brand, bearing and constraint	Number at risk	Cumulative failure (%)	Difference in failure (%)	95% CI	Equivalence status	p-value
[TKR] PFC Sigma Bicondylar Knee Cement, unconstrained fixed	2,538	2.85	[REFERENCE]			
[TKR] AGC Cement, unconstrained fixed	915	3.55	0.70	[-0.57 , 1.98]	Non-inferiority not shown	0.140
[TKR] Genesis 2 Cement, unconstrained fixed	311	3.41	0.57	[-1.24 , 2.38]	Non-inferiority not shown	0.270
[TKR] Genesis 2 Oxinium Cement, unconstrained fixed	458	3.15	0.30	[-1.28 , 1.89]	Non-inferiority not shown	0.354
[TKR] LCS Complete Uncemented, unconstrained mobile	331	4.59	1.74	[-0.41 , 3.90]	Non-inferiority not shown	0.057
[TKR] NexGen Cement, posterior-stabilised fixed	761	4.36	1.52	[0.11 , 2.93]	Non-inferiority not shown	0.017
[TKR] NexGen Cement, unconstrained fixed	781	2.92	0.07	[-1.09 , 1.23]	Non-inferiority not shown	0.452
[TKR] PFC Sigma Bicondylar Knee Cement, posterior-stabilised mobile	408	3.30	0.46	[-1.28 , 2.20]	Non-inferiority not shown	0.304
[TKR] PFC Sigma Bicondylar Knee Cement, posterior-stabilised fixed	1,137	3.48	0.64	[-0.49 , 1.76]	Non-inferiority not shown	0.134
[TKR] PFC Sigma Bicondylar Knee Cement, unconstrained mobile	528	3.42	0.57	[-1.01 , 2.15]	Non-inferiority not shown	0.240
[TKR] Triathlon Cement, posterior-stabilised fixed	256	2.79	-0.05	[-1.88 , 1.78]	Non-inferiority not shown	0.477
[TKR] Triathlon Cement, unconstrained fixed	865	2.63	-0.21	[-1.26 , 0.84]	Non-inferiority not shown	0.346
[TKR] Vanguard Cement, unconstrained fixed	584	3.61	0.77	[-0.68 , 2.22]	Non-inferiority not shown	0.150
[UNI] Oxford Partial Knee Unicondylar, mobile	2,693	6.80	3.95	[2.94 , 4.97]	Inferior by ≥100%	<0.001
[UNI] Physica ZUK Unicondylar, fixed	551	2.61	-0.24	[-1.48 , 1.00]	Non-inferiority not shown	0.353
[UNI] Sigma HP Unicondylar, fixed	481	3.99	1.14	[-0.43 , 2.71]	Non-inferiority not shown	0.078

For peer review only

Supplementary table 12: Difference in cumulative percentage revision of knee implants compared to a contemporary benchmark at 5 years post primary in men less than 55 years for knee replacements with ≥250 procedures remaining at risk

Knee brand, bearing and constraint	Number at risk	Cumulative failure (%)	Difference in failure (%)	95% CI	Equivalence status	p-value
[TKR] PFC Sigma Bicondylar Knee Cement, unconstrained fixed	1,753	4.00	[REFERENCE]			
[TKR] AGC Cement, unconstrained fixed	740	5.29	1.29	[-0.30 , 2.88]	Non-inferiority not shown	0.056
[TKR] Genesis 2 Oxinium Cement, unconstrained fixed	325	4.65	0.65	[-1.37 , 2.67]	Non-inferiority not shown	0.263
[TKR] NexGen Cement, posterior-stabilised fixed	520	6.80	2.80	[0.93 , 4.67]	Inferior by ≥20%	0.002
[TKR] NexGen Cement, unconstrained fixed	454	3.75	-0.25	[-1.68 , 1.19]	Non-inferiority not shown	0.368
[TKR] PFC Sigma Bicondylar Knee Cement, posterior-stabilised mobile	306	4.31	0.31	[-1.72 , 2.34]	Non-inferiority not shown	0.383
[TKR] PFC Sigma Bicondylar Knee Cement, posterior-stabilised fixed	796	4.85	0.85	[-0.54 , 2.25]	Non-inferiority not shown	0.115
[TKR] PFC Sigma Bicondylar Knee Cement, unconstrained mobile	415	4.63	0.63	[-1.26 , 2.51]	Non-inferiority not shown	0.258
[TKR] Triathlon Cement, unconstrained fixed	452	3.69	-0.31	[-1.69 , 1.07]	Non-inferiority not shown	0.331
[TKR] Vanguard Cement, unconstrained fixed	255	4.79	0.79	[-0.98 , 2.56]	Non-inferiority not shown	0.190
[UNI] Oxford Partial Knee Unicondylar, mobile	1,972	9.71	5.71	[4.44 , 6.98]	Inferior by ≥100%	<0.001
[UNI] Physica ZUK Unicondylar, fixed	289	4.17	0.17	[-1.57 , 1.92]	Non-inferiority not shown	0.423

For peer review only

Supplementary table 13: Difference in cumulative percentage revision of knee implants compared to a contemporary benchmark at 7 years post primary in men less than 55 years for knee replacements with ≥250 procedures remaining at risk

Knee brand, bearing and constraint	Number at risk	Cumulative failure (%)	Difference in failure (%)	95% CI	Equivalence status	p-value
[TKR] PFC Sigma Bicondylar Knee Cement, unconstrained fixed	1,100	5.11	[REFERENCE]			
[TKR] AGC Cement, unconstrained fixed	500	7.67	2.56	[0.54 , 4.58]	Non-inferiority not shown	0.006
[TKR] NexGen Cement, posterior-stabilised fixed	320	9.92	4.82	[2.32 , 7.31]	Inferior by ≥20%	<0.001
[TKR] PFC Sigma Bicondylar Knee Cement, posterior-stabilised fixed	468	5.92	0.81	[-0.87 , 2.50]	Non-inferiority not shown	0.172
[TKR] PFC Sigma Bicondylar Knee Cement, unconstrained mobile	283	5.48	0.37	[-1.80 , 2.54]	Non-inferiority not shown	0.370
[UNI] Oxford Partial Knee Unicondylar, mobile	1,254	12.89	7.78	[6.21 , 9.35]	Inferior by ≥100%	<0.001

For peer review only

Supplementary table 14: Difference in cumulative percentage revision of knee implants compared to a contemporary benchmark at 3 years post primary in women less than 55 years for knee replacements with ≥250 procedures remaining at risk

Knee brand, bearing and constraint	Number at risk	Cumulative failure (%)	Difference in failure (%)	95% CI	Equivalence status	p-value
[TKR] NexGen Cement, unconstrained fixed	1,105	2.18	[REFERENCE]			
[TKR] AGC Cement, unconstrained fixed	1,331	2.56	0.39	[-0.72 , 1.50]	Non-inferiority not shown	0.248
[TKR] Genesis 2 Cement, unconstrained fixed	472	1.79	-0.38	[-1.65 , 0.89]	Non-inferiority not shown	0.278
[TKR] Genesis 2 Oxinium Cement, posterior-stabilised fixed	301	3.11	0.93	[-0.97 , 2.83]	Non-inferiority not shown	0.168
[TKR] Genesis 2 Oxinium Cement, unconstrained fixed	556	2.66	0.48	[-0.98 , 1.95]	Non-inferiority not shown	0.260
[TKR] Kinemax Cement, unconstrained fixed	266	2.92	0.74	[-1.39 , 2.88]	Non-inferiority not shown	0.248
[TKR] LCS Complete Uncemented, unconstrained mobile	412	3.54	1.36	[-0.46 , 3.19]	Non-inferiority not shown	0.072
[TKR] NexGen Cement, posterior-stabilised fixed	1,253	2.99	0.81	[-0.32 , 1.94]	Non-inferiority not shown	0.080
[TKR] PFC Sigma Bicondylar Knee Cement, posterior-stabilised mobile	436	4.36	2.18	[0.21 , 4.16]	Non-inferiority not shown	0.015
[TKR] PFC Sigma Bicondylar Knee Cement, posterior-stabilised fixed	1,637	3.08	0.90	[-0.18 , 1.99]	Non-inferiority not shown	0.052
[TKR] PFC Sigma Bicondylar Knee Cement, unconstrained fixed	3,827	2.27	0.10	[-0.78 , 0.97]	Non-inferiority not shown	0.415
[TKR] PFC Sigma Bicondylar Knee Cement, unconstrained mobile	520	3.29	1.11	[-0.53 , 2.76]	Non-inferiority not shown	0.092
[TKR] Triathlon Cement, posterior-stabilised fixed	364	3.91	1.74	[-0.17 , 3.64]	Non-inferiority not shown	0.037
[TKR] Triathlon Cement, unconstrained fixed	1,260	2.53	0.35	[-0.71 , 1.42]	Non-inferiority not shown	0.258
[TKR] Vanguard Cement, unconstrained fixed	807	1.39	-0.79	[-1.80 , 0.22]	Non-inferior	0.064
[UNI] Oxford Partial Knee Unicondylar, mobile	3,251	5.92	3.75	[2.70 , 4.80]	Inferior by ≥100%	<0.001
[UNI] Physica ZUK Unicondylar, fixed	526	4.48	2.30	[0.64 , 3.96]	Inferior by ≥20%	0.003
[UNI] Sigma HP Unicondylar, fixed	395	6.28	4.10	[1.96 , 6.25]	Inferior by ≥20%	<0.001

For peer review only

Supplementary table 15: Difference in cumulative percentage revision of knee implants compared to a contemporary benchmark at 5 years post primary in women less than 55 years for knee replacements with ≥250 procedures remaining at risk

Knee brand, bearing and constraint	Number at risk	Cumulative failure (%)	Difference in failure (%)	95% CI	Equivalence status	p-value
[TKR] PFC Sigma Bicondylar Knee Cement, unconstrained fixed	2,522	3.60	[REFERENCE]			
[TKR] AGC Cement, unconstrained fixed	1,072	4.19	0.59	[-0.62 , 1.80]	Non-inferiority not shown	0.168
[TKR] Genesis 2 Cement, unconstrained fixed	282	2.51	-1.09	[-2.51 , 0.32]	Non-inferior	0.065
[TKR] Genesis 2 Oxinium Cement, unconstrained fixed	392	4.68	1.08	[-0.76 , 2.92]	Non-inferiority not shown	0.125
[TKR] Kinemax Cement, unconstrained fixed	258	4.77	1.17	[-1.43 , 3.76]	Non-inferiority not shown	0.189
[TKR] LCS Complete Uncemented, unconstrained mobile	316	5.70	2.10	[-0.17 , 4.37]	Non-inferiority not shown	0.035
[TKR] NexGen Cement, posterior-stabilised fixed	859	4.62	1.02	[-0.23 , 2.27]	Non-inferiority not shown	0.054
[TKR] NexGen Cement, unconstrained fixed	642	3.94	0.34	[-0.92 , 1.60]	Non-inferiority not shown	0.300
[TKR] PFC Sigma Bicondylar Knee Cement, posterior-stabilised mobile	333	5.51	1.91	[-0.23 , 4.06]	Non-inferiority not shown	0.040
[TKR] PFC Sigma Bicondylar Knee Cement, posterior-stabilised fixed	1,153	4.93	1.33	[0.16 , 2.51]	Non-inferiority not shown	0.013
[TKR] PFC Sigma Bicondylar Knee Cement, unconstrained mobile	423	5.73	2.13	[0.08 , 4.18]	Non-inferiority not shown	0.021
[TKR] Triathlon Cement, unconstrained fixed	681	4.15	0.55	[-0.68 , 1.77]	Non-inferiority not shown	0.191
[TKR] Vanguard Cement, unconstrained fixed	341	2.57	-1.03	[-2.27 , 0.21]	Non-inferior	0.052
[UNI] Oxford Partial Knee Unicondylar, mobile	2,358	9.86	6.26	[5.12 , 7.39]	Inferior by ≥100%	<0.001
[UNI] Physica ZUK Unicondylar, fixed	256	6.21	2.61	[0.65 , 4.57]	Non-inferiority not shown	0.005

For peer review only

Supplementary table 16: Difference in cumulative percentage revision of knee implants compared to a contemporary benchmark at 7 years post primary in women less than 55 years for knee replacements with ≥250 procedures remaining at risk

Knee brand, bearing and constraint	Number at risk	Cumulative failure (%)	Difference in failure (%)	95% CI	Equivalence status	p-value
[TKR] PFC Sigma Bicondylar Knee Cement, unconstrained fixed	1,538	4.39	[REFERENCE]			
[TKR] AGC Cement, unconstrained fixed	735	5.15	0.75	[-0.65 , 2.16]	Non-inferiority not shown	0.147
[TKR] NexGen Cement, posterior-stabilised fixed	551	6.60	2.21	[0.55 , 3.86]	Non-inferiority not shown	0.004
[TKR] NexGen Cement, unconstrained fixed	334	5.20	0.81	[-0.84 , 2.46]	Non-inferiority not shown	0.169
[TKR] PFC Sigma Bicondylar Knee Cement, posterior-stabilised fixed	720	5.57	1.18	[-0.16 , 2.51]	Non-inferiority not shown	0.042
[TKR] PFC Sigma Bicondylar Knee Cement, unconstrained mobile	285	7.06	2.67	[0.30 , 5.03]	Non-inferiority not shown	0.014
[TKR] Triathlon Cement, unconstrained fixed	268	5.80	1.40	[-0.32 , 3.13]	Non-inferiority not shown	0.056
[UNI] Oxford Partial Knee Unicondylar, mobile	1,495	13.01	8.62	[7.24 , 10.00]	Inferior by ≥100%	<0.001

For peer review only

Supplementary table 17: Difference in cumulative percentage revision of knee implants compared to a contemporary benchmark at 3 years post primary in men between 55 and 75 years for knee replacements with ≥250 procedures remaining at risk

Knee brand, bearing and constraint	Number at risk	Cumulative failure (%)	Difference in failure (%)	95% CI	Equivalence status	p-value
[TKR] NexGen Cement, unconstrained fixed	10,041	1.34	[REFERENCE]			
[TKR] AGC Cement, unconstrained fixed	14,760	1.71	0.37	[0.09 , 0.65]	Non-inferiority not shown	0.005
[TKR] AGC Hybrid, unconstrained fixed	274	2.77	1.43	[-0.47 , 3.33]	Non-inferiority not shown	0.070
[TKR] AGC Uncemented, unconstrained fixed	402	3.74	2.40	[0.59 , 4.21]	Inferior by ≥20%	0.005
[TKR] Advance MP Cement, unconstrained fixed	1,736	2.43	1.09	[0.41 , 1.77]	Inferior by ≥20%	0.001
[TKR] Columbus Cement, unconstrained fixed	1,543	1.73	0.39	[-0.20 , 0.97]	Non-inferiority not shown	0.096
[TKR] E-Motion Bicondylar Knee Uncemented, unconstrained mobile	516	1.61	0.27	[-0.74 , 1.29]	Non-inferiority not shown	0.299
[TKR] Genesis 2 Cement, posterior-stabilised fixed	2,118	2.39	1.05	[0.45 , 1.64]	Inferior by ≥20%	<0.001
[TKR] Genesis 2 Cement, unconstrained fixed	7,447	1.61	0.27	[-0.05 , 0.59]	Non-inferiority not shown	0.047
[TKR] Genesis 2 Oxinium Cement, posterior-stabilised fixed	447	3.07	1.73	[0.27 , 3.19]	Non-inferiority not shown	0.010
[TKR] Genesis 2 Oxinium Cement, unconstrained fixed	1,105	2.22	0.88	[0.04 , 1.72]	Non-inferiority not shown	0.020
[TKR] Insall-Burstein 2 Cement, posterior-stabilised fixed	684	2.25	0.91	[-0.20 , 2.01]	Non-inferiority not shown	0.054
[TKR] Journey Oxinium Cement, posterior-stabilised fixed	252	3.43	2.09	[-0.12 , 4.29]	Non-inferiority not shown	0.032
[TKR] Kinemax Cement, unconstrained fixed	2,948	2.12	0.78	[0.23 , 1.32]	Non-inferiority not shown	0.003
[TKR] LCS Complete Cement, unconstrained mobile	2,624	1.76	0.42	[-0.09 , 0.94]	Non-inferiority not shown	0.053
[TKR] LCS Complete Uncemented, unconstrained mobile	3,377	1.87	0.53	[0.07 , 0.99]	Non-inferiority not shown	0.012
[TKR] LCS Uncemented, unconstrained mobile	376	2.31	0.97	[-0.53 , 2.47]	Non-inferiority not shown	0.103
[TKR] MRK Cement, unconstrained fixed	1,730	1.62	0.28	[-0.29 , 0.84]	Non-inferiority not shown	0.166
[TKR] Maxim Cement, unconstrained fixed	406	1.44	0.10	[-1.06 , 1.26]	Non-inferiority not shown	0.432
[TKR] NRG Cement, posterior-stabilised fixed	1,179	1.84	0.50	[-0.25 , 1.26]	Non-inferiority not shown	0.096
[TKR] NRG Cement, unconstrained fixed	1,693	2.06	0.72	[0.07 , 1.38]	Non-inferiority not shown	0.015
[TKR] Natural Knee II Cement, unconstrained fixed	712	1.33	-0.01	[-0.85 , 0.83]	Non-inferiority not shown	0.490
[TKR] NexGen Cement, posterior-stabilised fixed	11,497	2.14	0.80	[0.49 , 1.11]	Inferior by ≥20%	<0.001
[TKR] NexGen Hybrid, unconstrained fixed	332	0.56	-0.78	[-1.58 , 0.01]	Non-inferior	0.027
[TKR] NexGen Uncemented, posterior-stabilised fixed	564	1.27	-0.07	[-0.92 , 0.78]	Non-inferiority not shown	0.437
[TKR] NexGen Uncemented, unconstrained fixed	1,469	2.24	0.90	[0.15 , 1.65]	Non-inferiority not shown	0.009
[TKR] Optetrak Cement, posterior-stabilised fixed	417	2.72	1.38	[-0.15 , 2.92]	Non-inferiority not shown	0.038
[TKR] PFC Sigma Bicondylar Knee Cement, posterior-stabilised mobile	1,927	1.93	0.60	[-0.02 , 1.21]	Non-inferiority not shown	0.028
[TKR] PFC Sigma Bicondylar Knee Cement, posterior-stabilised fixed	15,069	1.72	0.38	[0.11 , 0.66]	Non-inferiority not shown	0.003
[TKR] PFC Sigma Bicondylar Knee Cement, unconstrained fixed	37,425	1.49	0.15	[-0.07 , 0.38]	Non-inferiority not shown	0.089
[TKR] PFC Sigma Bicondylar Knee Cement, unconstrained mobile	2,249	1.94	0.60	[0.02 , 1.17]	Non-inferiority not shown	0.021
[TKR] PFC Sigma Bicondylar Knee Hybrid, unconstrained fixed	499	0.97	-0.37	[-1.24 , 0.50]	Non-inferiority not shown	0.202
[TKR] PFC Sigma Bicondylar Knee monobloc polyethylene tibia	1,086	1.39	0.05	[-0.56 , 0.66]	Non-inferiority not shown	0.439
[TKR] Profix Cement, unconstrained fixed	304	2.18	0.84	[-0.77 , 2.44]	Non-inferiority not shown	0.154
[TKR] Profix Uncemented, unconstrained fixed	631	1.23	-0.11	[-0.98 , 0.75]	Non-inferiority not shown	0.399
[TKR] Rotaglide + Cement, unconstrained mobile	468	4.00	2.67	[0.93 , 4.40]	Inferior by ≥20%	0.001
[TKR] Rotaglide Cement, unconstrained mobile	264	3.61	2.27	[0.17 , 4.38]	Non-inferiority not shown	0.017
[TKR] Scorpio Cement, posterior-stabilised mobile	388	1.98	0.64	[-0.73 , 2.02]	Non-inferiority not shown	0.179
[TKR] Scorpio Cement, posterior-stabilised fixed	1,531	2.37	1.03	[0.26 , 1.81]	Non-inferiority not shown	0.004
[TKR] Scorpio Cement, unconstrained fixed	2,891	2.60	1.27	[0.67 , 1.86]	Inferior by ≥20%	<0.001

For peer review only

[TKR] Scorpio Cement, unconstrained mobile	341	1.97	0.63	[-0.83 , 2.09]	Non-inferiority not shown	0.198
[TKR] Scorpio Hybrid, unconstrained fixed	269	2.13	0.79	[-0.91 , 2.49]	Non-inferiority not shown	0.181
[TKR] Scorpio Uncemented, unconstrained fixed	1,184	1.96	0.63	[-0.18 , 1.43]	Non-inferiority not shown	0.063
[TKR] TC Plus Cement, unconstrained fixed	2,427	2.45	1.11	[0.48 , 1.75]	Inferior by ≥20%	<0.001
[TKR] TC Plus Cement, unconstrained mobile	1,442	2.13	0.79	[0.05 , 1.54]	Non-inferiority not shown	0.019
[TKR] TC Plus Uncemented, unconstrained mobile	373	1.03	-0.30	[-1.23 , 0.62]	Non-inferiority not shown	0.260
[TKR] Triathlon Cement, posterior-stabilised fixed	2,263	2.00	0.66	[0.14 , 1.18]	Non-inferiority not shown	0.006
[TKR] Triathlon Cement, unconstrained fixed	8,595	1.44	0.10	[-0.18 , 0.39]	Non-inferiority not shown	0.244
[TKR] Vanguard Cement, posterior-stabilised fixed	833	1.96	0.62	[-0.14 , 1.38]	Non-inferiority not shown	0.054
[TKR] Vanguard Cement, unconstrained fixed	5,651	1.50	0.16	[-0.17 , 0.48]	Non-inferiority not shown	0.169
[UNI] AMC/Uniglide Unicondylar, fixed	335	2.93	1.59	[-0.13 , 3.30]	Non-inferiority not shown	0.035
[UNI] AMC/Uniglide Unicondylar, mobile	458	8.38	7.04	[4.69 , 9.40]	Inferior by ≥100%	<0.001
[UNI] MG Uni Unicondylar, fixed	888	3.13	1.79	[0.65 , 2.93]	Inferior by ≥20%	0.001
[UNI] Oxford Partial Knee Unicondylar, mobile	14,444	3.69	2.35	[2.01 , 2.69]	Inferior by ≥100%	<0.001
[UNI] Physica ZUK Unicondylar, fixed	1,765	2.33	0.99	[0.36 , 1.62]	Inferior by ≥20%	0.001
[UNI] Preservation Unicondylar, fixed	455	4.16	2.82	[1.03 , 4.62]	Inferior by ≥20%	0.001
[UNI] Sigma HP Unicondylar, fixed	1,404	2.97	1.64	[0.89 , 2.38]	Inferior by ≥20%	<0.001

For peer review only

Supplementary table 18: Difference in cumulative percentage revision of knee implants compared to a contemporary benchmark at 5 years post primary in men between 55 and 75 years for knee replacements with ≥250 procedures remaining at risk

Knee brand, bearing and constraint	Number at risk	Cumulative failure (%)	Difference in failure (%)	95% CI	Equivalence status	p-value
[TKR] NexGen Cement, unconstrained fixed	6,119	1.92	[REFERENCE]			
[TKR] AGC Cement, unconstrained fixed	11,861	2.48	0.56	[0.21 , 0.91]	Non-inferiority not shown	0.001
[TKR] AGC Hybrid, unconstrained fixed	258	3.14	1.23	[-0.81 , 3.27]	Non-inferiority not shown	0.119
[TKR] AGC Uncemented, unconstrained fixed	350	4.52	2.60	[0.60 , 4.60]	Inferior by ≥20%	0.005
[TKR] Advance MP Cement, unconstrained fixed	1,248	3.28	1.37	[0.53 , 2.20]	Inferior by ≥20%	0.001
[TKR] Columbus Cement, unconstrained fixed	879	2.61	0.70	[-0.10 , 1.49]	Non-inferiority not shown	0.044
[TKR] E-Motion Bicondylar Knee Uncemented, unconstrained mobile	382	2.28	0.36	[-0.90 , 1.63]	Non-inferiority not shown	0.287
[TKR] Genesis 2 Cement, posterior-stabilised fixed	1,221	3.38	1.47	[0.69 , 2.24]	Inferior by ≥20%	<0.001
[TKR] Genesis 2 Cement, unconstrained fixed	4,599	2.39	0.47	[0.05 , 0.89]	Non-inferiority not shown	0.014
[TKR] Genesis 2 Oxinium Cement, posterior-stabilised fixed	259	4.21	2.29	[0.45 , 4.13]	Inferior by ≥20%	0.007
[TKR] Genesis 2 Oxinium Cement, unconstrained fixed	841	2.94	1.02	[0.02 , 2.03]	Non-inferiority not shown	0.023
[TKR] Insall-Burstein 2 Cement, posterior-stabilised fixed	643	3.70	1.79	[0.37 , 3.21]	Non-inferiority not shown	0.007
[TKR] Kinemax Cement, unconstrained fixed	2,804	3.37	1.46	[0.76 , 2.15]	Inferior by ≥20%	<0.001
[TKR] LCS Complete Cement, unconstrained mobile	2,077	3.22	1.30	[0.58 , 2.02]	Inferior by ≥20%	<0.001
[TKR] LCS Complete Uncemented, unconstrained mobile	2,561	2.43	0.51	[-0.04 , 1.07]	Non-inferiority not shown	0.035
[TKR] LCS Uncemented, unconstrained mobile	359	3.10	1.19	[-0.56 , 2.93]	Non-inferiority not shown	0.092
[TKR] MRK Cement, unconstrained fixed	1,182	2.35	0.43	[-0.30 , 1.16]	Non-inferiority not shown	0.123
[TKR] Maxim Cement, unconstrained fixed	375	2.19	0.27	[-1.17 , 1.71]	Non-inferiority not shown	0.356
[TKR] NRG Cement, posterior-stabilised fixed	893	2.58	0.66	[-0.26 , 1.58]	Non-inferiority not shown	0.080
[TKR] NRG Cement, unconstrained fixed	1,162	3.42	1.51	[0.61 , 2.40]	Inferior by ≥20%	<0.001
[TKR] Natural Knee II Cement, unconstrained fixed	563	1.91	-0.01	[-1.03 , 1.02]	Non-inferiority not shown	0.496
[TKR] NexGen Cement, posterior-stabilised fixed	8,138	3.17	1.25	[0.85 , 1.65]	Inferior by ≥20%	<0.001
[TKR] NexGen Hybrid, unconstrained fixed	282	1.47	-0.45	[-1.75 , 0.86]	Non-inferiority not shown	0.251
[TKR] NexGen Uncemented, posterior-stabilised fixed	415	1.69	-0.22	[-1.27 , 0.82]	Non-inferiority not shown	0.336
[TKR] NexGen Uncemented, unconstrained fixed	1,234	2.97	1.05	[0.17 , 1.94]	Non-inferiority not shown	0.010
[TKR] Optetrak Cement, posterior-stabilised fixed	380	4.38	2.47	[0.52 , 4.41]	Inferior by ≥20%	0.006
[TKR] PFC Sigma Bicondylar Knee Cement, posterior-stabilised mobile	1,449	2.32	0.41	[-0.29 , 1.10]	Non-inferiority not shown	0.125
[TKR] PFC Sigma Bicondylar Knee Cement, posterior-stabilised fixed	10,973	2.29	0.37	[0.03 , 0.72]	Non-inferiority not shown	0.016
[TKR] PFC Sigma Bicondylar Knee Cement, unconstrained fixed	26,348	1.97	0.05	[-0.24 , 0.34]	Non-inferior	0.364
[TKR] PFC Sigma Bicondylar Knee Cement, unconstrained mobile	1,860	2.36	0.45	[-0.21 , 1.11]	Non-inferiority not shown	0.092
[TKR] PFC Sigma Bicondylar Knee Hybrid, unconstrained fixed	454	1.60	-0.32	[-1.45 , 0.81]	Non-inferiority not shown	0.289
[TKR] PFC Sigma Bicondylar Knee monobloc polyethylene tibia	458	1.75	-0.16	[-0.92 , 0.59]	Non-inferiority not shown	0.336
[TKR] Profix Cement, unconstrained fixed	283	4.50	2.59	[0.27 , 4.91]	Non-inferiority not shown	0.014
[TKR] Profix Uncemented, unconstrained fixed	583	1.38	-0.53	[-1.47 , 0.40]	Non-inferiority not shown	0.131
[TKR] Rotaglide + Cement, unconstrained mobile	444	4.84	2.92	[1.02 , 4.83]	Inferior by ≥20%	0.001
[TKR] Scorpio Cement, posterior-stabilised mobile	366	3.00	1.08	[-0.61 , 2.77]	Non-inferiority not shown	0.105
[TKR] Scorpio Cement, posterior-stabilised fixed	1,441	3.55	1.63	[0.68 , 2.58]	Inferior by ≥20%	<0.001
[TKR] Scorpio Cement, unconstrained fixed	2,655	3.57	1.66	[0.94 , 2.37]	Inferior by ≥20%	<0.001
[TKR] Scorpio Cement, unconstrained mobile	325	3.43	1.51	[-0.41 , 3.43]	Non-inferiority not shown	0.062
[TKR] Scorpio Hybrid, unconstrained fixed	258	2.13	0.21	[-1.49 , 1.92]	Non-inferiority not shown	0.403

For peer review only

[TKR] Scorpio Uncemented, unconstrained fixed	1,053	2.82	0.90	[-0.07 , 1.87]	Non-inferiority not shown	0.034
[TKR] TC Plus Cement, unconstrained fixed	2,283	3.23	1.31	[0.58 , 2.05]	Inferior by ≥20%	<0.001
[TKR] TC Plus Cement, unconstrained mobile	1,340	2.89	0.97	[0.09 , 1.85]	Non-inferiority not shown	0.015
[TKR] TC Plus Uncemented, unconstrained mobile	303	1.58	-0.34	[-1.54 , 0.86]	Non-inferiority not shown	0.290
[TKR] Triathlon Cement, posterior-stabilised fixed	1,289	2.64	0.73	[0.07 , 1.38]	Non-inferiority not shown	0.015
[TKR] Triathlon Cement, unconstrained fixed	4,224	1.95	0.04	[-0.33 , 0.41]	Non-inferiority not shown	0.423
[TKR] Vanguard Cement, posterior-stabilised fixed	326	2.90	0.98	[-0.10 , 2.07]	Non-inferiority not shown	0.037
[TKR] Vanguard Cement, unconstrained fixed	2,489	2.10	0.18	[-0.25 , 0.61]	Non-inferiority not shown	0.207
[UNI] AMC/Uniglide Unicondylar, mobile	381	9.21	7.30	[4.82 , 9.77]	Inferior by ≥100%	<0.001
[UNI] MG Uni Unicondylar, fixed	845	4.35	2.43	[1.09 , 3.77]	Inferior by ≥20%	<0.001
[UNI] Oxford Partial Knee Unicondylar, mobile	10,935	5.34	3.42	[2.99 , 3.86]	Inferior by ≥100%	<0.001
[UNI] Physica ZUK Unicondylar, fixed	890	3.82	1.91	[0.99 , 2.83]	Inferior by ≥20%	<0.001
[UNI] Preservation Unicondylar, fixed	428	7.58	5.66	[3.27 , 8.06]	Inferior by ≥100%	<0.001
[UNI] Sigma HP Unicondylar, fixed	642	4.53	2.61	[1.55 , 3.67]	Inferior by ≥20%	<0.001

For peer review only

Supplementary table 19: Difference in cumulative percentage revision of knee implants compared to a contemporary benchmark at 7 years post primary in men between 55 and 75 years for knee replacements with ≥250 procedures remaining at risk

Knee brand, bearing and constraint	Number at risk	Cumulative failure (%)	Difference in failure (%)	95% CI	Equivalence status	p-value
[TKR] PFC Sigma Bicondylar Knee Cement, unconstrained fixed	16,697	2.31	[REFERENCE]			
[TKR] AGC Cement, unconstrained fixed	8,081	3.16	0.85	[0.52 , 1.18]	Inferior by ≥20%	<0.001
[TKR] Advance MP Cement, unconstrained fixed	777	3.82	1.51	[0.60 , 2.43]	Inferior by ≥20%	0.001
[TKR] Columbus Cement, unconstrained fixed	462	2.89	0.58	[-0.28 , 1.45]	Non-inferiority not shown	0.093
[TKR] E-Motion Bicondylar Knee Uncemented, unconstrained mobile	270	2.88	0.57	[-0.92 , 2.07]	Non-inferiority not shown	0.227
[TKR] Genesis 2 Cement, posterior-stabilised fixed	641	4.09	1.78	[0.87 , 2.70]	Inferior by ≥20%	<0.001
[TKR] Genesis 2 Cement, unconstrained fixed	2,429	2.96	0.65	[0.21 , 1.09]	Non-inferiority not shown	0.002
[TKR] Genesis 2 Oxinium Cement, unconstrained fixed	537	3.34	1.03	[-0.04 , 2.11]	Non-inferiority not shown	0.030
[TKR] Insall-Burstein 2 Cement, posterior-stabilised fixed	604	4.47	2.16	[0.61 , 3.71]	Inferior by ≥20%	0.003
[TKR] Kinemax Cement, unconstrained fixed	2,644	4.22	1.91	[1.17 , 2.65]	Inferior by ≥20%	<0.001
[TKR] LCS Complete Cement, unconstrained mobile	1,342	3.84	1.53	[0.76 , 2.30]	Inferior by ≥20%	<0.001
[TKR] LCS Complete Uncemented, unconstrained mobile	1,584	2.97	0.66	[0.05 , 1.27]	Non-inferiority not shown	0.016
[TKR] LCS Uncemented, unconstrained mobile	345	3.10	0.80	[-0.94 , 2.53]	Non-inferiority not shown	0.184
[TKR] MRK Cement, unconstrained fixed	762	3.31	1.00	[0.09 , 1.92]	Non-inferiority not shown	0.016
[TKR] Maxim Cement, unconstrained fixed	314	3.08	0.77	[-0.96 , 2.50]	Non-inferiority not shown	0.192
[TKR] NRG Cement, posterior-stabilised fixed	413	3.08	0.77	[-0.25 , 1.79]	Non-inferiority not shown	0.070
[TKR] NRG Cement, unconstrained fixed	666	3.68	1.37	[0.43 , 2.32]	Non-inferiority not shown	0.002
[TKR] Natural Knee II Cement, unconstrained fixed	417	3.49	1.18	[-0.29 , 2.66]	Non-inferiority not shown	0.057
[TKR] Nexgen Cement, posterior-stabilised fixed	5,295	4.16	1.85	[1.44 , 2.27]	Inferior by ≥20%	<0.001
[TKR] Nexgen Cement, unconstrained fixed	3,522	2.48	0.17	[-0.19 , 0.53]	Non-inferiority not shown	0.180
[TKR] Nexgen Uncemented, ps fixed	313	2.01	-0.30	[-1.49 , 0.89]	Non-inferiority not shown	0.309
[TKR] Nexgen Uncemented, unconstrained fixed	968	3.30	0.99	[0.07 , 1.91]	Non-inferiority not shown	0.017
[TKR] PFC Sigma Bicondylar Knee Cement, PS mobile	915	2.80	0.49	[-0.27 , 1.26]	Non-inferiority not shown	0.103
[TKR] PFC Sigma Bicondylar Knee Cement, posterior-stabilised fixed	6,928	2.64	0.33	[0.03 , 0.63]	Non-inferiority not shown	0.016
[TKR] PFC Sigma Bicondylar Knee Cement, unconstrained mobile	1,328	2.85	0.54	[-0.17 , 1.25]	Non-inferiority not shown	0.067
[TKR] PFC Sigma Bicondylar Knee Hybrid, unconstrained fixed	391	1.60	-0.71	[-1.82 , 0.40]	Non-inferior	0.104
[TKR] Profix Cement, unconstrained fixed	251	5.23	2.92	[0.41 , 5.42]	Non-inferiority not shown	0.011
[TKR] Profix Uncemented, unconstrained fixed	469	1.95	-0.36	[-1.47 , 0.74]	Non-inferiority not shown	0.260
[TKR] Rotaglide + Cement, unconstrained mobile	377	5.78	3.47	[1.38 , 5.56]	Inferior by ≥20%	0.001
[TKR] Scorpio Cement, PS mobile	320	3.00	0.69	[-0.99 , 2.37]	Non-inferiority not shown	0.210
[TKR] Scorpio Cement, posterior-stabilised fixed	1,320	4.58	2.27	[1.22 , 3.33]	Inferior by ≥20%	<0.001
[TKR] Scorpio Cement, unconstrained fixed	2,216	4.36	2.05	[1.29 , 2.81]	Inferior by ≥20%	<0.001
[TKR] Scorpio Cement, unconstrained mobile	302	4.34	2.03	[-0.12 , 4.19]	Non-inferiority not shown	0.032
[TKR] Scorpio Uncemented, unconstrained fixed	740	3.50	1.19	[0.10 , 2.28]	Non-inferiority not shown	0.016
[TKR] TC Plus Cement, unconstrained fixed	1,784	3.69	1.38	[0.62 , 2.15]	Inferior by ≥20%	<0.001
[TKR] TC Plus Cement, unconstrained mobile	1,101	3.50	1.20	[0.24 , 2.15]	Non-inferiority not shown	0.007
[TKR] Triathlon Cement, posterior-stabilised fixed	443	2.81	0.50	[-0.16 , 1.16]	Non-inferiority not shown	0.070
[TKR] Triathlon Cement, unconstrained fixed	1,424	2.43	0.12	[-0.29 , 0.54]	Non-inferiority not shown	0.280
[TKR] Vanguard Cement, unconstrained fixed	749	2.28	-0.03	[-0.46 , 0.40]	Non-inferior	0.449
[UNI] AMC/Uniglide Unicondylar, mobile	253	11.09	8.78	[5.99 , 11.56]	Inferior by ≥100%	<0.001

For peer review only

[UNI] MG Uni Unicondylar, fixed	753	5.64	3.33	[1.82 , 4.85]	Inferior by ≥20%	<0.001
[UNI] Oxford Partial Knee Unicondylar, mobile	7,296	6.84	4.54	[4.09 , 4.98]	Inferior by ≥100%	<0.001
[UNI] Physica ZUK Unicondylar, fixed	432	4.54	2.23	[1.14 , 3.32]	Inferior by ≥20%	<0.001
[UNI] Preservation Unicondylar, fixed	388	9.77	7.46	[4.77 , 10.15]	Inferior by ≥100%	<0.001

For peer review only

Supplementary table 20: Difference in cumulative percentage revision of knee implants compared to a contemporary benchmark at 10 years post primary in men between 55 and 75 years for knee replacements with ≥250 procedures remaining at risk

Knee brand, bearing and constraint	Number at risk	Cumulative failure (%)	Difference in failure (%)	95% CI	Equivalence status	p-value
[TKR] PFC Sigma Bicondylar Knee Cement, unconstrained fixed	6,049	2.72	[REFERENCE]			
[TKR] AGC Cement, unconstrained fixed	2,803	4.43	1.71	[1.24 , 2.18]	Inferior by ≥20%	<0.001
[TKR] Genesis 2 Cement, unconstrained fixed	621	3.54	0.82	[0.22 , 1.42]	Non-inferiority not shown	0.004
[TKR] Insall-Burstein 2 Cement, posterior-stabilised fixed	362	7.30	4.58	[2.50 , 6.66]	Inferior by ≥20%	<0.001
[TKR] Kinemax Cement, unconstrained fixed	1,648	5.26	2.54	[1.69 , 3.38]	Inferior by ≥20%	<0.001
[TKR] LCS Complete Uncemented, unconstrained mobile	471	3.52	0.80	[0.06 , 1.54]	Non-inferiority not shown	0.017
[TKR] LCS Uncemented, unconstrained mobile	317	3.10	0.38	[-1.36 , 2.12]	Non-inferiority not shown	0.333
[TKR] Nexgen Cement, posterior-stabilised fixed	1,814	5.64	2.92	[2.33 , 3.51]	Inferior by ≥20%	<0.001
[TKR] Nexgen Cement, unconstrained fixed	890	2.85	0.13	[-0.34 , 0.61]	Non-inferiority not shown	0.293
[TKR] Nexgen Uncemented, unconstrained fixed	265	3.42	0.70	[-0.25 , 1.66]	Non-inferiority not shown	0.075
[TKR] PFC Sigma Bicondylar Knee Cement, posterior-stabilised fixed	2,369	3.13	0.41	[0.03 , 0.78]	Non-inferiority not shown	0.018
[TKR] PFC Sigma Bicondylar Knee Cement, unconstrained mobile	322	3.19	0.47	[-0.32 , 1.26]	Non-inferiority not shown	0.122
[TKR] Scorpio Cement, posterior-stabilised fixed	611	5.47	2.75	[1.56 , 3.95]	Inferior by ≥20%	<0.001
[TKR] Scorpio Cement, unconstrained fixed	1,044	4.99	2.27	[1.43 , 3.12]	Inferior by ≥20%	<0.001
[TKR] TC Plus Cement, unconstrained fixed	456	4.51	1.79	[0.81 , 2.78]	Inferior by ≥20%	<0.001
[TKR] TC Plus Cement, unconstrained mobile	527	4.48	1.76	[0.61 , 2.90]	Inferior by ≥20%	0.001
[UNI] MG Uni Unicondylar, fixed	346	7.79	5.07	[3.18 , 6.97]	Inferior by ≥100%	<0.001
[UNI] Oxford Partial Knee Unicondylar, mobile	2,454	9.97	7.25	[6.57 , 7.93]	Inferior by ≥100%	<0.001

For peer review only

Supplementary table 21: Difference in cumulative percentage revision of knee implants compared to a contemporary benchmark at 3 years post primary in women between 55 and 75 years for knee replacements with ≥250 procedures remaining at risk

Knee brand, bearing and constraint	Number at risk	Cumulative failure (%)	Difference in failure (%)	95% CI	Equivalence status	p-value
[TKR] TC Plus Cement, unconstrained mobile	1,695	1.000	[REFERENCE]			
[TKR] AGC Cement, unconstrained fixed	18,655	1.500	0.510	[0.02 , 0.99]	Non-inferiority not shown	0.021
[TKR] AGC Hybrid, unconstrained fixed	297	2.600	1.600	[-0.24 , 3.43]	Non-inferiority not shown	0.044
[TKR] AGC Uncemented, unconstrained fixed	377	5.720	4.730	[2.41 , 7.04]	Inferior by ≥100%	<0.001
[TKR] Advance MP Cement, unconstrained fixed	2,023	1.800	0.810	[0.10 , 1.52]	Non-inferiority not shown	0.013
[TKR] Advance MP Stature Cement, unconstrained fixed	494	1.540	0.540	[-0.51 , 1.59]	Non-inferiority not shown	0.158
[TKR] Advance PS Cement, posterior-stabilised fixed	282	2.730	1.730	[-0.09 , 3.55]	Non-inferiority not shown	0.031
[TKR] Columbus Cement, unconstrained fixed	1,895	1.810	0.810	[0.12 , 1.50]	Non-inferiority not shown	0.011
[TKR] E-Motion Bicondylar Knee Cement, unconstrained mobile	311	3.320	2.320	[0.47 , 4.16]	Inferior by ≥20%	0.007
[TKR] E-Motion Bicondylar Knee Uncemented, unconstrained mobile	495	2.220	1.220	[-0.06 , 2.51]	Non-inferiority not shown	0.031
[TKR] FS Cement, unconstrained fixed	253	1.160	0.160	[-1.22 , 1.55]	Non-inferiority not shown	0.408
[TKR] Genesis 2 Cement, posterior-stabilised fixed	3,026	1.770	0.770	[0.16 , 1.39]	Non-inferiority not shown	0.006
[TKR] Genesis 2 Cement, unconstrained fixed	9,401	1.200	0.200	[-0.30 , 0.70]	Non-inferiority not shown	0.213
[TKR] Genesis 2 Cement, unconstrained mobile	331	1.480	0.480	[-0.89 , 1.85]	Non-inferiority not shown	0.245
[TKR] Genesis 2 Oxinium Cement, posterior-stabilised fixed	615	2.210	1.210	[0.09 , 2.32]	Non-inferiority not shown	0.017
[TKR] Genesis 2 Oxinium Cement, unconstrained fixed	1,426	1.160	0.160	[-0.52 , 0.84]	Non-inferiority not shown	0.324
[TKR] Insall-Burstein 2 Cement, posterior-stabilised fixed	812	1.450	0.450	[-0.49 , 1.38]	Non-inferiority not shown	0.174
[TKR] Journey Oxinium Cement, posterior-stabilised fixed	311	4.310	3.310	[1.05 , 5.56]	Inferior by ≥100%	0.002
[TKR] Kinemax Cement, unconstrained fixed	3,607	2.160	1.170	[0.51 , 1.82]	Inferior by ≥20%	<0.001
[TKR] LCS Complete Cement, unconstrained mobile	3,170	1.850	0.850	[0.21 , 1.49]	Inferior by ≥20%	0.005
[TKR] LCS Complete Uncemented, unconstrained mobile	3,736	1.750	0.750	[0.15 , 1.35]	Non-inferiority not shown	0.007
[TKR] LCS Uncemented, unconstrained mobile	458	2.330	1.330	[-0.11 , 2.76]	Non-inferiority not shown	0.035
[TKR] MRK Cement, unconstrained fixed	2,339	1.070	0.070	[-0.52 , 0.66]	Non-inferiority not shown	0.407
[TKR] Maxim Cement, unconstrained fixed	478	1.840	0.840	[-0.44 , 2.11]	Non-inferiority not shown	0.099
[TKR] NRG Cement, posterior-stabilised fixed	1,574	2.030	1.030	[0.21 , 1.84]	Inferior by ≥20%	0.007
[TKR] NRG Cement, unconstrained fixed	2,181	1.410	0.410	[-0.24 , 1.06]	Non-inferiority not shown	0.107
[TKR] Natural Knee II Cement, unconstrained fixed	965	2.070	1.070	[0.08 , 2.06]	Non-inferiority not shown	0.017
[TKR] NexGen Cement, posterior-stabilised mobile	324	2.070	1.070	[-0.42 , 2.57]	Non-inferiority not shown	0.080
[TKR] NexGen Cement, posterior-stabilised fixed	15,772	1.490	0.490	[-0.00 , 0.98]	Non-inferiority not shown	0.025
[TKR] NexGen Cement, unconstrained fixed	13,193	1.030	0.030	[-0.45 , 0.52]	Non-inferiority not shown	0.447
[TKR] NexGen Hybrid, unconstrained fixed	333	1.150	0.160	[-1.06 , 1.37]	Non-inferiority not shown	0.401
[TKR] NexGen Uncemented, posterior-stabilised fixed	419	1.420	0.420	[-0.72 , 1.57]	Non-inferiority not shown	0.235
[TKR] NexGen Uncemented, unconstrained fixed	1,110	2.720	1.720	[0.69 , 2.76]	Inferior by ≥20%	0.001
[TKR] Optetrak Cement, posterior-stabilised fixed	579	3.140	2.140	[0.68 , 3.60]	Inferior by ≥20%	0.002
[TKR] PFC Sigma Bicondylar Knee Cement, posterior-stabilised mobile	2,132	1.940	0.940	[0.22 , 1.65]	Inferior by ≥20%	0.005
[TKR] PFC Sigma Bicondylar Knee Cement, posterior-stabilised fixed	20,665	1.600	0.600	[0.12 , 1.09]	Non-inferiority not shown	0.007
[TKR] PFC Sigma Bicondylar Knee Cement, unconstrained fixed	47,826	1.240	0.240	[-0.23 , 0.71]	Non-inferiority not shown	0.158
[TKR] PFC Sigma Bicondylar Knee Cement, unconstrained mobile	2,332	1.510	0.520	[-0.14 , 1.17]	Non-inferiority not shown	0.062
[TKR] PFC Sigma Bicondylar Knee Hybrid, unconstrained fixed	557	1.570	0.570	[-0.54 , 1.69]	Non-inferiority not shown	0.158
[TKR] PFC Sigma Bicondylar Knee monobloc polyethylene tibia	1,703	1.490	0.490	[-0.18 , 1.15]	Non-inferiority not shown	0.077

For peer review only

[TKR] Profix Cement, unconstrained fixed	335	1.170	0.180	[-1.06 , 1.41]	Non-inferiority not shown	0.390
[TKR] Profix Oxinium monobloc polyethylene tibia	270	2.180	1.180	[-0.60 , 2.96]	Non-inferiority not shown	0.098
[TKR] Profix Uncemented, unconstrained fixed	680	1.710	0.720	[-0.35 , 1.78]	Non-inferiority not shown	0.094
[TKR] Rotaglide + Cement, unconstrained mobile	597	2.120	1.120	[-0.11 , 2.35]	Non-inferiority not shown	0.037
[TKR] Rotaglide Cement, unconstrained mobile	397	1.760	0.760	[-0.53 , 2.06]	Non-inferiority not shown	0.124
[TKR] Scorpio Cement, posterior-stabilised mobile	483	1.210	0.210	[-0.85 , 1.28]	Non-inferiority not shown	0.349
[TKR] Scorpio Cement, posterior-stabilised fixed	2,156	1.450	0.450	[-0.23 , 1.13]	Non-inferiority not shown	0.097
[TKR] Scorpio Cement, unconstrained fixed	3,812	1.580	0.580	[-0.02 , 1.18]	Non-inferiority not shown	0.029
[TKR] Scorpio Cement, unconstrained mobile	462	2.310	1.310	[-0.11 , 2.74]	Non-inferiority not shown	0.035
[TKR] Scorpio Hybrid, unconstrained fixed	373	1.810	0.810	[-0.59 , 2.22]	Non-inferiority not shown	0.129
[TKR] Scorpio Uncemented, unconstrained fixed	1,215	1.900	0.900	[0.02 , 1.79]	Non-inferiority not shown	0.022
[TKR] TC Plus Cement, unconstrained fixed	2,740	1.660	0.670	[0.01 , 1.32]	Non-inferiority not shown	0.024
[TKR] TC Plus Uncemented, unconstrained mobile	565	1.810	0.810	[-0.31 , 1.92]	Non-inferiority not shown	0.078
[TKR] Triathlon Cement, posterior-stabilised fixed	3,116	1.680	0.680	[0.08 , 1.29]	Non-inferiority not shown	0.013
[TKR] Triathlon Cement, unconstrained fixed	11,030	1.580	0.580	[0.08 , 1.08]	Non-inferiority not shown	0.011
[TKR] Triathlon Uncemented, unconstrained fixed	262	2.860	1.860	[-0.05 , 3.77]	Non-inferiority not shown	0.028
[TKR] Vanguard Cement, posterior-stabilised fixed	1,116	2.040	1.040	[0.22 , 1.85]	Inferior by ≥20%	0.006
[TKR] Vanguard Cement, unconstrained fixed	7,465	1.380	0.380	[-0.13 , 0.89]	Non-inferiority not shown	0.072
[UNI] AMC/Uniglide Unicondylar, fixed	390	3.200	2.200	[0.49 , 3.92]	Inferior by ≥20%	0.006
[UNI] AMC/Uniglide Unicondylar, mobile	407	7.070	6.070	[3.66 , 8.47]	Inferior by ≥100%	<0.001
[UNI] MG Uni Unicondylar, fixed	673	3.990	2.990	[1.47 , 4.51]	Inferior by ≥100%	<0.001
[UNI] Oxford Partial Knee Unicondylar, mobile	12,307	3.830	2.830	[2.28 , 3.38]	Inferior by ≥100%	<0.001
[UNI] Physica ZUK Unicondylar, fixed	1,431	2.090	1.090	[0.30 , 1.89]	Inferior by ≥20%	0.003
[UNI] Preservation Unicondylar, fixed	333	8.230	7.230	[4.37 , 10.09]	Inferior by ≥100%	<0.001
[UNI] Sigma HP Unicondylar, fixed	959	3.090	2.090	[1.09 , 3.09]	Inferior by ≥100%	<0.001
[UNI] Sled Unicondylar, fixed	264	6.670	5.680	[2.81 , 8.54]	Inferior by ≥100%	<0.001

For peer review only

Supplementary table 22: Difference in cumulative percentage revision of knee implants compared to a contemporary benchmark at 5 years post primary in women between 55 and 75 years for knee replacements with ≥250 procedures remaining at risk

Knee brand, bearing and constraint	Number at risk	Cumulative failure (%)	Difference in failure (%)	95% CI	Equivalence status	p-value
[TKR] MRK Cement, unconstrained fixed	1,647	1.47	[REFERENCE]			
[TKR] AGC Cement, unconstrained fixed	15,179	2.11	0.64	[0.14 , 1.14]	Non-inferiority not shown	0.006
[TKR] AGC Hybrid, unconstrained fixed	282	2.93	1.46	[-0.48 , 3.40]	Non-inferiority not shown	0.071
[TKR] AGC Uncemented, unconstrained fixed	358	6.74	5.27	[2.77 , 7.77]	Inferior by ≥100%	<0.001
[TKR] Advance MP Cement, unconstrained fixed	1,520	2.45	0.98	[0.19 , 1.78]	Non-inferiority not shown	0.008
[TKR] Advance MP Stature Cement, unconstrained fixed	267	2.49	1.02	[-0.38 , 2.42]	Non-inferiority not shown	0.077
[TKR] Columbus Cement, unconstrained fixed	1,052	2.83	1.36	[0.50 , 2.23]	Inferior by ≥20%	0.001
[TKR] E-Motion Bicondylar Knee Uncemented, unconstrained mobile	387	2.88	1.41	[-0.07 , 2.88]	Non-inferiority not shown	0.031
[TKR] Genesis 2 Cement, posterior-stabilised fixed	1,842	2.39	0.92	[0.24 , 1.60]	Non-inferiority not shown	0.004
[TKR] Genesis 2 Cement, unconstrained fixed	5,902	1.59	0.12	[-0.39 , 0.64]	Non-inferiority not shown	0.320
[TKR] Genesis 2 Cement, unconstrained mobile	301	1.78	0.31	[-1.18 , 1.79]	Non-inferiority not shown	0.342
[TKR] Genesis 2 Oxinium Cement, posterior-stabilised fixed	391	3.33	1.86	[0.44 , 3.28]	Inferior by ≥20%	0.005
[TKR] Genesis 2 Oxinium Cement, unconstrained fixed	1,039	2.11	0.63	[-0.23 , 1.50]	Non-inferiority not shown	0.075
[TKR] Insall-Burstein 2 Cement, posterior-stabilised fixed	775	3.28	1.81	[0.51 , 3.11]	Inferior by ≥20%	0.003
[TKR] Kinemax Cement, unconstrained fixed	3,479	2.96	1.49	[0.78 , 2.20]	Inferior by ≥20%	<0.001
[TKR] LCS Complete Cement, unconstrained mobile	2,534	2.91	1.44	[0.70 , 2.17]	Inferior by ≥20%	<0.001
[TKR] LCS Complete Uncemented, unconstrained mobile	2,848	2.79	1.32	[0.63 , 2.01]	Inferior by ≥20%	<0.001
[TKR] LCS Uncemented, unconstrained mobile	438	2.98	1.51	[-0.10 , 3.11]	Non-inferiority not shown	0.033
[TKR] Maxim Cement, unconstrained fixed	432	2.48	1.01	[-0.45 , 2.47]	Non-inferiority not shown	0.088
[TKR] NRG Cement, posterior-stabilised fixed	1,187	2.73	1.26	[0.34 , 2.18]	Inferior by ≥20%	0.004
[TKR] NRG Cement, unconstrained fixed	1,577	2.12	0.65	[-0.10 , 1.40]	Non-inferiority not shown	0.044
[TKR] Natural Knee II Cement, unconstrained fixed	773	3.08	1.61	[0.43 , 2.80]	Inferior by ≥20%	0.004
[TKR] NexGen Cement, posterior-stabilised mobile	275	3.40	1.93	[-0.03 , 3.90]	Non-inferiority not shown	0.027
[TKR] NexGen Cement, posterior-stabilised fixed	11,170	2.61	1.14	[0.62 , 1.66]	Inferior by ≥20%	<0.001
[TKR] NexGen Cement, unconstrained fixed	8,293	1.58	0.11	[-0.40 , 0.61]	Non-inferiority not shown	0.338
[TKR] NexGen Hybrid, unconstrained fixed	303	1.46	-0.01	[-1.36 , 1.34]	Non-inferiority not shown	0.493
[TKR] NexGen Uncemented, posterior-stabilised fixed	339	1.68	0.21	[-1.04 , 1.47]	Non-inferiority not shown	0.370
[TKR] NexGen Uncemented, unconstrained fixed	1,007	3.46	1.98	[0.84 , 3.13]	Inferior by ≥20%	<0.001
[TKR] Optetrak Cement, posterior-stabilised fixed	508	5.04	3.57	[1.75 , 5.38]	Inferior by ≥100%	<0.001
[TKR] PFC Sigma Bicondylar Knee Cement, posterior-stabilised mobile	1,587	2.74	1.27	[0.45 , 2.08]	Inferior by ≥20%	0.001
[TKR] PFC Sigma Bicondylar Knee Cement, posterior-stabilised fixed	15,223	2.26	0.78	[0.29 , 1.28]	Non-inferiority not shown	0.001
[TKR] PFC Sigma Bicondylar Knee Cement, unconstrained fixed	34,361	1.75	0.28	[-0.19 , 0.75]	Non-inferiority not shown	0.121
[TKR] PFC Sigma Bicondylar Knee Cement, unconstrained mobile	1,974	2.28	0.81	[0.06 , 1.56]	Non-inferiority not shown	0.017
[TKR] PFC Sigma Bicondylar Knee Hybrid, unconstrained fixed	523	1.76	0.28	[-0.89 , 1.46]	Non-inferiority not shown	0.318
[TKR] PFC Sigma Bicondylar Knee monobloc polyethylene tibia	721	2.00	0.53	[-0.25 , 1.32]	Non-inferiority not shown	0.092
[TKR] Profix Cement, unconstrained fixed	315	2.08	0.61	[-0.98 , 2.20]	Non-inferiority not shown	0.228
[TKR] Profix Oxinium monobloc polyethylene tibia	262	2.91	1.44	[-0.60 , 3.48]	Non-inferiority not shown	0.083
[TKR] Profix Uncemented, unconstrained fixed	619	2.16	0.69	[-0.49 , 1.86]	Non-inferiority not shown	0.125
[TKR] Rotaglide + Cement, unconstrained mobile	571	2.78	1.31	[-0.07 , 2.69]	Non-inferiority not shown	0.032
[TKR] Rotaglide Cement, unconstrained mobile	299	3.20	1.72	[-0.07 , 3.52]	Non-inferiority not shown	0.030

For peer review only

[TKR] Scorpio Cement, posterior-stabilised mobile	455	2.04	0.57	[-0.76 , 1.90]	Non-inferiority not shown	0.201
[TKR] Scorpio Cement, posterior-stabilised fixed	2,061	2.23	0.76	[-0.01 , 1.53]	Non-inferiority not shown	0.026
[TKR] Scorpio Cement, unconstrained fixed	3,488	2.43	0.96	[0.29 , 1.62]	Non-inferiority not shown	0.002
[TKR] Scorpio Cement, unconstrained mobile	445	3.17	1.70	[0.05 , 3.34]	Non-inferiority not shown	0.022
[TKR] Scorpio Hybrid, unconstrained fixed	356	2.35	0.88	[-0.71 , 2.47]	Non-inferiority not shown	0.138
[TKR] Scorpio Uncemented, unconstrained fixed	1,106	2.49	1.02	[0.04 , 2.00]	Non-inferiority not shown	0.021
[TKR] TC Plus Cement, unconstrained fixed	2,588	2.39	0.92	[0.19 , 1.65]	Non-inferiority not shown	0.007
[TKR] TC Plus Cement, unconstrained mobile	1,584	1.48	0.01	[-0.72 , 0.74]	Non-inferiority not shown	0.488
[TKR] TC Plus Uncemented, unconstrained mobile	456	2.74	1.27	[-0.11 , 2.64]	Non-inferiority not shown	0.035
[TKR] Triathlon Cement, posterior-stabilised fixed	1,765	2.61	1.14	[0.43 , 1.85]	Inferior by ≥20%	0.001
[TKR] Triathlon Cement, unconstrained fixed	5,497	2.07	0.60	[0.08 , 1.12]	Non-inferiority not shown	0.011
[TKR] Vanguard Cement, posterior-stabilised fixed	426	2.94	1.46	[0.44 , 2.49]	Inferior by ≥20%	0.003
[TKR] Vanguard Cement, unconstrained fixed	3,246	2.20	0.72	[0.16 , 1.28]	Non-inferiority not shown	0.006
[UNI] AMC/Uniglide Unicondylar, fixed	275	4.59	3.12	[1.04 , 5.20]	Inferior by ≥20%	0.002
[UNI] AMC/Uniglide Unicondylar, mobile	346	9.71	8.23	[5.43 , 11.04]	Inferior by ≥100%	<0.001
[UNI] MG Uni Unicondylar, fixed	651	5.28	3.81	[2.09 , 5.53]	Inferior by ≥100%	<0.001
[UNI] Oxford Partial Knee Unicondylar, mobile	9,400	6.04	4.57	[3.96 , 5.18]	Inferior by ≥100%	<0.001
[UNI] Physica ZUK Unicondylar, fixed	754	3.52	2.05	[1.00 , 3.11]	Inferior by ≥20%	<0.001
[UNI] Preservation Unicondylar, fixed	318	11.84	10.37	[7.01 , 13.72]	Inferior by ≥100%	<0.001
[UNI] Sigma HP Unicondylar, fixed	446	5.02	3.54	[2.09 , 5.00]	Inferior by ≥100%	<0.001

For peer review only

Supplementary table 23: Difference in cumulative percentage revision of knee implants compared to a contemporary benchmark at 7 years post primary in women between 55 and 75 years for knee replacements with ≥250 procedures remaining at risk

Knee brand, bearing and constraint	Number at risk	Cumulative failure (%)	Difference in failure (%)	95% CI	Equivalence status	p-value
[TKR] TC Plus Cement, unconstrained mobile	1,341	1.95	[REFERENCE]			
[TKR] AGC Cement, unconstrained fixed	10,578	2.61	0.66	[-0.04 , 1.36]	Non-inferiority not shown	0.032
[TKR] AGC Uncemented, unconstrained fixed	278	8.16	6.21	[3.41 , 9.00]	Inferior by ≥100%	<0.001
[TKR] Advance MP Cement, unconstrained fixed	1,054	3.35	1.40	[0.35 , 2.45]	Non-inferiority not shown	0.005
[TKR] Columbus Cement, unconstrained fixed	585	2.95	1.00	[-0.01 , 2.01]	Non-inferiority not shown	0.026
[TKR] E-Motion Bicondylar Knee Uncemented, unconstrained mobile	279	4.06	2.11	[0.19 , 4.03]	Non-inferiority not shown	0.015
[TKR] Genesis 2 Cement, posterior-stabilised fixed	938	2.62	0.67	[-0.19 , 1.53]	Non-inferiority not shown	0.063
[TKR] Genesis 2 Cement, unconstrained fixed	3,140	2.06	0.11	[-0.62 , 0.84]	Non-inferiority not shown	0.383
[TKR] Genesis 2 Oxinium Cement, unconstrained fixed	632	2.69	0.74	[-0.37 , 1.85]	Non-inferiority not shown	0.096
[TKR] Insall-Burstein 2 Cement, posterior-stabilised fixed	738	4.30	2.35	[0.81 , 3.89]	Inferior by ≥20%	0.001
[TKR] Kinemax Cement, unconstrained fixed	3,294	3.96	2.01	[1.10 , 2.93]	Inferior by ≥20%	<0.001
[TKR] LCS Complete Cement, unconstrained mobile	1,676	3.88	1.94	[0.97 , 2.90]	Inferior by ≥20%	<0.001
[TKR] LCS Complete Uncemented, unconstrained mobile	1,824	3.09	1.14	[0.28 , 2.01]	Non-inferiority not shown	0.005
[TKR] LCS Uncemented, unconstrained mobile	425	2.98	1.03	[-0.64 , 2.70]	Non-inferiority not shown	0.114
[TKR] MRK Cement, unconstrained fixed	1,125	2.02	0.07	[-0.82 , 0.96]	Non-inferiority not shown	0.438
[TKR] Maxim Cement, unconstrained fixed	344	3.50	1.55	[-0.27 , 3.36]	Non-inferiority not shown	0.047
[TKR] NRG Cement, posterior-stabilised fixed	572	3.26	1.31	[0.17 , 2.45]	Non-inferiority not shown	0.012
[TKR] NRG Cement, unconstrained fixed	920	2.41	0.46	[-0.47 , 1.39]	Non-inferiority not shown	0.165
[TKR] Natural Knee II Cement, unconstrained fixed	584	3.82	1.87	[0.45 , 3.29]	Inferior by ≥20%	0.005
[TKR] NexGen Cement, posterior-stabilised fixed	7,194	3.32	1.37	[0.65 , 2.10]	Inferior by ≥20%	<0.001
[TKR] NexGen Cement, unconstrained fixed	5,031	2.15	0.21	[-0.51 , 0.92]	Non-inferiority not shown	0.286
[TKR] NexGen Uncemented, posterior-stabilised fixed	251	2.36	0.41	[-1.22 , 2.04]	Non-inferiority not shown	0.310
[TKR] NexGen Uncemented, unconstrained fixed	909	4.05	2.10	[0.78 , 3.43]	Inferior by ≥20%	0.001
[TKR] Optetrak Cement, posterior-stabilised fixed	319	5.23	3.28	[1.37 , 5.20]	Inferior by ≥20%	<0.001
[TKR] PFC Sigma Bicondylar Knee Cement, posterior-stabilised mobile	1,016	3.32	1.37	[0.35 , 2.40]	Non-inferiority not shown	0.004
[TKR] PFC Sigma Bicondylar Knee Cement, posterior-stabilised fixed	9,914	2.66	0.72	[0.02 , 1.41]	Non-inferiority not shown	0.022
[TKR] PFC Sigma Bicondylar Knee Cement, unconstrained fixed	22,205	2.06	0.11	[-0.56 , 0.78]	Non-inferiority not shown	0.375
[TKR] PFC Sigma Bicondylar Knee Cement, unconstrained mobile	1,414	2.45	0.50	[-0.40 , 1.41]	Non-inferiority not shown	0.138
[TKR] PFC Sigma Bicondylar Knee Hybrid, unconstrained fixed	453	1.76	-0.19	[-1.46 , 1.07]	Non-inferiority not shown	0.383
[TKR] PFC Sigma Bicondylar Knee monobloc polyethylene tibia	265	2.00	0.06	[-0.86 , 0.98]	Non-inferiority not shown	0.451
[TKR] Profix Cement, unconstrained fixed	289	2.73	0.78	[-1.10 , 2.66]	Non-inferiority not shown	0.208
[TKR] Profix Uncemented, unconstrained fixed	508	2.16	0.21	[-1.05 , 1.48]	Non-inferiority not shown	0.370
[TKR] Rotaglide + Cement, unconstrained mobile	497	3.13	1.19	[-0.35 , 2.72]	Non-inferiority not shown	0.065
[TKR] Scorpio Cement, posterior-stabilised mobile	393	2.50	0.55	[-1.00 , 2.09]	Non-inferiority not shown	0.243
[TKR] Scorpio Cement, posterior-stabilised fixed	1,896	2.92	0.97	[0.00 , 1.94]	Non-inferiority not shown	0.025
[TKR] Scorpio Cement, unconstrained fixed	2,905	2.86	0.92	[0.07 , 1.77]	Non-inferiority not shown	0.017
[TKR] Scorpio Cement, unconstrained mobile	415	4.49	2.54	[0.55 , 4.53]	Inferior by ≥20%	0.006
[TKR] Scorpio Hybrid, unconstrained fixed	324	2.94	0.99	[-0.85 , 2.82]	Non-inferiority not shown	0.145
[TKR] Scorpio Uncemented, unconstrained fixed	820	3.17	1.22	[0.03 , 2.41]	Non-inferiority not shown	0.023
[TKR] TC Plus Cement, unconstrained fixed	2,030	2.99	1.04	[0.12 , 1.96]	Non-inferiority not shown	0.013

For peer review only

[TKR] TC Plus Uncemented, unconstrained mobile	330	2.98	1.03	[-0.49 , 2.56]	Non-inferiority not shown	0.092
[TKR] Triathlon Cement, posterior-stabilised fixed	633	2.91	0.96	[0.04 , 1.88]	Non-inferiority not shown	0.020
[TKR] Triathlon Cement, unconstrained fixed	1,918	2.48	0.53	[-0.20 , 1.26]	Non-inferiority not shown	0.079
[TKR] Vanguard Cement, unconstrained fixed	1,009	2.74	0.80	[-0.02 , 1.61]	Non-inferiority not shown	0.027
[UNI] AMC/Uniglide Unicondylar, mobile	254	11.38	9.44	[6.34 , 12.53]	Inferior by ≥100%	<0.001
[UNI] MG Uni Unicondylar, fixed	584	6.64	4.69	[2.72 , 6.66]	Inferior by ≥100%	<0.001
[UNI] Oxford Partial Knee Unicondylar, mobile	6,459	8.09	6.14	[5.31 , 6.97]	Inferior by ≥100%	<0.001
[UNI] Physica ZUK Unicondylar, fixed	327	6.01	4.06	[2.32 , 5.81]	Inferior by ≥100%	<0.001
[UNI] Preservation Unicondylar, fixed	292	14.69	12.74	[9.02 , 16.45]	Inferior by ≥100%	<0.001

For peer review only

Supplementary table 24: Difference in cumulative percentage revision of knee implants compared to a contemporary benchmark at 10 years post primary in women between 55 and 75 years for knee replacements with ≥250 procedures remaining at risk

Knee brand, bearing and constraint	Number at risk	Cumulative failure (%)	Difference in failure (%)	95% CI	Equivalence status	p-value
[TKR] PFC Sigma Bicondylar Knee Cement, unconstrained fixed	8,221	2.4	[REFERENCE]			
[TKR] AGC Cement, unconstrained fixed	3,879	3.4	1.0	[0.65 , 1.35]	Inferior by ≥20%	<0.001
[TKR] Advance MP Cement, unconstrained fixed	299	4.4	2.0	[0.77 , 3.13]	Inferior by ≥20%	0.001
[TKR] Genesis 2 Cement, unconstrained fixed	853	2.4	0.0	[-0.45 , 0.41]	Non-inferior	0.462
[TKR] Insall-Burstein 2 Cement, posterior-stabilised fixed	489	5.3	2.9	[1.34 , 4.49]	Inferior by ≥20%	<0.001
[TKR] Kinemax Cement, unconstrained fixed	2,094	5.4	3.0	[2.20 , 3.76]	Inferior by ≥20%	<0.001
[TKR] LCS Complete Cement, unconstrained mobile	352	4.9	2.5	[1.50 , 3.53]	Inferior by ≥20%	<0.001
[TKR] LCS Complete Uncemented, unconstrained mobile	531	3.5	1.1	[0.43 , 1.76]	Non-inferiority not shown	0.001
[TKR] LCS Uncemented, unconstrained mobile	393	3.0	0.6	[-0.96 , 2.13]	Non-inferiority not shown	0.231
[TKR] NexGen Cement, posterior-stabilised fixed	2,484	4.4	2.0	[1.51 , 2.40]	Inferior by ≥20%	<0.001
[TKR] NexGen Cement, unconstrained fixed	1,217	2.7	0.3	[-0.14 , 0.72]	Non-inferiority not shown	0.095
[TKR] NexGen Uncemented, unconstrained fixed	322	4.5	2.1	[0.83 , 3.34]	Inferior by ≥20%	0.001
[TKR] PFC Sigma Bicondylar Knee Cement, posterior-stabilised mobile	313	3.3	0.9	[0.13 , 1.72]	Non-inferiority not shown	0.012
[TKR] PFC Sigma Bicondylar Knee Cement, posterior-stabilised fixed	3,360	3.3	0.9	[0.52 , 1.19]	Inferior by ≥20%	<0.001
[TKR] PFC Sigma Bicondylar Knee Cement, unconstrained mobile	338	3.1	0.7	[-0.15 , 1.45]	Non-inferiority not shown	0.056
[TKR] PFC Sigma Bicondylar Knee Hybrid, unconstrained fixed	270	2.0	-0.4	[-1.58 , 0.81]	Non-inferiority not shown	0.263
[TKR] Rotaglide + Cement, unconstrained mobile	289	4.8	2.4	[0.56 , 4.24]	Inferior by ≥20%	0.005
[TKR] Scorpio Cement, posterior-stabilised fixed	842	3.7	1.3	[0.42 , 2.13]	Non-inferiority not shown	0.002
[TKR] Scorpio Cement, unconstrained fixed	1,414	3.6	1.2	[0.52 , 1.83]	Inferior by ≥20%	<0.001
[TKR] TC Plus Cement, unconstrained fixed	504	3.6	1.2	[0.35 , 2.00]	Non-inferiority not shown	0.003
[TKR] TC Plus Cement, unconstrained mobile	635	2.5	0.1	[-0.71 , 0.92]	Non-inferiority not shown	0.399
[UNI] MG Uni Unicondylar, fixed	287	8.8	6.4	[4.12 , 8.61]	Inferior by ≥100%	<0.001
[UNI] Oxford Partial Knee Unicondylar, mobile	2,271	11.9	9.5	[8.75 , 10.29]	Inferior by ≥100%	<0.001

For peer review only

Supplementary table 25: Difference in cumulative percentage revision of knee implants compared to a contemporary benchmark at 3 years post primary in men over 75 years for knee replacements with ≥250 procedures remaining at risk

Knee brand, bearing and constraint	Number at risk	Cumulative failure (%)	Difference in failure (%)	95% CI	Equivalence status	p-value
[TKR] NexGen Cement, unconstrained fixed	3,575	0.63	[REFERENCE]			
[TKR] AGC Cement, unconstrained fixed	5,857	1.06	0.43	[0.11 , 0.76]	Non-inferiority not shown	0.004
[TKR] Advance MP Cement, unconstrained fixed	586	1.71	1.08	[0.13 , 2.02]	Inferior by ≥20%	0.013
[TKR] Columbus Cement, unconstrained fixed	541	1.58	0.96	[0.07 , 1.85]	Non-inferiority not shown	0.018
[TKR] Genesis 2 Cement, posterior-stabilised fixed	845	0.61	-0.02	[-0.49 , 0.46]	Non-inferiority not shown	0.468
[TKR] Genesis 2 Cement, unconstrained fixed	3,120	0.92	0.29	[-0.06 , 0.64]	Non-inferiority not shown	0.050
[TKR] Insall-Burstein 2 Cement, posterior-stabilised fixed	270	0.35	-0.28	[-0.99 , 0.44]	Non-inferiority not shown	0.223
[TKR] Kinemax Cement, unconstrained fixed	1,108	1.07	0.44	[-0.18 , 1.06]	Non-inferiority not shown	0.081
[TKR] LCS Complete Cement, unconstrained mobile	825	0.71	0.08	[-0.49 , 0.65]	Non-inferiority not shown	0.391
[TKR] LCS Complete Uncemented, unconstrained mobile	999	0.81	0.19	[-0.34 , 0.71]	Non-inferiority not shown	0.244
[TKR] MRK Cement, unconstrained fixed	644	1.02	0.39	[-0.31 , 1.10]	Non-inferiority not shown	0.138
[TKR] NRG Cement, posterior-stabilised fixed	447	1.60	0.97	[-0.15 , 2.09]	Non-inferiority not shown	0.045
[TKR] NRG Cement, unconstrained fixed	646	0.84	0.21	[-0.44 , 0.86]	Non-inferiority not shown	0.265
[TKR] Natural Knee II Cement, unconstrained fixed	250	0.00	--	[-.-- , -.--]	No failures to date	
[TKR] NexGen Cement, posterior-stabilised fixed	4,136	1.00	0.37	[0.03 , 0.71]	Non-inferiority not shown	0.016
[TKR] NexGen Uncemented, unconstrained fixed	267	0.68	0.05	[-0.91 , 1.02]	Non-inferiority not shown	0.456
[TKR] PFC Sigma Bicondylar Knee Cement, posterior-stabilised mobile	318	2.09	1.46	[0.01 , 2.91]	Non-inferiority not shown	0.024
[TKR] PFC Sigma Bicondylar Knee Cement, posterior-stabilised fixed	6,056	1.11	0.49	[0.17 , 0.81]	Inferior by ≥20%	0.001
[TKR] PFC Sigma Bicondylar Knee Cement, unconstrained fixed	13,197	0.84	0.22	[-0.04 , 0.47]	Non-inferiority not shown	0.048
[TKR] PFC Sigma Bicondylar Knee Cement, unconstrained mobile	375	1.53	0.90	[-0.25 , 2.05]	Non-inferiority not shown	0.062
[TKR] PFC Sigma Bicondylar Knee monobloc polyethylene tibia	1,004	1.26	0.64	[0.01 , 1.26]	Non-inferiority not shown	0.023
[TKR] Profix Uncemented, unconstrained fixed	296	0.63	0.00	[-0.89 , 0.89]	Non-inferiority not shown	0.498
[TKR] Scorpio Cement, posterior-stabilised fixed	624	0.89	0.26	[-0.48 , 1.00]	Non-inferiority not shown	0.246
[TKR] Scorpio Cement, unconstrained fixed	1,038	1.15	0.53	[-0.13 , 1.19]	Non-inferiority not shown	0.059
[TKR] Scorpio Uncemented, unconstrained fixed	373	0.52	-0.11	[-0.86 , 0.64]	Non-inferiority not shown	0.386
[TKR] TC Plus Cement, unconstrained fixed	831	1.54	0.91	[0.08 , 1.74]	Non-inferiority not shown	0.016
[TKR] TC Plus Cement, unconstrained mobile	418	1.02	0.39	[-0.52 , 1.31]	Non-inferiority not shown	0.201
[TKR] Triathlon Cement, posterior-stabilised fixed	764	0.78	0.15	[-0.38 , 0.68]	Non-inferiority not shown	0.289
[TKR] Triathlon Cement, unconstrained fixed	2,911	1.01	0.38	[0.02 , 0.74]	Non-inferiority not shown	0.018
[TKR] Vanguard Cement, posterior-stabilised fixed	291	0.36	-0.26	[-0.82 , 0.30]	Non-inferiority not shown	0.178
[TKR] Vanguard Cement, unconstrained fixed	1,887	0.97	0.34	[-0.07 , 0.75]	Non-inferiority not shown	0.053
[UNI] Oxford Partial Knee Unicondylar, mobile	2,355	2.45	1.82	[1.23 , 2.42]	Inferior by ≥100%	<0.001

For peer review only

Supplementary table 26: Difference in cumulative percentage revision of knee implants compared to a contemporary benchmark at 5 years post primary in men over 75 years for knee replacements with ≥250 procedures remaining at risk

Knee brand, bearing and constraint	Number at risk	Cumulative failure (%)	Difference in failure (%)	95% CI	Equivalence status	p-value
[TKR] Nexgen Cement, unconstrained fixed	2,068	0.90	[REFERENCE]			
[TKR] AGC Cement, unconstrained fixed	4,270	1.31	0.41	[0.01 , 0.81]	Non-inferiority not shown	0.022
[TKR] Advance MP Cement, unconstrained fixed	362	2.15	1.25	[0.11 , 2.40]	Non-inferiority not shown	0.016
[TKR] Columbus Cement, unconstrained fixed	273	1.58	0.69	[-0.22 , 1.60]	Non-inferiority not shown	0.070
[TKR] Genesis 2 Cement, posterior-stabilised fixed	445	1.04	0.14	[-0.56 , 0.84]	Non-inferiority not shown	0.350
[TKR] Genesis 2 Cement, unconstrained fixed	1,766	1.51	0.62	[0.11 , 1.12]	Non-inferiority not shown	0.008
[TKR] Kinemax Cement, unconstrained fixed	949	1.35	0.46	[-0.26 , 1.18]	Non-inferiority not shown	0.107
[TKR] LCS Complete Cement, unconstrained mobile	585	1.14	0.25	[-0.53 , 1.02]	Non-inferiority not shown	0.266
[TKR] LCS Complete Uncemented, unconstrained mobile	681	1.02	0.12	[-0.50 , 0.75]	Non-inferiority not shown	0.348
[TKR] MRK Cement, unconstrained fixed	413	1.18	0.28	[-0.51 , 1.07]	Non-inferiority not shown	0.245
[TKR] NRG Cement, posterior-stabilised fixed	303	2.34	1.44	[0.04 , 2.85]	Non-inferiority not shown	0.022
[TKR] NRG Cement, unconstrained fixed	378	0.84	-0.06	[-0.74 , 0.62]	Non-inferiority not shown	0.432
[TKR] Nexgen Cement, posterior-stabilised fixed	2,698	1.37	0.47	[0.03 , 0.91]	Non-inferiority not shown	0.017
[TKR] PFC Sigma Bicondylar Knee Cement, posterior-stabilised fixed	4,060	1.31	0.41	[0.02 , 0.80]	Non-inferiority not shown	0.019
[TKR] PFC Sigma Bicondylar Knee Cement, unconstrained fixed	8,777	1.02	0.12	[-0.20 , 0.44]	Non-inferiority not shown	0.234
[TKR] PFC Sigma Bicondylar Knee Cement, unconstrained mobile	292	2.41	1.51	[-0.01 , 3.03]	Non-inferiority not shown	0.026
[TKR] PFC Sigma Bicondylar Knee monobloc polyethylene tibia	431	1.40	0.51	[-0.20 , 1.21]	Non-inferiority not shown	0.080
[TKR] Scorpio Cement, posterior-stabilised fixed	543	1.39	0.49	[-0.46 , 1.44]	Non-inferiority not shown	0.154
[TKR] Scorpio Cement, unconstrained fixed	861	1.56	0.67	[-0.13 , 1.46]	Non-inferiority not shown	0.050
[TKR] Scorpio Uncemented, unconstrained fixed	304	0.79	-0.11	[-1.04 , 0.82]	Non-inferiority not shown	0.409
[TKR] TC Plus Cement, unconstrained fixed	719	1.79	0.89	[-0.02 , 1.81]	Non-inferiority not shown	0.028
[TKR] TC Plus Cement, unconstrained mobile	353	1.26	0.36	[-0.68 , 1.40]	Non-inferiority not shown	0.249
[TKR] Triathlon Cement, posterior-stabilised fixed	423	1.34	0.44	[-0.41 , 1.29]	Non-inferiority not shown	0.153
[TKR] Triathlon Cement, unconstrained fixed	1,328	1.39	0.49	[0.01 , 0.97]	Non-inferiority not shown	0.024
[TKR] Vanguard Cement, unconstrained fixed	749	1.34	0.44	[-0.10 , 0.99]	Non-inferiority not shown	0.054
[UNI] Oxford Partial Knee Unicondylar, mobile	1,681	3.30	2.40	[1.67 , 3.14]	Inferior by ≥100%	<0.001

For peer review only

Supplementary table 27: Difference in cumulative percentage revision of knee implants compared to a contemporary benchmark at 7 years post primary in men over 75 years for knee replacements with ≥250 procedures remaining at risk

Knee brand, bearing and constraint	Number at risk	Cumulative failure (%)	Difference in failure (%)	95% CI	Equivalence status	p-value
[TKR] NexGen Cement, unconstrained fixed	1,099	1.11	[REFERENCE]			
[TKR] AGC Cement, unconstrained fixed	2,540	1.54	0.43	[-0.05 , 0.91]	Non-inferiority not shown	0.040
[TKR] Genesis 2 Cement, unconstrained fixed	846	1.78	0.67	[0.06 , 1.28]	Non-inferiority not shown	0.015
[TKR] Kinemax Cement, unconstrained fixed	790	1.82	0.71	[-0.17 , 1.58]	Non-inferiority not shown	0.057
[TKR] LCS Complete Cement, unconstrained mobile	356	1.32	0.21	[-0.66 , 1.09]	Non-inferiority not shown	0.317
[TKR] LCS Complete Uncemented, unconstrained mobile	376	1.53	0.42	[-0.46 , 1.31]	Non-inferiority not shown	0.174
[TKR] NexGen Cement, posterior-stabilised fixed	1,561	1.60	0.49	[-0.03 , 1.02]	Non-inferiority not shown	0.033
[TKR] PFC Sigma Bicondylar Knee Cement, posterior-stabilised fixed	2,298	1.59	0.48	[-0.00 , 0.96]	Non-inferiority not shown	0.026
[TKR] PFC Sigma Bicondylar Knee Cement, unconstrained fixed	4,935	1.15	0.04	[-0.35 , 0.44]	Non-inferiority not shown	0.417
[TKR] Scorpio Cement, posterior-stabilised fixed	418	1.83	0.72	[-0.42 , 1.86]	Non-inferiority not shown	0.109
[TKR] Scorpio Cement, unconstrained fixed	634	1.56	0.45	[-0.37 , 1.27]	Non-inferiority not shown	0.140
[TKR] TC Plus Cement, unconstrained fixed	499	1.79	0.68	[-0.26 , 1.62]	Non-inferiority not shown	0.079
[TKR] TC Plus Cement, unconstrained mobile	261	1.58	0.47	[-0.76 , 1.70]	Non-inferiority not shown	0.228
[TKR] Triathlon Cement, unconstrained fixed	381	1.85	0.74	[-0.01 , 1.48]	Non-inferiority not shown	0.027
[UNI] Oxford Partial Knee Unicondylar, mobile	975	4.75	3.64	[2.64 , 4.64]	Inferior by ≥100%	<0.001

For peer review only

Supplementary table 28: Difference in cumulative percentage revision of knee implants compared to a contemporary benchmark at 10 years post primary in men over 75 years for knee replacements with ≥250 procedures remaining at risk

Knee brand, bearing and constraint	Number at risk	Cumulative failure (%)	Difference in failure (%)	95% CI	Equivalence status	p-value
[TKR] PFC Sigma Bicondylar Knee Cement, unconstrained fixed	1,319	1.25	[REFERENCE]			
[TKR] AGC Cement, unconstrained fixed	660	1.79	0.54	[0.08 , 1.00]	Non-inferiority not shown	0.011
[TKR] Kinemax Cement, unconstrained fixed	359	2.30	1.05	[0.05 , 2.06]	Non-inferiority not shown	0.019
[TKR] NexGen Cement, posterior-stabilised fixed	385	2.00	0.75	[0.12 , 1.39]	Non-inferiority not shown	0.010
[TKR] PFC Sigma Bicondylar Knee Cement, posterior-stabilised fixed	590	2.04	0.80	[0.23 , 1.36]	Non-inferiority not shown	0.003
[UNI] Oxford Partial Knee Unicondylar, mobile	290	5.90	4.65	[3.41 , 5.90]	Inferior by ≥100%	<0.001

For peer review only

Supplementary table 29: Difference in cumulative percentage revision of knee implants compared to a contemporary benchmark at 3 years post primary in women over 75 years for knee replacements with ≥250 procedures remaining at risk

Knee brand, bearing and constraint	Number at risk	Cumulative failure (%)	Difference in failure (%)	95% CI	Equivalence status	p-value
[TKR] Scorpio Cement, posterior-stabilised fixed	1,132	0.58	[REFERENCE]			
[TKR] AGC Cement, unconstrained fixed	9,844	0.75	0.17	[-0.28 , 0.63]	Non-inferiority not shown	0.229
[TKR] Advance MP Cement, unconstrained fixed	846	1.21	0.63	[-0.15 , 1.41]	Non-inferiority not shown	0.057
[TKR] Columbus Cement, unconstrained fixed	858	1.56	0.99	[0.18 , 1.80]	Inferior by ≥20%	0.009
[TKR] Genesis 2 Cement, posterior-stabilised fixed	1,628	0.96	0.39	[-0.19 , 0.96]	Non-inferiority not shown	0.095
[TKR] Genesis 2 Cement, unconstrained fixed	4,807	0.91	0.33	[-0.16 , 0.81]	Non-inferiority not shown	0.092
[TKR] Insall-Burstein 2 Cement, posterior-stabilised fixed	378	1.01	0.43	[-0.64 , 1.51]	Non-inferiority not shown	0.214
[TKR] Kinemax Cement, unconstrained fixed	1,824	0.63	0.05	[-0.50 , 0.61]	Non-inferiority not shown	0.426
[TKR] LCS Complete Cement, unconstrained mobile	1,434	1.12	0.54	[-0.11 , 1.19]	Non-inferiority not shown	0.050
[TKR] LCS Complete Uncemented, unconstrained mobile	1,444	0.97	0.39	[-0.23 , 1.02]	Non-inferiority not shown	0.110
[TKR] MRK Cement, unconstrained fixed	1,124	0.62	0.04	[-0.53 , 0.62]	Non-inferiority not shown	0.443
[TKR] NRG Cement, posterior-stabilised fixed	692	0.65	0.07	[-0.64 , 0.78]	Non-inferiority not shown	0.421
[TKR] NRG Cement, unconstrained fixed	991	0.42	-0.16	[-0.72 , 0.41]	Non-inferiority not shown	0.295
[TKR] Natural Knee II Cement, unconstrained fixed	439	0.44	-0.13	[-0.88 , 0.61]	Non-inferiority not shown	0.361
[TKR] NexGen Cement, posterior-stabilised fixed	7,284	0.84	0.27	[-0.20 , 0.73]	Non-inferiority not shown	0.129
[TKR] NexGen Cement, unconstrained fixed	5,865	0.67	0.10	[-0.37 , 0.56]	Non-inferiority not shown	0.343
[TKR] NexGen Uncemented, unconstrained fixed	259	1.09	0.51	[-0.78 , 1.80]	Non-inferiority not shown	0.220
[TKR] Optetrak Cement, posterior-stabilised fixed	253	1.46	0.88	[-0.60 , 2.37]	Non-inferiority not shown	0.121
[TKR] PFC Sigma Bicondylar Knee Cement, posterior-stabilised mobile	514	0.82	0.24	[-0.59 , 1.07]	Non-inferiority not shown	0.287
[TKR] PFC Sigma Bicondylar Knee Cement, posterior-stabilised fixed	10,390	0.80	0.22	[-0.23 , 0.68]	Non-inferiority not shown	0.167
[TKR] PFC Sigma Bicondylar Knee Cement, unconstrained fixed	21,321	0.69	0.12	[-0.32 , 0.55]	Non-inferiority not shown	0.299
[TKR] PFC Sigma Bicondylar Knee Cement, unconstrained mobile	573	1.29	0.72	[-0.22 , 1.66]	Non-inferiority not shown	0.068
[TKR] PFC Sigma Bicondylar Knee monobloc polyethylene tibia	1,500	0.82	0.24	[-0.33 , 0.81]	Non-inferiority not shown	0.205
[TKR] Profix Uncemented, unconstrained fixed	479	0.98	0.41	[-0.55 , 1.37]	Non-inferiority not shown	0.202
[TKR] Rotaglide + Cement, unconstrained mobile	276	2.03	1.46	[-0.21 , 3.12]	Non-inferiority not shown	0.043
[TKR] Scorpio Cement, unconstrained fixed	1,946	1.08	0.50	[-0.12 , 1.12]	Non-inferiority not shown	0.055
[TKR] Scorpio Uncemented, unconstrained fixed	602	1.27	0.69	[-0.28 , 1.66]	Non-inferiority not shown	0.082
[TKR] TC Plus Cement, unconstrained fixed	1,222	0.78	0.21	[-0.44 , 0.85]	Non-inferiority not shown	0.265
[TKR] TC Plus Cement, unconstrained mobile	684	0.66	0.09	[-0.63 , 0.80]	Non-inferiority not shown	0.408
[TKR] TC Plus Uncemented, unconstrained mobile	278	1.33	0.75	[-0.61 , 2.12]	Non-inferiority not shown	0.140
[TKR] Triathlon Cement, posterior-stabilised fixed	1,437	1.17	0.59	[-0.04 , 1.22]	Non-inferiority not shown	0.033
[TKR] Triathlon Cement, unconstrained fixed	4,541	1.14	0.56	[0.07 , 1.06]	Non-inferiority not shown	0.013
[TKR] Vanguard Cement, posterior-stabilised fixed	524	1.67	1.10	[0.13 , 2.06]	Inferior by ≥20%	0.013
[TKR] Vanguard Cement, unconstrained fixed	3,003	0.97	0.39	[-0.11 , 0.90]	Non-inferiority not shown	0.064
[UNI] Oxford Partial Knee Unicondylar, mobile	2,333	3.46	2.89	[2.10 , 3.68]	Inferior by ≥100%	<0.001

For peer review only

Supplementary table 30: Difference in cumulative percentage revision of knee implants compared to a contemporary benchmark at 5 years post primary in women over 75 years for knee replacements with ≥250 procedures remaining at risk

Knee brand, bearing and constraint	Number at risk	Cumulative failure (%)	Difference in failure (%)	95% CI	Equivalence status	p-value
[TKR] NexGen Cement, unconstrained fixed	3,677	0.77	[REFERENCE]			
[TKR] AGC Cement, unconstrained fixed	7,591	1.03	0.27	[-0.01 , 0.54]	Non-inferiority not shown	0.031
[TKR] Advance MP Cement, unconstrained fixed	598	2.15	1.39	[0.41 , 2.36]	Inferior by ≥20%	0.003
[TKR] Columbus Cement, unconstrained fixed	453	1.68	0.91	[0.16 , 1.67]	Inferior by ≥20%	0.009
[TKR] Genesis 2 Cement, posterior-stabilised fixed	928	1.35	0.58	[0.03 , 1.13]	Non-inferiority not shown	0.019
[TKR] Genesis 2 Cement, unconstrained fixed	2,845	1.04	0.27	[-0.06 , 0.60]	Non-inferiority not shown	0.052
[TKR] Insall-Burstein 2 Cement, posterior-stabilised fixed	349	1.55	0.79	[-0.46 , 2.03]	Non-inferiority not shown	0.109
[TKR] Kinemax Cement, unconstrained fixed	1,634	0.97	0.20	[-0.29 , 0.69]	Non-inferiority not shown	0.209
[TKR] LCS Complete Cement, unconstrained mobile	1,039	1.75	0.98	[0.30 , 1.66]	Inferior by ≥20%	0.002
[TKR] LCS Complete Uncemented, unconstrained mobile	1,043	1.21	0.44	[-0.13 , 1.01]	Non-inferiority not shown	0.064
[TKR] MRK Cement, unconstrained fixed	772	0.89	0.13	[-0.41 , 0.66]	Non-inferiority not shown	0.321
[TKR] NRG Cement, posterior-stabilised fixed	485	0.82	0.05	[-0.63 , 0.74]	Non-inferiority not shown	0.442
[TKR] NRG Cement, unconstrained fixed	626	0.56	-0.20	[-0.71 , 0.30]	Non-inferiority not shown	0.218
[TKR] Natural Knee II Cement, unconstrained fixed	348	0.44	-0.32	[-0.97 , 0.32]	Non-inferiority not shown	0.161
[TKR] NexGen Cement, posterior-stabilised fixed	4,965	1.19	0.42	[0.11 , 0.73]	Non-inferiority not shown	0.004
[TKR] PFC Sigma Bicondylar Knee Cement, posterior-stabilised mobile	363	1.55	0.78	[-0.33 , 1.89]	Non-inferiority not shown	0.083
[TKR] PFC Sigma Bicondylar Knee Cement, posterior-stabilised fixed	7,316	1.13	0.36	[0.08 , 0.64]	Non-inferiority not shown	0.006
[TKR] PFC Sigma Bicondylar Knee Cement, unconstrained fixed	14,883	0.94	0.17	[-0.06 , 0.40]	Non-inferiority not shown	0.077
[TKR] PFC Sigma Bicondylar Knee Cement, unconstrained mobile	457	1.68	0.91	[-0.10 , 1.93]	Non-inferiority not shown	0.039
[TKR] PFC Sigma Bicondylar Knee monobloc polyethylene tibia	747	1.09	0.32	[-0.21 , 0.85]	Non-inferiority not shown	0.116
[TKR] Profix Uncemented, unconstrained fixed	413	1.21	0.44	[-0.54 , 1.43]	Non-inferiority not shown	0.188
[TKR] Scorpio Cement, posterior-stabilised fixed	1,004	0.77	0.00	[-0.54 , 0.54]	Non-inferiority not shown	0.499
[TKR] Scorpio Cement, unconstrained fixed	1,668	1.45	0.69	[0.12 , 1.25]	Non-inferiority not shown	0.008
[TKR] Scorpio Uncemented, unconstrained fixed	532	1.97	1.20	[0.08 , 2.33]	Non-inferiority not shown	0.018
[TKR] TC Plus Cement, unconstrained fixed	1,097	1.20	0.44	[-0.20 , 1.07]	Non-inferiority not shown	0.090
[TKR] TC Plus Cement, unconstrained mobile	588	1.27	0.50	[-0.35 , 1.36]	Non-inferiority not shown	0.123
[TKR] Triathlon Cement, posterior-stabilised fixed	780	1.87	1.10	[0.40 , 1.81]	Inferior by ≥20%	0.001
[TKR] Triathlon Cement, unconstrained fixed	2,200	1.45	0.68	[0.31 , 1.05]	Inferior by ≥20%	<0.001
[TKR] Vanguard Cement, unconstrained fixed	1,253	1.18	0.42	[0.03 , 0.81]	Non-inferiority not shown	0.018
[UNI] Oxford Partial Knee Unicondylar, mobile	1,802	4.68	3.91	[3.09 , 4.74]	Inferior by ≥100%	<0.001

For peer review only

Supplementary table 31: Difference in cumulative percentage revision of knee implants compared to a contemporary benchmark at 7 years post primary in women over 75 years for knee replacements with ≥250 procedures remaining at risk

Knee brand, bearing and constraint	Number at risk	Cumulative failure (%)	Difference in failure (%)	95% CI	Equivalence status	p-value
[TKR] NexGen Cement, unconstrained fixed	2,110	0.98	[REFERENCE]			
[TKR] AGC Cement, unconstrained fixed	4,857	1.28	0.30	[-0.04 , 0.65]	Non-inferiority not shown	0.043
[TKR] Advance MP Cement, unconstrained fixed	383	2.15	1.17	[0.18 , 2.16]	Non-inferiority not shown	0.010
[TKR] Genesis 2 Cement, posterior-stabilised fixed	452	1.99	1.01	[0.15 , 1.87]	Non-inferiority not shown	0.011
[TKR] Genesis 2 Cement, unconstrained fixed	1,415	1.11	0.13	[-0.25 , 0.51]	Non-inferiority not shown	0.252
[TKR] Insall-Burstein 2 Cement, posterior-stabilised fixed	295	1.55	0.57	[-0.69 , 1.83]	Non-inferiority not shown	0.188
[TKR] Kinemax Cement, unconstrained fixed	1,416	1.23	0.24	[-0.33 , 0.82]	Non-inferiority not shown	0.201
[TKR] LCS Complete Cement, unconstrained mobile	653	1.85	0.87	[0.14 , 1.60]	Non-inferiority not shown	0.010
[TKR] LCS Complete Uncemented, unconstrained mobile	591	1.44	0.46	[-0.22 , 1.13]	Non-inferiority not shown	0.092
[TKR] MRK Cement, unconstrained fixed	494	1.03	0.05	[-0.57 , 0.66]	Non-inferiority not shown	0.440
[TKR] NRG Cement, unconstrained fixed	295	0.56	-0.42	[-0.95 , 0.11]	Non-inferior	0.062
[TKR] Natural Knee II Cement, unconstrained fixed	251	0.84	-0.14	[-1.16 , 0.87]	Non-inferiority not shown	0.391
[TKR] NexGen Cement, posterior-stabilised fixed	2,942	1.54	0.56	[0.16 , 0.96]	Non-inferiority not shown	0.003
[TKR] PFC Sigma Bicondylar Knee Cement, posterior-stabilised fixed	4,344	1.31	0.33	[-0.01 , 0.67]	Non-inferiority not shown	0.029
[TKR] PFC Sigma Bicondylar Knee Cement, unconstrained fixed	9,161	1.05	0.07	[-0.22 , 0.36]	Non-inferiority not shown	0.320
[TKR] PFC Sigma Bicondylar Knee Cement, unconstrained mobile	314	1.95	0.96	[-0.18 , 2.11]	Non-inferiority not shown	0.050
[TKR] PFC Sigma Bicondylar Knee monobloc polyethylene tibia	252	1.43	0.45	[-0.42 , 1.31]	Non-inferiority not shown	0.156
[TKR] Profix Uncemented, unconstrained fixed	300	1.21	0.23	[-0.77 , 1.23]	Non-inferiority not shown	0.327
[TKR] Scorpio Cement, posterior-stabilised fixed	842	0.97	-0.01	[-0.64 , 0.61]	Non-inferiority not shown	0.482
[TKR] Scorpio Cement, unconstrained fixed	1,272	1.65	0.67	[0.04 , 1.29]	Non-inferiority not shown	0.018
[TKR] Scorpio Uncemented, unconstrained fixed	350	1.97	0.99	[-0.15 , 2.12]	Non-inferiority not shown	0.044
[TKR] TC Plus Cement, unconstrained fixed	824	1.29	0.31	[-0.37 , 0.99]	Non-inferiority not shown	0.184
[TKR] TC Plus Cement, unconstrained mobile	456	1.27	0.29	[-0.58 , 1.16]	Non-inferiority not shown	0.257
[TKR] Triathlon Cement, posterior-stabilised fixed	260	2.31	1.33	[0.38 , 2.27]	Inferior by ≥20%	0.003
[TKR] Triathlon Cement, unconstrained fixed	665	1.51	0.52	[0.11 , 0.94]	Non-inferiority not shown	0.007
[TKR] Vanguard Cement, unconstrained fixed	366	1.39	0.41	[-0.10 , 0.92]	Non-inferiority not shown	0.059
[UNI] Oxford Partial Knee Unicondylar, mobile	1,146	6.42	5.44	[4.38 , 6.49]	Inferior by ≥100%	<0.001

For peer review only

Supplementary table 32: Difference in cumulative percentage revision of knee implants compared to a contemporary benchmark at 10 years post primary in women over 75 years for knee replacements with ≥250 procedures remaining at risk

Knee brand, bearing and constraint	Number at risk	Cumulative failure (%)	Difference in failure (%)	95% CI	Equivalence status	p-value
[TKR] PFC Sigma Bicondylar Knee Cement, unconstrained fixed	2,749	1.29	[REFERENCE]			
[TKR] AGC Cement, unconstrained fixed	1,448	1.48	0.19	[-0.15 , 0.54]	Non-inferiority not shown	0.136
[TKR] Genesis 2 Cement, unconstrained fixed	280	1.41	0.12	[-0.36 , 0.61]	Non-inferiority not shown	0.310
[TKR] Kinemax Cement, unconstrained fixed	691	1.55	0.27	[-0.40 , 0.93]	Non-inferiority not shown	0.216
[TKR] NexGen Cement, posterior-stabilised fixed	851	1.77	0.48	[0.07 , 0.90]	Non-inferiority not shown	0.011
[TKR] NexGen Cement, unconstrained fixed	447	1.08	-0.21	[-0.56 , 0.14]	Non-inferior	0.117
[TKR] PFC Sigma Bicondylar Knee Cement, posterior-stabilised fixed	1,202	1.46	0.18	[-0.15 , 0.50]	Non-inferiority not shown	0.148
[TKR] Scorpio Cement, posterior-stabilised fixed	305	1.72	0.44	[-0.54 , 1.41]	Non-inferiority not shown	0.191
[TKR] Scorpio Cement, unconstrained fixed	449	2.07	0.78	[0.04 , 1.52]	Non-inferiority not shown	0.019
[UNI] Oxford Partial Knee Unicondylar, mobile	356	8.61	7.32	[5.87 , 8.78]	Inferior by ≥100%	<0.001

For peer review only

The RECORD statement – checklist of items, extended from the STROBE statement, that should be reported in observational studies using routinely collected health data.

	Item No.	STROBE items	Location in manuscript where items are reported	RECORD items	Location in manuscript where items are reported
**Title and abstract**					
	1	(a) Indicate the study's design with a commonly used term in the title or the abstract (b) Provide in the abstract an informative and balanced summary of what was done and what was found	Title page 1 and abstract page 2	RECORD 1.1: The type of data used should be specified in the title or abstract. When possible, the name of the databases used should be included.<br><br>RECORD 1.2: If applicable, the geographic region and timeframe within which the study took place should be reported in the title or abstract.<br><br>RECORD 1.3: If linkage between databases was conducted for the study, this should be clearly stated in the title or abstract.	Title, abstract page 2.<br><br>N/A
**Introduction**					
Background rationale	2	Explain the scientific background and rationale for the investigation being reported	Introduction Page 4 and 5		
Objectives	3	State specific objectives, including any prespecified hypotheses	Introduction Page 5		
**Methods**					
Study Design	4	Present key elements of study design early in the paper	Methods pages 6 to 7		
Setting	5	Describe the setting, locations, and relevant dates, including periods of recruitment, exposure, follow-up, and data collection	Methods page 6		

For peer review only

Participants	6	*(a) Cohort study* - Give the eligibility criteria, and the sources and methods of selection of participants. Describe methods of follow-up<br>*Case-control study* - Give the eligibility criteria, and the sources and methods of case ascertainment and control selection. Give the rationale for the choice of cases and controls<br>*Cross-sectional study* - Give the eligibility criteria, and the sources and methods of selection of participants<br><br>*(b) Cohort study* - For matched studies, give matching criteria and number of exposed and unexposed<br>*Case-control study* - For matched studies, give matching criteria and the number of controls per case	Methods page 6	RECORD 6.1: The methods of study population selection (such as codes or algorithms used to identify subjects) should be listed in detail. If this is not possible, an explanation should be provided.<br><br>RECORD 6.2: Any validation studies of the codes or algorithms used to select the population should be referenced. If validation was conducted for this study and not published elsewhere, detailed methods and results should be provided.<br><br>RECORD 6.3: If the study involved linkage of databases, consider use of a flow diagram or other graphical display to demonstrate the data linkage process, including the number of individuals with linked data at each stage.	Methods Page 6, supplemental figure 1<br><br>N/A<br><br>N/A
Variables	7	Clearly define all outcomes, exposures, predictors, potential confounders, and effect modifiers. Give diagnostic criteria, if applicable.	Methods page 6 and 7	RECORD 7.1: A complete list of codes and algorithms used to classify exposures, outcomes, confounders, and effect modifiers should be provided. If these cannot be reported, an explanation should be provided.	Methods page 6 (description of data selection, no codes)
Data sources/ measurement	8	For each variable of interest, give sources of data and details of methods of assessment (measurement).<br>Describe comparability of assessment methods if there is more than one group	Methods Page 6		
Bias	9	Describe any efforts to address			

For peer review only

		potential sources of bias			
Study size	10	Explain how the study size was arrived at	Supplemental figure 1		
Quantitative variables	11	Explain how quantitative variables were handled in the analyses. If applicable, describe which groupings were chosen, and why	Methods Page 6 and 7		
Statistical methods	12	(a) Describe all statistical methods, including those used to control for confounding<br>(b) Describe any methods used to examine subgroups and interactions<br>(c) Explain how missing data were addressed<br>(d) *Cohort study* - If applicable, explain how loss to follow-up was addressed<br>*Case-control study* - If applicable, explain how matching of cases and controls was addressed<br>*Cross-sectional study* - If applicable, describe analytical methods taking account of sampling strategy<br>(e) Describe any sensitivity analyses	Methods Page 6 and 7		
Data access and cleaning methods		..		RECORD 12.1: Authors should describe the extent to which the investigators had access to the database population used to create the study population.<br><br>RECORD 12.2: Authors should provide information on the data cleaning methods used in the study.	Acknowledgement section, page 21

For peer review only

					Methods Page 6
Linkage		..		RECORD 12.3: State whether the study included person-level, institutional-level, or other data linkage across two or more databases. The methods of linkage and methods of linkage quality evaluation should be provided.	No linkage
**Results**					
Participants	13	(a) Report the numbers of individuals at each stage of the study (*e.g.*, numbers potentially eligible, examined for eligibility, confirmed eligible, included in the study, completing follow-up, and analysed)<br>(b) Give reasons for non-participation at each stage.<br>(c) Consider use of a flow diagram	Supplemental figure 1 and Results page 9	RECORD 13.1: Describe in detail the selection of the persons included in the study (*i.e.,* study population selection) including filtering based on data quality, data availability and linkage. The selection of included persons can be described in the text and/or by means of the study flow diagram.	Methods; Results page 9 and supplemental figure 1
Descriptive data	14	(a) Give characteristics of study participants (*e.g.*, demographic, clinical, social) and information on exposures and potential confounders<br>(b) Indicate the number of participants with missing data for each variable of interest<br>(c) *Cohort study* - summarise follow-up time (*e.g.*, average and total amount)			
Outcome data	15	*Cohort study* - Report numbers of outcome events or summary measures over time<br>*Case-control study* - Report numbers in each exposure category, or summary measures of exposure	All supplemental tables		

For peer review only

		Cross-sectional study - Report numbers of outcome events or summary measures			
Main results	16	(a) Give unadjusted estimates and, if applicable, confounder-adjusted estimates and their precision (e.g., 95% confidence interval). Make clear which confounders were adjusted for and why they were included<br>(b) Report category boundaries when continuous variables were categorized<br>(c) If relevant, consider translating estimates of relative risk into absolute risk for a meaningful time period	Results Pages 9 to 17, embedded tables and Supplemental tables		
Other analyses	17	Report other analyses done—e.g., analyses of subgroups and interactions, and sensitivity analyses	Results Page 17		
**Discussion**					
Key results	18	Summarise key results with reference to study objectives	Discussion Page 18		
Limitations	19	Discuss limitations of the study, taking into account sources of potential bias or imprecision. Discuss both direction and magnitude of any potential bias	Discussion page 19	RECORD 19.1: Discuss the implications of using data that were not created or collected to answer the specific research question(s). Include discussion of misclassification bias, unmeasured confounding, missing data, and changing eligibility over time, as they pertain to the study being reported.	Discussion Page 19
Interpretation	20	Give a cautious overall interpretation of results considering objectives, limitations, multiplicity of analyses, results from similar	Discussion Pages 18-19		

For peer review only

		studies, and other relevant evidence			
Generalisability	21	Discuss the generalisability (external validity) of the study results	Discussion Page 19/20		
**Other Information**					
Funding	22	Give the source of funding and the role of the funders for the present study and, if applicable, for the original study on which the present article is based	Page 21		
Accessibility of protocol, raw data, and programming code		..	N/A	RECORD 22.1: Authors should provide information on how to access any supplemental information such as the study protocol, raw data, or programming code.	N/A

*Reference: Benchimol EI, Smeeth L, Guttmann A, Harron K, Moher D, Petersen I, Sørensen HT, von Elm E, Langan SM, the RECORD Working Committee. The REporting of studies Conducted using Observational Routinely-collected health Data (RECORD) Statement. *PLoS Medicine* 2015; https://doi.org/10.1371/journal.pmed.1001885.

*Checklist is protected under Creative Commons Attribution (CC BY) license.

For peer review only
